# Dynamic evolution of bitter taste receptor genes in vertebrates

**DOI:** 10.1186/1471-2148-9-12

**Published:** 2009-01-15

**Authors:** Dong Dong, Gareth Jones, Shuyi Zhang

**Affiliations:** 1 School of Life Sciences, East China Normal University, Shanghai, PR China; 2 School of Biological Sciences, University of Bristol, Bristol, UK

## Abstract

**Background:**

Sensing bitter tastes is crucial for many animals because it can prevent them from ingesting harmful foods. This process is mainly mediated by the bitter taste receptors (T2R), which are largely expressed in the taste buds. Previous studies have identified some T2R gene repertoires, and marked variation in repertoire size has been noted among species. However, the mechanisms underlying the evolution of vertebrate T2R genes remain poorly understood.

**Results:**

To better understand the evolutionary pattern of these genes, we identified 16 T2R gene repertoires based on the high coverage genome sequences of vertebrates and studied the evolutionary changes in the number of T2R genes during birth-and-death evolution using the reconciled-tree method. We found that the number of T2R genes and the fraction of pseudogenes vary extensively among species. Based on the results of phylogenetic analysis, we showed that T2R gene families in teleost fishes are more diverse than those in tetrapods. In addition to the independent gene expansions in teleost fishes, frogs and mammals, lineage-specific gene duplications were also detected in lizards. Furthermore, extensive gains and losses of T2R genes were detected in each lineage during their evolution, resulting in widely differing T2R gene repertoires.

**Conclusion:**

These results further support the hypotheses that T2R gene repertoires are closely related to the dietary habits of different species and that birth-and-death evolution is associated with adaptations to dietary changes.

## Background

Taste perception refers to sensations triggered by taste buds on the surface of the tongue, which sense sweet, salty, sour, bitter and umami flavors. These gustatory senses are closely related to an animal's diet and external environments [1-3]. Most taste sensations are triggered via receptor-based sensors expressed in different taste-cell types [4-6]. Among these chemical senses, bitter tastes are particularly important because many poisonous substances tend to be bitter, and bitter taste perception can allow animals to detect and avoid toxins in food [7]. This process is mainly mediated by bitter taste receptors (T2R) which are encoded by T2R genes.

T2R genes belong to one type of G-protein-coupled receptors (GPCR) which are characterized by their seven conserved transmembrane regions [8]. T2Rs are the largest family of taste receptors, which bind to tastants. T2R genes contain an average of 300 codons, and there are no introns in their coding regions, making them easy to detect in whole-genome sequences. Like olfactory and other chemosensory receptor genes, T2R genes also form a multi-gene family and display high sequence similarity [9,10]. T2R genes are not randomly distributed through chromosomes; instead they tend to cluster together in a few specific genomic regions, perhaps corresponding to their generation by tandem gene duplications. For example, human T2R genes are mainly located in chromosomes 7 and 12, and mouse T2R genes are concentrated in chromosomes 6 and 15 [10,11].

In previous studies, T2R gene repertoires have been described in some mammals, chickens, frogs and some teleost fishes [9-18]. With the availability of whole-genome sequences of animals, T2R genes can be detected by applying data-mining methods. The nearly complete human and mouse T2R gene repertoires have been reported by Conte et al. [9,18] and Shi et al. [10]. Although these studies used different methods, their results were similar. Conte and colleagues [9,18] identified 34 and 40 T2R genes in human and mouse genomes, respectively, and Shi and co-workers [10] identified 33 and 36 T2R genes, respectively. Other vertebrate T2R gene receptors have also been identified [15,16], for example, those of rat, dog, opossum, chicken and some teleost fishes. By analyzing the low-coverage genome sequence, T2R genes have also been identified in the cow. Owing to the different data-mining criteria used for the identification of T2R genes, results have differed among studies. For example, Shi et al [16] identified 64 T2R genes in the frog, whereas Go [15] only identified only 54 T2R genes. Furthermore, some studies focused on identification of G-protein-coupled receptors (GPCRs) from genome sequences [19-21]; however, not all GPCR-encoding genes are T2R genes, they also include other gene families, such as olfactory receptor (OR) and vomeronasal pheromone (VR) receptor genes. These studies also distinguished T2R genes from non-T2R GPCR genes. In Additional file 1, we list the sizes of the T2R gene repertoires that have been well documented from a range of species. These results indicate that the number of T2R genes shows extensive variation among taxa. For example, the chicken and zebrafish have only 3 and 4 intact T2R genes, respectively, whereas the frog (*Xenopus tropicalis*) has nearly 50 intact T2R genes. The number of intact genes in the frog is therefore about 16 times greater than in the chicken and 12 times greater than in the zebrafish. Furthermore, massive pseudogenization has occurred in some species. These observations indicated that ability to bitter taste might be largely determined by the number of functional genes. Bitter taste perception in animals is tightly coupled with diet and habitats. Differences in gene family size are due to lineage-specific gene duplications and losses in vertebrates, which represents an extreme form of birth-and-death evolution [15]. Therefore, it is interesting to study the pattern of gains and losses of T2R genes during vertebrate evolution.

Go has previously predicted the numbers of ancestral T2R genes using the linearized tree method [15], and identified some lineage-specific gene expansions and contractions that occurred throughout vertebrate evolution. However, this method did not characterize the gains and losses of T2R genes in detail, especially in mammals. To gain further insight into the evolutionary dynamics of T2R genes, we identified more T2R gene repertoires in vertebrates, and used the reconciled-tree method to study evolutionary changes in T2R gene families [22-24]. In this study, we provide a more comprehensive view of birth-and-death processes involving T2R genes during the evolution of vertebrates, and suggest that this approach might further improve our understanding of bitter taste sensitivities.

## Results

### T2R gene repertoires

T2R gene repertoires have previously been described in some vertebrates [10,15,16,19-21]. In see Additional file 1, we list the documented sizes of T2R gene repertoires in several species. Here, we have updated these results using the most recent versions of the genome sequences to obtain more accurate results. Furthermore, we have identified the T2R gene repertories of the rhesus macaque (*Macaca mulatta*), horse (*Equus caballus*), platypus (*Ornithorhynchus anatinus*), lizard (*Anolis carolinensis*) and stickleback (*Gasterosteus aculeatus*) for the first time; Table 1 shows the number of T2R genes identified for these species.

**Table 1:** Numbers of T2R genes and pseudogenes in vertebrates and their chromosomal/contig locations^a^

Species		Intact	Partial	Pseudo	Total	Fraction of pseudogenes	Chromosome/contig locations
Mammal	Human	24	0	10	34	28%	chr5,chr7,chr12
	Macaque	26	1	11	38	29%	chr3,chr6,chr9,chr11,chrUn
	Mouse	33	0	9	42	21%	chr2,chr6,chr15
	Rat	36	1	5	42	12%	chr2,chr3,chr4
	Dog	15	0	5	20	25%	chr14,chr16,chr27,chr34
	Cow	18	0	15	33	45%	chr4,chr5,chr20,chrUn
	Horse	19	0	36	55	65%	chr4,chr6,chr21,chrUn
	Opossum	26	3	7	36	19%	chr2,chr3,chr7,chr8
	Platypus	4	1	1	6	16%	chr4,Ultra450,Contig68031,Contig12097
Bird	Chicken	3	0	0	3	0	chr1,chr3
Reptiles	Lizard	37	2	10	49	20%	
Amphibians	Frog	49	3	14	66	21%	
Teleost fishes	Fugu	4	0	0	4	0	chrUn
	Puffer fish	6	0	0	6	0	chr2,chr12
	Stickleback	3	0	0	3	0	chrXVI,chrXIII
	Zebrafish	4	0	0	4	0	chr8,chr9

^a ^Genome sequences of frog and lizard are not assembled into chromosomes so we do not list their scaffold locations.

Many genome sequences are still incomplete, so we must be aware of the risk that some of the T2R gene repertoires are also incomplete. Using recently available high coverage of genome sequences, most of our results are consistent with previous reports [15,16], indicating that the T2R gene repertoires identified are nearly complete. However, some genome sequences were still unassembled into chromosomes, and short contigs/scaffolds might lead to an underestimate of the number of T2R genes. Therefore, we also identified partial T2R genes from draft genome sequences, and these might contain many potential T2R genes. Furthermore, we have also updated some previously incomplete results. Therefore, we believe that we have identified a nearly complete T2R gene repertoire for each species. For example, Shi et al. [16] found 12 intact genes and 7 partial genes using the low-coverage of the cow genome (3 × coverage), whereas we identified 18 intact cow T2R genes. The discrepancy between the two results could be because we used different versions of the genome sequence or be due to the different T2R data-mining criteria used in the two studies. Fischer et al. [25] reported the T2R repertoire of the rhesus macaque using molecular cloning methods and identified 22 intact T2R genes and the fraction of pseudogenes was about 15%. In our results, 26 intact T2R genes were identified and the fraction of pseudogenes was about 29% in the rhesus macaque, which is close to the estimate for humans. The horse and cow are both herbivorous animals, and we identified 19 intact T2R genes in the horse. The fractions of pseudogenes in the cow and horse are much higher than those in other vertebrates (Table 1). Go has previously suggested that gene expansion occurred in the mammalian lineage [15], but we found only a small T2R gene repertoire (four intact genes) in the platypus. The genome sequence of a lizard (*Anolis carolinensis*), a common reptile found in the southeastern United States, has recently become available and a total of 49 T2R genes were identified, including 37 intact genes, 2 partial genes and 10 pseudogenes. Both the frog and the lizard have a large number of intact T2R genes. The DNA sequences of all T2R genes from 16 vertebrates are shown in Additional file 1.

### Phylogenetic analysis

**Figure 1:**
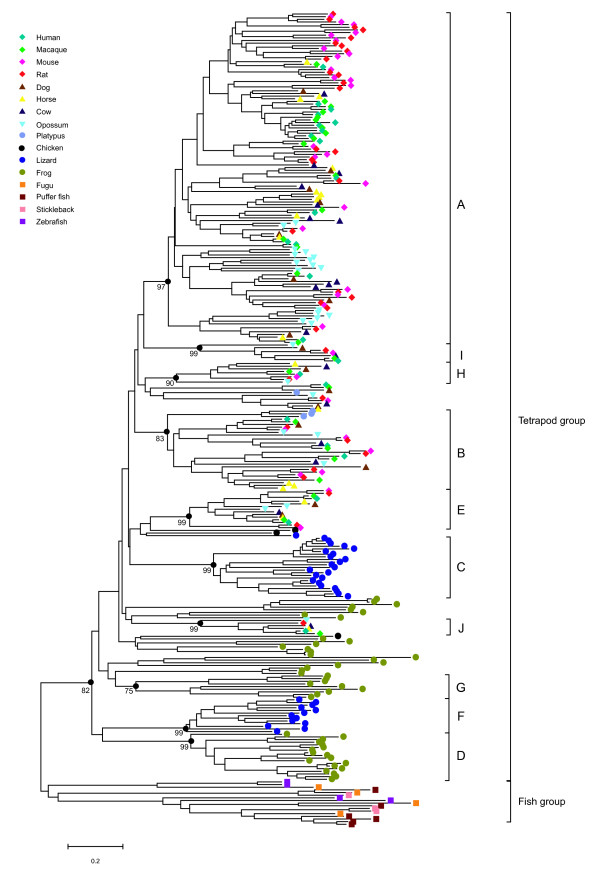
**Phylogenetic tree of 307 T2R genes in 16 vertebrates**. A branch specific to each species is indicated according to the color code at the top left. The bootstrap value obtained from 500 replicates is shown for the families within each group.

To investigate the evolutionary relationships among intact T2R genes, we constructed a neighbor-joining tree [26] for 307 amino acid sequences of intact genes identified from 16 vertebrates (Fig. 1). We did not use the partial T2R genes and pseudogenes because most of them were much shorter than intact genes. The results suggested a major divergence between the intact T2R genes of teleost fishes and tetrapods. The T2R genes in tetrapod vertebrates form a monophyletic clade with high bootstrap support (82%); the genes in this clade were classed as the 'tetrapod group' and other genes in teleost fishes as the 'fish group'. The genes in the tetrapod group can be further manually divided into different phylogenetic families, each of which contains more than five genes and is supported by a bootstrap value >70%. If some smaller families were nested within a larger family, we selected the larger one and ignored the smaller families. Using this approach, we identified ten gene families, most of which had bootstrap values >90%, labeled A-J according to the number of the genes in each family. As shown in Figure 1, mammalian T2R genes can be subdivided into several phylogenetic families that are distinct from the frog and lizard lineage-specific families.

Most of the T2R genes are organized into clusters on specific chromosomes [10,11,16]. For example, T2R genes in the mouse and rat are mainly located on chromosomes 6 and 4, respectively. To describe the relationship between the genes in specific phylogenetic families and genomic distributions, we compared chromosomal distributions for each family containing more than two genes. Owing to the lack of chromosome information in frogs and lizards so far, we also compared their scaffold distributions. As shown in Table 2, T2R genes belonging to the same family are always located together on the same chromosome/scaffolds which is in agreement with the expansion of T2R gene families by tandem gene duplication.

**Table 2:** Number of T2R genes in each family.

Families	Species	Chromosomal/Scaffold location	Number of genes
A	Human	Chr12	14(15)
	Macaque	Chr11	14(16)
	Mouse	Chr6	23(23)
	Rat	Chr4	25(25)
	Cow	Chr5	10(11)
	Horse	Chr6	10(11)
	Dog	Chr27	6(7)
	Opossum	Chr2,chr8	9,6(17)
C	Lizard	Scaffold_206,Scaffold_662	13,8(23)
B	Macaque	Chr3	4(4)
	Mouse	Chr6	4(5)
	Rat	Chr4	4(5)
	Horse	Chr4	3(3)
	Opossum	Chr8	3(3)
G	Frog	Scaffold_672	8(9)
F	Lizard	Scaffold_19	7(13)
D	Frog	Scaffold_672	10(18)

Only the families that contained at least three genes in each species are counted.

It has been reported that the evolution of vertebrate T2R genes is characterized by rapid turnover and species-specificity [10,15,16]. The phylogenetic tree showed that vertebrate T2R genes can be clearly divided into different, divergent families. Shi et al. described lineage-specific gene duplications in teleost fishes, frogs and mammals [16]. As shown in Figure 1, lizard T2R genes form two separate lizard-specific families with high bootstrap support (99%), perhaps indicating that multiple recent gene duplications occurred in lizards. It is also known that there are lineage-specific gene expansions in mammals. One might wonder about the mechanisms underpinning the form of birth-and-death evolution, especially in mammals, and we describe a scenario for the evolution of T2R genes.

### Evolutionary changes in the number of T2R genes

The number of T2R genes is variable among different species. This variability indicates that some lineages have gained additional functional genes through gene duplication or that these genes have been lost from specific lineages. To understand the evolutionary changes in the number of T2R genes, we estimated the gains and losses of the T2R genes in a diverse range of vertebrates using the reconciled-tree method [22-24]. The resulting phylogenetic tree (Figure 1) shows lineage-specific families in teleost fishes, frogs, lizards and mammals, indicating that the T2R genes underwent independent gene expansions. The divergence time among these vertebrate species is so long (>450 million years ago [MYA]) that it is difficult to accurately estimate the number of ancestral genes with a deep divergence based on the parsimony principle. In this study, we therefore mainly focused on mammals.

**Figure 2:**
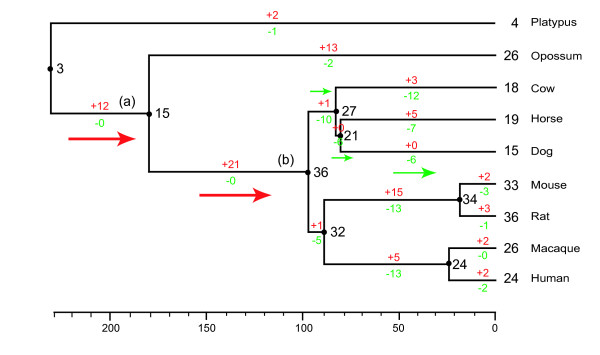
**Evolutionary changes in the number of T2R genes in mammals**. The phylogenetic tree and the divergence times were obtained from Murphy et al. [28] and Glazko et al. [29]. The numbers to the right of each black dot indicate the numbers of genes in the common ancestral species. The numbers below or above each branch indicate the numbers of gene gains and losses, respectively. The red arrows represent gene expansions and the green arrows represent gene contractions.

First, we reconstructed a phylogenetic tree of all T2R genes in mammals and used mouse VR genes as outgroups [27]. The species tree was constructed according to the mammalian phylogeny principle reported by Murphy et al. [28] and Glazko et al. [29] and we then estimated the number of T2R genes in common ancestors. It is difficult to verify the real phylogenetic relationships of genes with low bootstrap values, so we generated a condensed tree with 50% bootstrap values. Figure 2 shows the estimated number of T2R genes in the common ancestors of mammals. In general, the principle of parsimony uses the simplest hypothesis, so the numbers of ancestral genes may be underestimated. Therefore, it is necessary to compare our results with those of previous studies that used different methods. The estimated number of genes in the common ancestor of placental mammals and marsupials in our study (~15 genes) is similar to that estimated in a previous study (16–17 genes) [15] that used the linearized-tree method. Therefore, we believe that, despite some limitations, our results broadly reflect the trends in gains and losses of T2R genes in mammals.

Our results showed that lineage-specific gene expansions and contractions are common in the branches leading to different mammal lineages. Figure 2 shows evolutionary changes in the number of T2R genes in mammals. We can clearly identify two extensive gene expansion events that occurred at two distinct times in evolution (branch a and branch b in Figure 2). The first occurred after the divergence of monotremes and placentals/marsupials, and the number of T2R genes increased almost five-fold in the mammalian lineages. The other gene expansion occurred in the branch of the common ancestor of all mammals including placentals, which is consistent with the observation previously been reported by Go [15]. We found that the common ancestor of placental mammals had one of the largest number of T2R genes. Furthermore, there was a lineage-specific expansion in the opossum lineage, which might be important for a taxon-specific sense of bitter taste. Primates and rodents are often omnivorous, but their functional T2R genes also underwent markedly different evolutionary changes, indicating many births and deaths of genes.

Moreover, multiple T2R gene losses have previously been reported to have occurred in the cow, horse and dog, all of which are of the superorder Laurasiatheria [28] ; however, there was no evidence for gene gains in the dog lineage. The phylogenetic relationships among Cetartiodactyla, Perissodactyla and Carnivora remain unclear [30], but we postulate that Cetartiodactyla and Perissodactyla are sister groups, as suggested by Waddell et al. [31] and conducted the same analysis (see Additional file 2); indeed, in a similar analysis, we identified a similar number of gene gains and losses. In this analysis, we also identified gene contractions in the dog, cow and horse.

Extensive independent gene expansions occurred in teleost fishes, frogs and lizards, which might indicate marked differences in the bitter taste sensitivities of these species. We also performed the same analysis of all 16 vertebrate T2R gene repertoires (see Additional file 3) using a conventional vertebrate phylogeny approach [32]. The results showed slight changes in the number of T2R genes in the evolution of teleost fishes and massive gene expansions in the frog and lizard. However, we did not find gene contractions in the chicken.

## Discussion

In the present study, we identified and updated T2R gene repertoires from a wide range of vertebrate taxa. Among these taxa, T2R genes in the rhesus macaque, horse, platypus, lizard and stickleback were reported for the first time based on currently available genome sequences. Data-mining methods based on high-coverage genome sequences are considered as a reliable method to detect T2R gene repertoires, and most previous studies have identified nearly complete gene repertoires. Furthermore, to avoid missing potential T2R genes, we also validated each of the Blast-hit sequences and identified partial T2R genes. Therefore, our methods should have provided almost complete coverage of T2R gene repertoires, although some genome sequences may still be incomplete.

To better understand the evolutionary dynamics of vertebrate T2R genes, we also performed an evolutionary analysis of the T2R gene repertoires from 16 vertebrates. The results indicated that there are frequent changes in the number of genes and there have been extensive gains and losses of T2R genes during vertebrate evolution. It should be mentioned that the number of genes in common ancestors is likely to be underestimated using the reconciled-tree method. Our estimated number of genes in the common ancestor of placentals and marsupials is similar to that reported by Go [15], which indicates that our results provide a reliable estimate of the gains and losses of T2R genes in mammals.

It has long been assumed that bitter taste evolved as a defense mechanism to detect potentially harmful toxins in food. Functional studies have shown that T2R proteins respond to bitter tastants and bitter taste reception is likely to associate with dietary selection [1,33]. Species-specific T2Rs might be required for animals to detect distinct bitter substances. For example, teleost fishes have only a small number of T2R genes, which show a low level of sequence similarity with those of tetrapods; in addition, the frog and lizard contain no mammalian-like gene family members and their T2R likely evolved in a lineage-specific manner. The platypus has one of the smallest T2R repertoires in mammals, which might be due to the semi-aquatic survival environment and diet (such as underwater crustaceans) where it seldom tastes bitter compondes [34]. It has often been assumed that any food that tastes bitter should be toxic. Glendinning and co-workers [2] have reported that bitter substances are unequally distributed in animal foods, and that plants contain more bitter constituents. Therefore, herbivorous and omnivorous mammals would be expected to need a greater level of bitter taste rejection compared with carnivores, which is in agreement with our results that the dog has the smallest T2R gene repertoire size among placental mammals. In addition, we also identified the T2R genes in the cat from its low-coverage genome sequence [35] (three intact genes, four partial genes and four pseudogenes, data not shown), which supports the hypothesis that T2R genes are less diverse in carnivores.

Surprisingly, our results show that the cow and horse have a larger fraction of pseudogenes compared with primates and rodents. It is possible that T2R genes lost functionality due to artificial selection or genetic drift during the domestication of cows and horses, resulting in extensive pseudogenization. So, vertebrate T2R gene repertoire size might be greatest in herbivorous taxa, with reduced numbers found in omnivorous, carnivorous and aquatic animals, respectively; however, this is speculative and needs further analysis. It would be interesting to examine the sense of bitter taste in more animals with a wide range of feeding habits.

As environments change, the feeding behavior of animals will also evolve [1]. Therefore, animals need to adapt their T2R to foods found in markedly changeable circumstances, possibly resulting in extensive birth-and-death evolution of T2R genes. In addition to the marked gene divergence among teleost fishes, frog, lizard and mammals, massive gene expansions and contractions were observed in the evolution of several mammal lineages. The T2R gene repertoires in teleost fishes seem to have diverged substantially from those of tetrapods, which indicated that there were few T2R genes before the separation of teleost fish and tetrapods. Regarding genetic changes during bird evolution, we obtained quite different results from those previously reported by Go [15]. Go concluded that the chicken lost T2R genes during evolution, whereas we identified no marked changes in the lineage leading to the chicken. Indeed, our results question whether birds lost T2R genes after their divergence from reptiles (~250 MYA) [36]. To answer this question, analysis of more bird T2R gene repertoires is needed.

## Conclusion

Investigation of the T2R gene repertoires in vertebrates has revealed extensive birth-and-death evolution in this study. One caveat of this analysis in vertebrates is that there are few representative amphibian, reptile and bird lineages, so the number of genes in the common ancestors of these species might be underestimated. In the future, analyses of additional species would be helpful to describe the dynamic evolution of T2R genes. Nevertheless, our results provide a new perspective on the relationships among T2R gene repertoires and different survival circumstances, and help explain how bitter taste reception has evolved.

## Methods

### Genome sequence data

In addition to the previously reported T2R genes, we identified six new T2R gene repertories in this study (rhesus macaque, cow, horse, platypus, lizard, and stickleback). The draft genome sequence of rhesus macaque [37] (*Macaca mulatta*; rheMac2, released in Jan. 2006; 5.1 × coverage), rat [38] (*Rattus norvegicus*; rn4, released in Nov. 2004; 7 × coverage), cow (*Bos taurus*; bosTau2, released in Aug. 2006; 7 × coverage), horse (*Equus caballus*; EquCab1, released in Jan. 2007; 6.8 × coverage), dog [39] (*Canis familiaris*; canFam2, released in May. 2005; 7.6 × coverage), opossum (*Monodelphis domestica*; monDom4, released in Jan. 2006; 6.5 × coverage), platypus [40] (*Ornithorhynchus anatinus*; v5.0.1, released in Mar. 2007; 6 × coverage), chicken (*Gallus gallus*; galGal3, released in May. 2006; 6.6 × coverage), lizard (*Anolis carolinensis*; AnoCar 1.0, released in Feb. 2007; 6.8 × coverage), frog (*Xenopus tropicalis*; xenTro2, released in Aug. 2005; 7.6 × coverage), fugu (*Takifugu rubripes*; fr2, released in Oct. 2004; 8.5 × coverage), zebrafish (*Danio rerio*; danRer5, released in Jul. 2007; 9 × coverage), puffer fish (*Tetraodon nigroviridis*; tetNig1, released in Feb. 2004; 7.9 × coverage) and Stickleback (*Gasterosteus aculeatus*; gasAcu1, released in Feb. 2006; 6 × coverage), were downloaded from UCSC Genome Bioinformatics Website .

### T2R gene identification

We identified T2R genes from each vertebrate species using the following methods. We first collected previously published T2R gene sequences from human, mouse, dog, opossum, chicken, frog and zebrafish genomes as query sequences. Next, we conducted a TBLASTN [41] search using the E-value 1e-10 against each genome sequence. There were so many TBLASTN query results that hit the same genomic region that we extracted non-overlapping sequences, each of which showed the lowest E-value among the hits to a given region. Functional, intact T2R genes were identified from these blast-hit sequences using the following approach. First, we collected the blast-hits that were >100 amino acids long. Then, each of the blast-hit sequences was extended in both 3' and 5' directions along the genome sequences. Obtained sequences were confirmed by BLASTP searches against the NCBI databases to ensure that genuine T2R genes were identified. Finally, the coding sequences with proper ATG and the stop codon were extracted (the average functional T2R gene was ~300 amino acids long). Sequences that contained interrupting stop codons or frameshifts were regarded as pseudogenes and the remaining sequences containing either initiation codons or stop codons were considered partial T2R genes.

### Phylogenetic analysis

The translated amino-acid sequences were aligned using the program FFT-NS-I nested in Mafft version 5 [42]. The phylogenetic T2R gene tree (Figure 1) was constructed using MEGA3 software [43] and the Neighbor-Joining [26] method with the protein JTT matrix model, and was evaluated by 500 bootstrap replications.

### The reconciled-tree method

The processes of gene gains and losses can result in incongruence between the topologies of gene trees and species trees. An alternative approach is to investigate the relationship between gene trees and species trees using reconciled trees, which can show the history of the genes by comparing the species tree with the gene tree using the parsimony principle. The resulting reconciled tree is a map of a gene tree and a given species tree, with any incongruence between the two trees being explained by predicted gene gains and losses [44]. The evolutionary changes in the numbers of the T2R gene in tetrapod animals were estimated using the method of Niimura et al [23] and the program was kindly provided by them. The predicted number of T2R genes in the most recent common ancestor would be minimal, but this method can also provide a good estimate of the evolutionary dynamic changes in T2R genes (see above).

## Abbreviations

T2R: bitter taste receptor; GPCR: G protein-coupled receptors; OR: olfactory receptor; VR: vomeronasal pheromone receptor; MYA: million years ago.

## Authors' contributions

DD and SZ conceived this study. DD performed the work and the statistical analyses. All authors discussed the results. DD, GJ and SZ wrote the manuscript, and all authors commented on it and approved the final version of the manuscript.

## Supplementary Material

Additional file 1 **Supplementary tables and data.** The data provided is the DNA sequences of all T2R genes in 16 vertebrates. Click here for file

Additional file 2 **Supplementary Figure 1.** Evolutionary changes in the number of T2R genes in mammals. The phylogenetic tree contains the Euungulata (Perissodactyla + Cetartiodactyla) clade based on the report by Waddell et al The divergence times were based on those reported by Waddell et al. and Murphy et al. Click here for file

Additional file 3 **Supplementary Figure 2.** Evolutionary changes in the number of T2R genes in teleost fishes, the frog, lizard and chicken. The phylogenetic tree was obtained from Benton, and the divergence times were based on those reported by Hedges et al., Janke et al. and Aeschlimann et al Separate analyses were performed for teleost fishes and tetrapods. Click here for file
