# Supplementary material for: Dynamic evolution of bitter taste receptor genes in vertebrates
Source: BMC Evol Biol. 2009 Jan 15;9:12. doi: 10.1186/1471-2148-9-12 (PMC2646699; doi:10.1186/1471-2148-9-12)
Supplement: Additional file 1 — Supplementary tables and data. The data provided is the DNA sequences of all T2R genes in 16 vertebrates. [file 1471-2148-9-12-S1.doc]

**Supplementary Table**

**Supplementary Table 1** The size of previously reported T2R gene repertoires.

|  | Conte et al. (2002) | Conte et al. (2003) | Shi et al. (2003) | Lagerström et al. (2006) | Shi et al. (2006) | Go et al. (2006) | Gloriam et al. (2007) | Fredriksson et al. (2005) |
| --- | --- | --- | --- | --- | --- | --- | --- | --- |
| Human | 34 | — | 33 | — | 36 | 36 | 35 | 38 |
| Mouse | — | 40 | 36 | — | 41 | 41 | 40 | 3 |
| Rat | — | — | — | — | 42 | — | 41 | — |
| Dog | — | — | — | — | 21 | 19 | — | — |
| Cow | — | — | — | — | 34 | 29 | — | — |
| Opossum | — | — | — | — | 34 | 34 | — | — |
| Chicken | — | — | — | 3 | 3 | 3 | — | — |
| Frog | — | — | — | — | 64 | 54 | — | — |
| Fugu | — | — | — | — | 4 | 3 | — | — |
| Puffer fish | — | — | — | — | 6 | — | — | — |
| Zebrafish | — | — | — | — | 4 | 4 | — | — |

Note: “—“ represents not available.

**Supplementary Dataset**

**Supplementary Dataset 1** DNA sequences of 307 T2R genes. s“Intact”, “Pseudo” and “Partial” indicate an intact gene, a pseudogene and a partial gene, respectively.

>Human_T2R40--Intact

ATGGCAACGGTGAACACAGATGCCACAGATAAAGACATATCCAAGTTCAAGGTCACCTTCACTTTGGTGGTCTCCGGAATAGAGTGCATCACTGGCATCCTTGGGAGTGGCTTCATCACGGCCATCTATGGGGCTGAGTGGGCCAGGGGCAAAACACTCCCCACTGGTGACCGCATTATGTTGATGCTGAGCTTTTCCAGGCTCTTGCTACAGATTTGGATGATGCTGGAGAACATTTTCAGTCTGCTATTCCGAATTGTTTATAACCAAAACTCAGTGTATATCCTCTTCAAAGTCATCACTGTCTTTCTGAACCATTCCAATCTCTGGTTTGCTGCCTGGCTCAAAGTCTTCTATTGTCTTAGAATTGCAAACTTCAATCATCCTTTGTTCTTCCTGATGAAGAGGAAAATCATAGTGCTGATGCCTTGGCTTCTCAGGCTGTCAGTGTTGGTTTCCTTAAGCTTCAGCTTTCCTCTCTCGAGAGATGTCTTCAATGTGTATGTGAATAGCTCCATTCCTATCCCCTCCTCCAACTCCACGGAGAAGAAGTACTTCTCTGAGACCAATATGGTCAACCTGGTATTTTTCTATAACATGGGGATCTTCGTTCCTCTGATCATGTTCATCCTGGCAGCCACCCTGCTGATCCTCTCTCTCAAGAGACACACCCTACACATGGGAAGCAATGCCACAGGGTCCAGGGACCCCAGCATGAAGGCTCACATAGGGGCCATCAAAGCCACCAGCTACTTTCTCATCCTCTACATTTTCAATGCAATTGCTCTATTTCTTTCCACGTCCAACATCTTTGACACTTACAGTTCCTGGAATATTTTGTGCAAGATCATCATGGCTGCCTACCCTGCCGGCCACTCAGTACAACTGATCTTGGGCAACCCTGGGCTGAGAAGAGCCTGGAAGCGGTTTCAGCACCAAGTTCCTCTTTACCTAAAAGGGCAGACTCTGTGA

>Human_T2R47--Intact

ATGATAACTTTTCTGCCCATCATTTTTTCCATTCTAATAGTGGTTATATTTGTTATTGGAAATTTTGCTAATGGCTTCATAGCATTGGTAAATTCCATTGAGTGGGTCAAGAGACAAAAGATCTCCTTTGTTGACCAAATTCTCACTGCTCTGGCGGTCTCCAGAGTTGGTTTGCTCTGGGTGTTATTACTACATTGGTATGCAACTCAGTTGAATCCAGCTTTTTATAGTGTAGAAGTAAGAATTACTGCTTATAATGTCTGGGCAGTAACCAACCATTTCAGCAGCTGGCTTGCTACTAGCCTCAGCATGTTTTATTTGCTCAGGATTGCCAATTTCTCCAACCTTATTTTTCTTCGCATAAAGAGGAGAGTTAAGAGTGTTGTTCTGGTGATACTGTTGGGGCCTTTGCTATTTTTGGTTTGTCATCTTTTTGTGATAAACATGGATGAGACTGTATGGACAAAAGAATATGAAGGAAACGTGACTTGGAAGATCAAATTGAGGAGTGCAATGTACCATTCAAATATGACTCTAACCATGCTAGCAAACTTTGTACCCCTCACTCTGACCCTGATATCTTTTCTGCTGTTAATCTGTTCTCTGTGTAAACATCTCAAGAAGATGCAGCTCCATGGCAAAGGATCTCAAGATCCCAGCACCAAGGTCCACATAAAAGCTTTGCAAACTGTGACCTCCTTTCTTCTGTTATGTGCCATTTACTTTCTGTCCATGATCATATCAGTTTGTAATTTTGGGAGGCTGGAAAAGCAACCTGTCTTCATGTTCTGCCAAGCTATTATATTCAGCTATCCTTCAACCCACCCATTCATCCTGATTTTGGGAAACAAGAAGCTAAAGCAGATTTTTCTTTCAGTTTTGCGGCATGTGAGGTACTGGGTGAAAGACAGAAGCCTTCGTCTCCATAGATTCACAAGAGGGGCATTGTGTGTCTTCTAG

>Human_T2R50--Intact

ATGATAACTTTTCTATACATTTTTTTTTCAATTCTAATAATGGTTTTATTTGTTCTCGGAAACTTTGCCAATGGCTTCATAGCACTGGTAAATTTCATTGACTGGGTGAAGAGAAAAAAGATCTCCTCAGCTGACCAAATTCTCACTGCTCTGGCGGTCTCCAGAATTGGTTTGCTCTGGGCATTATTATTAAATTGGTATTTAACTGTGTTGAATCCAGCTTTTTATAGTGTAGAATTAAGAATTACTTCTTATAATGCCTGGGTTGTAACCAACCATTTCAGCATGTGGCTTGCTGCTAACCTCAGCATATTTTATTTGCTCAAGATTGCCAATTTCTCCAACCTTCTTTTTCTTCATTTAAAGAGGAGAGTTAGGAGTGTCATTCTGGTGATACTGTTGGGGACTTTGATATTTTTGGTTTGTCATCTTCTTGTGGCAAACATGGATGAGAGTATGTGGGCAGAAGAATATGAAGGAAACATGACTGGGAAGATGAAATTGAGGAATACAGTACATCTTTCATATTTGACTGTAACTACCCTATGGAGCTTCATACCCTTTACTCTGTCCCTGATATCTTTTCTGATGCTAATCTGTTCTCTGTGTAAACATCTCAAGAAGATGCAGCTCCATGGAGAAGGATCGCAAGATCTCAGCACCAAGGTCCACATAAAAGCTTTGCAAACTCTGATCTCCTTCCTCTTGTTATGTGCCATTTTCTTTCTATTCCTAATCGTTTCGGTTTGGAGTCCTAGGAGGCTGCGGAATGACCCGGTTGTCATGGTTAGCAAGGCTGTTGGAAACATATATCTTGCATTCGACTCATTCATCCTAATTTGGAGAACCAAGAAGCTAAAACACACCTTTCTTTTGATTTTGTGTCAGATTAGGTGCTGA

>Human_T2R42--Intact

ATGGCCACCGAATTGGACAAAATCTTTCTGATTCTGGCAATAGCAGAATTCATCATCAGCATGCTGGGGAATGTGTTCATTGGACTGGTAAACTGCTCTGAAGGGATCAAGAACCAAAAGGTCTTCTCAGCTGACTTCATCCTCACCTGCTTGGCTATCTCCACAATTGGACAACTGTTGGTGATACTGTTTGATTCATTTCTAGTGGGACTTGCTTCACATTTATATACCACATATAGACTAGGAAAAACTGTTATTATGCTTTGGCACATGACTAATCACTTGACAACCTGGCTTGCCACCTGCCTAAGCATTTTCTATTTCTTTAAGATAGCCCACTTCCCCCACTCCCTTTTCCTCTGGCTGAGGTGGAGGATGAACGGAATGATTGTTATGCTTCTTATATTGTCTTTGTTCTTACTGATTTTTGACAGTTTAGTGCTAGAAATATTTATTGATATCTCACTCAATATAATAGATAAAAGTAATCTGACTTTATATTTAGATGAAAGTAAAACTCTCTATGATAAACTCTCTATTTTAAAAACTCTTCTCAGCTTAACCAGTTTTATCCCCTTTTCTCTGTTCCTGACCTCCTTGCTTTTTTTATTTCTGTCCTTGGTGAGACATACTAGAAATTTGAAGCTCAGTTCCTTGGGCTCTAGAGACTCCAGCACAGAGGCCCATAGGAGGGCCATGAAAATGGTGATGTCTTTCCTTTTCCTCTTCATAGTTCATTTTTTTTCCTTACAAGTGGCCAATGGGATATTTTTTATGTTGTGGAACAACAAGTACATAAAGTTTGTCATGTTAGCCTTAAATGCCTTTCCCTCGTGCCACTCATTTATTCTCATTCTGGGAAACAGCAAGCTGCGACAGACAGCTGTGAGGCTACTGTGGCATCTTAGGAACTATACAAAAACACCAAATGCTTTACCTTTGTAG

>Human_T2R46--Intact

ATGATAACTTTTCTGCCCATCATTTTTTCCATTCTAATAGTGGTTACATTTGTGATTGGAAATTTTGCTAATGGCTTCATAGCATTGGTAAATTCCATTGAGTGGTTCAAGAGACAAAAGATCTCTTTTGCTGACCAAATTCTCACTGCTCTGGCAGTCTCCAGAGTTGGTTTACTCTGGGTATTAGTATTAAATTGGTATGCAACTGAGTTGAATCCAGCTTTTAACAGTATAGAAGTAAGAATTACTGCTTACAATGTCTGGGCAGTAATCAACCATTTCAGCAACTGGCTTGCTACTAGCCTCAGCATATTTTATTTGCTCAAGATTGCCAATTTCTCCAACCTTATTTTTCTTCACTTAAAGAGGAGAGTTAAGAGTGTTGTTCTGGTGATACTATTGGGGCCTTTGCTATTTTTGGTTTGTCATCTTTTTGTGATAAACATGAATCAGATTATATGGACAAAAGAATATGAAGGAAACATGACTTGGAAGATCAAACTGAGGAGTGCAATGTACCTTTCAAATACAACGGTAACCATCCTAGCAAACTTAGTTCCCTTCACTCTGACCCTGATATCTTTTCTGCTGTTAATCTGTTCTCTGTGTAAACATCTCAAAAAGATGCAGCTCCATGGCAAAGGATCTCAAGATCCCAGCATGAAGGTCCACATAAAAGCTTTGCAAACTGTGACCTCCTTCCTCTTGTTATGTGCCATTTACTTTCTGTCCATAATCATGTCAGTTTGGAGTTTTGAGAGTCTGGAAAACAAACCTGTCTTCATGTTCTGCGAAGCTATTGCATTCAGCTATCCTTCAACCCACCCATTCATCCTGATTTGGGGAAACAAGAAGCTAAAGCAGACTTTTCTTTCAGTTTTGTGGCATGTGAGGTACTGGGTGAAAGGAGAGAAGCCTTCATCTTCATAG

>Human_T2R43--Intact

ATGATAACTTTTCTACCCATCATTTTTTCCAGTCTGGTAGTGGTTACATTTGTTATTGGAAATTTTGCTAATGGCTTCATAGCACTGGTAAATTCCATTGAGTGGTTCAAGAGACAAAAGATCTCCTTTGCTGACCAAATTCTCACTGCTCTGGCGGTCTCCAGAGTTGGTTTGCTCTGGGTATTATTATTAAACTGGTATTCAACTGTGTTGAATCCAGCTTTTAATAGTGTAGAAGTAAGAACTACTGCTTATAATATCTGGGCAGTGATCAACCATTTCAGCAACTGGCTTGCTACTACCCTCAGCATATTTTATTTGCTCAAGATTGCCAATTTCTCCAACTTTATTTTTCTTCACTTAAAGAGGAGAGTTAAGAGTGTCATTCTGGTGATGTTGTTGGGGCCTTTGCTATTTTTGGCTTGTCATCTTTTTGTGATAAACATGAATGAGATTGTGCGGACAAAAGAATTTGAAGGAAACATGACTTGGAAGATCAAATTGAAGAGTGCAATGTACTTTTCAAATATGACTGTAACCATGGTAGCAAACTTAGTACCCTTCACTCTGACCCTACTATCTTTTATGCTGTTAATCTGTTCTTTGTGTAAACATCTCAAGAAGATGCAGCTCCATGGTAAAGGATCTCAAGATCCCAGCACCAAGGTCCACATAAAAGCTTTGCAAACTGTGATCTCCTTCCTCTTGTTATGTGCCATTTACTTTCTGTCCATAATGATATCAGTTTGGAGTTTTGGAAGTCTGGAAAACAAACCTGTCTTCATGTTCTGCAAAGCTATTAGATTCAGCTATCCTTCAATCCACCCATTCATCCTGATTTGGGGAAACAAGAAGCTAAAGCAGACTTTTCTTTCAGTTTTTTGGCAAATGAGGTACTGGGTGAAAGGAGAGAAGACTTCATCTCCATAG

>Human_T2R9--Intact

ATGCCAAGTGCAATAGAGGCAATATATATTATTTTAATTGCTGGTGAATTGACCATAGGGATTTGGGGAAATGGATTCATTGTACTAGTTAACTGCATTGACTGGCTCAAAAGAAGAGATATTTCCTTGATTGACATCATCCTGATCAGCTTGGCCATCTCCAGAATCTGTCTGCTGTGTGTAATATCATTAGATGGCTTCTTTATGCTGCTCTTTCCAGGTACATATGGCAATAGCGTGCTAGTAAGCATTGTGAATGTTGTCTGGACATTTGCCAATAATTCAAGTCTCTGGTTTACTTCTTGCCTCAGTATCTTCTATTTACTCAAGATAGCCAATATATCGCACCCATTTTTCTTCTGGCTGAAGCTAAAGATCAACAAGGTCATGCTTGCGATTCTTCTGGGGTCCTTTCTTATCTCTTTAATTATTAGTGTTCCAAAGAATGATGATATGTGGTATCACCTTTTCAAAGTCAGTCATGAAGAAAACATTACTTGGAAATTCAAAGTGAGTAAAATTCCAGGTACTTTCAAACAGTTAACCCTGAACCTGGGGGTGATGGTTCCCTTTATCCTTTGCCTGATCTCATTTTTCTTGTTACTTTTCTCCCTAGTTAGACACACCAAGCAGATTCGACTGCATGCTACAGGGTTCAGAGACCCCAGTACAGAGGCCCACATGAGGGCCATAAAGGCAGTGATCATCTTTCTGCTCCTCCTCATCGTGTACTACCCAGTCTTTCTTGTTATGACCTCTAGCGCTCTGATTCCTCAGGGAAAATTAGTGTTGATGATTGGTGACATAGTAACTGTCATTTTCCCATCAAGCCATTCATTCATTCTAATTATGGGAAATAGCAAGTTGAGGGAAGCTTTTCTGAAGATGTTAAGATTTGTGAAGTGTTTCCTTAGAAGAAGAAAGCCTTTTGTTCCATAG

>Human_T2R16--Intact

ATGTTCAGTCCTGCAGATAACATCTTTATAATCCTAATAACTGGAGAATTCATACTAGGAATATTGGGGAATGGATACATTGCACTAGTCAACTGGATTGACTGGATTAAGAAGAAAAAGATTTCCACAGTTGACTACATCCTTACCAATTTAGTTATCGCCAGAATTTGTTTGATCAGTGTAATGGTTGTAAATGGCATTGTAATAGTACTGAACCCAGATGTTTATACAAAAAATAAACAACAGATAGTCATTTTTACCTTCTGGACATTTGCCAACTACTTAAATATGTGGATTACCACCTGCCTTAATGTCTTCTATTTTCTGAAGATAGCCAGTTCCTCTCATCCACTTTTTCTCTGGCTGAAGTGGAAAATTGATATGGTGGTGCACTGGATCCTGCTGGGATGCTTTGCCATTTCCTTGTTGGTCAGCCTTATAGCAGCAATAGTACTGAGTTGTGATTATAGGTTTCATGCAATTGCCAAACATAAAAGAAACATTACTGAAATGTTCCATGTGAGTAAAATACCATACTTTGAACCCTTGACTCTCTTTAACCTGTTTGCAATTGTCCCATTTATTGTGTCACTGATATCATTTTTCCTTTTAGTAAGATCTTTATGGAGACATACCAAGCAAATAAAACTCTATGCTACCGGCAGTAGAGACCCCAGCACAGAAGTTCATGTGAGAGCCATTAAAACTATGACTTCATTTATCTTCTTTTTTTTCCTATACTATATTTCTTCTATTTTGATGACCTTTAGCTATCTTATGACAAAATACAAGTTAGCTGTGGAGTTTGGAGAGATTGCAGCAATTCTCTACCCCTTGGGTCACTCACTTATTTTAATTGTTTTAAATAATAAACTGAGGCAGACATTTGTCAGAATGCTGACATGTAGAAAAATTGCCTGCATGATATGA

>Human_T2R48--Intact

ATGATGTGTTTTCTGCTCATCATTTCATCAATTCTGGTAGTGTTTGCATTTGTTCTTGGAAATGTTGCCAATGGCTTCATAGCCCTAGTAAATGTCATTGACTGGGTTAACACACGAAAGATCTCCTCAGCTGAGCAAATTCTCACTGCTCTGGTGGTCTCCAGAATTGGTTTACTCTGGGTCATGTTATTCCTTTGGTATGCAACTGTGTTTAATTCTGCTTTATATGGTTTAGAAGTAAGAATTGTTGCTTCTAATGCCTGGGCTGTAACGAACCATTTCAGCATGTGGCTTGCTGCTAGCCTCAGCATATTTTGTTTGCTCAAGATTGCCAATTTCTCCAACCTTATTTCTCTCCACCTAAAGAAGAGAATTAAGAGTGTTGTTCTGGTGATACTGTTGGGGCCCTTGGTATTTCTGATTTGTAATCTTGCTGTGATAACCATGGATGAGAGAGTGTGGACAAAAGAATATGAAGGAAATGTGACTTGGAAGATCAAATTGAGGAATGCAATACACCTTTCAAGCTTGACTGTAACTACTCTAGCAAACCTCATACCCTTTACTCTGAGCCTAATATGTTTTCTGCTGTTAATCTGTTCTCTTTGTAAACATCTCAAGAAGATGCGGCTCCATAGCAAAGGATCTCAAGATCCCAGCACCAAGGTCCATATAAAAGCTTTGCAAACTGTGACCTCCTTCCTCATGTTATTTGCCATTTACTTTCTGTGTATAATCACATCAACTTGGAATCTTAGGACACAGCAGAGCAAACTTGTACTCCTGCTTTGCCAAACTGTTGCAATCATGTATCCTTCATTCCACTCATTCATCCTGATTATGGGAAGTAGGAAGCTAAAACAGACCTTTCTTTCAGTTTTGTGGCAGATGACACGCTGA

>Human_T2R13--Intact

ATGGAAAGTGCCCTGCCGAGTATCTTCACTCTTGTAATAATTGCAGAATTCATAATTGGGAATTTGAGCAATGGATTTATAGTACTGATCAACTGCATTGACTGGGTCAGTAAAAGAGAGCTGTCCTCAGTCGATAAACTCCTCATTATCTTGGCAATCTCCAGAATTGGGCTGATCTGGGAAATATTAGTAAGTTGGTTTTTAGCTCTGCATTATCTAGCCATATTTGTGTCTGGAACAGGATTAAGAATTATGATTTTTAGCTGGATAGTTTCTAATCACTTCAATCTCTGGCTTGCTACAATCTTCAGCATCTTTTATTTGCTCAAAATAGCGAGTTTCTCTAGCCCTGCTTTTCTCTATTTGAAGTGGAGAGTAAACAAAGTGATTCTGATGATACTGCTAGGAACCTTGGTCTTCTTATTTTTAAATCTGATACAAATAAACATGCATATAAAAGACTGGCTGGACCGATATGAAAGAAACACAACTTGGAATTTCAGTATGAGTGACTTTGAAACATTTTCAGTGTCGGTCAAATTCACTATGACTATGTTCAGTCTAACACCATTTACTGTGGCCTTCATCTCTTTTCTCCTGTTAATTTTCTCCCTGCAGAAACATCTCCAGAAAATGCAACTCAATTACAAAGGACACAGAGACCCCAGGACCAAGGTCCATACAAATGCCTTGAAAATTGTGATCTCATTCCTTTTATTCTATGCTAGTTTCTTTCTATGTGTTCTCATATCATGGATTTCTGAGCTGTATCAGAACACAGTGATCTACATGCTTTGTGAGACGATTGGAGTCTTCTCTCCTTCAAGCCACTCCTTTCTTCTGATTCTAGGAAACGCTAAGTTAAGACAGGCCTTTCTTTTGGTGGCAGCTAAGGTATGGGCTAAACGATGA

>Human_T2R49--Intact

ATGATGAGTTTTCTACACATTGTTTTTTCCATTCTAGTAGTGGTTGCATTTATTCTTGGAAATTTTGCCAATGGCTTTATAGCACTGATAAATTTCATTGCCTGGGTCAAGAGACAAAAGATCTCCTCAGCTGATCAAATTATTGCTGCTCTGGCAGTCTCCAGAGTTGGTTTGCTCTGGGTAATATTATTACATTGGTATTCAACTGTGTTGAATCCAACTTCATCTAATTTAAAAGTAATAATTTTTATTTCTAATGCCTGGGCAGTAACCAATCATTTCAGCATCTGGCTTGCTACTAGCCTCAGCATATTTTATTTGCTCAAGATCGTCAATTTCTCCAGACTTATTTTTCATCACTTAAAAAGGAAGGCTAAGAGTGTAGTTCTGGTGATAGTGTTGGGGTCTTTGTTCTTTTTGGTTTGTCACCTTGTGATGAAACACACGTATATAAATGTGTGGACAGAAGAATGTGAAGGAAACGTAACTTGGAAGATCAAACTGAGGAATGCAATGCACCTTTCCAACTTGACTGTAGCCATGCTAGCAAACTTGATACCATTCACTCTGACCCTGATATCTTTTCTGCTGTTAATCTACTCTCTGTGTAAACATCTGAAGAAGATGCAGCTCCATGGCAAAGGATCTCAAGATCCCAGCACCAAGATCCACATAAAAGCTCTGCAAACTGTGACCTCCTTCCTCATATTACTTGCCATTTACTTTCTGTGTCTAATCATATCGTTTTGGAATTTTAAGATGCGACCAAAAGAAATTGTCTTAATGCTTTGCCAAGCTTTTGGAATCATATATCCATCATTCCACTCATTCATTCTGATTTGGGGGAACAAGACGCTAAAGCAGACCTTTCTTTCAGTTTTGTGGCAGGTGACTTGCTGGGCAAAAGGACAGAACCAGTCAACTCCATAG

>Human_T2R5--Intact

ATGCTGAGCGCTGGCCTAGGACTGCTGATGCTGGTGGCAGTGGTTGAATTTCTCATCGGTTTAATTGGAAATGGAAGCCTGGTGGTCTGGAGTTTTAGAGAATGGATCAGAAAATTCAACTGGTCCTCATATAACCTCATTATCCTGGGCCTGGCTGGCTGCCGATTTCTCCTGCAGTGGCTGATCATTTTGGACTTAAGCTTGTTTCCACTTTTCCAGAGCAGCCGTTGGCTTCGCTATCTTAGTATCTTCTGGGTCCTGGTAAGCCAGGCCAGCTTATGGTTTGCCACCTTCCTCAGTGTCTTCTATTGCAAGAAGATCACGACCTTCGATCGCCCGGCCTACTTGTGGCTGAAGCAGAGGGCCTATAACCTGAGTCTCTGGTGCCTTCTGGGCTACTTTATAATCAATTTGTTACTTACAGTCCAAATTGGCTTAACATTCTATCATCCTCCCCAAGGAAACAGCAGCATTCGGTATCCCTTTGAAAGCTGGCAGTACCTGTATGCATTTCAGCTCAATTCAGGAAGTTATTTGCCTTTAGTGGTGTTTCTTGTTTCCTCTGGGATGCTGATTGTCTCTTTGTATACACACCACAAGAAGATGAAGGTCCATTCAGCTGGTAGGAGGGATGTCCGGGCCAAGGCTCACATCACTGCGCTGAAGTCCTTGGGCTGCTTCCTCTTACTTCACCTGGTTTATATCATGGCCAGCCCCTTCTCCATCACCTCCAAGACTTATCCTCCTGATCTCACCAGTGTCTTCATCTGGGAGACACTCATGGCAGCCTATCCTTCTCTTCATTCTCTCATATTGATCATGGGGATTCCTAGGGTGAAGCAGACTTGTCAGAAGATCCTGTGGAAGACAGTGTGTGCTCGGAGATGCTGGGGCCCATGA

>Human_T2R10--Intact

ATGCTACGTGTAGTGGAAGGCATCTTCATTTTTGTTGTAGTTAGTGAGTCAGTGTTTGGGGTTTTGGGGAATGGATTTATTGGACTTGTAAACTGCATTGACTGTGCCAAGAATAAGTTATCTACGATTGGCTTTATTCTCACCGGCTTAGCTATTTCAAGAATTTTTCTGATATGGATAATAATTACAGATGGATTTATACAGATATTCTCTCCAAATATATATGCCTCCGGTAACCTAATTGAATATATTAGTTACTTTTGGGTAATTGGTAATCAATCAAGTATGTGGTTTGCCACCAGCCTCAGCATCTTCTATTTCCTGAAGATAGCAAATTTTTCCAACTACATATTTCTCTGGTTGAAGAGCAGAACAAATATGGTTCTTCCCTTCATGATAGTATTCTTACTTATTTCATCGTTACTTAATTTTGCATACATTGCGAAGATTCTTAATGATTATAAAACGAAGAATGACACAGTCTGGGATCTCAACATGTATAAAAGTGAATACTTTATTAAACAGATTTTGCTAAATCTGGGAGTCATTTTCTTCTTTACACTATCCCTAATTACATGTATTTTTTTAATCATTTCCCTTTGGAGACACAACAGGCAGATGCAATCGAATGTGACAGGATTGAGAGACTCCAACACAGAAGCTCATGTGAAGGCAATGAAAGTTTTGATATCTTTCATCATCCTCTTTATCTTGTATTTTATAGGCATGGCCATAGAAATATCATGTTTTACTGTGCGAGAAAACAAACTGCTGCTTATGTTTGGAATGACAACCACAGCCATCTATCCCTGGGGTCACTCATTTATCTTAATTCTAGGAAACAGCAAGCTAAAGCAAGCCTCTTTGAGGGTACTGCAGCAATTGAAGTGCTGTGAGAAAAGGAAAAATCTCAGAGTCACATAG

>Human_T2R44--Intact

ATGACAACTTTTATACCCATCATTTTTTCCAGTGTGGTAGTGGTTCTATTTGTTATTGGAAATTTTGCTAATGGCTTCATAGCATTGGTAAATTCCATTGAGCGGGTCAAGAGACAAAAGATCTCTTTTGCTGACCAGATTCTCACTGCTCTGGCGGTCTCCAGAGTTGGTTTGCTCTGGGTATTATTATTAAATTGGTATTCAACTGTGTTTAATCCAGCTTTTTATAGTGTAGAAGTAAGAACTACTGCTTATAATGTCTGGGCAGTAACCGGCCATTTCAGCAACTGGCTTGCTACTAGCCTCAGCATATTTTATTTGCTCAAGATTGCCAATTTCTCCAACCTTATTTTTCTTCACTTAAAGAGGAGAGTTAAGAGTGTCATTCTGGTGATGCTGTTGGGGCCTTTACTATTTTTGGCTTGTCAACTTTTTGTGATAAACATGAAAGAGATTGTACGGACAAAAGAATATGAAGGAAACTTGACTTGGAAGATCAAATTGAGGAGTGCAGTGTACCTTTCAGATGCGACTGTAACCACGCTAGGAAACTTAGTGCCCTTCACTCTGACCCTGCTATGTTTTTTGCTGTTAATCTGTTCTCTGTGTAAACATCTCAAGAAGATGCAGCTCCATGGTAAAGGATCTCAAGATCCCAGCACCAAGGTCCACATAAAAGCTTTGCAAACTGTGATCTTTTTCCTCTTGTTATGTGCCGTTTACTTTCTGTCCATAATGATATCAGTTTGGAGTTTTGGGAGTCTGGAAAACAAACCTGTCTTCATGTTCTGCAAAGCTATTAGATTCAGCTATCCTTCAATCCACCCATTCATCCTGATTTGGGGAAACAAGAAGCTAAAGCAGACTTTTCTTTCAGTTTTGCGGCAAGTGAGGTACTGGGTGAAAGGAGAGAAGCCTTCATCTCCATAG

>Human_T2R38--Intact

ATGTTGACTCTAACTCGCATCCGCACTGTGTCCTATGAAGTCAGGAGTACATTTCTGTTCATTTCAGTCCTGGAGTTTGCAGTGGGGTTTCTGACCAATGCCTTCGTTTTCTTGGTGAATTTTTGGGATGTAGTGAAGAGGCAGGCACTGAGCAACAGTGATTGTGTGCTGCTGTGTCTCAGCATCAGCCGGCTTTTCCTGCATGGACTGCTGTTCCTGAGTGCTATCCAGCTTACCCACTTCCAGAAGTTGAGTGAACCACTGAACCACAGCTACCAAGCCATCATCATGCTATGGATGATTGCAAACCAAGCCAACCTCTGGCTTGCTGCCTGCCTCAGCCTGCTTTACTGCTCCAAGCTCATCCGTTTCTCTCACACCTTCCTGATCTGCTTGGCAAGCTGGGTCTCCAGGAAGATCTCCCAGATGCTCCTGGGTATTATTCTTTGCTCCTGCATCTGCACTGTCCTCTGTGTTTGGTGCTTTTTTAGCAGACCTCACTTCACAGTCACAACTGTGCTATTCATGAATAACAATACAAGGCTCAACTGGCAGATTAAAGATCTCAATTTATTTTATTCCTTTCTCTTCTGCTATCTGTGGTCTGTGCCTCCTTTCCTATTGTTTCTGGTTTCTTCTGGGATGCTGACTGTCTCCCTGGGAAGGCACATGAGGACAATGAAGGTCTATACCAGAAACTCTCGTGACCCCAGCCTGGAGGCCCACATTAAAGCCCTCAAGTCTCTTGTCTCCTTTTTCTGCTTCTTTGTGATATCATCCTGTGCTGCCTTCATCTCTGTGCCCCTACTGATTCTGTGGCGCGACAAAATAGGGGTGATGGTTTGTGTTGGGATAATGGCAGCTTGTCCCTCTGGGCATGCAGCCATCCTGATCTCAGGCAATGCCAAGTTGAGGAGAGCTGTGATGACCATTCTGCTCTGGGCTCAGAGCAGCCTGAAGGTAAGAGCCGACCACAAGGCAGATTCCCGGACACTGTGCTGA

>Human_T2R16--Intact

ATGATACCCATCCAACTCACTGTCTTCTTCATGATCATCTATGTGCTTGAGTCCTTGACAATTATTGTGCAGAGCAGCCTAATTGTTGCAGTGCTGGGCAGAGAATGGCTGCAAGTCAGAAGGCTGATGCCTGTGGACATGATTCTCATCAGCCTGGGCATCTCTCGCTTCTGTCTACAGTGGGCATCAATGCTGAACAATTTTTGCTCCTATTTTAATTTGAATTATGTACTTTGCAACTTAACAATCACCTGGGAATTTTTTAATATCCTTACATTCTGGTTAAACAGCTTGCTTACCGTGTTCTACTGCATCAAGGTCTCTTCTTTCACCCATCACATCTTTCTCTGGCTGAGGTGGAGAATTTTGAGGTTGTTTCCCTGGATATTACTGGGTTCTCTGATGATTACTTGTGTAACAATCATCCCTTCAGCTATTGGGAATTACATTCAAATTCAGTTACTCACCATGGAGCATCTACCAAGAAACAGCACTGTAACTGACAAACTTGAAAATTTTCATCAGTATCAGTTCCAGGCTCATACAGTTGCATTGGTTATTCCTTTCATCCTGTTCCTGGCCTCCACCATCTTTCTCATGGCATCACTGACCAAGCAGATACAACATCATAGCACTGGTCACTGCAATCCAAGCATGAAAGCGCGCTTCACTGCCCTGAGGTCCCTTGCCGTCTTATTTATTGTGTTTACCTCTTACTTTCTAACCATACTCATCACCATTATAGGTACTCTATTTGATAAGAGATGTTGGTTATGGGTCTGGGAAGCTTTTGTCTATGCTTTCATCTTAATGCATTCCACTTCACTGATGCTGAGCAGCCCTACGTTGAAAAGGATTCTAAAGGGAAAGTGCTAG

>Human_T2R1--Intact

ATGCTAGAGTCTCACCTCATTATCTATTTTCTTCTTGCAGTGATACAATTTCTTCTTGGGATTTTCACAAATGGCATCATTGTGGTGGTGAATGGCATTGACTTGATCAAGCACAGAAAAATGGCTCCGCTGGATCTCCTTCTTTCTTGTCTGGCAGTTTCTAGAATTTTTCTGCAGTTGTTCATCTTCTACGTTAATGTGATTGTTATCTTCTTCATAGAATTCATCATGTGTTCTGCGAATTGTGCAATTCTCTTATTTATAAATGAATTGGAACTTTGGCTTGCCACATGGCTCGGCGTTTTCTATTGTGCCAAGGTTGCCAGCGTCCGTCACCCACTCTTCATCTGGTTGAAGATGAGGATATCCAAGCTGGTCCCATGGATGATCCTGGGGTCTCTGCTATATGTATCTATGATTTGTGTTTTCCATAGCAAATATGCAGGGTTTATGGTCCCATACTTCCTAAGGAAATTTTTCTCCCAAAATGCCACAATTCAAAAAGAAGATACACTGGCTATACAGATTTTCTCTTTTGTTGCTGAGTTCTCAGTGCCATTGCTTATCTTCCTTTTTGCTGTTTTGCTCTTGATTTTCTCTCTGGGGAGGCACACCCGGCAAATGAGAAACACAGTGGCCGGCAGCAGGGTTCCTGGCAGGGGTGCACCCATCAGCGCGTTGCTGTCTATCCTGTCCTTCCTGATCCTCTACTTCTCCCACTGCATGATAAAAGTTTTTCTCTCTTCTCTAAAGTTTCACATCAGAAGGTTCATCTTTCTGTTCTTCATCCTTGTGATTGGTATATACCCTTCTGGACACTCTCTCATCTTAATTTTAGGAAATCCTAAATTGAAACAAAATGCAAAAAAGTTCCTCCTCCACAGTAAGTGCTGTCAGTGA

>Human_T2R4--Intact

ATGCTTCGGTTATTCTATTTCTCTGCTATTATTGCCTCAGTTATTTTAAATTTTGTAGGAATCATTATGAATCTGTTTATTACAGTGGTCAATTGCAAAACTTGGGTCAAAAGCCATAGAATCTCCTCTTCTGATAGGATTCTGTTCAGCCTGGGCATCACCAGGTTTCTTATGCTGGGACTATTTCTGGTGAACACCATCTACTTCGTCTCTTCAAATACGGAAAGGTCAGTCTACCTGTCTGCTTTTTTTGTGTTGTGTTTCATGTTTTTGGACTCGAGCAGTGTCTGGTTTGTGACCTTGCTCAATATCTTGTACTGTGTGAAGATTACTAACTTCCAACACTCAGTGTTTCTCCTGCTGAAGCGGAATATCTCCCCAAAGATCCCCAGGCTGCTGCTGGCCTGTGTGCTGATTTCTGCTTTCACCACTTGCCTGTACATCACGCTTAGCCAGGCATCACCTTTTCCTGAACTTGTGACTACGAGAAATAACACATCATTTAATATCAGTGAGGGCATCTTGTCTTTAGTGGTTTCTTTGGTCTTGAGCTCATCTCTCCAGTTCATCATTAATGTGACTTCTGCTTCCTTGCTAATACACTCCTTGAGGAGACATATACAGAAGATGCAGAAAAATGCCACTGGTTTCTGGAATCCCCAGACGGAAGCTCATGTAGGTGCTATGAAGCTGATGGTCTATTTCCTCATCCTCTACATTCCATATTCAGTTGCTACCCTGGTCCAGTATCTCCCCTTTTATGCAGGGATGGATATGGGGACCAAATCCATTTGTCTGATTTTTGCCACCCTTTACTCTCCAGGACATTCTGTTCTCATTATTATCACACATCCTAAACTGAAAACAACAGCAAAGAAGATTCTTTGTTTCAAAAAATAG

>Human_T2R39--Intact

ATGACTAAACTCTGCGATCCTGCAGAAAGTGAATTGTCGCCATTTCTCATCACCTTAATTTTAGCAGTTTTACTTGCTGAATACCTCATTGGTATCATTGCAAATGGTTTCATCATGGCTATACATGCAGCTGAATGGGTTCAAAATAAGGCAGTTTCCACAAGTGGCAGGATCCTGGTTTTCCTGAGTGTATCCAGAATAGCTCTCCAAAGCCTCATGATGTTAGAAATTACCATCAGCTCAACCTCCCTAAGTTTTTATTCTGAAGACGCTGTATATTATGCATTCAAAATAAGTTTTATATTCTTAAATTTTTGTAGCCTGTGGTTTGCTGCCTGGCTCAGTTTCTTCTACTTTGTGAAGATTGCCAATTTCTCCTACCCCCTTTTCCTCAAACTGAGGTGGAGAATTACTGGATTGATACCCTGGCTTCTGTGGCTGTCCGTGTTTATTTCCTTCAGTCACAGCATGTTCTGCATCAACATCTGCACTGTGTATTGTAACAATTCTTTCCCTATCCACTCCTCCAACTCCACTAAGAAAACATACTTGTCTGAGATCAATGTGGTCGGTCTGGCTTTTTTCTTTAACCTGGGGATTGTGACTCCTCTGATCATGTTCATCCTGACAGCCACCCTGCTGATCCTCTCTCTCAAGAGACACACCCTACACATGGGAAGCAATGCCACAGGGTCCAACGACCCCAGCATGGAGGCTCACATGGGGGCCATCAAAGCTATCAGCTACTTTCTCATTCTCTACATTTTCAATGCAGTTGCTCTGTTTATCTACCTGTCCAACATGTTTGACATCAACAGTCTGTGGAATAATTTGTGCCAGATCATCATGGCTGCCTACCCTGCCAGCCACTCAATTCTACTGATTCAAGATAACCCTGGGCTGAGAAGAGCCTGGAAGCGGCTTCAGCTTCGACTTCATCTTTACCCAAAAGAGTGGACTCTGTGA

>Human_T2R60--Intact

ATGAATGGAGACCACATGGTTCTAGGATCTTCGGTGACTGACAAGAAGGCCATCATCTTGGTTACCATTTTACTCCTTTTACGCCTGGTAGCAATAGCAGGCAATGGCTTCATCACTGCTGCTCTGGGCGTGGAGTGGGTGCTACGGAGAATGTTGTTGCCTTGTGATAAGTTATTGGTTAGCCTAGGGGCCTCTCGCTTCTGTCTGCAGTCAGTGGTAATGGGTAAGACCATTTATGTTTTCTTGCATCCGATGGCCTTCCCATACAACCCTGTACTGCAGTTTCTAGCTTTCCAGTGGGACTTCCTGAATGCTGCCACCTTATGGTCCTCTACCTGGCTCAGTGTCTTCTATTGTGTGAAAATTGCTACCTTCACCCACCCTGTCTTCTTCTGGCTAAAGCACAAGTTGTCTGGGTGGCTACCATGGATGCTCTTCAGCTCTGTAGGGCTCTCCAGCTTCACCACCATTCTATTTTTCATAGGCAACCACAGAATGTATCAGAACTATTTAAGGAACCATCTACAACCTTGGAATGTCACTGGCGATAGCATACGGAGCTACTGTGAGAAATTCTATCTCTTCCCTCTAAAAATGATTACTTGGACAATGCCCACTGCTGTCTTTTTCATTTGCATGATTTTGCTCATCACATCTCTGGGAAGACACAGGAAGAAGGCTCTCCTTACAACCTCAGGATTCCGAGAGCCCAGTGTGCAGGCACACATAAAGGCTCTGCTGGCTCTCCTCTCTTTTGCCATGCTCTTCATCTCATATTTCCTGTCACTGGTGTTCAGTGCTGCAGGTATTTTTCCACCTCTGGACTTTAAATTCTGGGTGTGGGAGTCAGTGATTTATCTGTGTGCAGCAGTTCACCCCATCATTCTGCTCTTCAGCAACTGCAGGCTGAGAGCTGTGCTGAAGAGTCGTCGTTCCTCAAGGTGTGGGACACCTTGA

>Human_T2R41--Intact

ATGCAAGCAGCACTGACGGCCTTCTTCGTGTTGCTCTTTAGCCTGCTGAGTCTTCTGGGGATTGCAGCGAATGGCTTCATTGTGCTGGTGCTGGGCAGGGAGTGGCTGCGATATGGCAGGTTGCTGCCCTTGGATATGATCCTCATTAGCTTGGGTGCCTCCCGCTTCTGCCTGCAGTTGGTTGGGACGGTGCACAACTTCTACTACTCTGCCCAGAAGGTCGAGTACTCTGGGGGTCTCGGCCGACAGTTCTTCCATCTACACTGGCACTTCCTGAACTCAGCCACCTTCTGGTTTTGCAGCTGGCTCAGTGTCCTGTTCTGTGTGAAGATTGCTAACATCACACACTCCACCTTCCTGTGGCTGAAGTGGAGGTTCCCAGGGTGGGTGCCCTGGCTCCTGTTGGGCTCTGTCCTGATCTCCTTCATCATAACCCTGCTGTTTTTTTGGGTGAACTACCCTGTATATCAAGAATTTTTAATTAGAAAATTTTCTGGGAACATGACCTACAAGTGGAATACAAGGATAGAAACATACTATTTCCCATCCCTGAAACTGGTCATCTGGTCAATTCCTTTTTCTGTTTTTCTGGTCTCAATTATGCTGTTAATTAATTCTCTGAGGAGGCATACTCAGAGAATGCAGCACAACGGGCACAGCCTGCAGGACCCCAGCACCCAGGCTCACACCAGAGCTCTGAAGTCCCTCATCTCCTTCCTCATTCTTTATGCTCTGTCCTTTCTGTCCCTGATCATTGATGCCGCAAAATTTATCTCCATGCAGAACGACTTTTACTGGCCATGGCAAATTGCAGTCTACCTGTGCATATCTGTCCATCCCTTCATCCTCATCTTCAGCAACCTCAAGCTTCGAAGCGTGTTCTCGCAGCTCCTGTTGTTGGCAAGGGGCTTCTGGGTGGCCTAG

>Human_T2R3--Intact

ATGATGGGACTCACCGAGGGGGTGTTCCTGATTCTGTCTGGCACTCAGTTCACACTGGGAATTCTGGTCAATTGTTTCATTGAGTTGGTCAATGGTAGCAGCTGGTTCAAGACCAAGAGAATGTCTTTGTCTGACTTCATCATCACCACCCTGGCACTCTTGAGGATCATTCTGCTGTGTATTATCTTGACTGATAGTTTTTTAATAGAATTCTCTCCCAACACACATGATTCAGGGATAATAATGCAAATTATTGATGTTTCCTGGACATTTACAAACCATCTGAGCATTTGGCTTGCCACCTGTCTTGGTGTCCTCTACTGCCTGAAAATCGCCAGTTTCTCTCACCCCACATTCCTCTGGCTCAAGTGGAGAGTTTCTAGGGTGATGGTATGGATGCTGTTGGGTGCACTGCTCTTATCCTGTGGTAGTACCGCATCTCTGATCAATGAGTTTAAGCTCTATTCTGTCTTTAGGGGAATTGAGGCCACCAGGAATGTGACTGAACACTTCAGAAAGAAGAGGAGTGAGTATTATCTGATCCATGTTCTTGGGACTCTGTGGTACCTGCCTCCCTTAATTGTGTCCCTGGCCTCCTACTCTTTGCTCATCTTCTCCCTGGGGAGGCACACACGGCAGATGCTGCAAAATGGGACAAGCTCCAGAGATCCAACCACTGAGGCCCACAAGAGGGCCATCAGAATCATCCTTTCCTTCTTCTTTCTCTTCTTACTTTACTTTCTTGCTTTCTTAATTGCATCATTTGGTAATTTCCTACCAAAAACCAAGATGGCTAAGATGATTGGCGAAGTAATGACAATGTTTTATCCTGCTGGCCACTCATTTATTCTCATTCTGGGGAACAGTAAGCTGAAGCAGACATTTGTAGTGATGCTCCGGTGTGAGTCTGGTCATCTGAAGCCTGGATCCAAGGGACCCATTTTCTCTTAG

>Human_T2R7--Intact

ATGGCAGATAAAGTGCAGACTACTTTATTGTTCTTAGCAGTTGGAGAGTTTTCAGTGGGGATCTTAGGGAATGCATTCATTGGATTGGTAAACTGCATGGACTGGGTCAAGAAGAGGAAAATTGCCTCCATTGATTTAATCCTCACAAGTCTGGCCATATCCAGAATTTGTCTATTGTGCGTAATACTATTAGATTGTTTTATATTGGTGCTATATCCAGATGTCTATGCCACTGGTAAAGAAATGAGAATCATTGACTTCTTCTGGACACTAACCAATCATTTAAGTATCTGGTTTGCAACCTGCCTCAGCATTTACTATTTCTTCAAGATAGGTAATTTCTTTCACCCACTTTTCCTCTGGATGAAGTGGAGAATTGACAGGGTGATTTCCTGGATTCTACTGGGGTGCGTGGTTCTCTCTGTGTTTATTAGCCTTCCAGCCACTGAGAATTTGAACGCTGATTTCAGGTTTTGTGTGAAGGCAAAGAGGAAAACAAACTTAACTTGGAGTTGCAGAGTAAATAAAACTCAACATGCTTCTACCAAGTTATTTCTCAACCTGGCAACGCTGCTCCCCTTTTGTGTGTGCCTAATGTCCTTTTTCCTCTTGATCCTCTCCCTGCGGAGACATATCAGGCGAATGCAGCTCAGTGCCACAGGGTGCAGAGACCCCAGCACAGAAGCCCATGTGAGAGCCCTGAAAGCTGTCATTTCCTTCCTTCTCCTCTTTATTGCCTACTATTTGTCCTTTCTCATTGCCACCTCCAGCTACTTTATGCCAGAGACGGAATTAGCTGTGATTTTTGGTGAGTCCATAGCTCTAATCTACCCCTCAAGTCATTCATTTATCCTAATACTGGGGAACAATAAATTAAGACATGCATCTCTAAAGGTGATTTGGAAAGTAATGTCTATTCTAAAAGGAAGAAAATTCCAACAACATAAACAAATCTGA

>Human_T2R14--Intact

ATGGGTGGTGTCATAAAGAGCATATTTACATTCGTTTTAATTGTGGAATTTATAATTGGAAATTTAGGAAATAGTTTCATAGCACTGGTGAACTGTATTGACTGGGTCAAGGGAAGAAAGATCTCTTCGGTTGATCGGATCCTCACTGCTTTGGCAATCTCTCGAATTAGCCTGGTTTGGTTAATATTCGGAAGCTGGTGTGTGTCTGTGTTTTTCCCAGCTTTATTTGCCACTGAAAAAATGTTCAGAATGCTTACTAATATCTGGACAGTGATCAATCATTTTAGTGTCTGGTTAGCTACAGGCCTCGGTACTTTTTATTTTCTCAAGATAGCCAATTTTTCTAACTCTATTTTTCTCTACCTAAAGTGGAGGGTTAAAAAGGTGGTTTTGGTGCTGCTTCTTGTGACTTCGGTCTTCTTGTTTTTAAATATTGCACTGATAAACATCCATATAAATGCCAGTATCAATGGATACAGAAGAAACAAGACTTGCAGTTCTGATTCAAGTAACTTTACACGATTTTCCAGTCTTATTGTATTAACCAGCACTGTGTTCATTTTCATACCCTTTACTTTGTCCCTGGCAATGTTTCTTCTCCTCATCTTCTCCATGTGGAAACATCGCAAGAAGATGCAGCACACTGTCAAAATATCCGGAGACGCCAGCACCAAAGCCCACAGAGGAGTTAAAAGTGTGATCACTTTCTTCCTACTCTATGCCATTTTCTCTCTGTCTTTTTTCATATCAGTTTGGACCTCTGAAAGGTTGGAGGAAAATCTAATTATTCTTTCCCAGGTGATGGGAATGGCTTATCCTTCATGTCACTCATGTGTTCTGATTCTTGGAAACAAGAAGCTGAGACAGGCCTCTCTGTCAGTGCTACTGTGGCTGAGGTACATGTTCAAAGATGGGGAGCCCTCAGGTCACAAAGAATTTAGAGAATCATCTTGA

>Mouse_T2R119--Intact

ATGGAAGGTCATATGCTCTTCTTCCTCCTGGTTGTGGTAGTGCAGTTTTTAACTGGGGTCTTGGCAAATGGCCTCATTGTGGTTGTCAATGCCATCGACTTGATCATGTGGAAGAAAATGGCCCCACTGGATCTGCTTCTTTTTTGCCTGGCAACTTCTCGAATCATTCTTCAATTGTGTATATTGTTTGCACAGCTGGGTCTATCCTGTTTGGTGAGACACACGTTATTTGCTGACAATGTTACCTTTGTCTACATTATAAATGAACTGAGTCTCTGGTTTGCCACATGGCTTGGTGTTTTCTACTGTGCCAAGATTGCTACCATCCCTCACCCACTCTTTCTGTGGCTGAAGATGAGGATATCCAGGTTGGTGCCATGGCTGATCCTGGCATCTGTGGTCTATGTAACTGTTACTACTTTCATCCATAGCAGAGAGACTTCAGAACTTCCTAAGCAAATCTTTATAAGCTTTTCTTCTAAAAATACAACTCGGGTCAGACCAGCGCATGCCACACTACTCTCAGTCTTTGTCTTTGGTCTCACACTACCATTTCTCATCTTCACTGTTGCTGTTCTGCTCTTGTTGTCCTCCCTGTGGAACCACAGCCGGCAGATGAGGACTATGGTGGGAACTAGGGAACCTAGCAGACATGCCCTCGTCAGTGCGATGCTCTCCATTCTGTCATTCCTCATCCTCTATCTCTCCCATGACATGGTAGCTGTTCTGATCTGTACCCAAGGCCTCCACTTTGGAAGCAGAACCTTTGCATTCTGCTTATTGGTTATTGGTATGTACCCCTCCTTACACTCGATTGTCTTAATTTTAGGAAACCCTAAGCTGAAACGAAATGCAAAAATGTTCATTGTCCATTGTAAGTGTTGTCATTGTGCAAGAGCTTGGGTCACCTCAAGGAACCCAAGACTCAGTGACTTGCCAGTGCCTGCTACTCATCACTCAGCCAACAAGACATCCTGCTCAGAAGCCTGTACAATGCCATCTTAA

>Mouse_T2R125--Intact

ATGATGGGTATTGCCATAGATATCTTATGGGCAGCTATTATCATTGTGCAATTCATAATTGGGAATATTGCAAATGGATTCATAGCATTGGTGAACATCATAGACTGGGTGAAGAGAAGAAAAATCTCTTTAATGGATAAGATCATTACTGCTTTGGCAATCTCTAGGATTTATCTGCTGTGGTCTACATTCTTAATTACACTAACATCTTCACTGGATCCAGATATTAAAATGGCTGTGAAAATCATTAGAATAAGCAATAACACCTGGATTATTGCAAATCATTTCAGCATTTGGTTTGCTACATGTCTCAGCATCTTTTATTTTCTCAAGATAGCCAATTTTTCTAACTATATTTTTCTCTACTTAAGGTGGAGATTTAAGAAGGTGGTTTCAGTGACATTGCTAATCTCTCTTATCTTCCTGCTTTTAAATATTTTACTGATGAACATGCATATTGATATCTGGAGTGATAAGTCCAAAAGAAACCTTTCTTTTAGTGTCAGATCAAATAATTGCACTCAGTTTCCCAGACTTGTCCTTTTAATCAACACAATGTTCACATCAATCCCCTTCACTGTGTCCCTGTTGGCTTTTCTGCTTCTCATCTTCTCCCTGTGGAGACACCTGAAAACCATGCAATACTATGCTAAAGGCTCCGAAGACACCACCACAGCTGCACATATAAAGGCCTTGCACATGGTAGTGGCCTTTCTCCTGTTCTACACAGTTTTCTTTTTGTCTCTTGCCATACAATATTGGACCTCTGGGTCTCAAGAGAATAACAACCTGTTTTATGCCACAATTGTAATTACTTTCCCTTCAGTCCATTCATGTATCCTGATTCTGAGAAACAGCCAGCTGAGGCAGGCATCTCTGTTGGTGCTGTGGTGGCTGCTGTGCAAGTCCAAAGATGTACGGATGTTGGTTCCCTGA

>Mouse_T2R11--Intact

ATGACCTCCCCTTTCCCAGCTATTTATCACATGGTCATCATGACAGCAGAGTTTCTCATCGGGACTACAGTGAATGGATTCCTTATCATTGTGAACTGCTATGACTTGTTCAAGAGCCGAACGTTCCTGATCCTGCAGACCCTCTTGATGTGCACAGGGCTGTCCAGACTCGGTCTGCAGATAATGCTCATGACCCAAAGCTTCTTCTCTGTGTTCTTTCCATACTCTTATGAGGAAAATATTTATAGTTCAGATATAATGTTCGTCTGGATGTTCTTCAGCTCGATTGGCCTCTGGTTTGCCACATGTCTCTCTGTCTTTTACTGCCTCAAGATTTCAGGCTTCACTCCACCCTGGTTTCTTTGGCTGAAATTCAGAATTTCAAAGCTCATATTTTGGCTGCTTCTGGGCAGCTTGCTGGCCTCTCTGGGCACTGCAACTGTGTGCATCGAGGTAGGTTTCCCTTTAATTGAGGATGGCTATGTCCTGAGAAACGCAGGACTAAATGATAGTAATGCCAAGCTAGTGAGAAATAATGACTTGCTCCTCATCAACCTGATCCTCCTGCTTCCCCTGTCTGTGTTTGTGATGTGCACCTCTATGTTATTTGTTTCTCTTTACAAGCACATGCACTGGATGCAAAGCGAATCTCACAAGCTGTCAAGTGCCAGAACCGAAGCTCATATAAATGCATTAAAGACAGTGACAACATTCTTTTGTTTCTTTGTTTCTTACTTTGCTGCCTTCATGGCAAATATGACATTTAGAATTCCATACAGAAGTCATCAGTTCTTCGTGGTGAAGGAAATCATGGCAGCATATCCCGCCGGCCACTCTGTCATAATCGTCTTGAGTAACTCTAAGTTCAAAGACTTATTCAGGAGAATGATCTGTCTACAGAAGGAAGAGTGA

>Mouse_T2R113--Intact

ATGGTGGCAGTTCTACAGAGCACACTTCCAATAATTTTCAGTATGGAATTCATAATGGGAACCTTAGGAAATGGATTCATTTTTCTGATAGTCTGCATAGACTGGGTCCAAAGAAGAAAAATCTCTTTAGTGGATCAAATCCGCACTGCTCTGGCAATTAGCAGAATCGCTCTAATTTGGTTGATATTCCTAGATTGGTGGGTGTCTGTTCATTACCCAGCATTACACGAAACTGGTAAGATGTTATCAACATATTTGATTTCCTGGACGGTGATCAATCATTGTAACTTTTGGCTTACTGCAAACTTGAGCATCCTTTATTTTCTCAAGATAGCCAACTTTTCTAACATTATTTTTCTTTATCTAAAGTTTAGATCTAAAAATGTGGTATTAGTGACCCTGTTAGTGTCTCTATTTTTCTTGTTCTTAAATACTGTAATTATAAAAATATTTTCTGATGTGTGTTTTGATAGTGTTCAAAGAAATGTGTCTCAAATTTTCATAATGTATAACCATGAACAAATTTGTAAATTTCTTTCCTTTACTAACCCTATGTTCACATTCATACCTTTTGTTATGTCCACGGTAATGTTTTCTTTGCTCATCTTCTCCCTGTGGAGACATCTGAAGAATATGCAGCACACCGCCAAAGGATGCAGAGACATCAGCACCACAGTGCACATCAGAGCCCTGCAAACCATCATTGTGTCTGTAGTGCTATACACTATTTTTTTTCTATCATTTTTTGTTAAAGTTTGGAGTTTTGTGTCACCAGAGAGATACCTGATCTTTTTGTTTGTCTGGGCTCTGGGAAATGCTGTTTTTTCTGCTCACCCATTTGTCATGATTTTGGTAAACAGAAGATTGAGATTGGCTTCTCTCTCTCTGATTTTTTGGCTCTGGTACAGGTTTAAAAATATAGAAGTATAG

>Mouse_T2R124--Intact

ATGGTACCTGTTCTGCACAGTCTCTCCACCATCATACTAATTGCAGAGTTTGTTTGGGGAAATTTGAGCAATGGTTTGATAGTGTTGAAGAACTGCATTGACTGGATCAATAAAAAAGAGCTCTCCACAGTTGATCAAATACTCATTGTCTTGGCAATTTCAAGAATTAGTCTCATCTGGGAAACACTAATTATATGGGTTAAAGATCAACTAATTTCATCTATTACTATTGAAGAATTAAAAATAATTGTGTTCAGCTTTATACTATCTAGCCACTTCAGTCTCTGGCTTGCTACAGCTCTCAGCATCTTCTATTTATTCAGAATACCTAATTGCTACTGGCAGATCTTTCTCTACTTGAAATGGAGAATAAAGCAACTGATTGTCCACATGCTTCTGGGAAGCTTGGTGTTCTTGGTTGCAAATATGATACAGATAACCATCACTCTTGAAGAGAGGTTCTATCAATATGGAGGAAATACAAGTGTAAATTCCATGGAGACTGAGTTCTCAATTTTGATAGAGCTGATGTTATTTAACATGACTATGTTCTCCATTATACCATTTTCATTGGCCTTAATTTCTTTTCTTCTGCTAATCTTCTCTTTATGGAAACATCTCCAGAAGATGCCACTCAATTCTAGAGGAGATAGAGACCCTAGTGCTACGGCCCACAGAAATGCCTTGAGAATTTTGGTCTCCTTCCTCTTGCTCTATACTATATATTTCCTGTCTCTTCTTATATCATGGGTTGCTCAGAAGAATCAAAGTGAACTGGTTCACATTATTTGTATGATAACTTCACTTGTGTATCCTTCATTCCACTCATATATCCTGATTCTGGGAAATTATAAATTAAAGCAGACCTCTCTTTGGGTAATGAGGCAGCTGGGATGTAGGATGAAAAGACAGAATACACCAACTACATAA

>Mouse_T2R102--Intact

ATGGAATCTGTCCTTCACAACTTTGCCACTGTACTAATATACGTGGAGTTTATTTTTGGGAATTTGAGCAATGGATTCATAGTGTTGTCAAACTTCTTGGACTGGGTCATTAAACAAAAGCTTTCCTTAATAGATAAAATTCTTCTTACATTGGCAATTTCAAGAATCACTCTCATCTGGGAAATATATGCTTGGTTTAAAAGTTTATATGATCCATCTTCCTTTTTAATTGGAATAGAATTTCAAATTATTTATTTTAGCTGGGTCCTTTCTAGTCACTTCAGCCTCTGGCTTGCCACAACTCTCAGCGTCTTTTATTTACTCAGAATAGCTAACTGCTCCTGGCAGATCTTTCTCTATTTGAAATGGAGACTTAAACAACTGATTGTGGGGATGTTGCTGGGAAGCTTGGTGTTCTTGCTTGGAAATCTGATGCAAAGCATGCTTGAAGAGAGGTTCTATCAATATGGAAGGAACACAAGTGTGAATACCATGAGCAATGACCTTGCAATGTGGACCGAGCTGATCTTTTTCAACATGGCTATGTTCTCTGTAATACCATTTACATTGGCCTTGATTTCTTTTCTCCTGCTAATCTTCTCTTTGTGGAAACATCTCCAGAAGATGCAGCTCATTTCCAGAAGACACAGAGACCCTAGCACCAAGGCCCACATGAATGCCTTGAGAATTATGGTGTCCTTCCTCTTGCTCTATACCATGCATTTCCTGTCTCTTCTTATATCATGGATTGCTCAAAAGCATCAGAGTGAACTGGCTGATATTATTGGTATGATAACTGAACTCATGTATCCTTCAGTCCATTCATGTATCCTGATTCTAGGAAATTCTAAATTAAAGCAGACTTCTCTTTGTATGCTGAGGCATTTGAGATGTAGGCTGAAAGGAGAGAATATCACAATTGCATATAGCAACCAAATAACTAGCTTTTGTGTATTCTGTGTTGCAAACAAATCTATGAGGTAG

>Mouse_T2R144--Intact

ATGGCAATAATTACCACAAATTCTGACTATTTTGCTCACAGGTATGAAGTCATAATCCCTTTCGTGGTCTCTACAATATGCTCTATTGTTGGCATCATTGGCAATGGCTTCATCACAGTCATCTATGGGACTGAATGGGTCAGGAGCAAAAGACTCCCCACTGGTGAGAACCTTATGTTGATGCTGAGTTTTTCCAGGCTGTTGCTACAGATATGGATGATGGTAGAGATTACTTATAGTCTACTTTTCCCGATCATTTATAACCATAATGCCATGTATAAACTATTCAAAGCCATCTCTGTGTTTCTAAACTACTGTAACCTCTGGTTTGCTGCTTGGCTCAATGTCTTCTATTGTCTTAAAATTGTGAACTTAGCTCACCCTCTGTTCCTTCTGATGAAGCAGAAAATCATAGGGCTGATGCCTCGGCTCCTGAGTCTGTCAGTGTTGGTTTCCTTCAGCTTAAGTTCCTTCTTCTCTAAAGACATCTTAAATGTGTATGTGAACACTTCTGTTCCCATCCCTTCTTCCAACTCCACAAAGATGAAGTACATCTTTATGATCAATGTACTCAGCCTAGCTTTCTTGTATTATATGGGGATCTTCCTTCCTTTGTTCATGTTCATCATGGCAGCCACTCTGCTGATCACCTCACTCAAGAGGCACACCCTGCACATGGAAAACAGCACCACAGGCTCTAGGGACTCCAGCATGGAGGCTCACGTGGGTGCCATCAAATCGACCAGCCACTCTCTCATTCTCTACATTATTAATGCACTGGCTTTATTTATTTCCATGTCAAACATCCTTGGTGCTTACAGTGTCTGGAATAGTTTGTGCAACATTATCATGACTGCCTATCCAGCCGGCCAGTCAGTGCATCTGATCTTGAGAAATCCAGGGCTGAGAAGAGCCTGGAGGCGGTTTCAGCACCATGTTCATCTTTACCTTAAAAGGTAG

>Mouse_T2R126--Intact

ATGCTACCAACATTATCAGTTTTCTTCATGTTGACCTTTGTTCTGCTCTGTTTCCTGGGGATCCTGGCCAACGGCTTCATTGTGCTGATGCTGAGCAGGGAATGGCTACTGCGTGGTAGGCTGCTCCCCTCGGACATGATCCTCTTCAGTTTGGGCACCTCCCGATTCTTCCAGCAGTGTGTGGGATTGGTCAACAGTTTCTATTACTTCCTCCATCTGGTTGAGTACTCCGGGAGCCTTGCCCGGCAGCTCATTAGTCTTCACTGGGACTTCTTGAACTCAGCCACTTTCTGGTTTTGTACCTGGCTCAGCGTCCTGTTCTGTATCAAGATTGCTAACTTCTCCCATCCTGCCTTCCTGTGGTTGAAGTGGAGATTCCCAGCGTTGGTGCCCTGGTTCTTGTTGGGCTCTATCTTGGTGTCCGTCATTGTAACTCTGCTGTTCTTTTGGGGAAACCACACTATATATCAGGCATTCTTAAGGAGAAAGTTTACTGGGAACACAACCTTTAAGGAGTGGAACAGAAGGCTGGAAATAGACTATTTCATGCCTCTGAAAGTTGTCACCATGTCAATTCCTTGTTCTCTTTTTCTGGTCTCAATTTTGCTGTTGATCAGTTCTCTCAGAAGGCATTCGCTAAGAATGCAGCACAATACCCACAGCTTGCAAGACCCCAACGTCCAGGCTCACAGCAGAGCCCTGAAGTCACTCATCTCATTCCTGGTTCTTTATGCGGTGTCCTTTGTGTCCATGATCATTGATGCTACAGTCTTCATCTCCTCAGATAATGTGTGGTATTGGCCCTGGCAAATTATACTTTACTTTTGCATGTCTGTACATCCATTTATCCTCATCACCAATAATCTCAGGTTCCGCGGCACCTTCAGGCAGCTACTCCTGTTGGCCAGGGGATTCTGGGTGGCCTAG

>Mouse_T2R139--Intact

ATGGCTCAACCCAGCAACTACTGGAAACAAGATGTGCTACCATTGTCTATTTTGATGTTAACACTTGTGGCCACTGAGTGCACCATAGGTATCATTGCAAGTGGGATTGTCATGGCTGTGAATGCAGTCTCATGGGTTCAGAAAAAGGCAATTTCCATAACTACTAGGATTCTGCTTCTTCTGAGTGTATCCAGAATAGGCCTCCAAAGCATCATGTTGATAGAAATTACCTCCTCCATATTCAACGTTGCTTTTTACAACAGTGTTTTATATAGAGTCTCAAATGTAAGTTTTGTATTCTTAAATTATTGTAGTCTCTGGTTTGCTGCTTTGCTTAGTTTCTTCCACTTTGTGAAGATTGCCAATTTTTCTTACCCCCTGTTCTTCAAACTAAAGTGGAGAATTTCTGAATTAATGCCCTGGCTTCTGTGGCTCTCAGTGTTTATTTCCTTCAGCTCCAGCATGTTCTTCAGCAAGCACAAGTTCACTGTGAACAACAACAATTCTCTAAGTAACAACATCTGCAACTTCACAATGAAACTTTACGTTGTTGAGACCAATGTGGTCAATGTGTCTTTTTTATTCATTTCGGGAATACTCCCTCCTTTGACAATGTTCGTCGCAACAGCTACTCTTCTGATTTTTTCTCTCAGGAGACACACCCTGAACATGAGAAACAGTGCCACTGGCTCCAGAAACCCCTGCATAGAGGCTCATATGCAGGCCATCAAAGAAACTAGCTGTTTTCTCTTTCTCTACATTTTAAATGCAGCTGCTCTGCTTCTGTCCACATCCAACATAGTCGATGCTAGTCTCTTCTGGAGTATTGTGATCAGAATTGTTCTGCCTGTCTACCCAGCTGGCCATTCAGTTTTACTAATTCAGAACAACCCTGGATTAAGAAGAACATGGAAGCATCTTCAGTCTCAAATTCATTTGTACTTACAAAATAGATTCTGA

>Mouse_T2R120--Intact

ATGAATTTGGTAGAATGGATTGTTACCATCATAATGATGACAGAATTTCTCTTAGGAAACTGTGCCAATGTCTTCATAACCATAGTGAACTTCATCGACTGTGTGAAGAGAAGAAAGATCTCCTCAGCTGATCGAATTATAACTGCTATTGCCATCTTCAGAATTGGTTTGTTGTGGGCAATGTTAACGAACTGGCATTCACATGTGTTTACTCCAGACACAGACAATTTACAAATGAGAGTTTTCGGTGGAATTACCTGGGCTATAACCAACCATTTTACCACTTGGCTGGGGACCATACTGAGCATGTTTTATTTATTCAAGATAGCCAATTTTTCCAACAGTCTATTTCTTCATCTAAAAAGAAAACTTGACAATGTTCTACTTGTGATTTTCCTGGGATCGTCTCTGTTTTTGGTTGCATATCTTGGGATGGTGAACATCAAGAAGATTGCTTGGATGAGTATTCATGAAGGAAATGTGACCACAAAGAGCAAACTGAAGCATGTAACAAGCATCACAAATATGCTTCTCTTCAGCCTGATAAACATTGTACCATTTGGTATATCACTGAACTGTGTTCTGCTCTTAATCTATTCCCTGAGTAAACATCTCAAGAATATGAAATTCTATGGCAAAGGATGTCAAGATCAGAGCACCATGGTCCACATAAAGGCCTTGCAAACTGTGGTCTCTTTTCTCTTGTTATATGCCACATACTCTTCCTGTGTCATTATATCAGGTTGGAGTTTGCAAAATGCACCAGTCTTCCTGTTTTGTGTGACAATTGGATCCTTCTACCCAGCAGGTCATTCTTGTATCTTGATTTGGGGAAACCAGAAACTTAAACAGGTCTTTCTGTTGTTGCTGAGGCAGATGAGATGCTGA

>Mouse_T2R19--Intact

ATGTCTTTCTCACATTCATTCATCTTCATCGTCATCTTTTGTATGCAGTCTCTAGCTGCTTTGCTGCAAAATGGCTTTATGGCCACCGTGCTGGGCAGGGAATGGGTACGAAGCCAGGGCCTCCCTGCAGGTGACATGATTATGGCTTGCTTAGCTGCCTCCAGGTTCTGTCTGCATGGAATAGCCGTCCTAAACAACTTTCTGGCCTCTGCTATGTTTTGGACCATAAAGAATTATTTTTCTATCCTCTGGGACTTCACCAACACTGTCAATTTCTGGTTTACCACCTGGCTTGCTATCTTCTACTGTGTAAAGATCTCTTCGTTTTCCCACCCCATCTTCTTCTGGATAAAATGGAGAATTTCTCGGTCAGTGCCCAGGTTACTGCTGGGATCCCTGATCATTGGTGGACTGTCAGCCATCTCCTCAGCCACTGGAAACACAATTGCCCTTCAGATGGCGGCCTGTGAAAACTACACAATTTATTATAAAATGATGGCATTCTATCTGTATTATTTTCGCTGTCATGCGATGCTGATGTGGGTCATTCCATTCTTCCTGTTTCTGCTGTCCATCATCTTGCTCATGTTCTCACTGTATCGGCATCTGGAACAGATGAGGTACCACAGACCCAGGACTCATGATTACAGCACCCAGGCTCACATTATGGCTCTGAAGTCCCTTGCCTTCTTCCTCATCTTCTATACATCATATACCCTGCTCCTTACGGTATCTGTTGCACATGTCATAAATGTCCACGGTTCCTGGCACTGGGCCTGGGAAGTGGTAACCTACATGGGCATCTCACTGCATTCCACCATTCTGATACTAAGCAACACCAAGATGAGAAAGGCCCTCAAGATAAAGTTCCCAGACCTTTGTATTCCCAGATCATAA

>Mouse_T2R110--Intact

ATGTTCTCACAGATAATAAGCACCAGTGATATTTTTACTTTTACAATAATATTATTTGTGGAATTAGTAATAGGAATTTTAGGAAATGGATTCATAGCACTAGTGAATATCATGGACTGGACCAAGAGAAGAAGCATTTCATCAGCGGATCAGATTCTCACTGCTTTGGCCATTACCAGATTTCTCTATGTGTGGTTTATGATCATTTGTATATTGTTATTCATGCTGTGCCCACATTTGCTTACAAGATCAGAAATAGTAACATCAATTGGTATTATTTGGATAGTGAATAACCATTTCAGCGTTTGGCTTGCCACATGCCTCGGTGTCTTTTATTTTCTGAAGATAGCCAATTTTTCTAACTCTTTGTTTCTTTACCTAAAGTGGAGAGTTAAAAAAGTAGTTTTAATGATAATACAGGTATCAATGATTTTCTTGATTTTAAACCTGTTATCTCTAAGCATGTATGATCAGTTCTCAATTGATGTTTATGAAGGAAATACATCTTATAATTTAGGGGATTCAACCCCATTTCCCACAATTTCCTTATTCATCAATTCATCAAAAGTTTTCGTAATCACCAACTCATCCCATATTTTCTTACCCATCAACTCCCTGTTCATGCTCATACCCTTCACAGTGTCCCTGGTAGCCTTTCTCATGCTCATCTTCTCACTGTGGAAGCATCACAAAAAGATGCAGGTCAATGCCAAACCACCTAGAGATGCCAGCACCATGGCCCACATTAAAGCCTTGCAAACAGGGTTCTCCTTCCTGCTGCTGTATGCAGTATACTTACTTTTTATTGTCATAGGAATGTTGAGCCTTAGGTTGATAGGAGGAAAATTAATACTTTTATTTGACCACATTTCTGGAATAGGTTTTCCTATAAGCCACTCATTTGTGCTGATTCTGGGAAATAACAAGCTGAGACAAGCCAGTCTTTCAGTGTTGCATTGTCTGAGGTGCCGATCCAAAGATATGGACACCATGGGTCCATAA

>Mouse_T2R130--Intact

ATGACATACGAAACAGATACTACCTTAATGCTTGTAGCTGTTGGTGAGGCCTTAGTAGGGATTTTAGGAAATGCATTCATTGCACTGGTAAACTTCATGGGCTGGATGAAGAATAGGAAGATTGCCTCTATTGATTTAATCCTCTCAAGTGTGGCCATGTCCAGAATTTGTCTACAGTGTATAATCCTATTAGATTGTATTATATTGGTGCAGTATCCAGACACCTACAACAGAGGTAAAGAAATGAGGACCGTTGACTTCTTCTGGACACTTACCAACCATTTAAGTGTCTGGTTTGCCACCTGCCTCAGCATTTTCTATTTATTCAAGATAGCAAACTTCTTCCACCCTCTTTTCCTCTGGATAAAGTGGAGAATTGACAAGCTAATTCTCAGAACTCTACTGGCATGTGTGATTATCTCCCTGTGTTTTAGCCTCCCAGTCACTGAAAATCTGAGTGATGATTTCAGACGTTGTGTTAAGACAAAGGAGAGAATAAACTCTACTTTGAGATGCAAAGTAAATAAAGCTGGACATGCCTCTGTCAAGGTAAATCTCAACTTGGTCATGCTGTTCCCCTTTTCTGTGTCTCTGGTCTCCTTTCTCCTCTTGATCCTCTCCCTGTGGAGACACACCAGGCAGATACAACTCAGTGTAACAGGGTACAAAGATCCCAGCACAACAGCTCATGTGAAAGCCATGAAAGCAGTAATTTCCTTCCTGGCCCTGTTTGTTGTCTACTGCCTAGCCTTTCTCATAGCCACCTCCAGCTACTTTATGCCAGAGAGTGAATTAGCTGTAATATGGGGTGAGCTGATAGCTCTAATCTATCCTTCAAGCCATTCATTTATCCTCATCCTGGGGAGTAGTAAACTAAAACAAGCATCTGTGAGGGTGCTTTGTAGAGTAAAGACCATGTTAAAGGGAAAAAAATATTAG

>Mouse_T2R131--Intact

ATGTATATGATACTGGTAAGAGCAGTATTTATAACTGGAATGCTGGGAAATATGTTCATTGGACTGGCAAACTGCTCTGACTGGGTCAAGAACCAGAAAATCACCTTCATCAACTTCATCATGGTCTGTTTGGCAGCTTCCAGAATCAGCTCTGTGCTGATGTTATTTATTGATGCAACCATACAAGAACTAGCGCCTCATTTCTATTATTCTTACCGTCTAGTAAAATGCTCTGATATATTCTGGGTTATAACTGATCAACTATCAACATGGCTTGCCACCTGCCTGAGCATATTCTACTTATTCAAAGTAGCCCACATTTCCCATCCCCTTTTCCTCTGGTTTAAGTGGAGATTGAGAGGTGTGCTTGTTGTTTTTCTTGTATTTTCTTTGTTCTTATTGATTTCTTATTTTCTACTGCTTGAAACACTTCCTATTTGGGGAGATATTTATGTAACCCTTAAAAACAATCTGACCTTATTTTCAGGTACAATTAAGACCACTGCTTTTCAAAAGATAATTGTTTTTGATATAATATATTTAGTCCCATTTCTTGTGTCCCTAGCATCATTGCTCCTTTTATTTTTGTCCTTGGTGAAACACTCCCGAAGCCTTGACCTGATTTCTACCACTTCTGAAGATTCCAGAACCAAGATTCATAAGAAGGCCATGAAAATGCTGGTGTCTTTCCTCATTCTCTTTATAATTCACATTTTTTTCATGCAGTTAGCACGGTGGTTATTATTTTTGTTTCCAATGAGCAGGCCAATTAATTTCATCTTAACATTAAATATCTTTGCCTTAACTCACTCATTTATTCTCATCCTGGGAAATAGCAATCTTCGACAGAGAGCAATGAGGATCCTGCAACATCTTAAAAGCCAGCTTCAAGAGCTGATCCTCTCCCTTCATAGATTCTCCAGTCTTTACTAG

>Mouse_T2R107--Intact

ATGCTGAATTCAGCAGAAGGCATCCTCCTTTGTGTTGTCACTAGTGAGGCTGTGCTCGGAGTTTTAGGGGACACATATATTGCACTTTTTAACTGCATGGACTATGCTAAGAACAAGAAGCTCTCTAAGATCGGTTTCATTCTCATTGGCTTGGCGATTTCCAGAATTGGTGTTGTATGGATAATAATTTTACAAGGGTATATACAAGTATTTTTTCCACACATGCTTACCTCTGGAAACATAACTGAATATATTACTTACATATGGGTATTTCTCAATCACTTAAGTGTCTGGTTTGTCACCAACCTCAACATCCTCTACTTTCTAAAGATAGCTAATTTTTCCAACTCTGTATTTCTCTGGCTGAAAAGGAGAGTCAATGCAGTTTTTATCTTTCTGTCAGGATGCTTACTTACCTCATGGTTACTATGTTTTCCACAAATGACAAAGATACTTCAAAATAGTAAAATGCACCAGAGAAACACATCTTGGGTCCACCAGCGGAAAAATTACTTTCTTATTAACCAAAGTGTGACCAATCTGGGAATCTTTTTCTTCATTATTGTATCCCTGATTACCTGCTTTCTGTTGATTGTTTTCCTCTGGAGACATGTCAGACAAATGCATTCAGATGTTTCAGGATTCAGAGACCACAGCACAAAAGTACATGTGAAAGCTATGAAATTTCTAATATCTTTTATGGTCTTCTTTATTCTGCATTTTGTAGGCCTTTCCATAGAAGTGCTATGCTTTATTCTGCCACAAAATAAACTGCTCTTTATAACTGGTTTGACAGCCACATGCCTCTATCCCTGCGGTCACTCAATCATCGTAATTTTAGGAAATAAGCAGTTAAAGCAAGCCTCTTTGAAGGCACTGCAGCAACTAAAATGCTGTGAGACAAAAGGAAATTTCAGAGTCAAATAA

>Mouse_T2R140--Intact

ATGAATGCTACTGTGAAGTGTACATTATTAATCATCTTAGGTGTAGAAATCATTATAGGGTGTTTAGGAAATGGATTCATAGCTGTGGTGAACATTATGGACTGGGCCAAGAGAAGAAAGATCTCCTTAGTGGATCAGATATTCACTGCTCTGGCCATCTCCAGACTTGCTTTTGTGTGGTCACTACTCACAGTTTTATTCACATCTGAGCTGTACTCAGCATTAATGACAACAAGAAAAGTGTTGATAATATTTAATAATTCCTGGACAGTGATCAATCATTTCAATATTTGGCTTGCTACATGTCTCAGCATCTTTTATTTTCTCATGATAGCTAATTTTTCAAATTCTATTTTTCTTTCCCTAAGGTGGAGGGTAAAAACTGTGGTTTCAGTAACACTATTGATGTCACTTCTCCTCTTGTTTGTAAACGTTTTAGTCATAAACACATTTATTGTTATCTCAGTTGATGTATATAAAGTAAATACGTCCTACAGTTCTCATTCAGATAACAATATACATATTTCTAGGATTTTTTTATTTACCAACACTATATTCACATTCATACCCTTCAGTGTGACGCTGACAATTTTTCTCCTGCTCATCTTCTCACTATGGACTCATCTGAAGAACATGCAACACAATGCCAAAGACTCCAGAGACCCCAGCACCACAGCCCATATTAAGGCCCTGCAAATGATGGTCACCTTTCTCTTATTATACACCATTTTCTTTCTGGCACTTGTCATGCAATCTTCAAAAATGAAGTTTCTTTCGAGCACTGTGTTCAATTATTTTTTTGAGGTTATATCACTTGCTTTTCCTTCGGGCCACTCTTGTGTCCTAATTCTGGGAAATTCTAAGCTCAGACAGACTTTTATATCCACAGTATGGTGGCTGAAGTCCAGTTTTAATGCTGCAGAACTCCCAGGTCCTTAG

>Mouse_T2R122--Intact

ATGTCGAGCCTACTGGAGATTTTCTTTGTGATCATTTCGGTTGTAGAATTCATAATAGGAACTTTGGGAAATGGATTTATTGTCCTGATAAACAGTACTTCTTGGTTCAAGAATCAGAAAATCTCTGTAATTGATTTCATTCTTACTTGGTTGGCCATCTCCAGAATGTGTGTTCTATGGACAACAATTGCTGGTGCCTCTCTCAGGAAATTCTACAAGACGTTAAGTTACTCTAAGAATTTCAAATTTTGTTTTGACATTATCTGGACAGGATCCAACTATTTATGCATAGCCTGTACAACGTGCATCAGTGTCTTCTACTTGTTCAAGATTGCCAACTTTTCTAATTCCATTTTCTTCTGGATTAAACAGAGAATTCATGCAGTACTTCTGGCTATTGTCCTAGGCACACTCATGTATTTCATTTTATTTCTCATTTTTATGAAAATGATAGCTAATAATTTTATCTACAAATGGACAAAATTGGAACAAAACACAACATTCCCTGTTTTAGATACTCTAAGTGGTTTCTTAGTCTACCATAGCCTCTACAATGGGATTCTCATTTTCTTTTTTATAGTGTCTCTGACCTCATTTCTTCTTTTAATCTTCTCTTTATGGAGCCACCTTAGGAGGATGAAACTACAGGGCATACATACCAAAGACATAAGCACAGAAGCACACATAAAAGCTATGAAAACTATGATGTCATTCCTTTTGTTCTTCATCATATATTATATTAGCAACATTATGCTTATTGTGGCAAGCTCCATTCTTGACAATGTGGTTGCACAAATTTTCTCTTATAACCTAATATTTCTGTATTTATCTGTTCATCCTTTTCTTCTGGTTTTATGGAACAGCAAATTGAAATGGACATTCCAGCATGTATTGAGAAAGCTGGTGTGTCATTGTGGAGGTTATTCTTGA

>Mouse_T2R106--Intact

ATGCTGACTGTAGCAGAAGGAATCCTCCTTTGTTTTGTAACTAGTGGTTCAGTCCTGGGAGTTCTAGGAAATGGATTTATCCTGCATGCAAACTACATTAACTGTGTCAGAAAGAAGTTCTCCACAGCTGGCTTTATTCTCACAGGCTTGGCTATTTGCAGAATCTTTGTCATATGTATAATAATCTCTGATGGATATTTAAAATTGTTTTCTCCACATATGGTTGCCTCTGATGCCCACATTATAGTGATTTCTTACATATGGGTAATTATCAATCATACAAGTATATGGTTTGCCACCAGCCTCAACCTCTTCTATCTCCTGAAGATAGCAAATTTTTCTCACTACATCTTCTTCTGCTTGAAGAGAAGAATCAATACAGTATTTATCTTTCTCCTGGGATGCTTATTTATATCATGGTCAATTGCTTTCCCACAAACAGTGAAGATATTTAATGTTAAAAAGCAGCACAGAAATGTTTCCTGGCAGGTTTACCTCTATAAGAATGAGTTCATTGTAAGCCACATTCTTCTCAACCTGGGAGTTATATTCTTCTTTATGGTGGCTATCATTACATGCTTCCTATTAATTATTTCACTTTGGAAACATAACAGAAAGATGCAGTTGTATGCCTCAAGATTCAAAAGCCTTAACACAGAAGTACATGTGAAAGTCATGAAAGTTTTAATTTCTTTTATTATCCTGTTAATCTTGCATTTCATAGGGATTTTGATAGAAACATTGAGCTTTTTAAAATATGAAAATAAACTGCTACTTATTTTGGGTTTGATAATTTCATGCATGTATCCTTGCTGTCATTCATTTATCCTAATTCTAGCAAACAGTCAGCTGAAGCAGGCTTCTTTGAAGGCACTGAAGCAATTAAAATGCCATAAGAAAGACAAGGACGTCAGAGTGACATGGTAG

>Mouse_T2R118--Intact

ATGGTGCCAACGCAAGTCACCATCTTCTCCATCATCATGTATGTGCTTGAGTCCTTAGTAATAATTGTGCAAAGTTGCACAACGGTTGCAGTGCTATTCAGAGAGTGGATGCACTTTCAAAGACTGTCACCGGTGGAGACGATTCTCATCAGCCTGGGCATCTCACATTTCTGTCTACAGTGGACATCAATGCTATACAACTTTGGTACTTATTCTAGGCCTGTCCTTTTATTTTGGAAGGTATCAGTCGTCTGGGAGTTCATGAACATTTTGACATTCTGGTTAACCAGTTGGCTTGCTGTCCTCTACTGTGTCAAGGTCTCTTCCTTCACTCACCCCATCTTCCTCTGGCTGAGGATGAAAATCTTGAAACTGGTTCTCTGGTTGATACTGGGTGCTCTGATAGCTTCTTGTTTGTCAATCATCCCTTCTGTTGTTAAATATCACATCCAGATGGAATTAGTCACCCTAGATAATTTACCCAAGAACAATTCTTTGATTCTAAGACTACAACAGTTTGAATGGTATTTTTCTAATCCTTTAAAAATGATTGGCTTTGGTATTCCTTTCTTCGTGTTCCTGGCTTCTATCATCTTACTCACAGTCTCATTGGTCCAACACTGGGTGCAGATGAAACACTACAGCAGCAGCAACTCCAGCCTGAAAGCTCAGTTCACTGTTCTGAAGTCTCTTGCTACCTTCTTCACCTTCTTCACATCCTATTTTCTGACTATAGTCATCTCCTTTATTGGCACTGTGTTTGATAAGAAATCTTGGTTCTGGGTCTGCGAAGCTGTCATCTATGGTTTAGTCTGTATTCACTTCACTTCACTGATGATGAGCAACCCTGCATTGAAAAAGGCACTGAAGCTGCAGTTCTGGAGCCCAGAGCCTTCCTGA

>Mouse_T2R136--Intact

ATGATGAGTTTCTTGGTAAGCATTGCATCCATTGCAATGCTGGTGAAAATTGTTCTTGGAACCTTTGCCAATGTCTTCATTGTTCTGGTAAACTTCACTGACTGCATCAAGAAAAGAAAATTCCTCTTAGCTGATAGAATTCTCACTGTTCTGGCTATCTTCAGGTTTGACTTGCTTTGGATAATATTAATGAATTGGAGCTCAAGTGTGTTTCATGTAGGTTTGTATTTCCAAGTAAGATTTTGTATTTGTGTTGTCTGGATAGTAACCAACCATTTTAATACATGGCTTGCAAATATACTCAGCATACTTTATTTGTTGAAGATAGACAATTTCTCAAATCTTATTTTTCTTGGCCTGAAAGGAAAAATTAAGTGTCCTTATATTGTACTTTTGCCATGTTTTGTGCTTTTATTTCCTAATCTTATAATGGTAACCATATGTGAGACAACACAAGCAAATGGACACCAGGGCAACTTGACTGGGAAGACAAAACTGACTTATTTCACGAACCTTATAGCTATGACTTTCACTCTAGGCAGTTTAGTTCCCTTCACCACATTCATGATCTGTTTCCTTCTCTTAATCTGTTCTCTGTGTAAACACCTTAGGACAATGAGGCTTTATGGAAAAGGATCCCAGGGCCCCAGTGCTTCAACCCACATTAAGGTTTTGCAAGTTTTGATCTCATTTCTGTTGTTATTCTCCATGTTTATTCTGTTGCTAATCATATCAGATTACAATTATACAAAGTCTCTGGAGGAACCAATCCACCTGATTTGCCAGGTTATTGGAACCTTGTATCCTTCAAGACATTCTTATATCTTGCTATGGGGAAACAAGAGGATCAAACAGGCCTTTGTGTTGGCAATGGTTCAGGTGAGAGCAAGGTTCTGGCTGAAAGAAAAGAAACCTTGA

>Mouse_T2R109--Intact

ATGGAGCATCTTTTGAAGAGAACATTTGATATCACCGAGAACATACTTCTAATTATTTTATTCATTGAATTAATAATTGGACTTATAGGAAACGGATTCACAGCCTTGGTGCACTGCATGGACTGGGTTAAGAGAAAAAAAATGTCATTAGTTAATAAAATCCTCACCGCTTTGGCAACTTCTAGAATTTTCCTGCTCTGGTTCATGCTAGTAGGTTTTCCAATTAGCTCACTGTACCCATATTTAGTTACTACTAGACTGATGATACAGTTCACTAGTACTCTATGGACTATAGCTAACCATATTAGTGTCTGGTTTGCTACATGCCTCAGTGTCTTTTATTTTCTCAAGATAGCCAATTTTTCTAATTCTCCTTTTCTCTATCTAAAGAGGAGAGTTGAAAAAGTAGTTTCAGTTACATTACTGGTGTCTCTGGTCCTCTTGTTTTTAAATATTTTACTACTTAATTTGGAAATTAACATGTGTATAAATGAATATCATCAAATAAACATATCATACATCTTCATTTCTTATTACCATTTAAGTTGTCAAATTCAGGTGTTAGGAAGTCACATTATTTTCCTGTCTGTCCCCGTTGTTTTGTCCCTGTCAACTTTTCTCCTGCTCATCTTCTCCCTGTGGACACTTCACAAGAGGATGCAGCAGCATGTTCAGGGAGGCAGAGATGCCAGAACCACGGCCCACTTCAAAGCCTTGCAAGCAGTGATTGCCTTTCTCCTACTATACTCCATTTTTATCCTGTCACTGTTACTACAATTTTGGATCCATGGATTAAGGAAGAAACCTCCTTTCATTGCATTTTGTCAGGTTGTAGATACAGCTTTTCCTTCATTCCATTCATATGTCTTGATTCTGAGAGACAGGAAGCTGAGACACGCCTCTCTCTCTGTGTTGTCGTGGCTGAAATGCAGGCCAAATTATGTGAAATAA

>Mouse_T2R105--Intact

ATGCTGAGTGCGGCAGAAGGCATCCTCCTTTCCATTGCAACTGTTGAAGCTGGGCTGGGAGTTTTAGGGAACACATTTATTGCACTGGTAAACTGCATGGACTGGGCCAAGAACAATAAGCTTTCTATGACTGGCTTCCTTCTCATCGGCTTAGCAACTTCCAGGATTTTTATTGTGTGGCTATTAACTTTAGATGCATATGCAAAGCTATTCTATCCAAGTAAGTATTTTTCTAGTAGTCTGATTGAAATCATCTCTTATATATGGATGACTGTGAATCACCTGACTGTCTGGTTTGCCACCAGCCTAAGCATCTTCTATTTCCTGAAGATAGCCAATTTTTCCGACTGTGTATTTCTCTGGTTGAAGAGGAGAACTGATAAAGCTTTTGTTTTTCTCTTGGGGTGTTTGCTAACTTCATGGGTAATCTCCTTCTCATTTGTTGTGAAGGTGATGAAGGACGGTAAAGTGAATCATAGAAACAGGACCTCGGAGATGTACTGGGAGAAAAGGCAATTCACTATTAACTACGTTTTCCTCAATATTGGAGTCATTTCTCTCTTTATGATGACCTTAACTGCATGTTTCTTGTTAATTATGTCACTTTGGAGACACAGCAGGCAGATGCAGTCTGGTGTTTCAGGATTCAGAGACCTCAACACAGAAGCTCATGTGAAAGCCATAAAATTTTTAATTTCATTTATCATCCTTTTCGTCTTGTATTTTATAGGTGTTTCAATAGAAATTATCTGCATATTTATACCAGAAAACAAACTGCTATTTATTTTTGGTTTCACAACTGCATCCATATATCCTTGCTGTCACTCATTTATTCTAATTCTATCTAACAGCCAGCTAAAGCAAGCCTTTGTAAAGGTACTGCAAGGATTAAAGTTCTTTTAG

>Mouse_T2R129--Intact

ATGGATGGAATCGTACAGAACATGTTTACATTCATTGTAATTGTGGAAATAATAATAGGATGGATTGGAAATGGATTCATAGCTCTGGTGAACTGCATACACTGGTACAAGAGAAGAAAGATCTCTGCACTGAATCAAATACTCACAGCCTTGGCTTTCTCCAGAATCTACCTTCTTTTAACAGTATTCACTGTTATAGCAGTGTCTACGCTATACACACACGTGTTGGTAACTAGAAGAGTGGTAAAACTGATTAATTTCCATTTGCTTTTCAGCAATCATTTTAGCATGTGGCTTGCTGCATGCCTTGGCCTTTATTATTTTCTTAAAATAGCTCATTTTCCTAACTCTATTTTTGTTTACTTAAAGATGAGAATTAACCAGGTGGTTTCAGGGACTTTGCTCATGTCTTTGGGCCTCTTGTTTCTAAACACTCTGCTGATAAACTCATACATTGATACCAAGATAGATGACTACAGAGAACATCTACTGTATGATTTCACTTCGAATAATACTGCTTCATTTTACAGGGTTATTTTAGTCATTAACAACTGTATTTTCACATCTATACCCTTTACACTTTCCCAGTCCACTTTTCTCCTGCTCATCTTCTCCCTGTGGAGACATTACAAGAAGATGCAACAGCATGCACAAAGATGCAGAGATGTCCTTGCAGATGCCCACATCAGAGTCTTGCAAACCATGGTCACCTATGTCCTACTCTGTGCCATTTTCTTTCTGTCTCTTTCCATGCAAATTTTGAGGAGTGAGTTGTTGAAGAACATTCTTTACGTTAGGTTCTGCGAGATTGTTGCAGCAGTTTTTCCTTCAGGACACTCCTGTGTCTTAATCTGTAGAGACACAAACCTGAGAGGGACCTTTCTTTCTGTGCTATCGTGGCTGAAGCAGAGGTTTACATCATGGATTCCTAACATAAATTGCAGATCATCTTGCATATTCTAA

>Mouse_T2R138--Intact

ATGCTGAGTCTGACTCCTGTCTTAACTGTGTCCTATGAAGCCAAGATTTCATTTCTGTTCCTTTCAGCCATGGAGTTTGCAGTGGGAATCCTGGCCAACGCCTTCATTGTCTTGGTAAATGTTTGGGATGTGGTAAAAAAGCAGCCCTTGAACAACTGTGACATCGCACTGCTGTGTCTCAGCATCACTCGGCTTTTCCTGCAGGGCCTTCTGCTTCTGGATGCTATTCAGCTCGCCTGCTTCCAGCAGATGAAAGACCCACTGAGCCACAACTACCAAGCCATCCTCACTCTCTGGATGATTGCAAACCAAGTGAGCCTCTGGCTGGCTGCCTGCCTCAGTCTCCTCTACTGCTCCAAGATTGTCCGCTTCTCTCACACCTTTCCACTCCATGTAGCAAGCTGGGTCTCCAGGAGATTTCTTCAGATGCTTCTAGTTGTTCTTCTTCTCTCCTGCATCTGCACTGCCCTTTGTTTGTGGGACTTTTTTTGCAGATCTCACTCCACGGTCACATCTCTACTGCACCTGAACAGCACAGAATTCAGTTTGCAAATTGCAAAACTCAATTTCTTTTACTCGTTTATCTTCTGCAATGTGGGCTCTGTCCCCCCTTCTCTAGCTTTCCTGGTTTCCTCGGGAGTGCTGGTTATCTCCCTGGGGAGTCACATGAGGACTATGAAGTCCCAAACCAGCAGCTCTGGTGACCCCAGCCTTGAGGCCCACATCAGAGCCATCATATTTCTGATCTCCTTTTTCTGTTTTTACGTGGTGTCATTCTGTGCTGCTTTAATATCAATACCCTTACTGATGCTATGGCACAATAAGGGGGGAGTGATGATTTGTATAGGGATGATGGCAGCTTGTCCTTCGGGACATGCAGCCATCCTGATATCAGGCAATGCTAAGCTGAGGAGGGCCATAGAGACCATGCTATTCTGGTTTCAAAGCAGGCAAAAGGTGAGACCAGTCCACAAGGTTCCTCCCAGGACACTCTGA

>Mouse_T2R115--Intact

ATGTGTGCTGTTCTACGTAGCATACTGACAATCATTTTCATTTTGGAGTTCTTCATTGGAAATCTGGGGAATGGATTCATAGCTCTGGTACAATGCATGGACTTACGAAAGAGAAGAACGTTCCCTTCAGCAGATCATTTCCTCACTGCTCTGGCCATCTCCAGGCTTGCTCTGATATGGGTTTTATTTCTAGATTCATTTCTGTTTATACAATCCCCATTACTGATGACTAGAAATACATTAAGACTGATTCAGACTGCCTGGAATATAAGCAATCATTTCAGTATATGGTTTGCTACCAGCCTCAGCATCTTTTATCTCTTCAAGATAGCCATTTTTTCTAACTATCTTTTCTTCTACCTGAAGCGGAGAGTTAAAAGGGTGGTTTTGGTGATACTGCTGCTATCCATGATCCTTTTGTTTTTTAATATATTTTTAGAAATCAAACATATTGATGTCTGGATCTATGGAACCAAAAGAAACATAACTAATGGTTTGAGTTCAAACAGTTTTTCAGAGTTTTCCAGGCTTATTTTAATTCCAAGTTTAATGTTCACATTAGTACCCTTTGGTGTATCCTTGATAGCTTTCCTCCTCCTAATCTTTTCCCTTATGAAACATGTAAGGAAGATGCAGTACTACACCAAAGGATGCAAAGATGTCAGAACCATGGCCCACACCACAGCCCTGCAGACTGTGGTTGCCTTCCTCCTATTATATACTACTTTCTTTCTGTCTCTAGTTGTGGAAGTTTCAACACTTGAAATGGATGAAAGTCTGATGCTTCTGTTTGCAAAAGTTACTATAATGATTTTTCCTTCCATCCACTCCTGTATTTTCATTTTGAAACATAATAAGTTGAGACAGGACTTGCTTTCAGTACTGAAGTGGCTACAGTATTGGTGCAAGCGTGAGAAAACCTTGGATTCATAG

>Mouse_T2R121--Intact

ATGGGAAGCAATGTGTATGGTATCTTAACTATGGTTATGATTGCAGAGTTTGTATTTGGAAATATGAGCAATGGATTCATAGTGCTGATAAACTGCATTGATTGGGTCAGGAAAGGAACTCTTTCTTCCATTGGTTGGATCCTGCTTTTCTTGGCCATTTCAAGAATGGTGTTGATATGGGAAATGTTAATAACATGGATAAAATATATGAAGTATTCATTTTCATTTGTGACTGGAACAGAATTACGGGGTATCATGTTTACCTGGGTAATTTCCAATCACTTCAGTCTCTGGCTTGCCACTATTCTCAGCATCTTTTATTTGCTCAAAATAGCCAGTTTCTCCAAACCGGTTTTTCTCTATTTGAAGTGGAGAGAGAAGAAAGTGCTTCTGATTGTCCTTCTGGGAAATTTGATCTTCTTGATGCTCAACATATTACAAATAAACAAACATATAGAACACTGGATGTATCAATATGAGAGAAATATAACTTGGAGTTCTAGAGTGAGTGACTTTGCAGGGTTTTCAAATCTGGTCTTATTGGAGATGATTGTGTTCTCTGTAACACCATTCACAGTGGCCCTGGTCTCCTTCATCCTGTTAATCTTCTCCTTGTGGAAACATCTACAGAAAATGCATCTCAATTCTAGAGGGGAACGAGACCCCAGCACTAAAGCCCATGTGAATGCCTTGAGAATTATGGTCTCCTTCCTCTTACTCTATGCCACTTACTTCATATCTTTTTTTCTATCATTGATTCCCATGGCACATAAAACACGACTGGGTCTTATGTTTAGCATAACTGTTGGGCTTTTCTACCCTTCAAGCCACTCATTTATCTTAATTTTGGGACATTCTAATTTAAGGCAAGCCAGTCTTTGGGTGATGACATATCTTAAATGTGGGCAAAAGCATTAG

>Mouse_T2R33--Intact

ATGGTGTTGACAATAAGGGCTATTTTATGGGTAACATTGATAACTATTATAAGTCTGGAGTTTATCATAGGAATTTTAGGAAATGTATTCATAGCTCTCGTGAACATCATAGACTGGGTTAAAAGAGGAAAGATCTCTGCAGTGGATAAGACCTATATGGCCCTGGCCATCTCCAGGACTGCTTTTTTATTGTCACTAATCACAGGGTTCTTGGTATCATTATTGGACCCAGCTTTATTGGGAATGAGAACGATGGTAAGGCTCCTTACTATTTCCTGGATGGTGACCAATCATTTCAGTGTCTGGTTTGCAACATGCCTCAGTATCTTTTATTTTCTCAAGATAGCTAATTTCTCAAATTCTATTTTCCTTGTTCTCAAATGGGAAGCTAAAAAAGTGGTATCAGTGACATTGGTGGTATCTGTGATAATCTTGATCATGAACATTATAGTCATAAACAAATTCACTGACAGACTTCAAGTAAACACACTCCAGAACTGTAGTACAAGTAACACTTTAAAAGATTATGGGCTCTTTTTATTCATTAGCACTGGGTTTACACTCACCCCATTCGCTGTGTCTTTGACAATGTTTCTTCTGCTCATCTTCTCCCTGTGGAGACATCTGAAGAATATGTGTCACAGTGCCACAGGCTCCAGAGATGTCAGCACAGTGGCCCACATAAAAGGCTTGCAAACTGTGGTAACCTTCCTGTTACTATATACTGCTTTTGTTATGTCACTTCTTTCAGAGTCTTTGAATATTAACATTCAACATACAAATCTTCTTTCTCATTTTTTACGGAGTATAGGAGTAGCTTTTCCCACAGGCCACTCCTGTGTACTGATTCTTGGAAACAGTAAGCTGAGGCAAGCCTCTCTTTCTGTGATATTGTGGCTGAGGTATAAGTACAAACATATAGAGAATTGGGGCCCCTAA

>Mouse_T2R117--Intact

ATGAAGCACTTTTGGAAGATATTATCTGTTATCTCCCAGAGCACACTTTCAGTCATTTTAATCGTGGAATTAGTAATTGGAATTATAGGAAATGGGTTCATGGTCCTGGTCCACTGTATGGACTGGGTTAAGAAAAAGAAAATGTCCCTAGTTAATCAAATTCTTACTGCTTTGTCAATCTCCAGAATTTTTCAGCTCTGTTTATTGTTTATAAGTTTAGTAATCAACTTTTCATATACAGATTTAACTACAAGTTCAAGGATGATACAAGTCATGTACAATGCTTGGATTTTAGCCAACCATTTCAGCATCTGGATTGCTACATGCCTCACTGTCCTTTATTTTCTAAAGATAGCCAATTTTTCTAACTCTTTTTTTCTTTATCTAAAGTGGAGAGTTGAAAAAGTAGTTTCAGTTACACTGTTGGTGTCATTGCTCCTCCTGATTTTAAATATTTTACTAACTAACTTGGAAACCGACATGTGGACAAATGAATATCAAAGAAACATATCATGCAGCTTCAGTTCTCATTACTATGCAAAGTGTCACAGGCAGGTGTTAAGGCTTCACATTATTTTCCTGTCTGTCCCCGTTGTTTTGTCCCTGTCAACTTTTCTCCTGCTCATCTTCTCCCTGTGGACACATCACAAGAGGATGCAGCAGCATGTTCAGGGAGGCAGAGATGCCAGAACCACGGCCCACTTCAAAGCCCTACAAACTGTGATTGCATTTTTCCTACTATATTCCATTTTTATTCTGTCTGTCTTAATACAAATTTGGAAATATGAATTACTGAAGAAAAATCTTTTCGTTGTATTTTGTGAGGTTGTATATATAGCTTTTCCGACATTCCATTCATATATTCTGATTGTAGGAGACATGAAGCTGAGACAGGCCTGCCTGCCTCTCTGTATTATCGCAGCTGAAATTCAGACTACACTATGTAGAAATTTTAGATCACTAAAGTACTTTAGATTATGTTGTATATTCTAG

>Mouse_T2R137--Intact

ATGTTTGGATTCATTGAAGGGGTGTTCCTGGTTCTGACTATCACTGAGTTTATTCTTGGAAATCTGGTGAATGGTTTCATTGTGTCAATCAATAGCAGCTATTGGTTCAAGAGCAAGAAGATTTCTTTGTCTAACTTCATCATTACCAGCTTGGCCCTCTTCAGGATCTTTCTGTTGTGGATTATCTTTATTGATAGTCTTATAATAGTGTTCTCTTACCAGACTCATGACTCAGGGATAATGATGCAACTAATTGATGTTTTCTGGACATTTACAAACCACTTCAGTATTTGGCTTATCTCCTGTCTCAGTGTTTTCTACTGCCTGAAAATAGCCAGTTTCTCCCACCCCTCATTCCTCTGGCTCAAATGGAGAGCTTCTAGAGTGGTTGTTGGGATGCTGTGGGGCGCACTGCTCTTATCCTGTGTCAGTACCATGTCTCTGATGAATGAATTTAAGATCTATTCTGCCCTCACTAGAAGCAAAGACACACCAAATATGACTGAATACATCAGACTGAAGCGACAGGAATATAATCTGATGCATGTTCTTGGGAATCTGTGGAAGATTCCTTCCTTAATTGTTTCCCTGGTTGCCTACCTTCTGCTGCTCCTCTCTCTGGGGAAGCACACACAGCAGATGCAGCAATACAGTATTGACTCCAGAGATCAGAGTGCTGAGGCCCACAAAAGAGCCATGAGAATCATCTCTTCCTTTCTCCTATTCTTCTTATTCTACTTTCTTTCCTTTATGATTTTGTCATCCAGTCGTTTCCTACCAGAAACCAGGATCGCCAGGATAATTGGAGTAGTGATTTCAATGTCATACCTTGTTGGTGATTCATTTATTCTCATAGTATGTAACAACAAGCTGAAGCATACATTTGTGGCCATGCTCCCATGTGAGTGTGGTCATCTGAAACCTGGATCTAAGGGACCCTCTGCTTCATAA

>Mouse_T2R116--Intact

ATGAATGGTGTCCTACAGGTTACATTTATAGTCATTTTGAGTGTGGAATTTATAATTGGCATCTTTGGCAATGGATTCATAGCGGTGGTGAACATAAAGGACTTGGTCAAGGGAAGGAAGATCTCTTCAGTGGATCAGATCCTCACTGCTCTGGCCATCTCCAGAATTGCACTGCTGTGGTTAATATTAGTAAGTTGGTGGATATTTGTGCTTTACCCAGGACAATGGATGACTGATAGAAGAGTTAGCATAATGCACAGTATATGGACAACATTCAACCAGAGTAGTCTCTGGTTTGCTACAAGTCTCAGCATCTTTTATTTTTTCAAGATAGCAAATTTTTCCAACCCTATTTTTCTTTATTTAAAGGTCAGACTTAAAAAAGTCATGATAGGGACATTGATAATGTCTTTGATTCTCTTTTGTTTAAATATTATCATTATGAATGCACCTGAGAACATTTTAATCACTGAATATAATGTATCTATGTCTTACAGCTTGATTTTGAATAACACACAGCTTTCTATGCTGTTTCCATTTGCCAACACCATGTTTGGGTTCATACCTTTTGCTGTGTCACTGGTCACTTTTGTCCTTCTTGTTTTCTCCCTGTGGAAACATCAGAGAAAGATGCAACACAGTGCCCATGGATGCAGAGATGCCAGCACTAAGGCCCACATCAGAGCCTTGCAGACATTGATTGCCTCCCTCCTCCTGTATTCCATTTTCTTCCTGTCTCATGTTATGAAGGTTTGGAGTGCTCTGCTTCTGGAGAGGACACTCCTGCTTTTGATCACACAGGTTGCAAGAACAGCTTTTCCGTCAGTGCACTCCTGGGTCCTGATTCTGGGCAATGCTAAGATGAGAAAGGCTTCTCTCTATGTATTCCTGTGGCTGAGGTGCAGGCACAAAGAATGA

>Mouse_T2R143--Intact

ATGCCCTCCACACCCACATTGATCTTCATTATCATCTTTTACCTGGTGTCATTGGCCTCTATGTTGCAGAATGGCTTCATGATGATTGTGCTGGGCAGAGAGTGGATGAGGAACCGGACACTACCGGCAGCTGACATGATTGTGGCCTCTCTTGCTTCCTCCCGGTTCTGCTTGCATGGGATCGCCATCCTGGCCAACCTCTTGGCCTCCTTTGATTTTTGTTACCAAGCGAACCTTATTGGCATCCTCTGGGATTTCACTAACACTCTCATTTTTTGGCTTACTGCCTGGCTTGCCATCTTCTACTGTGTGAAGATCTCCTCTTTCTCCCACCCTGTCCTCTTTTGGCTCAAGTGGAGGATTTCCCAGTTAGTTCCCAGGCTGCTGGTTGTATCTCTCATCATAGGTGGCCTGTCAGCTGTCATTTCAGCCACCGGGAACTTCATGGCCAATCAGATGACCATCTCCCAGGGTTTCCATGGAAACTGCACTTTTGGTCACATGTCACTGGACTTCTATCGGTACTATTACCTGTATCACTCAGTGCTCATGTGGTTCACTCCTTTCTTCCTGTTTCTAGTGTCCGTTATCGTGCTCATGTTCTCACTGTACCAGCATGTGGAGAAGATGAGGGGCCACAGGCCTGGGCCTTGGGATCTCCATACTCAGGCACATACCATGGCTCTGAAATCCCTTACCTTCTTCTTCATCTTTTATATCTTTTTTTTCTTGGCCCTGGTAATTTCTAGTACAAAAAGGAAAAGCATGCAGAGTTACTATTGGGCCAGAGAGGCTATCATCTACACAGGCATCTTTTTGAACTCCATCATCCTGCTGTTTAGCAACCCCAAACTGAGAAAGGCCCTGAAGATGAGGTTTTAG

>Mouse_T2R123--Intact

ATGTTTTCACAGAAAATAAACTACAGCCATTTGTTTACTTTTTCAATCACCTTGTATGTGGAAATAGTAACGGGAATCTTAGGACATGGATTCATAGCATTAGTGAACATCATGGACTGGGTCAAAAGAAGAAGGATCTCTTCAGTGGATCAGATTCTCACTGCTTTGGCCCTTACCAGATTCATTTATGTCTTGTCTATGCTGATTTGCATATTGTTATTCATGCTGTGCCCACATTTGCCTAGGAGATCAGAAATGCTTTCAGCAATGGGTATTTTCTGGGTAGTCAACAGCCATTTTAGCATCTGGCTTACTACATGCCTCGGTGTCTTTTATTTTCTCAAGATAGCCAATTTTTCTAACTCTTTTTTTCTTTATCTAAAGTGGAGAGTTAAAAAAGTGATTTTAATAATAATCCTGGCATCACTGATTTTCTTGACTTTACACATTTTATCTTTAGGGATATATGATCAGTTCTCAATTGCTGCTTATGTAGGAAATATGTCTTATAGTTTGACAGATTTAACACAATTTTCCAGTACTTTCTTATTCTCCAACTCATCCAATGTTTTCTTAATCACCAACTCATCCCATGTTTTCTTACCCATCAACTCCCTGTTCATGCTCATACCCTTCACAGTGTCCCTGGTAGCCTTTCTCATGCTCATCTTCTCACTGTGGAAGCATCACAAAAAGATGCAGGTCAATGCCAAACAACCTAGAGATGTCAGTACTATGGCCCACATTAAAGCCTTGCAAACTGTGTTCTCCTTCCTGCTGCTGTATGCCATATACTTACTTTTCCTTATCATAGGAATTTTGAACCTTGGATTGATGGAGAAAATAGTGATACTGATATTTGACCACATTTCTGGAGCAGTTTTTCCTATAAGCCACTCATTTGTACTGATTCTGGGAAACAGTAAGCTGAGACAAGCCAGTCTTTCTGTGTTGCCTTGTCTAAGGTGCCAGTCCAAAGATATGGACACCATGGGTCTCTAG

>Mouse_T2R135--Intact

ATGAGCACAGGCCATACAGTTCTTGGATGTCAGACTACTGATAAGACAGTCGTCACCTTATTTATCATTTTAGTCCTTTTGTGCCTGGTGGCAGTGGTAGGCAATGGATTTATCATTATAGCACTGGGCATGAAATGGTTGCTCCGGAGAACACTGTCAGCTCATAATAAGTTACTGATCAGTCTAGCAGCCTCTCGATTCTGTCTCCAATGTGTGGTGATAGGTAAGAATATTTATGTTTTCCTGAATCCAACAAGCTTCCCATACAACCCTGTAATACAGCTCCTAAATTTAATGTGGGACTTCTTGACTGCTGCAACCATCTGGCTCTGTTCTTTGCTAGGTTTCTTCTATTGTGTGAAAATTGCAACCTTAACCCATCCTGTCTTTGTCTGGCTAAAGTACAGGTTGCCTGGGTGGGTACCATGGATGCTGCTCAGTGCTGTGGGGATGTCGAGCTTAACTAGTATCCTATGTTTCATAGGCAATTATATGATATATCAGAACCATGCAAAGAGTGGCCATCAACCTTGGAATGTCACTGGGAATAGCTTAAGACACTCACTTGAGAAATTCTACTTCTTTTCTATAAAGATAATCATGTGGACAATTCCCACTGTTGTCTTCAGCATCTTCATGAGTTTGCTCCTCGTATCTTTGGTAAGACACATGAAGAAGACTTTCTTGGCCCTTTCAGAACTTCGGGATGTCTGGGCACAGGCCCATTTCAAGGCTCTTCTTCCTCTGCTCTCCTTCATCGTCCTTTTCATCTCCTGTTTTCTGACGCTGGTACTCAGTTCTGCCAGCAACACACCATATCAGGAATTCAGGTACTGGATGTGGCAGGTGGTGATTCATCTGTGCACAGTGATACATCCCATTGTTATACTCTTCAGCAACCCTGTTTTGAGAGTGGTGATAAAGAGGGGCTGCTGCTGA

>Chicken_T2R2--Intact

ATGTTACCACCAGTTCTCATCGTTTCAATGTGCATTGTTGCTATTGAAGTTGTTGTTGGATTTATGGGAAATGGATTTATTACCGCTGTTAATAGCATTAACTGGATCAAAAGCAAAAGAATATCTGCAGCTGATATGATCCTGATCTTCCTGAGCACATCAAGATTTATTTTGCAGGTGACTGTAATGATGTACATTCACAGTCTCTACTTTACAGATGTGTTTAAGTTGGCTTCTGTGTACAAAACTTTTGGTGCTATATGGATGTTTGTAAACCATGCTAGTTTGTGGTTCAGTACTTGGCTCTATGTCCTCTACTGTGTTAAAATAATCAATGTCACCCAATGGTTGTTGCTACAAATCAAGCACAGAATAACTGCGATGATTCCATGGCTGCTTCTAGGATCACTGTTAATTTCTTCTGTAACTTCATTTCCTTTATTGTGGATTACACCCAACACCTACCTCTGCAGGTCAACGGGGAACTGTGGAGACAATAGCACAGCACATATCAGTGGCTGGGATAGTTCACATTTGTACCTGCTTCTTCTTTACTTTACAGGTTGTTTTTTTCCTCTGGTACTATCTATGATAACTTCAGGCCTATTAATTAATTCTCTGTGGAAACACACCAAGAAGATGCAATGCTATGTAGATAGCTTCAGGGATCCTTTGATAGATGTTCACCTAATTGCCATCAAGTCCATTATTTCTTTCCTGATCCTATATATTTCCAGTTTTGTTGCTCAAATTCTTTTGACTTTGTCAACTTCTCAAAGCAAAGATTTTGTGAAGGTTGCAGTATCTTTAGTTGTAACTGGAGCATATCCTTCAATACACTCTATTATCCTGATCATAGTTAATTCAAAGCTGAAACTGGCATTGAGGATGCTTTGCCAACATTTTATGTGTCATTTGGAAAATGTGAATCTCTAA

>Chicken_T2R7--Intact

ATGGCAGAGCAGCACAACACAACTTCATCCAGTAGCGCCTTCGTGGTCATCTTCGCCTTCCAGGCGCTGGCCGGCATGGGGATCAATGCCTTCATTGTGGCTGCGTCTTGTATGGCGTGGTTCAAGAAGAAGGGCATGAACTCTAACGAGAAGATCTTACTGTTTCTGGGCTGCTTCAGGTTTTGGTATCTGTGCGCCACGTGGATATACCTCATCATTTCAGTGTTGTTTCCCCAGAACCTTTTAGACACAGGCATCTCTCTCACTTTTGCAATTTTTATGTGCTTTTTAAGCTCTTCCAACCTGTGGACTTCTACCTGCCTCTATGCTTTTTACCTTATGAAAATTGCCAATTTCAGGCACCATTTCTTCATCTACCTGAAAGCCAGAATCGACAGGATTGTGCCGTGGCTGCTGCTGAGCTCCGTGGTTCTGTCCCTGCTCAACTGCAGCCCTTTCCTCAAAGTTATTGATGAAGAGAACAGAACCAGCCCCAATTTCACCACCCAAGGGATTTTCTGGAAAACGAACGAAGAAATAAGGAAACATTTTAACAGCATAATTTCCATCTGCACATGTGGGTTTTCCATGGCATTCATATTGGTAACCCTCTTTGCCTTCTTCCTCCTCTTCTCCCTCTGCAGACACAAGCACAAGATGCAGACCAGCTCCACCAGGAGCCTCAGCATGGATGCCCACATCAAAGCCATGAAATCCCTTCTGTCCTTCTTCTTCACCTTCAGCATTCATTACATCTTGTTGATCACAACAGTGTATTACAGCAGCAAGGAAAATTTCCTGGTGTTACTCCTTTTGGTCCTTCAATACTCTTTTCCGGTCATTCATTCCCTTATTCTGATTTTTAGCAACCCCAGACTGGAAAGGATAGCACTAAGGATTCTGCCCTGTGCAAAGTGCAAGGAGTGCGCCAGGCAGCCCACCGAAACACCGATGCTTTGTTCTTAA

>Chicken_T2R1--Intact

ATGTCCAGTTTATTTTCTTCTTTCTGTCTAGTAATTGCCATATTTGAGTCAGTTGTGGGGCTTCTAGGAAATGGGACTATCGTGGCTGTCAGTTCAACTAGTTGCATCAGGAGCAAAATTTTGTCCTCCTATGATGTGATTGTGATTTTTCTGAGTTTATCCAGGTTCTTCTTGCAGCTCTGGATGATCCTGGATTTCCTCCTAATTTTTTTTTGTCAACCCTCCTATTATGAAGAAAATTTATTTGTAACTTTCAAGACAGTTTTTATATTCCTGAACTCTTATAGCTTTTGGTTTGCTGCCTGGCTTAGTGTCTTCTATTGTGTCAAGGTTGCCAGTTTTACCCAGTCATTCCTCAGCTGGCTGAAGCAAAGGATTGCCAGTCTCATACCCTGGATGCTGATAACATCATCTCTTTTTTCCTTTGCAACCTCTCTTCCTTTCTTCTGGGATAGCTACAACGCACACAGCAACTTCACTACTCCTTTAACCATGACAAACTCTTCAAAAAGGATAACCACAAGGAAAACCAATTTGATTTTTTTGATCCTTCTCTGTAATGTTGGTATAGCTTTGCCTTCAATAATGCTTGTTTTTTCAAGTATCCTGCTGATTAGATCTCTGTGGAGGCACACCAGACAGATGCAAAATAATGCAACTGGCTTCAGGGATCCCAGCTTAGAAGCTCTTATTGGTGCCATCAAGACAGTCTTCTCCTTCCTCCTCCTGTACATTACAAATTTTATCGCTTTGATTCTCATTTTATCTGACACTTTTGTACCTTTAAGCACTGAGGAAGCTATATGTGTTGTTGTAGTGGCTGCCTGTCCTGCAGGACAGTCTATGGTCTTAATCTGGAGCAACCCCAGATTTCGAGAGCTGCTCAGTAGCATTTTGCACTACGTAAACTCTTGTGTCAGAGCTAGATGCAGCTGA

>Cow_T2R1--Intact

ATGCTTCGGATAGTCTTTTTTTCTTCTGTCGTTGTCTCTGAAATTTTAACTTTTGTAGGACTCATTGTGAATCTCTTCATTGTAGTGGTCAGTTACAAGACTTGCATCAAAAGCCACAGGATCTCTTCTTCTGACAGACTCCTGTTCAGTTTGGGCATCACCAGATTTTTTATACTGTTACTGAATGTTGTTGTCATCATCTCTCCAAATATGGAAAGGTCAGTCTCCTTATCCTATTTTTTTCTGTCATGTTGGATGTTTTTGGACTGTAGTAGTCTTTGGTTTGTAACCTTGCTCAACGTCTTGTATTGTGTGAAGATTGCTAACTACCAACACTCAGTGTTTCTCCTGCTGAAACGAAATCTCACCACCAAGATGCCCCGGCTGCTGCTGGTCTGTATGCTGCTTTCTGTCTTCACCACTCTCCTGTATATTATGCTCAGACAGTTGGCACCCTCTCTTGAATTTGTGACTATGAGAAATGGCACAGTATTTGACATCAATGAGGGACTCTTGTCTTTGGTGACTCCTTTGGTCTTGAGCTCATTTCTCCAATTCATCATTAATGTGACTTCTGCTTCTTTGTTAATCAATTCCTTGAAGAGACATATACAGAAGATGCAGAGAAGTGCCACTGTTCTTTGGAATCCCCAGACTGAAGCTCATGTGGGTGCTATGAAGCTGATGATCTGTTTCCTCGTACTCTACATTCCATATTCAGTTGCTACCCTGGTCCATTATCTCCCTCCTTCTATAGGGATGGATTTGAGAACCAAGTCTATTTATGTTATTATGTCCACCATTTACCCTCCAGGACATTCTCTTCTTATTATTCTCACACATCCTAAACTGAAAACAAAAGCAAAGAATATTCTTTGTTTCAGTAAATAG

>Cow_T2R10--Intact

ATGATAATGTTTATGTCAAACATTGTTTCCATTCTATTAATGACAGAATTTGTTCTGGGAAATTTTGCCAATGTCCTCATAGCACTGGTGAACTGCAATGACTGGACCAAGAGACCAAAGATCTCAGCTGATGGGATTCTCACTGCTCTGGCATTCTGCAGAATTGTTATGCTCTGGGCAATGTTAATAAATTGGTATGTAATTGTGTATAATTTAACTCTATATAATTCAGAAGTAAAAATTATTGTTCATGTTGCCTTGACAGTAAGCAACCATTTTAGTAACTGGCTTGCTACTAGCCTCAGTATATTTTATTTGTTGAAGATAGCCAATTTCTCCAGCCTAATTTTTCTTCACCTGAAGTGGAGAGTTAAAAGTGTAGTTCTCATGATGATGTTGGGGACGTCATTGTTCTTGTTTTTTCAAGTTGCAGTGTTAAGCATGGATGAGGCTATTCAGACAAATGAATATGAAGGAAATACCACTCAGAAGATCAAACTAAGGGACATTTTACACCTTTCAAATGTGACTCTGTTCACACTAACAAACTTTATACCCTTCACTATGTCCTTGACATCTTTTCTGCTGCTAATCTTTTCCCTGTGGAAACATCTCAGGCAGATGCAGCTCAATGGTAAAGGATCCCAAGATCCCAGCACCAAGGTCCACATAAAAGCCATGCAAACTGTCATCTCCTTTCTTTTCCTGTTTGTTATTTACATTCTGGCTCTAATTGTATCAGTTTGGAATTCTAATCAGCTGCAGAAAGAACCAGTCCAAATGCTTTATGATGTTGTTTTAATCATGTATCCTTCAATCCACTCATGTTATCTTGATCTGGGGAAATGGGAAATTAACTCAAGCCTTTCTGTCATTTCTATGGCAGTCAAGATGCTGGCTGAAGGAAAGGAAATAGGTGGAAACATATGTCTTTTAGCATAA

>Cow_T2R11--Intact

ATGTCAAATGTCATCAAATATGTTTTTTTGATCATTGAAATCTCAGAATTCATAACAGGAATTTGCGGAAATGGATTCATTGCACTAGTACTTTGTGCTGACTCTCTCAAAAGCAAGACTATCTCCTTGCTTGACTTCATCTTCACATGCTTGGCCATCTCCAGAATTGGTATGGTATTCATACTTCTCCTGGATGGCATTAGAATAGTGTTCCATCCAGAAATATTAGATAGTCATCAGGTAATAGAAGTAACTTTTGATTTCTTCTGGAATCTGAGCAATTCCTTAGCTACCTGGTGTGCTGCCTGCCTCAGCATCTTCTACTTCCTCAAGCTATCTAATTTTTCCCACCCCTTCTTTCTCTGGCTAAAATGGAGAAGAAATAGAGTTGTTTTCACCATTATGTTGGGATTCTGTCTCTCTTTGTTTTTTAATCTTCTGAACATAAAGTTCAATACTCTCAGGGTCAGTGACCATTTAGAAATAGAAAACAACTTGACTTGGGAAAAATGCATGCCTAAAACACAGTACTACAGCAGTCAAATTCTCCTCCACCTGGGATCTCTCATCCCCTTGGCTGTGTCACTCATTTTATTTTTCCTGTTAATATTTTCCTTATGGAGACATACCAGGCAGATGACACATCATGCCAAAGGATCCAAAGACCTCAACACAGGAGTTCTTGTGAGAACAAGAAATACTTTGACTTCTTTCATCATTCTCTTAGTTGTGCACTATTTGGCTACATTCATGTTAACTTGGTTCTATTTCACACTAGAAAATGACGTGACTTTTATTGCTGCTCAAACTGTAGCATTTCTCTATCCTGCAATTCACCCTTTTATTCTGATTCTGGGGAGCAGGAAACTGAGACAGATTTCTGTGAATCTGCTAAGGCAAATTGAATCCTGTGTCAAGCGATTGTAA

>Cow_T2R12--Intact

ATGCTGAGTATAGTAGAAGGCCTCCTCCTTTTTGTAGCAGTTAATGAGTCAGTATTGGGGGTTTTAGGGAATGGGTTTATTGGACTAGTAAACTGCATTAACTGTGTGAAAAATAAGAAGATCTCTACACTCAGCCTTATTCTCACTGGCTTAGCCTCTTCCAGATTTTGTCTGATATGGATAATAACTACAGATGCATATGTGAGGGTGTTTTCTCCAGATATGTATTTGTCTGGTAATCTAAGTCAATATATAGCTTACTTATGGATAATTATGAATCAATCAAGTGTCTGGTTTACCACTAGCCTCAGCATCTTCTACTTCCTGAAAATAGCCAACTTTTCCCACTGCATTTTTCTCTGGCTGAAGGGTCACATTACTGAGATTCTTCTTCTTCTAATGGGATGTTTGCCCATTTCATGGTTATTTACTTTTCCAAACATTACAATGCCTTTTATTAATAATATTATGAAGAACAGAAGCACAACCGGGTTGGTCACCATGCAGAAAAGTGAATACTTTATAAATCAGATTTTGTTCAATCTTGGAACATTTCTTGTCTTTGTACTATGCCTGATTACATGTTTCTTAATAATCACTTCCCTTTGGAGGCACAACAGGAGGATGCAATTGAATGCCACAGGATTCAGAGACCCCAGTACAGAAGCACACATCAAAGCAATGAAGATTTTGGTGTCTTTTATCATCCTCTTTATCCTGTATTTTGTAGGCACTGCCATACAAATATTAAGTGTGACAGTGCCTGAAAACAAACTGCTATTTATTTTTGGTATGACAACCACCATCCTCTATCCCTGTGGACACTCATTTATCCTAATTCTTGGAAACAGCAAGCTTAACCAAGCCTCTTTGAGGGTACTGAAGCTATTAAAGTGCTAG

>Cow_T2R13--Intact

ATGGAAGATTCCTTGGAAAACATCTTTATCACTTTAATAAATTCAGAATTCATAATTGGCATTCTGGGGAATGGGTTCATAACACTGGTGAACTGCACTGACGAGATCAAGATGCAAAAGGTCTCCTTGGCTGATCAAATCCTCACTGCTTTGGCAATTTCCAGAATTGGTCTGATTTTGGTAATGATAGTGAGTTTGTTTACAAAGGAGTCTTATCCATCTTCATCTTTAGACATAAAGGGAAATAAAGTCATACTTTTTAGTATTGCTGGGCTCTTGGCCAATCATTTTAGTGTCTGGCTTGTCACAGGCCTCAGCCTCTTCTATTTCCTCAAGATAGTCAATTTTTCAAATGCTGTTTTTCTTCACCTAAAGTTTAGAATTGGAATGGTAGTTATGGTAATGTTTCTGGGGACATTAGTATTGCTGCCTTTAAGTCTTACTCTGGTGAGTAGCTATATTAATATCAAGATACATCCGTATGAAAGAAATATGACTTTAAATTCTAAAAGGCATGACACTGAAACCTTTTCCAAATTAATTATATTCACCGTAGGATCTTTCTTACCTTTTATTATATCCCTGAGTTGTTTTCTCCTATTAATGTTCTCCCTACTGAAACATGTCAAGAAGATGAGGAGCCATGCAACAGGATTCAGAGATCCCAGCAGCAAAGCCTACGTCAGAGCCATGATCATGGTGATATCTTTTCTCATACTACTTGCCATTCACTTCCTATCTCATCTCATGACAACTTTTCATCACAATGTGATACAGAGTGAACTGGCCTTTATGCTTGCTGAAGCTCTTGGAACTATTTACCCTTCAGTTCACTCATTTGTCCTGATTCTGGGAAATGACAAGCTAAGAAAAGCTTCACTTTTGGTGCTGTGGCAGTTGAGGTGTGGCTGA

>Cow_T2R14--Intact

ATGCCATCTGGAATTGAAAACACTTTTCTAGCAGCAACAATAGGAGGATTCCTGATTGGAATTTTGGGGAATGGGTTCATTGTACTAGTTAACTGCATTGACCTGGTGAAGAGACAAAAGCTCTCATCAGCTGACTGCATCCTCACAGGCCTGGCTATCTCCAGAATCAGTCAACTTTGGGTAATACTATGTGACTCATTTTTATTGGTACTATGGCCACACCTATATGCCATTGATAAACTAACAAAAGTTGTTAGTAGTTTTTGGATATTGTCCAATCACCTAGCTACCTGGTTTGCCACCTGTCTAAGTGTTTTCTACTTCTTTAAAGTAGCCAACTTCTCCCACCCCTGCTTCACTTGGCTGCGGTGGCGAATTCGTAGTGTGGTACTGGTGCTTCTCTTGGGGTCTTTGTCCTTACTGTTTTTGAATTATGAATCAATATATACACTTAGTCATATCTTAACTAACAGCTACAAAATATATGTAAGAAACTCAACGTGGTCCTCAGATGTAAGTGAAACTCATTATCTTCACCAGTTGATTGTTTTTAACTTCATCAACTTAATCCCCTTTCTTCTGTCCCTGACCTCACTGCTCCTCTTAGTTCTCTCCTTGATGAGACACATCAGGAATTTGCAGTTCAACCCCAGCTCAAAGGATCTCAGCACAGAGGCCCATAAAAGAGCCATGAAAATCGTGATGTCTTTCCTCTTCCTCTTCATCATTCATGTCTCTTCCATCCTATTAATAGCTTGGGTTTTCCTTAAACTGCAGGGACGTCTGGCCCAATTGGTGGTTGTGTTAACTTCGACTGTTTTTCCTTCAAGCCACTCCTTTATCCTAATTTTGGGAAATAGCAAGCTGAGACAGAATGCCTTACGACTACTGTGGTATCTTAACTGCCACCCGAAAAGAGTGAAATCTTTAGCTTCATAG

>Cow_T2R15--Intact

ATGGAGAGAACATTGAACAATATACTTACGATCATTTATGCTGGAGAGTTCTTACTGGGTATTTTGGGAAATGGATTCATTGTTCTGGTTAACTGTATTGATTGGATCAGGAGTAGGAAGTTCTCCCTGATTGACTTTATTCTCACCTGCTTGGCTATTTCCAGAATATGTGTGCTGTGCATAATGATTTCAAGTACAGGTTTATATGTAATCTCTAAGGAAATACGGTACAATAAGAATCTCCTGATAAATTTGAGGTTCCTCTGGACAGGATCCAATTATTTCTCCATAGTCTGCACCACCTGCATCAGTGTCTTCTATCTCCTCAGAATAGCCAACTTTTCGAATTTCCTTTTCCTCTGGATGAAATGGAGAATTCACAAGGTGCTTCTCATTATTGCACTGGGGGCTGTCTTCTCTTTCTGCTTGTGCCTTCTTCAAAAGGATGCGGTAGTTGAAAGCCGGCTCCAAAACCAGGTAAACAGCGAAAACAATGTGACATTGGACTTTCTAATGATAAAATATGATTTGTTCCTTACCATAATGTTCCTCATCCCCTTTGTAGTGTCCCTGGCCTCCTTTCTCCTTTTAATCCTCTCCTTATGTGGTCATCTCAGGCGTATGAACGGTGTAGACTGTAGCTCGGAGGCCCATGTGAGAGCCCTGAAGGCTATGATTTCATTCCTACTCCTCTTCGTTCTATACTATTTGAGCACTATTATAACTGTGTGGGCCAATCACATTCTAGGTAGTTTTGTGGCAAAGATTTTTGTGAACATGCTGTTATTTTTCTGTCCTTCTGGCCACACTTTGCTTCTGATTTTGTGGAACAGCAAATTGAAACAGGCTTCACTCTGTGTCCTAAGGAAGCTGAAGGGTTACATGAATCTAAGAAAACCTGCTCTTCCAAAAAGAAGCCTGAAGCGATGA

>Cow_T2R16--Intact

ATGGTGACTCTGACTCACATCGTATCTGTGCCCTCTGAAGTCAGGAATGCATTTCTGTTCTTTTCAGTCCTGGAGTTTGCAGTAGGGATCCTACTCAACGCCTTCATTTTCTTGGTCAATTTCCGGGACCTGGTGAGGAGGCAGCCACTGAGCCACTGTGATCTTGTCCTATTGAGTCTCAGCCTCACCCGGCTTGTCCTACACGGGCTGCTCTTTCTGAAGGCCATCCAGCTTACTCATTTCCAGCGGATGAAAGACCCGCTGAGCTTCAGCTACCAGACCATCATCGTACTCTGGATGATCGTCCACCAAGCCGGACTCTGGCTCACCATGTGCCTTAGTCTCCTCTACTGCTCCAAGATTGTCCGTTTCTCTCACGCCTTCCTGCTCCATGCAGCAAGCTGGATCTCCAGAAAGATCCCCCAGATGCTTCTGGGTGCTATGGTTCTCTCCTGTGTCTGCACTCTTCTCTGCTTATGGGACTTTTTTAGTGGATCTCATTTATCAGCTGTAACTAGGCTACTCATGAATAACAGTACTGAACTCAATTTGAACATTGCAAAACTCAGTTTCTTTCATTCCTTCCTCTTCTGCAGCCTGGCATCCATCCCTTCTTTCTTGCTTTTCCTGGTTTCCTCTGGGATGCTGGTGTTCTCCCTGGGGAGGCATATGAGGATGATGAGGGCCAAAACCAGAGGCTCTGGGGACCCCAGCCTGGAGGCTCACACACGGGCGCTCAGGTCTCTTGTCTCTTTCTTCTGCCTGTATGTGCTGTCACTCTGTGCTGCCTTATTCTCGATACCGTTGCTGATGCTGTGGCACAGCAAGGTCGGGGTGATGGTCTGCATAGGGATAATGGCAGCCTGTCCCTCAGGACATGCAGTCATTCTGATCTCAGGGAATGCCAAGCTGAGGAGGGCTGTGGACACCATTCTGCTTTGGGCAAAGAGCAGCTTCAAGGTAAGGGTGGACCACAAGGCATATCCCAGGACGCCAGATCTGTGTTGA

>Cow_T2R17--Intact

ATGATCTCTTTGTCAGGTATTCCTCATGTTATCATCATGTCAGCAGAATTTATCACAGGGGTTACAGTAAATGGATTTCTTATAATCATCAACAGCAATGAATTGGTCAAAAGCAGAAAGCTAACACCAATGCAACTCCTGTTCGTATGTATAGGGATATCTAGATTTGGTCTACAGATGGTGTTAATGGTACAAGGTTTTTTCTCAGTGTTCTTTCCACTCTTTTATAGCGCAAAAATTTATGGTACACCAATGCTGTTTTTTTGGATGTTTTTCAGCTCTGTCAGTCTCTGGTTTGCCACCTGTCTCTCTTTATTTTACTGCCTCAAGGTTACAGGCTTTACCCAGTCCTGTTTTCTTTGGCTGAAAGTCAGGATCTCAAAGTTAATGCCTTGGATGCTTCTGGGAAGCCTGCTGACCTCTGTGAGCATTGCAGCTCTGTGTGTCAAGGTGGATTACCCTAAAATTGTGGATATTGATATCCTCGGGAATGCCACAGCTAAGAGGACTAAACTCAACACAAAGCAAATTAATGAAGTTCTTCTCATCAACTTGGCATTAATATTTCCTCTGACTATATTTATAATATGCACTGTTATATTATTAATTTCTCTCTACAAGCACACTCATCGGATGCAAAATGGACCTCTTGGTTTTAGAAACACCAGGACTGAAGCCCATATTAATGCATTAAGAACAGTGATAACATTCTTTTGCTTCTTTATTTCTTACTTTGGTGCCTTCATGGCAAATATGACATTCAATATTCCTTATGGAAGTCATTGCTTCTTTGTGGTAAAGGATATTATGGCAGCATATCCCTCTGGTCATTCAGTTATAATGATTTGGAGTAATTCTAAGTTCCAGCAACCAATCAGGAGACTTCTCTGCCTAAGAAGGAGTCAATGA

>Cow_T2R18--Intact

ATGTTTGTGAATGGATGCATTGGACTAGTAATATGTGTTGATTGGATTAAGAAGAAAAAGATCTCCATAGCTGACTACATCCTCACCAGTTTAGCTCTCTCCAGAATGTATTTGCTTTGTGTAATGACACTCAACGGCACCATACTGGCACTCTACCCAGGTGTTTATGAAAATGAGAAAATAAAGGTAGTTCTTAATATCTTCTGGACATTCACCAACTACTTAAGTATGTGGTTTGCCACCTGCCTCAATGTCTTCTGTCTCTTCGAGATAGCCAATTTCTCCCACCGACTTTTTCTCTGGCTGAAGTGGAGAATTGAGAGGGTGGTTCACTGGAGCCTACTGGGGTCCCTGGCCATTTCCATGTTGATCAGCCTTATACAAGCAACGTTAACAAATTCTGATTATGATTTTCTTAAAATTGCAAAACATAAAAGAAACGTCACCGAATTGTTCCATGTGAGTAAAATTCAATACTTCGACCCATTGACATTGTTTAACCTGTTTGCTATTATTCCATTTACTGTGTCATTGATCTCATTTTTCTTTTTAATTACATCCCTGTGGAGATACAGTAAACAAATGAAATCCAGTGTTACAGGCTCCAGAGACTCCAGCACAGAGGCCCACGTGGAGGCCAGGAAAACAGTGACCTCATTTCTTTTCTTCCTTTTTGTATACTACCTGGCCTGTCTTTTGGCAACATTTAGCGACTTTATGAAAGAAAGCAAGTTAGCTATGATGTCTGGAGAGATTATAGAAATTCTTAATCCCTTAGGTCACTCACTGTTTTTTATTGTTGGAAATAACAAGCTGAGGCTGGCATCTGTCAGGACGCTGAGATGTGGGAAAACAGCCTGCATGATGTGA

>Cow_T2R2--Intact

ATGTTGAGACTCAGCAATATGGGGTTTCTGGTTCTGACCGCCATTCAGTTCATCCTGGGAATGCTGGGGAATGGTTTCATAGGGTGGGTCAATGGCAGCAGCTGGTTCAAGAGCAAGAGGATCTCTTTGCATGACTTCGTTATCACTAACCTGGCTGTCTCCAGGATTGTTTTGCTGTGGATTCTCTTGATCGATGGTATTTTACTGGTGTTCTTTCCCAAACTACATGATGAAGGGATAATCATGCAAATTATTGATGTTTTCTGGACATTTACAAACCATCTGAACATTTGGCTTACCACCTGTCTCAGTGTCTTCTACTGCCTGAAAGTGGCCAGTTTCTCCCATCCTATGTTCCTCTGGCTCAAATGGAGAGTTTCCAGGGTGGTTGTATGGATGCTGTTGAGTACCCTGCTGTTATCATGTTGCAGTGCCATCTCTCTGATCCGGGAATTTAAGATCTATTCTGTTCTTGGTGGAATTGATAGAACCGGGAATATGACTGAACTTTTTAGAAAGAAAGAAAAAGAATATAAACTGATCCATGTTCTTGGGACTCTGTGGGACCTCCCTCCCCTAGTCATATCGCTAATCTCCTACTTTCTGCTTATCCTCTCCCTGGGGAGGCATATGCGGCAGATGCATCAAGACTGTGCCAGCTCCAGAGATCTCAGTACCGAGGCCCACAGGAGGGCCATCAGAGTCATCCTCTCCTTCCTCTTTCTCTTCCTACTCTACTATCTTTCCTTTTATGTTTTAACATCCAGTTATTTCTTACCAGCAACTAAGATGATTGCAAAGATTGGAGAAGTAATTGCAATGTTATATCTTGCTGGCCACTCCTATGTTCTCATTCTGGGAAATAGTAAGCTGAAGCAGATGTTTGTGGCGATGCTACGGTGTGAGCCTGGTTGTCTGAAGCCTGGATCCAAGGGATCTGTTTATCCATAG

>Cow_T2R3--Intact

ATGAACGGAGGGGACATGGTTCCTGGACCTCAGTTGGTTGATAAGACAGCCCTTGTCTGCATTATTATTTTATTCCTTTTGTTCCTGGTGGCATTGGTAGGTAATGGCTTAATCATCATGGCACTGGGCAGCGAGTGGCTGCTGCAGAGAACGTTGTCGCCTTGCGATAAGTTATTGGTCAGCCTGGGGGCCTCTCGCTTCTGTCTGCAATGGGTGGTGATTAGTAAGAACATTTACATTTTCCTGAATCCCATGGCCTTCCCATACAACCCCGTGTTCCAGCTCCTGGCCGTTCAGTGGGACTTCTGGAACTCTGCAACACTGTGGTTCTCCACCTGGCTCAGTGTCTTCTACTGTGTGAAAATTGCCACCTTCACCCACCCCGTCTTCCTCTGGCTAAAGCGGAATGTATCTGGGTTGGTTCCTTGGATGCTACTCAGCTCTCTGGGGTTCTCTACCTTTACCACCATTCTATTTTTCATAGGCAACCACAGAATGTATCAGAACTATATAAAGAAGGGTCTGCAACCTTGGAATGTCACTAGGAATGCTGTGAGAACATATGAGAGGTTCTGCCTCTTCCCTTTGAGAATTGTTACCTGGACCGTCCCTACTGTTATCTTTATTGTGGGCACAGTTTTGCTCATTACATCTCTGGGAAGACACACCAAGAAGGTCTTCTTCTCCATCTCAGGCTTTCACAGTTCCAGTGCCCAGGCACACATCAAGGCTCTCTTGGCTTTTATCTCCTTTGCTATCTTCCTCACTTCCTCTTTTCTGTCACTGGTTCTCACTGCCTCAGGTATGTTTCCTTTTGGGGAATTCCGGTTCTGGATATGGCAGACTGTGATTTATCTGGGTACAGCAATCCACCCCCTTATTCTTCTCTTGAGTAACCGCAGGCTGAGAGCTCTGCTAGGGAGGGGCTGCTCCTCAGCACATGGGGCATCTTGA

>Cow_T2R4--Intact

ATGACAACCAGCCAACTCTCTGTCTTCTTCATGATTATCTATATGCTCGAGTTCTTGATAATAACTGGGCAGAGCAGCCTGATTGTTGTAGCGCTGGGCAGAGACTGGGTGCAGACTCAAAGGCTGCCACCTGCGGACATGATTCTCATCAGCCTGGGCATCTGCCGCTTCTGTCAACTGTGGTCATCGATGCTGTACAACTTTTGTTCCCACTTCCACCCTAATTACAATTTTTGGTATTTCGGGATCATCTGGGAATTTACTAACATCCTTTCCTTCTGGTTGACCAGCTTGCTTGCTGTCTTCTACTGTGTCAAAGTCTCTTTCTTCAGCCACCCCGTCTTCCTCTGGCTGAAGTGGAGAATTGTGAGATGGGTTCCTCGGCTGTTGCTGGGCTCTCTGCTGATTTCTTGTGTGTCTACCATATTTCCAGCTACTAGTTATTACATTGATATTCAATTCATCGCCATGAAGCATTTCCCTAGAAACAGCACCATGCTTGAGAGACTTGAGGCGTTCCTGTGGGATTTTTCCACACTGCACAAAGTAGTTGTGTTGGTTATTCCTTTCCTCCTGTTCCTGGCCTCCACAGTCTTGCTCATGGCCTTATTATCCCGACATCTGAAGCAGATGAAAGACCTTCACACAGGCTGCTCCAACTCCAGCCCGGAAGCTCACTCTGCCGCCCTGAGGTCCCTTGCCATCGTCCTCATCTTGTTCACCTTTTATTTTCTCACCGTGCTCCTCTCCATATTGGATGTCCTATTTAATAAAGAGTCCTGGTTCTGGGCCTGGGAAGCTATCATCTATGCATTAGTCTCTATTCATTCTACTTTACTAATGCTGAGCAGTGTCAAACTGAAAAGAGTTTTAAAGGCAAGGTGCTGGAGCCTAGAAGCTGCCTGA

>Cow_T2R5--Intact

ATGATCCAAACCTGCAGTTCCTCAGAAAATGATCTGTCACCATCTCTTGTCACTTTGATGTTAATAATTATCGGCACGGAATGCATCCTTGGTATCCTCGCAAATGGGTTCATTGCAGCGATAAATACAGCTGAATGGATTCACAGTAAGGTACTCTCCACCAGTGGCAAGATCCTGCTTTTCCTGGGTGTATCCAGAATAGTTCTACAAAGCTTCATGATGCTAGAACTTACCTTAAGCTCAACATCCCCACAGTTTTATAATGATGACATCATGTATCACACATTCAGAGGATGTTTCATGTTCTTAAATCACTGCAGCCTCTGGTTTGCTGCCTGGCTCAGTGTCTTCTACTTCGTGAAGGTGGCGGATTTCTCCTACCCCCTTTTCCTCAAGCTGAAGTGGAGAATTTCCGGACTGATGCCCTGGCTTCTGCAGCTATCAGTGTTTGTTTCCTTGGGCCAGAGTGTGCTCTTCTTCCAAAACAACTATACTATGAATAGTAACAATCTTTTTTCTCTCCCGTCCTTCAACTCCACTAAGAAAAAGTCCTTCGCGGAGTCCGCTGTGATCAACCTGGTTCTTTTCCTTAACCTGGGGATCTTCATCCCTCTGATCATGTTTATGCTGGCGGCCACCCTGCTGATCATCTCTCTCAAAAGACACATCTTCCACATGAAAAGCAACGCCACTGGCTCCAGAGACCCCAGCATGGAGGCTCACCTGGGGGCCATCAGAGCCATCAGCTATTTTCTCATTCTCTATATTTTCAAAGTACTTGCTCTCTTTCTCTACATGTCCAACTTCTTTGACATCAATAGTCCCTTGAATATTTTGTGCAAAATCATCATGGCTACCTACCCTGTGGGCCATTCCATTCTACTGATTCAGGACAATCCTGGGCTGAAAAGAGCCTGGAAGAGGCTTCAGACTCAAGTTCACCTTTATTTTAAAAAGTAG

>Cow_T2R6--Intact

ATGCTGGAGTCTCACCTTGTTAGCCACCTTGTTTTGGCAGTGGTACACCTTCTCTTGGGGATTTTAGTAAATGGCATCATTGTGATTGTGAACGGTACTGACTTCATCAAGCAGAGAAAGTTGATCCCGCTGGATCTCCTTGTTTCCTGCTTGGCGATTTCCAGGATGGGAATTCAGCTGGCCTTCTTCTACACTAACCTGGCTCTTCTTTCCTTGATCAAATTCCCTCAATTTACTGAGACGCTTGTAGTTTTCACATTTGTAAATGATTTGGGACTTTGGTTTGCCACCTGGCTCAGTGTCTACTACTGCACCAAGATTGCTACCATCGCTCACCCGCTCTCGTTCTGGTTGAAGATGAAGATCTCCAAGTTGGTTCCTTGGCTGATTCTTGTGTCCCTGCTGTATGCATGTAGTACTTCTGCTATGCATGTCAAATATAAGTGGGTATTTTATGGAGAAGACTTCCTGGGCCTTTTCTTCCCAAATGTAACAACTCACATCAAAGTAACCCCTACCTTACAGTTTGCCTTTCTGTTTGCTGAGTTTGCATTGCCATTGTTCATCTTCCTGATTTCTTCTCTGCCCTTGATATTTTCCTTGGGAAGACATGCCTGGCAGGTGAGAAACACATGGACAGGCCCCAGAAACCCTCACACACGTGCGTACATCAGGGCCTTTCTCTCCATCCTGTCCTTCTTGGCCCTCTATCTCTGCCACTACCTGATCATTGCTTTGATCTTTTTTCAAATTTTTAACCTCAGAAGCTTTCTATTTCTGTTCTGCACCTTCGTGGTTGGTTCATACCACTCCGTCCACTCTATTACTTTAATTTTAGGAAACCCGAAAATGAAACAAAATGCAAAGGCATTGCTCCTCCTCAGAAAGTGA

>Cow_T2R7--Intact

ATGTTCCCTGGGTTGAGTACCATCTTTCTGATACTATCAGGAGTGGAATTCTTAATCGGAATTCTAGGCAATGTGTTCATTGGACTGGTACTCTGCTCTGAATGCGTTAAGAACCAAAAGACATCTTTATTTGACTTCATTCTCACTGGCTTGGCTATCTCCAGAATCAGTCAACTGTTGGTGTTTTTTGTGGAGTCACTTATGATGGGACTAGATTCACAGGTATTTGCCATTTTTAAACTAGCAAAACCCATTACTTTACTTTGGAGAATATCTAATCATTTGACTACCTGGCTTGTCACCTGTCTAAGTATTTTCTATCTCCTTAAGATAGCTCATTTCTCCCACTCTCTTTTTTTCTGGCTGAAGTGGAGAATGAACAGAGTCATTCTTGCGATGCTTGCATTTTCTTTGGTCTTTCTGATTTTGGATATTCTTTTGCTAGAAACATTTAATGATCTCTTCTGGAATTTAATAAATGAAGGCAATTGGACTTTAGTTGAAAGTAAAACTCATTATATTAAAAGCGAGAATCTTCTTAGTTTCTCCTATTTCATTCCTATTGTTCTGTCCCTGCTCTCATTGTTTTTTTTATTTTGGTCCTTGGTGAAACACACCAGAAATTTGCAGCTCAATTTTATGGGTTCCAGGGACTTCAGCACAAAGGCCCATAAAAGAGCCATGAAAATGGTGACGTCGTTCCTCCTCCTTATTATGGTTCATTTTCTTTTTACACAATTGGCAAATTGGATGTTTCATAGGTTTTTGGACAATAAGTTCACAAAGTTCATCATGTTAGCACTATATGTCTTTCCTTCAGGCCACTCGTTCATGTTGATTCTGGGAAATAGCCAGTTAAGACAGATAGCCTTGAAGGTACTGAAGCATCTTAAAAGCTCCTTGAAAAGACAAAATCCATTGGCTTTATAG

>Cow_T2R8--Intact

ATGCTGAATATAGTGGAAGGCCTCCTCATTTATGTAGCAGTCAGTGAATCAGTATTGGGGGTCTTAGGGAATGGATTTATTGGAGTTGTAAGCTGCATTGACTGTGTGAAAAGCAAGAACATCTCTACTGTCAGCCTTATTCTCACTGGCTTAGCCTCTTCCAGATTTTGCCTGATATGGATGATAATTACAGATGCATATATAAGGATGTTTTTTCCAGATATATATTTGTTTGGTAATATAAGTCAATATATAGTTTACTTAAGGATAATTATGAATCAATCAAGTACCTGGTTTGCCACCAGCCTCAGCATCTTCTATTTCCTGAAGATAGCCAATTATTCCCACTGCATTTTTCTCTGGCTGAAGTGTCACATCAACAGGGTTCTTCTCCTTTTCATGGGGTCCTTGCTTATTTCATGGTTATTTGCTTTTCCAAGCATTGCAAAGCCTAGTACCAATAATATTATGAAGAACAGAAGCACAACCTGGCTGATCACCATGCATAAAAGTGAATACTTGACAAATCAGATTCTGCTCAATATTGGAGTCATTCTTGTCTTTGTACTATGCCTGATTACATGTTTCTTATTAATCACTTCCCTTTGGAGACACAACAGAAAGACGCGATTGAGTGCCACAGGATTCAGAGATCCCAGCACTGAAGCACATATCAAAGCAATGAAGATTTTGGTGTCTTTTATCATCCTCTTTATCTTGTATTTTGTAGGCACTGCCATACAAATATCAGGTAGTAGTACTATGCCTGAAAACAAACTGTTGTTCATTATTGGTATAACAACCAGACTCCTCTATCCCTGGGGACACTCATTGATTCTAATGCTAGGAAACAGGAAGCTGAAGCAAGACTCTTTGAGGGTACTGAAGCCATTAAAGTGCTGGGAAAAAGAGAAACTTCTTAGAATTCCATGA

>Cow_T2R9--Intact

ATGCTGAGTGTACTGGAAGGCCTCCTCATTTTTGTAGCACTTAGTGAGTCAATATTGGGGGTTTTAGGGGATGGATTTATTGGACTTGCATACTTCATTGAATGTGTGAAGAACAAGAAGTTTTCTACTATCAGCTTTATTCTCATGGGATTGGCTACTTCCAGAATTTGCCTGATAGGGTTAATAACTACCGATGGATTTGTGAAGATTTTTTCTCCAGAAATGTATTCCTCTGGTTACCTAATTGACTGTATTACTTACTCATGGGTAATTCTGAATCCAACAAGTGTCTTTTTTGCCACCAGCCTCAGCATCTTCTATTTCCTGAAGATAGCCAATTTTTCCCACCACATTTTTCTCTGGTTGAGGAGTGACGTCAAAAGGGTTCTTCTCCTTCTGATAGGATACTTGCTTATTTCATGGTTAGTTACTTTTCCACTAACTATGAAGATAATTAGTGATTCTAGAGCAAAGAATAGAAGTGTAGTCTTTTCAGTTGAAGTGCATAAAGGTGAATTCTTTAGAAACCAGATTTTGCTCAATCTTGGAACCCTTACCATCTTCATACTATGCCTGATTACATGTATCTTATTGCTCATTTCCCTTCGGAGGCACAACCAGAGGATGCTACTGAATGCCACAGGATTCAGAGACCCCAGCACAGAAGCACATATCAAAGCAATGAAAGTTTTGATATCTTTTATCATCCTTTTTATTTTGTATTTTATAGGCATTACCATAGAAATATCATGCACTACTATGTCAGAAAGCAAGCTGTTGTTTATTTTTGGTCTGACCATCACTGCCCTCTATCCCTGGGGACACTCATTTATCCTAATTCTAGGAAACAACAAGCTAAAGCAAGTTTTTTTGAGAGTACTGAAGCAATTAAAATGCTGGAAGAAAGAGAAGCTCCTCAGAACTCCTTGA

>Dog_T2R12--Intact

ATGGCAGGCACAATGAAGAATGTATTTATGATGATTTTTGCCGGAGAATTCATAATAGGGATTTTGGGAAATGGATTCATTATATTGGTTAACTGTATCGATTGGATCAGGAGCTGGAAGTTCTTCCTGATTGACTTTATTCTTACCTGCTTAGCCATTTCCAGGATATTTCTGCTGTGCATAATAATGTTAGGCATAGGTCTAGATATAATTTGTAAGGAAATATGGTACAATGATAATCAACTGATAACCTTTGAAGTCCTCTGGACAGGATGCAATTATTTCTGCACAATCTGTACTGTGTGCCTCAGTGTCTTCTACTTCCTCAAGATAGCCAACTCTTCCAATCCCATTTTCTTCTGGCTAAAACGGAGAATTCACAGACTGCTTCTCATTATTGTCCTGGGAGCAGTCTTCTATTTCTGCTTGTCCCTGCTTTTGAAGGATATAGTATTTAAGAACATGATCAAAACCAAGGTAAACACTGAAAGCAATGTGACATTAAATTTCACAGCGAGAAAATATGATTTACTAACTTCTAATATATTCCTGAACATGCTATTCGTCATCCCCTTTGCAGTGTCTCTGGCTTCCTTTGTCCTTTTGATCCATTCCTTATGGAACCATACCAGGCGGATGAAGGGCATTGATTCTGGGGATCTTATCACAGAGGCCCATGTAAGAGCCATGAAGTTTATGATTTCATTCCTGCTATTCTTCTTTATATACTATTTGAGCAATATTATAATATATTTTGCCTATGTTGTTCTGGATAGTCTGGTGGCAAAAATTTTTGCTAATATATTAGTATTTTCCTATCCTTCTGGCCATCCATTTCTTCTGATTTTATGGAACTGCAAATTGAAACAGGCTTCTCTCTATGTCCTGAGGAAGCTGAAGTGGTGCATGAATCTAAGGAAACCCGCATACATAAAGCATACCTGA

>Dog_T2R40--Intact

ATGGCCACAGTGAGCACAGATGCCACGGATAGAGACATGTCCAGGTTTAAAATCGTCCTCACCTTGGTGGTCCCCGGAATAGAGTGCCTCACTGGCATCGTTGGGAATGGCTTCATCACAATCATCCATGGGGCCAAGTGGGCCAGAGGCAAAAGGCTCCCGGTCACTGACTGCATTCTGCTGATGCTCAGCTTTTCCAGGCTCTTACTGCAGATCTGGATGATGCTGGAGAATATTTACAGTCTACTATTCCGGGTCACTTACAACCAAAGCACAGTGTTTATAGTCTTCAAAGTCACTGTCATTTTCCTGAACTATTTCAACCTCTGGCTTGCTGCCTGGCTCAACATCTTCTATTGTCTGAGAATCACAAACTTGGCTCACCATGTGTTCTTCATGATGAAGAGGAAAATCACGGAGCTGATGCCTCGGCTTCTGGGACTGTCACTGTTCATCTCCTTATGCTTCAGCTTTCCTTTCTCTACAGATATCTTCCATGTGTACGTAAACAGTTCCATCCCTATCCGTTCCTCCAATACCACCGAGAAGAAGTACTTCTCTGAGACCAATGTGGTCAACCTGGTTCTTCTCTATAACCTGGGGATCTTCATTCCTCTGATCATGTTCATCCTTTCGGCCACCCTGCTGATCATCTCTCTCAAGAGACACACACTACACATGGAAAGCAATGCCACTGGCTGCAGGGACCCCAGCATGGAGGCTCACTTTGGGGCCATCAGAGCGACCAGCTACTTTCTCATTCTCTACATTTTCAATGCAGTTGCTCTATTTCTTTCCATGTCCAACATCTTCGACATCAACAGCTCCTGGAATATTTTGTGCAAAATTGTCATGGCCGCCTACCCAGCTAGCCACTCAGTGCTACTGATCTTGGGTAACCCTGGGCTGAGAAGAGCCTGGAAGAGGTTTCAGCACCATGTTCCTCTTCACCTGTAA

>Dog_T2R3--Intact

ATGTCAGGGCTGGGGAAATCCGTGTTCCTGGTTCTGTCTGTCACTCAGTTCATTCTGGGGATGCTGGGGAATGGTTTCATAGTGTTGGTCAATGGCAGCAGCTGGTTCAAGAACAAGACAGTCTCTTTGTCTGACGTTATCATCACTAACCTGGCTCTCTCCAGGATTGTTCTGCTGTGGATTCTCTTGGTTGATGGTGTTTTAATGGTCTTCTTTTCCAAAGTACATGATGAAGGGACAGTAATGGAAATTATTGATATTTTCTGGACATTTACGAACCACCTGAGCATTTGGCTTGCCACCTGTCTCAGTGTCCTCTACTGCCTGAAAATTGCCAGTTTCTCCCATCCGACGTTCCTCTGGCTCAAGTGGAGAGTTTCCAGAGTGGTCGTACAGATGATTTTGGGTGCACTGCTCTTATCGTGTGCCAGTGCCATGTCTCTGGTCCATGAATTTAAGATCTATTCTATTCTCAGTGGAATTGCTGGTACAGGGAATGTGACCGAGCACTTTAGAAAGAAGAGAAATGACTATAAAGTGGCCCATGTTCTTGGGACTCTGTGGAACCTCCCTCCCCTAATTGTTTCTCTGGCCTCCTACTTTCTGCTCATCTTCTCCCTGGGAAGGCACACACAGCAGATGAAGCACAGTGGCACCAGCTCCAGAGATCTGAGCACGGAGGCCCACCAGAGAGCCATCAAAATCATCGTCTCTTTCCTCTTTCTCTTCCTGCTTTACTTTCTTGCCTTTTTAATTACATCATCCAGTTATTTCATACCAGAAACTGAGATGGTTAAGAGAGTTGGAGTAGTTGTTACAATGTTTTACCCTGCCAGCCACTCATTCGTTATCATTCTGGGAAACAATAAGCTGAAGCAGATGTTTACGGAGATGCTGTGCTGTGAGCCTGGTTATCTGAAGCCTGGATTCAAAAGACCTTTTGCCCCATAA

>Dog_T2R62--Intact

ATGTCCTCCTCACCTACATTGATCTTCATGGTCATCTTCTTCCTGGAGTCGTTGGCTGCAATGCTGCAGAATGGCTTCATGGTTACTGTGTTGGGCAGGGAGTGGGTGCGACGCCGGACGCTGCCTGCAGGTGACATGATTGTGGCCTCCCTGGCTGCCTCCTGGTTCTGCCTGCATGGGGTGGCCATCCTGAACAACCTCTTGATCTTCTTTGGTTTTCACTTCGTAAGGGATTATTACAACACCCTCTGGCACTTTGTCAACACTCTCACTCTCTGGCTCACTGCCTGGCTTGCTGTCTTCTACTGTGTGAAGGTCGCCGTCTTCTCTCACCCGGTCTTCTTCTGGCTGAAATGGAGGATTTCTCGGTTAGTGCCCAGGCTGCTGCTGGGCTCCCTGGTCTTAGTTGGCCTGACAGTCATCTCATCAGCCATTGTGACTGGAATTCTGAAACAGATGATTGCCTCCAAGAGTTCCCAAGGAAACAGCACCTGGGCTGAGAGAGTACAGGCCTTCTATAGGTCTTTTCAAATATTTGATGTAATGCTTATGTGGTCAGTTCCATTCCTCATGGCTTGTGTCCATGCTCTGCTGGGGATTCGAAGGTCTGATGACAGGCTTTGGAAAGCTCTGGACAAGGCTGATTTGTCTCAAGTTATCAGTATCTATAATCACTTCTCTCTTTACATTTCCCTCTTTCTTTCCTACTCCTTCTATGCCCTATTAATTCTTTTTGCAGCCCTCTCTGTTCCTGACATCCCCTCTACTGTTTCTAGTCCTCAGGGTGGGTTCCAAACAATCCTGGGCCATGATGATGGCTTCATTGCCTCACAGACCTTTCACACCCTGTATATGAGCAACTATTATAAAGTTCTCCAAGCATCCTTTGGTCCTGACTGCTTTATGGAGACCTGTAATGGTTGTCAGTTGTCTCTGTGGTCAACTTGA

>Dog_T2R41--Intact

ATGCAGCCCGCCGTGTCCGCCTTCTTCATGCTGCTCTTTGTCCTGCTGTGTGTCCTGGGGATCCTGGCCAACGGCTTCATCGTGCTGGTGCTGAGCAGGGAGAGGATGCGGCGGGGGAGGCTGCTCCCCTCCGACGTGATCCTCCTTAGCCTGGGCGCCTCCCGCTTCTGCCTGCAGTGCATTGGGATGATGAACAACTTTTACTACTACCTCCACCTGGAGGAGTACAGCACGGGCCCGGCTCGGCAATTCTTTGGCCTCCACTGGGACTTCCTGAACTCGGCCACCTTCTGGTTCGGCTCTTGGCTCAGCGTCCTCTTCTGCATGAAGATCGCCAGCTTCACCCACCCCACCTTCCTCTGGCTGAGGTGGCGGCTCCCAGGCTCGGTGCCCTGGCTCCTCGGGGCTTCCCTCCTGATCTCCTTCCTCGTCACCCTGCTCTTCTTTTGGGGAAACCATGCCGTGTATCAAGGATTCCTAATCAGAAAATACCCCGGGAACATGACCTTCCAGCAGTGGAGCAGGAGGCTGGAAATTCACTATTTCTTGCCCCTGAAATTCATCACCTTGTCAGTGCCTTGCTCTGTCTTCCTGGTGTCCATCGCACTGTTGATTAATTCCCTGAGGCGACACAGGGGGAGGATGCGGCGCAGTGGCCACGGCCTGCAGGACCCCAGCAGCCAGGCTCACACCAGGGCTCTGAAGTCCCTCGTCTCCTTCCTCATTCTGTATGCTCTGTCCTTTGCGTCCCTGGTCATCGATGCTGCGGGTTTCTTCTGCTCGCAGAGTGACTGGTACTGGCCCTGGCAGATTTTAATCTACCTGTGCACCTCTGTCCATCCCTATATCCTCATCCTCAGCAACCTCCGGCTCCGAGGGGGGTGCAGGCAGCTACTTCTGTTGGTCAGGGGCTCCCAGCTGGCCTAG

>Dog_T2R5--Intact

ATGCTGACTGCTGCCCTACCACTGCTGATGGTGGTGGCAGTGGTTGAATTTCTCATTGGCTTGGTGGGAAATGGAGTCCTTATGGTCTGGAGTTTTGGTGAATGGGTCAGAAAATTCAACGGGTCCTCATACAACCTCATTGTCCTGGGCCTGGCTGTCTGCCGATTTCTCCTGCAGTGTCTGATTATGATGGACTTAAGCCTGTTTCCATTTTTCCAGAGTAGCCGTTGGCTTCACTATCTCAGTATCTTCTGGATCCTGGTAAGCCAGGCCAGCCTGTGGTTTGCCACTTTCCTCAGCGTCTTCTACTGCAGGAAGATCATGACCCTTGAACATCCTGTCTGCTTGTGGCTGAAGCAGAGGGCCTATTGCCTGAGTCTCTGGTGCCTTCTGGTGTACCTCATGATCAGTTTGTTACTTGTAGCACACATTGGCTTAAAGCCCTATAATCCTTCTCAAGGCAACAGCAGCATTCTGTACCCCCTTAAAAGCTGGCACTACCTGTATATAGTAAAGCTCAACGCAGGAAGTGGATTGCCTCTCATGGTGTTTCTTGTTTCTTCTGGGATGCTGATTGTCTCTTTGTATAGACACCACAAGAAGATGGAGGTACATACAGCTGGTAGGAGAGATGCTCAGGCCAAGGCTCACATCACTGTACTGAAGTCCTTGGGCTGCTTCCTTATCCTTCATGTGATTTATATCCTGGCCAGCCCCTTTTCCATTACCTCCAAGTCTTCTGCTGATCTCCTCGTTGTCTTCATCTCTGAGACAGTCATGGCTGCCTATCCTTCTCTTCATTCTGTCATTCTGATCCTGGGGAATCCCAGGATGAAGCAGACTTGTCAGAGAATTCTGTGGAAGACAGTGTGTGCTTGGAAATCCTAG

>Dog_T2R39--Intact

ATGATGGAAACCTGCAATCCCCCAGAAAATGAATTGTCACCATTTGGCATCCTCTCGATTTTAACAATTACAGGCACTGAATGCATCGTTGGTATCATTGCAAATGGGTTCATCATGGCTATAAATGCGGCTGAATGGATTAAAAATAAGACAGTTTCCACAAGTGGCAGAGTCCTGTTTTTCTTGAGTGCATCCAGAATAGCTCTCCAAAGCTTCACAATGCTAGAAATTACCTTCAGTTCAACATCCCCACGTTTTTATAATGAAGATGTTATGTATGACACATTCAAAGTAAGTTTCATGTTCTTAAATCATTGTAGCCTCTGGTTTGCTGCTTGGCTCAGTTTCTTCTACTTCGTGAAGATTGCTGATTTCTCCCACCCCCTTTTTCTCAAGCTGAAGTGGAGAATTTCCAGACTGATGCCCTGGCTTCTGTGGCTTTCAGTGCTTATTTCCTTGGGCTACAGTATGCTCCTCTCCAATGACATCTACACTGTGTATTGTAACAATTCTTCTATCCCCTCTTCCAACTCCACTAAGAAAAAATACTTCACTAAGACCAATGTGGTCAACCTGGTTCTTCTCTATAACCTGGGGATCTTCATTCCTCTAATCATGTTCATCCTTTCGGCCACCCTGCTGATCATCTCTCTCAAGAGACATACACTACACATGGAAAGCAATGCCACTGGCTGCAGGGACCCCAGCATGGAGGCTCACATAGGGGCCATCAGAGCGACCAGCTACTTTCTCATTCTCTATATTTTCAATTCAGTTGCTCTATTTCTCTATATGTCCAACATCTTTGATATCAACAGCTCCTGGAATATTTTGTGCAAATTCATCATGGCTGCCTACCCTGCTGGTCACTCCATTCTGCTGATTCAGGACAACCCTGGGTTGAGAAGAGCCTGGAAGCGGCTTCAGCCTCAAGTTCATTTTTACCTAAAAGAGCAGACTCCATGA

>Dog_T2R55--Intact

ATGTTAGCTGGATTGGATATAATCTTTCTTACACTGTCAACAGCAGAATTCATAATTGGAATGTTGGGGAATGCGTTCATTGGACTGGTAAACTGCTCTGAATGGGTCAAGAACCGGAAAATCTCTTTAGCTGACTTCATTCTCATCTGCTTGGCTATCTCCAGAATCGCTCAGCTGTTGGTGTCATGGTTTGAATCATTTATGATGGGACTATCTCCACTTTTCTTTTCCACTTATAAACTGGCAAAATCTATTACTTTGCTTTGGAGAATAACTCATCATTTGGCTACGTGGTTTAGTACCTGCCTAAGCATTTTCTACCTCCTTAAGATAGCTCAGTTCTCTCATTCCCTTTTCCTCTGGCTGAGGTGGAGAATGAACAGAGTGGTTCTTGCAATTCTTGTATTTTCTTTGTTCTTTCTACTGTTTGACTTTCTAATGCTAGAAACATTCAATGATCTCTTCTCGAATGTCGATGCAATGGATGAAAGTAATCTGACTTTATATATATATGAAAGTAAAACTTTTTATGTTAAAACCTTGATTCTTCTTAGTTTTTCCTATATCATTCCTATTATTCTGTCCCTGACCTCATTGCTCCTTTTATTTCTGTCCTTGGTAAAACACATCAGAAATTTGCAGCTCAACTCCATGGGCTCCAGGGATTCCAGCACACAGGCCCATAAAAAAGCCATTAAAATGGTGATGTCTTTCCTCTTCCTTTTCACAGTTCACTTTTTTTCCATACAATTGTCAAATTGGATGTTTTTTTTATTTTGGAACAAGAAGATCACAAAGTTTATCATGTTGGCCGTTTATGTCTTTCCTTCAAGCCACTCACTAATTTTGATTCTGGGAAACAGCAAGCTGAGACAGACAGCCTTGAAGGTACTGTGGCATCTTAAAAGCTCCCTGAAAAGAGAAAAACCAAATTCATCTTTACCGATAGACTTTCCAGAATCTTTCCAATGA

>Dog_T2R67--Intact

ATGCCATCTAGAATTGAAAATGCTTTTCTGGTAGCAGCAGCAGGAGAACTCATAACTGGAATGTTGGGGAACGGTTTCATTGTACTAGTTAACTGCATTGACTTGGTGAAGAATCTAAAGCTCTCTACTGCTGACTGCATCCTCACCAGCCTGGCTCTTTCCAGAATCATTCTTCTTTGTATAATACTACTTGATTCACTTTTAATGGTGTTTTGGCAACATCTTTATGCCATTGATAAGCTAGCAAAATTCATTAGTGTTTTTTGGACACTAAGCAATCACCTAACTACCTGGATTGTTACCTGTCTAAATGTTTTCTACTTCTTTAAAATAGCCAATTTTTCCCACCCCTGTTTCACCTGGCTGAGGTGGAGAATTAGCAGAGTGCTACTTGTGCTTCCACTGGGGTCTTTATTCTTACTGTTTTTCAACTTTGAATTATTAGATACATTTACGAATTTCTGGGTTAATCTCTATCAAAGACATGAAAGAAACTCAATTTGGTCCCTAGATGTAAGTAAAACTCTGTATCTTAACAGCTTGATTGTTTTCAGTTTCATCTACTTAATCCCCTTTCTTCTGTCCCTGGCCTCTTTGCTCCTTTTATTTCTTTCCTTAATGAGACATATCAGGAATGTGCAACGGAACTCCAGCTCTAGGGACTTCAGAACAGAGGCCCATAAAAGGGCCATGAAAATGGTGATGTCTTCTCTTTTTCTTTCCATGGTTAATTTTACTTCCATCCTATTAACAGGATGGTTTTCCCTTTTACTGCAGAATCATCAGGCCAATTTGGCTGTCCTGTTATTATCGACTCTTGTACCCTCAGGCCACTCATTTATTCTAATTTTGGGAAACAACAAGTTGAGACAAGCTGCGTTAGGTCTACTGTGGCATCTTAATTGCCACCTGAAAATGGTGAAGCCTTTCGCTTCCTAG

>Dog_T2R10--Intact

ATGCTAAGCATACTGGAAGGCCTCCTCATTTTTATAGCTGTTAGTGAATCAATACTGGGAGTTTTAGGGAATGGATTTATTGGACTTGTCAATTGTATTGACTGTGTGAAGAACAAAAAGTTTTCTATGGTTGGCTTTATTCTCACTGGCTTAGCTACTTCCAGAATTTGTCTGATATTGATAATAATTACAGATGGATTTATAAAGATATTCTCTCCAGATATGTATTCCTCTGGTAACTTAATTGATTATATTAGTTACCTATGGGTAATTATCAATCAATCAAGTATCTGGTTTGCCACCAGCCTCAGCATCTTCTATTTCCTGAAGATAGCAAATTTTTCCCACCACATTTTTCTCTGGCTGAAGGGTAGAATCAATAGCGTTCTTCCCCTTCTGATGGGATCCTTGTTTATTTCATGGTTATTTACTTTTCCACAAATTGTGAAGATTATTAATGATAATAGAATGAAGAGTAGAAATACAACCTGGCAGCTCAACATGCAGAAAAGTGAATTCTTTACTAAGCAGATTTTACTCAACCTAGGAGTCATTCTTCTCTTTACTCTATGCCTGATTACATGTTTCTTGCTAATCGTTTCCCTTTGGAGACACAACAGGCACATGCAATTGAATGTCACTGGACTCCGAGACCCCAGTACAGAAGCACATGTGAAAGCAATGAAAATTTTGGTATCTTTTATCATCCTCTTTATCTTGTATTTTATAGGCATTGCCATAGAAATATCATGTTTCATTCTGCCAGAAAACAAACTGCTGTTTATTTTTGGTATGATGACCACAGCCATCTATCCCTGGGGTCATTCATTTATCCTAATTCTAGGAAACAGCAAGCTAAAGCAAGCTTCTTTGAAGACCCTGCAGCAACTCAAGTGCGAGGCAAGGAGACTGCTCACAGCTGCACAGATCCATGTGGGGGGAAATGGATGTTCCAGGAGAATAATCTAG

>Dog_T2R7--Intact

ATGCCGGATAAAGTGGAGAGCATCTTAATGCTCGTAGCAGCTGGAGAATTTTCAATGGGGATTTTAGGGAATACATTCATTGGATTGGTAAACTGCATAGGCTGGATCAAGAAGAGGAAGATTGCCTCCATTGATTTAATCCTCACAAGTCTGGCCATATCCAGAATTTGTCTATTATGTATAATACTATTAGATTGTTTTATATTGGTGCTGTATCCAGATGTCTATGCTACCGGTAAACAAATGAGAATAATTGACTTCTTCTGGACACTAACCAACCATTTAAGTGTCTGGTTTGCCACCTGTCTCAGCATTTTCTATTTCCTCAAGATTGCGAATTTCTTCCATCCCCTTTTCCTCTGGATGAAGTGGAGAATTGACAGTGCGATTCCTAGGATCCTGCTGGGATGCTTGGCCCTTTCTGTGTTTATTAGCCTTGTTGTCACTGAGAATTTGAATGATGATTTCAGATGTTGTGTTAGGACAAAGAAGAAAACAAACTTAACTGTGAGATGCAGAGTAAAGAAAGCTAAATATTCTTCCATCAAGATTTGCCTCAACCTGTTAACGCTATTCCCCTTTTCTGTGTCCCTGATCTCATTTCTCCTCTTGATCCTCTCCCTCTGGAGACATACCAGGCAGATGAAGTTCAATGCCACAGGGTGTAGAGACTTCAGCATAGAAGCCCACATGGGAGCCATGAAAGCTGTCATCTCCTTTCTCCTCCTTTTCATCGCCTACTATTTGGCCTTTCTTGTAGCCACCTCTAGCTACTTTATGCCAGAGACTGAATTAGCTGTGATCATTGGTGAGTTGATAGCTCTAATCTATCCCTCGAGCCATTCGTTTATCCTAATTCTGGGGAGCAATAAATTAAGACAGGCATCTCTAAGGGTACTATGGAAAGTAAAATATGTCTTAAAAAGAAGAAACTTCTAA

>Dog_T2R43--Intact

ATGCTACCTTTACTACAGAGCATTTTTTCCATCCTAGTAATGACAGAATTTGTTCTAGGAAATTTTGCCAATGGCTTCATAGTGCTGGTGAACTACATTGCATGGGTCAAGAGACAAAAGATCTCCTCAGCTGATCAAATTCTCACTGGTCTGGCTGTCTCCAGAATTGGTTTACTCTGGGTAATATTAATAAATTGGTATGCAACTCTGTTGAATCCAGCTTTATATAGCTTAGAAGTAAGGCTTCTTGTTCATATTGCCTGGACAGCGAACAATCATTTTAGCATCTGGCTTGCTACTAGCCTCAGTGTATTTTATTTGTTCAAAATAGCCAATTTCTCTAACCTTATTTTTCTTCGCCTAAAGTGGAGAGTTAAAAGTGTAGTTTTTGTGATGCTGTTGGGGTCTTTGTTCTTTTTGGTTTTTCATGTTGCAGTGGTAAGCATATATGAGCAAATGCAGATGAAGGAATATGAAGGAAACATCACTAGGCAGACCAAACTGAGGGACATTGCACAGCTTATGAATATGACTGTATTCACGCTAATGAACTTTGTACCCTTTGCTATATCCCTAACATCTTTTCTGCTGTTAATCTTTTCCCTGTGGAAACATCTCAAGAAGATGCGATCCGGTGGTAAAAGATATCAAGATTCCAGCACCAAGGTCCACATAAAAGCCATGCAGACTGTGATCTCTTTTCTTTTGTTATTAGTTTGTTACTTCCTGACTTTAATTGCCATAGTTTGGAGTTCTAATAGGCTGCAGAACAAGTTGATCTTCTTGCTTTGCAAGGCTATTGGAATCCTGTATCCTTCAAGCCACTCATTTATCCTGATTTGGGGAAACAAGAAGCTCAGAGAGGACTTTCTGTCATTTCTGTGGCAGCTGAAGGGCTGGCTGAAAAAAGGATATAAGAGGAGCATCATGTGTCTTCTAGGAGAAAACAAATTGATGGAGTCTGTAATATTTTTTTCTTCTACTTCTTTTTCTAATGAGTATGTAATTGAGCAATTTCCAAAGATTTACCTAAAAAAGTCTTTTCTCTGA

>Dog_T2R2--Intact

ATGATCTCCTTTTTGTCAGCTCTTCCTCATGTTATTGTTATGTCAGCAGAATTTATCACAGGGATTACAGTAAATGGATTTCTTATCATCATGAACTGTAAAGAATTGATCAAAAGCAGAAAGCCAACACCAGTGCAACTCCTTTTCATATGTATAGGGATGTCGAGATTTGGTCTGCTCATGGTGTTAATGATACAAAGTTTTTTCTCTGTGTTATTTCCACTCTTTTATAAGGTAAACATTTTTGGTACAGCAATGTTGTTCTTTTGGATGTTTTTTAGCTCTGTCAGTTTCTGGTTTGCCACCTGCCTTTCTGTATTTTACTGCCTCAAGATAGCAGGCTTCACTCAATCCTGTTTTCTTTGGCTGAAATTCAGGATCTCGAAGTTAATGCCTTGGCTACTTCTGGGAAGTTTGCTGGCCTCCATGAGCATTGCAGCTCTGTGTATTGAAGCAGATTACCCTAAAAAGGTGGATGATGATGCCCTCAAGAATGCCACATTGAAGAGGACTGAACCCAAGATAAGGCAAATTAGTGAAATGCTGCTTGTCAACTTGGCATTACTATTTCCTCTAGCCATATTTGTGATGTGCACTTTTATGTTATTCATTTCTCTCTATAAGCACACTCATCGGATGCAAAATGGATCTCATGGTGTTAGAAATGCCAGCACAAAAGCCCATATAAATGCATTAAAAACAGTGATAACATTCTTTTGCTTCTTTATTTCTTATTTTGCTGCCTTCATGGCAAATATGACATTCAGTATTCCTTATGGAAGTCATTGCTTCTTTGTAGTAAAGGACATAATGGCAGCATTTCCCTCTGGTCATTCAATTATAATCCTCCTGAGTAATTCTAAATACCAACAACCTTTCAGGAGACTTCTCTGCTTCAAAAAGAATCAATGA

>Dog_T2R1--Intact

ATGTTAGAGTTTTACCTTATTATCCATTTTCTTTTCACAGTGATGCAATTTCTCATCGGGGTTTTAGCAAATGGCATCATTGTGGTGGTGAATGGCACTGAGTTGATCAAGCAGAGAAAGATGATTCCCTTGGCTCTCCTTCTTTGCTGTCTGGCGATTTCCAGGATTTGTCTACAATTGATCATCTTCTTCATGAATCTGGGTACTCTCTTCTTGATTGAAGTCCCCCTACTTGCTGATAATTTTGTAATTTTCGTGTTTGTAAATGAATTGGGACTTTGGTTCGCCACATGGCTTGGGGTTTACTACTGTGCCAAGATCGCCCCCATAACTCACTCATTCTTTTTCTGGTTGAAGATAAGGATATCCAAGTGGATGCCATGGCTGATCCTCGGGTCCATGATGTATGCATCCGTCCCTTCTGTTTTCTGCAGCAAACAGATATGGGTTTATTCCCAAAACGTTTTGTCCAGCCTTTTTTCCCCAAACGCAACTCAAATCAAAGAAACATCTGCTTTACAGATTGCCTTTCTTATTAGGTTATTATTGCCACTGCTTATCTTTCTCGGTTCCACCCTACTTTTGATATTTTCCCTGGGGAGACACACCTGGCAGATGAGAAACACAGCAACAGGCCCCAGGGACCCTAGCACAGGTGTCCACGTGAGCACGATCCTGTCCGTTCTATCCTTCCTGGTCCTCTGCCTCTCCCACTACATGGCAGCTGCTTTGCTCTCTTTTCAGATCTTTCAGCTCAGAAGCCTCGTCTTTCTGATCTGTCTCTGGGTGTTTGGGTCCTATCCTTCTGGACACTCTATGATCTTAATTTTAGGAAATCCTAAATTGAAACAAAATGCAAAGAAGCTCCTCCTCCACGGGAAGTGCTGCCAGTGA

>Dog_T2R9--Intact

ATGTTGGCTCTGACTCCTGTTATAACTGTGTCCTATGAAGTCAAGAGTGCATTTATGTTCCTTTCAGTACTGGAGCTCGCAGTGGGGATCCTGACCAATGCCTTCATTTTCTTGGTGAATTTTTGGGATGTGGTGAGGAGGCAGCCACTGAGCAACTGCGATCTTATCCTTCTGAGTCTCAGCCTCACTCGACTTTTCCTGCATGGGCTGCTGTTTCTGGATGCCATCCAGCTTACATACTTCCAGCGGATGAAAGACCCACTGAGCCTCAGCTACCAGACCATCATCATGCTCTGGATGATCACAAACCAAGCTGGGCTCTGGCTCACCACCTGTCTCAGTCTTTTCTACTGCTCCAAGATTGTCCGTTTCTCTCATACCCTCCTTCTCTGCTTGGCAAACTGGGTCTCCAGGAAGGCACCCCAGATGCTCCTGGGTGCCATGCTTTTCTCTTCTGCCTGCACTCTCCTCTGTTTGGGGGACTTCTTTAGTAGATCTGGCTTTGCATTCACAACTGTGCTACTCATGAATAATACAGAATTTAATTCACAAATTGTAAAACTCAATTTCTATTATTCCTCCATCTTCTGTACCCTGGGGTCAATCCCTCCTTTCATGTTTTTTCTGGTTTCTTCTGGGGTGCTGATTATCTCTCTGGGAAGGCACATGAGAACAATGAAGGCCAACACCAAAGACTCCGGTGACCCCAGCCTGGAGGCCCATATCAAAGCACTCATATCTCTCATCTCCTTTCTCTGCCTCTATGTGGTGTCATTCTGTGTTGCCCTTATCTCAGTGCCTTTAACCATGGTGTGGCACAACAAGATCGGGGTAATGATCTGTGTAGGGATCCTAGCAGCTTGTCCCTCTATACATGCAGCCATCCTGATCTCAGGCAATGCCAAGCTGAGGAGAGCTGTGGAGACCATTCTACTCTGGGTTCAGAGCAGCCTTAAGGTAAGGGCAGGCCACAGGGCAGATCTCAGGACTCCAGATCTATGTTGA

>Frog_T2R1--Intact

ATGACTACATCAAACCAGTCCTTCCCTGTGAACCCCTATCAGGTGGTCTCCTTGGGCATCATAATGTCCCAGACACTCATTGGAAGTCTGGCAAACGGATTCATGTTTCTAATCAACCTGATGGACTTTGCATCTCATGGAAGTTTGGGATCTGGTGATGCTCTGTTACTTTGCCTTGGTCTCTCACGCTTCGTTTTCCAGTGGCTTTTATTCACAATATACTTGTTGTCTTTTTGGTTTACCGACCTGGCGGTTTTATACATACCCAAAATCATATTCTCCTTCCTGTTTTTTAGCAGCACCAGCTTGTGGTTTGCCACCTTGCTCTGCAGTTTCTACTGTGTTACTTTGAGTAAACTTAACAACTGTTTTTTTTCTATACATAAAAAGGATTTTGACCGCTGGCTTCCTAAATATCTTCTATTTAGTGTTGCCATGTCAGTGATCTTCAGCCTTCCTCCTGTCTACGTTACGTTCCACAATATGGGCAACTGGAGCATTTCGGGGACCTCCTCTGCATTTCTGTTTGTCAGAACCAATAGCGTGGCCTTTGTTTCAGTTTCGTTCCTGGGAAGTGTCCTGCCATTCCTTGTATTCTGTAGGGCAGTTATTGTGCTGGTTGTGTTCCTCTGGAAGCACGTCCTGAAGATGAAGAGGCAGGAGAGGACTGACTATAAGGAACCATCAATGCAGGCTTACTACAGAGCAGCCAAGGCATTGGGCTCATTTTTCCTGTTTTATTCTTTCTACATTGTGGCTTTCAACCTGTACATATCAGGAATTGCCACTTTAAACAACTTAATTGGCTGCTTTTGTACAATGTTGATTGGATCCTATCCATCTGTGCATTCAGTTTTCCTTATTCTTCAGAACACCAAGCTCCAGCAAGCCCTCGCCAGCTTTCAGCAGAAGATCCGATGCTGCTCTTCCCCGGGGAACCAGACTGTTACTGACCCAACCTGA

>Frog_T2R10--Intact

ATGCTGTCTGCCATTCAGATAATAAAAACAATTATTCTGATCATAACCGGATCATGTGGGCTCATCCTAAACTCATGGATTGTAGCTGTGCATCTCAGCCATTGGAAGAAGGGAGTGAGCCTTGGGGACTGTGATCAAATCATTCTCATCAAAGGGGTCACCAACGTTCTCCTCCAGTGCTTAGTAACTTTCAATGGGATACTTATAAACTTTCAGCTGAATGACTATTTTGACAAGGAATTCCTTTATGTGACTAACATTGTCTTCTTCTTCCTGACTTCCCTCTGGAACTGGCTCACTGCCTGGCTCGCTATCTGCTACTGCTTCAGACTCGGCAACATTTCACATCGGGTCTTTATTGGCTTAAAAAAGAGAATCTCCTCTGGAATTACTCAACTCCTGTTGGGAACAGTGGTCGTTTTAGGCATGATTAGTATTCCATATTTTTGGACAACGCACATAAAAGCTAAGCAAAACACAACTTCCACCTCGGTCTTTGAACAAGACATTAAATACCTGTACTTTATGACTGCATTTTGTTGCTGCCTGCCAACTCTTATAACTTCTCTCTGTATGGGACTCAGCTTAAAGTCCCTTTTGAAACATGTCCATAGGATGAAGCAGAATCACTCTCAGTCCTGGAGCGGGAAAATGAAGACCCATGCAAGAGCCTGCATGACAATATTCCTCCTTATGGCTCTGAACTTGTTTTTCTTCTTGATGATTTTTATTAGTGTTATATCAACCAATATTCTAAGTCTTTGGGATATTTTTTTCTGGTCTATAATCATGGCAAGTCCCTCAGGCCAAGCTCTCATTCTGTTGTTTGGAAATTCAAAGTTACGGTCTGATTTGCTAAAGACTTGTTTCTGA

>Frog_T2R11--Intact

ATGCTGTCTGCCATTCAGATAATAAGAACAATTATTCTGATCATAACCGGACCATGTGGGATCGTCCTAAACTCATGTATTGTAGCTGTGCATCTCAGCCATTGGAAGAAGGGAGTGAGCCTTGGGGACTGTGATCAAATCATTCTCATCATGGGGGTCACCAGCGTTCTCCTCCAGTGCTCATTAACTTTCAATGGGATAGCTGACAATTTTGAACTATATGGGCATTTTGACAAGGAAATCGTTTTTGTGAATGACATGTTCTTCTTATTTCTAAACTTTTTCTGGATTTGGCTCACTGCCTGGCTCGCTATCTGCTACTGCTTGAGACTCGTCAACATTTCACATCGGTTCTTTATTGGCTTAAAAAAGAGAATCTCTTCTGGGGTTTCCCTGCTCCTGTTGGGAACAGCGGTGATTTTAGGCGTGATTAATATTCCAATTTTTTGGACACTGAACATCAAAGCAAAGCAAAACATAACCTCCACCTTACCAGTTGACTTCCTTATCTCTGACTCAGACATTAAATACCTGTCCTTCACTGCTGCATTTGGTTGCTGCCTGCCAACTCTTATAACTTCTCTCTGTATGGGACTCAGCTTAATGTCCCTTTTGAAACATGTCCAGAAGATGAAGCAGAATCACTCTCAGTCCTGGAGCGGGAAAATGAAGACCCATGCAAGAGCCTGCATGACAATATTCCTCCTTATGGCTCTCAACTTGTTTTTTTTCTTGACAATTTTTAGTATTATTCTATCAAAATTCGAGATTGAAAATAATTGGAATACTCTCATCTTCTGTATAATCATGGCAAGTCCCTCCGGTCAAGCTCTCATTCTGTTGTTTGGAAACTCAAAGTTACGGTCTGATTTGTTAAAGACTTGTTTCTGA

>Frog_T2R12--Intact

ATGCTGGCAGCGTACACAGTGATTGTCACAGTGATACTGATCGTAACATGGCCATGCGGGACCATCCTCAACTCATCCATTATAGCTGTCTATCTCAGCGACTGGAAGAAGGGAGTTAAATGTGGGGAGTGCGATCAGATCACCCTCAGCATGGGGTGCAACTACCTACTCATACAATGCTTCATAATAATTTTTTGGGCATTCAGGTTCTATGGACTGGACCTTCCATTTGCAGAAAAACTCACTTTTGCTATCAATACTGGGTTCTGGTTTTCTGTTCCTCTCTCATTTTGGCTCACAGCCGATCTCTCCATCTGCTACTGCCTGAGACTGGTCAATCTATCATCTGCACTTTTTAATCAGATAAAAAGAAGACTCTCTCGCATTGTTACCCCTCTCCTTCTGTGGTCAGTGGGAATTTCATTTATTTTTCCAGTTACCCGGATATTTGCCATACAAATTGACCAGAACGAGACTTTTATTAATCTTGAAAATACCTCTAATGTTAATTTGGGTATTGTGATCCCTGCTGTTGCATTTAATGTCTGCCTGCCCTTCATTATTACCTCCATTTGTATTGTACTTTCCCTGATATCGCTCCTGAGGCACATCCGGAGAATGAAGCAGAACACTCAGTTCGGGAGTCCTCAGCTGAAGAACCTTATAAGAGCTTGCAGGACAATGTTCCTACTCATGGCTCTGAATTTTATTTTCTCCCTGATAATTTGCAGTTCTATGCTGATATCAGATAGAATGGGAACTGTATGGGGCAAGGGGGCCTTGTTAGGGAACATGTTGAACCCCTCATGTCAAGCCATTGTTCTGATCTTTGGGAATTCCAAGTTACTCGGTGCCTGGATAAAGACCTTGTTTCCTCAGTGA

>Frog_T2R13--Intact

ATGGATCTGTCCAGTAAAGTAACACTTGTGTATTCTTTCTTTGCACCAATTCTGGCAGTCACTGCAGGAGCCTTCACTAATGCCTATATTACTTTTGTGATTTTGCTGGATTATTTTAAGACCAAAATGATGAGCTCAAGCAATAAAATATTACTTGCCCTGAGCTTGTCCAATGGTTATTTTTCTTTTCTTTTATTTGTTTGCTCAATTATTAGTTTTGTGTGGCCCCACATTGCCACAAACAATTACATCAAGGGCTGTATCCTGGCTCTGCTCATATTTGGCATTTCTTCCACTGCCTGGATAACAACCTGTCTCTGTGTCTTCTACTTTGTGAAGATAATCAACTTCAGCTCTGGGCTTTTCGCTTGGTTTAAGTTGAAGATCGACATCATTGTTCCGTGGCTCATACTGGTTTCAGAAGTGGTGTCCTTGGGCTGTAGTTTCCTTACTTTACTGCCCTCTGTTAATATCCAAGAACCTTCATCAAACTCCTCAATGTTTTACTCACTCAACTCAACATCAGGAGCAACTGGCATCAGCGCTGACTTCATTAAGGTTACGTTCATTGCTGTTTGTGTTCCTTTGCTGATTATGATTGTTACTACGTTCCCTACTATTAGGACACTTTATTTGCACAGTAGGAGAATGAAGAACACAGGGACATCTAGCAGTCTTGCTCCTCATCAAAGTGCAGTGTTTATGATGGCATGGCTCCTATTTTTATATACCGTCTTTTTTGTGGTATTGTTTACAGGTTTTATTCAAAGTTTTACTCCACCAAGCTTTGCATATTGGATGACTTATAACCTGATATATGTAGCAACATTAGTACAATCCGTTGTTCAAATTCTAGGTAACCCTAAACTAAAAGAAGCAATTACAATATGTTGTTGTTTTGTTTGCCACAAAAGCTGGTAA

>Frog_T2R14--Intact

ATGGTTGTGGACGTTACTACCATTTTGCCAGCCCTATTTATAGCTGCATCCATTGTGGGAATGCTCACCAGCATCTTCATCCTGTCTGTAAACGTCCATTCTCTGATAAAAGGCCAACGCTTGAACCCAAGTGACCTCTTGATTATTTCTCTGGCCTTTTCCAACATGGTCTTTGTTGTTTCAAATTTTGCCTTTTCCCTATGTTTATTTTTCACAACTTGTTTGGTCTTCGAAGATCAATTCTACGTGGAAGGTTATCCTGTGGCGTATGTGCTCTTCTCCAATTCCTGGCTCAGCGCCTGCCTATGTTTCTTCTACTTTGTGAAAGTCAGTAACTTCAAACCCAATTACCTGGCACGGCTCAAGTCCAAGATAAACACCTTGGTGCCAAGGCTAATACTGGGCGCTCAAGCGTTCTCCATCTTGAACTCACTATTTTACATGTTGGGGTTTTCAGAAGTGAAGACTGGAAATTCAACACTTTCTCTTGTGACCAACCAGACTTCAGATATTACTGGTTACAGTATAAACATTTATTTCAGTTTATTTTTCTTGCTAATGAATTGCTATATTCCATTCCTCATTATAGTGGTCACCACCAGCTTTATCATTGCCTCGCTCTACAAGCACATTTGTCATATGCAAAAAAACAGGGGGGAGTTTGGTGGCCCCAGCCTGAAAATCCATCACAGAACAGCACTTACAATGACCTTGCTCCTTATTTTTTATTTATTCTTTTATTCTATAATACTGGGAACCAACTTCTTTCTAACTACGGAGTTGATGATCTGGGTTTACGTCACGGCGCGGTGTCTTTTTTCCCCTATTCAGTCCCTCATTCTAATCATGGGCAACTCTAGACTGAAGAAGACCTGTGTGAACGTTTTTAGTAGCTGTAGAAAAATGACCAGTGACGGGGAAATGACGCCTACTATATGCACCTAA

>Frog_T2R15--Intact

ATGGATCCCTCCAGCAAACTCAACATTGTGTATTCTCTTTTTACACCAAATGTGGCCACCATCGCGGGAGCCTTCACTAATGCCTATATTACTTTGGTGATTTTGTTCGATTATTTTCAAACAAAAATAATGAGCGCCAGCAATAAGGTATTAGTTGCCCTGAGCTTATCCAATGTTTACGTTTCTCTTATATTATCTGTTAACTCAATTATCAGTTTTTTTTGGCCCCAAATGTACGCAGAACCATACGTCAAGGGCTACATCCTTGCTCTGCTCATATTTGGAATTGCTTCATGTGCCTGGACTACTACCTGCCTCTGTGTCTTCTACTTTGTGAAGATAATCAACTTCAGCTCCAGGCTTCTTACTAGCTTCAAGATGAGGATCAACATCCTTGTTCCGTGGCTCATACTGTTTTCAGAAGTGGTGTCCTTGGGCTGCAGTTTCCTTACTTTACTGCCTTATGCTAATAGCCTAGAACCTTCATCAAACACCACATTGTTCTACTCAGTCAACTCAACATCAGGAGTAAGTGGCATCTACACTGGATTTATGAAGGTTACCTTTGTTGCTGTTTGTGTTCCTTTGTTGATTATATTTGTTACTACCTTCTCTACCATTGGCTCGCTATATTTGCACAGTAGGAGAATGGAGAGTACAGGGACAACTAACAGTGTGACTCCTCATCGAAGTGCAGTGTGTATGATAGCATGGCTCCTTCTTCTATATACTGTTATTTTTGTGGTGCTGTTTTCATATTTTCTTCATCCTATCAGTCCACCAAGCTTTGGCTATTGGATGAGTTATAACCTGATATATGTATTTACTTTGGTGCAATCTGTCGTTCTAATTCAAGGTAACCCTAAACTGAAGGAAGCATTTGCAAGATATTTCAATGGTTTCTGTTCCATGAAAAAGGCTACCTAG

>Frog_T2R16--Intact

ATGGGTGTATTTATACTCTTGGCAGTAGAGTTTGCAGGGATATTCTCCAGCGTGTTCATAATATTTGTGAATGTCCAGTCCTGGATGAAGGGGCAGAAGATGAGTTCTAGGGATCAGATTATCATTGCCCTCTCCTTCTCAGACATGGTGTTCTCTGCTGTGAATGCTGCCATCGTACTAGGCTCCGCTTTTGTCCCTCAGCTGCTGTCGGTGGATTATATCTATTATGGGCTGTATGATTTGATGACATATTCAATCTGTTCCAACTCCTGGCTCAGTGCCTGTCTAGGCGCATTATTCTTTGTGAAAACCACTAACTTTAATATTGGTTTCCTTGCACAGCTTAAGATGAGGATTGAAATGGTCATGCCTTGGGTAACATCAGGCATTGAAGTATTCTCCTTCATAATTGCACTGGTTCAACCTTCAGCTTCTGCAGAAGTATATGGCGAGAATTCTACACTTCCACTGATAACCAATGAGACTTCCGAAGTTATCGGTTATGAGACAGATGTTCTCAATGATGTCTCATTCTTACTTAAAGCCTGTCTTACTCCATTCATGATCGTAACTGTCACCACTGCTCATATCAACATTTCTCTCTGCAAGCATACTCAACAAAACATGGACTCTGGTGGTCCGACTCTCAAAGTTCACCGAAGGGCTGCTTCTACGATGACATCTCTCCTCATACTCAACACTGTGTTTTATGTTTTACAAATGTGGCTTGGGTTTCTTCAAACCAGTGATCCTCTGTATAGGATTGGCATAACCCTAGTTTGCTCTTTTTCTTTAGTTCAGTCCATCATTCTAATCCTGGGGATTGGCAGGCTGAAAAAGACCTTTATCCAGATAATAACCATGTGTAGAAGAAAGGCTTTGTCTTAG

>Frog_T2R17--Intact

ATGACAGGAGCCTTCACTAATGCCTATATCTCTTTTGTGATTTTGCTTGACTATTTTAAAACACAAACGATGAGCACCAGCAATAAGATATTACTTGCCCTGAGCTTATCCAATGTTTTTCTTTCTCTTATTTTATTCGTTTCCGCGGTGAGTGTTTTCTGGCCGGAGATATATGCAGATTATTATGTCAAGGCCTATATTTTTGCACTGCTCCTGTTTGGCATTTGTTCCTCCGTCTGGAATACCACCTGTCTCTGTGTCTTCTACTTTTTGAAAATAATCAACTTCGGCTCTGGCATTCTTGCTTTCTTCAAGATAAAGATCAACGTCATGGTTCCATGGTTCATATTCTTTTCAGAAGCATTGTCTTTAGGCTGCAGTTTCATTACATTATTGCCTTCTGTCAATAGCCCAGGATCTTCATCAAACACCTCATTGGTCTACTCACTCAACTCCACATCAGTAGATATTGCCAGCATCCCTGGATTCATGAAGGTTACCTTCATTGTTGCTTTTGTCCCTTTGCTGATTATGCTTGTCACTACCTTCTCTACAATGGGGTCACTTTATTTGCACAGTCGGAGAATGGAGAACACAGGGACATCTAGCAGTGTGGCCCCTCATCGAAGTGCTGTGTTTATGATGGCATGGCTCCTTCTTTTATATGCTGTTGTTTTTGTTGCATTGTTTACGTATTTCTTCAAGCTCTTTGATTCAAAAAGCTTTGGATATTGGATAACTTATATGCTGTTATATTCATTCACTTTAGTACAATCCATCGTTCTAATTCTGGGTAACCCTAAACTAAAAGAAGCAATAACTATATGTTGCTGTTTATTTGCCACAAAAGTAAACTAG

>Frog_T2R18--Intact

ATGGATGGAGTAACTAATCACAGCTGGAATAACTACTCAACTGACCGACTTGCGGACTCTGTGGTTGGGAATGATACAACAATATCTCCAGTTCTACTCATAGTTATATCTGTTGTTGGAATAGTCATTAACATGTTCATCTTGTCTGTTAATTTTCATTCCTGGATAAAAGGGCAAAGCTTGAACCCCAGTGACCTCTTAATTGTTACCCTGGCTTTCTCCAACTTGGTCTTTTCTGTCACAGCCGGTGTCTGGATCATATACTTTGGTTTCATCACTTATGGGGACTTCAAAGAATATCTATCATATTCTATCATGGTGTATGTGCTCTTCTGCAATTCCTGGCTCAGCACCTGCCTGTGTTTCTACTACTTTGTGAAAGTCAGTAACTTTAAACCTGGTTACCTGGCACGGCTCAAGTCCAAGATAAACACCCTGGTGCCAAGGCTAATATTGGGCGCTCAAGTGTTCTCCATCTTGAACTCACTATTTTACATGTTGACATTTTTTAAAGTCAACAATGACAACTCAACGCTTCTTTTTTTGACAAACAAGGCTTCAAGTACTACTAATAACAGGATAGACGTTTTTTACAATGCATTCTTCTTGTTGGTCAATTGTTTGATTCCTTTCTTCATTATAGTGGTCACCACCAGCCTTATCATTGCCTCGCTCTACAAGCACACTCGTCGTATGCAACGGAACGTAGGAGAGTTTGGTGGCCCCAGTCTGCATATTCATCACAGAGCAGCACGTACAATGATATCTTTTCTTATTATTTATCTTTCCTTTTACGTTTTATCACTGGGGAATAGCATTTTTCTAAATAAGCACTTGTTAAACTGGGTTAACTACATGTTGGGTTGTGCTTTCTCCCCGACTCAGTCCATCGTTCTAATCATGGGGAACTCCAGGCTCAGGCAGACCTGTAGGAACATACTTAAGAGTTGTATGAAAATATTAAGCAGAGAGACAACTATATCCACTGTGGGCACCTAA

>Frog_T2R19--Intact

ATGCTCACTAACGTGTTCATCTTGTCTGTTAATTTTCATTCCTGGATAAAAGGCCAAAGCTTGAACCCAAGTGACCTCTTACTTGTTACCCTGGCTTTCTCCAACTTGGTCCTTCCAGTCACAGCCGGTGTCTGCACCATATACTTTGGTTTCATCAGTTGTGGGGTCATTAATGATTATCAGTTCTTTGTGCAAACTTCTATCATGGTGTATGTGCTCTTGTCCAATTCCTGGCTCAGCGCCTGCCTGTGTTTCTTCTGCTTTGTGAAAGTCACTAACTTTAAACCTGGTTACCTGGCACGGCTCAAGTCCAAGATAAACACCCTGGTGCCAAGGCTAATATTGGGCGCTCAAGTGTTCTCCATCTTGAACTCACTATTTTACATGTTGACATTTTTTAAAGTCAACAATGACAACTCAACGCTTCTTTTTTTGACAAACAAGGCTTCAAGTACTACTAATAACAGGATAGACGTTTTTTACAATGCATTCTTCTTGTTGGTCAATTGTTTGATTCCTTTCTTCATTATAGTGGTCACCACCAGCCTTATCATTGCCTCGCTCTACAAGCACACTCGTCGTATGCAACGGAACGTAGGAGAGTTTGGTGGCCCCAGTCTGCATATTCATCACAGAGCAGCACGTACAATGATATCTTTTCTTATTATTTATCTTTCCTTTTACGTTTTATCACTGGGGAATAGCATTTTTCTAAATAAGCACTTGTTAAACTGGGTTAACTACATGTTGGGTTGTGCTTTCTCCCCGACTCAGTCCATCGTTCTAATCATGGGGAACTCCAGGCTCAGGCAGACCTGTAGGAACATACTTAAGAGTTGTATGAAAATATTAAGCAGAGAGACAACTATATCCACTGTGGGCACCTAA

>Frog_T2R2--Intact

ATGCTGGCAGCGTACACAGTGATTGTCACAGTGATACTGATCGTAACATGGCCATGCGGGACCATCCTCAACTCATCCATTATAGCTGTCTATCTCAGCGACTGGAAGAAGGGAGTTAAATGTGGGGAGTGCGATCAGATCACCCTCAGCATGGGGTGCAACTACCTACTCATACAATGCTTCATAATAATTTTTTGGGCATTCAGGTTCTATGGACTGGACCTTCCATTTGCAGAAAAACTCTCTTTTGCTATCAATACTGGGTTCTGGTTTTCTGTTTCTCTCTCATTTTGGCTCACAGCCGATCTCTCCATCTGCTACTGCCTGAGACTGGTCAATCTATCATCTGCACTTTTTAATCAGATAAAAAGAAGACTCTCTCGCATTGTTACCCCTCTCCTTCTGTGGTCAGTGGGAATTTCATTTATTTTTCCAGTTACCCGGATATTTGCCATACAAATTGACCAGAACGAGACTTTTATTAATCTTGAAAATACCTCTAATGTTAATTTGGGTATTGTGATCCCTGCTGTTGCATTTAATGTCTGCCTGCCCTTCATTATTACCTCCATTTGTATATTTCTTTCTCTGATATCGCTCCTGAGGCACATCCGGAGAATGAAGCAGAACCCTCAGTTCGGGAGTCCTCAGCTGAAGAACCATATAGGAGCTTGCAGGACAATGTTCCTACTCATGGCTCTGAATTTTATTTTCTTCCTGATAATTTGCAGTTCTATGCTGATATCAGATAGAATTGGAACTATATGGGACATAGTGGTCTTGTTAGGGGAAATGTTGAACCCCTCATGTCAAGCCGTTGTTCTGATCTTTGGGAATTCCAAGTTACTCGGTGCCTGGATAGACCTTGTTTCCTCAGTGATGGAACAATGTACCAAAATTATAGTACCATACATTTATTGTGATAAGGAACATTATATAGAGAAGTAA

>Frog_T2R20--Intact

ATGGATCTCCACAACTTTGGCTTAGTGTTCTGGTTTTTTTTACTGATCCTGGAGGTTATTATTGGAACACTCACAAATGCCTTCATCGTAGTGGTGCTTCTGCTCGGATACTTCAAAAAACAGACTATGAACGAAAGTGATAAAATATTAATAGCTCTGAGCATCACCAATATTTGTTCTTCTCTTGTCAGTTCTGCTGCAATTATGATATTGTTTATGTGGCCTTGGATTTACAGCCATAGTAATGCTACTTTCTGTATCTTCAGCCTGACCATTTTTGGCACAATTTCAATGGTTTGGCTCACAGCCTGCCTTTGTGTCTTCTACTTCGTAAAAATACTCAATTTCAGCTCCGGGATCCTCCTTTGGGCTAAGATGAAGATCAGCAACTTCGTTCCATGGTTGATATTCTTCTCAGAATTGGTCTCCTTGTGCTGGACTTTCTTTACCATGTTGCCCTTAGTCACCAAGGAACAATCTTCAGGAAACATCTCATCTCTCCATTCAGTCAACGCAACGTCGGACACAAATACCATCATCTTTGAACTGATATGCACTGCTGTTTCTTTGCCTCTGATGATCATTATAATCACTACTTTCTCTACTACTGGGTCACTTTATTTGCACAGGCGAAGAATGGAGAAGAACTTGGGTGCTTCAAGCAGTCTGAAAGCTCATCAAAGTGTTGTCTGGATGATGATACGCCTCCTGCTTTTATACACACTTGTATTTGTGGTACAAATTCTTCATTTTAGTGGTACCGTTGCCCCATTAAGCTTTGAATATTGTCTCAATTACTTTATCTTATTTTCCATCCCAGTAGCACAATCCGTCCTTCTCATTCAAGGTAACCCTAAACTGAAGGACAAGCTGAAGCAAATAAGCCTTGTTTGCACCACAGCTGATGGGACAAAGTAA

>Frog_T2R21--Intact

ATGGAATTTATGTTCAGCTCTGTACTCACAACCATATATCTGGTTGAAAACATTGCCGGGTTGGTGTGCAGTGTGTTCATCATTTCAGTGAATTGCTATTGCTATATAAGAGGGCAAGGTTTGAACCCTATCGACCAGATTATCGTCTGCCTCACCTTTTACGACGCCATCTTCTCTGGCTCCAATGGGGCTTCCATCCTCTGCTCTGTTCTTCTCCCAAACGCAACCTCTGTGGACAAAATCCTCAGCTGTATCTCGATTTTCTCAGTACTCTCATGCGCTTGGTTCAACGCTTGTCTGTGTTGCTACTTCTTTGTCAAAATCTGTGACCACAAACTTGGCTTCCTTGCCCAGCTCAAGAGGAACATTGACCCCCTGATGCCCAGGATCATTTTGGCCATTCACTTTCTACCCATCGCTAGCACCTTAGTTACCAGTTTGAGTTCTAATGGAGTATACACGGCCAATACTACAGCTCCAGCCTTGCAGAAGTCCTTCTTTACTTATAGTGGGGAAAATGTCTCCATTGCTTTTGCTTTGATGTTTATCAACTGCGGTATTCCCACCCTGATTGTAACAGTCACCACAGCCCATATCATCGTGTCTCTGTGCAAGCACACTCGTCATATGCAGGAGAATGTGGGGGACGCTGGTGGCCCCAACCTGAAGGTTCTCCGAAGAGCTGCTTATACCATGATATGTCTCCTTATATTTTATATTATAGCCTGTGCTGTATTAATAATTGTCTTGCTATCTCAGGACCAAAGGGTACATTACATCAATTATCTGATGGGTTATGCTTTACCCTCTGCAGAGGGCGCCATTTTAATCCTTGGAAACAACAGACTGAGGGGGGCATGTTTACACATACTGCACAAGTACAGGGCAAAAAGTAACCATGGAGGTGAAGGAGTAAGCACTACAGGAGCATCCCAACCTGCTTCGTGA

>Frog_T2R22--Intact

ATGCTCTCAGAACTCCACTTGATTTTCAGCATTGGTCTTGTTCTATCATGGATATGTGGGACCGTCCTAAACTCATCCATTGTAGCCGTGTATCTCAGTGACTGGAAGAAGGGATTGAACCTTGGTGCCTGCAATCAAATCATTCTCACCATGGGTTGCACCAACCTACTCCTGCAGAGCTTCTTAACATTTCATCTGATGTTCATAACTTATCAGTTCTCCGTTCTGCTTGACAAAGAATTACTTTTAGCCGCTGTCTCTTTTGGTTTACACTTTTCAATTTCTCTCTCATTTTGGCTCACTGCCTGGCTCAGCGGCTACTACTGTGTAAAACTGGCCAACTCCTCCAACAGATTCTTCATTCGATTAAAAAGAGAAGTTTCTACTGTGGTTGCCTACTGCCTACTGGGAACAGTGCTTACTCTGTTCATTGCTGAAATGCCCGTTATTTGGACAACGCATATAATAACAGACCAAAATCAAACAAGCATTTATTGTATTATGGATGATAATATTGCACTTGTTTCATTTAACTTCACAATTTCTTGCCTCCTTCCAACTATTATGACGTCCTTTTGTATTGGACTCAGCCTGATCTCCCTTCTGAAACACGTCCATAAGATGAAGCAGAATACTTCTCAGTTTTGGAACCCTCAGCTGAAAAGCCATGTAAAGGCCTGCAGGACAATGCTACTTCTGTTGGCTGTGAACTTGATATTCTCATTGGCTGTTTTTATTTCCTCCCAGCCGACAGATAAGGCTGAAGTTATTGGGGTATATGTACCCTGGTTTATTTTGATGTCATATCCCTCAGCTAAAGCCATAATTCTTCTCTTTGGTCAAAGTTCTTGTTTTCTCAGTAAAAGGGTCATATGA

>Frog_T2R23--Intact

ATGCTTAACATTGTGACCATTGCCATAGATATTTGCCTTCTCATGGTTGCAGTTCCATGGGATTGGTTTATTATTTTTACGATGATCTCTGAGAGAGTGAGAAACAAAAGGCTTGGTAGAAGTGAACAGCTGATCTGCGTCATCAGTGCTTTCAACTGCTTTTATTACCTAATAAAAGTCAGCAGATACCTCAATCTCTTGCTAATCATGCACAGCATCTCCATTTTCAGCAAGAAATATAAAATGGGAGAATTCATTTTTCTGCTTACTATGGTGTCCAGCAAACTCTGGGTTTCCAACTGGCTGTGCGCATACTTTTGTCTGAAAATAGTTCGTGTCAACCACAAGTTTTACATCTGCCTACAAAGACTGTTTCCCAAATTATGTCCGTGGATACTTATTATATCTATATTTGTATCTTTCGTTCTAAGTCTTACTGAAGTATGCCTGAATGATGAAAAGCTTCTTGATTTTCCCTACAATCAAAATGTATCTTCCAATACAGTGTTCCAAAATGTCAATTTTATTGCACAAGTATACATCATGTTCTGCTTAGCTGCTATCCTGATTTTCATTATTTTGGCTTTGACAATCATTGGCTCTCTGTGCAGGCACATGAATCGAATGAAGAACAATTTGAATGGAACCAGAATAAGCAATGTAGAGGCTCATGTTCAGGCATCCAAGACACTTGTCATCCTCCTGTTTACCAATGTTATCTGGTTTAACATTGCAAATTTATTAATTATTGATACCCCAAAAGGTTTAAATAATGTAACTCCTCCTCTCCTCACTCTGAATACTCTCCTCAGCTCTGTAAACCTAATAAGAGGAAATAATACACTTAAAAATAAACTGTCTGATATTTTAATATGCTTATCTTGTTGTAGTCAAGCTTAG

>Frog_T2R24--Intact

ATGTTATTCCCAATTTTCTTTTCTATTTTCATTACCACAATGCTCATTTTAGGTCTGGCAGTCAATGCCTCCATCACCATTTTTATTTTGAAAGCCTGGTCGAGAGACGGAAAACTAAAGCCCAATGATAAAATCCTAACCGCCTTGTGCTTCATCAGGTTCTTCTTGCAATGCAACTTCATTACAGAAATCTTTGGAATGTGGCTGGAACTTATCCCTCTGTCGGAGTACAAACCGCGCTGTATCTTCTACTTCATCCAGCTGTTCATGGACTTCTTCAGCCGCTGGCTCAGCGCTTGGCTGGCCATGCTCTACTACGTGAAGATCACTATCTTTAAAAATCCTTTCTTCCTACAGCTTCAGTCTCTGATTCCCCGAATCACCGGATATGTGATCTTTATATCCGTGTTCATTTCCTTTGTACCGGGACTGATTTATTCCCTGAGTGCTAAACAGGGATTTTGTGAGGATGCCAAAGGCGTCAATGTGACAACAAACACGGATGAGTTACTGGCTTTCCGTATCATTGCCTTTTTCTTTGGACACAGCTTCCCCTTCATGCTAGAGATGCTTTCATCCATATATCTCCTCTGCACCCTGTTTGCCCACATCAGACACACCGAATCCTACATTTCCAACTTCACGGCGCCCAACATGGAAGCCCACTGGACTGTTATTAGGTACATTTTTCTAATGAATCTCTTGTCAATGTGCAATTTCTTTGGCAACTTTTTTCTCTGGTCCTTCATCTCCAGTTTTATTGGAAGAGCCGTTGGATACTTTCTAGCTTTCTCTTATCCTACCTTCCATTCAGTTGTCCTCATATTGTCCAACCCAAAGATGAAAAGAGAAGTAGTCAACATGTTCCATTGTGCAACAAAACTCTGGAGTTTCAGCAAGCAAGAACCAGGGACGGTTACCCAATGA

>Frog_T2R25--Intact

ATGTTATTCCCAATTTTCTTTTCTATTTTCATTACCACAATGCTCATTTTAGGTCTGGCAGTCAATGCCTCCATCACCATTTTTATTTTGAAAGCCTGGTCGAGAGACGGAAAACTAAAGCCCAATGATAAAATCCTAACCGCCTTGTGCTTCATCAGGTTCTTCTTGCAATGCAACTTCATTACGGAAATCTTTGGAATGTGGCTGGAACTTATCCCTCTGTCGGAGTATAAACCGCGCTGTATCTTCTACTTCATCCAGCTGTTCATGGACTTCTTCAGCCGCTGGCTCAGCGCTTGGCTGGCCATGCTCTACTACGTGAAGATCACTATCTTTAAAAATCCTTTCTTCCTACAGCTTCAGTCTCTGATTCCCCGAATCACCGGATATGTGATCTTTATATCCGTGTTCATTTCCTTTGTACCGGGACTGATTTATTCCCTGAGTGCTAAACAGGGATTTTGTGAGGATGCCAAAGGCGTCAATGTGACAACAAACACGGATGAGTTACTGGCTTTCCGTATCATTGCCTTTTTCTTTGGACACAGCTTCCCCTTCATGCTAGAGATGCTTTCATCCATATATCTCCTCTGCACCCTGTTTGCCCACATCAGACACACCGAATCCTACATTTCCAACTTCACGGCGCCCAACATGGAAGCCCACTGGACTGTTATTAGGTACATTTTTCTAATGAATCTCTTGTCAATGTGCAATTTCTTTGGCAACTTTTTTCTCTGGTCCTTCATCTCCAGTTTTATTGGAAGAGCCGTTGGATACTTTCTAGCTTTCTCTTATCCTACCTTCCATTCAGTTGTCCTCATATTGTCCAACCCAAAGATGACAAGAGAAGTAGTCAACATGTTCCATTGTGCAACAAAACTCTGGTGCTTCAGCAAGCAAGAACCCCAGACAGTTACCCAATGA

>Frog_T2R26--Intact

ATGTTCTCCCCATTTGTGACTGCAATGAAAATACTGTGTCTGGCTGAGTTTGTGATGGGAATCCTTCTCAATGCTTTCATCGTGGTAGCCAATGCCGTTTCATGGATGGAGAGGAAGCCCCTTGACTCTATTGACCTGATTCTGACCAGCTTGGGGCTTTCTAGACTTGCTCTTCTGATAACATGGCTGCTTTATGTCCTATCAGAAGATAGGTATGAGCACATAGAGATACTGAATGTAATGTCTTCTTTTTTTGGCTTCTGCAGCCTCTGGTTTGGGACGGTTCTGTGCACGTTCTACTGCGTGAAGATCCCCAACTACAACCATCGCTTCTTTCTCTATGTGAAACTGAGGATCTCCAAGATGATCCCGTGGCTGCTATTAGTATCGGTGACAAGCTCCTTCATCAGCTGCCTGCCCATTGGATGGAGTATGGACAGTTCTATGTTCCACTACAATTCCACAAATGGCACAAACATGGAGACCACCTTGTACCTCCAATTCTTCATTTATGTAGCTGGAAATTCAGTGCCGTTCTTCATGTTCTGCGTTGCCATCACTCTTCTAATCAGGTCCCTGTGGAACCACACAAGGCAAATGGCTGCGGGGGAGGTTGGATTTGGGACCCCCCAGCTTCAGGCTCACTACAGCGCAATTAGATGCATGATGTCTTTCATGGTACTGTATATTATCTTCTCATCCTCTGTGTTCTTGGCTCTTCCAATGGTGTTAATGAATGATACGTTGCTATGGTTGTATCTCTTTATTGCTGGTTTGTACCCGAGCCTGCACTCTCACATTCTCATACTGAGCAACAGAAAGCTCCGACGGGCCCTGTGCTCTCTCCTTTCTTATACAAGTACATTTATCCCCCAGGAGAGGAACATATCCCCTTGTCAGTAA

>Frog_T2R27--Intact

ATGTCATTTCCGATTCCCGTGGCTGTTTGTTCCCTGGTTCCGACCGTGACAGGGTTGGTGGTCAATGCCTTTATCACCCTTGTAAACATCCGGAACTATTTAAGGGAACAGGGGCTGAAACCATGTGATAAGATTCTAATGGCTTTGTGCTTGACCAGGTTTCTCCTGCAATGGACTTTCCTTCTGGATATCATTGGGATTTTGCTCCAGTTGATTCCTTTCTCCACATTCGCTATCTACTGTATCTTTTATGTGGTGGAACAGTTTTTGGACTACTTCAGCCGGTGGCTAGCGATGTGGCTCTCCATTCTGTACTACGTCAACATCACAATCTTCAAAAATGTCTTAGTCCTCTATTTGAAGTCAAGGATCCCCCACGTCACTAGTTACATGATCTTCGTCTCGGCGTTCCTTTCCTTCGGCCCGGGACTGATTTATTCTTTGACCAGCAGTGAGGTCTCTTGCCTGAGGGTCTCCAACCAAAGTCTTTCCAAGAATGCAACTTATGAAACCAAAGAATTCTTGTCCATCTCCTTTGTCTTCGGACAGTGTTTGCCTTCTGTGATAGAGATGGTTTCATCCATTTATCTGCTCTGGACTTTATTTGCTCATGTCAACTACACCAAGGCCAATGTTTCCAGCTTCACGGCACCCAACATGGACGCCCACTGGTCTGCAATCCGATGTATTCTTCTACTGTATTTCATGTCTGCCTGTAATTTCATTGGGAACCTCATTTTATATTTCTTTTCTAATGATTCTTTTGGAAGTTCCCTTAGTTACATAATAACCTTTGCTTACCCCACTCTGCATTCTGTCGTTTTTGTACTGAACCATTCGAAGCTGAAGAAAGAACTGGCAAAAATATTGTACTGTGAGAAATCTGTGGTTGGTTTCAGAGCAGAGACTGTGGTAAGATCATCTCTAGGCCAGGCCTAA

>Frog_T2R28--Intact

ATGTATTGGGCTGTATGTTGTGGGGTTATAATGGCGGCCGGACTGGCGGTGAATGGATTTATCACTCTAGTTAATATCACCGAGTGGTTGAGGGAAGGAAGGTTGAAGCTCTGTGATAAGGTCCTAACGATATTGTGTTTGACCAGATTTTTCTTACTATGGATTTTTTTCCTGGAAATGATTGGGGTTCTACTGCAACTCATCCCATTCTCCGCATTTGGAATATATTGTATTTTTTATGTGTTTGAGCTTTCCCTGGACTATTTCAGCCGCTGGGTGGCCATGTGGCTCAGCGTCCTGTATTTTGTGATGATAACCATCTCCAAACAACCTTTCATGCTGAGTCTCAGGTCCAAGATCCCCTCCATCACGAGGTCCATGCTGTGGGTGTCCATCTTGCTTTCCTTCTTCCCAGCCTTGACTTACTCCCTGACTGAGAAAGACTTTCCTTGTGTACAAAACCCCGACACAAATATCTCAGCCAATGGGACGTCTGGATTTCCCAGACCTTTCCTATTGACTTCGTTCTTCTTTGGACATTGTGTCCCTTATATGATGGAAATGGTCTCATCCGTGTATCTTCTCTGTACGTTGTTTGTTCACGTTAAACACACAGAAACCAAGGTTTCCAGCTTTTCCACCCCCAACATGGCCGCCCATTGGTCGGTTATACAATACATTTTTGTACTGAACTTTATGTCTATGTGTAATTTTGTTGCTAATATTTTTATCTGGTTCTGGATCTCGGATAGAGATACCCTCGCCGTCTTATATAATAGTTTATTCATATCCAGTCTTGCATTCAATCGTTTTCATCCAGAACAATTTAAAGCTGAAAAGAGAAATTCAAAAAATACTCCATTGTTCAAAAACATTGTGTTGCCCCAAGAAAGAACCATCTGA

>Frog_T2R29--Intact

ATGGAACCAGGATACAAAAATTTGACAATGGTATCAATGAATTTCAATCAGTCATTCATTGACTATAAGACTGTCACCTCTACGATCGTATCATTTGAGGTGCTCATTGGAAGTCTGGCTAATGGATTTATGGTTACGGCAAGTCTGATAGACTGTGCTTCTCGCAGGAAGTTGGGCTCCTGTGAATCCATATTAATCTGCCTTGGCCTTTCACGCTTTGCCTTCCTGTGGACCCTGTTTTTTATGTACTTGATGTCGGTTTATCTTACTAGCCTGAACGGTAAATATACGGATGCTGTCATGTACATCTTCCTGTGCTTTAGCAATAGCAGTTTGTGGTATGCCACATGGCTCTGCATGTTCTACTGCGTCCGCATCGTCAACATCAGCAACTGCGTCTTTGTTGTTTTCAAAAAGAATTTCGACCGCTGCCTTCCCGTCTTGCTTCTTGGAAGTTTAGCCATATCAGTAGCCTTCAGTCTTCTTTTACTCAGCAATAGTTTGACCAATGTGGACTTCATCAGCTCATCAAACTCACAACCATTAGGGGTTCCCAGCAAATCCACCTCCCGCTTTCTGATTACTTCTTTCATAGGGAGCGCTCCGCCATTTCTAGTTTTCTGCATAGCAGCTTGGCTGGTGGTATATTCCCTTACGAAGCACACAAGGAGGATGAAGGAACAAGAACGGACAAGTTTCAAGGAACCATCACTAGAAGCTCATTATGGGGCAGTGAAATCTGTGGGACACTTCTTCCTGTTTTATGCAATTTACATCATCTCATTTAACCTGTACCAAGCCGGAATAGCCAGCCCTAACTCCATAGAGGGATTGTTTTGCACTGTATTGACCGGATCTTATCCGTCGGTGCATTCGGTGCTGCTTCTTCTTCAGAACAACAAACTGCAACAAACCCTCATCAGTTTTTGGCAAAAAATTAGTTGCTATCATCATGCCAAAGTGAAAAAAGCAGAGTCTACTTCTACATGTAATACTGCATAG

>Frog_T2R3--Intact

ATGCTGACCATTATGGATATTGTTACATTGTCTATTGACTTATTTTCTGTAGTGGTATCATCTCCCGGACACCTGTTTGTTATGCTTGTGAATCTACAGGACCTGATCAATAACGTAAAATTTCAGCTTAACGACCAGCTGATTTTTTGTATCAGTCTCTTCAGTTTTGTTCATGGAATTGTCAAAATATGCAGTGATTTTTTGCTTTGTTTGGCTATGGGATATATCACAGTTGATGGTAAAAGGTATATTTCTGTGCTGCTTTCGACTATATCTTTCTGTATTCTCTGGTATTGTACCTGGCTGTCTTTGTACTTTTGCTTAAAAATTGTCAATTTCAAGCAAGGATTCTATGTATATCTACAGAAGAACTTCCACAGGATGTTTCCATGGATTCATATTATATCAACGATGGGGTCGTTTTTGGCCTCTCTTCCCGTTGGCTGGGATGTCACTGAATACTCATTAAATTCAACAAGTTGTGCAGCCAATCAAAATATGTTTCTTCTTATGTCCAATGGAAAATCGCTCAAGGTTATCATGGTGGTCTCCTTTGTGGCTTTTTTGCTTTTCTTCAGTTCATCTTTATCCATAATCATATCACTTTACAGACACATCAGGAGAATGAAAACCAACATGCAGTCCTTTAGTGACACCAGTTGTGATGTCCATGTTCGTGTAACGGAAACAGTGGCTGCTCTCTTTGCAGCTAATGCTCTGTACTATTTGACATTGCTTCTCTGTGCTTTTCTCCAAAATTCCTATATTTGGCAATGTGTCATTGCTATCATATTGTCCCTTACTCGTGCCCCCAGGCCTATAATCCTAGTAAGGGCTAATAAGAAGCTGGAGGTGCACCTAAGACAATGGTTACATTGCTGTGCATGCATCACATCAAGTTCAGATAATTTTGAGTAA

>Frog_T2R30--Intact

ATGATGTGGGAATCGACAACAAAACTTGTTGCTCAAAGCGTCTTCTTACTTTTTGGACTGGCAGGAAACATGTTTATCCTGGTGATGAGTTTCACTGACTGGAGGAAGACTCATAGCTGGAATCCTTACGCTGTTATCACAATCAGTATTGGGATCTCTAACATCCTGCTCCAAACTACTACATTTCTAAATGAGTTCTTTGTTCTGCTGTTGACAAATGTTTCTGTGCAGGAAACAATGATTAAGTACTTTATTGCCACCCAGATATCTCTTTTTATAAACAGCCTGTTATTTTCCCTCTGCCTCTGCTTTTACTATTGCGTGAAGATCCTCCAAATGAACCAACCGTTCTTCCGGAAGGTGAAGCAAGAGATTGCTAGAATAACCCCCTGGTTTCTCTCAGTATCCATGCTTGTATCTTTGGGCATTGGAATACCCTCCTATTGGGACCTTCACTGGACTCTCACCGGAGCTACAAACTCATCAGCAAGTTGGATACAAGTAAATGTGAAGCTCAGTAGAAGATACCAGTGGGTCTTTATTCTACAGATGCTGATCTCCTCAGGAGCTTTAATAGTCTTCTTCTGCCTGGCTGTCACAATTATCTTCTCCCTTTGCAGACACATGAGGCGAATGAAACTCAACAGTACTGGCTTCAATAAATCCAGCTTAGACGTCCATGTATCGGCTGCTAAAACACTGACTTTGATCTTGCTTCTCCATATCTACTTCTTTGCGGCAGTATGTATCCTCTTCAACTCTCCGCTCACTTGGGGAACCTGGTTTTTTAATCTCAGTTACATAATGGTCGGCAGCTTTCCGTCATTGGATTCTTTCATTCTCATCACTGGGAACTCACAGCTATGGAATTCTCTAAAAAATATATGTCTACTTTGTAGGATTTGCCATAAAATTCCTCAGGACAGGCATACGGTCCTTCATCTTTGCTCTGGTATCCATCCCCCTTTGGAGAGGGTGACAGAAGAACCAGTGTAA

>Frog_T2R31--Intact

ATGGCAGACGGAGAATGGGTTGCCATTGGCTGGGCCCTTCTCGTCCTACTGGGAACCAGTTTTCTGGCTGGGGCAGCAATTAATATATTCATTGTGGCCGTGAATCTCGCTGACTGGTTGAAGAGAAGGCAACTTAGTGATCTGGACAAAGTTCTGACCTGTATTGGGGTCTCCAGACTGGGCCTTCAGATTACGACCACCATGACTGTCTATGGGGTCATTTTCTTTCAGATGGATTTACTGTACGAAGCCAAAACGGTCTCAGAAGCTCTCAGGTCCCTTGAGTTGTTCTTCAACTACTCCAGCACTTGGGTCACAACGTTACTCTCTTTCTTCTACTTGGTGAAAATCGCCAACTTCCACCACCCGGCGTTTGTCCTCCTCAAAGAGAGAATTTCTCAACTCCTGACGGCTCTGGTTATTTCTTGCTTAGTCTTTGCCTTAATTAACACGTTGCTGCTTACGTTGTGGCCTCCTCATATCAATGGGCTTCTTGGGAACTCAACGCACGACCTCCATGAAAATGTGACCGCGTCCCAATCGGACAGAAGAGTTTTTGTTTATTTGTTTGTAGCTGGGAAACTCCTTCCCTTCGCTATATCCGCGGTCTCATTAACCCTCCTGCTTGTGTCGCTCTCGCTCCACATCAGGCAAATGAGAGACAGCAGCCACTCCTCGCCCAATCTGGACAAATACTACACTGCCATCAAATCCATGATATTTTGCTTTCTGATCTATAGTATGCACATTGGCATTGGCTTTGCCGGGCTCCCATATTCTTTTTCTATGAATATCATATGGATACAAATAATGAAAAATCTCTTTCCAACATTACATTCTCTTTTCCTTATAGTAGGGACGGTCAAGCTGAGGCGACAGTTCTGGAGTATAACCCAACGGGCGGCAAACTGCTTCTCAAGGGGGAGCTCCATGGAACTGACAGAGACAATTCCTCCCTAG

>Frog_T2R32--Intact

ATGCTGTCTGCCATTCAGATAATAAAAACAATTATTCTGATCATAACCGGATCATGTGGGCTCATCCTAAACTCATGGATTGTAGCTGTGCATCTCAGCCATTGGAAGAAGGGAGTGAGCCTTGGGGACTGTGATCAAATCATTCTCATCATGGGGGTCACCAACGTTCTCCTACAGTGCTTATTAACTTTCAGTGGGATAAATTATAATTTTCAGCTTAATTACTATCTTAACAAGGAATTCATGTATGTGACTTACATTGTCTTCTTCTTTCTGACTTCCCTCTGGAACTGGCTCACTGCCTGGCTCGCTATCTGCTACTGCTTAAGACTCGGCAACATTTCACATGAGGTCTTTGTTGGCTTAAAAAAGAGAATCTCCTCTGGAATTACTCAACTCCTGTTGGGAACAGTGATCATTGTAGGCATGATTAATATTCCATTGTTTTGGACAACGCACATAAAAGCTAAGCAAAATACAACTTCCACCTTTGTCTTTGAACAAGACATTACATACCTGTACTTTATTACTGGATTTTGTAGCTGCCTGCCAACTCTTATAACTTCTCTCTGTATGGGACTCAGCTTAAAGTCCCTTTTGAAACATGTCCATAGGATGAAGCAGAATCACTCTCAGTCCTGGAGCGGGAAAATGAAGACCCATGCAAGAGCCTGTACGACAATATTCCTCCTTATGGTTCTGAACTTGTTATTCGTCTTGATGATGTTTATTAGTATTATAGGAACCAATATTCAAAATCTTTGGGATATTTTTTTCTGGTCTATAAAAATAGCAAGTCCCTCAGGCCAAGCTCTCATTCTGTTGTTTGGAAATTCAAAGTTACGGTCTGATTTGTTAAAGACTTGTTTCTGA

>Frog_T2R33--Intact

ATGCTGTCAGAACTCCACTTGATTTTCGGCATTGCTCTTGTTCTATCATGGATATGTGGGACCGTCTTAAACTCATCCATTGTAGCCGTGTATCTCAGTGACTGGAAGAAGGGATTAAACCTTGGTGCCTGCAATCAAATCATTCTCACCATGGGTTGCACCAACCTACTCCTGCAGAGCTTCTTAACATTTCATCTGATGTTCATAACTTATCAGTTCTCCGTTCTGCTTGACAAAGAATTACTTTTAGCAGCTGTCTCTTTTGGTTTACACTTTTCAATTTCTCTCTCATTTTGGCTCACTGCCTGGCTCAGCGGCTGCTACTGTGTGCAACTGTTCAACTCCTCCAACAGATTCTTCATTCTATTAAAAAGAGAAGTTTCTACTGTGGTTGCCTACTGCCTACTGGGATCAGTGCTTACTCTGTTCATTGCTGAAATGCCCATTATTTGGACAACGCATATAACAACAGACCAAAATCAAACAAGCATTTATAGTATGATGGATGATAATATTGCACTTGTTTTATTTAACTTCACAATTTCTTGCCTCCTTCCAACTATTATAACGTCCTTTTGTATTGGACTCAGTCTGATGTCCCTTCTGAAACACGTCCATAAGATGAAGCAGAATTCTTCCAAGTTTTGGAACCCTCAGCTGAAAAGCCATGTAAAGGCCTGCAGGACAATGCTACTTCTGTTGGCTGTGAACTTGATATTCTCTCTGACTGTTTTTATTTCCTCCCAGCTGAAAGATAAGGCTGGAGTTAGTGGGATATATGTACCCTGGTTTATTTTGATGTCAAATCCCTCAGGTAAAGCCATAATTCTTCTCTTTGGGAACTCCAGGTTAGCAACTGCCTGGTCAAAGTTCTTGTTTTCTCAGTAA

>Frog_T2R34--Intact

ATGCTCATTGTAACATGGCCATGTGGGACCATCCTGAACTCATCCATTATAGCTGTCTATCTCAGCGACTGGAAGAAGGGAGTGAAACTTGGGGAGTGCGATCAGATCTCTGTCAGTATGGGGTGCACCAACCTGCTCTTGCAGTGCTTCATAACGCTTGGTGTGGCCTTCCTCTCATATGGACTCCACCTGCCGTTTGCCGGACAAGTCTCTGCTGCTATCGGTGCTGTGTTCTGGTTTTCCATTTATCTCTCATTTTGGCTCACAGCCGGTCTCTCCGTCTGCTACTGCCTGAGACTGGTCAATCCGTTACCTAAATACTTTATTCAGTTAAAAAGGAGACTCTCTCGCATTGTTACCCCACTTCTTCTGTGGTCAGTGGCAATTTCATTTATTATGAGCGTTCCAATGTACGGCATAGTAGGCATGGCAACTGACCAGAATACAACAACAAATTATTATTCTATGATTTCCAATGTTATTGATGTTTACAAAATCTTTGGTGGTGCATTTGGCATGGGCTTGCCCACCCTTATTACCTCTGTTTGCATTCTACTTTCCCTGATATCGCTCCTGAGGCACATCCGTAGAATGAAGCAGAAACCTCAGTTCGGGAGTCCTCAGCTGAAGAACCTTATAGGAGCTTGCAGGACAATGTTCCTACTCATGGCTCTGAATTCTTTTTCCTTCCTAATAATTTTCAGTTCAGTACTATCTCCATTTAATGTGGAAACTATATGGGAGACTGTGGTTTTTTCATGTTTCATGTTAAACCCTTCATGTGAAGCCATTGTTCTGATCTCTGGAAATTCCAAGTTGCTCAGGACCTGGTTAAAATCCTTAGCTCCTCAGGGATAG

>Frog_T2R35--Intact

ATGCTGTCGGCACTCCAGGTGATCAGGGCGGTGATGCTCATTGTAACATGGCCATGTGGGACCATACTGAACTCATCCATTATAGCTGTCTATCTCAGCGACTGGAAGAAGGGAGTGAAACTTGGGGAGTGCGATCAGATCAGTCTCAGTATGGGGTGCACCAACCTGCTCTTGCAGTGCTTCGTAACGCTTGGAGTGGCCTTCCTCTCATATGGACTCCACCTGCCGTTTGCAGTACAAGTCTCATTTACCGTTGGCCACATTTACTTGTTTTTTGTTTTTCTCTCATTTTGGCTCACAGCCAGTCTCTCCACCTGCTACTGCCTGAGACTGGTCAATCCGTTACCTAAATGCTTTATTCAGTTAAAAAGGAGACTCTCTCGCATTGTTACCCCACTCCTTGTGTGGTCAGTGGCAATTTCATCTATTACTACGGTTCCAATGAACTGGACATTAGCCATAGCAACTGACCAGAATATAACAACGATGTCTTATAATATTTCTGTTAATGTTGTTCACTTGATTTTTGAAACTGCATTTGGCATCGGCCTGCCCTCCATTATTACCTCTATTTGTATTCTACTTTCCCTGATATCGCTCCTGAGGCACATCCGGAGAATGAAGCAGAACCCTCAGTTCGGGAGTCCTCAGCTGAAGAACCTTATAAGAGCTTGCAGGACAATGTTCCTACTCATGGCTCTGAATTTGCTCTTTTTCCTGGTAATTTTGAGTTCAGCGCTACCCCCATATAGTGGGAATACTTTATGGCAAACTGTGATTTATTCATGCGTCATGTTAACCCCCTCAGGTCAAGCCACTGTTCTGATCTTTGGAAATTCCAAGGTACTCAGTGCCTGGTCAAAAACCTTAGTTCCTCAGGGATAG

>Frog_T2R36--Intact

ATGATGTCCGTGGTCATGACGGTGATACTGATTGTAACATGGCCATGTGGGACTATCCTGAACTCATCCATTATAGCTGTCTATCTCAGCGACTGGAAGAAGGGAGTGAAACTTGGGGAGTGCGATCAGATCAGTCTCAGCTTGGGGTGCACCAACATTCTCTTGCAGTGCTTCACAACATTTGGAGTGGCATCCATATCATATGGACTATGCTTTCCGCTTATCGAAAAAGTCTGTTTAGTGGTCTTCACTGTGCTCTGGTTTCCTGTTTATCTCTCATTTTGGCTCACTGCCGGTCTCTCCATCTGCTACTGCCTGAGACTGGTCAATCTGTCACCTAAATTCTGCATTCCGTTAAAAAGGAGACTCTCTCATATTGTTCCCCCACTACTTGTGTGGTCAGTGGCAATTTCATCTATTATTATGGTTCCAATGAACTGGACTATAGCAACTGACCAGAATACAACAATGATGTATTATAATATTTCTGTTAATGTTGTTTACTTGATCCTTGAAACTGCATTTGGTATCTGCCTGCCCTCCATTGTTACCTCTATTTGTATTGTACTTTCCCTGATATCGCTCCTGAGGCACATCCGGAGAATGAAGCAAAACACTCAGTTCGGGAGTCCTAAGCTGAAGAACCTTATAAGAGCTTGCAGGACAATGTTCCTACTCATGGCTCTGAATTTTCTTTTTTTCCTGATAATTTTTAGTTCAATGGTACCGCCATATAGTGGGGACACAATATGGCAAACTGTGATGTTTTCATGTGTCATGTTAAACCCCTCAGGTCAAGCCGTTGTTCTGATCTTTGGAAATTCCAAGTTACTCAGTGCCTGGTCTATAACCTTAGTTCCTCAGGGATAG

>Frog_T2R37--Intact

ATGTTGTTCTTTATAAATGTGATCAAGATAACATATCTTATAGTAACTTGGCTATGTGGGTCCATCTTAAACTCATCTATTGTAGCTGTGTATCTCAGAGAGTGGAAGAATGGAATGAGCCTTGGTGAGTGTGATCGGACCATTCTGACCATGGGGTGCAACAACCTATTCCTACAGTGCTTTTTAACCATGAATGAAGTCATTACTATTTTTGAACTGTACGGGTTGTTTCTTAAAGAGTTCACTCTAGTTGGTTGTATATTGTACTTCTTTTTGTGCTACGTCTCTATGTGGCTCACTGCCTGGCTTTCCATTTGCTACTACGTGAAACTAGTCAACTTTTCACATCGATTGTTAATTAGGTTTAAAAGAACAATCCCCTCTGCAAGTGCTCCTTTCCTCTTCGGATCAGTGGTGGGTTCATGCTTGATTAATGTTCCCTTCATTTGGACAATGGATACGGAATTTCTGCAAAATACCACCGTATCGGCTGAAAACTTTCTCTTCAAAATGGATCTTAGATTCATGTCTTTCAACGTTGTTATTGGTTCCTGTGTGCCTGTCCTTGTCACCTCTGTTTGTATGGGATTTAGTGTGATGTCCCTTCTGAGACATGTCCAGAGGATGAAGAACAACACTTCTCAGTCCTGGAATCCTCAGTCGAAGAGCCATGTCCGAGCTTGCAGAACAATGTCCCTCCTGATGATTATGAACTTGATTTTCTTTTTGGCAGTTATTACTGTGGCCATGGGTCTTACAAAATTCAATGCCATCATTGCTAGACGTATAATATACTGGTTAGTTGTTATGGCAAGTCCCTCAGCCGAAGCCATCATTCTGATCCTTGGAAATACCAAGCTAAAAATGGCCTTGCCAAAGATCTGTTTTTGA

>Frog_T2R38--Intact

ATGCTGTCAGAATTCCACTTGATTTTCGTCATTGCTCTTGTTCTGTCATGGACATGTGGGACCGTCCTGAACTCATCCATTGTAGCCGTGTATCTCAGTGACTGGAAGAAGGGATTGAACCTCGGTGCCTGCAATGGAATCATTCTCGCCATGGGTTGCACCAACCTACTCCTGCAGTGGTTTTTAACATTCCATCTGATGTCTCTGACGTACCAATTATTCATTATTTTTGCCAAACCGTTACTTTTATCTGTTGTTTCCTTTATAGTAAACTTTTCCGTTTCTCTCTCATTTTGGCTCACTGCCTGGCTCTCCGGCTACTACTGTGTGCGACTGGTCAACTCCTCCAACAGATTTTTCATCCGATTAAAAAGAGGAATGTCCATGGTGGTTACATACTGCCTACTGGGAACAGTGGTTACTCTGTTCATTATTCAAGTGCCCGTTATTTGGAAAGTACACACTAAACTCAACCAGAATCTAACAAATATTTATAGTGCCTTCGACAATTATACTGAACTTGCTTCAGTTAATGCCACATTTGCGTGCTTCCTCCCAACTATTATAACTTCCTTCTGCATTGGACTCAGCCTGATCTCCCTTCTGAAACATATCTGGAGGTTGAAGCAGAATGCTTCTCAGTTCTGGAACCCTCAGCTGAAGAACCACTTTAAAGCCTGCAGGACAATGCTACTTCTTTTGACTGTGAACTTGATATTCTTTCTGGCTATTTCTATTTCCTTTCGGTATAAGCTTGATGATCCTAGGCAATATGTAGCTTGGTTTATTATGTCGTCCAATCCCTCAAGCCAAGCCGTAATTCTTTTGTTTGGCAACTCCAGGTTAGCAACTGCCTGGTCAAAGGTCTTGTTTTCTCATTAA

>Frog_T2R39--Intact

ATGTCACTCACTATGGAAGTGATAAAGATAACAATTATTATAGTGACTTGGCTATGTGGGTCCATCCTAAACTCATCTATTGTAGCTGTGTATCTCAGAGACTGGAAGAATGGAATGAGCCTTGGGGAGTGTGATCGGACCATTCTGAGCATGGGGTGCAACAACCTACTCCTGCAGGGCTGTCTGTCCGTCAGTGAATTCATTAATATTTTTCAACTGTACAGGTTGTTCTTCAAAGAGTTCACTGTGTTGGGTTGCATATTATTCTTATTTTTGAGTTATCTCTCCATGTGGCTCACTGTCTGCCTCTCCATTTGCTACTGTGTGAAGCTGGCCAATATCTCACATGGATTGTTTGTTAGATTAAAAAGGGGAATCTCTACTGCAAGTACTCTCTTCCTATTTGGATCAGTGGTGGCTTCATGCTTGATTAACGTCCCCCTAATTTGGACAATGGATACAGAATTCCTGGAGAATACCACAGCTGATAACGTCATCTACAAACTGGATATTAAATTCATGTCTTTCAACATTGTCTTTGGTTGCTGTGTGCCGATTCTTGTAACTTCTCTTTGTATTGGACTCAGTGTGATGTCCCTCCTAAGACATGTTCAGAGGATGAAGAACAACACTTCTCCGTCCTGGAATCCTCAGTTGAAGAGTCACGAGAGAGCTTGCAGGACAATGTCCTTGGTTTTGATTCTCAACTTGACGTTCTCTGTGACAATCATTACTTTGGCCATGGGTCTAATGAACCACAAGGATGTCCTCGTTAATGATATATTATACTGGTCTGTTGTTTTGACAAATCCCTCGGCGGCTGCCATCGTTCTGATCTTTGGAAACACCAAGCTGAAGACGGCCTTGTCAAAGATCTGTTGTTGA

>Frog_T2R4--Intact

ATGGATTTGGGTTTTCAAGTCATCTTATTGGTTCAGTTCTTCCTGGGGATCTCCGTAAATGGATTCATTCTTGGGGTACTTTTCACCGAGTGGATAACAGGTGGAAGCCTCAATGCCATCGATATGATCCTTATCTCTGTGGGTCTCATCAGGTTCCTCTGGCATTGGGTCATTTCTCTGACCTGCCTATTTCCCGACGCTGGTTTATTGGTATCGGCCATTTGTATTACTCTCAGCACTTTACTGAACTGGAGCAGCTTTGCACTGGTTTCTGTGCTCTGCGTCACCTTCTGTATCAAAATCTCGAACTATAACAACGCCATTTTCATTTACGTCAAGCTCAGGATCTCCGATATACTGAAAAGGCTGATCCTGGGTTCTCTGGGGATCAGTTTGGCTTACAGTGTGATTTCAGTGCCCTGGGATATGGTTGTGCCCCGACAGAATAACTCTGACATTTTGCTGGAGAGGAGCGCGTTCTTCCTAATGGGGACAAGCACGGACTATAGCTTCGCTCTGTTCTGCTTTGCATTTTCCGTATCGTTTATAATGCGATTTGCCTCCATCTCAGCTTTGATTCACTCTCTCAGGAGACACATGCGACAGATACAATGCAGCGGGGAAGCCCTACAAAACCCACACCTGGCGGCCCATATAAGAGTAATAAAAGTAATGCTAATATTTCTGGCCCTTTACATTATGTATTTCACATCGCTAGTCATGGGTTCCCTCGAAAGGTACAGAACTAAGCAGCTGCCGAATTACTATGTCATTGTACTGTGCCTGTACCCCTCTGTCCATTCTGGGGTCCTGATCTACTCCAACAGGAGGCTGAGAAAGGCCTGTGTGGCCATGTGTCTTGGATCCATTCATTGGGCAAAGGCCAGGAAACCAATCGCCCAAACCCAGAACCCCGTGTAA

>Frog_T2R40--Intact

ATGCTGTCAGAATTCGACTTGATTTTTGCCGTCGCTCTTGTCATATCGTGGACATGTGGGACCGTTCTAAACTCATCCATCGTAGCCGTGTATCTCAGCGACTGGAAGAAGGGATTGAACCTTGGTGCCTGCGATCAAATCATTCTCACCATGGGTTGCAACAATCTACTCCTGCAGTGGTTTTTAACATTAAACCTGATCTTCGTGGTTTATGGATTATACAGTCTATTGGCCAAAGTATTACTTGTAGCCGTTGTCTCCTTGGTATTAAACTTTGGAATTGCTTTCTCGTTTTGGCTCACTGCCTGGCTGAGTAGCTACTACTGTGTGAAACTGGTCAACTTCTCCAACAGATTCTTTATACGATTAAAGAGAGAAGCTTCCGGTGTGGTTACATACTGCCTACTGGGTACAGCGGTTATTTTGTTCAGTACTCGCCTGCCCATTATTTGGTCAGTGCAAATAACAACAGACCAAAACCTAACAAGGATTAGTAATGTCTTATATGATAATATTGTACATCTTTCATTCAACACCATATTTATCTACTTTCTTCCTACTATTATAACATCATTCTGTATTGGACTCAGCCTGATGTCCCTTCTGAAACACGTCCGGAAGATGAAGCAGAATACTTCTCAGTTCTGGAACCCTCAGCTGAAGAGCCACATTAAAGCCTGCAGGACAATGTTCCTCCTTTTGACTGTGAACGTGATATTCTTTCTGTCTATTTCTATTTTCTCAATGCTGTTCTCTAAGACTGAAGGTACCGGACAATATGTAACCAGGTTTATTATGTTGTTAAATCCTTCATCTCAAGCCATAATTCTTTTGTTTGGAACTTCCAGGTTAGCAACTGCCTGGTCAAAGGTCTTGTTTTCTAAGTAA

>Frog_T2R41--Intact

ATGTTGTTCTCCATCAAAGTGATCAAGATGACAATTGTTGTAGTAACTTGGCTATGTGGGTCCATCTTAAACTCATCTATTGTAGCTGTGTATCTCAGAGAGTGGAAGAACGGAATGAGTCTTGGGGAGTGTGATCGGACCATTCTGACCATTGGGTGCAACAATTTTTTCCTGCAGTGCTTTTTAACCTCCTATGAAATAATGGCTATTTTTGAGCTCTATGGGTTGTTTCTAAAAGAATTCAAAGTGGCAGGTTTGATATTCTTCTTCTTTTTTAATTATATCACCATGTGGCTCACTGCCTGCCTCTCCATCTGCTACTGCGTAAAACTGGTCAGTTTTTCACATGAACTACTGATAAGATTGAAAAGGGGAATGTCCTCTGCAATCACTCTCTTCCTGCTTGGATCAGTGGTGCTTTCAGGCTTGATTAACGTTCCGTTCATTTGGACAATGGATACGGAATTTCTGGGAAATACGACCTTAACAGCTGATAACGTTATCTACAAACCGGATCTCAAATTCCTGTGTTTCAACATTGTCATCGGTTCCTGTGTGCCCATTCTTGTAACTTCTCTTTCTATTGGACTCAGTGTGATGTCTCTTCGGAGACATGTCCAGCAGATGAAGAACAACACTTCCCAGTCCTGGACTCCGCAGTTGAAGAGTCACGTGAGAGCTTGCAGGACAATGTCCTTACTTTTGATTCTAAACCTGATTTTCTTCATCACATTTATTACCGTGGCCTTGGGTCTAACACAGTTCAAAGGTATACTTATTGGAGACATTTTGTACTGGTCTGTGATTATGTCAGGTCCTTCGATTGAAGCCGTCCTGCTAATCCTTGGAAATACCAAGCTTAAAACAGCTTTTTCAAAGATTTGCTTTTGA

>Frog_T2R42--Intact

ATGCTGACAGTATCGCAAATATCTTGGGCAGTCATCCTTATAATAACATGGCCATGTGGATTCATCCTGAACTCATCGATTGTAGCCGTGTATCTCCGCACCAGGAAGAATGAAATGAAGCGTGGGGAGTGCGACAAAATCCTTCTGGCCATGGCTTGCTCCAATGTTCTCCTGCTGAGCATGATAGCATTTGATATGACCTTTGTTACCTATGGACTCTATATCATGTTTGCCAAAGAATTCTCCTTGGCCATCAGCGTTATACTCTTCTTTTCTATTCACTTCTCATTCTGGCTTACAGCCTGTCTGTCACTCTTCTATTGCCTGAGACTGGTCAACTTCTCACATCAAGTCTTGACTCATTTACAAAGAAGAATGTCGATAGTGGTTCCCCTGTTCCTGCTGGCCTCACTTCTGATTTCATGGCTTATTAATGTTCCATTAATCTGGATGGTCCAAATAGACACCAATCAAAACTCTACGAGTATTTATCAGGATTACATATTTCATTATGATCGACTCTACATGATTTTTAACATTGTTTTTGGTAGCACTTTGCCTTTTGTTGTAACTTCTCTCTGCATTGGACTCTGCCTGATATCTCTTCTGAAGCACGTTCAGAGTATGAGACAGCACATTTCTCAGTACTGGAGTCCACAGTTGAAAAGCCATGTTAAGGCCTTCAGGACAATGTTGCTTCTTTTGATTCTAAACCTGATTTTTTTCACAACGTTTGGTAGCCTTTATCTAGTACAGAATAATCTTGGAGCTGTATTCCAAGCTGTGCTTTGGTCTGCTACCATGTTTATTCCATCAGGGCAAGCCACTATTCTGATCTTTGGAAATTCTAAGTTTGCGAGCACCTGGTCAAAGGCTTTGCCAATGTTTGGGTCATGTGATAGAAATGTTTAA

>Frog_T2R43--Intact

ATGCTGCCTATTTTTTTGCTTGTCTCTATGGCTATTTTGGGAGCTACCACTATATTGGGAATTGCCACAAACCTTGTAATTGTAGTTGTGAATCTTGTTGACAGGGTAAAGGGAAAAAGTCTTAACCCGTCAGACCTCATCCTTGTCACCCTTGGCCTATCAAACATGACTTTCCAGTTCAGCATGACTGCAAATGACTTCTTAAGCATTTTGTGGAGTGACCTTTACTTCTCAGATGCTGTTTATACAACTTTCATTACCCTGCTGCTTTTTCCCATCTTCTCTAGCTTTTGGTTTACTGTATGTCTATGTGTCTACTACTGCTTGCAGATTGTCATATTCACCCATCCTTTTCTTGTGCAGCTAAAGCTTAAAATCTCTCAACTTGTACCATTTCTTCTTGCGACATCTGTCTTTATTTCTGTGGTTATTAGTATTCCTGGAATATGGAGCACCTACAGGGATCCCCCTATTTCAAATATTTCAAACAATCAAAGCTTGGAAATGGAGCTACCCAAATTGAGCTTTACGTACTTGTTTTACAGCAATATTATTGGATGTTCACTTCCACTGGTGTTTGTTGGGATTTCCAATTGTCTAATCCTTAAGTCACTGATAAGCAAGAGCACTATGTTTGAGAAAAACAAGAGTGATGTGTACAGCCCACGAACTGAGGCCAGAGAGCGAGCAGCAAGGACGGTCGGTTGCCTACTGTTACTATACATGGCCTTCTATATCTCTGAGATCTTTATGTTTGTTGATTTTTTTCCACCCGGTAGCCCTGGATTTTGTACCTGCTTAATGGTAATTTATAGCTACCCACCTACACAATCAGTGATTCTAATTTTTGGTAGCCCCAAACTAAAGAAAGCGTTGCTGAACCTTCTCCATTTCTCTAAGATATGTGGCATGGAACAGATAGAGACTCCTAAGATTCTTTCAGTCAGCTTTTAA

>Frog_T2R44--Intact

ATGCTGCCCGTTTTTCTTTTTGCCTCTATGGCTATCTTGGGAGTTACCACTATGATGGGAATTTTTACAAACTCTATAATTGTAGTCGTAAATATTGTTGACAAGGTAAAGGGAAAAAGTTTTAATCCATCAGACCTCATTCTTGTCACACTTGGCCTATCCAATATCACTTTCCAGTTCACAATGACTGCAAATGACTTCTTGATCATTTTGTGGAGTGACTTATATTTCTCATCTGCAATTTATGCTACTTTCAAAGTTTTGCTGTTTTCAACCATTTTTGCCAGTTTTTGGTTTACTGTTTGCCTTTGTGTCTACTACTGCTTGCAGATTGTCATATTTACCCACCCCATTCTTGTGCGTCTCAAACTTGCACTCTCTCGACTTGTACCATATTTTCTTGCAGCTTCTGTCTTTATTTCTGTGGTTATCAGTACTCCTGGAATATGGAGTACAAACAGTGACCCCCCCATTTCAAATTTAACAAGCAATCAGAGTTTGGAAATTGAGGTACCCAAGCTGAGCCTTGTATACCTGTTTTCCAGTAATATTATTGGATGTTCACTTCCACTGGTGCTAGTTGGGATTTCCAATTGTCTAATCCTTAAGTCACTCATAAGGAAGCGCGCCATGCTAGAAAAAAACAAGAGTGATGCCCATAGCCCACGAACTGAGGCCAGAGAGCGAGCAGCAAGGACCGCTGGATGCCTATTATTACTTTACATGACCTTCTATATCTCTGAGATCTTTATGTTTGTTGATTTTTTCCCACCCAGTAGCCCTGGATTTTGTACCTGCTTAATGATTATTTATGGTTACCCACCTACACAGTCGGTCATTCTAATATTTGGCAGCCCCAAACTAAAGAAAGCATTGCTGAACCTTCTCCGTCTTCCAAAGAAATGCAATGAGTCAAAAAAAACAACCAAGATTCTTTTCATTAATTTTTGA

>Frog_T2R45--Intact

ATGCTGCCTGTGTTTGTCCTTGCCTCCGTGGTCATCTTGGGGGCTACCACAATAATGGGAATTGTCACAAATTCTCTAATTGTAGTTGTGAATGTTGCAGACAAGATAAAGGGCAAAAGCTTTAATCCTTCGGACCTCATCCTTGTCACACTCGGCATGTCGAATATCACTTTCCAATTCACCATGACTGTAAATGACTTCTCAATCATTTTGTGGAGTGACTTATATTTCTCAGATGCAGTTTATGGTACTTTTAAAGCTTTGCTCTATTCAACCATTTTTGCCAGCTTTTGGTTTACTGTTTGCCTGTCTGTCTACTACTGCTTGCAGATTGTCATATTCACCCATCCCTTTCTTGTGCGTCTCAAACTTGGAATGTCTCGACTTGTGCCATTTTTCCTGGGGGCTTCGGTGTTCACCTCTTTAGTAATCAGTATTCCAGCATTATGGAGCATCTACAAGGACCCCCAGAATGGCAATTTTTCAAGTAATCAGAGTTTGAAAATTGAGTTACCCAAGCTGAGTGCTGTCTACTTGTTTTCCAGTAATATTATTGGATGTTCACTTCCATTGATGCTTGTTGGGATTTCCAATAGTCTAATCCTTAAATCACTGATAAGCAAAAGCACCATGCTTGAGAAAAACAAGAGTGACGTCTATAGTCCACGAACTGAGGCAAGAGAGCGAGCAGCAAGAACTGTTGGTTGCCTATTGCTACTATACATGGCCTTCTATATCTTTCAGATCTTGATGTTTATTAATTTTTTCCCACCTAGTAGCCCTGGATTTTGTACCTGCTTAATGGCTATTTATGTCTACTCACCTTTGCAGTCAATCATTCTCATTTTTGGCAGCCCCAAACTGAAGAAAACATTGCTGAACCTTCTCGGGTTTTTAAAGAAATGTGGCAATGAACAGAAGGAGACACCCAAGATTCTTTTTATCAAACTTAGAGTTCAAAGAACACTAAACTGCAAAGAAAGCTAA

>Frog_T2R46--Intact

ATGTCATATTTGATTGATGATGTTCTCTCAACTGTTTTTTCAGTAACTGCCGTTCTTTTGGGGCTGGCAGTAAATGGATTTATCCCACTTGTAAACATCAAAGACTGGGTCACAGTGAGGAAGATGAAGCCCTCTGACCGGATCTTAACGGTTCTGTGTTCCACCAGATTTTTATTGCAATTTACGTTTTTTCTGGAACTTACGGGCATTTCTTTTGGCCTGATCCCATTGTCGGCCTATGCTGCCTACTGTATCACTTATGTGGTGGAGCTTTTACTCGATCTATTCAGTCATTGGCTCAGTATGTGGCTTTGTTGCTTGTACTACGTGAAAATCACAACCTCCAAACATCCATTGATCCTCCACCTGAAGTCAAGGATCCCCGGCATCACCAAATATGCTCTCCTCCTCTTTGGGTTCCTTTCCTTCCTAACAGGACTTGTATATTACGTGAGCGGAGATGACATCTCTTGTCTACACGGCATCAGCAAGAATTTGACAGCAAACAGAACCTTTGAAAGCCTCCAAGAACGGTTGATGATCGCGTATTTCTTTGGCCAAGCGTTTCCTTTTATGATGGAGATGATGTCGTCGATGTATCTCCTCTCGTTGTTGGTTTCCCACGTGAAACACACCATGAGCAATTTTTCCAGTTTTAAGGCACCCAGCATGGACGCCCACTGGTCCCTAATTCGATACATTCTTCTACTGTATTTCTTGTCTGCCTGTAATTTAGTTGGTAACTTGCTCCTGTGGCAAGTAACATCTCATTCTATTGGGCGTTCAGTTGGTTACTTTATTATCTTCTCTTATCCTTCCTTCCATTCCATAACTCTTGTCCTATGTAATCCCAAGCTGAAAAGGGAAATGGTAAAAACCTTCATCTGGACAAGAAACGTACTTTGCTGTTTTCGAGGAGACGGAGGATTCCGGACTGAGACTGTTGCCCAGTGA

>Frog_T2R47--Intact

ATGGCTTCCCCGCTGGAGATATTTCTATTAACTCTTATCTGGATTGTGACTGCGGTGGGAATTCTGCTCAATGCTTTCATCGTAGCCATGCCCGTCATTTGGTGGGTGAGGTATAACAAAGTAGAAATGATAGAGTTCCTGCTGGCCAGCGTGGGGATGTCCAGAGTGGTCCTACTGATATTATGGGATGTCGTATACCTTTGGTTCCCCAGCAATTCAGTCCTATTTGCAGTGGTTTCCATGTTCCTCAGCTTCTGGAGCCTCTGGGTTGCTACAATCCTGTGCGTCTTCTACAGCGTGAAGATCAGTAGTTGTCACCATCCGTTCTTCATGTTCCTCAAGCTGAACGTCTCCAAGATGCTGCTGGGCTTGTTCTTGGTGTCCCTGGCAAGTTCTCTCCTATTCAGCCTCCCCTTTAAATGGCTGGTGTACAGTACCTCCATCAACAACGCTACCAACTCTACCAACACTACCAACTCTACCGGACAAGGAGGGACCATCCTGAAGGTCAATAATATCAACCAGTTCTTCCTCATCCTGACCGGATCCTCGGTGCCGCTCCTTATCTTCTGTGTGGCAGTTGCTATTCTGATCCGATCCCTGTGGAGCCACACTAGGAACATGGCCGGGGGCAACGTGGATTTTGGGAACCCCCAGATTCAGGCTCATCTGAGTGCAGTTAAAAGCATGGTCTCTTTCCTGATTCTGTTTACCATTTATTTTGCCCTATTTGTTGTATCATGTTTACCCCCATTGTTGGATGATACTGTGTTGCAATTGGTCTTTAACATTATTTCTAATGCCTACCCGCTCCTACACTCTCTCATCTTGGTAATGTACAGCAGGAAGCTCAGAGAGGCTCTCTACTGGTGTCTCCATTGTACGTGTAGAGTGCCTTCCATGGCGAGAGGTTCAGCTTAG

>Frog_T2R48--Intact

ATGGATTTTACGACAGTGGATATCGTTGAGTTGGTCGTGTCATTTATTCAGTTTCTCTTTGGAATAACGATCAATGGGTTTATCATGGGGACATTTTCCATGCAGTGGAGAAGGAATAAAAGCCTTCAAGCCAGTGATACGGTCCTGATGTTCCTGAGTACAACAAGGTTCTTCTGGCAGTGGGTCCTTTCTCTAATGATTCTCTATAATTACATCACATTTTCTTTGGACTTTCTCCAACTTTTGACTTATATCAGTAATTTTTTAGAGTTGAGCAGCCTGTGGTTGACCTTTACTCTCTGCTTCACTTACTGTGTGAAGATCACAGACCATCCCAACTCCTTGTTCATCTACGTGAAGCTCAGGGTCTCCATGCTTGTGAAATGGATGCTTCTGGCCAGTCTGCTGGCTTCTCTGGCTTTTACTCTCCTGCTCAGGTTTTCATGGGATATTTTTTCTACAGACACCGACCCACGGAACAGCACCGAGACGAATGGAACTCCGAGCTCTGAGCTGCTTGTGTGCTACATGGGAGCTGTGCCACCGTTCCTGATATTCTGCCTGACACTTTTGCTTTTCATTTACTCTTTTTGGATTTTCATCCACAGGTTTAAAAGAAGCAGAACGGGTTTCGAGAGTCCTGTGTTGAGTGATCATTTAAAAGTGCTCAAAAAGATCTTTTTTGCTCTTTTCCTTTATGTTATGTATTTCATCAGCTCTGGTTTGTTCTTGTTTAAACGGCAGCAAATGGCGCATTGGGAACGATGGCTTTTTATTATTTACCAGTTCTACCCAACGGCTTATTTGGGTTGGCTCATCCTCCTCAACTGCAGACTGAAAACGGTTTTCCTGTCTATTTATCAGAGGGCGTTGCGCCTTATAAGAAATAAAAGCCAAATTACCCAGACCCAGAGGGCTGTGTAG

>Frog_T2R49--Intact

ATGCTGGCAGCGTACACAGTGATTGTCACAGTGATACTGATCGTAACATGGTTGTGTGGGACCATCCTCAACTCATCCATTATAGCTGTCTATCTCAGCGACTGGAAGAAGGGAGTTAAATGTGGGGAGTGCGATCAGATCACCCTCAGCATGGGGTGCAACTACCTACTCATGCAATGCTTCATAATATTTTTTTGGGCATTCAGGTTCTATGGACTGGACCTTCCATTTGCAGAAAAACTCTCTTTTGCTATCAGTACTGTGTTCTGGTTTTCTGTTTTTCTTTCATTTTGGCTCACAGCCAGTCTCTCCATCTGCTACTGTCTCAGACTGGTCAATCTATCGTCCGCAGTTTTTAATCAGATAAAAGGAAGACTCTCTCGCATTTTTATCCCTCTTCTTCTGTGGTCAGTGGCAATTTCATTTATTTTTCCAGTTACCCGGATAGTTGACATAAAAATTGACCAGAACGGGACTTTTATTTATCATGAAAATATCTCTAATGTGGGTATTCTGATCTCTGCTGTTGCATTTAATGTCTGCCTGCCCTTCATTATTACCTCCATTTGTATATTTCTTTCCCTGATATCGCTCCTGAGGCACATCTGGAGGATGAAGCAGAACACTCAGTTTGGGAGTCCTCAGCTGAAGAACCTTATAAAAGCTTGCAGGACAATGTTCCTACTCATGGCTCTGAATTTGCTTTTCTTCCTGATAATTTGCAGTTCTATGCTGATATCAGATAGAATGGGAACTGTATGGGGCAAGGTGGTCTTGTTAGGGAACATGTTGAACCCCTCATGTCAAGCCATTGTTCTGATCTTTGGGAATTCCAAGTTACTCGGTGCCTGGATAAAGACCTTGTTTCCTCAGTGA

>Frog_T2R5--Intact

ATGGAATTGGCTGTGACTGGGATGTTGTTTAGCCAACTGGCGCTGGGGGTGGCCATTAATGGCTTCATCGTGGGGACGTACCTGCTGGAATGGAGGAAGAAGCGAAGTCTCCGTATGACCGACGTAGTCCTGGTGTGCTTGGCCGTCAATAGATTCCTGTGGCAGCTGTTCCATACCTTGGGGTCGGTATTTTCATTTGTGCAAGTCGTAGTCACCATCACGTATCAGGTTGTCTATTCCGTCTGTGCCTTTCTAAACTGGAGCGGCCTGTGGCTCGCCTCGGTGCTCAGCGTCATTCACTGTGTGAAGATCTCCAACTACAACAACTGGCTGTTCATCAATCTGAAGCGGAGGATCTGCTGGCTGGTCCAATGGCTCCTTCTGGCCAATCTCCTGACTTCTCTGGCTTTCACTCTTGCACTTGTGTGGTTCACATTCCCTGAGAATCCAGCCAATTCTACAGATGTCTCCTCACAGACCAACGCAACCGTGAACCTTCTCCAGAACATTCCCAGTCCGTTATTGGTTCTGTGCTTGGGATCCCTTCTGCCGTTCATCATATTCTGTGTCGCCGTTTCACTACTGATCAATTCCCTGCGAATACACGTCCAACAGATGAGAAGCCGAGCAACAGGGTTCCAGACGCCACACTTAGAAGCTCATATAAGAGTGATAAAGAGCATGGCCTTTTTCCTTGTCCTCTTTCTTCTTTATTTAGTGGTCACTATTTTGGGGGCAGGTAAGAAGAATGTTGGTAATTGGCTGTACTTCTATCTTGGGATGGCCTTCTACCCATCACTCCATTCTGCTGTGCTGATCTATTCCACTCGCCAGTTAAGAATGGCATGTTTAGCTATTTATTATGGAACCAAAAAGTTAATTTGTGTGCATCATGGAAAGGTCCCACCGGATCTGCAGTAA

>Frog_T2R6--Intact

ATGGCTTCCCCGCTGGAGATATTTCTATTAACTCTTATCTGGATTGTGACTGCGGTGGGAATTCTGCTCAATGCTTTCATCGTAGCCATGCCCGTCATTTGGTGGGCGAGATATAACAAAGTAGAAATGATAGAGTTCCTGCTGGCCAGCGTGGGGATGTCCAGAGTGGTCCTACTGATATTATGGGAGGTCGTAAACCCATTGGTACCCAGCAGTTCTATCCTATTTGGAGTTGTCTCCATGTTCCTCAGCTTCTGGAGCCTCTGGGTTGCTACAATCCTGTGCGTCTTCTACAGCGTGAAGATCAGTAGTTGTCACCATCCGTTCTTCATGTTCCTCAAGCTGAACATCTCCAAGATGCTGCTGGGCTTGTTCTTGGTGTCCCTGGCAAGTTCTCTCCTATTCAGCCTCCCCTTTAAATGGCTGGTGTACAGTACCTCCATCAACAACGCTACCAACTCTACCAACACTACCAACTCTACTGGACAGGGAGGGACCATCTTGGAGGTCAATGTTATCAACCAGTACTTTCTCATCCTGATTGGATCCTCATTGCCACTCTTCATATTCTGTGTGGCGGTTGCTATTCTGATCCGATCCCTGTGGAGGCACACTAGGAACATGGCCGAGGGCAACGTGGATTTTGGGAACCCCCAGATTCAGGCTCATCTGAGTGCAGTTAAAAGCATGGTCTCTTTCTTGGTTCTGTTTACCATTTATTTTGTAATAGTCATTGTTATGTCTTTACCCCCACTGTTGGACCAAAATGTGTTGCAGTTGGTCTTTAACATTATTTGTAGTGCCTACCCACTCCTACACTCTCTCATCTTGGTAATGTACAGCAGGAAGCTCAGAGAGGCTCTCTACTGGTGTCTCCATTGTACGTGTAGAGTCCCTTCCACGGAGAGAGGTTCAGCTTAG

>Frog_T2R7--Intact

ATGGCTTCTCCACTGAAGATATCACTATTAACGCTTACCTGGATTGAGACTGTGGTGGGAATTCTGCTCAATGCTTTCATCGTAGCCATGCCCGTCATTTGGTGGGTGAGGTATAACAAAGTAGAAATGATAGAGTTCCTGCTGGCCAGCGTGGGGATGTCCAGAGTGGTCCTACTGATATTATGGGATGTCATATACCTTTGGTTCCCCAGCAATTCAGTCCTATTTGCAGTGGTTTCCATGTTCCTCAGCTTCTGGAGCCTCTGGGTTGCTACAATCCTGTGCGTCTTCTACAGCGTGAAGATCAGTAGTTGTCACCATCCGTTCTTCATGTTCCTCAAGCTGAACGTCTCCAAGATGCTGCTGGGCTTGTTCTTGGTGTCCCTGGCAAGTTCTCTCCTATTCAGCCTCCCCTTTAAATGGCTGGTGTACAGTACCTCCATCAACAACGCTACCAACTCTACCAACACTACCAACTCTACCGGACAAGGAGGGACCATCCTGAAGGTCAATAATATCAACCAGTTCTTCCTCATCCTGACCGGATCCTCGGTGCCGCTCCTTATCTTCTGTGTGGCAGTTGCTATTCTGATCCGATCCCTGTGGAGCCACACTAGGAACATGGCCGGGGGCAACGTGGATTTTGGGAACCCCCAGATTCAGGCTCATCTGAGTGCAGTTAAAAGCATGGTCTCTTTCCTGATTCTGTTTACCATTTATTTTGCCCTATTTGTTGTATCATGTTTACCCCCATTGTTGGATGATACTGTGTTGCAATTGGTCTTTAACATTATTTCTAATGCCTACCCGCTCCTACACTCTCTCATCTTGGTAATGTACAGCAGGAAGCTCAGAGAGGCTCTCTACTGGTGTCTCCATTGTACGTGTAGAGTGCCTTCCATGGCGAGAGGTTCAGCTTAG

>Frog_T2R8--Intact

ATGCGGTCTGCAGTGCAAATGGTCATAATGGCGTTCGACTGCATCGCCTTGATAGTCGGAGGTCTGGGGGACGTGGTTGTTTTAGCTCTGTGTGTCCTAGAACGGTCAAGAAAACGTGTCCTCTGTCCGTACAGAGTCATCTCATTGAGCGTGAGCATCAGCAACCTCACCGCCATATTTCTCCAGCTGGCTTCAGATAATGAGCTGTTGCCGAACACGTTGCTCGTGGAATATGTTGATCTCTTATCAGTGTCATTTCTATGCTCCAACCTGTGGTTTTCGACATTACTGTACGTGTACTACAGCATCAAGATTGGGGCCAGACGTTTAACCTTTTACACGTGGCTTAATGCAAAGTTTCCTACAATCTTGCCATATCTGCTGTTTTCCTTACTAACAATGTCTCTTTTGACCAGCTTCATATTTCTTCTTCAACCTCTTGACAATCCCCCAAACAACTTTTCTTCAGTGCCTACCAACTACGAAGAAGCATATTTGGAAAACCCATTTATTAAGTCAGGATTTACTCAATCCTTAATAGCGCTAATATGTTTCCTGATTTCCTTCATCCTAGTGGGTCAAATCCTCCTCTCCTTGTATCGACATGTTCGGCACGTGCAAAGCAATAATGAAGCCGCTGGGAACTTCAGCCTTGAAGCTCATTTTAAAGCAGCCAAAACACTCACCATTCTCCTGGTCTTCAATGTCACCTTTTTTGTATCTATGATCACTACTTTACTCAGTCCATCTCCATCCATGCCCTTTGCCGTAAGTTGCATCTTCATTGCCGTCTCACTGTCAACCCAGCCGTATATCTTAATCCTGGGAAATACAAATATGAAGAAGCAAGCAAAAGAGACTTTTCTTCCTGTTTTTATGAATGTTGCCAACTGA

>Frog_T2R9--Intact

ATGCTGTCGGCACTCCTGCTGATCAGGACGGTGATGCTCATTGTAACATGGCCATGTGGGACCATCCTGAACTCATCCATTATAGCTGTCTATCTCAGCGACTGGAAGAAGGGAGTGAAACTTGGGGAGTGCGATCAGATCTCTGTCAGTATGGGGTGCACCAACCTGCTCTTGCAGTGCTTCGTAACGCTTGGAGTGGCCTTCATCTCATATGGACTCCACCTGCCGTTTGCCTCAAAAGTCTCTCTTGCTGTCGGTGCTGTGCTCTGGTTTTCTGTTTTTCTCTCATTTTGGCTCACAGCCGGTCTCTCCGTCTGCTACTGCCTGAGACTGGTCAATCCGTTACCTAAATGCTTTATTCATTTAAAAAGGAGACTCTCTCGCATTGTTACCCCACTTCTTCTGTGGTCAGTGGCAATTTCATTTATTATTACCGTTCCAATAAACGCCATAGTAGGCATGGCAACTGACCAGAATATAACAACAAATGATCATTATATTATTTCCAGTGTTATTTTTATGATCCTTGATGGTGCATTTGGCATTGGCTTTCCCACCCTTATTACCTCTATTTGCATTCTACTTTCCCTGATATCGCTCCTGAGGCACATCCGGAGAATGAAGCAGAACCCTCAGTTCGGGAACACCCAGCAGAAGACCCTTATAGGAGCTTGCAGGACCATGATCCTACTCATGGCTCTGAATTCTATTTTATTCCTGGTAATTTTGCGTTCAATGCTACCCCCATATAATGTGGAAACTATATGGCAAACTGTGATGTTTTCATGCGTCATGTTAAACCCCTCAGGTCAAGCCGTTGTTCTGATCTTTGGAAATTCCAAGTTACTCAGTGCCTGGACGAAAACCTTAGTTCCCCAGGGATAG

>Fugu_T2R1--Intact

ATGCTGAGCGCGAACAAAGTGGTGCTGTTTGTGCTGACGGGCCTGGTGGCTGTCGCCACCGTCTTCTTTAACGGCTACATCTTCCTGACCAGTCTGTCCAGGCTGCAAGAGAAGCAGCAGCAGACCCCCAGTGACACCATCATCATGGCTCTGGCGCTGGCCGACATGGCCCACCAGCTGGTCTGCTATTTCTGGATGACCATGGACCAGGTGGACGTCAACTGTCAGATCGAGACGAGCCCCTACACCTTCATGCTGCTCCTGATCTACAGCCTGAAGTTCACCATCATGTGGGACGTCAGCTTTCTGACCTTCTACTACAGCACCAAGCTGGTGAACACCGCCAACCGCTGCCACACCTGCGTCCAGGCCATCCTGAAGTACGTGACCCCTGCCATGGTCCTCATTCCCCTCTGCGGTTTGGGCACCTGCATGCCGATGTTGGCCGCGTTTCATCATGACAACTACACGATAGCAAACAAGGACTGCGGTGTTCTGGTGCCAGACACCCGTTCTGGGAAAATCTACGAGGTCACGTACCTGCTTCTGGCCGACGTCCTCCCCGGCGTGGTGATGGTGAAATGCTGCGTGTCCATCTCCATCCACCTCGCCATCCACCTCCGCCACATGAAGGCCAGTACCAACGGTGCCCACGGCCCAAAACTGGGATCCCAGATGAGGGTGATCCAGATGGCACTGTCCCTGGTGGCCATCTTCATCGTGTTCCTCGTGGTTGACCTGTATGTACAGTACCAAATAGCCGTGAACCATGAGAACATGCTCACGCTGACCTTCTTCTTCACGTCCACCTACACCACCATCACCGCCATGGTACTAATTTATGGGAAAAAATCCTTCTGGAAGGCTCTGATACATGAATTCAACGCTTTGCTGGATGGTTCCTGTCTGGCTTGTCTGAAAGTGCCAGAACACAAGGCTAAAGCGAACCCAAAAGTGAGGAGCTGA

>Fugu_T2R2--Intact

ATGCTTGAATCAGATGATTTGAAGCTGTGTGGCATCGCTCTGTTCCTTGTCCTGGGAGTCCTCGCCAACCTTTTCAACCTGGGTGCAATGCTGGGGCAGCAGCAGGGGAGAACTGTGGCTGTCATTATTTGCTTCATCTCGCTGGGCAATATCCTGCTCCAGACCTCCACGTGTGTCCTTGTGGCTTCCATCAGGGCTGGGGTGCTCTGCCGCCCTCACTTACCCTTCTTCTTCAGCGGGGTTCTGTATGTTTGGTTCATCAGCAGCTCCGTCAGCATCTGGTCTGTTGCCTGGCTGAATGTCTTCTACTGTGTGAAGGTCTGTAGGTTCTCCTGGATTATCTGCAGAACCCTCGAGGAGAACATCTCTAGCATCCTGAACATTACCATGGTGATAATGTTCCTGACCTCCTGCGTGATGTTCACCCCTTTCTTCGGCCTCCACTTCCAAGACCAACACGTGAATGCGACAGAAATGGGTGCTTGTGTCATCAGGAAACCTCTGCTGCCAGCCTGGGTTGACATCAACACCTATGTGATCACTTTTATCTGCTTCCTCACGCTGATGCCCTCCACCATCATGCTGCCCACCTCACTAGGCCTGGTGGTCTACCTCTGCAGACGTACGGCGAAGACGCAGAGGAGCAGCTCAGCAGAGTCCTACCTGCTGGTCTGCAGGCTGACAGTCGCTCTGGTTTGGGTTTACCTCACCACTCTGCTCATCATCTCGCTCTACTACTTCCATGCTCTCTTCGCTTCAGGGCTGAGTGCAGACGTGCTCTTCTCGGGCTTGTCCTTTTATTGTGTGGCTTGTGCAGCGCTCCTCAGCTGCTCCAACAAACACTTGAGAGGGAAGTTGAGAGCGTTGCTCTGCAGAGGAAAGAGGACAAATACAATGGTCATTAATGCAGAAGGGGAGTAG

>Fugu_T2R3--Intact

ATGGAGGTTGCTTATATCATGAGGGTTAACATGAGTGCGATCGTCCTCAACATTCTCATCAACGCGTTCTACATCGCCTGCCTGGTGCGTCCGTTTCGAGGCGAAACGCCCAAACAGCCGCTGAAGCTCCTTCTCTGGACCATGCTCTGCTGCAACCTGTCTTTCCAGGCGTCAGTCCAGGTTCTGTTCTTCTTTGTCAACGCCAGCATTGGGCTTATTGTATCCATTTTGGGGATTTTTATCTTCAGTTTGTCCACCAGCATGACCACCACTGTTTGGTTGAACTTCTTCTACTTCATCCAGATTGTGCCTTTGAAGAGCCCCGTCTTCCTTTGGATGAAGAGGAATCATAAAGCCACCATCTACTGCATCTGGATGGTTGAGAAGCTAATAACTGGGATAACTGTTACCAGCATTGTTATGTTTGATGTTTCTGTATTTAGTCAAATTTCTGATCTGATGTCGTTCAATGCCACGTTTAATAGCGATACCGTATTCAATAAATTACCCTCCCACCTGATAAAGCTGGCTAAAGCCACAATGATCATGAATGAGGTCTACTTCGTTATCTGCCTGTTCATCATGTCGCTGTCCAGCTTGTCCACTGCCATCTACCTCAGCAGACACTTGCGTCGAATGGCCTCCAAAGTGCGGTCTTGCTCCCTCTTCCGAAGCCAGGTGAGGGTCACTGTCACCGGCATCCTGCAGGGGGCGCTCTACGTACTGTTATCTACGTGGATTCTGTCCCACTATTTCTTCTATGACATTGCAGCCGGACCCGCTGCGTATATGACCTTAGCCAATATCACTGTCATCAACGTCTACATGATGGGCACCATTGTAAATCTGGGGGTGGGTCAGACCGTGTTCAGGCAGAGAGCTGTGGACCTGTGGCACAGAGCGGCACGGTGCTGCACAACCATGAGAGCACAGCAGCCTCAAAAGGGATGA

>Fugu_T2R4--Intact

ATGGAACGGCGGAATATAGATGTGGAACGCTGGCTCTACCAGCTCATCATGTTCCCTCTCAGTGGGATCAACTTCTTTTTAAACATCTTCTACATCCACTGTCTGATCTCCAACAGACAAAAGCTCAGGCAGCCTTTGAAGCTGCTGCTGTGCTTCCTCATTGGCTGCTCCGCCGCCTTTAGCATCCATTTAGCTCTCTGGAGTCCGATATTGGTCGAAATGGACGGCTCCAGCATCCCCCACCATTTTTTTTGGATGCTGATAATATTCTTCACGCACAGCAGCATGACGTGTTCGGGGTGGATGAGCATCTACGCTTACGTTAAGGTCGTCCCTTCCAAGAGAGCTCTTTTGATCTGGATCAAGAGGAACATCAAGTCCGTGGTCTACCTGTTTTTTTTCAGCCAAGAAACGCTGATCATCTTCGAAGCTTCCCTGAAAATTTCCACCTTGGTCCTGGAGTATAGGATTATTGAGGGGGCCAACTCTACGAGCAATGGCCTCCGCGACAGTGGACTCGAGGTCGGCAGCGCTGTCGTTCTCATCTTCCTCAAAGTCCACCTCCTGAGCTGCATAGCCATGATAGGAATGTGTAACTTCTCCATGGCCCACTACCTGCTCAAGCACATCAAAAGCATAACCCGGGAGGGGTTCACCACCTCGGGAATTCACGATCAAATGCGAATCGTCATCTCTGAGTTCTTCCAGGGAGCGTTCTTCTTGATGTGCAGCATTCTGTACTTCATGGACACCTTCAGCTTCCAGTACTCCCCGCATTTTTTCTTTGGTTCGTTGATGTCTCTCACGGTCACTTTACTGTACATGACAGGAACCACGGCCAGTCTGTTCATCGGTCAGGTTATATTCAGGCAAGGGGCCGTGGGGCTGTGGAAGTGGCTTACGGCACCCTGCTGTGCAAATATTTGA

>Horse_T2R1--Intact

ATGCCCTTCTCACCCATGTTGATCTTCGTGGTCATCTTTTTCCTGGAGACCTTGGCTGCGATGTTGCAGAATGGCTTCATGGTTGCTGTGCTGGGCAGGGAGTGGGTGAGATGCCTCACACTGCCTGCATGTGACATGATTGTGGCCTGTCTAGCTGCCTCTAGGTTCTGCCTGCATGGGCTGGCCCTCCTAAACAACCTCATTGACTCCTTTAACTTTTGTTCCAAAGTTTACTATTTCAACATCCTCTGGAACTTTATCAACATTCTCACTTACTGGCTTACTGCCTGGCTTGCTGTCTTCTACTGTGTGAAGATCTCATCCTTCTCTCATCCCATCTTCTTCTGGCTGAAGTGGAGGATTTCTCGGTCAGTGCCCAGGCTGCTGCTGGGCTCCCTGATCATCTCTGCTGTGACAGTCATTCCAGCAGCAGCTAGCAATGTAATTCTTATACAGATGATTGCCTCGCAGAGTTCCCATGGAAACCACACTCTGGCTGATAGAGCACAGACCTTCCATAGGTACTTTTCTCTGTCTAATTTAGTGCTTGTATTGTTGATTCCCTTCCTGCTGTTCCTGGTATCCACTCTTTTGCTCATATTCTCACTGCACCAGCACTTGGGACAGATGAGGGCCCGCAGACCCGGCTCACGTGATCCCAGCACCCAGGCTCACATCATGGCCTTGAAGTCACTTACCTTCTTTCTTGTGTTCTACGCATCGTATTTCCTGTCCCTGATTATTGCGTTTATGAAAATCACAGCCCTGCGGCGTCAGTGGCACTGGGTCTGGGAAGTGGTGACCTATGCAGGCATCTGTCTGCACTCCAGCATCCTGGTGCTAAGCAGCCCTAAGCTGAGAAATACCCTGAAGACAAAGCTTTGGAAAGCCCTGGACAAAAGGTGA

>Horse_T2R10--Intact

ATGGCAAGCACACTCAGCAATATATTTATGATCCTTTATGCTGGAGCATTCACAATGGGGATTTTGGGAAATGGATTCATTGTGCTGGTTAACTGTATTGACTGGATCAGGAGCTGGAAGTTCTCCCTGATTGACTTTATTCTCACCTGCTTGGCTATTTCCAGAATATTTCTGCTGTGCATAATAATTTTAAGAATAGCCGTAGTTGTAAACTATGAGAAAACATTGTACACTAATAATAATCTACTGATAAGTTTGGAAACCCTCTGGACAGGATCCAATTATTTCTGCATGACCTGTACCACCTGCCTCAGTGTCTTCTATTTCCTCAAGATAGCCAACTTTTCTAATCCCGTTTTCCTCTGGATGAAATGGAGAATTCACAAGATGCTTCTCATCATTGTACTGGGAGCAACCTTCTCTTTCTGCTTGTGCCTTCTTTTTAAGAATACACTAGTTAAGAGCCTGATCGTAAACCAGGTAAATGCTGAAAGAAATTTGACTTGGAACTTCACAGTGAGAAAATATTTATTAACTTCTCAAATTCTCCTTGACATAATGTTCATCACCCCCTTTGTAGTGTCACTGGCCTCCTTACTTCCTTTAATCCTCTCCTTATGGAGCCATACTAAGCAGATGAAAGGTACAGGTTCTAGGGATACTAGGACAGAGGCCCACGTGAGAGCCATGAAGTGTATGATTTCATTCCTACTCCTCTTCTTTATGTACTATTTTAACCATATTATAAAATATTCAGTCTATGCTGTTGTAGATACTTTTGTAGTAAACACTTTTGCTAATGTGCTAGTGTTTTTTGATCCATCTGGCCATCCATTTCTTCTGATTTTGTGGAACACCAAATTGAAACAGGCTTTTCTCAGTGTCCTGGAGAAGCTGAAGTGTTGCGTGAATCTAAGGAAACCTACAATCCCATAA

>Horse_T2R11--Intact

ATGGTAAATTTACTACCAAGCATTTTTTCTGTCTTAATAACGACGGAATTTATTCTGGGAAATTTTGCCAATGGCATCATAGCACTGGTGAATTGCATTGACTGGGTCAAGAGACATAAGATGTCCTCAGCTGATCAAATTCTCACTGCTCTGGCGGTCTCCAGAATTGTTTTGCTCTGGGTACTATTAATGAATTGGTATACAGTTGTGCTCCATCCGGGTTTATATAGTTTGGAAGTAAGAATTTTTGTTCGTATTGCCTTGACAGTAAGCAACCATTTTAACATCTGGTTTGCTGCTAGCCTCAGCATATTTTATTTGCTCAAGGTAGCTAATTTCTCTAGCTTTATATTTCTTTACCTAAAGCGGAGAGTTAAAAGTGTACTTCTCATAATACTGTTGGGGACTCTGGTCTTTTTGGTTCCTCATCTTGCAATTCTATGCATATATGAGAATATTCAGACTAATGAGTATGAAAGAAACATCACTCAGAAGACCAAATTGAGGGACATTTTTCACTTCTCATATATGAATCTATTCATGCTAGTAAACTTCACACCATTTTCTATGTCGCTGACATCTTTTCTGCTGTTAATCATTTCCCTGTGGAATCATCTCAAGAAGATGCAGCTCAGTGGCAAAGGATCCCAAGATATCAGCACCAAGGTCCACATAAGAGCCATGCAAACTGTGGTCTCCTTTCTCTTCTTGTATGTCAGTTACTTCCTAGCTCTGGTTACTTTAGTTGAGAGTTATAATAGGCTGCATAATACACTGCTTGTCATGCTTTCTGAGGCTCTTGCAATGCTCTATCCTTTAAGCCACTCATTTATCCTGATTTGGGGAAACAAGAAGCTAAGACAGGCCTTGAAAACCATAAGAAAGATGATAAGATTCCCATACCATTAG

>Horse_T2R12--Intact

ATGGTCAGTGTCGTACAGAGCACACTTACAATCATTCTAAGTGCTGAATTCATAATTGGAAATTTAGGAAATGGATTCATAGCACTCGTGAACTGCATTGACTGGGTCAAGAGGAGAGAGATCTCTTCAGCTGATCAAATCCTCACTGCTTTGGCAATCTCCAGAATTGGTCTGCTCTGGTTAGTATCTATAAACTGGTATATATCTGTGTTTTTTACAGTTTTACTTGTGCCTGGAAAACTGTTAAGAGTGAATAGTATTGGCTGGACAGTGACCAATCATTTCAGCAACTGGCTTGCTACAAGCCTCAGCATCTTTTATTTTCTCAAGATAGCCAGTTTTTCTAACTCTATTTTTCTTTACCTAAAGTGGAGAGTTAAAAAGGTGATTTCAATGATACTGCTGGTGACCTTGGTCCTCTTGATTTTTAATATTGCACTGATGAACATGCATATTAATGTCTGGATCAATGAACATAAAGTAAACATGACCGGCACTTCTAGGATGAGCAACTTTGTACAACTTTCCACTCGTACTTTATTCATTAACACTCTGTTCACTATCATACCCTTTGCTGTGTCCCTGATAATTTTTCTTCTGCTTATCTTCTCCTTGTGGAAACACCTCAAGAAGATACAGCACAATGCCAAAGACTCCAGAGATGCCAGCACCGAGGCCCACATAAAAGCCATGAAAAGCATGATTGCTTTCCTCCTACTATTTGCCATTTACTTTCTGTCTCTTTTTGTGTCAATTTGGAGCTTTAAATTTCCAGAGAGAAAGCAGATCATTATGTTTTGCCAGGTTATCGGAATTAGCTATCCTGCAGGTCACCCATATGTCCCGATTCTGGGATACAATAAGCTGAGACAAGCCTTTCTTTCAGTGCTGTGCTGGCTTAGGTCGAAGATGGAGAATCTTCAGGCCCGTAGACCATTTAGAGATTCATCTTGCATATCCTAG

>Horse_T2R13--Intact

ATGCTCAGTACAGAAGAAAACATCTTTATGGTCATAATAACTGGTGAATTCACAGTTGGAATGTTGGGGAATGGATACATTGGACTAGTAAACTGGATTGACTGGATTAAGAAGAAAGAGACCTCTTCAATTGACTACATCTTCACCAGTTTAGCTATCTCCAGAATTTGTTTGATTTGTGCAATGGTGCTGAATGCCCTTATAATAGTATTCTACCCAGAGGTTCATGAAAATGATAAAATAAAGATAGTCAACATCTTCTGGACACTCACCAACTACTTAAGTATGTGGTTTGCCACCTGCCTCAATGTCTTCTATTTCCTCAAGGTAGCTAACTTCTCCCATCCACTTTTTCTCCAGCTGAAGTGGAGAATTGACAGGGTGGTTCACTGGATCCTGCTGGGGTGCTTGGCCATTTCCTTGTTGATCAGCCTTATATTTGCAATGACCCCAAAATATGAGTTGCTGAAAATTGCAAAACATAAAAGAAACTTCACTGAATCATTTCATGTGAGTAAAATTCAATACTTCAGCCCAGTGACAATCTTTAGCCTGTTGGCAACTGTCCTATTTACTGTGTCATTGATTTCATTTTTCCTTTTAATTATGTCCCTATGGAAACATATTAAGCAAATGAAACTCAATGTTACAGGCTGCAGAGACCCCAGCACAGAGGCCCATGTGAGACCCATGAAAACTGTGACTTCATTTCTCTTTCTTCTTTTTGTATACTATCTGGCCTCTCTTTTAATGACGTTTAGCTACCTGATGAAAGAAAGAAAGTTAGCTGTGATGTTTGAAGAGGTTATAGCAATTTTCTATCCCTCAGGTCACTCACTTATTTTAATTATTGGAAACAACAAGCTGAGGCAGGCATTTGTCAGAATGCTGAGATGTGGAAAAACAGCCTGCATGATGTAA

>Horse_T2R14--Intact

ATGTTCACTGTATTGGATAAAATCTTTCTGACGCTGGCAGGAGTGGAATTCATAATCGGAATGTTAGGGAATGTGTTCATGGGGCTGGTAACCTTCTCTGAATGGGTCAAGAACCAAAAGATTTCCTTAGCTGACTTCATCCTTACCTGCTTGGCTATCTCCAGAATCACTCAACTGTTGGTTTTATTGTTTGAATCATTTATGCTGGGACAACCTTCGTGTTTCTATGCCACTTATAAACTAGCAAAACCTATCACTTTGCTTTGGAGAATGACTAATCACTTAACCACATGGTTTGCTACCTGCCTAAGCATTTTCTACCTCCTTAAGATAGCTCACTTCTCCCATTCCCTTTTCCTCTGGCTGAAGTGGAGAATGAACAGAGTGGTTCTTGTGATTTTTGTATTTTCTTTGTTGTTTCTGATTTTTGACTTTCTATTGCTAGAAACATTTAACGATCTCTTCTTCAATATCTGTAAAATAGATCAAAGTAATCTGACTTTATATTTAGATGAAAGAAAAATTCTTTATGTTAAAACCCAGATTCTTCTTAGCTTGACCTATTTCATACCTATTGCTCTGTCTCTGATTTCGTTGCTCCTTTTATTTCTGTCCTTGGGAAGACACAGCAGAAATTTGAAGCTCAACTCCATGGGCTCAAGAGATTCCAGTACAGAGGCCCACAAAAGGGCCATGAAAATGGTGATGTCCTTCCTCTTCCTTTTCGTAGTTCATTGTTTTTTCACACAATTGACACATTGGATAATTATGTTTTGTAACAGCAAATTCACAAAGTTTGTCTTATTAGCATTATATGTCTTTCCTTCAGGCCACGCATTTATTTTGATTCTGGGAAATAACAAGCTAAGACAGACAGCCTTGAAGGTACTGTGGCATCTTAAAAGCTCCTTAAAAAGAGAAAATCCATTAGCTTTACAGGATAGACTTTCCAGAGCCTTTTCAAAGATAGTAACTCAGTGA

>Horse_T2R15--Intact

ATGGCAAGCACACTCAGCAATATATTTGTGATCCTTTATGCTGGAGCATTCACAATGGGGATTTTGGGAAACGGATTCATTGTACTGATTAACTGTATTGACTGGATCAGGAGCTGGAAGTTCTCCCTGATTGGCTTTATTCTCACCTGCTTGGCTATTTCCAGAATATTTCTGCTGTGCATAACAATTTTAGGTATAGGCTTAGATGTAAACTTTGAGAAAATATTGTACACTAATAATAATCTACTGATAAATTTGAAAAACCTCTGGATAGGATCCAATTATTTCCGCATGACCTGTACCACCTGCCTCAGTGTCTTCTATTTCCTCAAGATAGCCAACTTTTCTAATCCCGTTTTCCTCTGGATGAAATGGAGAATTCACAAGATCCTTCTCATTATTGTGTTGGGAGCAACCTTCTCTTTCTGCTTGTGCCTGATTTTTAAGGAGGCAGTATTTAAGAGCCTGATCAAAAACAAGGTAAATGCTGAAAGAAATTTGACATGGAACTTCACAATGAGAAAATATTCATTAACTTCTCAAATGCCCTTTGACATAATGTTCATCATTCCCTTTTTAGTGTCACTGGCCTCTTTATTTCCTTTAATCCTCTCCTTGTGGCGCCATATCAAGAAGATGAAGGGCACAGGTTCTAGGGATCCCAGGACAGAGGCCCATGTGAGGGCCATGAAGTGTATGATTTCATTCCTACTCCTCTTCTTCTTGTACTATTTGAGCCATATCATAAAATATTCTGCCGATGCGGTTGTAGATACTTTTGTAGCAAAGATATTTGGTAATGTGCTAATATTTTTGGATCTGTCTGGCCATCCATTTCTTCTGATTTTGTATAACAGCAAATTGAAACAGGCTTCTCTCAGTGTCCTGGAGAAGCTGAAGTGTTGCATGAATCTAAGGAAACCTACAATCCCATAA

>Horse_T2R16--Intact

ATGACAATTGACATGAAGAGCACCTTAATGATCATAGCAGCTGGAGAGTTCTCGATGGGGATCTTAGGGAATGCATTCATTGGATTGGTGAACTGTATGGACTGGATCAAGAACAGAAAGATTGCCTCCATTGATATAATCCTCACGAGTTTGGCCATATCCAGAATTTGTCTATTGTGTATTATACTATTAGATTGTTTTATATTGGTGCTGTATCCAGATGTCTATACCAGTGGTAAACAAATGAGAATCATTGATTTCTTCTGGACACTAACCAACCATTTAAATGTCTGGTTTGCCACCTGCCTCAGCATTTTCTATTTCCTCAAGATAGCAAATTTCTTCCATCCCCTTTTCCTCTGGATGAAGTGGAGAATTAACAGTATGATTCCTAGGATTCTGCTGGGATGTTTGGCCCTCTCTGTGTTTATTAGCCTTCCTGTCCCTGAGAATCTGAATGATGATTTCAGGAGCTGTGTCAAGAGAAAGTGGAAAACAAACTTAACTTTGAGATGCAGAGTAAATAAAGCTCAATATGCTTTCATAAAGCTATTTCTCAACCTGTTAACACTATTCCCCTTTTCTGTGTCCCTGATCTCATTTTTCCTCTTGATTCTTTCCCTGTGGAGACACACCAGGCGGATGCAACTCAATGCCACAGGGAGCAGAGACCCCAGCCTGGAAGCCCACGTGGGAGCCATGAAAGCTGTCATCTCCTTCCTCCTCCTTTTCATTGCCTACTATCTGGCCTTTCTTGTGGCCACCTCCAGCTACTTCATGCCAGAGACTGAATTAGTTGTTATGGTTGGTGAGGTGATAGCTTTAATCTATCCCTCAAGCCATTCATTTATCCTAATTCTGGGGAACAACAAATTAAGACAAGCATCTCTAAGGGTGCTATGGAAAGTAAAGTATCTTCTAAAAAGAAGAAATTTCTGA

>Horse_T2R17--Intact

ATGGAAAGCACATTGAAGAATATATTTATGATCATTTTGGCTGGAGAATTGTTAATGGGGATTTTGGGAAATGGATTCATTGTGCTGGTTAACTGTATTGATTGGATCAGGAGCTGGAAGTTCTCCCTGATTGACTTTATTCTCACCTGCTTGGCTAGTTCCAGAATATTTCTGCTGTGCATAATAATTTTAGGTATAGCTTTAGATGTAAACTTTGAGGAAATATGGTACAATAATAATAATCTACTGAGAAGTTTGGAAATCCTCTGGACGGGATCCAATTATTTCTGCATGATCTCTACCACCTGCCTCTGTGTCTTCTATTTCCTCAAGATAGCCAACTTCTCTAATTCCATTTTCTTCTGGATGAAGTGGAGAATTCACAAGATTCTTCTCATTATTGTGTTGGGGGCAACCCTCTCTTTCTGCTTGTGCATTCTTTTTAAGGATACATTAGTTAGGAGCCTGATCAAATACCAGGTAAACGCTGAAAGAAATTTTACATGTAACTTCATAGAGAGAAAATATGATTTATTAACTCCTCAAAAGCTCATTAACATAATGTTCATCATCCCCTTTTTAGTGTCTCTGGCCTCCTTAGTCCTTTTAATCCTCTCTTTATGGAGTCATGCCAGGCAGATGGAAGACACAGGTTCTAGGGATCCTAGCAGAGAGGCCCATGTGAGAGCTATGAAGTCTATGATTTCATTCCTACTCCTCTTCTTCATATACTATTTGAGCCATATTATAATAAATTCAGCCAATACCACTCTAAACACTTTTGTGGCAAAGATTTTTGCTCACGTGCTGTTATATTTGTACCCATCTGGCCATCCGTTTCTTCTGATTTTGTGGAACAGCAAATTGAAACAGGCTTCCCTCAGTGTCCTGAAGAAGCTGAAGAGTTGCATGAACCTAAGGAAACCTACAATCCCATAA

>Horse_T2R18--Intact

ATGATAACTTTACTACCAAGCATTTTTTCCATCCTAATAACAACAGAATTTTTTCTTGGCAATTTTGCCAATGGCTTCATAGCACTGGTGAACTGCATTGACCGGGTCAAGAGACAAAAGTCGTCCTCAGCTGATCAAATTCTCACAGCTCTGGCGGTCTCCAGAATTGGTTTACTCTGGGTAATATTAATAAATTGGTATGTGACTGTGCTTCCTTCAGTTTTTTGTAGTTTAGAAGTAAGAATTATTGTTTGTGTTGCCTGGACAGTAAGCAACCATTTTAGCATCTGGCTTGCTACTAGCCTCAGCATATTTTATCTGCTCAAGATAGCTAATTTCTCTTGCTGTATATTTCTTTACCTAAAGTGGAGAGTTAATAGTTTACTTCTTGTAATACTGTTGGCAACTTTGGTCTTTTTGGTTCCTCACTTTGCAGTGCTGTGCGTAGATGAGACTATGCAGACTAAAGAGTATGAAGGAAACGTCACTCGGAAGACCAAATTGAGAGATGTTGTAGGCCTTTCAAATATGACTCTATTCACGCTAATACACTTCATACCCTTTACTATGTCCCTGACATCTTTTCTGCTGTTAATCGTTTCCCTATGGAAACATCTCAAGAAGATGCAGCTTAATGGCAAAGGATACCAAGATCCTAGCACCAAGGTCCACATAAGAGCCATGCAAACTGTGCTCTCTTTTCTCCTGCTATATTCCGGTTACTTCTTGGCTCTAGTTATCTCAGTTTGGAGTTCTAATCGACTGAAGAATGAACTAGTTCTCATGATTTGCCAGGCTCTTGGAATGCTGTATCCTTCAAGCCACTCATTCATCCTGATTTGGGGAAACCAGAAGCTAAGAAAGGCCTTGAAAAATGTAAGACAGATTATAAGATTACCACACGATTAA

>Horse_T2R19--Intact

ATGCCGAATACATGGGAGAAAGTTTTCATGATTGTAACAGGTGGAGAATTTATAATAGGAATTTTAGGGAATGGATTTATTGGACTCACAAATTGCATTGCCTGGATTAGAAATCGGAAGTTGTGCTTGGTTGACTTCATTCTTACCAGTTTGACCTTGGCCAGAATCAGTCAATTATGCCTAACAATTATCAATTTGTTTTTAGCACTGGTCTATCAGAAAATCCCTGAAACTATGAAAACAAACAATATCCTTACCATCATGGGGATACTGATCAACCACTTGACCACTTGGTTGACTGCTTGTCTCGCTGTCTTTTATTTCCTGAAGATCTCCAGTTTCTCCCATCCTTTTTTCCTTTGGCTGAAGTGGAGAATTAACAAGGTAGTTTACATGGTTCTGCTGTCATCTTTGCCCTTCCTGTTGATCAACTTTCCTTTGCCACTTAATTTTGATGTCTTCTGGTATCATGTCCAAAAGAAACATCAAAGAAATATGACGGGATTAGTCAATGTGAGTAAAAGCAAACATTTAAGTGCCAGGACCGTCTTCATTATTGGATCCATCCTTCCTTTCTCTCTTTCCTTGATTTCCTTTTTCCTTTTGCTCTTTTCCTTGCGGAGACACATGAAACGCAATTTGCTCAACTTCAAGGATTCCAGAGACCCCAGCATGGAGGCCCATGTCAGAGCCATGAAAACTGTGCTTCTCTTTCTTGTGCTCTTTGCTCTGTACCAATTATCATTTTTCATGACATTTTGGGGATATTTTTCACTACAGAACAAGCTGGTTGTGATGTTTGCTTATATGATAGGAATTCTCTATCCTTCCGCTCACTCATATGTTGTGATTTTTGGAAATAGTCAAATGAGGAAAGCCTTCTTGGGGCTTCTTTGTCACGTGAAGTGTGTCCTGAAAGGAAGAGCAATCTCAGCTGCATAG

>Horse_T2R2--Intact

ATGGCGACAGTGAACACAGATGCCATGGATAAAGACACGTCCAGGTTTAAAATCATCTTTACCTTGCTGGTCTCCGGAATAGAGTGCACCATTGGCATCGTTGGGAATGGCTTCATCACAGCTATCTATGGGGCGGAGTGGGCCAGAGGCAAAAGACTACCCATTGGTGACCGCATCCTTTTGATGCTGAGCTTTTCCAGGTTCTTGCTACAGATCTGGATGATGCTGGAGAATACTTACAGTCTACTATTGCGGGTCATTTATACCCAAAACGCAGTGTATAAACTTTTCAAAGTCATCATCATGTTTCTGAACTATTCCAACCTCTGGCTTGCTGCCTGGCTCAATGTCTTCTATTGTCTTAGAATTGCAAGCTTTACTCATCCTTTGTTCTCCATGATGAAGAGGAAAATCATGGTGCTGATGCCTTGGCTTCTGAGGCTGTCACTCCTCATCTCTTTATGCTTCAGCTTTCCCTTCTTTAAAGATATCTTCACTGTGTATGTGAATAGTTCCATTCCTATCCCCTCCTCCAACTCCACTGAGAAGAAGTACTTCACTGAGACCAATGTCTTCAATCTGATTCTTCTTTATTATCTGGGGATCCTCATTCCTCTGATCATGTTCATCCTTGCAGCCACCCTGCTGATCATCTCTCTCAAGAGACACACCCTACACATGGAAAGCAATGCCACTGGCTTCAGGGACCCCAGCATGGAAGCTCACATGGGGGCCATCAAAGCTATCAGCTACTTTCTCATTCTCTACACTTTCAATGCAGTTGCTCTATTTCTTTCTATGTCCAACATCTTTAATGCTGATAGTTCCTGGAATATTTTGTGCAAAATCATCATGGCTGCCTACCCAGCTGGCCACTCAGTGCTACTGATCTTGGGAAATCCTGGGCTGAGAAGGGCCTGGAAGAGGTTTCAGAACTGA

>Horse_T2R3--Intact

ATGATGAAAACCTGCGGTTCCCCAGAAAATAAATTGTCACCTTTTTGGATCACCTTAGTTTTCACAATTATAGGCGCTGAATGCATCATTGGTATCTTTGCAAATGGATTCATCGTGGCTATAAATGCAGCTGAATGGATTCAGAATAAGGCAGTTTCCACAAGTGGCAGGATCCTGCTTTTCCTGAGCGTATCCCGAATAGCTCTCCAAAGCTTCCTGATGCTAGAACTTACCTTCAGCTCAACATCCCCAAGTTTTTATAATGAAGATGTTGTATATGACACATTCAAAGTAAGTTTCATGTTCTTAAATTATTGTAGCCTCTGGTTTGCTGCCTGGCTCAGTTTCTTCTACTTTGTGAAGATTGCTGATTTCTCCTACCCCGTTTTCCTCAAGCTGAAGTGGAGAATTTCTGGATTGATGCCCTGGCTTTTATGGCTATCAATGCTTATTTCCTTGGGCAACAGTGTGGTCTTCTTCAAAGACATCTACACCGTGTATTCTAACAATTCTTTTCCTATCCCCTCCTCCAACTCCACTGAGAAACAATACTTCACTGAGACAAATGTGTTCAATCTGGTTCTTTTCTATTACCTGGGGATCCTCATTCCTCTGATCATGTTCATCCTTGCAGCCACCCTGCTGATCATCTCTCTCAAGAAACACACCCTACACATGGAAAGTAATGCCACTGGCTTCAGGGACCCCAGCATGGAGGCTCACATGGGGGCCATCAAAGCTACCAGCTACTTTCTCATTCTCTACGTTTTCAATGCAATTGCTCTGTTTCTCTATATGTCCAACATCTTTGACATCAACAGTTCCTGGAACATTTCGTGCAAAATCATCATGGCGGCCTACCCTGCTGGTCACTCCATTCTACTGATTCAGGACAACCCTGGGTTGAGAAGAGCCTTGAAGAGGCTTCAGGCTCGAGTTCATCTTTACCTAAAAGACTAG

>Horse_T2R4--Intact

ATGCCCTCTTCACTCATGTTGATCTTCATGGCCACTTTTTCCCTGGAGACGTTGATTGCAATGTTGCAGAATGGCTTCATTGTTGCTGTGCTGGGCAGGGAGTGGGTACAAGGCTGCACACCCTCCTCGGGGGATGTGATTGTGGCCTGCCTGGCTGCGTCCCGGTTCTGCCTGCATGGGCTGGCCCTCCTGAACAGCTTCCTGGGCTTCTTTAAGTTTTCTTCCAAAATTTACTATTTCAGCATCCCCTGGGACTTTATCAACACTCTCAATTTCTGGCTGACTGCCTGGCTTGCTGTCTTCTACTGTGTGAAGATCTCAACCTTCTCTCATCCCACCTTCCTCTGGCTGAAGTGGAGGATTTCTCGGTCAGTGCCCAGGCTTCTGCTGGGCTCCCTGATCATATCTGGTGTGACAGTCATCTCATCAGCTACTGGGAATAGCATCGCTGTGCTGAGGAGTACCTCCCAGAGTTCCCCTGGAAACCACACTTTGGCTGATAGAATAAGCCCCTTCTTTCGGCACTTTTTTCTGAGTCAAGAGCTACTTGTGTTGTTGCTTCCTTTTCTCCTGTTCCTGGTGTCCACCCTCTTGCTCATGTTCTCACTGCACCAGCACCTGCAGCAGATGAGGGCCCACAGACCCAGCCCACATGATCCCAGCACCCAGGTTCACATCACGGCCCTGAAGTCACTTTCCTTCTTCTTTGTGTTCTACACATCATATTTCCTGTCCCTGATTATTGTTTCTATGCAAATCACAGCCCTGCAGCATCAGTGGCACTGGGCCTGGGAAGTGGTGACCTATGCAGGCATTTGTCTGCATTCCAGCATCCTGGTGCTAAGCAGCCCCAAGCTGAGAAAGGCCCTGAAGACAATCTTTGGAAAGCCCTTGACAAAAGATGCTTCATCTCAAGTTATCAGTATCAATAACCAGTATCAATGGACAAGCCCATGA

>Horse_T2R5--Intact

ATGGTCTCCCCTTTATCAGCTATTCTTCATGTTCTCATCATGTCAGCAGAATTTATCACAGGGATTACAGTAAATGGATTTCTTATAATCATCAACTGTCATGAATTGATCAAAAGCAGAAAGCTAACACCAATGCAACTCCTTTTTGTATGTATAGGGACGTCTAGATTTGGTCTGCAGATAGTGTTAATGGTACAGAGTTTCTTCTCTATCTTCTTCCCACTCTTATATGCTGTAAAAATTTATGGTCCAGTGATGATCTTCCTTTGGATGTTTTTTAGCTCTGTCAGTCTCTGGTTTGCCACCTGCTTGTCTGTATTTTACTGCCTCAAGATAGCAGGCTTCACTCAGTCCTATTTTCTTTGGCTGAAATTCAGAATCTCAAAGTTAATGCCTTGGCTGCTTCTGGGAAGCCTGCTGGCCTCCGTGAGCATTGCAGCTCTGTGTACGGAGGTAGATTACCCTCTACACGTGAGTGATATCCTCAGGAACACCACGCTAAAGAGAACGGAACTCAAGATAAAGCAAATTAGTGAAGTGCTTCTTGTCAACTTGGCATTGATATTTCCTCTGGCCATATTTGTGATGTGCACTTTTGTGTTATTCATTTCTCTCTATAAGCACACTCGTCGGATGCAAAAAGGACCTCATGGTTTCAGCGATGCCAGCACAGCAGCCCATATAAATGCATTAAGAACAGTAATAACATTCTTTTGCTTCTTTATTTCTTATTTTGCTGCCTTCATGACAAATATAACATTCAGTGTTCCTTACAGAAGTCAGAACTTCTTTGTGTTGAAGTTCATAATGGCAGCATATCCCTCTGGCCATTCGGTTATTATAATCTTGAGTCATTCTAAGTTCCAACAACCATTCAGGAGACTTCTCTGCCTCAAAAAGAATTAA

>Horse_T2R6--Intact

ATGCCTTCCTCACTCACATTGATTTTCATGGTCATCTTTCTTCTAGAGTCCTTGGCTGCAATGTCGCAGAATGGCTTCATTGTTGTTGTGCTAGGCAGGGAGTGGGTGCGATGCCGCACACTGCCCTCAGGTGACATGATTGTGTCCTGCCTGGCTGCCTCCCAGTTCTGCCTGCATGGGATGGCTCTCCTAAGCAACTTTATGTCGTTATTTAATTTTTGTTCCCAAGTTTCCTATTTGGGCGCTCTTTGGGAGTTTATCAACAGTCTCACTTTCTGGCTTACTGCCTGGCTTGCTGCCTTCTACTGTGTGAAGATCTCATCCTTCTCTCATCCCATCTTCTTCTGGCTGAAGTGGAGACTTTCTCGGTCAGTGCCCAGGCTGCTGCTGGGCTCCCTGATCATATCTGTTGTGACAGGCATCTCATCAGTCAGTGGGAATATAATTCTTATGCAGATGATTGCCTCCCTGAGGTCCCATGGAAACCACACTCTGGCTGATAGAATAAGGACCTTCTCTTGGCACTTTTTTCTACCTCAAGATGTGCTTGCAATGTCAATTCCCTTCCTCCTGTTCCTGGTATCCACTCTCTTGCTCATGTTCTCACTGCGCCGGCACTTGCGGCAGATGAGGGCCCATAGACCCGGCCCACATGATCCCAGCACCCAGGCTCACACCATGGCCCTTAAGTCACTTACCTTCTTCCTCGTGTTCTACACATCATATTTCCTGTCCCTGATTATTACTGTTATGAAAATCACAACTCTGCAGGATCAGTGGCACTGGGCCTGGGAAGTGGTGACCTATGCAGGCATCTGTTTGCACTCCAGCATTCTTGTGCTAAGCAGCCCCAAGCTGAGAAAGGCCCTGAAGATGAGACTTTGGAAAGCCCCGGAGAAAAGGTGGTTCATCGCAAGTATCAATATCAATAATCAGTATCAACAGACAAGCCCTTAA

>Horse_T2R7--Intact

ATGCTTGAGACTTACCTTTTTGTCTATCTTCTTTCTGCAGTAATTCAATTGCTCGTTGGAGTTTTAGCAAATGGCATCATTGTGGTTGTGAATGGCACTGGCTTGATCAAGCAGAGAAAGATGATTCCATTGGACCTCCTTCTTTCCTGCCTGGCGATTTCTAGAATTTGTCTGCAGTTGGTCATCTTCTGCATTAATCTGAATGTTCTCTCCTTGACTGAATTTACTATATTTCCGGACAATTTTGCAATTTTCACGTTTGTAAATGAATTGGGACTTTGGTTTGCCGCATGGCTCAGCGTTTTCTACTGTGCCAAGATTGCCTGCATCGCTCACCCACTCTTCTTCTGGTTGAAGATGAGGATAGCCAAGTTGGTGCCATGGCTGATCTTCGGGTCCCTGCTATATGCATCGATCATTTCTGTTTTGCATAGCAAACATACAGGGATTCTGTTCCAAAAAATCTGGTTGGACCTTTTCTCCAATAACACAACAGCTCAAATCAGAGAACTATCTGTTTTACAGCGTTCCTTTCTTGTCATTGAGTTCTCATTACCGTTTCTTATCTTCCTTTTTTCTACTCTGCTCTTGATATTTTTCCTGGGGAGACACACCTGGCAGATGAGAAACACAGTGACAGGCACCAGGAATGCTAGCATGCGTATCCACATCAGTGCACTTCTGTCCATCCTGTCCTTCCTGGTCCTCTACCTCGCCTACTATGTGATGCCTGCTTTATTCTTTTCTCAAATTTTTAAGCTCAGGAACCCCATCTTTCTATTCTGCCTCTTTGTGGTTGGATCATACCCCGGTGGACACTCTGTTATCTTAATTTTAGGAAATCCTAAACTGAAACAAAATGTGAAGAAGACCCTCCTTCACAGTAAGTACTGTCAGTGA

>Horse_T2R8--Intact

ATGTTGGGACTCACTGAGTGGGTGTTTCTGGTTCTATCTGCCACTCAGTTCCTTCTGGGAATGCTGGGGAATGGTTTTATAGAGTTGGTCAATGGCAGCAGCTGGTTCAAGAACAAGAGAATCTCTTTGTCTGACTTCATCATCACTAACCTGGCTCTCTCCAGGATCGTTCTGCTGTGGGTTCTCTTGGTTGATGGTGTTTTAATGGTGTTCTCTTCCAAAGTACGTGAGGAAAGGATAGTAATGCAAATTATTTATGTTTCCTGGACATTTACAAACCATCTGAGTATTTGGCTTGCCACCTGTCTCAGTGTCCTCTACTGCCTGAAAATTGCCAGTTTCTCCCACCCTACATTCCTCTGGCTCAAGTGGAGAGTTTCCAGAGTCGTCATATGGATGCTCTTGGGTGCGCTGCTCTTATCATGTGGCAGTGCCGTGTCTCTGACCCATGGATTTAAGATTTATTCTGTTTTCCGTGGAATCAATGGCACAAGGAATGTGACTGAGCACTTTAAAAAGAGAAATGAATATGGATTGATCCATGTTCTTTGGACTCTGTGGAACCTCCCTCCGTTAATTGTGTCTCTGGCCTCCTACTTTCTGCTCATCCTCTCCCTGGGAAGGCACATGTGGCAGATGCAGCAAAATGGTACCAGTGCCGGAAATCTAAGCACTGAAGCTCACAAGAGGGCCATCAAAATCATCCTCTCCTTCCTCTTTCTCTTCCTACTTTACTTTCTTGCCTTTATAATTACAACAGCCAGTGATTTCCTACCAGGAACTAAGATGGTTAAGATGATTGGAGAAATAATTACAATGTTTTATCCTGCTGGCCACTCATTTATTCTCATTCTGGGAAACAGCAAGCTGAAGCAGATGTTTGTGGAGATGCTCTGGTGTAAGCCTGGTCATCTGAAGTCTGGATCCAAGGGCTCCTTTTCCCCATAG

>Horse_T2R9--Intact

ATGCTTCGGATATTTTTTATCTGTTCTGTTATTGTCTCAGTAATTTTGACCTGTGTAGGACTCATTGTGAACCTGTTTATTGCAGTAGTCAATTATAAGACTTGGGTCAAAAGCCACAGAATCTCCTCTTCTGATAGGATCCTGTTCAGCTTGGGCATCACCAGATTTCTTATGCTGGGAATTCTTCTACTGAATAATGTCTACATCAACTCTCTGAAAGTTGAAAGGTCAGTCTACTTATCCACTTTTTTCCTGTTGTGTTGGATATTTTTGGACTCTAATAGTCTCTGGTCTGTAACCTTGCTCAACGCCTTGTACTGTGTGAAGATTACTAACTTCCAACACTCCATGTTTCTCCTGCTGAAACGAAATCTCTCCCCAATGATCCCCAGGCTACAGCTGGCCTGTGTGCTGATTTCTGCCTTCACCACTCTCCTGTATGTTTTGCTCAGACAGACATCACCCGTCCCTGAATTTGTGACTGGGAGAAACAGCACAGTATTTAACATCAATGAGGGCGTCTTGTTTTTGGTGACCTCTTTGGTCTCGAGCTCATTTCTCCAGTTCATGATTAATGTGACGTCTGCTTCCTTGTTAATAAATTCCTTGAGGAGACATATACAGAAGATGCAGAGAAATGCCACTGGCTTTTGGAATCCCCAGACTGAAGCTCATGTGGGTGCAATGAAGCTGATGATCTGTTTCCTCATCCTCTACATTCCATATTCAGTTGCTGCCCTACTCCATTATGTCCCTTCTTCTGTAGGGATGAATTTAGGAGCCAGATCCATTTGTGTGATTGTTTCCGCCTTATACCCTCCAGGACATTCTGTTCTTATTATTCTCACACATCGTAAACTGAAAACAAAAGCAAAGAAGATTCTTTGTTTCAACAAATGGTGGAATTTCAGTAGTAAATAG

>Lizard_T2R1--Intact

ATGTCCCCTCTTGACTCCATTGTTTTTTTGGTAACCGCAGTTGTCTTAACCATCAGTGGACTCATCTCCAATGGCTTTATTGTTACAACAATTACCATTAAATGGATCAAATTCAGGAGCCTTGCTTCTAGTGAACTGCTCTTTCTGACTTTGAGTCTTTCCAATTTTGGGGCCGGAGTGTTCCTGCTTCCATTTTATATTGATGATTCCACAATATTCAGCTTCAAGCAAAACATGGCACTAAAAATACTCTTCCCAGTTGCTGTATTTGCTGTCTTCTCCAGATTCTGGCTCACTGCTTGGCTCTGTGTCTTCTATTGCATCAAGATAGTGAACAGCACCCATTTCCTCTTCCTTTGGTGCAAACTCAGGATATCATGGCTAGTAACACATCTTATTACAGGATCTCTGGTCATATCCTTTTTTGCTTCTCTGGGGGCATTGGAAAAGGATTCTATACACCTCCAAAGCAACGTAACAACAATGTTCCCAACATTGAGCCAAGGAAAATCACTGAAAGCTAGTGACTTTCGTTTCCAATTATTCTTTTTAATTTTTGGTTCATGTTCTCCTCTTCTTATAGTTTTTCTTTGCTCTACCTTGGTTGTTGCCTCACTCTCTAGACATGTTTGTCGGATGACAGATAATAATAACTTTCAAAGAAGAGCTCATTTCAAGGCAACTGGGACAGTGCTCTCCTTGCTGTTGGTTTATCTTTCATTTTTCATGGCACAGATTTTGTCTATGGCTGCAAATGTAACGTGCACTGGAAGACAATTTATTTCATCAGTGATGATCGCATATGCTCCAGTCCAGGCTGCCATCCTGGTGCTGAGCAACCCCAAATTAAAGCAGGCATTAACTGTGATGGTTCAAAGAGCAAAGCCTTAA

>Lizard_T2R10--Intact

ATGGCTGCTTACCCAATGTATCCATTTGCTATCTTTTCCTGGAGTATTATCGGTATTTTGTGGATTGTTTCCCTTTCTGGGAATGGATTTATCTTCACTGTGACTGTGCTGCAATGGCTCCAGAAGAGGAAGATGCCACCTTGTGACTTCCTCCTGTCTTGTTTGAGTGCCTCCAGATTGCTAACACAGTTTAATTATATGGCCAGCTATTTTTTGCCTTTCTTTTATTCGCCAAGTATAAGAAAAATGTTTTTTTTCTCCAGGGTCTTTCTTCATATGGCCAGTCTTTGGTGTGTCTCCTGGCTCAGCATTTTCTACTGTGTGAAAGTCATCAACTTTTCCAGCTCTTTACTCCTTTGGCTAAAGTTAAGGATCAATCTGCTTGTACCCAAACTACTTGGAATATCAATGGTCATTTTCATGGTCTTTTCTCTTCCTTCCATCTTCACATTTCATAAATTCAATAAACCATGTAATCAGACGATAACACCACCAACCAGCCATGAACCTGAAGATAGCATGTGGATTCGTTTTTTTCCAGTGCAGATAACTTTCACTTGCATAAATTTCAGCATGAACATAGCAGCAACCCTTCTTTTGCTCATCTCTTTGTGGAGACATGTGAGAAACCTCAGAAAGAGTGGAACTAGTGTTCAGGACCTCAACACTCAGGTCCACCTCAAAGTCATGAGGCTTTTGTTCATCACTCTCTTACTCTACCTTTTATTTATTGCTTGTTCGATAACAATGACAACTGGCTTTTTTCATGTTCAAGAAAACCAAGCACTGATTTCAGAGATAATGATTTCCATATTTCCTTCAGTGCATCCCATAATATTAATATGGACCAATCCAAAACTCAAAGATGTGGCTGCTCACATGTTAAACATAAGAGAAAGACCTTAA

>Lizard_T2R11--Intact

ATGTCTCCTCCATTTGGTATCTTCTCCTGGAGCATTCTAGGTATTTTGTGGATTGTTTCCCTTTCAGGGAATGGATTTATCTTCACTGTGACTGTGCGGCAATGGCTCAAGAAGAGGAAGATGCCACCTTCTGAATTCCTTCTGACCTGTTTGAGTGCCTCCAGATTACTAACACTGTTGACTACTATGGTCAGTTATTTGCCTCTCTTTTATTCTTCAGGTAGAAGTGCAATGCTCCTTATTCCCTGGGTCTTTCTCAATATGGCCAGTCTCTGGTGTGTCTCCTGGCTCAGCATTTTCTACTGTGTGAAGGTCATCAACTTTTCCAACTCTTTACTCCTTTGGCTAAAGTTAAGGCTCAATCTGCTTCTACCCAAACTACTTGGAATATCAATGGTCATTTCCATGGTCTCTTTTCTTCCTTTCATCTTCATATTTCATCAATGCAACAAGCCATGTAATCAGACTGTAACACCACCAATCAACCATGAAGATGAAGCTGATGACAGCATGTGGATTCGTTTTATTCCACTGCAGATAACTTTTACTAGTATAAATTTCAGTGTGAACATAGCAGCAACCCTTCTTTTGCTCATCTCCTTGTGGAGGCATGTGAGAAACCTCAGAAAGAATGGTACTAGTGTTCAGGACCTCAACACTCAGGTCCACCTCAAAGTCATGAGGCCTTTGTTGATCACTCTCTTACTCTACCTTTTATATATTGCTAGTTTGATATTAATGCATACTGGCTTTTTTTATTTACAAGCAAACCTATCACTGATTGGAGAGATAATGATTACCATATTTCCTTCAGTGCATGCCATAATACTAATATGGACCAATCCAAAACTCAGAGGAGTGGCTGCTCACATGCTAAACATCAGACAAAGAGCTTAA

>Lizard_T2R12--Intact

ATGGTTAGCAATTCAACTTCTCTACTTGATATCCTTAAATCGACCTATCTGGGAATAGTATCTGTGTTTACTCTTTTAGAAAATGGATTCATCATAGTTGTATTGGGATATCAAGGACTTCAGAAGAGAAATATATTGCCTCATGATATCCTCTTGATAGGTTTGAGCGCCTCCAGACTAATGTCGCAGATGCTGAGTTCCACAAGCTATCTTCTGTATTTCAAGAAAGACATGGGTATTTTTAAACAGGATGTTGTATTTATTTCCTGGAACTTTTTCAACATGACTAGCATGTGGTCTGCAACCTGGCTTAGTGTTTTATACTGTGTGAAGGTCACTAACATTGCCAACTGCCTCTTCCTCTGGCTGAAGCCAAGGATCAACATGCTTGTACTCAGGCTGCTTGCGATGTCAGTAGTCATTTCCAGTATCTTCTTTGTTCCTTCAGTCCTTGAATACTTTCAACAGAAAAAGTGGGACAATCTGACTAGAAACTCACCCGTGAGTGCCAATCAAAGTGAGGGTTATAATAACGAATTCATTAACCTTGATGTGCAACTCTTTTATGTTTCCATAACTTTCTGCATAAGTATAATTGCATCCACGCTTTTGCTTGTCTCATTGTGTAAGCAGATTAGGAATCTGAAGAAGAGTGGCCTAGGTGGCAAAGATCTCAACACTCAGGTCCACAGGGATGTCATTACACTGTTGCTGTCTTACATCTTCTTCTATATTGTACATTTCACTGGTTTCATAATTTTGAAAAATGATGTTTCTAGGCATCGAAGTCTTGAAATGTTAGTTATTCAGATTCTGACAATTTCATTTCCTTGTGTACACAGCATTATGTTAATATTGACTAATCCCAAAGTAAAAGAAATGGCTGGTCATATTCTGAATATTACACAAAGAGCTTCTTAA

>Lizard_T2R13--Intact

ATGGTTAACAATTCAATTTCTCCATTTGATATTCTTAAATGGACCATCCTGGGAATTATATCTGTGTTTACTCTTTTAGGAAATGGATTCATCATAGTTGTACTGGGATATCAAGGGCTTCAAAAGAGAAATATATTGCCTCATGATATCCTCTTGATAGGTTTGAGTGCCTCCAGACTAATGTCGCAGATGCTGAGTTCCACAAGCTATCTTCTGTATTTCGAGAACGACATGGGTATTTTTAACCAGGATGTTGTACTTATTTGCTGGAACTTTTTCAACATGACTAGCATGTGGTCTGCAACCTGGCTTAGTGTTTTATACTGTGTGAAGGTCACTAACTTTGCCAACTGCCTCTTCCTCTGGCTGAAGCCAAGGATCAACATGCTTGTACTCAGGCTGCTTGCGATGTCAGTAGTCATTTCCAGTATCTTCTTTGTTCCTTCAGTCCTTGAATACTTTCAACAGAAAAAGTGGGACAATCTGACTAGAAACTCACCCGTGAGTGCCAATCAAAGTGAGGGTTATAATAATGGATTCATTATCCTTCTTGATATGCAGCTCTTTTATGTTTCCATAACTTTCTGCATAAGTGTAATTGCATCCACGCTTTTGCTTGTCTCACTGTGGAAGCACATTAGGAATCTGAAGAAGAGTGGCCTAGGTGGCAAAGATCTTAGCACTCAGGTCCATATGAATGTCATTACACTATTGCTGTCTTATATAATTTTCTATATTTTACATTTCACTGGTTTCATAATTTTGATAAGTGATGTTTCCAGATTTAGAAGTGTTGCCACGCTAGTTACTTCCATCCTGATAACTTCATTTCCTTGTGTACACAGCATTATGTTAATATTGACTAATCCCAAACTAAAAGTAATGGCTGGTCATATTCTGGGTATCATGCGAAGAGCTTCCTAA

>Lizard_T2R14--Intact

ATGGTTAGCAATTCGACTTCTCCACTTGATATCCTTATCTGGATCATTGTGGGAATAGTTACCATGTTTTCCTTCTTAGGAAATGGATTCATCACAATAGTACAGGGACACCAATGGCTTCAAAATAGAAAGATTTTGCCATGTGATTTTCTTTTGACCAGTCTGAGCACCTCCAGATTTTTGATGCAGTTACTGTCTTCAGTGAACTATTTTCTGTATTTCATCTCTTTAGAGTCCTATATGAATCCCATCAAACAGGCAATTGTATATGTTATCTGGTTGTTTTTTAACATGGTCAGCCTCTGGTCTGCCACGTGGCTAAGTGTTTTCTACTGTGTGAAGATCACTAACTTTGCCAATTGCCTCTTCCTTTGGCTGAAGCCAAGGATCAACGCACTTGTACTCAGGCTGCTTGGAATATCAATAGTCATCTCCAGCATCTCCTCGCTCCCGTCCATCATCGAATATATTGGGCAAAAAAAGGGGGGCAATTTGACGGGAAGTGCCAACCACAGTGAGGCTTATAACCACAGAAATATGCTTCCTCTGCATGTCACTTTTGCTTTCATAAATTTCACCATTAACATAACTGCAACCATTGTTTTGCTCACCTCACTATGGAAGCACACAAGGAATCTGAAGAAGAGTGGTGTTGGTGGCAAAGACTTTAACACTAAGGTCCATTTCAATATCATAATACCATTGCTGTTTTATGTCGTCTTCTACTTTGTTCATATCTCCAGTCAGATAATTGTTTCAAATGAAATTACCATAGTTGGATCAGTGAAACAACGAATTACTGATATCATGGTGTCTACATTCCCAACTGTGCACTCTATTATATTAATATTGACTCATCCTAAACTGAGAGAAACGGTTGTTCGCATTCTGAATATCAAACGAAGAATTTGA

>Lizard_T2R15--Intact

ATGGATTGCAGCTCAATTTCTCCACTTCATATCCTTATGTGGAGCATCGCAATAACTGGAAACATAGTTGCTCTTTTAGGAAATGGATTTATCACAGTTGTTCAAGGTCACCAGTGGCTTCAAAAAAGAAAGATTTTGCCTTGTGATTTCATCTTAATTAATTTAAGTGCCTCCCGATTTATGATGCTGTTGTCGAATTCCGTGAACTATATCTTGTATTCCATCTCCTCGGAGAGTTATCTGCGTTCTTATAAAAAGGCATATCTAATGATTACCTGGACTTTCATGAATATGGCCAGTCTCTGGTCTGCCACATGGCTAAGTATTTTCTACTGTGTGAAGGTTGCTAACTTTACCAACTGCCTCTTTCTCTGGCTGAAGACGAGGATCAACATGCTTGTGCCCAGGCTGCTTGGAATGTCAATAGTCATTTCCAGCATCTTCTCTGTTCCTTCAGTCATTGAATATTTTGGACAAATAAGGGGTGGCAACTTGACGATAATCTTGCCGCTGAATGTCAGTCAAAATGAGCGTTATGCTAAAGGTTTATTTCCTCTGTATCTGACTTATACTTCCATAAATGTCTGCATAAGTATAATTGCATCCAGTCTTTTGCTTGCCTCCTTATGGAAACACACGAGGAATCTGAAAAAAAGTGGTCTAAGTGGTAAAGACCTCAGCACTCAGGTTCACAAGAATGTCATAATAGTGGTATTCTCTTATGTTTTTTTTTACCTTACATTTTTCACTGCTTTAATAATTGAGGTAACTAATGTTTTTAAGCCTCGAAGTCCTGAATCGTTAATAGTTGAAATTTTGGCAACTTCATTTCCTTCTACACACTGCATTGTATTAATATTGACTAATCCCAAACTGAAAGAAATGGCTGCTCGCATTCTGAATATTGGATAA

>Lizard_T2R16--Intact

ATGACTAGCGACAATATAGTTAAAGTTGATTTCCCTGTTTGGATCATTTTTGGAACTCTGTCTCTTATTGGTATTTTGGGAAATGGATTCATCATGGCTGTGAACGGACTTCAGTGGCTTCAAAACAGAAAGATAATCCTTTGTGATTTTCTCCTGACCAGCGCAAGTACCTACAGATTTATCATGCAGTTGACTCTTCTGCTATACAACATCTTTTACTACATCCCAGAGAATATTCACTGTATTTACAGAATTGATCTTCTGTTCTTTTCCTGGATGTTTTCTAATATGATCAGCTACTGGTGTGCCACATGGCTCAGTGTTTTCTACTGTGTGAAAGTCGCCAACTTTGCCAACCCCCTCTTCCTCTGGCTGAAAACAAGGATCAATATGCTTGTACCCAGGCTGCTTGGATTGTCCATAGCAGTTTTCACAGTCTCTTGTCTTCCTTCAATTGTTGATTATTTTGGACAAACAAAGTGGGACAATCTGACAGAAATCTTGCAAGAGAATACCAGCCAAAGAAACATTTGTGACATTCCTCACATGACTTTTCTTCCCATTCAACTTTCATTTTATGTCATAAATTTGTGCCTAAGCACAATTGCAATCATTCTTTTGCTTGCCTCTCTGTGCAAACACATAAGAAATCTCAAGAAAAGTGGTGTTGGTATTAAGGACCTCAGCACTCAGGTCCACATTAAAGTCATGACATTTTTGTTGCTCTGGCTCTTCCTCTACTTTTTAGATTTCATTGGTATGATAATTTATACTAACAACACTGTTAAAACAATAAAATTGGAAGGGGTGCTTATTGACATCTTGATGTCTGCATTTTCTTCCGCTCATCCCATTATATTAATATTAACCAATCCTAAACTGAAAGAAATGTCTGCTTGCATCATTAAAAAAATGTATGCTCGTGTCATAAACATCAGATGTAGCACTTTATAA

>Lizard_T2R17--Intact

ATGGTGAACAATATGTCTACAGTTAAAATCTTTTTTTTGATCATTTTTGAAATTGTGTCCTTTATTGGTATTTTGGGAAATGGATTCATTATAGTTGTGAATGGACACAAGTGGTTCCAAAGCAGGAAGATGATCCCTTCCGATTTCCTCCTGACCAGCCTGAGTACATCCAGGTTTATCATGCAGTTGAGTCTTCTGATAAACTACGTTCTGCTCTTCAGCCTAAAGAATAATTTCCGTTTTGCCGTAGAAGATGTTATGTTCTTTTCCTGGATGTTTTCCAACATGATCAGCCACTGGTGTGCCACAGGGCTCTGTGTTTTCTATTGTGTGAAGGTCGCCAACTTTGCCAACCCCCTCTTCCTCTGGCTGAAAGCAAGGATCAATATGCACCTACCCAGGCTGCTTGGACTTTCCATAGCAATTTTCATGGTTTCTTGTCTTCCTTTCCTTTTTGAATATTTCGGACACAGAAAGTGGTGCAATCTGACAGAAATTCTGCCGGAGAATGCTAGCCAAAGCGAGTTTGGTGACACTCCTGCCATTGTTTTTCTTCCTATGCAATTTTCTTTTTATGTCATAAATTTGTGCCTAAGCACAATTGCATCCATTCTTTTGCTCGTTTCTCTGTGGAGACACACAAGAAATCTCAAGAAAAGTGGTGTTGGTGTTAAGGACCTCAGTACTCAGGTCCACATTAAAGTCATGGCATTTTTGTTGTTCTGGATCTTCTTCTACTTTGCAGATTTAATTGCTCTGATCATTTATGCTGACCTCATTAATAGTATTGGGACAGTTCAAGGACTGCTTTTGGGAATCTCGATGTCTGCATTTCCTTCTGCACACTCCATTATATTAATATTAACCAATCTTAAATTGAAAGAAATGTTTGATTACATCATTAAAAACATATGCTCATATCATAGACATCAGGAACAGAATATGGAAAAAGGGCATTCCCTACAAGACAGGAAAAGACATTCCCTCCCTATTTAA

>Lizard_T2R18--Intact

ATGGATTACAGCTCAATTTCTCCACTTCATATCCTTATATGGAGCATCGCAATAACTGGAAACATAGTTGCCCTTTTAGGAAATGGGTTTATCACAGTTGTTCAAGGTCACCAATGGCTTTTAAAAAGAAAGATTTTGCCTTGTGATTTCCTCTTAATTAATTTAAGTGCCTCCAGATTTATTATGCTGCTGTTAAATTCTGTGAACTATATTATGTATTTCATCTGCTCAGAGAGCTCTCTGATTTCTTATAAAAAGGCATATACATTGATTACCTGGACTTTCATGAATATGGCCAGCCTCTGGTCCGCCACATGGCTAAGTGTTTTCTACTGTGTGAAGGTCGCTAACTTTACCAACTGCTTCTTTCTCTGCCTGAAGCCGAGGATCAACATGCTTGTGCCCAGGCTGCTTGGAATGTCAATAGTCATTTCCAGCATCTTCTCTGTTCCTTCAGTCATTGAATATCTTGGACAAATAAGGGGTGGCAACTTGACTGTAATCTTATCACTGAATGTCAGCCAAAATGAGTCTTATATTAAACCCTTATTTCATCTGCAGCTGACTTATACTTCCATAAATGTCTGCATAAGTATAATTGCATCCAGTCTTTTGCTTGCCTCATTATGGAAACACACAAGGAATCTGAAAAAAAGTGGTCTAGGTAGTAAAGACCTTAGCACTCAGGTTCACAAGAATGTCATAATAGTGGTGTTCTCTTTTGTTTTTTTTTACCTTGCGTATTTCACTGCTTCAATAATTGCAGCAAGTGATGTTTTTAAGCCTCATCGTCCTGAATTTTTAATAGTGGATGTTTTGGCAACTTCATTTCCTTCGACACACTGCATTGTATTAATATTGACTAATCCCAAACTGAAAGAAATGGCTGCTCGCATTCTGAATATCAGATAA

>Lizard_T2R19--Intact

ATGGCTAGCAACTCACCTTCTCCACTTGATATCCTTATCTGGACTATTATAGTAATCGAATACATTGTTTCTTTTCTAGGAAATGGATTTATCATGGTTGTTCATGGACATCAATGGCTTCAAAAAAGAAAGATGTTACCTTATGGTTTCCTCTTAATTAGTTTAAGCACCTCCAGATATATGATGCATCTGCAATCTTCACTGAACTATATTCTGCATATTGCCTTCTCCGAGCCCTGTATCGGTTTTTCTATTACAAAAATTGGAGATGTTAACTGGATCTTCTTCAACATGATCAGTGTTTGGGCTGACACATGGCTAAGTGTTTTATACTGTGTGAAGGTCACTAACTTTGCCAACTGCCTCTTCCTCTGGCTGAAGCCAAGGATCAACAATCTTATACCCAGACTGTTTGGAATGTCAATAGTCATTTCCAGTATATTCTCTGTTCCTTCAGTTATTTACTTTCTAGGACAAAAAAGCGGGGGCAATTTGACTGTAATCTTGCCACTGAATGTCAGCCAAAATGATCCTTGTAGTAAACACTTACTTCATCTGCAGCTGATTTATACTTCCATAACTTTCTGCACTAGTGTTATTGCATCCACTCTTTTGCTTGCCTCACTGTGGAAGCACACAAGGAATCTAAAAAAAAGTAGTCTGGGTGGTAAAGACCTCAGAACTCAAGTCCATATGAATGTCATAATACTGTTGCTGTCTTATGTTTTCTTCTACCTTGTATTTTTTACTGGTTTAATCCTTTTTAAAACTGATGTTATTAATATGACAATTCTCGAGATCTTGGGAACTTCATTTCCTTCTGCACACTCCATTATATTAATATTGACTAATCCCAAACTAAAGGCTGTGGCAGTTCGCATTCTGAATATCAGCCAAAGAGCTTCATAA

>Lizard_T2R2--Intact

ATGGCTTCTCCCAACTATATTGCTTTTTTGGTCATTGAAGTTGTCTTGGTCATCAGCGGGCTCATCTTTAATGGCTTTATTGTTATGGTGATGATCACTCAATGGATTAAATACAGAAGACTAGCATCATGTGAACAGCTCATTCTAAGCCTGGGACTGTCCAATTTGTGGGTAACAATTGTCTTTGGTCTGTTCCACTCTGCTTTCTCACATGAATCTGAATTCAGTTCATTAAATTTCCAAATATGGTACTCCATTTTTTTCTTTTGTATTACATTCAGATATTGGCTCACTGCCTTGCTATGTTTCTTCTACTTCATTAAACTTGTGAACAGTTCCCATGCTTTCTTCCTTTGGTGCAAATTGAGGATATCATGGCTGATACCCCGGTCCCTTATGGGATCTTTGATCATCTCCTTGTTTGTTTTCATTGTGATAGTAAGTAATATGTATACACCTTCTCAAGAAAGCCCTGCAACCAATACTACAACAGTGACCCAGGGAAAGTCACTGAAAGACATTCTCACAAAATTTGATGTGCTCTTTTCAACTATTGGATCTGGTTGTCCTTTCCTTGTGGTGTTCCTATGCTCCATTTTAGTTGTTGTCTCACTCTGTGGGCACGTCTGTCAAATGAGAAGTAAAGAATCCCATCTCAGGAGTTTTCAGGCAAAAGCTCATATCCAGGCAACCTGGACAGTGATCTCTCTGCTATTACTTTTTCTTTCATTTTTTGTGGCACAGACTTTGTCTATGACTGTAGATATAGGATATAATGGAAAATTATTTATTTTCACAGTGATGTCAATATACTCTCCAGCTCAGGCTGTCATTCTGGTGTTGAACAACCCAAAATTAAAGCAGGCCTTAGCCGTGATGGTTCAAAGAACACAGTCTTAA

>Lizard_T2R20--Intact

ATGGATAATAACTTAATTTCTCCATTGGGCATCTTTAGATGGACCATGGTAGGAAGTATATCCATGGTTTCCATTTTAGGAAATGGATTTATCATAATTGTGAGTGGGAACCGATGGCTCCAAAACAGGAAGATGGCCGCTTCTGATTTACTCTTGACTAGTTTGAGTATCTCCAGAATTTTTTTGCATGTAACCTTTGGACTTTACTATGTTTTAGAAGTCAGCATTGGTGACACCTATATGTGTACTTTTGCCTATGATGCTGTCATCTTTGCCTGTATGTTTTCTAGCATGGCCAGCCTCTGGTGTGCTTCATGGCTTAGTGTTTTCTATTGTGTAAAGGTCACCAACTTTGCCAACTGCTTTTTACTTTGGCTGAAGCCAAGGATCAATGTGCTCTCAATTAGACTGCTTGGAATGTCAGTAATTAGTCTTATGGTCATCTTTGTTCCCTTCTTCTGGAGTTACACTGAAGACAAAAAGCGGCGCAATCTGACAGGGAGCCTGCCAGTGAACATCAGCCAAAGAATGGACTGCAAAGCCTTATTTTTTATTTTTTATCCTTTTCAGTTAAGTGTTTTATCCATGAATTTCATCATCACCATAACTGCAAACGTTCTTTTGATCACCTCTTTGTGGAAACACACACAGAATCTGAAAAAGAGTGGTATTGTTGCAAAAGACCTAAGTACTCAGATCCATATTACTATCATGAAACCTTTGTTGTGTTATATTTTGCTCTACCTTTTATTTTTCACAGGTATGCTAATTTTTTTAGGCAGTTTTGCGTACACTTTTAATGGTAAGGGTTTTTTATCTGACATCATTTTTACCACATTTCCTTCAGCACACACCATAATATTAATCTTGACCAACCCAAAACTGAAAGCCCTGTTAATTTGTCATTTAAATATAAGATCAAAGGCTTAA

>Lizard_T2R21--Intact

ATGGTTAGCATTCCAACTTCTCCAGTTGATATCCTTAGATGGACCATTCTGGGAATTGTATCCTTGTTTACTCTTTTAGGAAATGGATTCATCATAGTTGTACTGGGATATCAAGGGCTTCAAAAGAAAAATATATTGCCTCATGATATCCTCTTGATAGGTTTGAGCGCCTCCAGGATAATGTTGCAGTTGCTGAGTTCAGCAAACTATATTTTGTGTTTCATCTCAGAGACCTACAGGGATACTTACAAACAGGATGTTGTACTTCTTTCCTGGAACGTTTTCAACATGACTAACTTGTGGTCTTCAACCTGGCTAAGTGTTTTATACTGTGTGAAGGTCACTAACTTTGCCAATTGCCTCTTCCTCTGGCTGAAGCCAAGGATCAACATGCTTGTACTCAGGCTGCTTGGGATGTCAATAGTGATTTCCATTATCTTCTCTGTTCCTTCGGTCATTAAATACCTTCAACAGAAAAAGTGGGACAATCTGACTAGAAACTTGTCAGTGAGTGCCATCCAATGTATGGATTATAAGAACAGATTCATAATTTTTCTGGATATGCAGCTCTTTTATGTTTCCATAACTTTCTGCATAAGTTTAATTGCCTCCACTCTTTTGCTTGTCTCACTGTGGAAGCACATTAGGAATCTGAAGAAGAGTGGCCTAGGTGGCAAAGATCTCAGCACTCAGGTCCATATAAATGTCATTACACTGTTGCTGTCTTATATCTTCTTCTATCTTTTATATTTCACTGGTTTCATAATTTTGGGAACTAATGTTTTCAACTATGAAAGTCTTGAAAGGTTAATTTTTAAGTTCCTGGCAATTTCATTTCCTTGTGTACACTGCATTATGTTAATTTTGACTAATCCCAAACTAAAAGAAATGGCTGGTCATATTCTGAATATCACACGAAGAGCTTCCTAA

>Lizard_T2R22--Intact

ATGATTAGTGATAATATCGTTAATATCCTTTCTTGGGTCATTTTTGGAACTGTGTCTCTTATTGGTATTTTGGGAAATGGATTCATTTTGGTTGTGAACGGACTCCATTGGCTTCAAAACAGAAAGATGATCCTTTCTGATTTTCTCCTGACCAGCGTGAGTACCTTCAGATTTATCATGCAGTTGGATATAGTGCTATTCAACATCCTGCACTACTTCCCAGAGAACATTCCCTGTATTTACAGAATAGATCTTATGTTCTTTTTCTGGGTGTTTCCCAAAATGATCAGCAACTGGTGTGCCACATGGCTCAGTGTTTTCTATTGTGTAAAGGTCACCAACTTTTCCAACCCTCTCTTCCTCTGGCTGAAAGCAAGGATCAATATGCTTGTACCCAGACTGTTTGGACTGTCCATATCTGTTTTCATGGTCACTTGTCTTCCTTTACTTGTTGATTATTTTGAACAGACAAAGTACTGCAATCTGACAGAAACTCTGCCAGAGAATGCCAGTCAAAGAGAGATTTGTGGCACTCCTGGCATGACTTTTCTTCCCATTCAACTTTCTTTTTATGTCATAAATTTGTGCCTAAGCACAATTGCAATCATTCTTTTGCTTGCCTCTCTGTGGAAACACACAAGAAATCTCAAGAAAAGTGGTGTTGATGCTAAGGACCTTAGCACTCAGGTCCACATTAAAGTCATGACATTTTTGTTGCTCTGGCTCTTCTTCTACCTTTTAGATTTCATTGGTCTGATAGTTTATACTAACATCATTCTTAATACTTTAAAATTGAATGGACAGCTTGTTGACATCTTAATGTCTGCATTTTCTTCTGCTCACCCCATTATATTAATATTAAGCATTCCTAAACTGAAAGAAACATCTGCTTGCATCATTAAAAAAATCTATGCTCATATCATAAACATCAGGCACAGCACTTTATAA

>Lizard_T2R23--Intact

ATGGATTGCAGCTCAATTTCTCCACTTCATATCCTTATGTGGAGCATCACAGTAATTGAAAACATAGTTGCTCTCTTAGGAAACGGATTTATCACAGTTGTTCAAAGTCACCAGTGGCTTCAAAAAAGAAAGATTTTGCCTTGTGATTTCATCTTAATTAATTTAAGTGCCTCCCGATTTATGATGATGTTGTTGACTTCCGTGCACTATATCCTGTATTCCATCTCCTCGGAGAGTTATCTGCGTTCTTATGAAAAGGCATATCTAATGATTACCTGGACTTTCATGAATATGGCCAGTCTCTGGTCTGCCACATGGCTAAGTATTTTCTACTGTGTGAAGGTCGCTAACTTTACCAACTGCCTCTTTCTCTGGCTGAAGACGAGAATCAACATGCTTGTGCCCAGGCTGCTTGGAATGTCAATAGTCATTTCCAGCATCTTCTCTGTTCCTTCAGTCATTGAATATCTTGGACAAATAAGGGGTGGCAACTTGACGATAATCTTGCCGCTGAATGTCAGTCAAAATGAGCATTATACTAAACGTTTACTTCCTCTGCATCTGACTTATACTTCCATAAATGTCTGCATAAGTATAATTGCATCCAGTCTTTTGCTTGCCTCATTATGGAAACACACGAGGAATCTGAAAAAAAGTGGTCTTGGTGGTAAAGACCTCAGCACTCAGGTTCACAAGAATGTCATAATAGTGGTAGTCTCTTATGTTTTTTTTTACCTTGCATTTTCCACCTCTCTAATAATTGAGGTAACTAATGTTTTTAAGCCTCAAAGTCCTGAAACGTTAATAGTTGAAATTTTGTCAACTTCATTTCCTTCTACACACTGCATTGTATTAATATTGACTAATCCCAAACTGAAAGAAATGGCTGCTCGCATTCTGAATATTGGTTAA

>Lizard_T2R24--Intact

ATGCTTTCACCTCAGTTCATTTTCTTCATTCTTGCTGTTATTGACTTGATGCTTGGTGGACTCATCTCCAATTGCTTTATAATTACTGTGATTCTCAGAGAATGGACCACAAGCAGAAGCCTTGCCTCCACTGAACAATTCTTTCTGAGTCTTTCTTTGACCAATTTAGGGGCGACTGTGGTACTGATTCCAAGTTACATCAATGCCTACATCTTCCCCATATTCACAAGAAATTTCATCATGCTAATCGTGTACCCTTTGGATGATTTTCTTGTCCTCTCCAGACATTGGTTCACTGCTTGGCTCATTGTCTTCTATTGTATCAAGATTGTAAACAGCACCCATTCCCTTTTTCTTTGGTGCAAACTGAAGATATCCTGGTTAGTACCATGGCTTATTGCAGGATCTCTGGTTGTTTCCTTGTTTTTTGCCCTTTTTAAATTATATATTATTCTTATGAAAATCCAAAGCAATACAACAATGATTGATATAGAAACGAATGAAGAAATGTCAGGGTATCATACTATTGGTGTTCATGAAATCTTGGTTTTAATTGTTGGCTCTGGTTCATCCCTTCTCATAGTTTTGGTTTGCTCCATCCTAATTTTGGCCTCACTCTGTAAGCATGTCTACCGGTTGAAGTGTAAAGAACACCATTCCAGAAGCATCCAAACTAAAGCTCATGTCAAGGCATCTGGGACTATACTCTTCAGCCTGTTTCTTTATATTTCATTTTATGTGGTGCAGACCTTGGTTATGACTGCAAATGTAGGGAAAATCGAAGGGACCTTTCTCACAATAGTGGTGATTGCATATCCTTCTGCTCAGGCTTGTATCCTGCTGCTGGTGAACCGTAAATTTAACCAGGCAGCCACTCAGATCCTTCCAAGATGCGACACCTAA

>Lizard_T2R25--Intact

ATGTCTTCTTTTCAGTTCATCCTCTTCATTCTAACTCTTGTTGATTTGGCTCTCGGTGGACTCATCTCCAATGGCTTTATACTTACAGTGATTCTCAGGGAATGGAAGAAAAGCAGAAGCCTTGGTTCCAGTGAACAGTTTCTTCTGGGCTTAGTTTTGTCCAATTTGTGGGCATCTGTGATACTGATCCCAGTTTACATCAACGATTATATTATTCCCATATTCCCCAGGAATTTTGGAAAACAAATAATGTACCCTTTAGGTGATTTTCTTGTCATCTCCAGACATTGGTTCACTGCTTGGCTCTGTGTCTTCTATTGCATCAAGATTGTGAACAGCACCCATTCCTTCTTTCTTTGGTGCAAACTGAGGATATCCTGGCTAGTACCGCGATTTATTGCAGGGTCTCTGGTTGTTTCCTTGTTTTTTGGCTTATTTATGTCATTTTTTACTTTTAGATATATCCAAAGCAATATAACAACGATTGATACAAAAAGGAATGAAGAAATGTTTCACTATCGTAAAATTGATGTCCCTGAAATATTATTTTTAATTGTTGGCTCTGGTCCACCTCTTCTCATGATCTTAGGTTGCTCCATTCTAGTTGTTGTCTCACTCTGCAGACACATGTACCGGATGAAGTATAAAGAACATTTTTCTAAGAACCTCCAAATTATAGCTCATGTCAAGGCAGCTGTGATTATTCTCTCAATTCTATTCCTTTATCTTATATTTTATGTGGTGCAAACCTTTTCTCTGATACTAACTATGGAAAAAATGGAAGGGATCTTTGTTACAATATCAATTATTGTATATCCCTGTGCTCAGGCTTACATCCTGCTGCTAGGTAACCCCAAGTTAAACCAGGCAGCTGCACAGGTGTTTCCAAGAAGAGAAACCTAA

>Lizard_T2R26--Intact

ATGATCAAGAGGAGAGCAGTCTTCTTCCCAGTCATAATCTTTGTGGTAGTTCTAGCTGATTTTGTTGTTGGTGGGCTCATCTCCAATGGATTTATAGTCAAAGTTATTATTAATGAATGGATTTTATACAAGTGCTTCACTGCCAATGAACAGCTCCTTCTGAGTCTGAGTATATCCAATTTCACCATCACAATTTTAATGCTCATATCGTTTTTTCTGCAAAACCAAGTGAACAGCTATACAAGGATTCAAACATCATTTTCTTATTCTGTCGTAGTCTTCAGATCCTGGCTCACTGTCTGGCTTTCTGTCTTTTATTGCATCAAGATTACAACCAGCACTCATTTCCTCTTCCTTTGGTGCAAATTGAGAATATCATGGCTGATACCCCGGCTTCTCGTGGGATCTGCAATCATCTCATTCGTTTCCATATATGCCTTTCGTGATATTTTAATACCATCCCACAGCAACACAACTGCCACAAATACGAGTATGATCCAAGAATGGAGACTACCACAGCTTGTTGATTCTTTCAAAGTCTTCTTTTTATCTGCTGGTTCTGCTTGTCCTGTTCTCATGATGGTACTTTTCTCCACTGTAGCTGTTGGTTCACTTTGCAGGCACATCTGTCGGATGACCGGGAAGGAATCTAGATTCAGGAACCCCCAAACGGAAGCTCATATCAAGGCAGCTGGGACAGTGATCTTGCTCCTGCTCTTTCATGTGTCGTTTTATATGGCAGAAACCATTTCTTTGACTATAAACATTAGAACAAAACATGACATCTTTGTGGTTGTTCTAACGGTGGTTTATCCGCCTGCTCAGGCTGCCATCTTGGTACTGGTTAACCCAAAACTAAAGCAGGCAGCTACTCACATACTTCTAAAAATATCTCAAGAACAAACTAGACATACTGTTTGTTCACATATTTAA

>Lizard_T2R27--Intact

ATGTCTTCTTTTCAGTTCATCCTCCTCATTTTAGCTCTTGTTGACTTGGCTCTCGGTGGACTCATCTCCAATGGCTTTCTACTTACAGTGATTCTCAGGGAATGGAACAAAAGCAGAAGCCTTGATTCCAGTGAACAGCTTCTTCTGAGCCTGGTTCTGACCAATTTGTGGGCATCTGTGATATTGATCCCAGTTTACATCAATGATTATATTATCCCCATATACCCCAGGAATTTTGGAAAACAAATAATGTACCCTTTAGGTGATTTTCTTGTCATCTCCAGACATTGGTTCACTGCTTGGCTCTGTGTCTTTTATTGCATCAAGCTTGTGAACAGCACTCATTCCTTCTTCCTTTGGTGCAAACTGAGGATATCCTGGCTAGTACCGCGATTTATTGCAGGGTCTCTGGTTGTTTCCTTGTTTTTTGGCTTTTTAATGGCATTTTTAAATTATAGAAATATCCAAAGCAATACAACAATGACTGACATAAAAAGGAAAGAAGACACGTTTCGCTATCGTAGCATTGATGTTCCTCAAATATTATTTTTAATTGTTGGCTCTGGTCCACCTCTTCTCATGATTTCAGGTTGCTCCATCCTAGTTGTTGTCTCACTCTGCAGACACATGTACCGGATGAAGTGTAAAGAACATTTTTCTAAGAACCTCCAAATTAAAGCTCACATCAAGGCAGCTGGGATTATACTCTCAATTCTATTCCTTTATCTTATATTTTATGTGGTGCAAACCTTTTCTCTGCTACTAACTATGAAAAAAATGGAAGGGATTTTTGTTACAGTACTAATTATTGTATATCCCTGTGCTCAGGCTTACATCCTGCTGCTAGGTAACCCCAAGTTAAACCAGGCAGCTGCACAGGTGTTTCCAAGAAGAGAAACCTAA

>Lizard_T2R28--Intact

ATGTCTTCTCCCAAGACTATTGCCTTATTGGTAACTGAAGCTGTCTTGGACATCAGTGGGCTCATCTGTAATGGCTTTATCGTTATTGTGATCATTCAGTGGACTAAATGCAGGACCCTTGCATCCATTGAACAGCTTCTTCTAAGCCTGGCTCTGTCCAATGGGTGCGTGACAATTGTCATTGGTGTATTCCACTTTGGTTTTGCATCTGAAGATAATTTCAGTTCTTTAATTTTTGAGATATGGTACTCCTTTTTTTTCTTTGCTGTGATATTCAGATATTGGCTCACTGCACTGCTATGTTTTTTCTACTGCATCAAGATTGTGAGCAGTACCCACACTTTCTTCCTTTGGTGCAAACTGAGGATGTCATGGCTAATACCCCGACTTCTGGTGGGATCTGTTATTATCGCTTTGTTAGCTTTCATTGTGTCATTAAGTACTATGCATATATCACCGCAAGAAAGCCCAGTGGCCAATGATACAATGCTTACCCGAGGAAAATTACTGAAAGAAACCATCACAAGTTTTGATATGTTCTTTTCAGCTGTTGGATCTGGTTGTCCTTTTCTTGTGGTTTTATTGTGTTCCATTTTAGTTGTTGCCTCACTCTGTGGGCACATCTGTCAAATGTCGGGTAAAGAACCCCATCTCAGGAGTTTTCAGACAAAAGCTCATATCAAGGCAGCTGGGACAGTGCTATCTCTGCTATTGCTTTATCTTTTATTTTTTTATGTACAGACTTTTTCTATGACTGAAAATATAGAATACAGTGAAGAATTTATTTTGGCAGTGATGGCTGTATATTCTCCAGCTCAAGCTGCCATTCTGGTGTTGAATAACCCCAAATTAAAGAAAGGATTATCCTTGATGGTTCAAAGAATCAAGCTTTAA

>Lizard_T2R29--Intact

ATGTTCTCTCTCGCAACCATTGCCTTTGCGGTAGCTGCAGTTGTCTTTGCTCTCAGTGGATTCCTCTCCAATGGCTTTATAGCTGCAGTAATACTCAGGGAATGGACCAAATCCAGGAGCCTTGCTTCCAATGAACAACTCCTTCTGAGCTTGGCTGCATCCAATTTCTGGGCCACAGCATTGCTGAGTCCATTTTACATCAATGCCACCTTAAGGGATTACAGCATCAGCGAGATTTTCCTGTTGCCAGGACTCTACCTTCTTGCTACTTTTGTCATAATGTCCAGATTCTGGTTCACTGCCTGGCTATGCTTCTTCTATTGCATCAAGATAGTAAACAGCACCCACTTTCTCTTCCTCTGGTGCAAACTGAGGATACTGTGGCTAATACCCAGGTTTCTAGCAGGATCTCTGTTCTGCTCTTTTCTTTTTTCTTTGTTTGTGTTACAGATTACTTCCAGACAAGCCAAAAGCAACATAACAGTGAATATTACAAACACAACTGAAGTGAAGTCACTGAAGCATACAGTCAATACCTTTGAAGCCTTCTTTTTAGCTGTTGGTTCTGGTTGTCCTCTTCTTGTGGTTCTACTTTGCTCCATCCTAGTCGTTGCCTCACTTTGCAGGCATGTCTGCCGGATTGCAGGTAAAGACTCTCATGGCAGGAATCTCCAAACCGAAGCTCACATCAAAGCAGCCTGGACAGTGCTCTCCCTCCTGCTCCTTTACGTATCATATTATGCAGCACAGACACTGTCTATTGTTGTGACATTGGGAAAAGATGACGGGACCTTGGTGGCAATGGTGAGAATGGTGTACCCCTCTGCCCAGGCTTCCATCTTGATGCTGGTTAACCCCAAGCTAAAGCAAGCAGCTATGCAGATGCTTCAAAGAGCCAAGGTGTGA

>Lizard_T2R3--Intact

ATGTTTTCTCCTGGGTACATTGCTTTTTTGGTAACTGCAGCTGTCTTGAACATCAGCGGGTTCATCTCTAATGGCTTTATTGTTACTGTGATGATCACTGCATGGACTAAAAGCAGGAGACTTGCATCCAGTGAACAGCTCCTTCTAAGCCTGGGTCTGTCCAATTTGTGGGTGACAATTGTCCTGATTGTGTTCTGCTTCGGTTTTGCAACTTTAACCAATTTCAATGATCAAATTTTCCTTTTCTCCTTTTTCAGCTTTGCTGTGGTAGTCAGGTATTGGCTCACTGTCTTGCTATGTTTTTTCTACTGCATCAAGATTGTGAACAGTACCCACACTTTCTTCCTTTGGTGCAAACTGAGGATATCATGGTTAATACCCCGACTTCTGGTGGGATCTATTATTATCACCTTGTTAGCTTTCGTTATGATTTTAAGTTTTATGTATATACTACCACCCCCGGCCAATGTTACAACAGTGATCCATGCAATGTCACATAGTGAAAGCATCAAGAGTTTAATTGTGTTCTTTTTAACTGTTGGATCTGGTTGTCCTTTCCTTTTGGTTTTATTATGCTCCATTTTAGTTGTTGCATCACTCTGTGGACACGTCTGTCAAATGACAGGTAAAGAATCCCATCTCAGGAGTTTCCAGACAAAAGCTCATGTTCAGGCAGCTCGGACAGTGCTCTCTCTGCTATTGCTTTTTCTTTCATTTTTTGTGGCACAGACTTTGTCTATGACTGTAGATATAGGATATAATGAAAGGTTATTTATTTTCACAGTGATGACAATATACTCTCCTGCTCAGGCTGCCATTCTGGTGTTGAATAACCCCAAATTAAAGCAGGCCTTAGCCGTGATGGTTCAAAGAACAGTCTTAATATGTGAGGAGAAAAATTGA

>Lizard_T2R30--Intact

ATGTTTTCTCTCCAAATTACTGCCTTTCTCGTAGTTGCAGCTGACTTGACTCTGGGTGGACTCATTTCCAATGGCTTTATAGCTACAGTGATTATACGAAAATGGATCAAATGCAGAAGCCTTGCTTCCAGTGAACAGCTCCTTCTGGTTCTGGGAATATCCAACGTTTTTGCCATCATTTTACAGACTGCATCTGTAATTGGTGAAAATGTGTTTATCTGCAGCGACCAGTTGATATTACCAATAATCTTTTTCTTTGTTTTCTTTGTCACATTCTTCAGATTTTGGCTTACTGCCTGGCTCTCTCTCTTCTATTGCATCAAGATAGTGAACAGCACCCATGTCCTGTTGGTTTGGTGCAAGATGAGGATATCTTGGCTAATACACCGTCTCCTGTTAGGATCCCTACTCATTTCCTTGTTCATTTCCTTTTTTGCATTTCATGAGTTTCTTTTTGAATTCCAGAGCAATAGAACAGCCAGCGTTGCAAACAGGACTCAAGAACAGACATTAAGAAAGACTGTTGATTATTTCAAAGTTTTATTTTTGGCTATCGGTACTTCTTGTCCCCTTCTTGTGGTTTTATTTTGTTCCATCTTGTCTATTGTGTCACTCTGCAGACATATCCACAGGATGACAAGAGAAAAGTCAAGTTTTAGGAGCATCCAAGCAGAAGCTCATCTCAAGGCAGCTCAGACAATGCTCTCTCTCTTATTCTTTTATGTACTGTTTTATGTGGGGGAAACATTAAGTATGACCATACATTTTGAGAATGGGAAACAAATTTCTGCTATATTTGTGGTGCTGCTGTACTCTCATGCTCAGGCTGCCATTCTGGTGCTGGTAAATTCCAAGCTAAAGAGGACAGCTACCCAGATTCTTCTAAGAATTTCTCAGGAACTATGCAGACATACTGTTTGTTCACAGATTTAA

>Lizard_T2R31--Intact

ATGTCTTCACCTCAGCTCATTTCCTTCATTCTGGCTCTTGTTGACTTGGCTCTCGGTGGATTCATCTCCAATGGCTTTATACTTACAGTGATTCTCAGGGTATGGAACAAAAGCAGAAGTCTTGATTCCAGTGAACAGCTTCTTCTGAGCCTGGTTCTGACCAATTTGTGGGCAACTGTCTTAGTCATTCTCACTTGCATCAATGACTACATCATCCCCATGTTTCCCAAGAGTTTAATGTACTCTTTAAATGATTTTATTATCATCTGCAGACATTGGTTCACTGCTTGCCTCTGTGTCTTCTATTACATCAAGATTGTGAATAGCACTCATTCCCTCTTCCTTTGGTGCAAACTGAGGATATCCTGGCTAGTACCACGACTTATTGCAGGATCTCTGGTTGTTTCATTGTTTCTTGTGTTATTTATGTCATTTTTTACTCTTATAAATATCCAGAGAAACACAACATTAATTGGGACCCAAATGAATGAAGAAATCTCACAGCATCATAACACTGGTATTCATGAAATCTTGTTTTTAATTGTTGGCTCTGGTTCACCCCTTCTTGTGATCTTAGTTTGCTGCATTCTGGTTGTTGCCTCACTCTGTAAGCACGTCTACCGGATGAAGAGTAAAGAACATAATTCTAGGAGTATCCAAACTAAAGCTCATATCAAAGCAACTGGAGTGGTGCTCTGCATCCTTCTCCTTTATCTGTTGTTTTATGTGGCGCAGACCTTTTCTCTGATTGTAATTAAGGGAAAAATTGAAATAATCTTGGTCACAACAACAGTGTATGTGTACTCTTGTGCTCAGGCCTATGTTCTGCTGCTGGTTAACCCCAACTTAAACCAGGCAGCTATTCAGGTACTTCCAAGAAGAGAAACCTAA

>Lizard_T2R32--Intact

ATGTCTTCACCTCAGATCATTCTCTTCATTCTGGGTCTTGTTGATTTGGCTCTTAGTGGACTCATCTCCAATGGCTTTATACTTACAGTGATTCTCAGGGAATGGAACAAAAGCAGAAGCCTCGCTTCCATTGAACAGCTCATTCTGAGCCTGGTTCTGTCCAACCTAGGAGCAACTCTGCTAGTGCTTCCAATGTTCATCAATGACTATATCTTCCCAATTTTCACAACGAATATTACATATCTAATAATGTACCCTTTAAGTGATTATTTGATCCTTTTCAGACATTGGTTCACTGCTTGGCTCTGTTTCTTCTATTGCTTCAAGATCGTCAAGAGTACCCATTCGCTCTTCCTTTGGTTCAAACTGAAGACATCATGGCTAGTACCACAACTTATTGCAGGATCTTTGGTTGTTTCCTTGTTTATTGCCCTTCCAATGTTTTTCTTGGTCCTTACAGATTTCCTAAGCAACATGACAATGAATAATACAAAAATTAGTACAGAAATGTTACGGAATCGTACTGTTAAGGCGCCTGAAATCTTTTTTTTAATTGCTGGCTCTGGTTCACCCCTTCTTGTGATCTTAGTTTGCTCTATCCTAGTTGTGGCCTCACTCTGTAAACATGTCTACCGGATGAAGTGTAAACAGCACCATTCCGGGAGCATCCAAACAAAAGCTCATGTCAAGGCAGCTGGGACTGTGTTCTCCATCCTGTTCCTTTACCTTTCGTTTTATATGGTGCAGACCTTGTCTATGACTGTAACTGTGGGAAAAATGGAAGGGACCTTTCTCACAGTAGTTGTGATTGCATATCCCTCTGCTCAGGCTTATATACTGTTACTGGTTAATCCCAAATTAAACCAGGCAGTTAATCAGATGCTTCCAAGAAGAGTGACCTAA

>Lizard_T2R33--Intact

ATGTTTTCTCCTGGGGACATTGCTTTTTTGGTAATTGCAGCTGTCTTGAACATCAGCGGGTTCATCTCTAATGGCTTTATTGTTACCGTGATGATCACTGCATGGACTAAATGCAGGAGACTTGCATCCAATGAACAGCTCTTTCTAAGCCTAGGCCTGTCCAATGTGTGTGTGACAATTGTCTTTAGTATGTACTGCTTCAGTTTTACAACTTTATCCAATTACAATGTAAACATTTTCCAGACATGGTTCTCCTTTTTTTCCTCTGTTGTGATATTCAGATATTGGCTCACTGTCTTGCTATGTTTCTTCTACTGCATCAAGATTGTGAACAGTACCCACACTTTCTCCCTTTGGTGCAAACTGAGGATATCATGGCTGATACCCCGACTTCTGATGGGATCTATTATTATCTCCTTGTTTGTTTTAATTATGGCTTTAAGTTTTATGTATATATTATCACCACCGGCCAATGCTACAACTGTGATCCAAGGGATGTCACATAATGAAAGTGTCAACATTTTAATTTTGTTCTTTTTAACTGTTGGATCTGGTTGTCCCTTCCTTTTGGTTTTATTGTGTTCCATTTTAGTTGTTGCCTCACTCTGTCGACATGTCTGTCAAATGACAGGTAAAGAATCCCATCTCAGGAGTTTTCAGACAAAAGCTCATATCCAGGCAGCTCGGACAGTGCTCTCTCTGCTATTGCTTTTTCTTTCATTTTATGTGGCACAGACTTTGTCTATGACCTTATTGAAGAGCCAATTATTTCTTCCAGGAGTGATGATGGTATACTCTCCCGCTCAGGCTGTCATTCTGGTGTTGAATAACCCCAAATTAAAGCAGGCCTTAGCCGTGATGGTTCAAAGAACAGTCTTAATATGTGAGGAGAAAAATTGA

>Lizard_T2R34--Intact

ATGGATAATAACTTAATTTCTCCATTGGGTATCTTTACATGGACCATTGTAGAAAGTATATCCATAGTTGCCATTTTAGGAAATGGATTTATCATAGTTGTGTGTGGGACCCGATGGCTCCAAGCCAGGAAGATGATCCCTTCTGATTTTCTCTTGACTAGTTTGAGTATTTCCAGAGTTTTTCTGCATTTAACCTTTGGACTTAGCTATATCTTGGAGGTCAGCATTGGTGACATCTTTATGTATTCTTCTGCACAGGAAGCTATCAACTTTATCGGCATGTTTTCTAGCATGGCCAGCCTCTGGTGTGCTTCATGGCTTAATGTTTTCTACTGTGTGAAGGTCACCAACTTTGCCAACCGTTTTTTACTCTGGTTGAAGCCAAGGATCAATGTGCTCTCAGTTAGACTGCTTGGAATGTCAATATGTAGTCTTGTGGTCATGTCCGTTCCCTTCTTCCGGAGTTACGCTGAAGAAAAAGAGTGGTGCAATCTGACAAGGAATCTGCAAGTGAATGTCAGCAAAATAAAGGCTTGCAAAGACTTAGTTTTAATTTTTCGTCGTTTTCAGTTAATTGTTGTTTCAATGAATTTCATCATCAGCATGATTGCAACCATTCTTTTGCTCACCTCTTTGTGGATACACATAAGGAATATGAAAAGGAGTGGTATTGGTGCAAAAGACCTAAGTGCTCAGGTCCATATTGATGTCATGAAGCCTTTGGTGTTTTATATTTTCTTCTACCTTTTATATTTTGCTGGTATGCTAAATCTTGCAAGTGGTTATGTGTCCATTTTTGATCCTATGGAGCTTTTATCTGACGTCCTTCTTACCATTTTTCCTGCAGCACACACCATAATATTACTTTTGAGCAATCCAAAACTGAAAGCATTGTTAGTTCGCACTCTAAATACAAGACAAAAGGTTGATCAAGAAAAGGGGCACCAGACATGCATTTCATGTCTCCAAGGATGA

>Lizard_T2R35--Intact

ATGGATAATAACTTAATTTCTCCATTGGGTATTTTTACATGGACCATTATAGAAGGTATATCCATGGTTGCCATTTTAGGAAATGGATTTATCATAGTTGTGAGTGGGAACCGATGGCTCCAAACCAGGAAGATGGTCCCTTCTGATTTTCTCTTGACTAGTTTGAGCATCTCCAGAGTGTTTTGGCATGTAACCTTTGGACTTAGCTATGTTTTGGAGGTCAGCATTGGTGACATCTTTATGTATTCTTCTGCACAGGAAGCTATCGACTTTATCAGCACGTTTTCTAGCATGGCCAGCCTCTGGTGTGCTTCATGGCTTAGTGTTTTCTACTGTGTGAAGGTCACCAACTTTGCCAACCGCTTTTTACTCTGGCTGAAGCCAAGGATCAATGTGCTCTCAGTTAGACTGCTTGGAATGTCAATAAGTAGTCTTGTGTTCATGTCTGTCCCCTTCTTCCAGCATTACGCTGAAGCAAAAAAGCGGTGCAATCTGACTGGGAGCCTGCCACTGAACACCAGCCAAAGAAACGATTGCAAATTCTTACTTTTAATTTTTCGTCATTTTCAGGTAATTGTTGCTACCATGAATTTCGTCATCAGCATAACTGCAACCATTCTTTTGCTCACCTCTCTGTGGAAACACACCAGGAATCTGAAAAAGAGTGGTATTGATGCAAAAGACCTAAGTGCTCAGATCCATATTAATGTCATGAAGCCGTTGGTGCTTTATATTTTCCTCTACCTTTCATATTTTGCTGGTATTCTAAATTTTGCAAGTCATTCTGTGCACAATGTTGATGCTGTGGAGCTTTTGTCTGATGTCCTTCGTACCATATTTCCTGCAGCACATACCATAACATTAGTTTTGAGCAATCCAAAACTGAAAGCACTGTTAGTTCGTACTCTAAATATAAGACAAAAGGTTAATCTTGTCTCCAAGGACGAAAAGACAAACCAAATTTCCAAATGTTGA

>Lizard_T2R36--Intact

ATGGGGACTGTAAATTCTCTTCAAACCACCTTTCAGGTTGTTATCATGACTTGCTGTGTTGTGGGGATAATAGTCAATGTATTTATTTTGGTCATTGGCTACCTAGACTGGGCAACAAGGAAAAAACTGCCCAATTGTTACATGATTGTAAGTTGTCTCAGTTCATCTAGACTTCTTCTGCAAGGGACAGTATTGTACTCTACGCTTTGCCAAGGGACACACCACTGGAATGTACTCGTGTTGTCCAGCACAGCTTGCCTTTGGTTTGCTGCTTGCCTCAGTGCCTTCTACTGTGTGAAGATTGCCACTTTCACCCAGCGGCATTTCCTGCTAATGAAGCTGAGAATCTCTGGGATGGTTCCATATCTTCTTCTGGGATCAATGTTGGTCTCTTTGATCTCTTCTGTCCCTTTCATCTGGACAGATTTTAGTGTTCACCTCTGTAACTCAACCAGGAGTTCTGTGAAAAACACCACCGGTGACAGCACCGTTGGGAACATCTCCTACTTCAAGGTTTTCATCTTGTACCTGACTTGGATGACCATTCCTCTTCTGTTCTTCACAGCATCATCCACCCTCCTAATTGCATCTCTCTGGAGACACACCAAACAGATGAGACGCAACATGACTGGCTTCAAGGATCTGAGCACACAGGTTCACAGCAATGCCATTAAGACTCTGATCTCCTTCCTAGTGCTTTATTTCTGCAGCTTTGTAGCTGAGATTCTGCTGGGGATCCCTTCCTGCCTAACTAGGAATAGATGGAAACACAGTATCTGTTCATTCATTGTTGCTGTATGCCCCTCCATCCATTCCATTCTTTTGATTTTCTTTAATGTTAGGCTGAAGCAGACATTCAAAGGCATTCTGCTTTCCATGAGGTGCCATGGGGGAAAAGCCTGTTCCTAA

>Lizard_T2R37--Intact

ATGGCTCTCATAATGTCTCCACTTGGCGTTCTTGCTTGGGCTACTTTTGGAATTTTGAATGTCATGGCCCTTTTAGGAAATGGATTTATCATAGTGGTGAATGGACATCAGTGGCTCCAGAGCAAGAAGATGATCCCTTACAAGTTCCTCTTGACTACTTTGAGCACCTCCAGGTTTCTGTTGCAGATGGATTCCGTAGTGGGCCATTTTATGTATCTTATCTTTGCAGAGATCCAGAAAGAGACCCATCTATATGCTTCCAGAGCAGAGGTTGTGAATTTTATCTGGATGTTCTTGAACATGGTCAGCCTCTGGAATGCCTCGTGGCTCAGTATGTTGTATTGTGTGAAGGTTACCAATTTTGCCAACAGGCTCTTCATCTGGCTGAAGGCAAGGGTCAATATGCTTGTACCCAGACTACTTGGAATCTCAATAATAGTATCTACAGTTTTCTTTTTTCCATCAGCAGCCAAGTATTATAGAAAGAAAAAGTGGTGCAATTTAACAGATGCCGTCCCGAGGAACAGTAGCCAAAGGGAGGGTTGTAATGATGCTTTCGATGTTTTTCATTTTCCGCAGTTATTTCTTGCTTCAGTTAATTTCGGCTTGACCCTCACTGCATCCTGTCTTTTGCTCACTTCTCTGTGGAAACACACAAATAATCTGGAAAAAAGTGGTGCTGCTTTTAAGGATCTTAGCACTCAGCTCCATTTCAAAGTTATGATGCCTTTGCTGGTCTCTTTATTGTTCTATGTTTTATATTTTCCTTGTTTCGTGTTAGCTGTAGGTGATATTTTTGAATTTGGAAGACTTGAGCGATGGGCTTCTGAGATAGTACTGCCTCTGTATGCTTTCGTACAGTCCATAATATTAATACTGACCAATCCTGAACTAAAAAAAGTAGCTGCTAGCATTCTAATAATCAGACAACGAGCTTCATGA

>Lizard_T2R4--Intact

ATGGCTGCTAACCAAATGCCTCCATATGCTATCATCTCCTGGTGCATTATAGGCATTTTGTGCATTGTTTCCCTTTCTGGGAATGGATTTATCTTCATGGTGACTGTGCTGCAATGGCTCCAGAAGAGGAAGATGCCACCTTGTGACTTCCTCCTGACTTGTTTGAGTGCCTCCAGATTACTAACACAGTTGAATGCTATGGCCATCTATTTTATGCAACTCTTTTATCCTTCAAGTAGAAGTGCAATGCTCTTTTTCTCCTGGGTCTTTCTCAAGATGGCCAGTCTTTGGTGTGTCTCCTGGCTCAGCATTTTCTACTGTGTGAAGGTCATCAACTTTTCCAACTCTTTACTCCTTTGGCTCAAGTTAAGGATCAATCTGCTTCTACCCAAACTACTTGGAATATCAGTGGTCATTTTCATGGTCTCTTCTCTTCCTTCCATCTTCACATTTCATAAATGCAATGAACCATGTAATCAGACTGTAGCACCCCTAATTAATGAGAAAGCTGACAACATGTGGATTAGTTATTTCCCAGTGGAGATAACATTTACTTGCATAAATTACAGCGTGAACATGGCAGCTACCCTTCTTTTGCTCATCTCCTTGTGGAGGCATGTGAGAAACCTCAGAAAGAATGGTACTAGTGTTCAGGACCTCAACACTCAGGTCCACCTCAAAGTCATGAGGCCTTTGTTGATCACTCTCTTACTCTACCTTTTATATATTGCTAGTTTGATATTAATGGATACTGGCTTTTTTTATTTTCAAGCAAACCTATCACTGATTGGAGAGATAATGGTTACCATATTTCCTTCAGTGCATGCCATAATATTAATATGGACCAATCCAAAACTCAGAGAAGTGGCTGCTCACATGCTAATAATCAGACAAAGAGCTTGA

>Lizard_T2R5--Intact

ATGTCTCCATTTGCTATCATCTCCCTGAGCATTCTAGGTATTTTGTGGATTGTTGCCCTTTCAGGGAATGGATTTATCTTCACGGTGACTGTGCTGCAATGGCTCCAGAAGAGGAAGATGCCACCTTGTGAATTCCTTCTGACCTGTTTGAGTGCCTCCAGATTACTAACAGAATTCAATTCTATGGCCATCTATCTTTCACGTCTCTTTTATTCTTCAAGTAAAAGAGCAATGCTCCTTATTCCCTGGGTCTTTCTCAATATTGCCACTCTCTGGTGTGTCTCCTGGCTCAGCATTTTCTACTGTGTGAAGATCATCAACTTTTCCAACTCTTTACTCCTTTGGCTAAAATTAAGGATCAATCTGCTTTTACCCAAACTACTTGGAATATCAATGGTCATTTTCATGGTCTCTTCTCTTCCTTCCATCTTCACATTTTTTAATTACAAAGAACCATGTAATCAGACTGTAACACCACTATCTAACCAGGAAGCTGACCTCAGCATGTGGATTAGTTTTGCTCCACTGCAATTAACTTTCACTTGCATAAATTTCATCATGAACATAGCGGCAACCCTTCTTTTGCTCATCTCCTTGTGGAGACATGTGAGAAACCTCAGAAAGAGTGGAACTAGTGTTCAGGACCTCAACACTAAGGTCCACCTCAAAGTCATGAGGCCTTTGTTGATCACTCTCTTATTCTATCTTTTATTTATTGCTAATTTAATAGTAATGATAATTGACTTTTTTGATTTACAAACAAACTTGTCACTGATTGGAGAGATAATGGTGTCTGTATTTCCTTCAGCACATCCCATAATATTAATATGGACCAATCCAAAACTCAAAGAAGTGGCTGCTCACACTGCTCAACATCAGACAAAGAGCTTAAAAAAAGGAGATGGTGGCAACAGGTATCCACCCCCGTCTTCGAACAGAGATTGCTTCTATCAGTGTGCGAGGTAA

>Lizard_T2R6--Intact

ATGTCTCCATTTGCTATCATCTCCTGGAGCATTCTAGGCATTTTGTGCATTGTTTCCCTTTCAGGGAATGGATTTATCTTCATGGTGACTGTGCTGCAATGGCTCCAGAAGAGGAAGATGCCACCTTGTGAATTCCTCCTGACCTGTTTGAGTGCCTCCAGATTACTAACAGAGTTGGATTGCATGGCCATCTATTTTATGCATCTATTTTCTTTTTCTGGTAGTAGAAGAATACTCTATTTCTTCTGGATCTTTTTCGATATGGCCAGTCTCTGGTGTACCTCCTGGCTCAGCATTTTCTACTGTGTGAAGGTCGTCAACTTTTCCAACTCTTTACTCCTTTGGCTAAAGTTAAGGATCAATCTGCTTCTACCCAGACTACTTGGAATATCAATGACCATTTTCATGGTCTCTTCAATTCATTCCATCTTCAGGTTTTTCAAATACAAAGAACCATGTAATCAGACTGTAACACCACTAACTAATGAGGATACTGACATCAGCATGTGGATTAGTTTTATTCCAGTACAGATAACTTTCACTTGCATAAATTTCAGCATGAACATAGCGGCAACCCTTCTTTTGCTCATCTCCTTGCGGAGGCATGTGAGAAACCTCAGAGGGAATGGTACTAGTGTCCAGGACCTCAACACTCAAGTCCACCTCAAAGTCATGAGGCCTTTGTTCATCACTCTCTTACTCTACCTTTTATTTATTGTTAGTTTGATAATAATGATAAGTAACTTTTCCCTGTTTCAAACAAATCTATCACTGAGTACAGAGATAACGATGTCCATATTTCCTTCAGCACATTCCATAATATTAATATGGACCAATCCAAAACTCCGAGAAGTGGCTGTTCACATGCTAAACATCAGACAAAGAGCTTAA

>Lizard_T2R7--Intact

ATGGCTGCTAACCTAGTGTCTCCATTTGCTATAATCTCCTGGAGCATTATAGGCATTTTGTGCATTGTTTCCCTTTCTGGGAATGGATTTATCTTCACGGTGACTGTGCTGCAATGGCTCCAGAAGAGGAAGATGCCACCTTGTGACTTCCTCCTGACTTGTTTGAGTGCCTCCAGATTACTAACACAGTTGAATGCTATGGCCATCTATTTTATGCAACTCTTTTATCCTTCAGCTAGAAGTGCAATGCTCTTTTTCTCCTGGGTCTTTCTCAATATGGCCAGTCTTTGGTATGTCTCCTGGCTCAGCATTTTCTACTGTGTGAAGGTCATCAACTTTTCCAACTCTTTACTCCTTTGGCTCAAGTTAAGGATCAATCTGCTTCTACCCAAACTACTTGGAATATCAGTGGTCATTTTCATGGTCTCTTCTCTTCCTTCCATCTTCACATTTCATAAATGCAATGAACCATGTAATCAGACTGTAACACCCCTAATTAATGAGAAAGCTGACAACATGTGGATTAGTTATTTCCCAGTGGAGATAACATTTACTTGCATAAATTACAGCGTGAACATAGCAGCAACCCTTCTTTTGCTCATCTCTTTGTGGAGGCATGTGAGAAACCTCAGAAAGAATGATACTAGTGTTCAGGACCTCAACACTCAGGTCCACCTCAAAGTCATAAGGCCTTTGTTGATCACTCTCTTACTCTACCTTTTATATATTGCTAGTTTGATATTAATGGATACTGGCTTTTTTTATTTTCAAGCAAACCGATCACTGATTGGAGAGATAATGGTTACCATATTTCCTTCAGTGCATGCCATAATATTAATATGGACCAATCCAAAACTCAGAGAAGTGGCTGCTCACATGCTAAACATCAGACAAAGAGCTTAA

>Lizard_T2R8--Intact

ATGTCTCCATTTGGTATCTTCTCCTGGAGCATTCTAGGTATTTTGTGGATTGTTTCCCTTTCAGGGAATGGATTTATCTTCACAGTGACTGTGCGGCAATGGCTCAAGAAGAGGAAGATGCCACCTTGTGAATTCCTTCTGATCTGTTTGAATGCCTCCAGATTACCTACACTGTTGAATACTATGGCCATCTATTTTATGCATCTCTTTTATTCTTCAGGTAGAAGAATGATGCTCCTTATTCCCTGGGTCTTTCTCAATATTGCCAGTCTCTGGTGTGTCTCTTGGCTCAGCATTTTCTACTGCGTCAAGGTCATCAACTTTTCCAACTCTTTACTCCTTTGGCTAAAGTTAAGGCTCAATCTGCTTCTACCCAAACTAGTTGGAATATCGATGGTCATTTCCATGGTCTCTTCTCTTTCTTCCATTTTCACATTTCATCAGTGCAATGAACCATGTAATCAGACTGTAACACCACCAACCAAACATGAAGCTGAAAACTGTATGTGGATTAGTTTTTTTCCACTGAAGATAACTTTTGCTTTCATAAATTTCAGTGTGAACATAGCAGCAACCCTTCTTTTGCTCATCTCCTTGTGGAGGCATGTGAGAAACCTCAGAAAGAAGGGTACTAGTGTTCAGGACCTCAACACTCAAATCTACCTCAAAGTCATGAGGCCTTTGTTGATCACTCTCTTACTCTACCTTTTATATATTGCTAGTTTGATTACAATGAGTGGCTTTTTAAATTTGCAAAGAAAACAAGCACTGACTGCAGAGATAATGATTACCATATTTCCTGCAATGCATCCCAGAATAATAATATGGACCAATCCAAAACTCAAAAATGTGGCTGCTCACATGTTAAACATCAAGCAAAGACCTTAA

>Lizard_T2R9--Intact

ATGGCTGCTAACCTAGTGTCTCCATTTGCTATAATCTCCTGGAGCATTATAGGCATTTTGTGCATTGTTTCCCTTTCTGGGAATGGATTTATCTTCATTGTGACTGTGCTGCAATGGCTCCAGAAGAGGAAGATGCCACCTTGTGACTTCCTCCTGACCTGTTTGAGTGCCTCCAGATTGCTAACACAGTTGAATGCTATGGCCATCTATTTTATGGAACTCTTTTATCCTTCAAGTAGAAGTGCAATGCTCTTTTTCTCCTGGGTCCTTCTCAATATGGCCAGTCTTTGGTGTGTCTCCTGGCTCAGCATTTTCTACTGTGTGAAGGTCATCAACATTTCCAACTCTTTACTCCTTTGGCTCAAGTTAAGGATCAATCTGCTTCTACCCAAACTACTGGGAATATCAGTGGTCATTTTCATGGTCTCTTCTCTTCCTTCCATCTTCACATTTCATAAATGCAATGAACCATGTAATCAGACTGTAACACCCCTAATTAACGAGAAACCTGGCAACATGTGGATCAGTTTTTTCCCAGTGGAGATAACATTTACTTGCATAAATTTCAGCATGAACATAGCGGCAACCCTTCTTTTGCTCATCTCCTTGTGGAGGCATGTGAGAAATCTCAGAAAGAATGGTACTAGTGTTCGGGACCTCAACACTCAGGTCCACCTCAAAGTCATGAGGCCTTTGTTGATCACTCTCTTACTCTATCTTTTATTTATTGCTAGTTTGATAACTATGGCAACTGGCTTTTTAAATTTGCAAACAAAACAAGCACTGATTGGAGAGATAATGGTTACCATATTTCCTTCAGTGCATGCCATAATATTAATATGGACCAATCCAAAACTCAGAGAAGTGGCTGCTCACATGCTAAACATCAGACAAAGTGCTTAA

>Macaque_T2R1--Intact

ATGATGGAACTCACCGAGGGGGTGTTCCTGATTGTGTGTGGCGCTCAGTTCACACTGGGAATTCTGGTCAATGGTTTCATTGGTTTGGTCAATGGTAGGAGCTGGTTCAAGACCAAGAGAATGTCTTTGTCTGACTTCATCATCGCCACCCTGGCACTCTCAAGGATCATTCTGCTGTGTATTATCTTGACTGATAGTTTTTTAATAGTATTCTCTGTCAACGAACATGATTCAGGGATAATAATGCAAATTATTGATGTTTTCTGGACCTTTACAAACCATCTGAGCATTTGGTTTGCCACTTGTCTTGGTGTCCTCTACTGCTTGAAAATCGCCATTTTCTCTCACCCCACATTCCTCTGGCTCAAGTGGAGAGTTTCTAGGGTGATGGTATGGATGCTGTTGGGTGTACTGCTCTTATCCTGTGGTAGTACCGCATCTCTGATCAATGAGTTTAAGCTCTATTCTGTCCTTAGGGGAATTGAGGCCACCAGGAATGTGACTGAACACTTCAGAAAGAAGAGGAATGAGTATTATCTGATCCATGTTCTTGGGACTCTGTGGTACCTGCCTCCCTTAGTTGTGTCCCTGGCCTCCTACTTTTTGCTCATCTTCTCCTTGGGGAGGCACACACGGCAGATGCTGCAAAATAGTACAAGCTCCAGAGATCCAAGCACTGAGGCCCACAAGAGGGCCATCAGAATCATCCTTTCCTTCTTTTTTCTCTTCTTACTTTACTTTCTTGCCTTTTTAATTGCATCATTTGGTAATTTCCTACCAAAAACCAAGATGGCTAAGATGATTGGCGAGGTAATGACAATGTTTTATCCTGCTGGCCACTCGTTTGTTGTCATTCTGGGGAACAGCAAACTGAAGCAGACATTTGTAGAGACGCTCCGGTGTGAGTCTGGCCACCTGAAGCCTGGATCCAAGGGACCCATTTTCTCTTAG

>Macaque_T2R10--Intact

ATGTTAACTCTAACTCACGTCTGCACTGTGTCCTATGAAGTCAGGAGCACATTTCTGTTCATTTCAGTCCTGGAGTTTGCAGTGGGGTTTCTGACCAACGCCTTCATTTCCTTGGTGAATTTTTGGGACGTAGTGAAGAGGCAGCCACTGAGCAACAGTGATTGTGTGCTTCTGTGTCTCAGCATCAGCCGGCTTTTCCTGCATGGACTGCTCTTCCTGAGTGCTATCCAGCTTACCCACTTCCAGAAGTTGAGTGAACCACTGAACCACAGCTACCAAGCCATCCTCATGCTATGGATGATTGCAAACCAAGCCAACCTCTGGCTTGCCGCCTGCCTCAGCCTGCTCTACTGCTCCAAGCTCATCCGTTTCTCTCACACCTTCCTGATCTGCTTGGCAAGCTGGGTCTCCAGGAAGATATCCCAGATGCTCCTGGGTATTATTCTTTGCTCCTGCATCTGCACTGTCCTCTGTGTTTGGTGCTTTTTTGGCAGACTTCACTTCACAGTCACAACTGTGCTATTCATGAATAACAATACAAGGCTCAACTGGCAGATTAAAGATCTCAACTTATTTTATTCCTTTCTCTTCTGCTATCTGTGGTCTGTCCCTCCTTTCCTATTGTTTCTGGTTTCTTCTGGGATGCTGACTGTCTCCCTGGGAAGGCACATGAGGACAATGAAGGTCTATACCAGAGACTCTCGTGACCCCAGCCTGGAGGCCCACATTAAAGCCCTCAAGTCTCTTATCTCCTTTTTCTGCTTCTTTGTGATATCATCCTGTGCTGCCTTCATCTCAGTGCCCCTACTTATTCTGTGGCATGACAAAATAGGGGTGATGGTTTGTGTTGGGATAATGGCAGCTTGTCCCTCTGGGCATGCAGCCGTCCTGATCTCAGGCAATGCCAAGTTGAGGAGAGCTGTGACAACCATTCTGCTCTGGGCTCAGAGCAGCCTGAAGGTAAGAGCCGATCACATGGCAGATTCCAGGACACTGTGCTGA

>Macaque_T2R11--Intact

ATGATACCCATCCAACTCAGTGTCTTCTTCATGATCATCTATGTGCTTGAGTCCTTGACAATTATTGTGCAGAGCAGCTTAATTGTTGCAGTGCTGGGCAGAGAATGGCTGCAAGTCAGAAGGCTGATGCCTGTGGACATGATTCTCATCAGCCTGGGCATCTCTCGTTTCTGTCTACAGTGGACATCAATGCTGAACGATTTTTGCTTCTATTTTAATTTTAATTATGTACTTTGCAACTTAACAATCACCTGGACATTTTTTAATGTCCTTACATTCTGGTTAAACAGCTTGCTTACCATCTTCTACTGCATCAAGGTCTCTTCTTTCACCCATCCCATCGTTCTCTGGCTGAGGTGGAGAATTTTGAGGTGGCTTCCCTGGCTATTACTAGGTTGTCTGATGATTACTTGTGTGACAATCATCCCTTCAGCTATTGGGAATTACATTCAAATTCAGTTTCTCACCATGGAGCATCCACCCAGAAACAGCACTGTAATTGACAGACTTCAAAAGTTTCATCAGTATCTGCACCAGGCTCATACAGTCGCGTTGGTTATTCCTTTCATCCTGTTCCTGGCCTCCACCATCTTGCTCATGGCATCATTGACCAAGCAGATACAACATCATGGCACTGGTCACTGCAATCCAAGCATGAAAGCGCACTTCACTGCCCTGAGGTCCCTTGCCATCTTGTTTATCGTGTTTACCTCTTACTTTCTAACCATACTTATCACCATGATAGGTACTCTATTTGATAAGAGATGTTGGTTATGGTTCTGGGAAGCTTTTGTCTATGCTTTCATCTTTATGCATTCCACTTCACTGATGCTGAGCAGCCCTACATTGAAAAGGATTCTAAATGGAAAATGCTAG

>Macaque_T2R12--Intact

ATGCCAAGTACAATAGAGGCAATATATATTATTTTAATTGCTGGTGAACTGACCATAGGGATTTGGGGAAATGGATTCATTGTACTAGTTAACTGTATTGACTGGCTCAAAAGAAGAGATGTTTCCTTGATTGACATCATCCTGATCAGCTTGGCCATCTCCAGAATCTGTTTGCTGTGTGTAATATCATTAGATGGCTTCTTTATACTGCTCTTTCCAGGTACATATGACACTAATGTGCTAGAAAGCATTATGGATGCTGTCTGGACATTTGCCAATAATTCAAGTCTCTGGTTTACTTCTTGCCTCAGTATCTTCTATTTACTCAAGATAGCCAATATATCCCACCCATTTTTCTTCTGGCTGAAGCTAAAGATCAACAAGGTCATCCTTGCGATTCTTCTGGGGTCCTTTCTTATCTCCTTAATTATTAGCTTTCCAATTAATGGTATGTGGTATCACCTTTTCAAGGTCAGTCATGAAGAAAACATTACTTGGGCATTCAAAGTGAGTACAATTCCAGGTGCTTTCAAACAGTTAACCCTCAACCTGGGGGCGATGGTTCCCTTTATCCTTTGCCTGATCTCATTTTTCTTGTTACTTTTCTCCTTAGTTAGACACACCAAGCAGATTCAACTGCATGCTACAGGGTTCAGAGACCCCAGCACAGAGGCCCACATGAGGGCCGTAAAGGCAGTGATCATCTTTCTGCTCCTCCTCATTCTGTACTACCCGGTCTTTCTTGTTATGACCTCTAGCACTCTGATTCCCCAGGGAAAATTAGTGTTGATGATTGGTGACATAGTAACTGTCATTTTCCCATCAAGCCATTCATTCATTCTAATCATGGGAAACAGCAAGTTGAGGGCAGCTTTTCTGAAGATGTTAAGATTTGTGAAGGGTTTCCTTAGAAGAAAGCCTTTTGTTCCATAG

>Macaque_T2R13--Intact

ATGCTAAGTGTAGTGGAAGGCATCTTCATTTTTGTTGTAATTAGTGAATCAGTATTTGCGGTTTTAGGGAATGGATTTATTGGACTTGTAAACTGCATTGACTGTGCCAAGAATAAGTTATCTACAATTGGCTTTATTCTCACCGGCTTAGCTATTTCTAGAATTTTTCTGATATGGCTAATAATTACAGATGGATTCATACGGATATTCTCTCCAGATATATATGCTTCTGGTAACCTAATTGAATATATTAGTTACTTTTGGGTAATTAGTAATCAATCAAGTATGTGGTTTGCCACCAGCCTCAGCATCTTCTATTTCCTGAAGATAGCAAATTTTTCCAACTACATATTTCTCTGGTTGAAGAGTAGAACAAATAGGGTTCTTCCCCTTCTGATGGGATTCTTACTTATTTCATGCTTACTTAATTTTGCATATATTGCGAAGATTCTTAATGATTTTAAAATGAAGAATGACACAGTCTGGCGTCTCAACATATTTAAAAATGAATACTTTGTTAAGCAGATTTTGCTAAATCTGGGAGTCATTTTCTTCTTTACACTATCCCTAATTACAAGTGTTTTGTTGATCATTTCCCTTTGGAGACACAACAGGCAGATGCAATCAAATGTGACAGGACTGAGAGACTCCAACACTGAAGCTCATGTGAAGGCAATGAAAGTTTTAATATCTTTCATCATCCTCTTTATCTTGTATTTTATAGGCATAGCCATAGAAATATCGTATTTTACTGTGCCAGAAAACAAACTGTTGCTTATGTTTGGAATGACAACCACAGCCATCTATCCCTGGGGTCACTCATTTATCTTAATTCTAGGAAACAGCAAGCTAAAGCAAGCCTCTTTGAGGGTACTACAGCAATTGAAGTGCTGTGAGAAAAGGAAAAACCTCAGAGCCACATAG

>Macaque_T2R14--Intact

ATGGAAAGTGCCCTGCTGAGTATCCTCACTCTTGTAATAATTGCAGAATTCGTAATTGGGAATTTGAGCAATGGATTTATAGTACTGATAAACTGCATTGACTGGGTCAGTAAAAGACAGCTGTCCTCAGTTGATAAAATCCTCACATTCTTGGCAATCTCCAGAATTGGGCTGATCTGGGAACTATTAGTAAGTTGGTTTTTAGGTCTGCATTATCTAGCCATATTTGTGTCTGGAACAGGGTTAAGAATTATGATTTTTAGCTGGGTAGTTTCTAATCACTTCAGTCTCTGGCTTGCTACAATCCTCAGCATCTTTTATTTGCTCAAAATAGTGAGTTTCTCTAGCCCTGCTTTTCTCTATTTGAAGTGGAGAGTAAACCAAGTGATTGTGATGATACTGCTGGAAACCTTGATCTTCTTATTTTTAAATCTGATACAAATAAACATACATATTAAAGACTGGCTGGACCGATGTGAAAGAAACACAATTTGGAATTTCAGTATGAGTGGCCTTCCAACATTTTCAGTGCCGGTCAAATTCACCATGACTATGTTCAGTCTAGCACCATTTACTGTGGCCCTCATCTCTTTTCTCCTGTTAATTTTCTCCTTGTGGAAACATCTCCAGAAAATGCAGCTCAATTACAAAGGACACAGAGACCCCAGGACCAAGGCCCACATAAATGCATTGAAAATTGTGATCTCATTCCTTTTACTCTATGCCAGTTTCTTTCTATGTATTCTCATATCATGGATTTCTGAGCTGTATCAGAATATACTGATCCACATGTTTTGTCAGACGATTGGAGTCTTCTATCCTTCAAGCCACTCCTTTCTTCTGATTCTAGGAAACCCTAAGTTAAGACAGGCCTCTCTTTTGGTGGCAGCTAAGGTATGGGCTAAACGTTGA

>Macaque_T2R15--Intact

ATGGATGGTGTCATAAAGAGCATATTTACATTCATTTTAATTGTGGAATTTATAATTGGAAATTTAGGAAATAGTTTCATAGTACTGGTGAACTGTATTGACTGGGTCAAGAGAAGAAAGATCTCTTTAGTTGATCAGATTCTCATTGCTTTGGCAATCTCTAGAATTAGTCTGGTTTGGTCAATATTTGGAAGCTGGTGTGTATCTGTGTTTTTCCCAGCTTTATTTGCCACTGAAAAACTGTTAAGAATGCTTACTAATATCTGGACAGTGACCAATCATTTTAGCGTCTGGTTAGCTACAATCCTAGGTACTTTTTATTTTCTCAAGATAGCCAATTTTTCTAACTCTATTTTTCTCTACCTAAAGTGGAGAGTTAAAAAGGTGGTTTTAGTGCTGCTTCTTGTGACTTTGGGCCTCTTGTTTTTAAATATTTTACTGATAAACATTCATATAAATACCAGTATCAATGGATACAGAGGAAACATGACTTGCAGTTCCGCTTCATGCAACTTTATACGATTTTCCAGGGCTATTGCATTAACCAGCACTGTGTTCGTTTTAATTCCCTTTACTTTGTCCCTGGCAACTTCTCTTCTGCTCAGCTTCTCCCTGTGGAAACATCACAAGAAGATGCAGCACACTGTCAAAGGATACAGAGACGTCAGCACCAAGGCCCACAGAGGAGTTATGCAAACTGTGATCACTTTCCTCCTCCTCTATGCCGTTTTCCTTCTGACTTTTTTCATATCAATTTGGGCCTCTGTACGGTTGAAGGAAAATCAAATTATTATTCTTTCTGAGATGATGGGACTGGCTTATCCTTCAGGTCACTCATGTGTTCTGATTCTTGGAAACAAGAAGCTGAGACAGGCCTCTCTGTCAGTCCTATGGTGGCTGAGGTACAGGTTTAAAGATGGGGAGCCCTCAGGTCACAAAGAATTTAGGGAATCATCTTGA

>Macaque_T2R16--Intact

ATGATAACTTTTTTACCCATCATTTTTTCCATTCTAGTAGTGTTTACATTTGTTATTGGAAATTTTGCTAATGGTTTCATAGCATTGGTAAATTCCATTGAGTGGGTCAAGAGACAAAAGATCTCCTTTGCTGACCAAATTCTCACTGCTCTGGCAGTCTCCAGAGTTGGTTTGCTCTGGATATTATTATTAAATTGGTATTCAACTGTTTTGAATCCAGCTTTTTATAGTGTAGAAGTAAGAACTATTGTTTATAATCTCTGGGCAGTAATCAACCATTTCAGCAACTGGCTTGCTACTAGCCTAAGCATATTTTACTTACTCAAGATTGCCAATTTCTCCAACCTTATATTTCTTCACTTAAAGAGGAGAGTTAAGAGTGTCGTTCTGGTGATACTGTGGGGGCCTTTGCTATTTTTGGTTTGTCATCTTTTTGTGGTAAACATGAATGAGATTATACAGACAAAAGAATATGAAGGAAACATGACTTGGAAGAGCAAATTGAGGAGTGCAATGTACCTTTCAAATACGACTGTAACCATACTAGCAAACTTAGTACCCTTCATTCTGACCCTAATATCTTTTCTGCTGTTAATCTGTTCTCTGTGTAAACATCTCAAGAAGATGCAGCTCCGTGACAAAGGCTCTCAAGATCCCAGCACCAAGGTCCACATAAAAGCTTTGCAAACTGTCATCTCCTTGTCGTTATGTGCCATTTACTTTCTGTCCATAATGATATCAAGTTGGAGTTTGGGAAGGGTGGAAAACAAAGCTATCTTCATGTTCTGCAAAGCTATTAGATTCAGCTATCCTTCAGCCCACGCATTCATACTGATTTGGGGAAACAAGAAGCTAAAGCAGACTCTTCTTTCAGTTTTGTGGAACGTGAGGTACTGCGTGAAAGGACAGAAGCTTCAATCTCCATAG

>Macaque_T2R17--Intact

ATGATAACTTTTCTACCCATCATTTTTTCCATTCTAGTAGTGGTTACATTTGTTATTGGAAATTTTGCTAATGGCTTCATAGCATTGGTAAGTTCCGTTGAGTTGGTCAAGAGACAAAAGATCTCCTTTGCTGACCAAATTCTCACTGCTCTGGCGGTCTCCAGAGTTGGTTTGCTCTGGGTATTATTACTAAATTGGTATTCAACTGTGTTGAATCCAGCTTTTTATAGTGTAGACCTAAGAACTACTGCTTATAACCTCTGGGCAGTAACCAGTCATTTCAGCAACTGGCTTGCTACTTGCCTCAGCATATTTTATGTGCTCAAGATTGCCAATTTCTCCAACCTTATGTTTCTTCACTTAAAGAGGAGAGTTAAGAGTGTCATTCTGGTGATGCTGTTGGGGCCTTTGCTATTTTTGGCTTGTCATCTTTTTGTCATAAACGTGAATGAGATTGTACGGACAAAAGAATATGAAGGAAACATGACTTGGAAGATCAAATTGATGAGTGCAATGCACTTTTCAAATACGACTGTAACCATGCTAGCAAACTTAGTACCCTTCACTTTGACCCTACTATCTTTTGTGCTGTTAATATGGTCTTTGTGTAAACATCTCAAGAAGATGCAGCTTTATGGTAAAGGGTCTCAAGATCCCAGCACCAAGGTCCACATAAAAGCTTTGCAAACTGTGATCTCCTTCCTCTTCCTATGTGCCATTTACTTTCTGTCCATAATGATATCAGTTTGGAATTTGGAGAGGCTGGAAAATAAATCTTTCTTCCTGTTCTGCAAAGCTATTAGAATCATGTATCCTTCAGCCCACACATTCGTCCTGATTTGGGGAAACAAGAAGCTAAAGCAGACTTTTCTTTCAGTTTTGTGGCAAGTGAGGTACTGGGTGAAAGGACAGAAGCCTTCATCTCCATAG

>Macaque_T2R18--Intact

ATGACAGATAAAGTACAGACTACTTTACTATTCTTAGCAATTGGAGAGTTTTCAGTGGGGATCTTAGGGAATGCGTTCATTGGATTGGTAAACTGCATGGACTGGGTCAAGAAGAGGAAAATTGCCTCCATTGATTTAATCCTCACAAGTCTGGCCATTTCCAGAATTTGTCTATTATGTGTAATATTATTAGATTGTTTTATGTTGGTGCTGTATCCAGATGTCTATGCCACTGGTAAACAAATGAGAATCATTGACTTCTTCTGGACACTAACCAACCATTTAAGTATCTGGTTTGCAACCTGCCTCAGCATTTACTATTTCTTCAAGATAGCTAATTTCTTTCACCCACTTTTCCTCTGGATGAAGTGGAGAATTGACAGGGTGATTTCCTGGATTCTACTGGGATGCATGGTTCTCTCTGTGTTTATTAACCTTCCAGCCACTGAGAATTTGAATGCTGATTTCAGGCGTTGTGTGAAGGCAAAGAGGAAAACAAACTTAACTTGGAGTTGCAGAGTAACTAAAGCTCAACATGCTTCTACCAAGTTATTTCTCAACCTGGTAACGCTGCTCCCTTTTTCTGTGTGCCTGATGTCATTTTTCCTCTTGATCCTCTCCCTGTGGAGACATATCAGGCGAATGCAGCTCAGTGCCACAGGGTGCAGAGACCCCAGCACAGAAGCCCACGTGAGAGCCCTGAAAGCTGTCATTTCCTTCCTTCTCCTCTTTATTGCCTACTATTTGTCCTTTCTCATCGCCACCTCCAGCTACTTTATTCCAGAGACGGAATTAGCTGTGATTTTTGGTGAGTTCATAGCTCTAATCTACCCCTCAAGCCATTCATTTATCCTAATACTGGGGAACAGTAAATTAAGACGTGCATCTCTAAAGGTGCTTTGGACAGTAATGTCTATTCTAAAAGGAAGAAAATTCCAACAACATAAACAAATCTGA

>Macaque_T2R19--Intact

ATGTTCAGTCCTGCAGATAACATCTTTATAATCCTAATAACTGGAGAATTCATAATAGGAATATTGGGGAATGGATACATTGGACTAGTCAACTGGATTGACTGGATTAAGAAGAAAAAGATCTCCACAATTGACTGCATCCTCACCAATTTAGTTATCTCCAGAATTTGTTTGATCAGTGTAATGGTTGTAAATGGCATTGTAATAGTACTTTACCCGGACATTTATACAAAAAGTAAACTACAGATAGTCATTTGTACCTTCTGGACATTTGCCAACTACTTAAATATGTGGTTTACTGCCTGCCTTAATGTCTTCTATTCTCTCAAGGTAGCCAATTCCTCTCACCCACTTTTTCTCTGGCTGAAGCGGAAAATTGATACGGTGGTTCGTTGGATCCTGCTGGGATGTTTTGCCATTTCCTTGTTGGTCAGTCTTATAATAGCAACAGTACTGAGTCATGATTATAGGTTTCATGCAATTGCCAAACATAAAAGAAACGTTACTGAAATGTTCCATGTGAGTAAAATGCCATATTTTGAACCCTTGACTCTCTTTAACTTGCTTGCAATTGTCCCATTTATTGTGTCATTGATGTCATTTTTCCTTTTAGTAAGATCTTTATGGAGACATACCAAGCAAATAAAACTCCATGCTACTGGCGGTAGAGACCCAAGCACAGAAGCTCATGTGAGAGCCATTAAAACTATGACTTTCCTTATCTTCTTTTTTTTCCTATACTATATTACTTCTCTTTTGGTGAACTTTAGCTATCTTATTACAAACTACAAGTTAGCTATGGCGTTTGCAGAGATTGTAGCAATTCTCTATCCCTCGGGTCACTCACCAAACTGA

>Macaque_T2R2--Intact

ATGCCCTCCTCGCCCACATTGATCTTCATGGCCATCTTTTGCCTGGAGTCATTGGCTGCAATGATGCAGAATGGCTTCTTGGTCACAGTGCTGGGCAGGGAGTGGGTAAGGTGCCGGACTCTGTCTGCAAGTGACATGATTGTGGCCTGTCTCGCTGCCTCCCGGTTCTGCCTCCATGGGGTAGCCATGGTGAACAACTTCCTGGCCTCCTTAGATTTTTGGCGCACAGTTCCCTATGTGAACACCTTCTGGGACCTTTTCAATGCCCTCACTTTGTGGTTTACTGCCTTGCTTGCTGCTTTCTACTGTGTGAAGATCTCATCTTTCTCCCACCCCACCTTCGCCTGGCTGAAGTGGAGGATCTCTCGGTCAGTGCCCAGGCTGATCCAGGTCTCCCTGATCATCTGTGGCCTGGAAGTCATCTCATCAGCCACTGGGAGTGTACTGTTTGGTCCGAGGAAGGTCTCCCTGAATTCCTGCGGAAACGAAACTCTAGTTTATAGAGTGCAGGCTTCATTTGAGCTCTACTTTTTCCTTTATGAAGGGTTTGTGTTGTTGATTCCATTCCTCCTGTTCCTAGTGTCCACTGTCTTGCTCATAGTCTCACTGTGCTGGCACTTGGGGCAGATGAGGGACCGCAGGCCCGGGCCCTGTCATCCCAGCACCCAGGCTTACACCGTGGCTCTAAAGTCACTCACTGTTTCCCTCATCTTCTGTACATTGTACTTCCTGTTCTTGTTTGTTTCTGCTTTGAAAATCATAAACTTTCAGAATCACTGGCACTGGGCCTGGGAAGTGCTAATCTATGCCAACATCTGTCTGCACTCTACCTTCCTGGTGCTGAGGAGCCCCAAACTGAAAAAGAGCCTGAAGACATGGCCTCAGCTGCAGTGCCCATGTGCTGCTGGCTCATAG

>Macaque_T2R20--Intact

ATGATAACTTTTCTGTCCATCACTTTTTCCATTCTAGTAGGGGTTATATTTGTTATTGGAAATTTTGCTAATGGCTTCATAGCATTGGTAAATTCCATTGAGTGGGTGAAAAGACAAAAAATCTCCTTTGCTGACCAAATTCTCACTGGTCTGGCTGTCTCCAGAGTTGGTTTGCTCTGGGTATTATTACTACATTTGTATGCAACTGAGTTTAATCTAGCTTTTTATAGTGTAGAAGTAAGAATCACTGCTTATAATGTCTGGATAGTGACCAACCATTTCAGCAACTGGCTTTCTACTAGCCTCAGCATGTTTTATTTGCTCAAGATTGCCACTTTCTCCAACCTGATTTTTCTTCACTTAAAGAGGAAAGTTAAGAGTGTCATTCTGGTGACACTGTTGGGGCCTTTGCTATTTTTGGTTTGTCATCTTTTTGTGATGAACATGAATCATATTGTGTGGAGAAAAGAATATGAAGGAAACATTACTTGGAGGATCAAATTGAGGAGTGCAATGTACCTTTCAAATGTGACTGTAACCATGCTAGCAAACCTTATACCCCTCACTCTGACCCTGATGTCTTTTCTGCTGTTAATCTGTTCTCTGTGTAAACATCTCAAGAAGATGCAGGTCCACGGCAAAGGATCTCAAGATCCCAGCACCAAGGTCCACATAAAAGCTTTGCAAACTGTGACCTCCTTTCTCCTGTTATGTGCCATTTACTTTCTGTCCATGATCCTATCAGTTTGGAATTTTGAGCTGGAAAAGAAACCTGTCTTCATGTTCTGCCAAGCTGTCATATTCAGCTATCCTTCAACCCACCCACTCATCCTGATTTGGGGAAACAAGAAGCTAAAGCAGATTTTTCTTTCAGTTTTGTGGAACGTGAGATACTGGGTGAAAGGACAGAAGCCTTCATCTCCATAG

>Macaque_T2R21--Intact

ATGATGAGTTTTCTACCCATTGTTTTTTCCATTCTAGTAGTGGTTGCATTTGTTCTTGGAAATTTTGCCAATGGCTTTATAGCACTGATAAATTTCATTGCCTGGTTCAAGAGACAAAAGATCTCCTCAGCTGATCAAATTATTGCTGCTCTGGCGGTCTCCAGAGTTGGTTTGCTCTGGGTAATAGTCTTACACTGGTATGCAACTGTGTTGAATCCAAATTCATCTAGTTTAAAAGTAAGAATTTTTCTTTCTAATGCCTGGGCAGTAACCAACCATTTCAGCATCTGGCTTGCTACTAGCCTCAGCATATTTTATTTGCTCAAGATCGTCAATTTCTCCAGACTTATTTTTCATCACTTAAAAAGGAAGGTTAAGAGTGTAGTTCTGGGGATACTGTCAGGGGCTTTGTTATTTTTGGTTTGTGATCTTGTGGCGGAAAACGTGTATATAAATGTGTGGACGAAAGAATATGAAAGAAACATAACTTGGAAGATCAAATTGAGGAATGCAACGTACCTTTCTAACTTGATTGTAGTCACGCTAGCAAACTTGATACCATTCACTCTGACCCTGATATCTTTTCTGCTGTTAATCTGCTCTCTGTGTAAACATCTGAAGAAGATGAAGCTCTATGGCAAAGGATCGCAAGATCCTAGCACCAAGATCCACATAAAAGCTCTGCAAACTGTGACCTCTTTCCTCATACTATTTGCCGTTTACTTTCTATGTCTAATCGTATCATTTTGGAATTATAAGAAGCAACAGAAAGAACTTGTCTTAATCCTTTGCCAAGCTATTGGAATCATATATCCATCGTTCCACTCATTCCTTCTGATTTGGGGGAACAAGAAGCTAAAGCAGAACTTTCTTTCAATTTTGTGGCAGGTGACTTGCTGGGCCAAAGGACAGAACCTGTCAACTCCATAG

>Macaque_T2R22--Intact

ATGATGTATTTTCTGTTCATCATTTTGTCAATTCTGTTAGTGTTTGCATTTGTTCTTGGAAATTTTGCCAATGGCTTCATAGCTCTAGTAAATGTCATTGACTGGGTTAAGACACGAAAGATTTCCTTAGTTGACCAAATTCTCACTGCTCTCGTGATCTACAGAATTGGTTTACTCTGGGCCATATTATTATATTGGTATGCAACTATGTTTAATTCGGCTTTATGTAGTTCAGAAGTAAGAATTTTTGCTTCTAATATCTCGGCAATAATCAACCATTTCAGCATCTGGCTTGCTGCTAGCCTCAGCATATTTTATTTGCTCAAGATTGCCAATTTCTCCAACCTTATTTTTCTCCACCTACAGAAGAGAATTAAGAGTGTTGTTTGGGTGATGCTGTTGGGGCCCTTGGTATTTTTTATTTGTAATCTTGCTGTGGTAACCACGGATGAGGGTGTGTGGACAAAAGAATATGAAGGAAATGTGACTTGGAAGATCAAATTGAAGAATGCAATACACCTTTCAAACTTGACTATAAGCACGCTAGCAAACCTCATACCCTTCACTCTGACCCTAATATGTTTTCTGCTGTTAATCTATTCTCTGTGTAAACATCTCAAGAAGATACAGCTCCATGGCAAAGGATCTCAAGATCTCAGCACCAAGGTCCACATAAAATCTCTGCAAACTGTGATCTCCTTCCTCATGTTGTTTGCCATTTACTTTCTGTGTCTAATCAGTTTAACCTGGAGTCCTTGGAAACAGCAGAACAAACTTGTGTTTCTGCTTTGCCAAACTCTTGCAATCATGTATCCTTCATTCCACTCCTTCATCCTGATTATGGGAAATAGGAAACTAAAACAGACTTTTCTTTCAGTTTTGTGGCAGGTGACATGCTGA

>Macaque_T2R23--Intact

ATGATAACTTTTCTACCCATCATTTTTTTCATTCTAGTAGTGGTTACATTTGTTATTGGAAATTTTGCTAATGGCTTCATAGCGTTGGTAAATTCCACTGAGTGGGTCAAGAGACAAAAGATCTCCTTTGCTGACCAAATTCTCACTGCTCTGGTGGTCTCCAGAATTGGTTTGCTCTGGGTGTTATTATTACATTGGTATTCAGTTGTGTTGAATCCAGCTTTTTATAGTGTAGAGGTAAGAACTACCACTTACAATGTCTGGGCAGTAACCAGCCATTTCAGCAACTGGTTTGCTACTTGCCTCAGCATATTTTATTTGCTCAAGATTGCCAATTTCTCCAACCTTATGTTTCTTCACTTAAAGAGGAGAGTTAAGAGTGTCATTCTGGTGATGCTGTTGGGGTCTTTGCTATTTTTGGTTTGTCATCTTTTTGTGATAAATATGAATGAGATTGTACAGACAAAAGAATATGAAGGAAACATGACTGGGAAGATCAAATTGAGGAGTGCAATGTACCTTTCAGATGCGACTGTAACCACGCTAGTAAACTTAGTACCCTTCACTCTGACCCTAATATCTTTTCTGCTGTTAGTCTGTTCTCTGTTTAAACATCTGAAGAAGATGCAGCTTCATGGCAAAGGATCTCAGGATCCCAGCACCAAGGTCCACATAAAAGCTTTGCAAACTGTGATCTCTTTCCTCTTGCTATGTGCCATTTACTTTGTGTTCCTAATCATATCAGCTTTGAGTTTTGAGAGTCGGGATAACAAACCTGTCTTCATGATCTGTGAAGCTGTTACATTCAGCTATCCTTCAACCCACCCATTCATACTGATTTGGGGAAACAAGAAGCTAAAGCGGACTTTTCTTTCAGTTTTGCGGCAAGTGAGGTACTGGGTGAAAGGACAGAAGCCTTCATCTCCGTAG

>Macaque_T2R24--Intact

ATGATACCTTTTCTACACATTTTTTTTTCAGTTCTAATATTGGTTTTATTTGTTCTTGGAAATTTTGCCAATGGCTTCATAGCACTGGTAAATTTCATTGACTGGGTGAAAAGAAAAAAGATCTCCTTGGCTGACCAAATTCTCACTGCTCTGGCAGTCTCCAGAGTTGGTTTGCTCTGGGCGTTATTATTAAATTGGTATTTAACTGAGTTGAATCCAGCTTTTTATAGTGTAGAGTTAAGAATTACTTCTTATAATGCCTGGGTTGTAACTAACCATTTCAGCATGTGGCTTGCTGCTAGCCTCAGCATATTTTATTTGCTCAAGATTGCCAATTTCTCCAACCTTAGTTTTCTTAATTTAAAGAGGAGAGTTAGGAGTATCATTCTGGTAATACTGTTGGGGTCTTTGTTATTTTTGGTTTGTCATCTTCTTGCGGTAAACATGGATGAGAATATGTGGACAGAAGAATATGAAAGAAACATGACCGGGAAGATGAAATTGAGGAATGCAGCACACCTTTCATATATGACTGTAACTACCCTGTGGAGCTTCATACCCTTTATGCTGTCCCTGATATCTTTTCTGATGCTAATCTTTTCTCTGTGTAAACATCTCAAGAAGATGCAGCTCCATGGTGAAGGATCTCGAGATCCCAGCACCACGGTCCACATAAAAGCTTTGCAAACTCTGATCTCCTTTCTCTTGTTATGTGCCATTTTCTTTCTATTCCTAATCATTTCGGTTTGGAGTCCTAGGAGGCTGCAGAATGAACCAGTTTTCATGGTTTGCAAGGCTGTTGGAAATATATATCTTTCTTTCGACTCATTCGTCCTAATTTGGAGAACCAAGAAGCTAAAACACATCTTTCTTTTGATTTTATGTCAGATTAGGTGCTGA

>Macaque_T2R25--Intact

ATGGCCACCGAAATGGACAAAATCTTTCTGACTCTGGCAACAGTGGAATTCATCATCGGCATGCTGGGGAATGTGTTCATTGGACTGGTAAACTGCTCTGAAGGGATCAAGAACCAAAAGGTCTTCTCAGTTGACTTCATCCTCACCTGCTTGGCTATCTCCACAATTGGTCACCTGTTGGTGATACTGTTTGATTCATGTGTAGTGGGACTTGCTCCACATTTATATGCCACAGATAGAGTACGAAGACCTGTTACTATGCTTTGGCACATGACTAATCACTTGACCACCTGGCTTGCCACCTGCCTGAGCATTTTCTATTTCTTTAAGATAGCCCACTTCCCCCACTCCCTTTTCCTCTGGCTGAGGTGGAGGATGAACAGAGTGATTGCTATACTCCTTACATTGTCTTTGTTCTTACTGATTTTTGACTGTTTAGTGCTAGAAATGTTTATTGATATCTCACTGAATATAATAGATAAAAGTAATCTGACTTTATACTTAGATGAAAGTAAAACTCCCTATGATAAACTCTCTCTGTTAAAAATTCTTCTTAGCTTGAACAGTTTTATCCCCTTTTCTCTGTGCCTGACCTCATTGCTTTTTTTATTTCTCTCCTTGGTGAGACATACTAGAAATTTGAAGCTTAGTTCCTTGGGCTCTAGAGACTCCAGCACAGAGGCCCACAGGAGGGCCATGAAAATGGTGATGTCTTTACTTTTCCTCTTCATAGTTCACTTTTTTTCCTTACAAGTGGCAAATTGGACATTTTGCATATTGGGGAACAACAAGTACACACAGTTTGTCACGTTAGCCTTACATGCCTTTCCCTCGTGCCACTCATTTATTCTCATTCTGGGAAACAGCAAGCTGCGACAGACAGCTGTGAGGCTACTGTGGCATCTTAGGAACTATACAAAAAGACCAAACCCTTTACCTTTGTAG

>Macaque_T2R26--Intact

ATGCTAGAGTCACACCTGATTATCTATTTTCTTTTTGCAGTGATACAATTTCTTCTTGGGACTTTCACGAATGGCATCATTGTGGTGGTGAATGGCATTGACTTGATCAAGCACAGAAAAATGGCTCCGCTGGATCTTCTTCTTTCTTGCCTGGCGGTTTCTAGAATTTTTCTGCAGTTGTTCATCTTCTACATTAATGTGGTTGTTATCTTCTTGATAGAATTCATCACGTGTTCTGCGAGTTGTGCATTTATCGTATTTGTAAATGAATTGGAACTTTGGCTTGCCACATGGCTCGGCGTTTTCTACTGTGCCAAGGTTGCCAGCGTCCCTCACCCACTCTTCGTCTGGTTGAAGATGAGGATATCCAAGTTAGTCCCGTGGATGATCCTGGGGTCTCTGCTATATGTATCTGTGATTTGTATTTTCCATAGCAAATATACAGGGTTTATGGTCCCGTACTTCTTAAGGAACCTTTTCTTCCAAAATGCCACAATTCAAATAGAAGTTAAACAGGCTATACAGATTTTCTCTTTTGTTGCTGAGCTCTTAGTGCCATTACTTATCTTCCTTGTTGCTGTTCTGCTCTTGATTTTCTCTCTGGGGAGGCACACCCGGCAAATGAGAAACACAGTGGCTGGCAGCAGGGTTCCTGGCAGGGGTGCCCACATCAGTGCGTTGCTGTCCATCCTGTCCTTCCTGATCCTCTACATCTCCCACTACCTGATAAAAGCTTTTCTCTCTTCTCTAAAGTTTCATGTCAAAAGGTTCGTCTTTCTGTTCTGCATCCTTGTGATTGGTACATACCCTTCTGGACACTCTCTCATCTTAATTTTAGGAAATCCTAAATTGAAACAAAATACAAAAGAGTTCCTCTGCCACAGTAAGTGCTGTCAGTGA

>Macaque_T2R3--Intact

ATGATTAAACTCTGCGATCCTGCAGAAAGTGAATTGTCGCCATTTCTCATCACCTTAACTTTAGCAGTTTTACTTGCTGAATACCTCACTGGTATCATTGCAAATGGGTTCATCACGGCTATACATGCAGCTGAATGGGTTCAAAATAAGTCAGTTTCCACAAGTGGCAGGATCCTGGTTTTCCTGAGTGTATCCAGAATAGCTCTCCAGAGCCTCATGATGTTAGAAATTACCATCAGCTCAACCTCCCTAAGTTTTTATTCTGAAGACGCTGTATATTATGCATTCAAAATAAGTTTTATATTCTTAAATTTTTGTAGCCTGTGGTTTGCTGCCTGGCTCAGTTTCTTCTACTTTGTGAAGATTGCCAATTTCTCCTACCCCCTTTTCCTCAAGCTGAGGTGGAGAATTTCTGGATTGATACCCTGGCTTCTATGGCTGTCTGTGTTTATTTCCTTCAGTCACAGCATGTTCTGCATCAACATCTGCACTGGGTATTGTGACAATTCTTTCCCTATCCACTCTTCCAACTCCACTGAGAAAACATACTTCTCTGAGATCAGTGTGGTCAGTTTGGCTTTTTTCTTTAACCTAGGGATTGTGATTCCTCTGATCATGTTCATCCTGGCAGCCATTCTGCTGATCCTCTCTCTCAAGAGACACACCCTACACATGGGAAGCAATGCCACAGGGTCCAAGGACCCTAGCATGGAGGCTCACATTGGGGCCATCAAAGCTACCAGCTACTTTCTCATTCTCTACATTTTCAATGCAGTTGCTCTGTTTATCTACCTGTCCAACATGTTTGACATCAACAGTCTGTGGAATACTTTGTGCCAGATCATCATGGCTGCCTACCCTGCCAGCCACTCAATTCTACTGATTAAGGATAACCCTGGGTTGAGAAGAGCCTGGAAGCAGCTTCAGCACCGACTTCATCTTTACCCAAAACAGTGGACTCTGTGA

>Macaque_T2R4--Intact

ATGAATGGAGATCACATGGTTCTAGGATCTTCGGTGACTGACCAGAAGGCCATCATCTTGGTTATCATTTTACTCCTTTTGTGCCTGGTAGCAATAGCAGGCAATGGCTTCATCACTGCTGCTCTGGGTGTGGAGTGGGTGCTACGGGGAACGTTGTTGCCTTGTGATAAGTTACTGGTTAGCCTAAGGGCCTCTCGCTTTTGTCTGCAGTGGGTGGTTATGGGTAAGACCATTTATGTTTTGCTGTATCCGACAGCCTTCCCATACAACCCTGTACTGCAGTTTCTAGCTTTCCAGTGGGACTTCCTGAATGCTGCCACCTTGTGGTTCTCTAGCTGGCTCAGTGTCTTCTATTGTGTGAAAATTGCCACCTTCACCCACCCTGTCTTCCTCTGGCTAAAGCACAAGTTGTCTGAGTGGGTACCATGGATGTTCTTCAGCTCTGTAGGGCTCTCCAGCTTCACCACCATTCTATTTTTCATAGGCAACCACAGTATATATCAGAATTATTTAAGGAACCATCTACAACCTTGGAATGTCACTGGCAATAGCATATGGAGCTACTGTGAGAAATTCTACCTTTTCCCTCTAAAAATGATTACTTGGACAATGCCCACTGCTGTCTTTTTCATTTGCATGATTTTGCTCATCACATCTCTGGGAAGACACATGGAGAAGGCTCTCCTTACAACCTCAGGATTCCGAGAACCCAGTGTGCAGGCACACGTAAAGGCTCTGCTGGCTCTCCTCTCTCTTGCCATGCTCTTCATCTCGTATTTCCTGTCACTGGTGCTCAGTGCTGCAGGTATTTTTCCACGTCTGGACTTTAAATTCTGGGTGGGGGAGTCAGTGATTTATCTGTGTGCAGGAGTTCACCCCATCATTCTGCTCTTCAGCAACCGCAGGCTGAGAGCTGTGCTGGAGAGGTGCCGTTCCTCGAGGTGCCGGACACCTTGA

>Macaque_T2R5--Intact

ATGGCAACCATGAACACAGATGCCACAGACAAAGACATATCCAGGTTCAAGGTCATCTTCACCTTGGTGGTCTCCGGAATAGAGTGCATCACTGGCATCCTTGGGAGTGGCTTCATCACGGCCATCTATGGGGCTGAGTGGGCCAGGGGCAAAACACTCCCCACTGGTGACTGCATTATGTTGATGCTGAGCTTTTCCAGGCTCTTGCTACAGATTTGGATGATGCTGGAGAACATTTTCAGTCTGCTATTCCGAATTGTTTATAACCAAAACACAGTGTATATCCTCTTCAAAGTCATCACTGTCTTTCTGAACCATTCCAATCTCTGGTTTGCTGCCTGGCTCAAAGTCTTCTATTGTCTTAGAATTGCAAACTTCAATCATCCTTTGTTCTTCCTGATGAAGAGGAAAATCATAGTGCTGATGCCTTGGCTCCTCGGGCTGTCAGTGTTGGTTTCCTTAAGCTTCAGCTTTCCTCTCTCAAAAGATGTCTTCAATGTGTATGTGAATAGCTCCATTCCTATCCCCTCCTACAACTCCACGGAGAAGAAGTACTTCTCTGAGACCAATATGGTCAACCTGGTATTTTTCTATAACATAGGGATCTTCATTCCTCTGATCATGTTCATCCTGGCAGCCACTCTGCTGATCCTCTCTCTCAAGAGACACACCCTACACATGGGAAGCAATGCCACAGGGTCCAGGGACCCCAGCATGAAGGCTCACATAGGGGCCATCAAAGCCACCAGCTACTTTCTCATCCTCTACATTTTCAATGCAGTTGCTCTATTTCTTTCCATGTCCAACATCTTTGACACTTACAGTTCCTGGAATATTTTGTGCAAGATCATCATGGCTGCCTACCCTGCCGGCCACTCAATACAACTGATCTTGGGCAACCCTGGGCTGAGAAGAGCCTGGAAGCGGTTTCAGAGCCAAATTCCTCTTTACCTAAAAGGGCAGACTCTGTGA

>Macaque_T2R6--Intact

ATGCAAGCAGCACTGAGGGCCTTCTTCATGTTGCTCTTTAGCCTGCTGAGTCTTCTGGGGATTGCAGCGAATGGCTTCATTGTGCTGGTGCTGGGCAGGGAGTGGCTTCGATATGGCAGGCTGCTACCCTTGGACATGATCCTCCTTAGCTTGGGTGCCTTCCGCTTCTACCTGCAGTTGGTTGGGATGGGGCACAACTTCTATCACTCTGCCCATGTGGTCCAGCGCTCTGGGGTTCTCACTCAACAGTTCTTCCATCTACACTGGCACTTCCTGAACTCAGTCACCTTCTGGTTTTGCAGCTGGCTCAGCGTCCTGTTCTGTGTGAAGATCGCTAACATCACCCACCCCACCTTCCTGTGGCTGAAGTGGAGGTTCCCAGGGTGGGTGCCCTGGCTCCTGTTGGGCTCTGTCCTGATCTCCTTCATCATAGCCCTTCTGTTGTTTTGGGTGAACTACTCTGCATATCAACAATTTTTAATTAGAACATTTTCTGGGAACATGACCTACGAGTGGAATGCAATGACAGAAATTTACTATTTCCCGTTCGTGCAACTGGTCATCTGGTCAATTCCTTGTTCTGTTTTTCTGGTCTCAATTATGCTGCTCATTAATTCTCTGAGGAGGCATACTTGGAGAATGCAGCACAACAGCCACAGCCTGCAGGACCCCAGCACCCAGGCTCACACCAGAGCTCTGAAGTTCCTCATCTCCTTCCTCATTCTTTATGTTCTGTCCTTTCTGTCCCTGATCATTGATGCCACAAAATTTATCTCCATGCAGAACGACTTTTACTGGCCATGGCAAATTGCAGTCTACCTGAGCGTGTCTGTCCATCCCTTCATTCTCATCTTCAACAACCTCAAGCTTCAAAGTGTGTTCTGGCAACTCCTGCTGTTGGCAAGGGGCTTCTGGGTGGCCTAG

>Macaque_T2R7--Intact

ATGCTTTGGTTATTCCATTTCTCTGCTATTATTGCCTCAGTTATTTTAAATTTTGTAGGAATCATTATGAGTCTGTTTATTACAGTGGTCAATTACAAAACTTGGGTCAAAAGCCATAGAATCTCCTCTTCTGAAAGGATCCTGTTCAGCTTGGGCATCACCAGGTTTTTTATGCTGGGACTATTTCTGGTGAACACCATCTATTTCGTCTCTTCAAATAAGGAAAGGTCAGTCTACCTGTCTGCTTTTTTCGTGTTGTGTTTCATGTTTTTGGACTCAAGCAGTCTCTGGTTTGTGACCTTGCTCAACAGCTTGTACTGTGTGAAGATTACCAACTTCCAACACTCAGTGTTTCTCCTGCTGAAGCGGAATATCTCCCCAAAGATCCCCAGACTGCTGCCGGCCTGTGTGCTGATTTCTGCTTTCACCACTTGCCTGTATATCACGCTTAGCCAGGCATCACCTTTTCCTGAACTTGTGACTAAGAGAAATAACACATCCTTTAATATCAGTGAGGGCATCTTGTCTTTAGTGGTTTCTTTTGTCTTGAGCTCATCTCTCCAGTTCATCATTAATGTGACTTCTGCTTCCTTGCTAATATACTCCTTGAGGAGACATATACGGAAGATGCAGAAAAATGCCACTGGTTTCTGGAATCCCCAGACGGAAGCTCATGTAGGTGCTATGAAGCTGATGATCTATTTCCTCATCCTCTACATTCCATATTCAGTTGCTACACTGGTCCAGTATCTCCCCTTTTATGCAGGGATGGATATGGGGACCAAATCCATTTGTCTGATTTTTGCCACCCTTTACTCTCCAGGACATTCTGTTCTCATTATTATCACACATCCTAAACTGAAAACAACAGCAAAGAAGATTCTTTGTTTCAAAAAATAG

>Macaque_T2R8--Intact

ATGCTGAGTGCTGGCCTAGGACTGCTGATGCTGGTGGCCGTGATTGAATTTCTCATCGGTTTAATTGGAAATGGAATCCTTGTGGTCTGGAGTTTAAGAGAATGGATCAGAAAATTCAGCTGGTCCTCATATAACCTCATTATCCTGGGCCTGGCTGGCTGCCGGTTTCTCCTGCAGTGGCTGATCATTTTGGACTTAACCTTGTTTCCCCTTTTCCAGAGCAGCCGTTGGCTTCGCTATCTTAATGTCTTCTGGGTCCTGGTAAGCCAGGCCAGCTTGTGGTTTGCCACCTTCCTCAGTGTCTTCTATTGCAAGAAGATCACCACCTTTGATCGCCCTGCCTACTTGTGGCTGAAGCAGAGGGCCTATAACCTGAGTCTGTGGTGCCTTCTGGGCTACTTTATAATCAGTTTGTTACTTACAGTCCAAGTTGGCTTAACGGTCCATCATCCTCCCCAAGGAAACAGCAGCATTCGTTATCCCTTTGAACACTGGCAGTACCTGTATGTATTTCAGCTCAATTCTGGAAGTTATTTGCCTTTAATGGTGTTTCTTGTTTCCTCTGGGATGCTGATTATCTCTTTGTATACACACCACAAGAAGATGAAGGTCCATTTAGCTGGTAGGGGGGATGCCCGGGCCAAGGCTCACATCACTGCCCTGAAGTCCTTGGGCTGCTTCCTCTTACTTCACCTGGTTTATATCGTGGCCAGCCCCTTCTCCATCACCTCCAAGACTTATCCTCCTGATCTCACCAGTGTCTTCATCTGGGAGACACTCATGGCGGCCTATCCTTCTCTTCATTCTCTCATGTTGATCATGGGGATTCCTAGGGTGAAGCAGACTTGTCAGAAGATCCTGTGGAAGACAGTGTGTGCTTGGAGATGCTGGGGCCCATGA

>Macaque_T2R9--Intact

ATGATAACTTTTCTACCCATCATTTTATCCACTCTAGTAGTGGTTACATTTGTTATTGGAAATTTTGCTAATGGCTTCATAGCACTGGTAAACTCCGTTGAGTTGGTCAAGAGACAAAAGATCTCCTTTGCTGACCAAATTCTCACTGCTCTGGCGGTCTCCAGAGTTGGTTTGCTCTGGGTATTATTATTAAATTGGTATGCAACTGTGTTGAATCCAGCGTTTTATAGTGTAGAAGTAAGAACTACCACTTACAATGTCTGGGCAGTAACCAACCATTTCAGCAACTGGCTTGCTACTAGTCTCAGCATATTTTATTTGCTCAAGATTGCCAATTTCTCCAACCTTATTTTTCTTCACTTAAAGAGGAAAGTTAAGAATGTCATTCTGGTGATGCTGTTGGGGCCTTTGCTCGTTTTGGCGTGTCATCTTTTTATGGTAAACATGAATGAGATTGTACGGACAAAAGAATATGAAGAAAACATGACTTGGAAGTACATATTGAGGAATGCGATTTACCATCCAGGTATGACTGTAACCACGCTACAGAACTTAGTACCTTTCACTCTGACCCTGATATCTTTTCTGCTGTTAATCTGTTCTCTGTGTAAACATCTGAAGAAGATGCAGCTCCATGGCAGAGGATCTCAAGATCCCAGCACCAAGGTCCACATAAAAGCTTTGCAAATTGTGATCTCCTTCCTCTTGTTATGTGTCATTTACTTTGTGTCTGTAATTATATCAATTTGGAGTTTTGAGAGTCTGGGAAACAAACCTGTCTTGATGTTCTGCCAAGCTATTAGATTCAGCTATCCTTCAGCCCACCCATTCATCATGATTTGGGGAAACAAGAAGCTAAAGCAGACTTTTCTTTCAGTTTTGTGGAACGTGAGGTATTGGGTGAAAGGATAG

>Opossum_T2R1--Intact

ATGACACGTCTATTTTTAATTATCCAATTAATTTTGGGAGTGGTCCAATTTCTTACAGGAATTGTGGCGAATGGAATTATCGTGATAGTGAGTGGCATTGAGTGTATCAAGAGGAAAAAAGTGACTGCATATGACCTCCTTCTGATCAGTCTGGGAATCTTCAGAATTTTTTTACAAGCTTTAATACTTACATGTCACATGATATTTGTCTTTACTCTAAATATATATGTAGAAAAGGAAGCATTTTTATTCTTTATTTTTGTAAATGAAGTCAATCTTTGGCTTGCTACCTACCTTTGTCTCTTCTACTGTGTTAAGATTGCCAACATTTTTCACCCCTTCTTCCTCTGGTTGAAGATGAGGATCTCTAGGCTTGTACCATGGCTGATTCTGGGATCACTGCTATTTTCACTTGCCCTTTCTGTATGCTATTTACTTCTCTATTGGCCTGAGGCCAAAGAGGAGATAAGAAGATATTTTTCAGGAAACCTCACTGCCTCCAATTTTTTATCTAGTCTTTTCCCAGTTCCAATTCTTGTGGTGGGACTGATAGTGCCATTAGTCATATTTGATGCTTCATTGTTTCTGATGATCTATTCTCTGTGTAGACATACCAGAAAAATGAAAAGCATGGCAACTGGCAGAGACTTAAGCACTGAAGCTCATATCCGTGCTGTGAAATCTGTGTTCTCCTTCTTCATCCTGTATACCTCCTATTATATGGGGATAATAATATCATTATCTAGAACTTCCTCACAGAATGAATTCTTCATGTTCATTTGTTTTTTTGTGGCTGCTGAATATCCTTCTGGACACTCCATCATCTTAATTCTAGGAAATCCAAAGTTGAAGCATTATGCAAGAAAGTTTCTGCTTTGTGCCAAGTGCTTGCTGGGAGGAGATTCAACAGGAGTATACAAACCCAATAGAACATGCTGCTTCTAA

>Opossum_T2R25--Intact

ATGCCAAGTGGCGGGGACAATATTTTGTTGGTTCTGGTAGTTGGACAGTTCTTAATGGGGATTCTGGGGAACGGATTCATGATATTGGTGAATGGCATTGATTGGATCAAGAGCAAGAAATTGGCAACAGGGGATATCATCTTGGTAAGCTTGGCCATCTCTAGAATTGGTATGTTAAGCACATTAACATGTGTTAGCTTTCAATTAGTGTTTAATCCAGACGAATTACTAAATGAGGGAATTAATGTCAGTGAAGTTTTCTGGATCCTGACCTACCTTTCAAGTATTTGGTTTGCCACCTGTCTCAGTGTCTTCTACTTCCTAAAGATTGCCAACTTTTCCCACTCCCTCTTTCTCTGGCTGAAGTGGAGAATCAACCAGCTTGTCGGTGTGCTAATGGTGGGACCTTGGCTCTTTAGTATGACCATCAATCTTCCAATGTTGGAGAGAGCTTATAACAATGCTCTTATTAGAAGAAACAACACAAAAGTATACAGAGACTACCAGGTGAATGAGAGTGAATATATCACCATACAAATTGTTACCAACCTCTCGAGTCTCATCCCCTTGTTTCTATCTCTGGTCTCATTCTTGCTCCTTGTTTTTTCTCTCCGGAGACATATGCATCAGATGAAGCACAGTGCCACCGGATCTAGGGATCCCAGCATGGAGGCCCATGTTCGAGCCATAAAAGCCATACTCTCTTTCCTCATCTTTGTTGTACTCTTCTTTTTGGGATCATTCATCATCTTGTGGAACTTCTCAATTCCAGGAAAAGACTCAAATTTAAGGTGTGGAATACCATTGATGTGTCTCTGTCCCTCAGGTCACACACTAATCCTGATTCTGCACAATAGCAAGCTGAGACAGGCAGCTTTGAAGGTGTGGTGGCAGGTGAGAGACTGCCTGAAAGGAAGCAAATGA

>Opossum_T2R7A--Intact

ATGTTAAATGCATTGAAGACCATCTTTATAATTTTGATGCTTGGAGAGTTCATAATGGGGGTTCTGGGGAATGGATTCATTGGACTGGTGAATTTCATTGACTGGATCAAGAAAAGGAAAATCTACTTAGTTGATTTAATTCTCACCAGTTTAGCCATTTCCAGAATTAGTCTGTTGTGTATAATGATGATAGATGGCCTTGTAGGGGTGTTTTTTCCAGAAGAATATACCAAGAGAAAGTTCACAAGAATTTTAGATGGCTTCTGGACAATTTCCAACCTTTTAAATGTCTGGTTTGCTACTTGCCTTAGCACGTTTTACTGCTTGAAGATAGCCAGCTTCTCCCATCCTCTTTTCCTCTGGCTGAAGTTGAGAATTAACAGAGTGATTCCCGTAATTCTACTGGCATCTATATTCATTTCTGTGTTCACTAGCCTTCCAATAACAGAGATATTTAATGAAGATGTCAGGAGCCAGATAAACAAAAAACATAAGGAAAATAACACTTCACTTCTCAAAATGAGTAAAGGTGAATATGTTGCCACCCCCACTCTCCTTAATCTTGGGCTTCTCTTTCCCTTTACTGTGTCTCTGATCTCATTTTTCCTGTTAATTCTTTCCATGTGGAAACATACCCGGAAGATAAAACTCAATGCCGTGGATACCAGAGATGCCAGCACAGAGATCCATTTGAGAGCCATGAAAGCTGTGATCTCTTTCCTCATCCTCTTTGTCATTTACTGTTTGGCCATTCTCATAGCAACCTTTAGCTATGTTTTCCCAGACCAGAATCTAGCAATGATATGTGGGGAGATAACTGCAGTCATCTACCCAGCTGGCCACTCATTTATCCTGATTTTGGGAAACATCAAGCTTAGGAGGATATCCCTGAATGCACTACAACAGGCGCAGCATTGTCTCAAAGGAGGGAAATCCTTAGTGCCCTAG

>Opossum_T2R7B--Intact

ATGCCAAATGCAATGAAGACCATCTTTATGATCATGATTATTGGATTGTTCATGATGGGGATTTTGGGGAATGGATTCATTGGACTGGTGAATTTCACTGACTGGGTTAAGAGAAGGAAAATCTACTTGGTTGATTTCATTCTTACCGGTTTAGCCATCACCAGAATTGGTTTGTTGTTCTCGTTGCTACTAAATGGATTTTTAGTGGTTTTTCACCCAGAAGTATATGAGTATGCTAATATAATGAGAATTATGGATAACTTCTGGACAATTTCCAACTATTTAAGTGTCTGGTTTGCTACATGCCTCAGTATATTCTACTGCTTGAAGATAGCCAATTTCTCCCACTTTCTTTTCCTCTGGTTGAAATGGAGAATTAACAGAGTGATTCCCATAATTCTACTGGCATCGTTGTTCATGTCTGTATTCATTAACTTTCCAATAATAGAGAAATTTAATGAGGATTTCAGGAGCCTGGTAAGCAGGAGGAACAAGAGAAATACTCCAATACTCCAAATGAATAATTCTACATACTTTAACACTCTGGTTTTCCTCAATCTGGGAACTCTCTTTCCCTTTACTGTGTCCCTGATCTCATTTTTCCTGTTAATTCTTTCCATGTGGAGACATACCAGGAAGATAAAGCTCAATGCCATGAATGGTAGAGATCCCAGTACAGAGATCCATCTGAGAGCCATGAAAGCTATAATCTCTTTCCTCATCCTCTTTATCATTTACTGTTTGGCCTTTCTCATAGCCACATCCAGCTACTTTTTCCCAGAGAGTGAACTAGCAATGATATTTGGTGAGATAATTGCAGTCATCTACCCCTCAGGCCACTCATTTATCCTGATTTTGGGGAACATCAAGCTGAGGCAGACATCCTTGAGTGTACTACAGCAAATGAAAAATTGTCTCAAAGGAAGAAATCCTTAG

>Opossum_T2R62--Intact

ATGCCCAACCCAATTACTCTCTTCTTCATGATCTTCTTTCTCCTAGAATCTGTGATTGCAATTGTAGAAAACAGTTTCATCTTCATGATACTGGGCAGGGAGTGGATGCGATGTCGGACCTTGCCCCCTGGTGATATTATCCTGGCCAGCCTGGGCATCTCCCGCTTCTTTCTGCAGTGGATGTCAATTTTTAGTAACTTCTTCACTTATTTCTTCCCGCTTAAACTAAGTGTATACTTTGGTACTTTTTGGACTTTTAGTAACATGACCACTTTCTGGTTCACCACTTGCCTTGGCGTTTTCTACTGTGTGAAAATTTCTGCTTTCACTCACCCAGTCTTCCTTTGGCTGAAGTGGAGAATTCCCCAAATGATCCACTTGTTCCTGTTTTGCTCCCTATTGACATCCATTCTTTTAACTACCCCACAAATTTTAACAACCTTTTTAACTTTCCAAGTGAAAGTTCCTGGGAATTCCTCAGAAAAGACCATTTTGGAAGATAAGATGTGTGCATACAGGATACATTATTTCATGCCTATGCAATTGTTTATTTTGTTACTTCCTTTCCTCTTCTTCTTAGTCTCTATCATCTTCCTCATTTCCACCCTGTGCCGACATTTGGGGAAGATGCAGCACCATAGCTCTAGTCTTCAAGATCCAAGTATGCAGGTCCATATCACAGCCCTAAAGTCCCTTTTCTTCTTTCTCATCCTCTACACATCATACTTTCTTCCTCTGATCATCAGCACCATCATACCCATTTCAGTAAGCAGTTCTTGGTTTTGGGTAAGGGAAGTAGTGACCTATGCTGGCATCTCCATCCATCCTGCCTTCCTGATCCTGAATAGTCCCAAACTGAGAGGGGCTCTGAAGAAGATATTTCATGTCCCAGAGGCTGCATAA

>Opossum_T2R4--Intact

ATGCTTCTGCCCATTTCTTTTTTCTTTCATATTTTCTTCATGATTGTTGCTCTAACTCAGCTCCTGACAGGAATTGTTGGAAATCTATTGATCATGATAGTCAATTGTAAGGCTTGGATCAAAAGTAGAAAACTTTCCTCCTTTGATAGGATCCTGTCTAGTCTCTCCATCACCAGATGCATAAGTCTGAGTCTAATGTTCCTGCATTTAATTTTCAGCTTAATTTTTCCAAAGAGCAAATACCCTGAGCTTATGGCCTTGATTATTTTGAACTCTTGGTTATTCTTGGATTCCTGCAGCCTCTGGATTGTGACCTTGCTCAACATCTTTTATTGTGTGAAGATTGCCAACCTCAACCATTTATTGTTTCTCTGGATAAAGCGAAATCTATCCTTGAAGACACCCTGGCCATTGCTAGCATGTCTACTGGTTCCCATCATTTCAAATTTCCTACAAATTTTGCTCAACAAAATCTTTCAGTTAGTTCATCCCACTCAGGAGAATGTGAGAAACAGCACCATGGTCAACATCAATGGCTCCATCTTCCTTTTGATGGCTCCTGTAATACTGAGCTTCAGCCTCCCGTTCATCATCAATTTGGTTTCCTGTTCCCTGTTAATCTCTTCCCTGAGGAAGCACATGCTGGAGATGCAGAAAAATGCCACCAGCTTTTGGAACCCTCAGACCAAAGCTCATGTAGGTGCTATGAAAGTTATGATATATTTCCTCATTCTCTATGTTCCTTACTCCATTGCTCAACTGGTTATCTTTCTCCCTTCCTTTGGGATTAGAAATAATTGGATCAGAAGCATATTGATAATAGTCAATTGTACTTACTCTCCAGGACATACTATTTTCATTATCCTATTGCATCCTAAACTCAAAGCAAGAATAAAGAGCATTCTATGGTGCAATTGA

>Opossum_T2R41--Intact

ATGGTAGCAGCACTCATTATCTTCTCTATGGTTCTCTATGCCCTATTGTGCCTTCTGGGGATCATGGCTAATGCCTTCATTGTTGTGGTATTGGGAAGGGAGTGGGTTCGATGCCACCGGCTGTCTCCTTCTGACATGATCCTGATCAGTCTGGGCATCTCCCGCTTCTGCCTGCAGTGGGTTGGAATGATAAACTGCTTTTACTATTTTCTCCACAGGACACAATATAACACGGGGCTTGCCCGCCAGTACTTTGGGCTTTACTGGGATTTTCTGAATACGTCCACATTCTGGTTTGGCACTTGGCTTAGTGTCCTCTTTTGTGTGAAGATTGCAAACTTCACTCATCCCATTTTCCTTTGGCTGAAGTGGAGAGTCAATGACCTGATACCGTGGCTTCTACTAGTTTCCCTGCTAATCTCGTTCATTGTCACCATGCTATTTTTTGTGGGGAACAACATTATGTACCAGGCATTTTTGAAGGGGACATTCTCCGGGAACCTAACCTTATATGGCTTCGCTAAGAGACTAGAAATCCAGTATTTTCTCCCTTTGAAACTCATTGCTTTGTCTATCCCCTGTTCTATGTTCATAGTCTCAACAGTTCTTCTGATTGCCTCTCTGCGGAGACACTCCTGCAGAATGCAACACAGTGCCCGAAGGACCCAGGATCCAAGTGCTCAGGCTCACTCCAGAGCTCTGAAGTTTCTGGTTTCTTTCCTGGTCCTCTATGCTCTTTCGTTTCTGTCTTCAATCATTGACGCTGCTGTCTTTGCTTTGATTGAGAAGATCTGGTACTGGCCATGGCAAATTCTGATTTTCTTGTGTGTTTCAATTCATCCCTTCATCCTTATCTTTGGAAACTCCCAGCTAGGAGGAACTCTCAGGAAGCTCCTTCGGCTGCCTAAAACCTTCTGTAATGACAAGGGGACATCGTCTCCTTCCTAG

>Opossum_T2R2--Intact

ATGATCTCTCTGGCAGAAGTTTGCTACACCTCAGTCCTGATAGCCCTGTTCATTATGGGGACTATTGGAAATGGCTTTCTAGTGGTGGTGAATGGTAGTAAACTGATGGGAGACCAGAGACTGATTGGCATTGAGCTCCTCCTCCTATGCCTTGGGATGTCACGGCTTGGCCTGCAGATTTTGTTAACGTTTCAAGGACTCATCAGTGTCTTCTTTGCCAAGTTTTACCTGCACAATGTCTATGGATCTCTGTTCCTCTTTATCTGGATGTTTCTAAACTCCTGTAGCCTCTGGTTTGCCACCTGCCTTGGCATTTTCTACTGCCTTAAAATCTCTGACTTCACACATCCCTGCTTTTTGTGGCTGAAGTTCAGGGTCTTCAAATTGATGCCCTGGATGCTCCTGGGAAGCCTGCTGGCCTCTGTGGTCATTGCTGGGCTCTGTGCCTGTATGTTGGACTACTCTATCGCCTCCAACACAGACTGGGCCAAGAATGTCTCTCAGGCTGGTGCTGATTCTGTAAGCGTCATCATCAATGATGTGCTCCTTGTCAACTTCGCATTAATATGCCCTCCGTGTCTGTTTGTAATGTGTACGGTGATGTTATTTGTCTCTCTTTACAACCATACACACCGGATGCAAACCCAGTCCCTGGATTTGGGGAATCCCAACACCGAGGCCCACATCAATGCCTTGAGGACTGTGATCACATTTTTTTGCTTCTTCGTTTCCTACTTTGCAGCCCTGATGGTGAACCTGACGTTCATTGTGCCCTTCAAAAGCCACTGGTTCTTCTTCCTAAAATCTGTGATGGCAGCCTACCCCTCCGGCCATTCGGTGATCATCATCTTAGGCAATTCCCAGTATTGGCAGCTGTTCAAGAGGATCCTGCCTTGA

>Opossum_T2R40--Intact

ATGGCAAAAGTGACTACTGATTCCACAAATGATGATATATCTGAATCTGTCCTTTTGATCACCTTAATAGTGCCAGGAATTGAGTGCATCACAGGCATGATTGGGAATGGTTTCATTGTGGCCACGAATGCCATTGAGTGGTTCCAGAGCAAAAGACTCTCCACTAGTGATTTTACTTTGATGATTTTGAGTTGTTCAAGACTCTTGTTACAGTTCTGGTTGATGTTGGAAAATACTTACAGTTTATTATTCCCATTTTCTTATAATCAAAATATAGTGTATAAAACCTTCAAAGTCATCTTCATGTTCCTGAACTATTCCAATCTCTGGTTTGCTGCTTGGCTCAATGTCTTCTATTGCATCAGGATTGCCAACTTCACCCATCCCCTGTTCCTCAGGCTAAAGTGGAGAATCACTGGATTAATGCCCTGGCTCCTCTGTTCATCTGCTTTCATTTCTTTGTGCTACAGTCTTCCCATTTCCATGGATGTCTACAATGTTTTTGTTAATTCTTCCATTCCAGTTCCCTCCTCCAATACCACAGAGAATAAATTCATTACAGAGACCAATGTGGTTAACTTTGCTATCATGTATCACCTGGGCATTTTTATCCCTCTAATCATGTTCATCTTTGCAGCTACTCTATTGATCATCTCTCTCAAGAAACATACTCTCCAAATGAAGAATAATGCCACAGGTTCCAGAAATCCCAGCATGGAGGCTCACATGGGAGCAGTCAAAGCAATTAGTTCCTTTCTCCTCTTCTATGTCTTCAACTTTGTGGCTTTGCTCCTCTATATGGCCAATGTCTTTAAAAACAACAGCGTTGGAAATATTTTATGCAAAATTATCATTACTGCCTACCCTGCTGGTCATTCAATTCTACTGATCTTCAGCAATCCCAAACTAAGAAGAACCTGGAAGAAGCTTCAGCACAATGTTAGATTCCACCTAAAACATTAG

>Opossum_T2R56--Intact

ATGTTCAGAGGAGACATAAAGCTTGAAAATGGCGTGATTACCTTAATTGCCTTTTTTTTCTTCCTGTGTCTGATACTGATTGTGGAGAATGGCTATATCCTTGTAGTGCTGGGCAGGGAATGGGCACAGTGTGGGACACTGACTCCAGTTAATAAGATCTTGACCTGCTTGAGTGCCTCTCGCTTCCTCTTGCAGTGGGGGGTATGTGGAAAAAACCTGTATGTGTTGCTCTATCCGATGGTCTTCCCATACAACCCTGTACATCAGTACTTATCCTTCCATTGGGATTTCTTGAACTCTGTCTCTCTATGGTCCTCTGCTTTGCTCGGTGTCTTCTACTGTGTGAAAATCACCACCTTCACTGACCCTGTTGTCCTCTGGCTGAAATGGAGGATCTCTAGGTTTGTGACCTGGCTACTATTTGGCTCCTTTGGCATCAGCTTGTTTACTGCCTTCCTGTTCTTCATAGGTAACTATTATATCTATTATATCTACTCAGTGAGGAGTCTTCTATCTGAGAATTCTACTATGGAAAATTTGAGAAGACAATTGGAAACATATTACTTTTTCCCTCTGAAAGTAATTTCTTCATCAATTCCAGCAGCTATCTTCTTCATCTCTATGGTTTTCCTTATCACTTCCCTGGAAAGGCACAAAAAGAAGATGCTACATAGTGATTCTGGCCTCTGGGACTTCAGGTTCCAGGCTCACACCAAGGCTCTTAAGACCCTCATCTCCTTCTTCATTCTCTTTGCCTCCTATTTTTTTGCAATAATGGTAAATGCTTCAAACATCTTGCCCTCTTTGAAGTCATGGTACTGGGGATGGCAGGCTTTGATTTACCTTTGCATAGCAATCCACTCCATGCTTCTGATCCTGAATAACCCTAGGTTACTATGTTGGAGAAGAGCTGCTTGA

>Opossum_T2R39--Intact

ATGTGTGAAATCTGCAGCTCCCCAGAAGAGAAGCCCTCAATGTTTGTTATTATCATATCTTTCACTATTTCAGGAACAGAGTGCATCATTGGCATTACTGGGAATGGTTTCATTATGGCTATCAATGGGACTGAGTGGGTTCAGAACAGAAAGTTCTCCACAAGTGGTAGGATTTTGTTTTTCCTCAGTTTGTCTAGAATTGCTCTCCAGACCCGCATGATGATAGAAAACTTTGCCTCTTTAATATTCCTATCTTTTTACAAAGAAGACACTATCTATGAACTATTCAGAGTCATTTTCATGTTCTTGACTTTTTGCAGCCTCTGGTTTGCTACTTGGCTTAGCATTTTCTATTTTGTAAAGATTGCTAATTTCAACAACCCCCTGTTCCTCAAACTAAAGTGGAGAATCACCAGATTGATGCCCTGGCTATTGTTGTCATCAGTGATCATTTCTTTCTGCAACAGTGTCCTCTTTTCTCTGCATGTTTACAATGCATCCTGCAGTGACTTAATCCTCAATCCATTCTCCAACTCCACAAAGAAGAAATACAACATTAATACCAATATGATTAACTTGGTTGTTATCTACAATGTAGGAATGTTCATCCCTCTCATCATGTTCATCCTTGCAGCCCTCTCATTGATCATTTCTCTCAAAAGACACACCCTGCAGATGGAGATTAATACCATAGGCTCCAGGAATCCCAGTATGGAGGCTCACATGGGAGCCATCAAAGCCATAAGTTCTTTCCTCATTCTCTACATTTTCAATTTTGTTGCTTTGCTCCTCTTCATGTCTAACATTTTTGATGAAAACAGTTTCTGGAATATATCCTGCAAAATCATTATGGCTGCCTATCCTTCTGGTCACTCAATATTATTAATCTTGGGCAATCCCAAACTAAGGAATGCATGGGGAACGTTTCAGCACCAAATGAAATTCTGCCTAAAAGGGAGGACTTCCTAA

>Opossum_T2R22--Intact

ATGTCAAATGCAGTGGAGAAAATGTATGTGGCTGTAGCATGTGGAGAGGTCCTACTTGGAATTATGGCAAATGGCTTCATTGGAGTCATGAAGTGCATTGACTGTGTCAAGACCAGAAAAGTTTCCTACTTAAATTTTATTCTTACTGGCTTAGCCATCTCCAGAATTGGTCTGCTAGGAATCATCACATCACATGTCTTTGCCATCTCATTCTATCCTTCTTCGTATTTGTTAGGCAACATGAAAAACTCCAATGCGTTATGGAGTTTGGCCAACAATTCCAGTACCTGGTTTTCTGCCTGCCTTAGTGTCTTTTACTTGCTGAAAATTGCCAACTTCTCCCATCCCATCTTCCTCTGGCTGAAGTGGAGGATAAACAAGGGAGTCCTCAAGGTGCTTCTAGGTTGCTATTTCCTCTCCTTTCTTCTTTCCCTTATGGTAACATTGATGAGTGATGTCTTAGAAGTAACTGTTAATTTAGAAAGCAAACTAAACTTGACTCAGAAGATACAAGTACATAAAAACCCAATTTTTGCTGTTATGATGATGTTCAGTGTGGGGGGAATCTTGCCTTTTGCTCTGTCTCTGATCTCCTGTTTCCTGCTGGTCCTATCACTATGGAGGCACACTCAGCAGATGCAACTTAATGCCACAGGTTCCAGAGATCCTAGCATAGAGGCTCATGTGAGGGCCATGAAATCCATGGTCTCTTTCCTCTTCCTCTTTGTTGGGTACTATGTAGGCATGTTCTCAATAATCACAAATGGCTCGATATTAGAAAGGAAGACGTCTGTACTGTTTACTTTGATGGCTATGATCATGTACCCATTGGCCCACTCCATTATCATTATTGTTGGACACAACAAACTAAGACTCACTGCTCTAAGGGTGCTATGGAAGTTCAGAGTCTATTTCAAGTGCTTTGGAAAATGCTGA

>Opossum_T2R20--Intact

ATGCCATGTTTAAGTGAAATTGTGGTCTTTGTTGTGACCTTCACTATGTTCTTTCTGGGTGCTTGGATAAATGGCTTCATCGTATTGCTGCACTGTATCACTTGGGTCAGAAATAAGAAAATTTCTTTTTCTGACTTCATCATTCTGAATCTGGCTCTCTCCAGGATCATCCTGCAAGGGACACCAGTGGTGGATATTGTGTTAAAGATATGTTATCCCCATTTACGTCACAGAGGTTTATATGTGAAAATTCTAGATATTTTCTGGTGGTTTACTAACAACTTAAGCATCTGCCTGACCACCTGCCTCAGTGTCCTCTACTGCCTGAAGATTGCCAACTTCTCCAACCAGACCTTCCTCTGGCTCAAGAGGAGGGTTTCTCATGTGGTTGTCTGTATTCTTGTGGGGTCTGTGCTCTATTCCTTCACCATATTGTTACTGATTCTAAAATACCACGTCTATTCTACCATGAATCAAATGATACTCTCAAGAAACCACACTGAAGAAATCAAAAGAATAAAAATGCACTATTTTCTCTTACATTTTCTTGGTACTCTCTGGTCAATCCTTCCACTCTCCTTGTCCCTGGTCTCCTCTGTCCTGCTCATTCTCTCCCTGGTGAGACATACCAGGCAGATGAAATATCATTCCATTGGCACCAGAGACCTCAGCACCATGGCCCATGTGAGAGCCACCAAAGTCATCCTCTCTTCTTTCATACTCTTCATTGGATACCTCCTCGCCTTCTTTCTTGCCATGTCTTCTTATTTATTTCTAACCATCAAGATGTCAGAGATGATTGCCTTACTGATTTTCACAATTTATCCCTCTATTCAAACCTTCACTCTAATCCTGGAAAATCAGAAGCTGAAGCAGGCATTCCTCAGGATGTTCCAGGTCAAAAAGTGGAGACTAAAATGTTAG

>Opossum_T2R3A--Intact

ATGAAGAGTTTATTCCAAAACTTTTTTTTGCTCATGGCCTTCATTGAGTTCATCCTGGGGATTTTAGGGAATGGCTTTCTTGGGCTTATCAATTGCATCCACTGGGTCAAGAGCAGCAAGATCTCCTTATCTGACTTAATTATTATGAATCTAGCTTTCTCTAGGATCATTCTGCAATGTGTGATAAGTTTGGATGGTGTTGTCCTGGTGTTCTATCCTAGTATCTATGATTCAGAGAAATTCATTCAAATTCGTGAAATTGGCTGGACCTTTAGTAACTATTTAAGCATTTGGCTGGTCACCTGCCTCAGTGTCTTCTACTGCCTGAAGATTGCCATTTTCTCCCACCCTGCCTTCCTCTGGCTTAAATGGAGGGTTTCCCAGGTAGTTGCCTGGATTCTGTTCTTTTCTGTGCTCTTCTCTCTCTTCAATATAGTGTCATTGATCCGAAAATTCAGTATGTATTCTGACTTCCTTAAAATGAAGCACATTGAAAACTGCACTGAAATTAGCAGAAGAAAAGAAATGGAATATTATAATGCCCAGATTATTGGTCTCCTGTGGTCAGTGATACCTTTCATTATATCACTGATCTCCTATTTCCTACTTATCCTCTCCCTGAAGAGGCATACCAGAATGCTACAGCACCACATCACTGGCCCCAGAGATATGAGTTCTGAAGCCCATGTGAAAGCCACCAAAGTAATGTTTTCCTTTCTCTTACTTTTTGTCTTTTATCTTATCATTATGTACATTGGGTCATCAAACTTCTTCTTGACAGACACCAAGTTATCAATAATGATTATAGAGTTGGCTGCCCCTCTCTATCCCTCTGTGCACTCATTCATCCTCATTCTTCAAAACAAAAACCTGAAACAGGAGTTTTTGAGGCTTCTGTGGTTGAAAAAGGGCTGCATAAAAGATGGAGTCACATCCTAA

>Opossum_T2R3F--Intact

ATGTCAAATTTAGAGGTAATGGTTATGTTTGTGGTCTCTATCGAGCTCTTTCTGGGAACTTCGCTAAATGGCTTCATTGTGTTGCTGCATTGTATCATTTGGGTCAAGACCAGGAAAATTTCTTTGTCTGACTTCATCATTCTGAATCTGGCTCTCTCCAGGATCATTCTGCTTGGGATAATGATGTTTGATACTGTTTTATCCATATTCTATCCCCATTTACAAAACGTAGGCATATTGATGCAAATTATTGATATTTTCTGGATGTTTACCAATAACTTAATCATCTGCCTGACCACTTGCCTCAGTGTCCTCTACTGCCTGAAGATTGCCAACTTCTCCCACCAAGCCTTCCTCTGGCTCAAGTGGAGGGTTTCCCATGTGATTTGGGGAATTTGGGGGGGCTCTGGGCTCTACTCCTTCTTCAGCGCACTGGCAATGATTCTGAAATTTAGTGAATATGCTGACCTCAGTCAAATGAAACTCTCAGGAAATTACAGTGAAGAGGTCAAGAAAAAGCTAACTAACTATTATGTTTTACATATTCTTGGTCTCCTCTGGTCAGTTATTCCTCTCTGTGTGTCTCTGCTTTCCTCTGTCCTGCTCTTCCTCTCCCTGGTGAGACATACCAGGCAGATGAAGCATCATGCCAATGGCACCAGGGACCTCAGCACCATGGCCCATGTGAGAGCCACCAAAGTCATCCTCTCTTCTTTCATACTCTTCATTGGATACTTCCTAAACTTATTTCTTGCCATGTCTAGTCATTTCTTTCCAAGACCCAAGCAGGCAGTGATGATTTGGAGACTGTTTTTTATTGCTTATCCCTCTATTCAAACCTTTGTTCTAATCCTAGAGAACCGGAAGGTAAAACAAGCATTCATCAAGATGTTCCAGGTCAAAAAGTGGAGGCTGAAATGTACGCCTTGGGAACTTTCACCCAAGACTTGA

>Opossum_T2R38--Intact

ATGGCCCCTCTGGCCCTAGCCATCACAGTGTCCTATGAAGCTAAGTGGATATATTTTTCCCTCTCAATCCTGGAACTGGCAATAGGGATTCTTAGCAATGCGTTCATTGTGTTGGTGAATGCCTGGGATCTAGTGAGGAGGCACCAGCTGTCCAAATTTGACTCTGTCCTGCTATGCCTTGGCGCCTCTCGGATCTTCCTACAGATTTTGCTCTTTATGGATGCTCTTCACCTCACCCATTTTCAAGTGATGAGGGACCCACTGAGCATCAAGTACAAAACCGTCATCTTATTTGGGATGCTTGTCAACCAAACCAGCCTCTGGCTTGCCACCTGGCTTAGCATTCTCTACTGTGCAAAGATTGCCCGAGTCTCTCATGCCTTTCTAATATGGCTTAAAGGCTGGCTCCTCATGACCATCCCCCAGCTGTTACTAGGTTCCCTGATTTTCCCTTGGCTCAGTTTCTTTCCTTGTATATGGAAACATTTTAGTTTATCCTACTCCAACTCTACAGTTTCTTTCTCTGGGAACATCACAGAGAATAGCATAAAGGAGAGATTCATTTTTTTCCATTTCTTTCTTCTTTGCAATTTGGGCAATATCTTTCCCTTCCTGCTCTTCCTGGCATCTTCCAGCATCCTTATCATCTCCTTAGGAAGACACATGAGGACAATGACAGCTCAGACTACTGGCTCTGGAGACCCCAGCCTGGAGGCTCACATCATTGCCCTCAGGTCCTTGATTTCTTTCCTTTTGCTCTATGTAATAGGTTTTGTAGCTACCCTAGCGGCAGTCCCCTTCACCTTACTTATTTCCAGCAAGATTGGGGTGATGGTATGTGTGGGGATAATGCTCACTTCTCCCTCAGTACACTCGATCATTCTGATCCTCAGCAACAACAAGCTGAAAAGAGTTCTCAAAAGCATTCTGTACTGGCTTCGGTACTTTCTAAAGGCATGCATAGTATAA

>Opossum_T2R3H--Intact

ATGCAGAATTTAAGTGAACTTGTGGTCTTTGTTGTGGCCTCCATTGAGTTCTTTCTGGGTTCGTGGGTAAATGGCTTCATTGTGTTGCTGCACTGCATCACTTGGGTCAGAAATAAGAAAATTTCTTTTTCTGACTTCATCATTCTGAATCTGGCTCTCTCCAGGATCATCCTGCAAGGTATACCAATGGTGGGTGTTGTGTTAATGGTTTTTTATCCCCATTTACATCACAGAGGTTTATATGTGAAAATTCTAGATATTTTCTGGTTGTTTACCAATAACTTAAGCATCTGCCTGACCACCTGCCTCAGTGTCTTCTACTGCCTGAAGATTGCCACTTTCTCCAACCAGGCCTTCCTCTGGCTCAAGTGGAGGGTTTTTCATGTGGTTGTCTGGATTCTTGTGGGATCTGTGCTCTATTCTTTCACTATATTGACACTGATTTTAAAATACCACATTTCTTCTGACATGAGTCAAATGAAACTCTCAGTAAATTACACTGAAGAAGTCAAAAGAATAAAAACTCATTATTATATCTTGAATTTTCTTGGTACTCTCTGGTCAATCCTTCCACTCTCCTGGTCCCTGGTCTCCTCTGTCCTGCTCATTCTCTCCCTGGTGAGACATACCAGGCAGATGAAACATCATTCTATTGGCACCAGGGACCTCAGCACCATGGCCCATGTGAGAGCCACCAAAGTCATCCTTTCTTCTTTAATACTCTTCATTGGATACCACCTTGCCTTCTTTCTTGCCATGTCTACTTATTTCTTTCTAACCACCAAGATGTCAGAGATGATTTGGTCACCGATTTTTGTAATTTATCCCTCTATTCAAACCTTCACTCTAATCCTGGAAAATCAGAAGCTGAAGCAGGCATTCTTCAGGATGTTCCAGATCAAAAAGTGGAGACCAAAATGCTAG

>Opossum_T2R3J--Intact

ATGATGAATTTAGCCAAAATTGTATATTTGGTGTTGTTCATCATTGAGTTTATTCTGGGTTTTTGCTGTAATAGTTTCATAGGACTGGTGAATTGCATCAACTGCATAAAGAGCAAGAGAATGACTTCATCTGATCTAATTATTACAAGTCTAGCCCTGTCCAGAATATGCTTGCTTTGGGTACCTATAGTTAACATTTTACAACTACTCTATCCCAATTTATATGAGAAAAAGATATTAGGTATTATTGTCATAATCTGGATATTTATTAACCATTTAAGCACTGGGTTGGTCACCGGCCTCAGTGTCCACTATTGCCTGAAGATTGCCAGATACTCCCACTCTGCCTTCCTCTGGCTCAAGTGGAGAGTTTCCTGTGTAGTTATCTGGATTCTGCTGGGTTCTGTGCTTTTCTCTTTTTTTAGTGTAGCACCAGTAATCAAAGAATTAATTGTTTCCTTTAGCAGCAGACAAATGAAATTCATAGCAAACTGCACTGAAAACATTACAAAAAGTTATGCATTTATTTTTAGTGCTGTCTGGATGGTCATCCCTTTTGTGATAATTCTGTGTTCATCTCTCCTACTTATTCTCTCCCTGAGGAGACACACCCAGCAGATGAATAACAGTATCACTGGCTCCAGAGACCCAAACACTGAGGCACATTTAAAAGCTGCCAGAATCATTGTCTCCTTCCTCTTTCTCTACATATTATATTTTGTTGCCTTTTTTATTCTTATATTCAGCGACTACCTGCCAGACTATAATTTAGCATTGATGATTGGGGAAATGATTACAACTGCCTATCCTTCTGCACATTCATTCATCCTCATTTTGAGCAACAACAAACTGAAACAGGCATTCCTGAGGATGTTCCAGATACGGTGTGGGACTTGGGGGGACTTGGTGACCCTTTGCCTCACTGGTAAATCTGGAAAACAATAA

>Opossum_T2R3D--Intact

ATGGTGAATTTAAGCCAACTTGGGGTTTTATTTGTAGCCTCCACTGAATTGTTTCTGGGAACTTGGCTAAATGGATTCATTATGTTGTTGCACTGTATCACTTGGGTCAGGACCAGGAAAATATCTTTGTCTGACTTCATCATCCTGAACCTGGCTCTCTCCAGATTCATCCTGCAGGGGATACCAATAATGGATATTATTTTACAAGTTTTCTATCCCCATTTCCATGGCATAAGCATATTTATGCGAATTATTGATATTTTCTGGGTGTTTGCCAATAATCTTAGCATCTGCCTGACCACTTGCCTCAGTGTCTTCTACTGTCTGAAGATTGCCAACTTCTCCCACCAAGCCTTCCTCTGGCTCAAGTGGAGGGTTTCCCATGTGGTTATCTGGAGTCTTCTGGGCTCTGTACCCTATTCCTTCTTCAGCACACTGGCATTGATCCTGAAGTTTAATGTTTATTCTAACCTCAATCAAATAAAACTCTTAGGAAACTGCACTGAAGATGACAAAAGAAAGATAATTGGATATTTTGTCCTGTATATACTTAGTGCCCTCTACTCAATCATTCCCCTCTTCTTGTCCCTGGTCTCCTCTATTCTTCTCATCCTTTCCCTGGTGAGACATACCAGGCAGATGCAACATCATGCCAGTGGCACCAGGGATCTGAGCACCATGGCCCATGTGAGAGCCACCAAAGCCATCCTATCATCTCTCTTGTTCTTATTTGGATACTTCCTTGTCTTTTTTCTTGCTATGTCTAGTCATTTCTTGCCAGACCCCAAACTGGCAGGAACAACTAGCATACTGATTTTTGCTGCTAATCCCTCTATTCAAACTTTTGTTCTCATCCTGGAAAACCAGAAGCTAAAGCAAGCATTCCTCAGGATGTTCCAGGTCAAAAAGTGGAGGTTGAAATGTTAG

>Opossum_T2R24--Intact

ATGCAAAATCATGTAGAGAAAATCTTGTTGGTTCTGATAATTGGAGAGTTTGTAACAGGGGTTCTAGGGAATGGATTCATGGTATTGGTGAATTGCATTGACTGGGTCAAGAGTAAGAAACTATCAACTGTTGATCTCATCCTGGTTGTCCTGGGCATCTCCAGAATTGGTCTATTGTGCTTGATTAAATGGGATAGTTTTCTATTCTTATTCTTTTTGGATCAAACAGATGATGAGCATCAAAATTTTATTAGAGATTTATTTTGGATAATTACCCACCTTTCTAGTGTTTGGTTTGCTACCTCACTCAGCATTTTCTACTTACTAAAGATTGCCAACTTCTCCCATCCTTTCTTTTTGTGGCTGAAGTGGAGAATCAATCAAGTGGTCTACATACTCTTGGTGGGGCCCTTATTTGTCTATGTATCCTATCAATCCATAAAGTTAGTGGAACTTTACTCTGATAGGACCCTTAGTAGAAGAGAGAATGAGAGAAATGAATCTCAAGCGGTCCAAATGAATAGAAAGCATTACTTTATTGAAGAGGTGGGAATAAATATTCTAAATATTCCTCCTTTCATCCTTTCTATCATATCTTGTTTCCTGCTACTATACTCCTTATGGAAGCATTCCCAGAAGATGCAGCTCAAAGTCAAGACCTCCAGAGATCCCAGCACAGAGGCCCACAAAAGAGCAATGAAAGCCATGCTTTCCTTCCTTATCCTTATAATAGTTTATCATATCGGCATCCTCTTCACCTACAAGAACTTCTCGAAGCTAACTTCCATGTTTGGTATGGCACTAATGTCCCTTTATCCCACAGGACATTCACTGATATTGATTCTATGGAATAGCAAGTTAAAACATGCTGCGCTATTGGTGTGGAGGCAGCTGAAATGCTGTCTGAAATTAAATAAATCCTCCAATGTCTAG

>Opossum_T2R26--Intact

ATGGATTTGTTTTCTACAGACATTCAAGATCCCATAATGAAAATCTGTGTGATGCTGGCAGCTACAGAGTTTCTAGTGGGAGGGTTGGCCAATGGCTTCATTGGACTGGTCAACTTCATAGACTGGGTCAAGACTCGAAGAGTGTATTCAATAGACTTAATCCTCACTGTATTGGCCATCTCTAGGATTACTTTCTTGGGATCATTCCTATCTATAATCATAATGAATTTCTATTTAAATTTATATGCCACTGGTAAGGCGAGCTACCTAGAACCTCTTTGGAACTTGAGCAACCATGTAAGTTCCTGGTTTGGCACCTGCCTCAGCATCTTCTACTTCCTGAAGATCGCCAACTTTTCCCACCCTGCCTTTCTCTGGCTGAAGTGGAGGATTAACAGGGTGATTCTCAGTATAATGATTATCAGCTTCCTCATTGCCTTGCTTGTCAATCTTCCATTGACAGTGAAAATTAATCAGATTTATATAATCCTTGCTGAGCATAAAAATAAAGCAAATGGAACTCATAAGATGAAGATAAATAAAAGCCACATCCTTTTCTATTTGGTTTTCTACCACATGGAGGGATTTGTTCCCTTCGGTGTCTCTGTTATCTCCTGCCTTTTGTTAGTCTTCTCCCTGTGGAGGCATACCCAGCAGATCACAGCCTCCAGAGACTTCAGCACAAAGGTGCATAAGAAGACCATGAAATCCATGGTGTCTTTTGTCTTCCTCTATTTAATATACCACTTGGGCATCATCATGGCAAATCTGAGCTACGGGTTTTTTGAAAGTACTCTACCTGTGTTTTTATCAATGTTAATAGCTACCACCTACCCCTTGGCCCATTCCATTATTTTAATCAAGGAGAATAACAAGCTTAGGCAGGCCTTCCTGAGCATTCTATGGCAGCTTAAACACTGCCTCTAA

>Opossum_T2R29--Intact

ATGCCAAATAGTGGGGAAACCATCTCAATTGTTCTGGTAACTGGTGAATTTCTATTGGGCATTCTGGTGAATGGATTTATAGCAATGGTGAACAGTACCGACTGGGTCAGGAGCAAGAAACTGTTAACAAGTGACATCATCCTGGTCAGCCTGGCTATCTCCAGAATTGGACTTGTGAGTATTATAACGTGGAAGAGCTATTTGGTTATGAATGATATTTATGTATTCATCTTCGATGGAGTTTTAAGAACAATTGATATCTTCTTGGTACTGACTCATAGTTCAAGTATTTGGTTTGGCACTGTCCTCAGCATTTTCTATTTCTTAAAGATTGCTAAATTCTCTAATCCCTTCTTCCTCTGGATGAAATGGAGAATTGACAGGATGATCTACATGCTTCTTGGAGGGCCTTTGATAGTTTATTTGTTCATTCTCTTTTCAGTGATGAAGACAATGTATTGCTACAGTAGCAGCTTCCTTTTTAGCAATAAAAAGGGAAATGATTCCCAGGAGTTCCAAGTGAATGAACCTTTGTTCATCATATTCCAGCTTTTCTTTGGTTTTCTGAGTTTTATCCCTTTTACTCTGACATTATTCTCATTCTCTCTACTCATTCTCTCCCTGTGGAGGCATACTCGTCAGATGCAACTCAATGCCACGGGTTCCAGAGACCCTAAAATAGAGGCCCATGTCCGAGCCATGAAAGCTGTGTCTTCCTTCATCCTCCTCTTTTTATTGTACTATATAGGTTTATGTTTCAACTATGGGAGCTACCTGACAAATAGTAAGGTGTTTTTCCTGCTGAGTATGTCAGTCATGTTGCTCTATCCCCTTGGTCACTCACTGATCCTGATTCTGTGGAACAGCAAGTTGAAGAAGGTTGCCCTGAGGGTATGGTGGAAGATGAGGTGCTACCAGAAAGGAAATCATTTGTAA

>Opossum_T2R23--Intact

ATGTTAGAAAATGGGGAGATCTTAATGGTTCTGATAACTAGTGAATTTCTATTGGGTGTTCTGCTGAATGGATTTATAGTAGTGGTGAACTGCACCGACTGGATCAGGCACAAGAAACTGTTAACAAGTGATCTCATCCTGGTTGGCCTGGCTATCTCCAGAATTGGACTATTATGCACGATGATGTGGACTGCCTATTTATTTGTAAATGGTCTTTATGCAGTCATCGCAGATAGAGTCAGAGTAATTGACGTCTTCTTGGTGCTGACACACAGTTCAAGTATTTGGTTTGCCACTGTCCTCAGCATCTTCTACTTCCTAAAGATTGCTAACTTCTCTAATCCCTTCTTCCTCTGGATGAAATGGAGAATTAACAGGATCATCTACATGCTTCTTGGAGGACCCTTGGTAATTTCTTTTGCCTTTTGCTTTCCAATGATGGAGAGAATGTATTACTATGCTGACCACTTTTCAAGAGAAAGAGAGAGAAATGTTTCTCAGGAGGTCCAAGAGAATAAAAATAAGCTCATCATGTTCCAGATTCTCTTCACCTTGCTGGGTCTTATCCCTTTTGCTCTGACTCTAGTCTCATTATCCTTATTCATTCTCTCCCTATGGAGGCATACCCAGCAGATGCAACTGAATGCCACTGGTTCCAGGGACCCTAATACAGAGGCCCATGTTCGAGCCATGAAAACTATGTCTTCGTTCCTCATTCTCTTTTTATTGTACCATATAGGTTTTCTTCTCAACTACTGGACTTACTTACTGGGTACGAGCAAGATGTTTTCCTTATTGAATATATCAATCATGTTTCTCTATCCCTTTGGCCACTCACTGATCCTGATTCTGTGGAATAGCAAGCTAAGGAAGGCTGTCCTGAAGTTGTGGTGGAAGATAAGGTGCTACCAGCAAGGAAGTCATCCTCAAGTCCTGTGGACAATTTGGCATTTTCTGAGGGGACTGAAGTCACGATGGAGTCCTTAA

>Opossum_T2R28--Intact

ATGGAGAACATCTGTGTCACTGTGGCATCTGGAGAACTCTCCATTGGAGTTTTGGCTAACATCTTCATTGGTGTGGTGAACTACTTGAACTGGCTCAAGACCAGGAAAGTGTCCACACTGGACTTCATCCTTACAGGCTTGGCCATATCCAGGATCATTCTTCTGTGGACAATAGCATCTATGATTATTTTATTGGGTCACTATTTCAAGAATAACCTTGAAATTAGACTGAGATTTTGGAAGCTTCTATGGTACCTATGCAGTCTGTCAAGTGCCTGGTTTGATGCTTCCCTCAACATCTTCTTGTTTCTGAAAATCGCCAGTTTCTCCCACCCTGTCTTTCTTTGGCTGAAGTGGAGAGTTGATAAGTTGGTCTTCAGGATGCTGATGGGGTGCTTGATTATTTCTTTGCTCATTTTACTTCCACTGGTAGAAAAATCACTTCCAATTTTCTTTTATAAAAGAAATAAAGCAAACATAACTGAAATGATTCAAATAGATAGCATCAACCTTTTACTTTCTACAGTATTCTTTTATATGGGGAATCTCCTCCCTTTCACTCTATGTCTGATTTCTTGTTTCCTACTAGTCCTATCACTATGGAGACACACCCAAAAGATGCAGCTCAATTTCAGAGACTCCAGAGACCCAAGCACACAAGCACATATAAAAGCCATGAAATTCACAATCTCCTTCCTCTTCCTCTTTGTACTATACCATGTGGCGATCATCATAGCAGTTTTAAGTTTTATTCTATTTGACAGTAATCTGATTATAATGTTTGCAATGATAACCATGTCCATCTATCCTTTGGTCCACTCCATAATTCTGATCCTGGAGAATAGCAAGCTGAGGCAGGCCTTACTGAAGGCATTTTGGAAACTCAGATGCTACCTCAGAAACTCTGGCAGACTCTTGGGAACATGA

>Opossum_T2R27--Intact

ATGCCAAGTGTACTTGAGAGCATCTGTATGATTGTGGTCTTTGGACAGTTCCTAGTCGGGGTTTTTGCCAATGGCTTCATTGGATTGGTAAACTGTATTGAGTGGGTGAAGACCAGAAGAGTGACCCCAATTGATTTCATCCTTACTGGCTTGGCCATCGCCAGGATTGGTACATTGTGGGCACTAATGATAATGACCTTTTTTGCTTTTCTCTATTTTAAGAGAAATTTCATTAATAATTGGACTCACTCAGAAGTTATTTGGAACATCAGCAACCATTCAAGTGCCTGGTTTGGAACTTGCCTCAGTATCTTCTACTTTCTGAAGATTGCAAATTTCTCCCACCCTGCCTTCCTCTGGCTGAAGTGGAGAATTAACAAGGTAGTCCTCAGGATGCTGATGGGGTGCTTACTCATATCCTTGGTCATTATCCTTCCAGTGATGGAGAGAATCACTAAGATGCAACTGATTCCCAGTGGTCAGGAAAATGGAATAAATATTAGTTATAATGTCCAAAGATTGAAAACCCTACAATTTTCTGCCTCAATTCTCTTAAATACTGGTGGTTTGATCCCCTTTGCTCTATCTGTCGTCTCCTGTTTCCTGTTAGTACTGTCACTGTGGAGGCATACACGGAAGATGCACCTCAAAGTCATAGGCTCCAGGGATCCCAACACAGAGGCCCATGTGAGGGCCATGAAATGCATGATCTCTTTCCTCTTTCTTTTTGTTGTATATCATTTGGGCCTCGCCATTGGAGTCTTAAACGTTTTTATATTCCATTGTAAACTTCTTGCCATACTTTCAGCGATTATCATGAATGTCTATCCCATGACCCACTCCATCATCCTAATACTGGGCCATAGCAAGCTGAGGCAGGCCTTTCTGAGGGGACTGAGGAAACTGCCTCAACATCCCTGGGGAAGTCTAAGTGGCAGAGGCTAA

>Opossum_T2R21--Intact

ATGCCAAATGTACTGGAGAAAATGTGTGTGACTATGGCATCTGGAGAATTCCTAGCAGGAGTTTTGGTCAATGGTTTCATTGGAATGGTAAACTTCATAGACTGCATCAAGACCAGAAGAATATCCTACTTGAATCTTATCTTCATTGGCTTAGCCGTCTCCAGAATTGGTCTGTTAGGAATTTTGACATCACAAATCTTTTTCCTCTCATTCCATCCATTATGTTTTGAGATCAACTGGAGATATTTTAATATGCTTTGGATTTTGGCCAATACTTCTAGTTCTTGGTTCTCTGCCTTTCTCAGTATCTTCTACTTTCTGAAAATTGCCAACTTTACCCATCCTATCTTCCTCTGGCTGAAGTGGAGGGTAAATAGAGGAGTCTTCAGGATGCTCTTTGGCTGCTATTTTCTTTCTTTTTTCATTTTCCTTTTGTTATCATTGTTAAGTGATGCTTTAGAAGTTACTTTTGATCTAGAAAACAACAGAAACTTGTCACAGGAGATCCAAAAATCTAAAACTTCCTTTTTTTCTTCAATGATACTCTTCAGTATTGGTGGTCTTATCCCTTTTGCTATGTCCCTGATCTCCTGTTTCCTTCTAGTCCTGTCACTTTGGAGGCACACTTGTAAGATGCAACTCAGTATCACAGGCTCCAAAGACCCTAGCATGGAGGCACATATAAAGGCCATGAAATCCATGGTCTCTTTCTTCTTCCTCTTTGTTCTGTATTATGTATGCATGTTCTTACTAGTCACAAATAATCCAGTGACGAAGAGGACACAGTCTGTACTGCTTATTTTGATAACTATGATCAAGTATCCCTTGGTCCACTCCATTATCCTTATTTTGGGACACAACAAGCTAAAACCCACTGCCCTGAGGGTGCTTTGGAAGCTCAGGATCTGTTTCAAGTGTTCTGGAAAAGGATGA

>Platypus_T2R1--Intact

ATGGCAGCCCCCCTGACCATCTTCTTTTTCAGCCTTTATCTGCTGGTGCTGCTGGTGGGGATCCTGGGTGACGGATTCATCGCGGGGCTGTTGGGCAGCGTGTGGGTCCGACGCAGGAAGCTGCCTCCGTGCGACATGATCGTGGCCAGCCTAGGAACCTCCAGGTTTTTCCTATTGTTGTTGTCGATGCTGAATGGGATCTGCATCTTGGTCTCCCCCAGACCATATTACTCCCCATTTCTCTTCTACTGCATGTTTTTCTGGGACCTTTTGAACTTGTTCTCCTTCTGGTTTGCGGCGGGGCTCAGCGTCTTCTACTGCGTGAAGATCACCACCTTCACCCACCCATTCTTCCTCTGGCTGAAGCAGAAGATCTCGGGGATGGTGCCTTGGTTCCTCATAGGTTCCATATTGGTTTCCTGTGTCTCCACTTTCCCCTTCATTATCCACAACAGCGCTAACTCCACCTGGTGGAACAACTCTGGGAACAGAACTGAGGTTGACTGGAAGACCTTCCAGTCACTCAAAGTCCTGGTCATGATCTTCATCCCAATGAGTGTCCCAGTCCTCCTGCTCCTCATCTCCTCCATCCTGCTGGCCACCTCCCTCCGGAGACACCTGAGGGCCATGCAGCACCACGGCCCCGGCCTGCAGGACTGTAGCACCAAGGCTCACAGGCGTGCGCTCGCATCGCTGACCTCCTTACTCTCCTTCTATGTTTGGTACTTCATCTCTCTGGTCTTAACCTCGGCCTTCACCATTCCATTTGACGGTTCCTGGATATGGCTAGTCCAGCTGGTGACTTTCTTGGGGACAACATGCCACCCTCTCTTGCTGACGTGGAACAACCCCAAAATCCGGCGGGCCTTAGAGAGGAGGCTGCATCACATCTCAGCCTGCCAGACACCGTGA

>Platypus_T2R2--Intact

ATGGAGGACGCCCTGACCATCTTCTTTTTCAGCCTTTTCCTGCTGGAGGTGCTGGTAGGGATCCTGGGTGATGGATTACTCGCGGGGCTGTTTGGCAGGGAATGGGTCCGACGCAGGAAGCTGCCTCCGTGTGACATGATCGTGGCCAGTCTGGGAGCCTCCAGGTTTTTCTTGCTGTGGGTGTCGATGCTGAATGGAATTTGCCTCGAGGTCATCCCCAAATTTTATTATTCCCCCATTATCTTCTATAGCAGTTTTGTCTGGGCCCTTTTGAACTTGTTCTCCTTCTGGTTTGCGGCGGGACTCAGCATCTTTTATTGCTTGAAGATCGCCACCTTCACCAACCCACTCTTCCTCTGGATGAAGCAAAAGACCTCTGGGATGGTGCCCTGGCTCCTCTTAGGTTCCGTATTTGTTTCCTGTGTCTCGACTTTCCCCTGCATTATCTATTACAGCATTAAGCCCCCCTGGATGAACAACTCAGAGAACGGCACTGAGGCTGTGTTGAAGACCTTCCAGTCACTCAAAATCATGCCTGTGATCTTCATCCCACTGAGTGTCCCGTTCCTCCTGCTCCTCATCTCCTCCATCCTTCTGACCACCTCCCTCCGGAGACACCTGAGAGCCATGCAGCATCACGGCCCCAGCCTGCAAGACTGCAGCACCAAGGCTCATGCCCATGCACTTGCAATGCTGGCCTCCTTCCTCTCCTTCTATGTTTGGTACTTCATCATTCTGATTGTCACCTCGACTACGGCCATTCCATCTGGGAGTCCCTGGTTCTGGCTATTCCAGGTGATAACTTTCTTGGGGACAACGTGCCACTCCCTCTTGCTGACGTGGAGCAACCCCAAAATCCGGGGGGCTTTGGAGAGGGGGCTGCGTCACATCCCAGCCTGCCAGACACCGTATGGGACAGCATAA

>Platypus_T2R3--Intact

ATGGCGGCCCCACTGACCGTGTTCTTTTTCAGCCTTTACCTGCTGGTGCTGCTGATGGGGATCCTGGGTGACGGATTCATCGTGGGACTGTTGAGCAGAGAATGGGTTCGAAGCAGGGAGCTGCCTCCGTGCGACAAGATCGTGGCCAGTCTGGGAGCCTCCAGGTTTTTCATGCATTGGATGTCAACCCTGAATGGGATCTGCATCCTGGTCTCCCCCAGATCATATTGTTCCACTAATGTCTTCTACAGTGGGATCCTCTGGATCCTTCTGAACTTGTTCTCCTTCTGGTTTGCTGCCGGGCTCAGCGTCTTCTACTGCTTGAAGATCGCCACTTTCACCCACCCACTCTTCCTCTGGCTGAAGCAAAAGATTTCGAGGATGGTGCCGTGGCTCCTCATAGGTTCTATACTGGTTTCCTGTGTCTCCACTCTACCCTTCATTATCCATTACAGCACTAATGACACCTGGAAGAACAAGTCAAGGAACAGCACCGAGACTGTGGCGAAGACCTTCCAGTCTCTAAAAATCTTGCCATTGATCTTCATCCCATTGTGTATCCCATTCCTCCTGCTGCTCATCTCCTCCGTCCTGCTGACCACCTCCCTCTGGAGACACCTGAAGGCCATGCAGCATCACGGCCCCGGCCTGCAGGACTGCAGCACCAAGGCTCACACCTGTGCGCTCACAACGCTGGCCTCCTTTCTCTCCTTCTACGTTTGGTACTTCATCTCTCTGATCGTCACCTCGACCTTGACCATTCCATTTGACAGTCCTTGGTTATGGCTAGTCCAGCTCTTATCTTTCTTGGGGACAACGTGCCACCCCGTCTTGCTGATATGGAGCAACCCCAAAATCCGGGGGGGCCTAGAGAGGGGACTCCATTACATACCAGCCTGCCAGACACCGTGTGGGACAGCATAA

>Platypus_T2R4--Intact

ATGGACCCATCGTGCATCACGTACCTGGTGGTCTTAGTAGCCGAGTTCCTGGTTGGGATGTCGGTCAACGGGCTCATCGTGGGAGTGAGCTGCACCCACAGGTACCGAAGAAGGAGAATGACACCGTGTGACCTCCTTCTCACCAGCTTGGGGTTCTCCAGGATGTGCCTGGAACTGGTTTTACTTCACGCCTCCATCGAGACGTTCCTGTTCCTGAAGAGTTACCCGAACAGCACGCTGCAGTATTCCTTCTGCATGTTCATCAACCAGGTGGATCTCTGGTTCTCCACCTGGCTCAGCGTCTTCTACTTCGTGAAGATCACCACCTTCACCCACCCTCTCTTCCTCTGGCTGAAGCGGAGGATCTCCGGGCTGGTGCCCTGGTTGCTCCTGTGTTCTCTGCTCTGTTCCGTCGTCACCAACTGCCCGTTATTAAGGAAGCGGACCGGAAACGGGCTCGCCGGCAATCTCTCCGGCGGCCACCCGGAACCGGGAAGCGCTCAGGTGGTCGTTAATCCGCTTTCCGGCCCAATCCTGATTCTGCCCTTCCCGATCTTCCTCACCGCGTCTTTCCTGCTGATGGTTTCTCTCTGCAGACACGTCAGGCGCATGAGACGCTCGGGCACCGGCGCCCGGGGTCCCAGCGTGGCGGCCCACTCCGGGGCCATCAAGACCGTGTTCTTCTTCCTCCTCTTCTGCAGCTCCTATTTCGTGGCCATGACTCTGTTGTGTAGCCGAACTTTCCCCATAGAAAGCCTCTTCACCACTCTCTGTGAAATTATTCTTTTAGCGTGTCCTACCGGGCACTCCGTTGTGCTGGTTCTGGGAAATCCCAAGATGAAGTGGGCGGGGAAACGGCTTCTCACCTGCCCCGGGGCCGGCCGAAGTCGAGGGGCTTCGTCTCTTCAGGGCCTCAGACTTAAGCCCTGA

>Pufferfish_T2R1--Intact

ATGCTGAGCTCCAGTAAGGCGGTGCTGTTTGTGCTGACGGGCCCGTTGGCCGTCGCCACCGTCTTCTTCAACGGCTACATCCTCCTGAGCGCGCTGTCCAGGCTGCGGCAGAAGCAGCGGCAGGCTCCCAGCGAGACCATCATCGTGGCGCTGGCGCTCGCCGCCATCGCCTACCAGCTGGTCTGCTACCTCTGGATGAGCATGGACCAGCTGGACACCGGCTGCCGGATGGACGCCGCGCCCTACACCGTCATGCTGCTCCTGGTCAAGAGCATGAAGTTCATCATCATGTGGGACATCAGCTTTCTGACCTTCTACTACAGCAGCAAGCTGGTGACCGCCGCCCAGCGCCACCACGCCCACATCCAGGCTGTCCTGGCGCACGCCACCCCCACCGTGGTCCTCATTCCCGTCTGCGGTTTGGTGACCTGCATGCCCATGTTGGCCGTGTTTCGCTATGACAACCAGACGCTGGCCAACGAGGACTGCGGCGTGCTGGTGCCGTACGGCACCGCCGGGAGTGTCTACGAGGTGGCGTACCTGCTGCTGGCCGACGTCCTGCCCGGTCTGGTCATGGTCAGGTGCTGCGCGTCCATCTCCGTCCACCTGGCCGTGCACCTGCGCCACATGAAGGCCAGCACCAACGGCACCCACGGCCCCCGGCTGGGCTCCCAGAGGAGGGTGATCCAGATGGCGCTCTCCCTGGTGGCCATCTTCATCGTCTTCCTCGTGGTCGACCTGTACGTGCAGTACCAGATATCTGTGCACCACGAGAACATCCTGGTGCTGACGTTCTTCTTCACCTCCATCTACACCACCGTCACCGCCTTGGTGCTCATTTACGGGAAAAAGTCCTTCTGGCAGGTTCTGACGCGCGAGTTCAACGCCCTCCTGCACGGCTGCTGCCTGGCTTGTCAGAAAGTGCCCGAACACAAGGCTAAAGCTAGCATTTCCGCTAAAGTGAAGAGCTGA

>Pufferfish_T2R2--Intact

ATGAATGTGGATACTGCTGAGCTCATCAGGGTTAACTTGCCCATCGTCATCCTGAATGCGCTCTCCAACCTGTTCTTCGTCTTCTGCCTGATGCGTCCTTCTCAGGGAGAAATGCTCAAACAACCCCTGAAGCTTCTCCTCTGGACCATGGTCTGCTCCAACCTGTCTTTCCTGGTGATGCTCCTGGTTCGGTTCCACTTTGTCAATGACAGCATCGTGACCTCCCTCATCGGTTTTGCCACGTTCATTTTCAGTTTGACCATGAGCCTGAACGCCAGTGTCTGGCTGAACTTCTTCTACTACATGCAGATTGTACCTTCCAAGAGCGCCCTCTTCGTTTGGATTAAGAGGAACCTTAAGCCCATCATCTACTACATTTGTGTGGCTGAGAAAGTACAAATCTGGCTATTAATTGGCAGCATAATTGTATACAATATCACTGAAAAAAATTATAAACTGGAATATTTAAACATTGTAAATACTAGTTATGTAGCATCCTTCGCAACATCCTCCCATCTGCGAACTTTTACGCTTACCATGATTTTCTCAGATGGGGTCTACTATATTATCACCCTGTGCGTCATGATGGCGTCCAGCTTGTGCACTGTCGCCTACCTGAGCAGACACATGCATCAGATGGCCTCTCAAGGACTGTCCGGCTCCCGCTTCCGAAGCCAGGTGAGAGTCACTGCCAGTGGGGTCCTGCAGGGGGCGCTCTACGTGGCAATATTTGTATTGACTCTGCCCCAGTTTCAGTTTAACATCTTAGACGGAGCTACTGCGTACATGACTTTATCCGATATCACCATGATCAACGTCTACATGATGGGCACCAGTGTGAATCTGGGCATGGGTCAGATCGTGTTCCGGCAGAGAGCTGTGGACCTGTTGCACAGAGCGGTGAGGAGCTGCACAACCATCAAAGCACCACAGCCTCAACCAGGAGGATCATGA

>Pufferfish_T2R3--Intact

ATGGATATTTTTTTGATCATCAGGATCAACGTGCCCATGGTCATCCTGAATCTGCTCTCCAACCTGTTCTTCGTCTTCTGCCTGATGCGTCCTTCTCAGGGAGAAACGCTCAAACAACCCCTGAAGCTTCTCCTCTGGACCATAATCTGCTCCACCCTGTCTTTCCTGGTGACGCTCCTGGTTCTGTTTTTCATGGACCATGATATCCCCGAGGCCAATCTGGCCATTTGTGGGATGTTTCTTCTCAGTTTGTCCATCAGCCTGAATGCCAGTGTCTGGCTGAACTTCTTCTACTACATGCAGATCGTACCTTCCAAGAGCGCCATCTTCGTTTGGATTAAGAGGAACCTTAAACTCATCATCTACTACATTTGGGTGGCTGAGAAAGTAATAAATGGGCTAATTGTTTGGAGCATTTTGACATTCAGCATCTCTATCACCAATTATTTGAACGATTTTAAATTCCCTAATAGCACAAATGCTAGCTATGTAATATTCATGCCCTCCCACCTGGTAAATATGAGGTCCTCAATTATTGCGACCCAGATCTACATTCTTATCTGCCTGTGCATCATGTTGGCGTCCAGCTGGTGCACTGTCGCCTACCTGAGCAGACACATGCATCAGATGGCCTCTCAAGGACTGTCCGGCTCCCACTTCCGAAGACAGGTGAGAGTCACTGCCACCGGTGTCATGCAGGGAGTGCTCTACATGATGGTTACCATGTGCATTGTGTCCGTGAATCTGTTATCTGACGTTTTAAGCACTCAAGCTTCATACTTCAGTTTATCCAACATCACCATGATCAACGTCTACATGATGGGCACCAGTGTGAATCTGGGCATGGGTCAGACCGTGTTCAGGCAGAGAGCTGTGGACCTGTGGCACAGAGCGGTGAGGAGCTGCACAACCATCAAAGCACCACAGCCTCAACCAGGAGGATCATGA

>Pufferfish_T2R4--Intact

ATGAGTGTGGATACTGCTGAGCTCATCAGGGTTAACTTGCCCATCGTCATCCTGAATGCGCTCTCCAACCTGTACTTCATCTTCTGCCTGATGCGTCCTTCTCAGGGAGAAATGCTCAAACAACCCCTGAAGCTTCTCCTCTGGACCATGGTCTGCTCCACCCTGTCTTTCCTGGTGACACTCCTGGTTCTGTTCCACTTGTTCAGTGACAGCATCGTGACCTCCTTCACCAGTTATGGCATGTTTGTCATCACTTTGTCCATCAGCATGAACACCAGTGTCTGGCTGAACTTCTTCTACTACATGCAGATCGTACCTTCCAAGAGCGCCGTCTTTGTTTGGATTAAGAGGAACCTTAAACCCATCATCTACTACATTTGGGTGGCTGAGAAAATATATATCGGGCTATTTGTTAGCAGCATAGTTTTATTTAATATTACTGTAATAAAATATGAACTGGAATATTTAAACATCGTAAATACTAGTTCTGTAGCACCCTTTGAAACAGCCTCCCACCTGCGAACACTGACAACTACCATAGTTCTATCAGGTGAGATCTACTTTATTTTCAACCTGTGCATCATGTCGGCATCCAGCTGGTGCACTGTCGTCTACCTGAGCAGACACATGCGTCAGATGGCCTCTCAAGGACTGTCCGGCTCCCGCTTCCGAAGCCAGGTGAGAGTCACTGCCACCGGTATCCTGCAGGGAGTGCTCTACCTGGTTATATCTGTGTGGACTCTGTCCCAGTTTCAGTTTAGTGACATAATAGCCGACTATGCTGCGTGCGTGAGTTTATCCAATATCACCATGATCAACATCTACATGATGGGCACCAGTGTGAATCTGGGCGTGGGTCAGACCGTGTTCAGGCAGAAAGCTGTGGACCTGTGGCACAGAGCGGTGAGGAGCTGCACAACCATCAAAGCACAACCAAAATGA

>Pufferfish_T2R5--Intact

ATGAATGTGGATACTGCTGAGCTCATCAGGGTTAACTTGCCCATCGTCATCCTGAATGCGCTCTCCAACCTGTTCTTCGTCTTCTGCCTGATGCGTCCTTCTCAGGGAGAAACGCTCAAACAACCCCTGAAGCTTCTCCTCTGGACCATGGTCTGCTCCAACCTGTCTTTCCTGGTGATGCTCCTGGTTCGGTTCCACTTTGTCAATGACAGCATCGTGACCTCCCTCATCGGTTTTGCCACGTTCATTTTCAGTTTGTCCATCAGCCTGAATGCCAGTGTCTGGCTGAACTTCTTCTACTACATGCAGATCGTACCTTCCAAGAGCGCCGTCTTTGTTTGGATTAAGAGGAACCTTAAACCCATCCTCTACTGCATTTGGGTGGCTGAGAAAGTACAAATCTGGCTATTAATTGGCAGCATAATTGTATACAATATCATTGAAAAAAAATATGAACTGGAATATTTAAACATCGTAAATACTAGTTATGTAGCATCCTTCGCAACATCCTCCCATCTGCGAACGTTTACGCTTACCATGATTTTCTCAGATGGGATCTACTATATTATCACCCTGTGCGTCATGTCGGCATCCAGCTTGTGCACTGTCGTCTACCTGAGCAGACACATGCATCAGATGGCCTCTCAAGGACTGTCCGGCTCCCGCTTCCGAAGCCAGGTGAGAGTCACTGCCAGTGGGGTCCTGCAGGGGGCGCTCTTCGTGGCAATATTTGTATTGACTCTGCCCCAGTTTGAGTTTAACGTCTTAGACGGAGCTACTGCGTACATGACTTTATCCGATATCACCATGATCAGCGTCTACATGATGGGCACCAGTGTGAATCTGGGCATGGGTCAGATCGTGTTCCGGCAGAGAGCTGTGGACCTGTGGCACAGAGCGGTGAGGAGCTGCACAACCATCAAAGCACCACAGCCTCAACCAGGAGGATCATGA

>Pufferfish_T2R6--Intact

ATGGAACGCTGCTATATTGACCTGGATAAGAGGACCTTCCAACTCATCATGTATCCTCTCTGTGGGATCAACCTGTTTTCAAACTTTTTCTTTGGCTACTGCCTGGTCTCTAACAGACGGAGGCTCAGGCAGCCTTTGAAGATGCTGCTGACCTTCCTCGTCTTGTGCACGATCGCCTTTGTCGTTCATTTGATCATCTCGCATCCGCTGTTTGCTGAGATGACCAACAACGATGTCCGCCACCATTTATCGTGGATGATCACACTGCTCATCATGCACAGCAGCATGACGGGTGCAGTTTCGATGAGCTTCTACTACTACGTGCAGATTGTCCCCTCCCAGAGAGCTCTTCTGATCTGGATAAAGAGGAACATCAAGTCCTTCATCTATGTGATTTTTCTCTTCGGAGAGATATTTCTTGCCTTCAGCTCTTTTGTGAATGTTTTGTCTGTGGTTCTGGATTCCTGGGTTGTCAGCGCTAACAACTGTACGAACAATGAGCTCCCTGTGGTTGGACCTACGGCCACCGACACCGTCGTCTCCATCTTTGTCAGAATACACATCCTGTGCTGCATGGCCATAATGGGAGTGTGTAACTTCTCCATGACCCACTACCTGCTCAGGCACATCAAAAGCAGAACTCGGCAGGGTTTCGCTGCCTCAGAAACTCAAATGCGAGTGGCTATCTCTGATCTTATTCAGGCTGTGTTCTTCTTGATCTGCGGTTTGCTGTACTCTGTGTGCACGTTCATCTTTGAATATTCTAAGCAATTTAGCTTCGGTCCCTTGCTGTATCTCACAGGTGTGTTGCTGTATATGACAGGAACCACGGCCAGTCTGGCCACTGGCCAGGCTATATTCAGGCAAGGGGCAGTAGACCTATGGAAGGTGCTCACGGCAAGTTTTTGA

>Rat_T2R34--Intact

ATGTCTTTCTTCTTCATCTTCATGGTCATCTTTTGTATACAGTCTCTAGTTGCTTTGCTGCAAAATGGCTTCTTGGCCACTGTGCTGGGCAGGGAATGGGTACGAAGCCAGGGACTCCCTGCAGGTGACATGATTGTGGCTTGCCTAGCTGCCTCCAGGTTCTGCCTGCATGGAGTAGCCATCGTAAACAACTTCCTGACCTTTGTTAAGCTATGGAGCCAAAAGATCTATTTTTCTGTCCTCTGGGACTTCGTCAACACTGTCAATTTCTGGTGTACCACCTGGCTTGCCATCTTCTACTGCGTAAAGATCTCTTCGTTCTCCCACCCCATCTTCTTCTGGATAAAATGGAGAATTTCTCGGTCTGTGCCCAGGTTACTGCTGGGATCCCTGGTCATTGGTGGACTGTCGGCCGTCTCCTCCGCCACTGGAAACACAATCGCCTTTCAGATGACCGCCTGTGAGAACTACACGCTTGCTTATAGAACGAGAGCCTTCTACGCGTATTATTTTCGCTGTCATGCAATGCTGATGTGGATCATCCCGTTCTTCCTCTTCCTGCTGTCCGTTATCTTGCTCATGTTCTCACTCTACCGGCATCTGGAACACATGAGGTACCGCAGACCCTGGTCTCACGATTACAGCACCCAGGCCCACACTATGGCTCTGAAGTCGCTGGCCTTCTTCCTCGTCTTCTATACATCGTATGTCCTGTTCCTTGTGATATCTGTTACACGAGTCGTGAATGTCCACAGTTCCTGGCACTGGGCCTGGGAAGTGATAACCTACATGGGCATCCTACTGCATTCCACCATTCTGACACTAAGCAACCCCAAGATGAGAAAGGCCCTCAAGATAAAGTTCCCAGACCTTTGTGTTGCCAGATCACAAGACAAGAGGCGTGGTTAA

>Rat_T2R143--Intact

ATGCCCTCCACACCCACATTGATCTTCATTGTCATCTTTTTTCTGGTATCAGTGGCCTCTATGTTGCAGAATGGCTTCATGATCATTGTGCTGGGCAGAGAGTGGATGAGGAACCGGGCACTGCCGGCAGTTGACATGATTGTGGCTTCTCTTGCTTCCTCCCGGTTCTGCCTACATGGGATAGCCATCCTCAACAATTTCTTGGCCTCCTTTGATTTTTGTTACCAAGCAAACTTTGTTGGCATCCTCTGGGACTTCATTAATACTCTCATTTTGTGGCTTACTGCCTGGCTTGCCATCTTCTACTGTGTGAAGATCTCCTCTTTCTCCCACCCTGTCCTCTTTTGGCTCAAGTGGAGGATTTCCCAGTTAGTTCCCAGGCTGCTGCTGGTATCTCTCATCATGGGTGGCCTGTCAGCCATCATATCAGCTACCGGGAACATCATTGCCAATCAGATGATCATCTCCCAAGGTTTCCATGGAAACTGCACTTTTGGTCACATGTCACTGGACTTCTATCGGTATTATTACCTGTCTCACGCAGTGCTCATGTGGTTCACTCCTTTCTTCCTGTTTCTAGTGTCCATTATCTTCCTCATGTTCTCACTGTACCGGCATGTGGAGAAGATGAGGGGCCATAGGCCTGGGCCTTGGGATCCCCGTACACAGGCACACACCATGGCTCTGAAATCCCTTACTGTCTTCATCACCTTCTATATATTATTTTTTCTGGCCCTGATAATTTCTAGTACAAAAAGTAAAACTATGCACAGTTACTGGTATTGGGTCCGAGAAATTATCATCTACACTGGCATCTTTTTGAACTCCATCATCTTGGTGCTTAGCAACCCCAAGCTGAGAAAGGCCCTGAAGATGAGATTTTAG

>Rat_T2R22--Intact

ATGACTTTCTTTTTCCCAGCTATTTATCACATGGTCATCATGACAGCAGAGTTCCTCATAGGGACTACAGTGAATGGATTCCTTATCATTGTGAACTGCTATGACTTGTTCAAGAGCCGAGCATTCCCGATCCTGCCTACCCTCTTGATGTGCACAGGGCTGTCCAGACTCGGGCTGCAGATAATGCTGATGACACAAAGCTTCTTCTCTGTGTTCTTTCCATACTCTTATGAGGAAAATATTTATAGTTCCAAGATAATGTTCGTTTGGATGTTCTTCAGCTCAATTGGCCTCTGGTTTGCCACATGTCTTTCTGTCTTTTACTGCCTCAAGATTTCAGGCTTCACTCAGCCCTGGTTTCTTTGGCTGAAATTCAGAATTTCAAAGCTCATATTTTGGCTGCTTCTGGGCAGCTTGCTGGCCTCTTTGGGGACCGCAACTGTGTGTATAGAGGTAGGTTTCCCTTTAATTGAGGATGGGTATATCCTGAGGAACACAAGACTAAATAATAGTAATGTCAAGCTAATGAGAAATAACAACTTACTCCTCATCAACCTGACCTTACTGCTTCCCCTAACTGTGTTTGTGATGTGCACCTCTATGTTATTCATTTCTCTTTACAAGCACATGTACCGGATGCGAAGTGAATCTCAGAGGATGTCAAATGCCAGAACCGAAGCCCATATAAATGCATTAAAAACAGTGACATCATTCTTCTGTTTCTTTGTTTCTTACTTCGCTGCCTTCATGGCAAATATGACATTTAGAATTCCATACAGAAGTCATCAGTTCTTTGTGGTGAAGGAAATCATGGCAGCATATCCTGCCGGCCACTCCGTCATAATCATCTTGAGTAACTCTAAGTTCAAAGACTTATTCACGAGAATGATATGTCTGCAGAAGGAAGGGTGA

>Rat_T2R40--Intact

ATGAGTTTCTTGGTAAGCATTGCAGGCATTGCGATGCTGGCACAAATTGTTCTTGGCACCTTTGCCAATGTCTTCATTGTTCTGGTGACCTGCACTGACTGCATCAGGAGAAGAAAATTGTTCCTGGCTGATGGAATTCTCACTTCCCTGGCCTTCTGCAGGATTGGCATGCTCTGGGTAATATTAATAAGTTGGTGCTCAATTGTGTTTCACCAAGCTTTGTCTTTACAAGTAAGATTTAGCATTTGCGTTGGCTGGGCAGTAACCAACCATTTTAATATGTGGCTTGCCACTATACTTAGCATACTTTATTTGTTGAAGATAGGTAATTTCTCTAATCTTATTTTTCTTGGCCTAAAGAGAAAAATCAAGAGTGTCTTTATAGTTGTACTTTTGGCGAGCTTGGTGCTTTTATTTCCTAATCTTATAACGGTAACCGTATGTGAGACAGTACAAGCGAATGGATACCGAGGCAACTTGACTGGGAAGACCAAACGGACTTATTTCATGAACCTTACAGCTATGATATCTTTTACTCTAGACAACATCATTTCCTTCACCATATCCATGGTCTGTTTTCTTCTGTTAATCTATTCCCTGTGTAAACACCTTAGGACAATGAGGCTTTATGGAAAAGGACCCCACAACCCGAGTGCGTCAGCCCACATTAAGGCTCTGCAAGCTGTGATCTCCTTTCTGTTGTTATTTTCCATGTTTATTCTGTCTCTAATCATATCAGGTTACAATTATATGAAGCCTCTAAATGAACCAGTCCACCTGATTTGCCAGCTTATTGGGACTTTGTATCCTTCAAGCCATTCTTACGTTTTGCTATGGGGAAATAGGAGGATCAAACTGGCCTTTGTGTTGGCTATGGTACAGGTGAGGGCAAGGCTCTGGCTAAAAGAAGAGAAACCTTGA

>Rat_T2R131--Intact

ATGTGTGGATTCCCTCTTTCTATTCAACTGCTTACTGGATTGGTTCAAATGTACGTGATATTGATAATAGCAGTGTTTACACCTGGAATGCTGGGGAATGTGTTCATTGGACTGGTAAACTACTCTGACTGGGTAAAAAACAAGAAAATCACCTTCATCAACTTCATCCTGATCTGTTTGGCAGCGTCCAGAATCAGCTCTGTGTTGGTGGTATTTATTGATGCAATCATCCTAGAACTAACTCCTCATGTCTATCATTCTTACAGTCGAGTGAAATGCTCTGATATATTCTGGGTTATAACTGACCAGCTGTCAACGTGGCTTGCCACCTGCCTCAGCATTTTCTACTTACTCAAAATAGCCCACTTCTCCCATCCCCTTTTCCTTTGGTTGAAGTGGAGATTGAGAGGAGTGCTTGTTGGTTTTCTTCTATTTTCTTTGTTCTCATTGATTGTTTATTTTCTACTCCTGGAATTACTGTCTATTTGGGGAGATATTTATGTGATCCCTAAAAGCAATCTGACTTTATATTCAGAAACAATTAAGACCCTTGCTTTTCAAAAGATAATTGTTTTTGATATGCTATATTTAGTCCCATTTCTTGTGTCCCTAGCCTCATTGCTCCTTTTATTTTTATCCTTGGTGAAGCACTCCCAAAACCTTGACAGGATTTCTACCACCTCTGAAGATTCCAGAGCCAAGATCCACAAGAAGGCCATGAAAATGCTATTATCTTTCCTCGTTCTCTTTATAATTCACATTTTTTGCATGCAGTTGTCACGGTGGTTATTCTTTCTGTTTCCAAACAACAGGTCAACTAATTTTCTTTTGTTAACATTAAACATCTTCCCATTATCTCATACATTCATTATCATCCTGGGAAACAGCAAGCTTCGACAAAGAGCAATGAGGGTCCTGCAACATCTTAAAAGCCAACTTCAAGAGTTGATCCTCTCCCTTCATAGATTGTCCAGAGTCTTCACTATGGAAATAGCTTAA

>Rat_T2R119--Intact

ATGATGGAAGGGCATATACTCTTCTTCTTTTTGGTTGTGATGGTGCAGTTTGTCACTGGGGTCTTGGCAAATGGCCTCATTGTGGTTGTCCATGCTATTGACTTGATCATGTGGAAGAAAATGGCCCCGTTGGATCTGCTTCTATTTTGCCTGGCGACTTCTCGGATCATTCTGCAGTTATGTATATTGTTTGCACAATTGTGTCTATTCTCTTTGGTGAGACACACTTTATTTGAGGACAATATTACCTTTGTCTTCATCATAAATGAACTGAGTCTTTGGTTTGCTACATGGCTCGGTGTTTTCTACTGTGCCAAGATTGCTACCATTCCTCACCCACTCTTTCTGTGGCTGAAGATGAGGATATCCAGGTTGGTACCATGGCTGATCCTGGGATCTGTGCTCTATGTAATTATTACTACTTTCATCCATAGCAGAGAGACTTCAGCAATCCTTAAACCAATTTTTATAAGCCTTTTTCCTAAAAATGCAACTCAAGTCGGAACAGGGCATGCCACACTACTCTCAGTCCTGGTCCTTGGGCTCACACTGCCATTGTTCATCTTTACTGTTGCTGTTCTGCTCTTGATATACTCCCTGTGGAATTATAGCAGGCAGATGAGGACTATGGTAGGCACCAGGGAGTATAGCGGACATGCTCACATCAGTGCAATGCTGTCCATTCTATCATTCCTCATCCTCTATCTCTCCCACTACATGGTGGCTGTTCTGATCTCTACTCAAGGCCTCTACCTTGGAAGCAGAACCTTTGTATTCTGCTTACTGGTTATTGGTATGTACCCCTCAATACACTCGATTGTCTTAATTTTAGGAAATCCTAAGCTGAAACGAAATGCAAAAATGTTCATTGTCCATTGTAAGTGTTGTCATTGTACAAGAGCTTGGGTCACCTCAAGGAGCCCAAGACTCAGTGACTTGCCAGTGCCTCCTACTCATCCCTCAGCCAACAAGACATCCTGCTCAGAAGCCTGTATAATGCCATCCTAA

>Rat_T2R107--Intact

ATGCTGAGTGCAGCAGAAGGCATCCTCCTTTGTGTTGTCACTAGTGAGGCAGTGCTGGGGGTTTTAGGAGACACATTCATTGCACTTGCAAACTGCATGGAGTATGCCAAGAACAAGAAGCTCTCTAAGATTGGTTTCATTCTCATTGGCTTGGCGATTTCCAGAATTGGTGTCGTATGGATAATAATTTTACAGGGGTATATGCAAGTATTTTTTCCACACATACTTACCTTTGGAAACATAACTGAATATATTACTTACATATGGGTGTTTCTCAATCACTTAAGTGTCTGGTTTGCTACCAACCTCAATATCCTCTACTTTCTAAAGATAGCAAATTTTTCCAACTCTGTATTTCTCTGGCTGAAAAGTAGAGTCCGTGTGGTTTTTATCTTTCTGTCAGGATGCTTACTTACCTCGTGGTTACTATGTTTTCCACAATTTTCAAAGATGCTTAACAACAGTAAAATGTACTGGGGAAACACGTCTTGGCTCCAGCAGCAGAAAAATGTCTTCCTTATTAACCAAAGTTTAACCAATCTGGGAATCTTCTTTTTCATTATTGTATCCCTGATTACCTGCTTCCTGTTGATTGTTTTCCTCTGGAGACACATCAGGCAAATGCACTCAGATGGTTCAGGACTCAGAGACCTCAACACAGAAGCTCATGTGAAAGCCATGAGAGTTCTAATATCTTTTGCGGTACTCTTTATCCTGCATTTCGTAGGTCTTTCCATACAAGTGCTATGCTTTTTTCTGCCACAAAACAACCTACTCTTTATAACTGGTTTGACAGCCACATGCCTCTATCCCTGTGGTCACTCAATCATCTTAATTCTAGGAAACAAGCAGCTGAAGCAAGCCTCCTTGAAGGCACTGCAGCACTTAACGTGCTGTGAGACAAAAAGAAATCTCTCAGTCACATAA

>Rat_T2R109--Intact

ATGGAGCATTTTTTGAAGAGTATATTTGATATCTCCAAGAATGTACTTCCAATTATTTTATTCATTGAATTAATAATTGGAATTATAGGAAATGGTTTCATGGCCCTGGTGCATTGCATGGACTGGGTTAAGAGAAAAAAAATGTCATTAGTTAACCAAATCCTCACCACCTTAGCAACCTCCAGAATTTGTCTGCTCTGGTTCATGCTATTAGGTTTACTAATTACCTTACTGGATCCAGATTTAGCTAGTGCTAGAATGATGATCCAGGTCGCCAGTAATCTGTGGATTATAGCTAACCATATGAGCATTTGGCTTGCTACATGCCTCACTGTTTTTTATTTTCTCAAGATAGCCAATTTTTCTAGCTCTCTTTTTCTTTATCTAAAGTGGAGAGTTGAAAAAGTCATTTCAGTTATATTTCTGGTGTCGCTGGTCTTACTGTTTTTAAATATGTTACTAATGAACTTGGAAAATGACATGTGTATAGCTGAATATCATCAGATAAATATATCGTACAGCTTCATTTACCATTACCGTGCAGACTGCGAAAGGCGTGTTTTAAGACTTCACATTATCATCTTGTCTGTCCCCTTTGTTTTGTCCCTGCCAACTTTTCTCCTGCTCATCTTCTCCCTGTGGACACATCACAAGAAGATGCAGCAGCATGTTCAAGGACGCCGAGATGCCAGCACCACGGCCCACTTCAAAGCCTTGCAGACCGTGATCGCCTTTCTCCTATTATACTGTATTTTTATTCTGTCTATGTTACTACAATTTTGGAAATATGAATTAATGAAGAAACCCCTTTTCATTTTATTTTGTCATATTGTATATGGAGCTTTCCCTTCATTCCATTCATATGTCTTGATTCTGGGCGACATGAAGCTGAGACAGGCCTCTCTCTCTGTGCTGTTGTGGCTGAAATGCAGGCCAAATTACATAGAAACGTTAGATCTCTAA

>Rat_T2R102--Intact

ATGGAACCTGTCATTTACAGCTTTGCCACTCTACTAATACATGTGGAGTTCATTTTTGGGAATCTGAGCAATGGATTTATAGTGTTGTCAAACTTCTGGGACTGGGTCATTAAACGAAAACTTTCCACAATTGATAAAATTCTTCTTACATTGGCAATTTCAAGAATCACTCTCATCTGGGAAATATATACTTGGTTTACAAGTGTATATGGTCCATCTTCATTTGCAATTGGAATGAAATTACAAATTCTTTATTTTACCTGGATCCTTTCTAGTCACTTCAGCCTCTGGTTTGCCACAGCTCTCAGCATCTTTTACTTACTCAGAATAGCTAACTGCTCCTGGAAGATCTTCCTGTATTTGAAATGGAGACTTAAACAAGTGATTGTGGGGATGTTGTTGGCAAGCTTGGTGTTCTTGCCTGGAATCCTGACGCAAAGGACTCTTGAAGAGAGGCCCTATCGATATGGAGGAAACACAAGTGAGGATTCCATGGAAACTGACTTTGCAAGGTTTACAGAGCTGATTCTTTTCAACTTGACTATATTCTCTGTAATACCATTTTCATTGGCCTCGATTTCTTTTCTCCTGCTAATCTTCTCCTTGTGGAAACATCTCCGGAAGATGCAGCTCAGTTCCAGAGGACATGGAGACCCTAGCACCAAGGCCCACACAAATGCTTTGAGAATTATGGTCTCCTTCCTCTTGCTCTATTCTATATATTTCCTGTCTCTTCTTTTATCATGGATTGCTCAGAAGCATCACAGTAAACTGGTTGACATTATTGGTATTATTACTGGACTCATGTATCCTTCTGCCCACTCATTTATTCTGATTCTAGGAAATTCTAAATTAATGCAGACTTCTCTTTGGATACTGAGTCATTTGAGATGTAGACTGAAAGGAGAGAATATTTTAAATCCATCTGGCAACCAAGTAACTAGCTGTTATATATTCTGTATTGCGAATAAATCTGTGAGTTAG

>Rat_T2R118--Intact

ATGGTGCCAACCCAAGTCACCATCTTCTCTATCATCATGTATGTGCTTGAGTCCTTAGTCATAATTGTGCAAAGTTGCACAACGGTTGCAGTGCTGTTCAGAGAGTGGATGCACTTTCAAAGACTGTCGCCGGTGGAAATAATTCTCATCAGCCTGGGCATTTCACATTTCTGTCTACAGTGGACATCGATGCTGTACAACTTTGGTACCTACTCTAGGCCTGTCCTTTTATTTTGGAAGGTATCGGTCGTCTGGGAGTTCATGAACGTTTTGACATTCTGGCTAACCAGTTTGCTTGCTGTCCTCTACTGTGTCAAGGTCTCTTCCTTCTCTCACCCCGTCTTCCTCTGGCTGAGGTTGAAAATTTTGAAACTGGTTCTCTGGTTGCTATTGGGCGCTCTGATAGCTTCTTGTTTGTCAATCATCCCTTCTGTTGTTAAATATCATATCCAGATGGAATTACTCACCCTAGATCATTTACCCAAAAACAGTTCTTTGATTCTAAGACTGCAAATGTTCGAGTGGTATTTTTCTAATCCTTTCAAAATGATTGGGTTTGGCGTTCCTTTCCTCGTGTTCCTGATTTCTATCATCTTACTCACAGTCTCGCTGGTCCAGCATTGGGGGCAGATGAAACACTACAGCAGCAGCAGCTCCAGCCTGAGAGCTCAGTGCACTGTTCTGAAGTCTCTTGCCACCTTCTTCATCTTCTTCACATCCTATTTTCTGACTATAGTCGTCTCCTTTATTGGCACCGTGTTTGATAAGAAGTCATGGTTCTGGGTCTGCGAAGCTGTCATCTATGGTTTAGTCTGTATTCACTTCACTTCCCTGATGATGAGCAACCCTACACTGAAAAAAGCACTCAGGTTGCAGTTCTGGAGCCCAGAGTCTTCCTAA

>Rat_T2R114--Intact

ATGCTGGGTGCAATGGAAGGTGTCCTTCTTTCAGTTGCAACTAGTGAGGCTTTGCTGGGCATTGTAGGGAACACATTCATTGCACTTGTGAACTGCATGGACTGTACCAGGAACAAGAATCTCTATAATATTGGCTTCATTCTCACTGGCTTGGCAATTTCCAGAATCTGCCTCGTGTGGATCTTAATCACAGAGGCATACATAAAAATATTCTCTCCACAGTTGCTGTCTCCTATCAACATAATTGAACTCATCAGTTATCTATGGATAATTACCAGTCAATTGAATGTTTGGTTTGCTACCAGCCTCAGTATCTTTTATTTCCTCAAGATAGCAAATTTTTCCCACCACATATTTCTCTGGTTAAAAAGAAGAATTAATATAGTTTTTGCCTTCCTGATAGGGTGCTTACTTATGTCATGGCTATTTTCTTTCCCAGTAGTTGTAAAGATGGTTAAAGATAAAAAAATGCTGTATATAAACTCATCTTGGCAAATCCACATGAAGAAAAGTGAGTTAATCACTAACTATGTTTTCACCAATGGGGGAGTGTTTTTACTTTTTATAATAATGTTAATTGTATGTTTTCTCTTAATTATTTCCCTTTGGAGACACAGCAAGTGGATGCAATCAAATGAATCAGGACTCAGAGATCTCAACACAGAAGTTCATGTGAAAACAATAAAAGTTTTATTATCTTTTATTATCCTTTTTATATTGCATTTAATTGGTATTACCATCAATGTCATTTGTCTGTTAGTCCCAGAAAATAACTTGTTATTCGTGTTTGGTTTGACTATTGCATTCCTCTATCCCTGCTGCCACTCACTTATCCTAATTCTAGCAAACAGCCGGCTGAGACAATGCTTTGTAAGGATACTGCAACAATTAAAGTGCTCTGAGGAAGGAAAAGAATTCAGAAACACATGA

>Rat_T2R144--Intact

ATGGCAATAATAACCACAGATTCCGACTACTATACTCACAGGTATGAAGTGATAATCCCTTTCGTGGTCTCGACCATAGATTGTATCGTCGGCATCATTGGCAATGGCTTCATCACAGTCATATATGGGACTGAATTGGTCAGGAGCAAAAGACTCCCCACTGGTGAGCACCTTATGTTGATGTTGAGTTTTTCCAGGCTCTTGCTACAGATCTGGATAATGGTAGAGATTACCTATCAACTATTTTTCCCCATGATTTATAACCATAATGCCATGTATAAACTATTCAAAACCATCTCTGTGTTTCTGAACTACTGTAACCTCTGGTTTGCCGCGTGGCTCAATGTCTTCTATTGTCTTAAAATTGTGAACTTTGCTCACCCTCTGTTTCTTATGATGAAGCAGAAAATCGTAGTGTTGATGCCTCGGCTCATGAGTCTGTCAGTGTTGGTTTCCATCAGCTTAAGCTCCTTCTTCTCTAAAGACATCTTCAATGTGTATATGAATACTTCAGTTCCCATCCCTTTCTCCAACTCCACAAAGATGAAGTACTTCTTTAAGACCAATGTACTCAACCTGGCTTTCTTATATTATATGGGGATCTTCATTCCTTTGTTCATGTTCATCATGGCAGCCATTCTGCTCATCACCTCACTCAAGAGGCACACCCTGAACATGGAAAGCAGCACCACAGGCTCTAGGGACTCCAGCATGGAGGCTCACTTGGGTGCCATCAAATCGACCAGCTACTCTCTCATTCTCTACATTATCAATGCACTGGCTCTATTTATTTCCATGTCAAATATCTTTGGTGCCTATAGTACCTGGAATAGTGTGTGCAGCTTTATCCTGACCGCCTATCCAGCTGGACAGTCAGTGCATCTGATCTTGAGAAACCCAGGGCTGAGAAGAGCCTGGAGGCGGTTTCAGCACCACGTTCGACTTTACCTTAAAAGATAG

>Rat_T2R14--Intact

ATGGGTGCTGCTATATTACTGATGACTGCAACAGTAGTTAATGTGGAGTTCATACTTGGAAATTTGGGGAATGGATTCATCGCTGTGGCAAACATAATGGATTGGGTCAAGAGAAGGAAGCTCTCTGCAGTGGATCAGCTCCTCACTGTGCTGGCCATCTCCAGAATCACTCTGTTGTGGTCATTGTACATACTGAAATCAACATTTTCAATGGTGCCAAACTTTGAAGTAGCTATACCGTCAACAAGACTAACTAATCTTGTCTGGATAATTTCTAACCATTTTAATATATGGCTAGCCACCATTCTCAGCATCTTTTATTTTCTCAAGATAGGAAATTTTTCTAACTCTATATTCTATTACCTAAGATGGAGATTTAAAAAGGTGGTTTTGGTGGCACTACTGGTGTCTCTGGTCCTCTTGTTTATAGATATTTTTGTCACAAACATACACATCAATATCTGGAAAGATGAATTCAAAGCAAACGTATCTTACAGTTACAAATTAAAGATCTTTTTACAGGTTTCCAGGCTTCTGGTGGTAACTAATACTATGTTCGCATGTGTACCTTTCGTTGTGTCCATGATAATGTTTTTTCTACTCATCTTCTCCCTGTGGAAAAATCTGAAGATGATGAAGCACATTGCCCAAAGCTCCCAAAATGCCAGCACTACAGCCCACATCAATGCCTTGAAAACTGTTGTTGCCTTCCTCCTGCTGTATATCATTTTTATTTTATCCCTTTTTGCACATGTTTGGAGCTATGACTTTGAAGAAAAGAAATATTTTATTTTCTTTTGCCTTGTTGGTATGTTTGCATTACCATCACTCCATTCATACATCTTGATTCTGGGAAACAGTAAGTTGAGGCAGATCTCTCTTTTGGTACTGTCACTGCTAAAGTGCAAGATCCAAGGATGTGAATCCCTGGGCCCCTGGCACACTAGGGGGGATACTTTTACATAA

>Rat_T2R13--Intact

ATGGAACCTGTCATTCACGTCTTTGCCACTCTACTAATACATGTGGAGTTCATTTTTGGGAATCTGAGCAATGGATTTATAGTGTTGTCAAACTTCTGGGACTGGGTCATTAAACGAAAACTTTCCACAATTGATAAAATTCTTCTTACATTGGCAATTTCAAGAATTACTCTCATCTGGGAAATGTATGCTTGTTTTAAAATTGTATATGGTTCATCTTCATTTATATTTGGGATGAAGTTACAAATTCTTTATTTTGCCTGGATCCTTTCTAGTCACTTCAGCCTCTGGTTTGCCACAGCTCTCAGCATCTTTTACTTACTCAGAATAGCTAACTGCTCCTGGAAGATCTTCCTGTATTTGAAATGGAGACTTAAACAAGTGATTGTGGGGATGTTGCTGGCAAGCTTGGTGTTCTTGCCTGGAATCCTGATGCAAAGGACTCTTGAAGAGAGGCCCTATCAATATGGAGGAAACACAAGTGAGGATTCCATGGAAACTGACTTTGCAAGGTTTACAGAGCTGATTCTTTTCAACATGACTATATTCTCTGTAATACCATTTTCATTGGCCTTGATTTCTTTTCTCCTGCTAATCTTCTCTTTGTGGAAACATCTCCAGAAGATGCAGCTCAGTTCCAGAGGACATGGAGACCCTAGCACCAAGGCCCACAGAAATGCTTTGAGAATTATGGTCTCCTTCCTCTTGCTCTACACTTCATATTTCCTGTCTCTTCTTATATCATGGATTGCTCAGAAGCATCACAGTAAACTGGTTGACATTATTGGTATTATTACTGAACTCATGTATCCTTCAGTCCACTCATTTATCCTGATTCTAGGAAATTCTAAATTAAAGCAGACTTCTCTTTGGATACTGAGTCATTTGAAATGTAGACTGAAAGGAGAGAATATTTTAACTCCATCTGGCAAACCAATTAACTAG

>Rat_T2R140--Intact

ATGAAGGTTACTGTGGAGTGTGCATTATTAATCACTTTAATTGTGGAAATCATTATAGGGTGTTTAGGAAATGGATTCATAGCTGTGGTGAACATCATGGACTGGACCAAGAGAAGACGGTTCTCCTTAGTGGATCAGATCCTCACTGCTCTGGCCATCTCCAGACTTGCTTTTGTGTGGTCACTACTCACAGTTTTAGTGATATCTGAGCTTCACTCATCATTGTTGATAACAAGAAAAATGTTGAGGATAATCAATAATTTCTGGACAGTGACCAATCATTTCAGCATCTGGCTTGCTACATGTCTCAGCATCTTTTATTTTCTCAAGATAGCTAACTTTTCAAATTCTATTTTTCTTTCCCTAAGGTGGAGGGTAAAAACTGTGGTTTCATTAACACTGCTGGTATCTCTTCTCCTCTTGCTTGTAAATGTTATCATCATAAACACATGTATTGTTATCTTGGTTGAAGGATACAAAGTAAATATGTCCTACAGTTCTCATTCAAACAACAATCCACAGATTTCCAGGATTCCTTTATTCACCAACACTATGTTCACATTCATACCCTTCACAGTGACTCTGACAATTTTCCTCCTGCTCATCTTCTCCCTGTGGAGGCATTTGAAGAAGATGCAGCATCATGCCAAGAGCCCCAGAGACCCCAGCACCACAGCCCACATTAAGGCTCTGCAAATGGTCGTCACCTTCCTCTTCCTATACACCATTTTCTTTCTGGCACTTGTCATGCAGGCTTGGAAAAATGAGATTCAGTCAAAGACTGTGTTCAACTTGGTTTTTGAGTCGATAGCACTTGCTTTTCCTTCAGGTCACTCCTGTGTACTAATTCTGGGAAACTCTAAGCTCAGACAGGCTTTTCTGACCATAATATGGTGGCTGAGGTCCAGTTTTAATGCTGCAGAACTCTCAAGTCCTTAG

>Rat_T2R15--Intact

ATGGTGGTGACAATGAGGGCTGCCCTACGGCTAATGTTGATAAGTACTGTAAGTCTGGAGCTCATCATAGGAATCTTAGCCAATGTATTCATAGCTCTGGTGAACATCATAGACTGGATTAAAAGAGGAAAGATTTCTGCAGTGGATAAGATCTACATGGGCCTGGCCATCTCCAGGACTGCTTTTGTATTGTCACTAATCACAGGGTTCTTGATAGCATTTTTGGACCCAGCTTCATTGGGAATTGGAATAATGATAAGACTCCTTACTATATCCTGGACAGTGACCAATCATTTCAGTGTCTGGTTTGCTACATGCCTCAGCATCTTTTATTTTCTGAAGATAACCAATTTCTCAAACACTGTTTTCCTTGCCCTCAAATGGAAAGTTAAAAAAGTGGTTTCGGTGACATTGGTGGTGTCTCTGATCATCTTGTTTATAAACGTTATAGTCATACACATATACACTGATAGATTTCAAGTGAACATGGTCCAGAAGTGTGGTGCAAATAACACTTTAAGAGCTTACGGGCTCTTTCTATCCATCAGCACGGTGTTTACATTCATCCCATTCACGACATCCCTGACAATGTTTCTTCTGCTCATCTTCTCCCTGTGGAGACACCTGAAGACCATGCACCACAATGCTACAGGCTCCAGAGATGTCAGCACCGTGGCCCACATAAAAGGCTTGCAAACTGTGGTCGCCTTCCTGTTACTATATACTGTTTTTGCTATGTCACTTTTTTCACAGTCTTTGAGTATTGATGCTCAACATACAAATCTTCTTTCTCACTTTTTACGGTGTATAGGAGTGGCTTTCCCCTCAGGCCACTCCTGTGCCCTGATCCTGGGAAACAATAAACTGAGGCAGGCCTCTCTTTCTGTGATATTTTGGCTGAGGTGTAAGTACAAACATACAGAGAATCAGGGTCCCTAA

>Rat_T2R125--Intact

ATGGGTATTGTCATAGGGATCATATGTGCCTTTATTATAATTGTGCAATTCATAATTGGGAATGTTGCAAATGGATTCATAGCACTGGTGAACATCATAGACTGGGTAAAGAGAAGAAAAATCTCTTTAGTGGATCAGATCATTACTGCTTTGGCTATATCCAGGATAGATATGCTGTGCTCTACATTCTTAATTATACTAATAACTTCATTGTATCCAGATCTAAATACGGCTGTGAACATGGTAAAAATAAGCAATAATATCTGGATTGTTGCCAATCATTTCAGCATCTGGCTTGCTACAAGCCTCAGCATCTTTTATTTCCTCAAGATAGCTAACTTTTCTAACTATGTTTTTCTCTGCTTAAGGTGGAGACTTAGCAAAGTGGTTTCAGTGACATTGCTGCTCTCTCTGGTCCTCTTGCTTATGAATATTTTAATAATGAACATGCATATTGATACCTGGAGTGATGGATTCAAAAGAAACGTCTCTTTTGGCTTCAGATCAAAGAATTGCACTCTCTTTTTCAAACTTGCTCTTTTAATCAACACAACGTTCACGTGTGACCCCTTCACTGTGTCCATGGTGGCGTTTCTGCTTCTCATCTTCTCCCTGTGGAGACACCTGAAGAACATGCAGTACCATGCTAAAGGCTCCAGAGACCCCAGCACTGCCGTGCATATAAAGGCCTTACAAATGGTGGTGGTCTTCGTTCTGTTCTACACATTTTTCTTTTTGTCTCTTGCCATACAACTTTGGACGTCCGAGTCTCTAGAGAAAAACAATCTGTTTTATGTCACTCTTATAATTACTTTTCCTTCAGTCCATTCATGTATGCTGATTCTGAGAAACAGTAAACTGAGGCAGGCATCTCTTTTGGTGCTGTGGTGGCTGCTGTGCAGATCCAAAGACATACAGACTTTGGTTCCCTGA

>Rat_T2R10--Intact

ATGTTCTTACACACAATAAAGCAACGTGATATTTTTACTTTGATAATCATATTTTTTGTGGAAATAACAATGGGAATCTTAGGAAATGGATTCATAGCACTAGTGAATATTGTGGACTGGATCAAGAGAAGAAGGATTTCTTCAGTGGATAAGATTCTCACTACCTTGGCCCTTACCAGACTCATTTATGCGTGGTCTATGCTCATTTTTATATTGTTATTCATACTGGGCCCGCATTTGATTATGAGATCAGAAATACTTACATCAATGGGTGTTATCTGGGTGGTGAACAATCACTTCAGCATCTGGCTTGCTACATGCCTCGGTGTCTTTTATTTTCTCAAGATAGCCAATTTTTCTAACTCTTTGTTTCTTTACCTAAAGTGGAGAGTTAAAAAAGTGGTTTTAATGATAATAGTAGTGTCATTGATTTTCTTGATATTAAACATTTTTTCATTAGAGATTTATGATCATTTCTCAATTGATGTTTATGAAGGAAATATGTCTTATAGCTTGGGGGATTCAACACATTTTCCCAGAATTTTCTTATTCGCAAACTCATCTAAGGTCTTCTTAATCACCAATTCATCCCAGGTTTTCTTACCCATCAACTCACTCTTCATGCTCATACCCTTCACAGTTTCCCTGGTAGCTTTTTTCATGCTTATCTTCTCACTGTGGAAGCATCACAAGAAGATGGAGGTCAATGCCAAAGGACCCAGAGATGCCAACACCACGGCCCACATTAAAGCTTTACAAACTGGGCTCTCCTTCCTGCTGCTGTATGCAATATACTTACTTTTTATTGTCATAGGAATTTTGAGTCATAAATTTATGGGGGGGAAATTGATACTCATATTTGACCACATTTGTGCAATAGTTTTTCCTATAAGCCACTCATTTGTGCTGATTCTGGGAAATAGTAAACTGAGACGAAGCACTCTTTCTGTGCTGCGTTTTCTGAGGTGCCGATCCAAGCATATACACATCATGGATCCCTAA

>Rat_T2R30--Intact

ATGGTGGCCGTTCTACAGAGCACATTTGCAATAATTTTCAGTATGGAGTTCATAGTGGGAACCTTAGGAAATGGATTCATTATTCTGATGACATGCATAGACTGGGTCCGAAGAAGAAAAATCTCTTTAGTGGATCAAATCCTCACTGCTCTGGCAATTACCAGAATCACTCTAATTTTGTTGGTATTCATAGATTGGTGGGTATCTGTTCTTTTCCCAGCATTACATGAAACTGGTAAGATATTAAGAATGTATTTTATCTCCTGGACTGTGATCAATCATTGTAATCTTTGGTTGACAGCAAGCCTGAGCATCATTTATTTTCTCAAGATAGCCAGCTTTTCTAGCATTATTTTTCTTTATCTAAAGTTTAGAGTTAAAAATGTGGTTTTTGTGACCTTGTTAGTGTCTCTATTTTTCTTGTTCATAAATACTGCTATTGTAAATGTATATTTTGATGTTTGTTTTGATGGTGTTCAAAGAAATGTGTCTCAAGTTTCCAGATTGTATAACCACGAACAAATTTGCAAATTTCTTTCTTTTACTAACCCTATGTTTGCATTCATACCCTTTGTTACGTCCATGGCAACGTTCTTTCTGCTCATCTTCTCCCTGTGGAGACATCTGAAAAACATGAAGCACAACGCAGAAGGATGCAGAGACGTCAGCACCATAGTACACATCAGAGCCTTGCAAACCATCATTGTGTCTGTAGTGTTATACAGTACTTTTTTCCTGTCATTTTTTGTAAAAGTTTGGAGTTCTGGGTCACCGGAGAGATACCTGATCTTTCTGTTTGTCTGGGCTCTGGGAAATGCTGTTCTTCCTGCTCACACGTTTGTCCTGATTTGGGGAAACTGTAGATTGAGGTGGGCCTCTCTCTCCCTGATGTTGTGGCTCAGGTACAGGTTCAAAAATATAGACGTATAG

>Rat_T2R39--Intact

ATGCAACATAATTTGAAGACAATATTTGTTATCTCTCACAGCACACTTACAATCATTTTATTCACTGAATTAGTAACTGGAATTATAGGAAATGGGTTCATGGCCCTGGTGCACTGTATGGACTGGCTAAGGAGAAAGAAAATATCATTAGTTAATCAAATCCTCACTGCTTTGGCAATTTCCAGAATTTTTCAACTCTGTTTATTGTTTATAAGTTTAGTCATCTCCTTTTCATATCCAGATTTAACTACAACTTCACTGATAAAAGTCACTTGTAATCTTTGGATTATAGTCAACCATTTCAACATCTGGCTTGCTACATGCCTCGGTATCTTTTATTTTCTCAAGATATCCAATTTTTCTAACTCTCTTTTTCTTTATCTAAAGTGGAGAGTTGAAAAAGTAGTTTTAGTTACACTGCTGGTGTCACTGGTCCTACTGACTTTAAATAGTTTACTAATTAACTTGGAAATTAACATATGCATAAATGAATACCAAAGAAACATAACATACAGCTTCAATTCTTATTATCATGCAAATTGTCACAGGCAGATGTTAAGCCTTCATATTATTTTCCTGTCTGTCCCCTTTGTTTTGTCCCTGTCAACTTTTCTCCTGCTCATCTTCTCTCTGGGGACACATCACAAGAAGATGCAGCAGCATGTTCAAGGACGCCGAGATGCCAGTACTATGGCCCACTTCAAAGCCTTGCAAACCGTGATTGCCTTTCTCCTACTATACTCTATTTTTATTCTGTCTGTCTTAGTACAAATTTGGAAATATGAATTACTGAAGAAAAATCTTTTCATTTTATTTTGTCAGGTTGCATATGTAGCTTTTCCTTCATTCCATTCATATATTCTGATTCTAGGAGACATGAAGATGAGACAGGCCTGTCTCTCTGTATTGTGGTGGCAGAAATTCAGGAAAAATTATGTAGAACCTTTAGATCTCTAA

>Rat_T2R121--Intact

ATGGGAAGCAGCCTGTATGATATCTTAACTATTGTCATGATTGCAGAGTTTATATTCGGAAATGTGACCAATGGATTCATAGTGCTGACAAACTGTATTGCTTGGCTCAGTAAAAGAACTCTTTCTTTCATTGGTTGGATCCAGCTTTTCTTGGCCATTTCCAGAGTGGTTTTGATATGGGAAATGTTACTAGCATGGCTGAAATATATGAAGTATTCATTTTCATATTTGGCTGGCACAGAATTAAGGGTTATGATGTTGACCTGGGTAGTTTCCAATCACTTTAGTCTCTGGCTTGCCACCATTCTAAGCATCTTTTATTTGCTCAAAATAGCTAGTTTCTCCAGACCTGTTTTCCTGTATCTGAAGTGGAGAGTAAAAAAAGTGCTCCTGCTGATTCTTCTCGGAAATTTAATCTTCCTGATGTTCAATATATTACAAATCAACACTCACATAGAAGACTGGATGGATCAATATAAGAGAAATATAACGTGGGATTCCAGAGTGAATGAATTTGTGGGGTTTTCAAATCTGGTTTTATTGGAGATGATTATGTTCTCTGTAACACCATTCACCGTGGCTCTGGTCTCCTTCATCCTGTTAATCTTCTCTTTATGGAAACATCTCCAGAAGATGCATCTCAGTTCCAGAGGGGAACGAGACCCTAGCACAAAAGCCCATGTGAATGCCCTGAGAATTATGGTCTCCTTCCTCTTACTCTATGCCACTTACTTCATATCCTTTTTTATATCATTAATTCCTATGGCACATAAAAAAGGACTAGATCTTATGTTTAGCCTAACTGTTGGACTTTTCTACCCTTCAAGCCACTCATTTATCTTGATTTTGGGACATTCTAATCTAAGGCATTCCAGTTGTCTGGTGATAACCTATCTGAGATGTAAGGAAAAGGATTAG

>Rat_T2R24--Intact

ATGGATGGAATCATACAGATCATATCTGCCTTTATTGTAATTATAGAAATCATAATAGGATGGTTTGGAAATGGATTTATAGTTTTGGTGAACTGCATGCATTGGATCAAGAGAAGAAGAATCTCTACAGTGAATCAAATACTCACAGCCTTGGCTTTCTCCAGAATCTACCTTCTTTTGACAGTATTCACTGTTATATTAGCATCTGTACAATACTCAAATATATTGGTAACTAGAAGGGAGGTAAAAGTGATTATTTTCCATTTGATTACCAGCAATCATTTTAGCATGTGGCTTGCTGCATGCCTTGGCCTTTTTTATTTTCTTAAAATAGCTAATTTTTCTAACTTTATTTTTGTTTTCTTAAAGAAGAGAGTTAACAAGGTAGTTTCAGGGACTTTGCTCATGTCTTTGGTCTTCTTGTTTCTAAACACTCTTCTGATAAACTCATACATTGATGCCCAGATAGATGACTACAGAGGATATCTGCTGTATGATTTCACTTCAAATATCACTGTATCATTTTACAGGGTTATTTTAGTCATTAATAACTGTATTTTCACATCCATACCATTTGCACTTTCACAGTCAACTTTTCTCATGCTCATTTTCTCCCTGTGGAGACATTACAAGAAGATGCAACAACATGCACAAAGATGTAGAGATACCCTCACCAATGCTCACATCAAAGTCTTGCAAACAATGATCATGTATGTCCTTCTTTCTGCCATTTTCTTTCTGTTTCTTTCAATGCAAATTTGGAGGAATAAGTTGATGGAGAACATTCTTTTTATCAGGTTTTGTGAAACTGTTGCAGCAGTTTTTCCTTCAGGACACTCATGTGTCTTGATCTGGGGAGACACAAACCTGAGACAGACCTTTCTTTCTGTGTTGTGGTGGCTGAAGCACAGGTTCACCTTATGGGTCCCTAAATTATATTGCAGATAA

>Rat_T2R120--Intact

ATGGATTTGACAGAATGGATCGTCACTATCATAATGATGATAGAATTTCTCTTAGGAAACTGTGCTAATTTCTTCATAATGGTAGTGAACGCCATTGACTGTATGAAGAGAAGAAAGATCTCCTCAGCCGATCGAATTATAACTGCTCTTGCCATCTCCAGAATTGGTTTGTTATGGGCAATGTTAATGAACTGGCATTCACGTGTGTATACTACAGATACGTACAGTTTTCAAGTGACAGCTTTTAGTGGAATTATCTGGGCGATAACTAATCATTTTACCACTTGGCTTGGGACCATACTCAGCATGTTTTATTTATTCAAGATAGCCAACTTTTCCAATTGTCTATTTCTTCATCTGAAAAGAAAACTTGACAGTGTTCTTCTTGTGATATTTTTGGTGTCTTCTTTGCTTGTGTTTGCATACCTTGGGGTAGTGAACATCAAGAAGATTGCTTGGTTGAGTGTTCATGAAGGAAATGTGACGGTAAAGAGCAAACTGATGAATATAGCAAGCATTAGAGATACGCTTCTCTTCAGCCTGATAAACATCGCACCATTTGGTATATCACTGACCTGTGTTCTGCTCTTAATCTACTCCCTAGGCAAACATCTCAAGAATATGAAATTCTATGGCAAAGGATGTCAAGATCAGAGTACCATGGTCCACATAAGGGCCTTGCAAACTGTGGTTTCCTTTCTCTTGTTATATGCTACATACTCTTCCTGTGTAATTATATCAGGTTGGAGTATACAAAATGTGCCAATCTTCTTATTTTGTGTGACAATTGGTGCCTTCTACCCAGCAGGTCATTCTTGTATCTTGATTTGGGGAAACCAGAAGCTTAAACAGTTCCTTCTGTTGTTTCTGAGGCAGATGAAATGCTGA

>Rat_T2R19--Intact

ATGCTGACTATACCAGAAGGCATCCTCCTTTGCTTTATAACTAGTGGTTCAGTACTGGGAGTTCTAGGGAATGGGTTTATCCTGCATGTGAACTGCACTGACTGTGTCAGGCAGAAGTTCTCCACGACTGGCTTTATTTTCACGGGCTTGGCTATTTCCAGAATCTGTGTCATATGTATAATAATCTCTGATGGATATTTAAAATTATTTTCTCCACATATGGTTGCCTCCGATGCCCACATTATAGGGATTTCTTACCTGTGGATAATCACCAATCACACAAGTACATGTTTTGCCACCATCCTCAACCTCTTCTATTTCCTGAAGATAGCAAATTTTTCTCACTACATCTTCTTCTGCTTGAAGAGAAAACTCAATACAATATTTATCTTTCTCCTGGGATGCTTATTTATATCATGGTCAGTTGCTTTTCCACAAACAGTGAAGATATTTAATGATAAAATGAAGCACAGAAATACTTCCTGGAAGTTTCACCTCCATAAGAGTAAGTTCATTATAAACCACATTCTTCTCAACCTTGGAGTCATATTCTTCTGTATGGTGGCCATCATTACATCCTTCCTATTAATTATTTCACTTTGGAAACACAACAGGAAGATGCAGTTGTATGTCTCGAGATTCAAAAGCCTTAACACAGAAGTTCATTTGAAAGTCATGAAAGTTTTAATTTCTTTTATTATCCTGTTAATATTGCACGTCATAGGTATTCTGATAGAAACATTGAGCTTTCTCCGATATGAAAATAAACTTCTACTTATTTTGGGTTTGAACTTTTCAAGCATGTATCCTTGCTGTCATTCATTTATCCTAATTCTAGCAAACAACCAGCTGAAGCAGGCTTCTTTGAAAGCACTGAAGCAATTTAAATGCCATAAGAAAGACAAGGATGTCAGAGAGACATGA

>Rat_T2R21--Intact

ATGCTAAGTATGCTGGAAAGCATCCTCCTTTCTGTTGCCACTAGTGAAGCTATGCTGGGTATTTTAGGGAATATATTTATTGTACTTGTAAACTGTACAAACTGGGTCAGGAATAAGAAACTCTCCAAGATTAACTTTATTCTCACTGGCTTGGCAATTTCCAGGGTTTTTACCATATGGATAATAACTTTAGATGCATATACAAAGGTTTTCTTTCTGACTACGCTTATGCCTAGCAATCTACATGAATGCATTAGTTACATATGGGTAATTATTAACCACCTGAGTGTCTGGTTTGCCACAAGCCTCAGCATCTTTTATTTCCTGAAGATAGCAAACTTTTCCCACTACATATTTCTCTGGTTGAAGAGAAGAGCTGATAAAGTTTTTGTCTTTCTAATTGGATACTTAATTATAACATGGCTAGCTTCCTTTCCACTAGCTGTGACAGTGATTAAAAATATTAAAGTGCATCATAACAACACATCTTGGCTGATCCAACTGGAGAAGAGAGAGTTACTTATAAACTATGTTTTTGCCAATATGGGGCCCATTTCCCTCTTTATGGTGGCCGTATTTACTTGTTTCCTGTTAACCATTTCCCTTTGGAGACACAGAAGGAGGATGCAATCCACTGGATCAAAATTCAGAGATCTCAACACAGAAGTTCACGTGAAAGCCATGAAAGTTTTAATTTCATTTATCATCCTCTTTATCTTATATTTTATGGGTGTTCTCATAGAAACATTATGCTTGTTTCTCACAGAAAATATACTTCTCTTTATTTTTGGCTTCACTTTGTCATCCACGTATCCCTGTTGCCATTCCTTTATCCTAATTCTAACAAGCAGGGAGCTGAAGCAAGCCTCCATGAGGGCACTGCAGAGATTAAAATGCTGTGAGACTTAA

>Rat_T2R105--Intact

ATGCTCAGTGCAGCAGAAGGCATCCTTCTTTCCATTGCAACTGTTGAAGCTGGGCTGGGAGTTTTAGGGAACACATTTATCGCCCTGGTTAACTGCATGGATTGGGCCAAGAACAAGAAGCTCTCTAAGATTGGTTTCCTTCTCTTTGGCTTAGCAACTTCCAGAATTTTTATTGTATGGATATTAATTTTAGACGCATATGCAAAGCTATTCTTTCCGGGGAAGTATTTGTCTAAGAGTCTGACTGAAATCATCTCTTGTATATGGATGACTGTGAATCACATGACTGTCTGGTTTGCCACCAGCCTCAGCATCTTCTATTTCCTAAAAATAGCAAATTTTTCCCACTATATATTTCTCTGGTTAAAGAGGAGAACTGATAAAGTATTTGCCTTTCTCTTGTGGTGTTTATTAATTTCATGGGCAATCTCCTTCTCATTCACTGTGAAAGTGATGAAGAGCAATCCAAAGAATCATGGAAACAGGACCAGTGGGACACATTGGGAAAAGAGAGAATTCACAAGTAACTATGTTTTAATCAATATTGGAGTCATTTCTCTCTTGATCATGACCTTAACTGCATGTTTCTTGTTAATTATTTCACTTTGGAAACACAGCAGGCAGATGCAGTCTAATGTTTCAGGATTCAGAGATCTCAACACTGAAGCTCATGTGAAAGCCATAAAATTTTTAATTTCATTTATCATCCTTTTCATCTTGTACTTTATAGGTGTTGCAGTAGAAATCATCTGCATGTTTATCCCAGAAAACAAACTGCTATTTATTTTTGGTTTGACAACTGCATCCGTCTATCCCTGCTGTCACTCAGTCATTCTAATTCTAACAAACAGCCAGCTGAAGCAAGCCTTTGTAAAGGTACTGGAGGGATTAAAGTTCTCTGAGAACGGAAAAGATCTCAGGGCCACATGA

>Rat_T2R6--Intact

ATGACATATGAAACTGATACTACCTTAATGTTTGTAGCTGTTTGTGAGGCCTTAGTAGGAATCTTAGGAAATGCATTCATTGCATTGGTAAACTTCATGGGCTGGATGAAGAATAGGAAGATCACTGCTATTGATTTAATCCTCTCAAGTCTGGCTATGTCCAGGATTTGTCTACAGTGTATAATTCTATTAGATTGTATTATATTGGTGCAGTATCCAGACACTTACAACAGGGGTAAAGAAATGAGGATCATTGATTTCTTCTGGACGCTTACCAACCATTTAAGTGTCTGGTTTGCCACCTGCCTCAGCATTTTCTATTTCTTCAAGATAGCAAACTTCTTCCATCCTCTTTTCCTCTGGATAAAGTGGAGAATTGACAAGCTAATTCTGAGGACTCTACTGGCATGCTTGATTCTCTCCCTATGCTTTAGCCTCCCAGTCACTGAGAATTTGACTGATGATTTCAGACGCTGTGTCAAGACAAAAGAAAGAATAAACTCTACTCTGAGGTGCAAATTAAATAAAGCTGGATATGCTTCTGTCAAGGTAAATCTCAACTTGGTCATGCTGTTCCCCTTTTCTGTGTCCCTTGTCTCATTCCTTCTCTTGATTCTCTCCCTATGGAGACACACCAGGCAGATGCAACTCAATGTAACAGGGTACAATGATCCCAGCACAACAGCTCATGTGAAAGCCACAAAAGCAGTAATTTCCTTCCTAGTTCTGTTTATTGTCTACTGCCTGGCCTTTCTTATAGCCACTTCCAGCTACTTTATGCCAGAGAGTGAATTAGCTGTAATTTGGGGTGAGCTGATAGCTCTAATATATCCCTCAAGCCATTCATTTATCCTGATCCTTGGGAACAGTAAACTAAAACAGGCATCTGTAAGGGTGCTTTGTAGAGTAAAGACTATGTTAAAGGGAAGAAAATATTAG

>Rat_T2R123--Intact

ATGTTCTCACAGAAAACAAATTACAGCCATTTGTTTACTTTTTCAATTATTTTTTATGTGGAAATAGTAACAGGAATCTTAGGAAATGGATTCATAGCACTAGTGAATATCATGGACTGGCTCAAGAGGAGGAGGATCTCTACTGCAGATCAGATTCTCACTGCTTTGGCCCTTACCAGACTCATTTATGTGTGGTCTGTACTCATTTGTATATTGTTACTATTTCTGTGCCCACATTTGTCTATGAGACCAGAAATGTTTACAGCGATAGGTGTTATCTGGGTAGTGGATAACCACTTCAGCATCTGGCTTGCTACATGTCTTGGTGTCTTTTATTTCCTCAAAATAGCCAGTTTTTCTAACTCTTTGTTTCTTTACCTAAAGTGGAGAGTTAAAAAAGTGGTTTTAATGATAATACTGATATCACTGATTTTCTTGATGTTAAACATTTCATCATTAGGGATGTATGATCATTTCTCAATTGATGTTTATGAAGGTAATATGTCTTATAATTTGGTGGATTCAACACATTTTCCCAGAATTTTCTTATTCACAAACTCATCTAAGGTCTTCTTAATCGCCAATTCATCCCATGTTTTCTTACCCATCAACTCACTCTTCATGCTCATACCCTTCACAGTTTCCCTGGTAGCTTTTTTCGTGCTCTTTCTCTCACTGTGGAAGCATCACAAGAAGATGCAGGTCAATGCCAAAGGACCCAGAGATGCCAGCACCATGGCCCACACAAAAGCCTTACAAATTGGGTTCTCCTTCCTCCTGCTGTATGCAATATACTTACTTTTCATTATCACAGGAATTTTGAACCTTGACTTGATGAGATATATAGTAATACTTTTATTTGACCACATATCTGGAGCAGTTTTTTCTATAAGCCACTCATTTGTGCTGATTCTGGGAAACAGTAAGCTGAGACAAGCCACTCTTTCTGTGCTGCCTTGTCTTAGGTGCCGGTCCAAAGATATGGACACTGTAGTTTTCTAA

>Rat_T2R138--Intact

ATGTTGACTCTGACTCCCGTCTTAACTGTGTCCTATGAAGCCAAGATTTCATTCCTGTTCCTTTCCGTGGTGGAGTTTGCAGTGGGAATCATGGCCAATGCCTTCATTGTCTTGGTAAACTTTTGGGACATGGTAAAAAAGCAGCCGTTGAACAACTGTGACATCGCACTGCTTTGTCTCAGCATCACCCGGCTTTTCCTGCAGGGCCTTCTCCTTCTGGATGCTATCCAGCTTGCCTGCTTCCAGCAGATGAAAGACCCACTAAGCCACAACTACCAAGCCATCCTCACTCTCTGGATGATCGCAAACCAAGTGAGCCTCTGGCTTGCTGCCTGCCTCAGTCTCCTCTACTGCGCCAAGATTGTCCGTTTCTCTCACACCTTCCCACTTCACTTAGCAAGCTGGGTCTCCAGAAGATTTCTCCAGATGCTTCTAGTTGCTCTTCTTTTCTCCGGCGTCTGCACTGCCCTCTGTTTGTGGGACTTTTTTAGCAGATCTCACACCGTGGTCACATCCATGTTGCACATGAACAACACAGAATTCAATCTGCAAATTGAAAAACTCAATTTCTTTTACTCGTTTGTCTTCTGCAATGTGGGCTCTGTGCCCCCTTCTCTAGTTTTCTTGATTTCCTCCGGAGTGCTGGTTATCTCCCTGGGAAATCACATGAGAACTATGAAGTCCCAAACCAGAGGCTCTCGTGACCCCAGCCTTGAGGCCCATGTCAGAGCCATCATATTTCTGGTCTCCTTTCTCTGTTTTTATGTGGTGTCATTCTGTGCCGCTTTAATATCAATACCCTTACTGGTACTGTGGCACAATAAGGGAGGAGTGATGGTCTGTATAGGGATGATGGCAGCCTGCCCTTCTGGACATGCAGCCATCCTGATATCAGGCAATGCCAAGCTGAAGAAGGTCATAGTGACCATTCTATTCTGGTTTCAAAGCAGGCAAAAGGTGAGAAGAGTCCACAAGGTTCTTCCCAGGATACTCTGA

>Rat_T2R33--Intact

ATGAATGGTGTCCTATACATCACATTTACAGTCATTCTGAGTGTGGAAGTTATAATTGGCAACTTTGGCAATGGAATCATAGCACTGGTGAACATCATGGACTTGGCTAAGAGAAGAAAAATCTCTTCAGTGGATCAGATCCTTACTGCCCTGGCCATTTCTAGAATTGTGCTTCTGTGGTTAGTATTAGTGAGTTGGTGGCTATCTATGTTCTACCCAGGACAATGGATGACTGAAGGAATAGATGTCATAGTACATAATGTATGGACAACATTGAACCAGATTAGTCTCTGGCTTGCTACAAGTTTCAGCGTCTTTTGTTTCCTCAAGGTAGCAAATTTTTCCAACACTATTTTCTTTTATTTAAAGATCAGAGTTAAAAAAGTAATGACAGGGACATTGATAATGTTTTTGCTTCTCTTGGGCTTAAATATTATAGTTATTAATGCATCTAAGACCATTCTAATCCCTGAATACAAAGTAAATATGTCAAACAGTTTGAATTTGAAGAACACACAGATTTCTATGCTGTTTCCATTTGCCAACACCTTGTTTGGGTTCATACCTTTTGCTGTGTCTCTGGTCACCTTCCTCCTGCTATTCTTCTCCCTGTGGAAGCATCAGAGGAAGATGCACCACGGTGCCCAAGGATGCAGAGATTCTAGCACCAAGGCCCACATCAGAGTCTTGCAGACACTGATTGCCTCCATCCTCCTGTATTTCGTTTTCTTTCTGTCTCTTGTTGTCAAGGTTTGGATTTCTCTTTTTTTGGAGAGAATGCTTTTGCTTTTGATTACACAGGCTGCAAAAATTGCGTTTCCCTCACTGCACCCCTGGGTCCTGATCCTGGGCAATGCTAAACTCAGAAAGGCTTCTCTCTCTGCACTCCAGTGGCTGAGGTGCAGGCACAAGGATGAACACCGTAGAGTGCAGAGACCTGAGGTTCATTCATGTGGATCATCTTGCATGCCTTAG

>Rat_T2R137--Intact

ATGTTGGGATTCACTGAAGGGATATTTCTGGTTCTGACTGTCACCGAGTTTATTCTTGGAAATCTGGTGAACGGTTTCATTGTGTCAGTCAATGGCAGCCATTGGTTCAAGAGCAAGAAGATTTCTTTGTCTGACTTCATCATTACCAGCTTGGCCCTCTTCAGGATCTTTCTGCTGTGGATCATCTTTACTGATAGCCTCATAATAGTGTTCTCTTACCACACCCACGACTCAGGGATAAGGATGCAACTTATTGATGTTTTCTGGACATTTACAAACCACTTCAGTATTTGGCTTATCTCCTGTCTCAGTGTTTTCTACTGCCTGAAAATAGCCACTTTCTCCCACCCCTCATTCCTCTGGCTCAAATGGAGAGCTTCTAGAGTGGTTGTTGGGATGCTGTGGGGTGCACTGGTCTTATCCTGTGTCTGCACCATGTCTCTGATGAATGAATTTAAGATCTATTCTGCCCTCACTGGAAGCAGAGACACACAGAATATGACTGAATATATCAGATTGAAGAGACATGAATATAATCTGATGCATGTTCTTGGGAATCTGTGGAAGATCCCTTCCTTAATCGTTTCCCTGATTGCCTACTTTCTGCTCCTTCTCTCTCTGGGGAAGCACACACAGCAGATGCAGAAATACAGTGTTGGCTCCAGAGATCAGAGTGCGGAGGCCCACAGGAGAGCCATGAGGATCATCCTTTCCTTTCTCTTATTCTTCCTATTCTACTTTCTTTCCTTTGTAATTTTGTCATCCAGTCGTTTTCTACCAGAAACCAAGATTGCCAGGATAATTGGAGTAGTAATTACAATGTCATACCTTGTGGGTGATTCATTAATTCTTATTTTAGGTAACAACAAGCTGAAGCAGACATTTGTAGCCATACTCCCATGTGAGTGTGGTCATCCAAAGCCTGGATCTAAGAGGTTCTTTGCTTCATAA

>Rat_T2R126--Intact

ATGCTATCAACTGTATCAGTTTTCTTCATGTCGATCTTTGTTCTGCTCTGTTTCCTGGGAATCCTGGCAAACGGCTTCATTGTGCTGATGCTGAGCAGGGAATGGCTATGGCGCGGTAGGCTGCTCCCCTCAGACATGATCCTCCTCAGTTTGGGCACCTCCCGATTCTGCCAGCAGTGCGTTGGGCTGGTGAACAGTTTCTACTATTCCCTCCACCTTGTTGAGTACTCCAGGAGCCTTGCCCGTCAACTCATTAGTCTTCACATGGACTTCTTGAACTCAGCCACTTTCTGGTTTGGCACCTGGCTCAGCGTCCTGTTCTGTATCAAGATTGCTAACTTCTCCCATCCTGCCTTCCTGTGGTTGAAGTGGAGATTCCCAGCATTGGTGCCTTGGCTCCTACTGGGCTCTATCTTGGTGTCCTTCATCGTAACTCTGATGTTCTTTTGGGGAAACCACACTGTCTATCAGGCATTCTTAAGGAGAAAGTTTTCTGGGAACACAACCTTTAAGGAGTGGAACAGAAGGCTGGAAATAGACTATTTCATGCCTCTGAAACTTGTCACCACGTCAATTCCTTGCTCTCTTTTTCTAGTCTCAATTTTGCTGTTGATCAATTCTCTCAGAAGGCATTCACAAAGAATGCAGCACAATGCTCACAGCTTGCAAGACCCCAACACCCAGGCTCACAGCAGAGCCCTGAAGTCACTCATCTCATTTCTGGTTCTTTACGCGCTGTCCTATGTGTCCATGGTCATTGACGCTACAGTTGTCATCTCCTCAGATAATGTGTGGTATTGGCCCTGGCAAATTATACTTTACTTGTGCATGTCCGTACATCCATTTATCCTTATCACTAATAATCTCAAGTTCCGAGGCACCTTCAGGCAGCTACTCCTGTTGGCCAGGGGATTCTGGGTGACCTAG

>Rat_T2R108--Intact

ATGCTCTGGGAACTGTATGCATTTGTGTTTGCCGCCTCAGTTGTTTTTAATTTTGTAGGAATAGTTGCAAATTTATTTATTATAGTGATAATTTCTAAGACTTGGGTCAAAAGTCACAAAATCTCCTCTTCAGATAAGATCCTGTTCAGCTTGGCCATCACTAGATTCCTGACCCTGGGGTTGTTTCTACTGAACACTGTCTACATTGCTACAAACACTGGAAGGTCAGTCTACTTTTCCACGTTTTTTCTCTTGTGTTGGAAGTTTCTGGACTCCAACAGTCTCTGGCTAGTGACCTTTCTGAACTGCTTGTATTGCGTGAAGATCACTCATTTCCAACATCCAGTGTTTCTTCTGTTGAAACGGACTGTCTCTATGAAGACCACCAGCCTGCTGCTGGCCTGCCTTCTGATTTCTGCCTTCACCACTCTCCTATATTTTGTGCTCACACAGATATCACGTTTTCCTGAACACATAATTGGGAGAAATGACACATTATTTGACGTCAGTGATGGCATCTTGACGTTAGCGGCTTCTTTGATCCTGAGCTCACTTTTACAGTTTCTGCTCAATGTGACCTTTGCTTCTTTGCTAATACATTCCCTGAGAAGACATGTACAGAAGATGCAGAGAAACAGGAGCAGCTTTTGGAATCCCCAGACGGAGGCTCACGTGGGCGCCATGAGGCTGATGATCTGTTTCCTCGTGCTCTATATTCCATATTCAATCGCTGCCTTGCTCTATTTCCCTTCCTATATGAGGAAGAATCTGAGAGCCCAGGCTGCTTGCATGATCATTACTGCTGCTTACCCTCCAGGACATTCTATCCTCCTTATTATCACACATCACAAACTGAAAGCTAAAGCAAAGAAGATTTGCTGTTTCTACAAATTGCGGGATTTCGTTAGTAACTGA

>Rat_T2R135--Intact

ATGGGACCCATCATGTCCACAGGAGAAACGAGCACAGCTCATACAGTTCTGGGATGTCAGATTACTGATAAGACAGTCATCACTTTATTTGTCATTTTAGTCTTTTCGTGTCTGGTGGCAGTGGTAGGCAATGGATTTATCATTATAGCATTGGGCATGAAATGGTTGCTCCGGAGGACATTGTCAGCTCATAATAAGTTACTGATCAGTCTAGCAGCCTCTCGCTTCTGTCTTCAGTGTGTGGTGATAGGTAAGAATATTTATGTTTTCCTGAATCCATCAAGCTTCCCATACAACCCTGTAATACAGCTCCTAAATTTAATGTGGGACTTCTTGACTGCTGCGACCATCTGGTTCTGCTCTTTGCTAGGTTTCTTCTATTGTGTGAAAATTGCAACCTTAACCCATCCTGTCTTTGTCTGGCTAAAGTACAGGTTGCCTGGGTGGGTACCATGGATGTTGCTTAGTGCTGTGGGGATGTCGAGCTTAACTAGTATCCTGTGTTTCATAGGCAATCATATGATATATCAAAACTATGCAAGGAGGGGCCATCAACCTTGGAATGCCACTGGAAATAGCTTAAGACACTCGATTGAGAAATTCTACTTCATTTCTATAAAAATAATCATGTGGACAGTTCCTACTGTTATCTTTAGTATCTTTATGAGTTTGCTCCTCGTATCTTTGGTAAGACACATGAAGAAGACTCTCTTGGCCCTCTCAGAACTTCGGGATGTTTGGGCACAGGCCCACTTCAAAGCCCTTCTTCCTCTGCTCTCCTTCATCATCCTTTTTATCTCCTGTTTTCTGACACTGGTACTCAGTTCTGCCAGCAGCACACCGTATCAGGAATTCAGGTACTGGATGTGGCAGGTGGTGATTCATCTGTGCACAGTGATACACCCCATTGTTATACTCCTCAGTAACCCCGTTTTGAGAGTGGTGATGAAGAGGGGCTGCTGCTGA

>Rat_T2R124--Intact

ATGGTATCTGTCCTGCACAGCATCTCCACCATTATAATAATCGCAGAGTTCGTTTGGGGAAATTTAAGCAATGGTTTGATAGTACTGAAGAACTGTCTTGATTGGATCAATATAAAAGAGCTCTCCACACTTGATCAGATACTCATTCTCTTGGCAATTTCCAGAATTAGTCTCATCTGGGAGACATTACTCATGTGGGTTAAAGATAAACTAATTTCATCTATTACCATCGAAGAATTGAAAATGATTATGTTCAGTTTTATGCTATCTAGCCACTTCAGTCTCTGGCTTGCTACAGCTCTAAGCACCTTCTATTTATTCAGAATAGCTAACTGCTCTTGGCAGATCTTTCTCTACTTAAAATGGAGACTAAAACATCTGATCGTGCAGATGCTACTGGGAAGCGTGATGTTCTTGATTGCAAATATAATACAAATAACCATCACTCTTGAAAAGAGGTTCTATCAATATAAAGGAAACACAAGTGTGAATTCCATACAGAATGAGTTTGCACTTTTGATAGAGATGATGTTATTTAACATGACTATATTTTCTGTGATACCATTTTTATTGGCCCTGATTTCTTTTTTTCTGCTAATCTTCTCTTTATGGAAACATCTCCAGAGGATGCAGCTCAATTCCAGAGAAGATAGAGACCCTAGTACCAAGGCTCACAGGAATGCCTTAGGAATTATGGTCTCTTTCCTCTTGCTCTATACTATGTATGTCCTCTCTCTTCTTATATCCTGGATTGCTCAGAAGAATCAAAGTGAACTGGTTCACATTATTTGTATGATAACTTCACTCTTGAATCCTTCAGTCCACTCATCTATCCTGATTCTGGGTAATTTTAAATTAAAACAGAGTTCTCTTTGTATACTGAGGCACCTGGGATGTAGGCTGAAATCACAGAATACACCAACTACATAA

>Rat_T2R139--Intact

ATGGCGCAACCCAGCAACTATTGGAAACAAGATTTGCTACCACTGTCCATCTTGATCCTAACACTTGTGGCCACTGAGTGCACCATAGGTATCATTGCAAGTGGGATCATCACAGTTGTGAATGCAGTGTCATGGGTTCAGAAAAGGGCAGTTTCCATAACTACTAGGATTCTGCTTCTTCTGAGCGTATCCAGAATAGGCCTCCAAAGCATCATCTTGATAGAAATGACTTCCTCCATATTCAACTTTTCTTCTTACAACAGTGTTTTATATAGAGTCTCAAGGGTAAGCTTTGTATTCCTAAATTATTGTAGCCTCTGGTTTGCTGCTTTGCTTAGTTTCTTCCACTTTGTGAAGATTGCCAATTTTTCTTACCCCCTGTTCTTCAAGCTAAAGTGGAGAATTTCTGAATTGATGCCCTGGCTTCTATGGCTCTCGGTGTTTATTTCCTTCAGCTCCAGCATGTTCTTCTGCAATCATAAATACACTGTGTACAACAACATTTCTCTAAGTAGCAACATCTGCAACTTCACAATGGAACTCTATGTCGCTGAGGCCAATGTGGTCAATGTGGCCTTTTTATTCAGTTTTGGAATCCTCCCACCTCTGACCATGTTCATTGCAACAGCTACTCTTCTAATTTTTTCTCTCAGGAGACACACCCTGCACATGAGAAACGGTGATGCTGACTCCAGAAATCCCCGAGTAGAGGCTCATAAGCAGGCCATCAAGGAAACCAGCTGCTTTCTCTTTCTCTACATCTTATATGCAGCTGTTCTGTTTCTGTCCACATCCAACATAGCTGATGCCAGTCTCTTCTGGAGTAGTGTTCTCAGAATCAGTCTGCCTGTCTACCCAGCTGGCCACTCAGTTTTACTGATTCAGAGCAACCCTGGCTTAAAAAGAACGTGGAAGCAACTTCTGTCCCAAATCCATCTGCACTTACAAAGTAGATACTGA

>Stickleback_T2R1--Intact

ATGGACTTTCAAACTTATGCCGCACTTAATGGGTCCCTCGCTGTTCTTAACATTGTGACTATTGCATTTTACATTTTTTGCTTGGTCCGTCCACTGCACGGAGAAAAAATCAAGCAGCCTCTGAAGCTTCTCCTGTGGACTTTGATTGGCTGTACAAAAACTTTCCTTTTGTCAGGTTTTGTGTCAGGTGTTACAGACGGCTTTTCTTTTCAATCAGCTGTCAGCTCCAAGATAAACCAAATATTTTATCTGCTGATGATCAGCAGTGTTTCCACCAGCATGACCTCCTCAGTCTGGCTGAACTTCTTCTACTACAGCCAGATTGTACCTGCACACAGTGCTCTCTTCATCTGGATTAAGAACAATGTCAAATCCATCATCTACGGCTTTTGGATCACTGAAAGGATTTACTGTCTGTTTGACTTTACATCCATGTTTTTACGCTTTACTGATTTTGATTTATTATTAATCAGAAACAATTTCACGATGGTTGATGACATGCCTGAGAACAATTTCTATAAAGAGATGTTTTGGATTGTGTTTTACACACTGAGGGCCCACTTTGTGTTCTGTCTGTGTGTGATGGTGATGTCCAGTGGTTCTACTGTACTCTACCTGTGTGGACACATGCGTCACATGGCAGCAAACGGACAACCTGCGTCCAGTCCCAGGTTTAGGAATCAGGTGAGAGTCACCGTCACCGGCCTCCTGCAGGGAGTTCTGTATGTTTTCTCTGCCTCTTGGATTATTTACAGTTTTTTTCCTAAGAATGATTTATACATCGATTTTACAATGATCGATTCCACTGTGATCATGTCGTACATGTCAGCCACTTTGTTCAACCTGGGAGCTGGTCAGGCTGTATTCAGGCAGCGAGCAGAACACATCTGGCTTAGAGCAGCTCACTGCTTCAAAGCACCTCAAGTACAACAGACTGATCAAGGAGCGTGA

>Stickleback_T2R2--Intact

ATGGACTTTCAAACTTATGCCGCACTTAATGGGTCCCTCGCTGTTCTTAACACGATGATTATTGCATTTTACATTTTTTGCATGATCCGTCCACTGCACGGAGAAAAGATCAAGCAGCCTCTGAAGCTTCTCCTGTGGACTTTGATTGGCTGTACCATAACTTACCTTTTGTCAGGTGTTGTGGCCTTCTTTTCTCAAATATCAGCTGTCAGCTCCAAGATAAACCAAATATTTGATCTGCTGATGATCTGCAGTGTTTCCACCAGCATGACCTCCTCAGTCTGGCTGAACTTCTTCTACAACAGCCAGATTGTACCTGCACACAGTGCTCTCTTCATCTGGATTAAAAACAATGTCAAATCCATCATCTACGGCTTTTGGATCACTGAAAGGATTTACAGTCTGCTTGACTTTACTTCCATGTTTTTACTCTTTACTAATATTGATGTATCTTTAATCAGCAACAATCTCACGATGGTTGATGACATGTCTGAGAACAATTTCTATGAAGAGATGTTTTGGATTGTGTTTTTCACACTGAAGGCCCACTTTGTGTTCTGTCTGTGTGTGATGGTGATGTCCAGTGGTTCTACTGTACTCTACCTGTGTGGACACATGCGTCACATGGCAGCAAACGGACAACCTGCGTCCAGTCCCAGGTTTAGGAATCAGGTGAGAGTCACCGTCACCGGCCTCCTGCAGGGAGTTCTGTATGTTTTCTCTGCCTCTTGGACTATTCACAGTACTTTTCAAAAGGATGGTATTATATACATCGGTTTTACAATGATCGATTCCACTGTGATTAACTTGTACATGTCAGCCACTTTGTTCAACCTGGGAGCTGGTCAGGCTGTATTCAGGCAGCGAGCAGAACACATCTGGCTTAGAGCAGCTCAGTGCTTCAAAGCACCTCAAGTACAACAGACTGATCAAGGAGCGTGA

>Stickleback_T2R3--Intact

ATGTACGTGATGAATAAATCGACCATCTGGGTACTGACTGGCCTGTTGGCCGTCACAACTGTCTTCTTCAATGTTTACATCTTCCTGATGAGCCTGTTGAGCTACAGGCAGAACAAGAAGTGGAGTCCCTGTGAAACCATCATCCTGGCTCTGTCGCTGGCCGACGTCGCCCACCAGCTGGTCTGCTACCTCTGGATGACCATGGATGAGGTGGACAGTAAGTGCCGCATCGCCGACCTGCCCTACGCTGTCATGCTGCTTTTGATCTTCAGCCTCAAGTTCACCATCATGTGGGATACCGGCTTCCTCACTTTCTACTACAGCACCAAGCTGGTCAACGCGCCCAATCACTGCTACACACGGATCCAGGCTGTCATCCTCAAACACGTGAGCTTGGCCGTGTTGCTCATCCCTCTGTGCGCCTTGGGCACCTGTATGCCGATGCTGGTGGTCTTTCAGTCCACCAACGTCACCGATGGGAACAAAGACTGCGGGCTTTTAATGCCCGCCTCCACCGCCGGCATGGTCTACGAAGTCATCTATCTGCTCCTCGCCGATGTGCTGCCAGGGGTGCTCATGGTGAAATGCTGCATCTCCATCTCCGTCCACCTGGCGTTGCATCTCCGCCACATGAAAGCCACCACCAACGGAACCCACGGGCCCAAGCTGGGCTCTCAGATGAGGGTGATCCGGATGGCTCTATCCCTGGTGGCCGTCTTCGTCCTCTTCCTGGTGATAGACCTGTACGTCAACTACCAGATAACGATGAACCACGAGAGCATCCTCACGCTCACCTTCCTGTTCACCTCCGTCTACACAACGGTCACCGCCATGGTGCTGATCTACGGGAAAAAGACTTTCTGGAAAGCGCTGATACACGAGGTCAACGTCTGCCTGGATGAATATCCGTGTTTGTCTTGTTTGAAGGTGCCTGAACATAAAGCTCAAACCGGCACTACTGCAAAAGATTAA

>Zebrafish_T2R1--Intact

ATGAGCACCGACGTTGGGAACGTTCTTTTTTTCGTTGGAGTTGGGGTTGTCGGTGTTTCTGGAAACATATTCAACCTTATCTTTAGCTTACAACAGCAAGTAAAAACCAGATCCATTCAGACTGTGGGCTTAATCCTAGATGTCATCTCCATCAGCAACATCATCTTGGTACTTTCCACTCTCGCCATGGTGGTTAGTGTGTTTCTGAATGCCCACATTTGGTGCATCAAGCCATATCCTCTCGGTCTCCGTTTTGAAATGTATCTAATGATGACTTGCGGCTTCATCAGCTTCTGGGCCATTGCTTGGCTGAGTCTTTTCTACTGCATCAAAGTTGTGAATTTCTCCTCTGAGATCTTCAGAACATTGAAGAAGAACATCTCAACTGTGATCAACACTGCGGTGACGTTGAGCTGCTTGTTCTCCTTCTTGCTATTCCTTCCAGCGTTCAGCCTCGATCTTCCAGATTCAGCGGATAAAAATATCAGCGAGACAAATATCACAACCTGTCCACAGCCGACTTTCACTCTACAGATAGACATAAATGCATACGCAGCCGCTGTCCTGCTCCTCATCTGCCCGATCCCTCTGATGATCATGTTGCCCACCTCTGTCAGAATGGTGGTCCACCTCTGCGCCCACACACGGGCACTCCAGAAGAACCAGACGCAGGTGCAGGGATCCGACTCGTATCTCTTGGTGTGCAAACTCACCATCTCTCTGGTGGGAGTTTATCTGTCCACTCTATTTATGGTGGCATTGTATTTCATTATAAAGGTTTTGGGGGCATTTATGACATATCAAGCCCTAGTCAGTGCCTTTACTTTTTACTGTGGAATGACTTCAGTGCTACTGACGGCATCAAACAGGTATCTGAAAGATAAACTTTGGAGTCTGTTCTGTTGCAGGAAAGCAAAGGAGCCAGTTAGCAAAAGTCAGACAGTTGTGACACAGGATGTCTGA

>Zebrafish_T2R2--Intact

ATGTCATATCAGTGCAGGACTCTGAAGAGAAAGATGAGCACCGACGTTGGCGACGTTCTTTTTTTCCTTGGAGTTGGGGTTGTCGGTGTTTCTGGAAATATATTCAACCTCATTTTCACTGTACAACAGCAAGTAAAAACCAGAACCATTCAGACTGTGGGCTTGATCCTAGATGTCATCTCCATCAGCAACATCATCTTAGCACTGGCTATTCTCAGCATGGTGGTTGGCATCTTTCTAAATCCCCAAATCTGGTGCATCAAGCCATATCCTTTTGATCTCCGTCTTGAAATTTATCTAATGTTGACTTGCGGCTTCATCAGCTTCTGGGCCATTGCTTGGCTGAGTCTCTTCTACTGCATCAAAGTTGTAAATTTCTCCTCTGAGATTTTCAGAACACTGAAGAAGAACATCTCAACTGTGATCAACACTGCGGTGCTGCTGAGCTGCTTGTTCTCCTGCTTGTTTTTCATCCCACTGTTCAGCCTTGATACTGTGGATTCAACGGAACAAAATGACAATGCGTATGGTAATGTGACCTGTCCAATGCCTTCTTTCACTATTCAGATGAACCAAGATGCATACTCAGCTGCTGTTCTGTTCCTCCTCTGCCCAATTCCACTGATGATCATGTTGCCCACCTCTGTCAGAATGGTGGTCCATCTATGCGCCCACACACGGGCACTCCAAAAGAACCAGACGCAGGTGCAGGGATCCGACTCATATCTCCTTGTGTGCAAACTCACCATCTCCCTGGTGGGAGTTTATCTGTTCAACCTATTCTTTGTGTCTTTGTTCATCCTTATGAAGCTAATCGGGGCATATATCACATATCAATACCTAGTCAGTACTTTTACCTTTTACTGTGGAGTGACTTCAGCACTCCTGACGGCTTCAAACAGGTATCTGAAAGATAAGCTCTGGAGTTTGTTCTGTTGCAGAAAAGCAAAGGAGCCAGCTAGCAAAAGCCACACAGTTGTGACAGGGGATGTTTGA

>Zebrafish_T2R3--Intact

ATGGGTTTCTTTTTTGTTTACATTAGCTTCTTGGCCTATGCCTTAGTCAATGTGCCAGTTTCTATTATCACTATCCTGATGAACGTATTTTTTGTGTACTGTATGTTTTCTTCAGAGAAAGGACAAGCAAACAGTGTAAAACCGCCGCTGAATGTTTTGCTGTGGTCCCTTATTGGATGCAGTCTTCTTCATAACATTTTCAACCTTTTATTTGTTTTGTATGAATTAGTTTACCCACCTGTCTGGCTGTACATTATTTCAGGTGCTACTATACTTTTCGCCATGAGGACAAGTTTTACTGCATGCCTCGGTCTGCAAATTTGTTACTTCTTGCAGATTGTTCCAGTCCGATGGCCTTGCTTTATCTGGATGAAGAAGCACATCAAACTCTTCATGTACGTCTTGTTGTTTCTCGACAGACTCTACTTTCTGTCTCAATATGTCATACGTGTTTTTCTTGAGATTCGGAGGGTAGTGATGTCCTTTAATTCATCCAGCGTTTATGACAACACCACCAGCCAGTCAGCAGATTTCGGATACTACATGTTTATCGCAGACTTTTGGCTGAAATGTTGCTATTTTTTCATTTGTCTTGGCATTATGCTGACGTCAGGCATCACGACTGTCGTCTACTTGTGGAAGCACATGAAGAGAATGAAGGAGAACACCAGCTCTTTATCTGCTCTTTGTAAAAGGCAGCAGATGAGAGTGACCATCATGGGCATCATTCAGACGGTCCTCTTCTTCTTTGCTTCAGGGTGGCTCATGACTGAAGAGTTCATTGAGTGTTATTTTGGTGGTTATGATGTGGGCACCCATTTGGCTTCTACTGTAATGGCACTGTATTCTCTTGGAACGACCTTACTTTTAGGGATTGGTCAGTCCAAATTCCGACTTCTGGCTAAGGATATCTGCAAAAAGACAAGAAAACCAAAATCCTGA

>Zebrafish_T2R4--Intact

ATGGAGCCATGGCTGTACGCTTTAATCTCCAGTCCCCTCTGCCTCATAGGCATGGTTTTCAACCTCCTCTTCTTTTTCTGCCTCATGCGGCCAGTCTCCGGAGTGACGCTTCGTAATCCACTGCGCTTCTTGTTAATTGTTGTGCTCGTCAATTCTACATTTCAATATCTGGTCATAGCCGTGACCATTATTATGCTTCTTTTTGATTACATTTTCTGGCTGGAAACAGTTACCAGAGCTCTGATTTATCAATTCTTTTGCGGAAACTTCTTGTGCAATGCCTGGATCAGCATTTTCTACTACATATCAATTGTTCCTCAACATCATGCCATCTTCATCTGGATTAAGAGGAATATTAAAGCTATTTTATATGGGGGCTTCATTCTAAATCAAATAGTGCTTACGTTTGCTATATCTACGGGGGCAGTGACATATTTTTTTCTTGGGCCCGTCCCAGTTAATTTTACTGCACTTGAACTGAACTCAACAGCACTAGCACAAACCTTAGAGGCAGACATGTTTTTGTTTCATGTGGCAAATTTTTCATATTTGCTATATTGCACCTGCCCGTTGGTCACACTGATAGTCTCTTGGGGCAAAACTTTTTTTTATCTGCGTGGACATATGAAAAAGATGGGACAGAGCGGCGAGTCTTTTTCCCAGCCCCAGCAGAAGAGCCAGATGCGAGTCACAGTGACAGGTATGGTGCAGGCAGCACTGTTCCTACCCAGTAGCCTGTGGACAGTAGCAGCTGCTCTCCTCTACATTACAGGCCTCTTTGAAGAGGTGGATCCCAGCAGGTTTATCACAATGACTTTCTGCTCACTGTCCAGCTTGGGAAATCTGCTGTGTTTCGGATTCTCCCAGTCTGTGTTTCGCCGTGGGATTGTAAGTGTGATTAAAAAGCTAAAAGGTTGA

>Cow_T2R19--Pseudo

ATGCCCTCTTCTATCCTAGGACTGCTGATGCTGGTGGCAGTAGCTGAATCTCTCATTGGCCTCATTGGAAATGGAGTTCTTGTGGTCTGGAGTTTCGGAGAATGTCTCCGAACGTTCAGGGCGTCCTCGTATAACCTCATTGTCCTGGGCCTGGCGGTCTGTCAGTTGCTTCTACAATGGTTGATTATGGTGGACTCAAGTCTGTTCGTGCTTTTCCAGAGCAGCCATTGGCTTCGCTGGCTCAGTGTCTTCAGGGTTCTGGTAAGCCAGGTCAGCCTGTGGTTTGCGAGTTTTCTCAGTGTCTTCTATTGTAGGAAGATCATGACCGTTGAACACCCTGTCTCTTTGTGGCTGAAGCAGAAGGCGTTACCTGAGTTGCTGGTGCTTTCTGGTGTACTTCACGATCCATTTGTTACTTACAGTCAGGGGTAGCTTAGACTTCTCCAGTCCTTCCCAAGGAAACAGCAGCATCTTATTCCCCATTTCAAACTGGCACTATATATGTATATTACAGCTCAATACAGAAAGTATGATGCCTTTCACGATGTTTCCTGTTTCCTCTGGGCTGCTGTGTCTCTTTGTATAGACGCTACAGGAAGATGAAGGTCCATACAGCCGGCAGAAGAGATGCTCAGGCCAAGGCTCATATCACTGTCCTGAAGTCCTTGGGCTGTTTCCTTGTACTTTACATGGTCTACATCCTGGCCAGCCCCTTCTCCATCAGCTCCAAGACTTTTCCTGCAGATCTCTTCACTGTCTTCATCTCTGAGACACTCATGGCCACCTACCCTTTTCTTCATTCTGTCATACTGATCATGGGGAACCCCAGGATGAAGCAGGCATGTCAGAGAATCCTGTGGAAGACTGTATGTG

>Cow_T2R20--Pseudo

ATGATAACTCTACTATCAACCATTTTTTCCATCCTAGGAATAATACAATTTGTTCTGGGAAATTTTGCCAATGGCTTCATAGCCCTGGTGAACTGCATTGACTGGGTCAAGAGACAAAAGATCTCCTCAACTGATGTGGTTGTCACTGCTATGGCAGTCTCCAGAATTGTTTTGTTCTGTGTAATGTTAATACATTGGTATTATATTTTGCTTCATCCAGCTTTATATAGTTTAAAAGTAAGAACTATTTTTCATGTTGCCTGGACAATAAGCAATCATTATAGCACCTGGCTTGCTACTAGCCTCAGTATATTTTATTTGTTGAAGATAGTCAATTTCTCCAGCCTAACTTTTCTTCACCTGAAGTGGAGAGTTAAAAGTGTAGTTCTCATGATGCTTCTGGGAACTTCATTCATTTTGGTTTTACAAGTTGTAGTTATAAGCGTAAGTGGGACTATGCAGAGAAGTGAATTTGAAGGAAACTTCACACAGAAGACCAAACTGAGGGATATTTTATGGCTTTCACATGTGACCCTGCTCATTCTAGGAAACCTCACACCCTTTACTATGTTCTTAATATCTTTTCTGCTACCAATCTTTTCCCTGTGTAAACATCTCAGGAAGATGCAGCTCAATGGCAAAGGATTCCAAGATCCCTGTACGAAGGTCCACATAAAAGCCATGCAAACTGTCATCTCCTTTCTCTTGCTATTTGCCTTTTACTTTCTGGTTCTAATCATATCAATCTGGAGGCCTAAAAAACTGCATGAGGAACCATTTCTCTTGCTTTTCCCAACAGTCAAAGTCATCTATCCTTCAGTCCACTCATTTATCCTGATTTGGGGAAACAGAAAGTTAACACAGGCCTTTCTGTTGTTTCTGTGGCAGCTGGGGTGCTGGCTGAAAGAGAGGAAATAGGTGGGTATCATGTGTCTTCTAGCAAAAAATGAATTGATGGAGTCTGTAACATTTTATACTTTCTACTCCTTTCTTAAT

>Cow_T2R21--Pseudo

ATGATAACTTTAGTATCGAGCATTATTTCCATTCTAATGGTGGCAGAATTTGTTCTGGGAAATTTTGTGAATGGTTTCATAGCACTGGTGAACTGCAATGACTGGCTCAGGAAACAAAAGGTCTCCTTAGCTGATGGGATTCTCACTGCTCTGGCAGTCTGCAGAATTGTTTTGCTCTGGACAATATTAATAAATTGGTATGCAACTATGTATAATCCAGCTCTATATAGTTTAAGAATTGTTATCCGTGTTGCCTGGACAGTAAGCAACCATTTTAGTAACTGGCTTGCTACTAGCCTCAGTATATTTTATTTGTTCAAGATAGCTAATTTCTCCAGCCTAATTTTTCTTCACCTGAAGTGGAGAGTTAAAAGTGTAGTTCTCATGATGATTTTGGGGACTTCAGTGATTTTGTTTTTTCAAGTTGCAGTGTTAAGTATAGATGAGACTATTCAGACAAGTGAATATGAAAGAAACATCACTGAGAAGACCAAATTAAGGGACATTTTACACCTTTCAAATATGACCCTGCTCACACTAACAAACTTCATACCCTTCACTATGTCTCTGGTATCTTTTCTGCTGCTAATCTTTTTCCTGTGGAAACATCTCAGGAAGATGCAGCTCAACGGCAAAAGATCCCAAGATCCCAGCACCAAGGTCCACATAAAAGCCATGCAAACTGTCATCTCCTTTCTTTTCCTGTTTGCCACTTACATGCTGACTGTAATTTTAACAATATGGAATTCTAATGAGCTGCAGAAGGAACTGGTCCAAATGCTTTTCCAGGCTCTTGCAATCACATATCCTTCAATACACTCATTTATCCTGATTTGGACAAACAGGAAATTAACACAGACCTTTCTGTCATTTCTGTGGCAGCCAAGATGCTGGCTAAAAGTA

>Cow_T2R22--Pseudo

GGCAACAGGAGAACTCATCTTAGGAGTGCTGAGAAATGGGTCAAGAACAGGAAAGTCTCATCAGCTGGTTTCATCCTTACCTGCTTAGCTGTGGAGAGAATCATTCAAATGTGGGTAACACTATTGGGTTCATTTACAGCGGGGCTATTTTCACATCTGTATGCTACCAGCAAACTAGCAGAGGTGATTACTCTTTTTTGGGCACTAACGAATCACTTAACTACCTGGTTTGCTAACCCCTAAGTGTGTTCCATTTCTTTAAGATAGCCAATTTCTCTCATTTCTTCTTCACATGGCTGGAGTGGAGAAGGAACAGAGTCATTCTTATACTTTTCCTGG

>Cow_T2R23--Pseudo

GGGAATGGATTTATTGGATTTATAAGCTGCATTGATGGTATGAAAAACAAGAAGATCTCTACTATCAGCTTTATTCTCGCTGGCTTAGCAACTTCCAGAGTTTGCCTGATATGGACAATAGTTACTGATGGATTTTTAAAGTTATGCTCTCCAGATGTACATTCCTCTGGGAACCTAATTGAATATAATGGTTACTTGTGGATAGTGATGAATCAATCAAGTATCTGGTTTGCTACCTGCCTCAGCATCTTCTATTTCCTGAAGATATCTAGTTTTTCCCACTGCATCTTTCTCTGGTTGAAGGGTAGACTCAACATGGTTGTTTTCCTTCTTTTGGGATGCTTGCTTATTTCATGGTTAGTTACTTTTCCACATTTTGTGAAGATTGTTAATGATGATAAAAGGAAAATAAAAACACAGTCTGGTCAATGGATATGCATAAAGGTGAACTCTTTGGAAAACAAATTTGGCTCCATCTTGGTGTCATTCTCCTTTTTATACAATACCTGATTATATGTGTCTTGTTGCTCACTTCTCTTTGGAGACACAACAGGAGGATGCAATTGAATGCCACAGGATTCCCTTTCCCCAGTACAGAAGCACATATCAAAGCAATAAAAGTCTTGGTATCTTTTATCATCCTCTTTATCTTGAATTTTGTAGGTACTGCCATACAAATATCTAGTGTGACAGTGCCTGAAAACAAACTGCTTTTTATTTTTGGTATGACAACCACAGTCCTCTATCTCTGAGGTCACTTGCTTATCCTAATTCTAGGAAATAGGAAGCTCAAGCAAGCCTCTTTGAGAGTACTGAAGTCATTAA

>Cow_T2R24--Pseudo

ATGTTCAGTATAGAAGACCACATCTTTCTGACCATAATGACTGCGTAATTCATCATAGGAATGTTTGTGAATGGATGCATTGGACTAGTAATATGTGTTGATTGGATTAAGAAGAAAAAGATCTCCATAGCTGACTACATCCTCACCAGTTTAGCTCTCTCCAGAATGTATTTGCTTTGTGTAATGACACTCAACGGCACCATACTGGCACTCTACCCAGGTGTTTATGAAAATGAGAAAATAAAGGTAGTTCTTAATATCTTCTGGACATTCACCAACTACTTAAGTATGTGGTTTGCCACCTGCCTCAATGTCTTCTGTCTCTTCGAGATAGCCAATTTCTCCCACCGACTTTTTCTCTGGCTGAAGTGGAGAATTGAGAGGGTGGTTCACTGGAGCCTACTGGGGTCCCTGGCCATTTCCATGTTGATCAGCCTTATACAAGCAACGTTAACAAATTCTGATTATGATTTTCTTAAAATTGCAAAACATAAAAGAAACGTCACCGAATTGTTCCATGTGAGTAAAATTCAATACTTCGACCCATTGACATTGTTTAACCTGTTTGCTATTATTCCATTTACTGTGTCATTGATCTCATTTTTCTTTTTAATTACATCCCTGTGGAGATACAGTAAACAAATGAAATCCAGTGTTACAGGCTCCAGAGACTCCAGCACAGAGGCCCACGTGGAGGCCAGGAAAACAGTGACCTCATTTCTTTTCTTCCTTTTTGTATACTACCTGGCCTGTCTTTTGGCAACATTTAGCGACTTTATGAAAGAAAGCAAGTTAGCTATGATGTCTGGAGAGATTATAGAAATTCTTAATCCCTTAGGTCACTCACTGTTTTTTATTGTTGGAAATAACAAGCTGAGGCTGGCATCTGTCAGGACGCTGAGATGTGGGAAAACAGCCTGCATGATGTAA

>Cow_T2R25--Pseudo

GCAGTAGCTGAATCTCTCATTGGCCTCATTGGAAATGGAGTTCTTGTGGTCTGGAGTTTCGGAGAATGTCTCCGAACGTTCAGGGCGTCCTCGTATAACCTCATTGTCCTGGGCCTGGCGGTCTGTCAGTTGCTTCTACAATGGTTGATTATGGTGGACTCAAGTCTGTTCGTGCTTTTCCAGAGCAGCCATTGGCTTCGCTGGCTCAGTGTCTTCAGGGTTCTGGTAAGCCAGGTCAGCCTGTGGTTTGCGAGTTTTCTCAGTGGACATTCACCAACTACTTAAGTATGTGGTTTGCCACCTGCCTCAATGTCTTCTGTCTCTAAGGCGTTACCTGAGTTGCTGGTGCTTTCTGGTGTACTTCACGATCCATTTGTTACTTACAGTCAGGGGTAGCTTAGACTTCTCCAGTCCTTCCCAAGGAAACAGCAGCATCTTATTCCCCATTTCAAACTGGCACTATATATGTATATTACAGCTCAATACAGAAAGTATGATGCCTTTCACGATGTTTCCTGTTTCCTCTGGGCTGCTGTGTCTCTTTGTATAGACGCTACAGGAAGATGAAGGTCCATACAGCCGGCAGAAGAGATGCTCAGGCCAAGGCTCATATCACTGTCCTGAAGTCCTTGGGCTGTTTCCTTGTACTTTACATGGTCTACATCCTGGCCAGCCCCTTCTCCATCAGCTCCAAGACTTTTCCTGCAGATCTCTTCACTGTCTTCATCTCTGAGACACTCATGGCCACCTACCCTTTTCTTCATTCTGTCATACTGATCATGGGGAACCCCAGGATGAAGCAGG

>Cow_T2R26--Pseudo

CTCTCGCCCATGTTGATATTCAAGGTCATCTTTTTCCTGGAGTCATTGGTTGCTGTGCTGCAAAATGGCTTCATAGTTACTGTGTTGAGCGGGGAGTGGGTGTGAAGCCAGATGCTGCCCGCTGGTGACATGATTGTGACCTGCCTGGCTGCCTCCTGGTTCTGTCTGCATGGGATGGCCCTCCTGAACAACATCATGGCCTCTTCTGGCTTTTGTTCCAAAATCGACTATTTCAGCATTCCCTGGGATTTCATCAACTGCCTCAGTTTCTGGCTGACTGCCTGGTTTGCTGTCTTCTACTGCGCGAAGATCTCCCTCTTCTCTCATCCCCTCTTCTTCTGGATAAAATGGAGGATTTCTCGGTCGGTTCCCCAGCTGGTGCTGGGCTCCTTGATCTTATCTGGTCTGTCCGTCATCTCAGCTGCTGGGAATACAATTCTTGCCCAGATGACGGCTGCCCAGATTTCCCATGGAAACGACACCCTGGCTGGTAGAATACATGCTACCTATTTGCACTTTTTCTACCTCATGTAATTCTCATGTGGTTGGTTCCATTCCTCCTGTTCCTGGTGTCCACCCTCTCGCTCATGTTCTCACTGCGCCGGCACCTCTGGCAGATGCAGGACCACAGACCCAGCCCACGTGATCCCAGTACCTGGGCTCACACCATGGCCCTGACGTCACTTGCCTTCTTCCTCATCTTCTACACCTTGTACTTCCTGTCCCTGGTTATCATTATGTACATCCCAGCCCTCCAGGAACACTGGCACTGGGCCTGTAAGGTGGTGACCTACACGGGCATCTGTCTGCACTCCAGCATCTTGGTGCACAGCAGCCCCAAGCTGAGAAAGGGCCTGA

>Cow_T2R27--Pseudo

ACAGTCCTCTTCATGCTGCTCTTTGTCCTGCTGTGTATCCTGGGCCTCCTGGCCAATGGCTTCATTGTGCTGGTGCTGAGCAGAGAATGGGTGCGACGTGGGAGGCTGCTCCCCTCTGACCTGATCCTCTTTAGCTTGGGACTCTCCCGCTTCTGCCTGCAGTGGGTTGGAATGGGGAATAACTTCTACTATTTCTTGCATCTGGTCGACTACTGCAGTGGTCCCGCCCGGCAGTTCTTTGGTCTACCCTGGGACTTCCTCAACTCTGTCACCGCCTGGTTTGGCTCCTGGCTCAGCGTCCTCTTCTGCATGAAGGTTGCTAACTTCACCCACCCTGGCTTCCTCTGGCTAAAGTGGAGGTTCCCCAGGTCAGTGCCCTGGCTTTTGCTGGGCTCTCTCCTCACCTCCTTCATTGTCACCCTACTGTTTTTTGGGGGGAACCACGCTTTGTATAAAGAGTCCTTCACTAGAAAACCTTTCGGGAATATGACCTACTATCAGTGGAACAGGATTCTGGAAATGTACTATTTCCTGCCCCTGAAACTGATCACTTTTTCAATTCCTGGCTCTGTTTTTCTGGTCTCGATTGCTTTGTTGATTGACTCTCTGAGGAGACACGCATGGAGGATGCAGCACAGTGCTCACAGCCTGCAGGACCCCAGTGGCCAGGCTCACACCAGAGCTCTGAAGTCACTAGTCTCCTTCCTTGTTCTTTATACTCTGTCTTTCATGTCCCTGATCATCGATGGTGAAGGGTTCTGCTCCTCAGAGAGTGACTGGTACTGGCCATGGCAAATTTTACCTACTCGTGCACATCCATCCATCCCTTTATCCTCATCCTTGGCAACCTCAGGCTTCGGGGGGCATTTGGGCAGCTGATTTTGTTGGCCAGGGGCT

>Cow_T2R28--Pseudo

ATGGTGACGGTGAACACAGATGCAATGGATAAAGACACGACCAGGTTTAAGATCGTCTTCACCTTGGTGGTCTCTGCAATAGAGTGCCTCATTGGCATTGCTGGGAATGGCCTCATCACCGTCATCCATGGAGCCGAGTGGGTCAGAGGCAAAAGACTCCCCATTGGACTGCATTCTGCTCATGCTGAGCTTTTCCAGGCTCTTGCTACAGATTTGGATGATGCTGGAAAACACGTACAGTCTGCTGTTCTGGGTCATCTACAATGAAAAAAGAGTATACATACTTTTCAAAACCATCATCATGTTTCTGAACTACTCCAACCTCTGGCTTGCTGCCTGGCTCAATATCTTCTATTGTCTTAGAATCGCAAGCTTTACTCACCCGTGGTTCTCCGTGATGAAAAGGAAGGTCATGTGGCTGATGCCTGGGCTTGTGAGGCTGTCCTTGTTCTTCTCCTTTTGCTCCAGCTTTCCCTTCTCTAAAGGTATATTCAACGTGTATGTGAACAATTCCGTCCCCATCCCCTCCTCCAACTCCACTGAGAAGGTGTACTTCTCCGAGACCAACATGGGCAACTTGGTTACCACCCTTTACCTGGGGATCTTCATCCCTCTGATCATGTCTATGCTGGTGGCCACCCTGCTGATCATCTCTCTCAAAAGACACACCTTCCACATGAAAAGCAATGCCACTGGCTCCAGGGACCCCAGCATGGAGGCTCACCTGGGGGCCATCAGAGCCATCAGCTATTTTCTCATTTTCTACATTCTCAATGCAGTTGCTCTGTTTTTTTTCCATATCCAACATCTTTGCCGCCAACAGCTCCTGGAATATTTTATGCAAAATCATCATGGCTGCCTACCCTGCTGGCCACTCAGTGCTACTGATCTTGGGCAACCCTGGGCTGAAAAGGGCATGGAAGCAGTTTCAGCACCAAGTTCATCTCT

>Cow_T2R29--Pseudo

ATTCTGGTAATAGAATTTATTCTAAGAAATTTTGCCAGTGGTTTCATGTCACTGGTGAACTGCATTGACTGGTCAAGAGACAAAAAATCTCTTCAGCAGATGGGATTCTCACTGCTCTGGCAGTCTCCAGAATTGGTTTGCTCTGAGTAACATTAATAAATTGGTGTGAAAATGTGTTTAATCCCAGCTTTAGACAATTTAAGAGCAAGAATTATTATTATTGCCTGGATAATAAGCAACTGTTTTGGTACCTGGCTTGCTGCTATCCTCAGCATATTTTATTTGCTCAAGATAGCCAATTTTCCAATATTATTTTTCTTTACCTAAAATGGAGAATTAAAAATGTTCTTCTTGTTCACTTTGTCCTGTTTGGTTTTATTAATTCATGGTGTAAATGTAAACAAGACTATCCAGACAAATGACTATGAAGGAAACATCACTCAGAAGACCAAGCTGAGGGACATTTTACACCTTTGAAATATGACTCTGTTCATGCTAGTAAACTTCACACCCTTTGCTATGTCCCTGACTTCTTTTCTGCTGTTTATCTTTTCCCCATGGAAACATTTCAGGAAGATGCAGCTCAGTGGTAAAGGACCCCAAGATCCCAGCACCAAGGTCCATATAAAAGCCATGCAAACTGTCATCTGTTTTCTATTTGCCATTTGCTTC

>Cow_T2R30--Pseudo

ATGATAACTTTACTATGGACCATTTTTTCCATCCTAGTATTAACAGAATTTGTTCTAGGAAATTTTGCCCATGGCCTCACAGCACTGGTGAACTGCATTGATTGGGTCAAGAGACAAAAGATCTCCTCAGCTGATGGGATTCTCACTGCTCTGGCAGTCTGCAGAATTGTTTTGCTCTGGGTAACGTTAATGAATTGGTACTTAGTTGTGTTGAATCCAGTTCTATATAGTTTAAAAGTAAGAATTATTGTTCATATTGCCTGGATAGTAAGCAACCATTATAGCACCTGGCTTGCTACTAGTCTCAGCATATTTTATTTGTTGAAGATAGCCATTTCTCCAGCCTAATTTTTCTTCACCTGAAATAGAGTTAAAAGTGTCATGCACATAATACTTCTGGGAACTTCATTCTCCTTGGTTTTTCATGTTGCAGTGATATACAACGATAAGGCTATCCAGACAAATGAATACAAAGGAAACATTCCTCAGAAGACCATATTGAGGGGCAGTTTATGGCTTCCACATGTGACTCTGCTTATGCCAGGAAATCTCATATGCTTTACTATGTCCCTGACATGTTTTCTGCTATTAACTGTTTGCCTGTGGAAACATCTCAAGAAGATGCAGCTCAGTGGTAAAGGATCTCCAGATTCTAGCACCAAAGTCCATATAAAAGCCATGCAAACTGTGATATCCTTTCTCTTGCTGTTTGCCATTCATTTCCTGGCTCTAAATGGGATCCATTTGGAGTTTTAAAAGGCAGCAGAAGGAAACTGTCTTTTTGTTCTTTGAGGCTCTTGGATTCCTCTATCCTTCAAACCACTCATGTATCCTGATTTGGGGAAACAGGAAGTTAACAAAGGCATTTCTGTCATTTCTGTGGCAGCT

>Cow_T2R31--Pseudo

GGGGAATTTATAATAGGAATTTTAGGGAATGGATTTATTGGACTCACAACTTGCATTGCCTGGATTAGAAATCAGAAGTTGAGCTTGCTTGACTTCATTCTTACTAGTTTGGCCTTTGCCAGAATCAGTCAATTATGGATAACCACTGTCATGTTCTTTTCAATGATGTTCTATCAGGCAGGCTTTGGTACTGTGGGAAGAAAATATATCTTTTTTTGTATCTGGATACTGACCAGTCACTCAAGCACTTGGCTTGCTACTTGCCTTGCTGTCTTTTATTTCCTGAAGATTGCCAGTTTCTCCCATCCTCCTTTCCTTTGGCTAAAATGGAGAATTAACAAGGTTGTTTTCATGCTTCCACTGGTATCTGTGCCCTTCCTAGTCATAAGTTTTCCTTGGCCAAATAATGTTGATGTCTTCTGGTGTCATGTCCAAAAGATGCATGAGAGAAGTATGACTGAGTTATGCAATGTGAATGAATATCAAAATTTAAATTTTATTATTATCTTCACAATGGAGTCCCTCCCACCTTCTTTCTTTCCCTGATTTCCTTTCTCCTGTTGCTCCATTCTTTGTGGAAACACAAGAAGAACATTGCACACACTGTCAGGAATTCCAGAGACCCCCGTGTTGAGGCCCATTTCAGAGCCATGAAAACTGTGTTTTTCTTTCTCATGCTCTTTGTCCTGTACCAATTTGGCCTTTTCATGACATTTGGGGGGCATTTTTTCCTACAGAACAAGCTGGCTGTGATGTTTGGTTATATGTTAGGAATGCTGTATCCTTCAAGTCACTCATATGTTTTAATTTTTGGAAACAGTCAAATGA

>Cow_T2R32--Pseudo

ATGTCAGTTGGAACAAGGGTCTTCTTTCTAGTGGTGTCAACAGGAGAACTTATCTTAGGAGTACTGGGAAATGGGTTCATTGGACTGGTCAACTGCATCGCGTGGGTCAAGAATGGAAAGGTCTGATCAGCTGATTTCATCCTTACCTGCTTGGCTGTGGCCAGAACCATTCTGGAGGTTCTGTGGGTAACACTTTTGGATTCATTTATAGTAGGATTAGCTCCACATCTGTATGCCACTGGCAAACTAGTAAAAGTAGTTTTTCTTCTTTGCTGCTGCTGCTGCTAAGTCGCTTCAGTCATGTCTGACTTTGTGCGACCCCATAGACAGCAGCCCACCAGGCTCCCCCATCCCTGGGATTCTCCAGGCAAGAACACTGGAGTGGGTTGCCATTTCCTCCTCCAATGCATGAAAGTGAAAAGTCAAAGTGAAGTCGCTCAGTTGTGTCTGACTCCTAGCGACCCCATGGACTGCAGCCTACCAGGCTCCTCCATCCATGGGATTTTCCAGGCAAGAGTACTGGAGTGGGGTTGGGCACTAACAAATCACTTAACTATCTGGTTTGCCACCTGCCTAAGCATATTCTACTTCCTTAAGATAGCCAGTTTCTCTCACTTCTTTTTCATGTGGCTGAAATGGAGAATGAACCGAGTGCTTCTGGTGCTTTTCCTGGCCTTTTTCTTTTTATTATCTTTTGACCTCTTAATGCAGGATGCTCTTGGTGAGTTGTGGATGAACACCTTTAGAGAACCTGAAAGGAACATGACTTTGCATTTAGATGCAAGTAAAATTTTCTATCTTAAAAGCCTTATTCTTCTCAGATTGACATATGTTATCCCTTTTCTTCTCTCCTTGGCTTCTTTCCTGCTTTTCTTTCTTTCCCTGGTGAGACACATCAAGAATTTCCAAGTCAACTTGAACCACTCGAGAGACTTCAGCACAGAGGCCGATAAAAGGGCCACGAAA

>Cow_T2R33--Pseudo

TGTGCTGACTTTGTCAAAAGCAAGAATAGTGCCTTGTTTGACTTCATCTTCACATGGATTAGCGTGATGTTCATACTTCTCCTAGATTGCGTTAAACTAGTGTTCCATCTAGAAATATTAGATGGTCACCAGGTAATAAGAGGAGTTTTTGAGTTCTCCTGGAGTCTGAGAAACTCATTAAGTACTGGATGTGCTGCCTGCCTCAGTGTCTTCTACTTCCTCAAGCTATCTAGTTTTTCTCACCCCTTCTTTCTCTGGCTGAAGTGCAGAAGAGATAGAGTTGTTTTCACCATTATGTTGGGATTCTGTCTCTTTTTGATTTTTAACCTTCTGAGCATAAAATTTATACTTTTGTGTTCAGCAAGCATTTAGAAAAGGAAAGACTTAACTTGGAAAAAAGATATGCATAAAAATCAGTATTATAACAGTCAAGTTCTCTTCAGCCTTGGGTCTCTCATCCCCTTGTCTGTATCACTCATTATATTTTTCCTGTTAATCTTTTCCTATGGGGACATACCAAGCAGATGACATGCCATAACACAGATCCCAGGGACTTCAATGCCGGGAGCC

>Dog_T2R16--Pseudo

ATCTTCTTGATTGCCGGAGAAATGACTATAGGAATTTGGGGAAATGGATTTATTGCACTGGTTAACTGCACTAGGTGGCTCAGAAGGAGAGACATCTCCGTGATTGACATCATCCTGGTGATCTTGGCCATCTCCAGAATCTGTTTGCTGTGTGTGGTATCTTTAGATGGCTTTATTTTGCTGCTCTCTCCAGATATATATGCCAATAGCGAGCTAATGAATATTGTGGATGTTGTCTGGACACTTAGCAATCATTCAAGTATCTGTTTTACTTCTTGCCTCAGTATTTTCTATTTACTGAAGATAGCCAATATATCCCATCCGTTTTTTCTCTGGCTGAAGCTAAAGATTAACAGAGTCATTCTGGGGATGTTTCTGATGTCTTTTCTTACCTGTATAATTATTAGTGTTTCATTGAATGAGGACTTCTGGGATCCCTTCAAAGTCAATCATAAGGAAAACATAACTTGGGAATCCAAAGTGAGTAAAATCCCAAGTGCTTTCAAACTGTTTATCCTGAATCTGGGAGCTATAGTTCCCTTTGTTCTTTGCCTAATCTCAGTTCTCTTGTTACTTTTCTCCCTATTTAGACACACTAGGCAGATGAAACTTTATGCCACAGGGTCCAGAGACCCCAGCACAGAGGCCCACATGAGGGCCATAAAGGCAGTGATGATCTTTCTGCTCCTCTTCATTATTTACTATGCAGTCTCTCTTGTAGTAACCTCTAGCTTCCTGATTCCTCACGGAAAATTAGTGGTTATGTTTGGTGGCGTGGTAGTTGGCATTTTCCCATCGAGCCATCGTTCATACTGATAATGGGCAACAGCAAGCTGAGGGGGGCTTTCCTAAAAGTGCTTAGGATTGTGAAGGGTTTCCACAAAAGAAGGA

>Dog_T2R17--Pseudo

ATGCTACCTTTACTACAGAGCATTTTTTCCATCCTAGTAATGACAGAATTTGTTCTAGGAAATTTTGCCAATGGCTTCATAGTGCTGGTGAACTACATTGCGTGGGTCAAGAGACAAAAGATCTCCTCAGCTGATCAAATTCTCACTGGTCTGGCTGTCTCCAGAATTGGTTTACTCTGGGTAATATTAATAAATTGGTATGCAACTCTGTTGAATCCAGCTTTATATAGCTTAGAAGTAAGGCTTCCTGTTCATATTGCCTGGACAGCGAGCAATCATTTTAGCATCTGGCTTGCTACTAGCCTCAGTGTATTTTATTTGTTCAAAATAGCCAATTTCTCTAACCTTATTTTTCTTCGCCTAAAGTGGAGAGTTAAAAGTGTAGTTTTTGTGATGCTGTTGGGGTCTTTGTTCTTTTTAGTTTTTCATGTTGCAATGGTAAGCGTATATGAGCAAATGCAGATGAAGGAATATGAAGGAAACATCACTAGGCAGACCAAACTGAGGGACATTGCACAGCTTATGAATATGACTGTATTCACGCTAATGAACTTTGTACCCTTTGCTATATCCTTAACATCTTTTCTGCTGTTAATCTTTTCCCTGTGGAAACATCTCAAGAAGATGCGATCCAGTGGTAAAAGATCCCAAGGTTCCAGCACCAAGGTCCACATAAGAGCGCAGACTGTGATCTCTTTTCTTTCGTTATTAGTTTGTTAATTCCTGACTTTAATTGCCATAGTTTGGAGTTCTAATAGGCTGCAGAATGGACTATTCTTCATGCTTTGCCAAGTTTTTGCATATGCATATCCTTCAAGCCACTCATTTATCCTGATTTGGGGAAACAAGAAGCTAAGAGAAGCCTTTCTGTCTGTTTTATACCAGGTGAAGTACTGGTTGAAA

>Dog_T2R18--Pseudo

ATGCTCAGTATGGAAGACATCATCTTCATGATCGTAATAACTGGAGAATTCATAATAGGAATGTTGGGGAATGCATGCATTGGACTAGTAAACTCTATTGACTGGATTAAGAAGAAAAAGATCTCCTCAATTGACTATATCCTCACCAGTCTAGCCAAAATTGGTTTGCTCTGTATAATGATACTAAATGGCACCAAAATCGTATTCTGCCCAGATTTTTATAAAAAGGATAAGCTACAAGCAGTCATTAATATCTTCTGGATACTCACCAACTACTTAAGTATGTGGTTCACCACCTGCCTTAATGTCTTCTATTTACTCAAGATAGCCAATTTCTCCCACCCACCTTTTTCTCTGGCTAAAGAGGAGAACTGACAGAGTGATTCACTGGATTCTGCTGGGTTGTTTGGCTCTTTCTTCCTTAATCAGCCTTATACTAGCAATGACACCAAATTATGATTGTGAGTTTTGTAACATTGCAAAACATAAAGGAAACTGAACTGAAATGCTCTCTGTAAGTAAAAGTCAATACTTCAAGCCATTGACTCTCTTTAACTTGTTGGCAATTGTCCCATGTACTGTGTCATTGGTCTCATTTTTCCTTTTAATTATGTCCCTATGGAGACATATCAAGCAAATGAAACTCAACGTTATAGGCTGTGGAGACCTCAGCACAGAGGCGGGAGCCATGAAAACTGTGACTTCATTTCTTTTCCTCCTTTTTGTGTACTATGGGGCTTCTCTTTTGGTAACTTTTAGCTACCTTATGAAAGAAAGCAAGTTAGCTGTGATGTTTGAAGAAATTATAGCAACACTCTATCCTTCTGGTCATTCACTTATTTTGGTTATTGGAAATAACAAGCTGAGGCAGGCATTTATCAGGATGCTGAGATACGGAAGAACAGTCTGCAT

>Dog_T2R19--Pseudo

CTGGCTATCATTTTGTCTTATTTTTTGTTCCTGGTGGCAGCAGTGACCAATAGCTTCATCACTATAGCCCTGGGCATGGTGTGGCTGCTACGGAGAACACTGTTAGCCTTGTGATAAATTATTAGTCAGCCTGGGGGCCTCTTGCCTCTGTCTGCAGTGGGTGGTGAGAGGGAAGAGCATTTATATTTTCCTGTATCCAGGGGCCTTCCCATACAATCCTGTACTGCAGTTCCTGGCCTCCCAGTGGGACTTGTTGAACACTGCCACCTTATGGTTCTTCACCTAGCTGGGTGCCTTCTGTTGCGTGAAAACCCGCAACCATCATTCATCCTCCCCATCTTTTCTGGCTAAAACAGAAGGTGTCTGGGTTGGTTCCATGGGTGCTGCTCAGTTCTGTGGGGCTCCAGCTTGAGCACCTATTTTCATAGGCAATCGGAGCTTACAGCCCTAATATCCCTACTTTTTAAGGAGAGGGTTGCAATCTTGGAAGCCTATTTCTTCTCTTTAAAACTTGTTACCTGGACAGTCTTTGCTGTTGTCTTTCTTGTTGGTATGGCTTTGCTCATGTCTCTGGAAAGACACACTAAGAAGGCCCTGCTCTCCATTGTGGCCTTTGTGACCCCAGTGCCTGGGCACACACCCAGGTTCTCCTGGCTC

>Dog_T2R20--Pseudo

ATGCTTCAGATATTCTTTTTATCTGCCATTATTTTCTCAGCAATTTTGAATTTTGTGGGACTCATTGTAAATCTGTTTATTGCAGTGGTCAGTTATAGGACTTGGCTCAAAAGCCATAGAATTTCCTCTTCTAATTGGATCCTCTTCAGCTTGGGCATCACCAGATGTCTTATGCTGGGACTGTTTCTACTCAACATCATCTACTTCTTCATCTCTCCAAAAATGGAAAGGTCGGTGCACCTATCCCACTTTTTCCTGTCGTAGTGGATGTTTTTGGACTCTAATAGTCTCTGGTTTGTAACCTTGCTCATTGCCTTGTACTGCGTGAAGATTACGGACTTCCAACTTGGAGTATTTCTCCTGCTGAAGCGAAATCTCTCCCCAAAGATCCCCAGGCTGTTGCTAGCCTGTGTACTGATTTCTGCCTTCACCACTCTCCTGTATGTTGTGCTCAAACAGACATCATCCCTTCCTGAATTTGTGACTCAGAGAAATGGTACAGGATGTGGCATCCATGGGAGTGTCTTGTCTTTGGTGACCTCTTTGGTCTTGCGCTCAGTTCTCCAGTTTATCATTAATGTGACTTCTGCTTCCTTGTTGATACATTCCTTGAGGAGACATATACAGAAGATGCAGAAAAACACCACTATTTTTTGGAATCCTCAGACTGAAGCTCATGTGGGCGCTATGAAGCTGATGATCTGTTTCCTCATCCTGTACATTCCTTACTCAGTTGCTACCTTGCTACATTATTTCCCTTATGGTGGGATGGATTTGAGAACCAGATCCATCTGTTTGGTTATTTCCAGCTTTTACCCTCCAGGACATTCTATTCTCATTATCCTCACACATCCTAAACTGAAAACAAAAGCAAAGAAGATTCTTTGTTTCAACAAA

>Frog_T2R50--Pseudo

TGGACCCTGTTTTTTATGTACTTGATGTCGGTTTACCTTACCAGCCTGATAGATTCATAGATAGATGTCATCATGTACATCTTCCTGTGCTTTAGCAATAGCAGTTTGTGGTATGCCACGTGGCTCTGCATGTTCTACTGTGTTTGTATTGTCAACATCAGCAACTGCGTTTTTGTTGTTTTCAAAAAGAATTTCGACCACTGCCTTCCCGTCTTGCTTCTTGGAAGTTTAGCTATATCAGTACCCATGGCTCTTCTTCTTCTCTCCGATAATATGACCGATACGGACTTAATTGACTCGTCAATTTCACAAGCATTAGGGATTTCCATCAAATCCACCTCAAGCTTTCTAATTATTTCTTTCATAGGGAGCGCACCGCCATTTCTAGTTTTCTGCATAGCAGCTTGGCTGGTGGTATATTCCCTTAGGAAGCACACAAGGAGGATGAAGGAACAAGAACGGACAAGTTTCAAGGAACCATCACTAGAAGCTCATTATGGGGCAGTGGGACACTTCTTTCTGTTTTATGCACTTTACGTCATCTCATTTAATCTGTACCAAGCCGGAATAGCCAGCCCTAACTCCTTCGCTGGATTCATTTGTACGATCTTATCCGTCGGTGCATTCGGTGCTCCTTCTTCTCCAGAACAACAAACTGCAACAAACCCTCATCAGTTTTTGGCAAAAGATCAGCTGTTATCAGCATGCCAAAGTGAAAAAAGTAGAGTCTACTTCTACATGTAATACTGC

>Frog_T2R51--Pseudo

ATGCTGACAAAGTTTGAATTGATTTTCACCGTCGCTCTTGTTCTATCATGGACATGTGGGACCATCCTAAACTCATCCATTGTAGCCGTGTATCTCAGTGACTGGAAGAAGGGATTGGACCTCGGTGCCTGCGATCAAATCATTCTCGCCATGGGTTGCACCAACCTACTCCTGCAGTGGACTTTAACATTCCATCTGATGTCTTTGACTTATCAATTCTACGTTCTGCTCACCAAAGAATTACTTCTGGCTGCTGTCTCCACCGTGGTACAGTTTTTCATTGTGCTCTCGTTTTGGCTCACTGCCTGGCTCAGCGGCTACTACTGTGTGCGACTGGTCAACTTCTCCAACAGATTCTTCATTCGATTCAAAAGAGGAATTTCGACTGTGGTTGCCTACTGCCTACTGGGAACGACGGTTACTTTATTTGCTATAGAAATTCCTGTGGTTTGGACCACGCACATAATAACACAATGCGACACATATTAGCATCTTTGATGGTCATGTTGCGCTTGTTTCATTTTACACTGTATTTATTTGCTTCCTTCCAACTATTTTATCTTCCTTCTGTATTGGACTCAGCCTGATGTCCCTTCTGAAACACGTCCGGAAGATGAAGCAGAATACTTCTCAGTTCTGGAACCCTCAGCTGAAGAGCCATGTAAAGGCTTGCAGGACAATGCTACTTCTTTTGGCCGTGAATTTGATATTAGCTCTTGATATCCCTCGAGCTCCAAAATAAGGCTGGGGCTACTGGGGTATATGTAACCTGGTTTACTTTGATGTCAAATCCCTCAGTTAAAGCCATAATTCTTCTCTTTGGGAACTCCAGGTTAGCAACTGCCTGGTCAAAGGTCTTGTTTT

>Frog_T2R52--Pseudo

ATGCTGTCGGCGGTCACGGTGATCAAGACGGTGATACTGATCGTAACATGGCCATGGGGACCATCCTGAACTCATCCATTATAGCTGTCTATTTCCAGCGACTGGAAGGAAGGGAGTGAAACTTGGGGAGTGCGATCAGATCAGTCTCAGTATGGGGTGCACCAACCTGCTCTTGCAGTGCTTAATAACATTGGGAGCGGCCTTCGTCTCATATGGACTGTACCTTCCATTTGCGAAAAAAGTTTTTTTTACTTTCTACACTGTTTTCTTGTTTTTTAATTCTCTCTCATTTTGGCTCACAGCCTGTCTCTCCATCTGTTACTGCCTGCGACTGGTCAATCTCTCCCCTAAATTGTTTATTTGGTTAAAAAGGAGACTCTCTCGCACCGTCGCCCCACTCCTTCTGTGGTCAGTGGCAATTTCATTTGCTTCTATTCTTCCAGTTATCTGGATATCAGACACAGAAACTGACCAGAATACAACTTTAATTTATTATGATAATACCTCTATTGCTAATTGGAGTTTTTTGGTCACTGCTTTTGCATTTGGCATCGGCCTGCCCTCCCTTATTACTGCTATTTGTATTGTACTTTCCCTGATATCGCTCCTGAGGCACATCTGGAGGATGAAGCAGAACCCTCAGTTCGGGAGTCCTCAGCTGAAGAACCTTATAAGAGCTTGCAGGACAATGTTCCTACTCATGGCTCTGAATTGGCTTTACTTTCTGATCATTTTCAGTTCAGTGCTAAAATCAAATAGCATGGACACTATATGGGACATGGTGCTCTTGTTAGGGAATGTGTTGAAACCCTCATGTCAAGCCATTGTTCTGATCTTTGGAAATTCCAAGTTACTCAGTGCCTGGATAAAGACCTTGTT

>Frog_T2R53--Pseudo

CTGGTCAACTTTTCACATGGATTATTTATTAGAATTAAAAGGAGAATATTCTCTACAATCACCCAGTTCGTGTTTGACTTAGGGATGGTTTTTATGCTTGCTTAATGTTTTTTTAATCAATGCATACAGAATTTTTGCAAAATACAACCTTTATCAGCTAATCACTTTATCTACATAGTGGATCTTAAATACATGTCTTTAAATGTTGTCATTGGTTGTTGAGTGCTGGTTCTTGTAACTTCTCTTTGCATTGGACTCAGTATAATGTCTCTTTTGAGATATGTC

>Frog_T2R54--Pseudo

ATGCTGTCTGCCATTCAGATAATAAGAACAATTATTCTGATCATAACCGGACCATGTGGGATCGTCCTAAACTCATGTATTGTAGCTGTGCATCTCAGCCATTGGAAGAAGGGAGTGAGCCTTGGGGACTGTGATCAAATCATTCTCATCATGGGGGTCACCAGCGTTCTCCTCCAGTGCTCATTAACTTTCAATGGGATAGCTGACAATTTTGAACTATATGGGCATTTTGACAAGGAAATCGTTTTTGTGAATGACATGTTCTTCTTATTTCTAAACTTTTTCTGGATTTGGCTCACTGCCTGGCTCGCTATCTGCTACTGCTTGAGACTCGTCAACATTTCACATCGGTTCTTTATTGGCTTAAAAAAGAGAATCTCTTCTGGGGTTACCCTGCTCCTGTTGGGAACAGCGGTGATTTTAGGCGTGATTAATATTCCAATTTTTTGGACACTGAACATCAAAGCAAAGCAAAACATAACCTCCACCTTACCAGTTGACTTCCTTATCTCTGACTCAGACATTAAATACATGTCCTTCACTGCTGCATTTGGTTGCTGCCTGCCAACTCTTATAACTTCTCTCTGTATGGGACTCAGCTTAATGTCCCTTTTGAAACATGTCCAGAAGATGAAGCAGAATCACTCTCAGTCCTGGAGCGGGAAAATGAAGACCCATGCAAGAGCCTGCATGACAATATTCCTCCTTATGGCTCTCAACTTGTTTTTTTTCTTGACAATTTTTCAGTATTATTCTATCAAAATTCAAGATTGACAGTAATGGGAATACTCTCATCTTCTGTATAATCATGGCAAGTCCCGTGGGCCAAGTTCTCATTCTGTTGTTTGGAAACTCAAAGTTACGGTCTGATTTGTTAAAGACTTGTTT

>Frog_T2R55--Pseudo

ATGCTGTCTGCCATTCAGATAATAAAAACAATTATTCTGGTTATAACCGGACCATGTGGATTCATCCTAAACTCATGGATTGTAGCTGTGCATCTCAGCCATTGGAAGAAGGGAGTGAGCCTTGGGGACTGTGATCAAATCATTCTCATCAAAGGGGTCACCAACGTTCTCCTCCAGTGCTCATTAACGTTCACCGGGACAGTCAGTGCTTTTCAACTATATGAGTATGTTAAAATAACTTAGTGGCCTACGTGTTCTTCTTCCTTCTGGCTTCCCTCTGGTTTTGGCTCACTGCCTGGCTCTCTATCTGCTACTGCTTGAGACTCGGCAACATTTCACATTGGTTCCTTATCGGCTTAAAAAAGAGAATCTCTTCCGGGATTACCCAACTCCTTTTAGGAACAGTGGTGATCTTAAGCATAATTAATATTCCAGATTTTTGGACATTTAACATAAAAGCTCAGCAAAACACATCTATCGCCATATTGATGAATTATTTTATCTTAGAACCGGATGTTAAATACCTGTCTTTCAATGTTGCATTCGGTTGCTGCCTGCCAAGTCTTATAAATTCTCTGTGTATTGGACTCAGCCTAATGTCGCTTTTTTTTTTGAGACATGTCCATAGGATGAAGCAGAATCACTCTCAGTCCTGGAGCGGGAAAATGAAGACCCATGCAAGAGCCTGCATGACAATATTCCTCCTTATGGCTCTGAACTTGTTTTTATTTTCAACGGTTATTATTTTGGTTATATCACAATTCAGTTCCGGAACTATTTGGAATACTCTCTTCTGGTCAGCAATCATGGCAAGTCCCTCAGGCCAAGCCATCATTCTGTTCTTTGGGAATTCAAAGTTACGGCCCGGTTGGTTAAAGACTT

>Frog_T2R56--Pseudo

ATGCTGACCATTATGGATATTTTTACATTGTCTATTGACTTATTTTCTGTAGTGGCATCCGCCCCAGGAAATCTGTTTGTTATGCTTTTGAATCTACAGGACTTAGCCAAAAATGTAAAATTTCAGCTTATCAATTGATGTTAAAAGGTATATGTCTGTTGTGCCTCCGATATATCTTTCTATATCTTTCTGCTTTTTCTGGTATTGTATCTGGCTGATTCTATGTATATCTTCATAAGACTTTCCACAAAATGTATCCATAAATTCATATCATGTCGACAATGACAATGGCCTCCTGTTAGCTGGGATGCCACTGAATACTCATCAAATTCAACAAGTTGAGCAGCCAATCAAAATATGTTTTTTCTTACGTCCAATGGAAATTTCTCAAGGTTATCTTCTTGGTCTCCTTTGTGGCTTTTTTGCTCTTCTTTAGTTCATCTTTATTCATTATCATATCACTCTACAGACACATCAGAAGAATAAAAACCAACATTG

>Frog_T2R57--Pseudo

CAGTTCTTCTTGGGGGCTGCAGTAAATGGGATCATTGTGGGGACCTTTTGCCTGGAATGGATAAGGAACCGAAGCCTCCAAGAAGGCGATACCACCCTTATGTGCCTGGGCTTCACCAGACTCTTTTTGCACGGGTCTCTCATTTTGTTCTATTTACCACAGTCCTTTCATGTGATCCATAAACCTGAAGTCGTAACCAGTATTTCTTGGGCTCTCTGTGTTTTTCCATACCTCCAGCCTGTGGTTTGCTTCCTTCCTCTGTGCCATCTACTGTGTGACAGTTGCAAACTATTCCAACTCCTTATTCGTCTATGTGAAGCGCAATATCTCCAGGCTTGGGTCATGGATGATTGTGGCCATTCTGCTGACTGCTCTGACTTTCAGTCTCCTGGACTGGTATATTGCGTATGGTTTTGGCCTGTCTCCGAGCAATTCTACAGGAATTATACAGCAGCGGAACAGCACTGGGGCCGATGGGAATGCAGCCACCACCCTTGGGGTTCAATTTGTTATATACTTTATGGCGGTCGCACTGCCATTCACATTATACTGCCTGGCGGCTTTGCTTTTGCTGAACTCTCTATGGAGACACGTCCGACAGATGAGAATCAGCGGAACAAGTTTCCGGAGTCGGAGTGTGGACGCTCACGTGGGAGTATTAATGAAACTGGCCGTGTCCCTGTTACTGTATGCTGTGTATTACGTAACGGATACTCTTGGCTTATATGGGCAGCTTGAAACACCTTGGCTCTTATTCTGCTTTGTTATTTGCTGTTTCTACCCCACTGCCCATTCTCTGGTCTTGATTTATTCCAACAGTCGGCTGAGGAAGGCCTGCGTAGCGACGATCCGTGGGGCCATGAGTTGGGAAAAGAGAGAAACTCAAGACAACCAAACCCAGAG

>Frog_T2R58--Pseudo

ATGCTCATTGTAACGTGGCCATGTGGGACCATCCTGAACTCATCCATTATAGCTGTCTATCTCAGCGACTGGAAGAAGGGAGTGAAACTTGGGGAGTGCGATCAGATCACTCTCAGTATGGGGTGCACCAACCTGCTCTTGCAGTGCTTCATAACACTTGGAGTGGCCTTCAACTCATATGGACTCCACCTGCCGTTTGCCGTCTCTCTTGCTATCGGTGCTGTGCTCTTGTTTTCCATTTATTTCTCATTTTGGCTCACAGCCGGTCTCTCCGTCTGCTACTACCTGAGACTGGCCAATCCGTTACCTAAATTCTTTCTTCAGTTAAAAAGGAGACTCTCTCGCATTGTTACCCCACTTCTTCTGTGGTCAGTGGCAATTTCATTTACTGTTACTTTTTCAAATATCATATTGTACACAGGAACTAACCAGAACCTAACAATCCTTTATCATAATAATAAGTCTAATGTAAATGATGATTACAGCATCCCTGCTATTACATTTGGTATCAGCCTGCCCTCCATTATTACCTCTATTTGTATTCTACTTTCCCTGATATCGCTCCTGAGGCACATCCGGAGAATGAAGCAGAACCCTCAGGTCGGGAGTCCTCAGCTGAAGAACCATATAAGAGCTTGCAGGACAATGTTTCTACTCATGACTCTGAATTTTCTTTTTTTCCTGATCATTTTTAGTTTCAATGGTACCGCCATATAGTGGGGACACAATATGGCAAACTGTGATGTTTTCATGTGTCATGTTAAACCCCTCAGGTCAAGCCGTTGTTCTGATCTTTGGAAATTCCAAGTTACTCAGTGCCTGGTCTATAACCTTAGTTCCTCA

>Frog_T2R59--Pseudo

ATGCTCACTAACGTGTTCATCTTGTCTGTTAATTTTCATTCCTGGATAAAAGGCCAAAGCTTGAACCCAAGTGACCTCTTACTTGTTACCCTGGCTTTCTCCAACTTGGTCCTTCCAGTCACAGCCGGTGTCTGCACCATATACTTTGGTTTCATCAGTTGTGGGGTCATTAATGATTATCAGTTCTTTGTGCAAACTTCTATCATGGTGTATGTGCTCTTGTCCAATTCCTGGCTCAGCGCCTGCCTGTGTTTCTTCTGCTTTGTGAAAGTCACTAACTTTAAGCCTGGTTACCTGGCACGGCTCAAGTCCAAGATAAACACCCTGGTGCCAAGGCTGATACTGGGGGCTCAAGCGTTCTCCATCTTGAACTCTATATTTTACATGGTGAAAATTCACCAATACCTTTTTTGACAAATGAGACTTCAAGTACTACTAAGGACAGTTTAGGCACTTTTTACAATGCATTTTTCTTGCTCAATTGTAATATTCCATTCTTCATTATAGCGGGAACCACCAGCCTTATCATTGCCTCGCTCTACAAGCACACTCGTCGTATGCAACGGAACATGGGAGAGTTTGGTGGCCCCGGGCTGAAAATTCATCGAAGAGCAGCACGTACAATGGCATCTTTTCTTATTATTTATCTTTGTTATCATGGTTTATCACAGGGGAGTATCATATTTCTAAGCAGGCAGTTGTTGTACTGGGTTAACTTTGCGTTGGGTTGTGCTTTCTCCCTGACTCAGTCCATCGTTCTAATCACGGGGAACTCCAGGCTCGGGCAGACCTGTAGGAACATACTTCACAGGTGTATGAAAATATTAAGCAGAGAGGAAAATATATCCACTGTGGGCA

>Frog_T2R60--Pseudo

ATGCTCACTAACGTGTTCATCTTGTCTGTTAATTTTCATTCCTGGATAAAAGGCCAAAGCTTGAACCCAAGTGACCTCTTACTTGTTACCCTGGCTTTCTCCAACTTGGTCCTTCCAGTCACAGCCGGTGTCTGCACCATATACTTTGGTTTCATCAGTTGTGGGGTCATTAATGATTATCAGTTCTTTGTGCAAACTTCTATCATGGTGTATGTGCTCTTGTCCAATTCCTGGCTCAGCGCCTGCCTGTGTTTCTTCTGCTTTGTGAAAGTCACTAACTTTAAGCCTGGTTACCTGGCACGGCTCAAGTCCAAGATAAACACCCTGGTGCCAAGGCTGATACTGGGGGCTCAAGCGTTCTCCATCTTGAACTCTATATTTTACATGGTGAAAATTCACCAATACCTTTTTTGACAAATGAGACTTCAAGTACTACTAAGGACAGTTTAGGCACTTTTTACAATGCATTTTTCTTGCTCAATTGTAATATTCCATTCTTCATTATAGCGGGAACCACCAGCCTTATCATTGCCTCGCTCTACAAGCACACTCGTCGTATGCAACGGAACATGGGAGAGTTTGGTGGCCCCGGGCTGAAAATTCATCGAAGAGCAGCACGTACAATGGCATCTTTTCTTATTATTTATCTTTGTTATCATGGTTTATCACAGGGGAGTATCATATTTCTAAGCAGGCAGTTGTTGTACTGGGTTAACTTTGCGTTGG

>Frog_T2R61--Pseudo

GAGATAATAGTTGCCCCGGGCTTATCACGTGCTTATGTTTCTCTGATCTGTTACTTAATTATTAGTTTTGTTTGGTCCCAACTTTACGCAGACCCATATGTCAAAGTTTTTATCATTACTCTGCTCATGTATGGGGTTTCTTCCTGTGCCTAGAATATGACCTGTCTATGTGTCTTCTACTTTGTGAAGATAACCAACCTCTGGGATTTTTACTTGCTTCAAGATAAAGATCAACATCATTTCTCCATGGTTCCTACTGGTTCCAGAAGTAGTGTCTTTGGGCTGAAGTTTCCTTACCTTATTGCCTTCTGTTAATAGCCAAGAATCATCAAACACCACATTGTTGTACTCAGTCAACTCAACATCAGGAGCAAGTGCCACCATCACTGGATTTATGAAGGTTACCCGTGTTTGCTGTTTTTGTTCCTTTGCTAATTATATTTGTTACTACTATCCTACTTTTGGATCACTTTATATGCACAGTAGGAGAATGGAGAAGACAGGAAGATCTAGACATTTGACTCC

>Frog_T2R62--Pseudo

ATGGCTTCCCCGCTGGAGATATTTCTATTAACTCTTATCTGGATTGTGACTGCGGTGGGAATTCTGCTCAATGCTTTCATCGTAGCCATGCCCGTCATTTGGTGGGTGAGGTATAACAAAGTAGAAATGATAGAGTTCCTGCTGGCCAGCGTGGGGATGTCCAGAGTGGTCCTACTGATATTATGGGATGTCGTATACCTTTGGTTCCCCAGCAATTCAGTCCTATTTGCAGTGGTTTCCATGTTCCTCAGCTTCTGGAGCCTCTGGGTTGCTACAATCCTGTGCGTCTTCTACAGCGTGAAGATCAGTAGTTGTCACCATCCGTTCTTCATGTTCCTCAAGCTGAACGTCTCCAAGATGCTGCTGGGCTTGTTCTTGGTGTCCCTGGCAAGTTCTCTCCTATTCAGCCTCCCCTTTAAATGGCTGGTGTACAGTACCTCCATCAAGGACAAGGAGGGACCATCCTGAAGGTCAATAATATCAACCAGTTCTTCCTCATCCTGACCGGATCCTCGGTGCCGCTCCTTATCTTCTGTGTGGCAGTTGCTATTCTGATCCGATCCCTGTGGAGCCACACTAGGAACATGGCCGGGGGCAACGTGGATTTTGGGAACCCCCAGATTCAGGCTCATCTGAGTGCAGTTAAAAGCATGGTCTCTTTCCTGATTCTGTTTACCATTTATTTTGCCCTATTTGTTGTATCATGTTTACCCCCATTGTTGGATGATACTGTGTTGCAATTGGTCTTTAACATTATTTCTAATGCCTACCCGCTCCTACACTCTCTCATCTTGGTAATGTACAGCAGGAAGCTCAGAGAGGCTCTCTACTGGTGTCTCCATTGTACGTGTAGAGTGCCTTCCATGGCGAGAGGTTCAG

>Frog_T2R63--Pseudo

ATGCTGGCAGCGTACACAGTGATTGTCACAGTGATACTGATCGTAACATGGTTGTGTGGGACCATCCTCAACTCATCCATTATAGCTGTCTATCTCAGCGACTGGAAGAAGGGAGTTAAATGTGGGGAGTGCGATCAGATCACCCTCAGCATGGGGTGCAACTACCTACTCATGCAATGCTTCATAATATTTTTTTGGGCATTCAGGTTCTATGGACTGGACCTTCCATTTGCAGAAAAACTCTCTTTTGCTATCAGTACTGTGTTCTGGTTTTCTGTTTTTCTTTCATTTTGGCTCACAGCCAGTCTCTCCATCTGCTACTGTCTCAGACTGGTCAATCTATCGTCCGCAGTTTTTAATCAGATAAAAGGAAGACTCTCTCGCATTTTTATCCCTCTTCTTCTGTGGTCAGTGGCAATTTCATTTATTTTTCCAGTTACCCGGATAGTTGACATAAAAATTGACCAGAACGGGACTTTTATTTATCATGAAAATATCTCTAATGTGGGTATTCTGATCTCTGCTGTTGCATTTAATGTCTGCCTGCCCTTCATTATTACCTCCATTTGTATATTTCTTTCCCTGATATCGCTCCTGAGGCACATCTGGAGGATGAAGCAGAACACTCAGTTTGGGAGTCCTCAGCTGAAGAACCTTATAAAAGCTTGCAGGACAATGTTCCTACTCATGGCTCTGAATTTGCTTTTCTTCCTGATAATTTGCAGTTCTATGCTGATATCAGATAGAATGGGAACTGTATGGGGCAAGGTGGTCTTGTTAGGGAACATGTTGAACCCCTCATGTCAAGCCATTGTTCTGATCTTTGGGAATTCCAAGTTACTCGGTGCCTGGATAAAGACCTTGTTTCCTCAGTGATGGAACAGTGTACCAAAATTATAGTACCATACATTTATTGTGATAAGAAACATTATATAG

>Horse_T2R20--Pseudo

ATGTTGACTCTGCCTTCCATCATAATTGTGTCCTACAAAGTCAAGAATGCATTTCTATTACTTTCATTCCTGGAGTTTGCTCCGGGAATCCTGGCCAATGCCTTTATTTTCTTGGTGAATTTTTGGTGAGGAAGCAGCCACTAAGCAACTGTGATCTTATCCTGCTATGTCTCAACCTCTCTTGGCTTTTCCTGCATGGGCTCTGTTTCTGGGTGCCATACAGCTTACCCATTTCCAGCAGATGGAAGACCCACTGAGCCTCAGCTACCAAACTATCATCATGCTCTGAATGATCACAAATCAAGCTGGCATCTGGCTTGCCACTTGCCTCAGTCTCCTCTACTGCTCCAAGATTGTCCATTTCTCTCACACCTTCCTGCTCTGCTTGGCAAGCTGCATCTCCAGGGAGATCCCCAAGATGCTCCTGGGTGCTATTCTTTTCTCCTGTGTCTGCACTGTCCTCTGTTTGCGGGACTTTTTCAGTACATCTCACTCCACAGTCTCAACTATGCTATTCATGAATAACAATTCACAACTCAATTTGCAAATTGCAAAACTCAATTTCTTTCATTCCTTCCTCTTCCACAGCCTGGCGTCCATCCCACCTTTCTTATTTTTTTCTGTTTCTTCTGGTATGTTAATTATCTTCCTGGGGAGGCACATGAGGACAATGAGGGCCAAAACTAGAGAGCCTTGCCATCCCAGCCTGGAGGCCCAGATCAAAGCACTCAAATATCTCTCATCTCCTTTCTCTGCCTCTATGTGGTGTCGTTCTGCGCTGTCCTCATCTCAATACCTCTACTGATGCTGTGACATAGCAAGATTGTCGTAATGGTCTCTGCATGGATAATGGCAGTTTGTCCCTCGGGACATGCAGCCATCCTGATCTCAGGCAATGTTAAGCTGAGGGGAGCTGTGGAGACCATTCCACTCTAGGCTCAGAGCAGCCTAAAGGTAAGGGCAGACTACAAGGCAGATCCCAGGACACCA

>Horse_T2R21--Pseudo

CTGATCATGGTAACAGGAGAACTCATTTCAGGAAAGCTTGGAAATCAGTTCATTGGACTAGTAATCTGCATTGAATGAGTCAAGAATGGGAAGATCTCAACAAGTGATTTCATCCTTACCAGATGATTTCATCCTTACCAGGGTGGCCATGGGCAGAATCATTCAACTGTGGGTAACACTATTTGATTCATTTATAATGTGGCTATCTCCACATCTGTATGCCACCAGTAAACCAGCAAAAGTGGTTACTATTCTTTGGGCACTAATCGATTACTTAACTACCTGGTTTGCCATCTGCCTAAGCGTTTTCTGCTTCCTTGAGATAGCCAATTTCTCCCACTTCTTTTTCATCTGGCTGAAGTGGAGAGTGAACAGAGTGGTTCTTGTGCTTTTCCTGGTGTCTTTGTCTGTTAACCTCTTAATGTAGAATGCTCTTAAACAGTTGCGAATGAATACCTATAGAGTACATGAAAGAAGCATGACTTTGCATTTAGATGTAAATAAAATTTTCTATCTTAAAAGCCGTCTTCTTCTTAGCTTGACCTATGTTATCACCTTTCTTCTGTCCCTGACCTCTTTGCTCCTTTTGTTTCTGTACTTAACGAAACACAGCAAGAATTTGCAGCTCAGCCTGTAGAAAGGAGGGACTCCAGTACAGAGGCCCAGAAAAGACCATGGAAGTGAGGACAACCTTCTTCCTCTTCTTCATCATTTACTTCATTTCCACTCTAATAGCAATTTGGATTTTCCTTAAGGTCCAGAGGTATCAGTTTGTCATCGTGATTTCATTCATCTTTCCCTCAGTCCACTCATTAATTATAATTTTGG

>Horse_T2R22--Pseudo

ATGTCAGTTGGAATTAAGGTCTCCTTTCTGGTCATGGCAACAGGAGAACTCATCTTACGAATGCTGGGAAATGGGTTCATTGGACTGGTAAACTGCATCGAATGGGTCAAGAATGGGAAAATCTCATCAGCTGATTTCATCCTTACCAGCTTGGTATGGCCAGAATCATTCAACTGTGGATAACACTATTTGGTTCATTTAATGTAGGGCTATTTCCACATCTGAATGCCACTAGTAAATTAGCAAAAGCGATTACTATTCTTTGGGCACTAACTAATCACTTGCTTGCCACCTGCCTAAGCATTTTCTGCTTCCTTAAGATTGCCGGTTTCTCCCACTTCTTGTTCATCTGGCTGAAGTGGAGAGTGAACAGAGTGGGTCTTGTACTTTTCCTGAGGTCTTTCTTCTTATCTCCTGCTAGTCTCTTAATGCAGGATGCTCTTATTGAGTTGTGGATGAATACGTATGGAGTACATGAAAGAAACATGACTTTGCATTTACACGTAAATAAAATGTTCTATCTTAAAAGCCTTCTTCTTAGTTGACCTATGTTATCCCCTTTCTTCTGTCCCTGACCTTTTTGCTCCTTTTATTTCTGTCTTTGGTGATACACACCAGGAATTTGTAGCTCAACCTGAAGGGCATGAGGGACTCCAGCACAAAGGCTCTTAAAGGGTCATGAAGATGGTGACAACCTTCTTCCTCCTCTTCATCATTTACTTTATTTCCACTCTAATA

>Horse_T2R23--Pseudo

GTAATGAGGGCTTCCTTTCTGCTCATGGCAACAGGAGAACTCATCTTAGGAATGCTGGGAAATGGGTTCATTGGGCTGGTAACCCGCATCAAATGGATCAAGAATGGGAAGAGCTCATCAGCTGATTTCATCCTACCAGCTTGGCTATGGCCAGAGTCATCCAACAGTAGGTAACACTATTTGGTTCATTGAATGTGGAGCTATCTCCACATCTGTATGCCACCAGGAAACTAGCAAAAGCAGTTACTATCCTTTGAGCACTAACTGATCACTTAACTACCTGGTTTACAACCTGCCTAAGCATCTTCTGCTTCCTTAAGATCGCCAATTTCTCCCACTTCTTTTTCATCTGGCTGAAGTGGAGAGAGAATAGAATGGTTCTTGTGCTTTTCCTGGGGT

>Horse_T2R24--Pseudo

TTCATCTTCATTTTAATAAATATAGAAATAATAACTGGTAGTTTGGCGAATGGATTCATAGCGCTGGTGAGCTGCATTGACTGACTCAAGAAACAAAAGATCTCCTCAGCAGATCGAATCATCACTGGTTTGGCGATCTCCAGAATTTGTCTGATTTTGGTAATAACGGTGACCTGGTTTACAAAGGAGTTTTATCCATCTTCATATACAAATAGAAAGAAAGTTACGCTTATTAGTATTGCTGGGACCATGGGCAATCATTTTAGCTTCTGGCTTGCCACAGGCCTCAGCTTCTTTTGTTTTCTCAAGATAGCCAATTCTTCAAATTCTGTTTTTCTTTACCTAAAGCATAGAGTATATCCTTAAATTTTTTTCTCCTATTAATCTTCTCCCTATGGAAACATGTCAAGAATATGAAGCGCAATACAACAGGATTCATAGATCCCAGCGTCAAGGTCCATATAAGAGCCATGAAAACTGTGATCGTTCCTCTTACTGTATACTATTTACTTCCCGTCGCTTATCATAACAATTTTCCGTTCTGAGGTGATACAGAACAAATCAACCATTGTGCTTGGTCAGGCTCTTGCACATCTTTATCCTTCAGGCCACTCACTTATCCTAATTCTGGGAAATGGTAAACTAAGAAAGGCTTCACTTTCTGTGCTGTGGCAGCTA

>Horse_T2R25--Pseudo

GGAATGAAGGTCTCCTTTCTTGTCATGGCAACAGGAGAACTCATCTTAGGAATACTGGGAAATGGGTTCATTGGACTGGTAAACTGCATCGAATGGGTCAAGAATGGGAAGGTCTCATCAGCTGATTTCATCCTTACCAGCTTGGTATGGCCAGAATCACTCAACCGTGGGTGACACTATCTGATTCATTTATAATGGCACTGTCTTCACATCTGTATGCCACCAGGAAACCAATAAAAGTGGTTACTATTCTTTGGGCACTAACTGATCACTGAACTACCTGGTTTGCCACCTGCCTAAGCATCTTCTGCTTCCTTAAGATGGCCAGTTTCGCCCACTTGTTTTTCATCTAGCTGTAGTGGAGAGTGAACAAAGTGGTTCTTATGCTTTTCCTGGGAACTTTCTTCTTGTTGTCTGTTAACCTCTTAATGCAGGATGCTCTTAGGAGTTGTGGATGAATATCTGTAGAGTACACGAAAGAAACATGACTTTGCATTTAGATGTAAATAAAATGCTCTATCTTAAAAGCTTTCTTCTTAGTTTGACCTATGTTATCCCCTTTCTTCTGTCCCTGACTTCTTTGCTCCTTTTGCTCCTGTCCTTGATGAGACACACCAAGAATTTGCAGGTCAACTTGATGGGCATGAGGGACTCCAGCACAGAGGCCATAAAAGGGCCATAAAAATGGTGAAAACCTTATTCCCCCTCTTGATAATTTGCTTTATTTCCACTCTAATAGCAAGTTGGATCTTCCTTAAGGTACAGAGGTATCATGTTATGATGTTTGTCATGGTGATTTCAACCATCTTTCCCTCAGGCCACTCATTAATTATAATTTTGGGAAACAGT

>Horse_T2R26--Pseudo

GGACAACATGTTTTTGGTTTCTTAATTATGGAATTCATACTTGGTACTTGGGGAAATGGATCTATTGGAATAATGAGCTATATTGTCTGGGTAAGACACAGGAAAAACAGCTTCGTTAGCTTCATCCTACTGAGCTTATCCATAAGCAGAATGTACTGGATCATCATACTGATTGATTCACTTCTGATGGTGCTGTCTGCAGATATACATAGCCTGGCCAATTGGAAAAATTAATATTCTCTTTTATGTAATGATCAACTACTTAAATGTCCGGTTTGAAACCTGCTTCAGCATCTTCTTTTCCCTGAAGATCGTTACTTTTTTTTCCCCATCCTCTTTTTCTTTGATTAAAGTGGAGAACTAACAGATAGTTATTATTATTTTCCTGAGGTCCCTGCTCATTTTTCTTTTCAGCATCATGGTGGTAGTCCACATAATTCCTGATATTCTAGATCTAGAAACAAACAAGACTTGGAAGATTAATACATATCAAACAAAACCCACCATGTTCTCAGTGCTCATCAGTGTTGAGATCACCCTGCCCATAACTCTTCCCACAGCATTGGTTCTTCCATTATTTCTTTCTCTTTGGAGGCATAGCTAGAGGATGGAGTTCAGTATCACAGGGTCCCAAGATCCCAGAAGTGAAGCTCATGGAAAAGCCATGAGAACGGTGAATTCTTTCTTCTTTCTCTTTCTGCTTTATATTGTGTTGATTCTGATAACAGTTACATCCATTATTGTCTCCTAACAAGCTGATATTGACGTTTGGTAAATTAATAGCATCTGCCCATCCTTTAGGCCATTCATTTATCCTAATTTTGAGAAACAGCAAGCTGAAGCAAGCTGCTGTTAGGATTTGGGGGCAGCTGAAGTGCTTTC

>Horse_T2R27--Pseudo

ATTGAAAGCACTTTTCTGATAGTGGCAATAGGAGAATTCATAACTGGAATGTTGGGGAATGCGTGCATTGTACTGGTTAACTGCATTGACTAGGTGAAGAGTCAGCAGCTCTCATTAGCGGACTGCATCCTCACCAGCCTGGCTATCTCCAGAACCAGTCATCTTTGGGTAATACTACTTGGTTAATTTGTCATATTGCCACATCTATATGCCAATGATAACTAGCAAACTTGGTTGGTATATTTTGGACACTGACCAATCACCTAGCTACCTGGTTTGCCGTCTATCTAAGCTTTTTCTACTTCTTTAAAATAGCCAGTTTCTCTCACCCCTGCTTCGCCTGGCTGAGGTGGAGAATTAGCATAGTGTTACTTGTGCTTCCACTGGAGTCTTTGTTCTGTTTTTCAACCTTGAACTGAAAGGTACAATTAATGGTTTCTCAATTTAGTCCTCAGATGTAAGTGAACTTCTGTATCTTAACAGCTCGTTTGTTGTCAGTTTGATCTACTTAATCCCCCTTCTTCTGTCCCTGAACTCACTGCTCCTTTTATTTCTCTCCTTGATGAGACATACCAGGAATTTGCCGCTGGACTCTAGCTCTTAGGGACCTCAGCTCAGAGACCCATAAAAGGGCCACGAAAATGGTGATGTCTTTCCTCCTCCTCCTCATGGTTCACTTTTCTTCCACTCTATTAACAGGGTGGGTTTTCCTTATACTGAAGAAACATTGGGCCAATTTGGTTAATGTCAACTCTTTTTCCTTCAGGCCACACATTTATCCTAATTTTAGGAAACAGCAAGCTGAGGCAAAATGCCTTAGGACTATTGTGGCATCTTAATTGTCACTGG

>Horse_T2R28--Pseudo

ATGCTGAGATCAGTGGAGAGTATCTTCATGACTGTGGCAGTGTTGGAAATTATAATGGGAATTTTAAGAAATGGATTGTTTAGACTGGCAAATTTAATTGATTGGGTTAAGAACCAGAAGATCTATTTGACTGATTTCATCCCTTCAAACTTAGCCATATCTAGAACATTTTTGTTATGGGTGTTCCTATTAGACGTATTTATAATGCTTATCTATCCAGATATAGATGACATTAGGAACCTAATTAAAATTATTGACATCCTCATTAGTTTCAAAATATTCGAGTATCTTGTTTGCCTCGTGTTTCAGTATTTTTTATTTGCTTAAAGTAGTATTTTTCTACCATGCCATTTCGTT

>Horse_T2R29--Pseudo

ATGATAACTTTACTACCAAGCATTTTTTCTGTCCTAATAACGACAGAATTTGTTCTGGGAAATTTCGCCAATGGCTTCATAGCACTGGTGAACTGCATTGACTGGGTCAAGAGACAAAAGATGTCCTCAGCTGATCAAATTCTCACAGCTCTGGCGGTCTCCAGAATTGGTTTGCTCTGGGTAATCTTTTTTGCTCTGGGTAATCTTAATAAATTGGTATATGACTGTGCTTCCTTCAGTTTTTCATAGTTTAGAAGTAAGAATTATTGTTTTTATTGCCTGGACAGTAAGCAACCATCTTAACATCTGGCTTGCTACTAGCCTCAGCATATTTTTTTACTTGCTAAAGATAGCTAATTTCTCTAGCCTTATATTTCTTTACCTAAAGTGGAGAGTTAAAAGTGTACTTCTCGTAATACTGTTGGGCGCTTCGGTCTTTTGGGTTTCTCATCTTGCAGTGCTATGGGTAAATAATAATGTGCAGACTAATGAATTTGAAGGAAACATCACTCAGAAGACCAAATTGAGGGATATTGTAGCTCTTTCGAATTTGACTCTATTCACGCTAGTAAACTTCATACACTTTTCTATGTCTCTGACATGTTTTCTGCTGTTAATCATTTCCCTGTGGAAACATCTCAAGAAGATTCAGCTTAATGGCAAAGGATCCCATGATCCCAGCACCAAGGTCCACATAAGAGCCATGCAAACTGTGGTCTCCTTTCTCTTGCTATATGCTGTTTACTTCTTGGCTCTAGTTATCTTAGTTTGGAGTTCTAATAGGCTGGAGAGTCAACTGCTTGTCATGCTTTGCCGGGCTTTTGAAATACTCTATCCTTCAAGCCATTCATTTATTCTGATTTGGGGAAACAAGAAGCTAAGACAGGCCTTGCAAAATATACGAAATATTATAAG

>Horse_T2R30--Pseudo

CTTTTTGTTGTAGCAACAGGAGAACTCATTTTAGAAATGCAGGGAAATGGGTTCATTGGACTGGTGAACTCATCAAATGCATCAACAATGGGAAGATCCTATCAGCTGATTTCAACCTTACCAGCATGCTATGACCAGAATCATTCAACTGTGGGTAACAGCATTTGGTTTATTTAATGTGAGGGCTCTTTCCACATCTGTAAGCCACCAGTAAATCAGCAAAAGCAGTTACTATCCTTTGGGCACTAACCAATCACTTAACTACCTGGTTTGCCACCTGCCTAAGCATTTTCTGCTTCCTTAAGATAACCAGTTTCTCCCACTTGTTCATCTGGCTGAAGTGGAGAGTGAACAGAATGTTTCTTCTGCTTTTTCTGGGGTCTTCCTTCTTGATGTCTGTTAACCTCTTAATGGAAGATGCTCTTAATGAGTTGTTGATTAGTACCTATTGCATATATGAAAGGAACATGATTTCACTTTTATTTAGATGTAAATAAATTTTCTATATCAAAAGACTTCTTCTTCTTAGCTTGACCTATGTCCTTTCTTCTGTCACTGACCTCTTTGCTGCTTTGCTGGTGAGACACACCAAGAATTTTCAGCTCAATCAGATGGGCCGGAGGGACTCCAGCACAGCAACCCATAAAAGGGCCATGAAAATGTTGACAACCTTCTTTCTCCTCTTCAGCATTTGCTTTATTTTCACTCTAATAACAAGTAGGATCTTCCTTAAGGTACAGAGGTATCAGGTTATGGTGTTTATCATGGTGATTTCACCCTTCTTTCCCTCAGGCCACTCATTCATTATAATTTTTGGAAACAGTGAGC

>Horse_T2R31--Pseudo

GTGATTGATAGGAGAGCCATCACCTTGGCTATCATTTTATTCCTTTTGTGCCTGGTGGCAGAGATGGGCAATGGCTTCATCACTGTGGCACTGGGCATGGAGTGGTTACTATGGAGAACATTGTCACCTTGTGATGTTATTGGTCAGCCTGGGAGTCTCTTGCTTCTATGTGCAGTGGGTGGTGATGAGTAAGAACATTTACGCTAGAATTGTGTGTCCACTGGTCCTTCCATACAACCCTGTACTATAGTTTCTAGCCTTATAGTGGGACTTCTTGAACACTACCACCTTTTGGTTCTCTACCTGGCTCAGTGTCCTCTATTGCAGGAAAATTTCAACCTTCACTCACCCTGTCTTCCTCTGGCTAAAACAGAAGGTGTCTGGGTTGGTTCCCTTGATGCTGCTCAGCTCCATGGGGTTCTCTAGCTTGAGCACCATCCTATTTTTCACAGGCAACCAGAGCCAATATCAGAACTTTTTAAGGAGAGGTCTGCAATATTGGAATATCATCAGGAATCCTATAAGGAGATCACATGAGAAATTCTACTTCTTTTGTTTAAAATTCGTTACTTAGGCAGTTCCTGCTGTTGTCTCCCTCACTGGTAGGATTTTAGCTCATCATGTCTCTCGGAAGACACACCAATAAGCCCTTTCTGTCTGTCTCGGGCTTTTGCCATCCTAGTGCCCAGGCACACATCAGGACTTTCCTGGCTCTCATCTCCTTTGCTATCTTCTTCAATTCCTATTTTCTGTCACTGGTGCTCAGTGCTGCAGTTATTTTTCCATCTTAGGAATTTAGGTACTGGGTGTGGCAGACAGTGATTTATCGGTGCACAGTAGTCCACCCCATCAGTCTACTCTTGAGCAACACCAGGCTGAGAGTGGTGCCGGAGAGGAGCTGCTCCTCAAGGC

>Horse_T2R32--Pseudo

ATGGCAAGCACACTGAAGAATATATTTATGATAATTTCTGTTGGAGCATTCACAATGGGGATTTTGGGAAACGGATTCATTGTACTGATTAACTGTATTGACTGGATCAGGAGCTGGAAGTTCTCCCTGATTGACTTTATTCTCACCTGCTTGGCTATTTCCAGAATATTTCTGCTGTGCATAATAATTTTAGGTATAGGCTTACATTTCATCAATGAGGAAATATGGTACAATGATAATAATCTACTGAGAAGTTTGAGAATTCTCTGGACAGGATCCGATTATTTCTGCATGACCTGTACCACCTGCCTCTGTGTCTTTTATTTCCTCAAGATAGCCAACTTTTCTAATCCCATTTTCCTCTGGATGAAGTGGAGAATTCACAAGGTGCTTCTCATTATTGTGCTCGGGGCATCCGTCTCTTTCTGCTTGTGTATTTTTTTAAGGATACAGAAACTAGGAGCCTGATCAAAAACCAGGTAAACACGGAACAAAATTTGACATGGAATATTGCAGTGAGAAAATATAATTTATCAACTTCTCATATGCTTATTAACATAATGTTCATCATCCCCTTTTTAGTGTCACTGGCCTCCTTACTCCTTTTAATTCTCTCTTTATGGAGCCACACCAGGCAGATGAAGGGCACAGGTTCTAGGGATCCTAGCACAGAGGCCCACGTGAGGGCCACGAAGTCTATGATTTCATTCCTACTCTTCTTCTTCTTGTACTATTTGAGTAATATTATGCTAAAGTCAATCTATGCCAATCTAGACAGTTTTACAGTAAAGATTTTTGCTAATGTGCTAGTGTTTTTCTTTCTATCTGGCCATCCATTTCTTCTGATTTTGTGGAATAGCAAATTGAAAAAGGCTTCTCTCAGTGTCCTGAGGAAGCTGAAGTGTTGCATGAATCTAAGGAAAC

>Horse_T2R33--Pseudo

ATGGCAAGCACATTGAAGAATATATTTAAGATCCTTTATGCTGCAGCATTCACAATGGGGTTTTGGGAAACGGATTCATTGTACTGATTAACTGTATTGACTGGATCAGGAGCTGGAAGTTCTCCCTGATTGACTTTATTCTCACCTGCTTGGCTATTTCCAGAATATTTCTGCTGTGCATAACAATTTTAGGTATCGGCTTAGATGTAAACTCTGAGAAAATATTGTACACTAATAATAATTGACTGATAAGTTTGGAAACCCTCCGGGTAGGATCCAATTATTTCTGCATGACCTGTACCACCTGCCTCAGTTTCTTCTGTTTCCTCAAGATAGCCAACTTTTCTAATCCCATTTTCCTCTGGATGACATGGAGAATTCATAAGGTGATTCTCAGTGCTGTGCTGGGGGAAGCCCACTCTTTCTGCTTGTGCCTTTTTTTAAGGATACAGTACTTAAGAGCCTGATCATAAACCAGGTAAATACTGAAAGAAATTTGATATGGAACTTCACAGTGAGAAAATATTCATTAACTTCTCAAATGCACTTTGACATAACGTTCATCACCCCCTTTGTAGTGTCACTGGCCTCCTTACTTCCTTTAATCCTCTCCTTATGGAGCCATACTAAGCGGATGAAGAGTACAGGTTCTAGGGATCCCAGGACAGAGGCCCATGTGAGGGCCATGAAGTGTATGATTTCATTCCTACTCTTCTTCTTGTACTATTTGAACCATCTGATAATAAATCCAGCCTGTGCCCTTCTAGACACTTTTGTGGCAAAGACTTATGCTAATGTGCTAGTATTTTTTGATCCATCTGGCCATCCATTTCTTCTGATTTTGTGGAACAGCAAGTTGAAACAGGCTTCTCTCAGTGTCCTGGAGAAACTAAAGTGTTGCATGAATCTAAGGAAACCTACATT

>Horse_T2R34--Pseudo

ATAACTTTACTACCAATCATTTTTTCCGTCCTGAAAATGACAGAATTTATTCTAGGAAATTTTGCCAATGGCTTCATAGCACTGGTGAACTGCATTTACGGGGTCAAGAGATGAAAGATGTCCTCAGCTGATCAAATTCTCATGGCTCTTTCCATCTCCAGAACTGGTTTGCTGTGGCTCATGTTAATAAATTGGCATGCAACTGTACTTACTCCAGTTTTATATAGTTTAGAAGTAAGAATGATTGTTCGTATTGCCTGGGCAGTGAGCAACCATTTTAGCATGTGGCTTGCTACTAGCCTCAGCATATTTTATTTGCTAAAGATAGCTAATTTCTCAAGCTGTATATTTCTTTACTTAAAGTGGAGAGTTAAAAGTGTACTTCTCGGAATACTGTTGGGGACATCGGTCTTTTTGGTTTCTTATCTTCCAGTGCTATGCATAGATGAGAATATACAGACTAATGAGTATGAAGGAAAGATCACTTGGAAGACCAAATTGAGGCACACGGTATACCTTTCAAATATGACTCTATTCATGCTAATAAACTTTGTACCCTTTGTTATGTCCTTGACATGTTTTCTGCTGTTAATTATTTCCCTATGGAAACATCTCAGGAAGATGCAGCTCAATGACAAAGGATCCCAAGATCCCAGCACCAAGGTCCATCTAAGAGCCATGCAAACTGTGGTCTCCTTTCTCTTCCTATTGTCCTGTTACTTCCTGACTCTAGTTATCTCAGTTTGGAATTCTAATAGGCTCCAGAATGAATGGCTTCTCATGCTTTGCCAGGCTCTTGGAATGTTGCATCCTTCAAGCCATTCGTTTATCCTGATTTGGGGAAACAAGAAGCTAAGACAGGCCTTGAAAAATATA

>Horse_T2R35--Pseudo

TCTGTCCTAATAACGACAGAATTTATTCTGGGAAATTTTGTCAAGGCTTCATAGCACTGGTGAACTGCATTGACTGGGTCAAGAGACAAAAGATGTCCTCAGCTGATCAAATATTCACACTCTGGTGGTCTCCAGAATTGGTTTGCTCTGGGTACTATCAATCACTTGGTATACAACTGTGCTCCTTCAAGTTTTATATAGTTTAGAAGTAAAAATTATTGCTTGTATTGACTGGACAGTGAGCAACCATTTTGATGTCTGGCTTGCTACTAGTCCCAGCATACATTAGTCCCAGCATACATTTTTGTTCAAGATAGATAATTTCTCTAGCTTTGTATTTCTTTACCTAAAGTAGAGAGTTAAAAGTGTACTTCTCGTTATGTTGTTAGGGACTTTGGTCTTTTTGGTTCCTCATCTTGCAGTGCTATGCATGGATAATAATATGCAGACTAATGAGTATGAAGGAAACATCACTCAGAAGGCCAAATTGAGGGACATTTTATACCTTCCAAATGTCACTCTATTCACACTAATACACTTCATACCCTTTACTATGTCCCTGACATCTTTTCTGCTGTTAATCATTTCCCTGTGGAAACATCTCAAGAACATGCAGCTCAATGGCAAAAGATTCCAAGATCCCAGCACCAAGGTCCACATAAGAGCCATGTAAACTGTGCTCTCCTTTCTCTTTCTATATGCTGGTTACTTCCTGATTCTAGTTATCTCTCAGTTTGGAATTTTCATAGTCTGCAGAACAAACTGGTTTTCATGCTTTGCCAGGCCCTTGGAATGTTGTATCATTTAAGCCAGTCATTTATCCTGATTTGGGGAAACAAGAAGCTAAGAGAGGCCTTGAAAATTATAAGAAAGATG

>Horse_T2R36--Pseudo

GGAAATGGATTGATTGGAATAACGAACTATATACTCTGGGTAAGACACAGGAAAATCAGCTTCATTAACCTCATCCTCATGAGCTTATCCGTAAGCAGAATGTGCTTCTTGATTATGATATTGTTTGATTCGTTTCTGCTGCTGCTGTCTTCAAATCCACATAGCATTAATCAAATTGGAAAACCTAATATTCTGTCATGCATAATGATCAAGCTCTTAAACGTCTGGTTTGATACCTGCCTCAGCATCTTCTGTTTCCTGAAGATAGCTACCTTTTTTCTGTCTTTTTCTTTGATTAAAGTGGAGAATTAACAGAGTCGTTATTATTATTTCTCTGAGACCCTTGCTATTTTTTCTTTTTAGCATCCTACTGATAGTTCATCTAATTCCTGATATTTTACATCTCGCAACAAACAAGACTTGGAGGATTAATATACATGAAACACAATCCATTATTTTCTTCGTGCTCATCAGTGTTGAGATCACCATGCCCATAACTATTTCCGTAGCATCAGTTCTTCTGTTACTTCTCTTTTT

>Horse_T2R37--Pseudo

TTTTTTTCCATCCTAGTAATAGTAGAATTTCTTCTAGGAAATTTTGCCAATGGCTTCATAGCACTAGTAAACTGCATTAACTAGGTCAAGAGACAAAAGATCTCCTCAACTGATCAAATTCTCCTGGCTCTGGAAGTCTCTAGAATTGGTTCACTCTAGGTAATATCAATAAATTGGTATACAGCTGTGTTTAATCCAGCTTTATATAGTTTAGAAGTAAGAACTGATGTTTACATTGCCTGGATAATAGTCAATCATTTTAGCATT

>Horse_T2R38--Pseudo

AAAATTTTCATGATTGTAACAGGTGGAGAATTTATAATAGGAATTTTAGGGAATGGATTTATTGGACTCACAAATTGCATTGCCTGGATTAGAAATCGGAAGTTATGCTTGGTTGACTTCATTCTTACCAGTTTGGCCTTCGCCAGAATCAGTAAATTATGGCTAACAATTGTCAATTTGGTTTTAGTGCTGGTCTATCAGGAAATCCCTGAAACTATGAAAACAAACAACATCCTTACCAGCATCTGGATACTGGTCAACCACTTGACCACTTTGTTGGCTGCTTGTCTCGCTGTCTTTTATTTCCTGAAGATCTCCAGTTTCTCCCATCCTCTTTTCCTTTGGCTGAAACGGAGAATTAACAAGGTAATTTACATGGTTCTGCTGTCATCTTTGCCCTTCCTGTTGATCAACTTTCCTTTGCCAGTTAATATTGATGTCGTCTGGTATCATGTCCAAAAGAAATATGAAAGAAATATGACTGGGTTAGTCAATGTGAGTAAAAGCAAACATTTAAGAGTCATGGTAGTCTTCATTATTGGGTGTGGAGACACACGAAACACAATTTGCTCAACTTCAAGGATTCCAGAGACCCCAGTATGGAGGCCCATGTCAGAGCCATGAAAACTGTATTTCTCTTTCTTGTCCTCTTTGCTGTGTACCAGTTATCTATTTTCATGACATTTTGGGGGTATTTTTCACTACAGAACAAGCTGGTTGTGATGTTTGCTTATATGATAGAAATTCTCTATCCTTCTGGTCACTCATATGTTGTGATTTTTGGAAATAGTCAAATGAGGAAAGCCTTCTTGGGGTTTCTTTGTCACTTGAAGTGTGACTTGAAAG

>Horse_T2R39--Pseudo

ATCCTAATAATGACAGAATTTATTCTGGGAAATTTTGCCAGTGGCTTCATAGCACTGGTGAACTGCACTGGCTGAGTCAAGAGATGAAAGATGTCCTCAGCTGATCAAATTCTCACTGCTCTTGTGGTCTTGAGAATTGGTTTGCTCTGGGTAATATCAATAAATTGGTATACAACTGTACTTACTCCAGTTTTATATTTTAGAAGTAAGAATTATTGTTTATGTTGCCTTCACAGTAAGCAGCCATTTTAGCATGTGGCTTGCTACTAGGCTCAGCATATTTTATTTGCTCAAGATAGCTAATTTCTCTAGCTTGAGAGTTAAAATTTTATTTCTCAGAATACTGCTGGGGAGTTTGGTCTTTTTCTTATCTTGCAGTGCTATTCATAGATGAGAATACACAGACTAATGCACATGAAGGAAACATCACTGGGAAGACCAAATAGAGGGATTTTGTAGGCCTTTCAAATATGACTCTACTCACGCTAGTAAACTTGATACCCTTTACCATGTCCCTGACATCTTTTCTGCTGTTAATCATTTCCCTCAATGGCAAAGGATCCCAAGATGCCAGGACCAAGGTCCACATAAGAGCCATGCAAACTGTGGTCTCCTTTCTCTTGCCATATGCCAGTTACTTCCTGGCTCTAGTTATTTCAGTTTGGAACCTAACAGGTGGCAGAATGAACCAGTTTTTATGCTTTGCCAAGCTCTTAG

>Horse_T2R40--Pseudo

GTGGCAGCAGGAGAACTCATTTTAGGAATGCTGGGAAATGGGTTCATTGGAGTGATAAACACTATCAGATGAGTCAAGAATGGGAAAGTCTCATCAGCTGACTTCAACCTTACTAGCTTGGCTCTGCCTAGAATCATTCAACTGTGGTTAACACTATTTGATTCAGTTATAATGGGGCTCTCTCCACATCTGTATGCCACCAATAAACTAGCAAAAGTGGTTAGTATTCTTTAGGCACTAACTCATCACTTAACTACCTGGTTTACCACCTGCCTAAGCATTTTCTGCTTCCTTAAGATAACCAGTTTCTCCCACTTCTTTTTCATCCTGCTGAAGTGCAGAGTGAACTGAGTGGTTTTTGTGCTTTTCCTGGGATCTTTCTTCTTGTTGTCTGTTACCTCTTAATGCAGGATCAGGAAGCTCTTAATGAGTGGTGGATGAACACCTCTAGAGTATATGAAAGAAACATGACTTTGTATTTAGATGTAAATAAAATTTTCTATCTTACAAACCTTCTTCTTCTTAATTTGACCTATGTTATCACCTTTCTTCTGTCCCTGACCTCTTTGTTGGTTTTATTTCTGTCCTTGGTGAGGCCCACCAAGAATTTATAGCTCAGCCTGATGGGCATGAGGGACTCCAGCACAGAGGCCCATAAAAGACCATGAAAATGGTGACAACCTTCTTCCTCCTGTTCATCATTTACTTTATTTCCACTCTAATAGCAAGCTGGATCTTCCTTAAGGGACATAAGTATTAGGTTATGATGTTTGTCATGGTGATTTCAACCATCTTTCTCTCGGGCCACTCATTTATTGTAATTTTGGGAAATAGCAAGGT

>Horse_T2R41--Pseudo

ATGGCAAGCACATTGAAGAATATATTTGTGATCCTTTACACTGGAGCATTCACAATGGGGATTTTGGGAAATGGATTCATTGTACTGATTAACTGTATTGACTGGATCAGGAGCTGGAAGTTCTCCCTGATTGACTTTATTCTCACCTGCTTGGCTATTTCCAGAATATTTCTGCTGTGCATAATAATTTTAGGTATAGGCTTACATTTCATCAATGAGGAAATATGGTACAATGATAATAATCTACTGAGAAGTTTGAGAATGCTCTGGATAGGATCCAATTATTTCTGCATGACCTGTACCACCTGCCTCAGTGTCTTCTATTTTCTCAAGATAGCCAACTTTTCTAATTCCATTTTCCTCTGGATGAAGTGGAGGATTCACAAGGTGCTTCTCATTATTGTACTAGGGGCAGCCATTTCTTTCTGCTTGTGCCTTTTTTTTTAAGGAGACAGTATTTAAGAGCCTGATCAAAAACCAGGCAAACACTGAAAGAAATTTGACATATAACTTATTAGTGAGAAAATATCATTTATTAACTCCTCATATGCTCATTAACGTAATGTTCATCATCCCCTTTGTGGTGTCGCTGGCCTCCTTACTCCTTTTAGCTCTCTCCTTATGGAGCCATACCAGGCAGATGAAGGGCACCGGTTCTAGGGATCTCAGCGCAGAGGCCCATGTGAGGGCCATGAAGTGTACGATTTCATTCCTACTCCTCTTCTTCTTGTACTATTTGAGTAACATTATGCTAAATTCAGCCTATGCTATTCTAGACAGTTTTACGGCAAAGATTTTCGCTAATGTGCTAGTGTTTTTCTATCCATCTGGCCATCCATTTCTTCTGATTTTGTACAACAGCAAATTGAAACAGGCTTCTCTCAGTGTCCTGGAGAAGCTGAAGAGTTGCATGAATCTAAG

>Horse_T2R42--Pseudo

CTGATCTTCGTGGTTATCTTTTTTCTGGAGACCTTGGCTGCAATGTTGCAGAACGGCTTCATGGTTGCTGTGCTGGGCAGGGAGTGGATGCGATGCTGCACACTGCCTGCAGGTGACATGATTGTGGCCTGCCTGGCTGCCTCCAGGTTCTGCCTGCATGGGATGGCCCTCCTGAACAACCTCCTGGACTCCTTTAATTTTCGTTCCATCGTTTCCTATTTCAACATCCCTTGGAACTTTATCAACACTCTCACTTACTGGCTGACTGCCTGGCTTGCTGTCTTCTACTGTGTGAAGATCTCATCCTTCTCTCATCCCATCTTCTGCTGGCTGAAGTGGAGGATTTCTCGGTCAGTGCTCAGGCTGCTGCTGGGTTCCCTGATCATATCTGGTGTGACAGTCATCCCAGCAGTCACCAGAGATATAATTCTTATACAGATGATTGCCTCCCAGAGTTCCCATGGAAACTGCACTCTGGCTGATAGAATAAGGACCTTCCATAGGTATTTTTTGATGCCTAATATAGTTCTGGTATTGTTGATTCCCTTCCTCCTGTTCCTGGTGTCCACCCTTTTGCTCATGTTCTCACTGCACTGGCACTTGCGGCAGATGAGGGCCCACAGATCCAGCCCACGTGATCCCAGCACCCAGGCTCACACCATGGCCCTGAAGTCACTTACCTTTCTTCCTCGTGTTCTACACATCATATTGCCTGTTCCTGATTATTGCTTTTATGAA

>Horse_T2R43--Pseudo

GGAATGCTAGGAAATGGGTTCATTGGACTGGTAAACGTCATCGAATGGATCAAGAATGGAAAGGTCTCATCAGGTGATTTCATCCTTTCCAACTTGGCTGTGGCCAAAGTCATTCAACTGTGGGTAACTCTATTTGGTTCATTTAATGTGGGTCTATCTCCACATCTGTATGCCACCAGGAAACTAGCAAAAACGCGGTCACATTCTTTGGACACTTACTGATCACTTTACCTGGTTTGCAACCTGCCTAAGCATTTTCTGCTTCCTTAAGTTGGCCAATTTCTCCCACTTCTTTTTCATCTGGCTGAAGTGGAGAGTGAACAGAGTGGTTCTTGCAATTTTCCTGAGGTCTTTTTTCTTACTGTTAATCTCTTAATGCAGGATGCTCTTAGTGAGTTGTGGGTGAATACCTGTAAAGTACAGGAAAGAAATATGACTTTGTACTTAGACAGTAATAAAATGTTCTGTCTCAAAAGCCTGTTCTTCTCAGGTTGACCTATGTTTTCCCCTTTCTTCTGTCTCTGACCTCTTTGTTCCTTTTATTTCTGTCCTTGCTGAGACACACCAAGAATTTGCTGCTCAACCTGATGGGTATGAGGGACTCCAGCATAGCGGCCCGTAAAAGGGCTGTGAAGATGGTGACAACCTTCTTCCTCCTCTTCATCATTTACTTTGTTTTCACTCTACTAGCAAGTTGGATCTTCCTTAAGGTATAAAGTATCAGGTTATGATGTTTCTCATCGTAATTTCAACCATCTTTCCCTCAGGCCACTCATTAATTATAATTTTGGGAAACAACGA

>Horse_T2R44--Pseudo

TTGGTTGGAATAATCAACTTCACTGACAAAGTAAGACACAGGAAAGTCAGTTTCATAAACCTCATCCTCACTAGCTTGTTCACAAGCAGAATATTTTTCTTGATCCTGATACTGACTGAATCATTTCTAGTGGTGTTATCTGCACATCCACATAGCATTGGTGCATTTTGAACAACTAATCTTCTGTTTTGCATAATGATCAACCACGTAAGTTTCTGGTGTGACACTTGCCTCAGTATCTTCTATTTCCTGACGATTACTACTTGTTTTGCATTCTCTCTTTTTCTGATTAAAGTGGGGAATTAGTAAGGTGGTTATATCTATTTTTCTGGAGTCTTTGTTCTTTTTAGCATTCCAGTGTTAGACCATAGAATTCCTCATATTTTAAATCTAGAAAAAAACAAGACTTGGAAGATTAATATGTATGAAACACAAACCATTTTCTTAGTGCTCATCAGTGTTGAGATCACCATGCCCATAACTACTTCCATACCATCGGTACTTCTGTTACTTCTTTCTCTTTGGAGGAACATCTAGAGAACGAAGTTCAGTATCACAGGGTCCCAAGATCCAAGTAGTGAAGCTTATGGAAGAGCAATGAGAATGGTAATTTCTTTCTTCTTTCTCTTTTTGATTAATATTGTGTTGATTCTTATAACAGTTACAGCCATTTATTGTACCCTAACAAGCTTATGTTGACATTTGGTAAATTCATAGCAACTGCTTATCCTTTAGACCATTCGTTCATCTTAATCTTGCAAAACAGCAAGCAGAAGCAAGCTA

>Horse_T2R45--Pseudo

TTTTTCCAACTAGCAACTGAAGAATTTGTTCTAGGAAATTTGGCCAATGTCTTCATAGCATGGGTGCATAGAACTGCATTGACTGAGTGAAGAGACAAAAGATGTCCTCAGCTGATTAAATTCTAACTTCTCTGACAGTCTCCAGGATTGGTCTACTTTGGGTAATATTAATAAATTGGTATTCAATTGTTTAACCCAGCTTTACATAGTTTAGAAGCAAGACTATTTATATTGCCTGGTCAATAACCAACCATTTTGGCATCTGGCTTGCCACTAGCCTCAGTATATTTTATTTGTTCAAGATAGCAAATATCTCCAGCTTTATTTTTCTTCACTTAAAGTGGAAAGTTAAAAGAGTGGTTCCCGTGATACTGTTGAGGACTTGGATCCTTTGGTTTTTCATCTAGTAGTGGTAAGCATAGATGACAGTATGTGCATGAATGACTACAAAGGATGCATCACTCGGAAGACCAAATTGAGGGACATTTTACGCCTTTCCAATTTAAGTATATTCACCCTAGCAAACTTCATACCCTTTATATGTCACTGACATCTTTTCTGCTGTTAATATTTTATCTATGGAAACATCTTAAGAAGATGCAGCTCAATGGCAACGATCCCAACATCCCAGTACCAAGGTCCACAAAAGAGCCATGCAAACTGCAGTCTCCTTTCTCTTGCTATATGCTGGTTACTTCCTGGCTCTAGTTATCTCAGTTTGGAGTTCTAATAGGCTGCATAACAGACCACTCATTTATCTTGATTTCAGGAAAGGAGAAGATAAGACAGGCCTTTCTGTCATTTCTGTGGCAGCTGAGGTGCTGGCTGAAAAAAAGAGAAGTAAGTGGGCCAGTATGTGTCTTCTAGCAAGAAACAAACTATCAG

>Horse_T2R46--Pseudo

ATGGTAACTTTACTACCCAGCATTTTTTCCGTCATAGAAATGACAGAATTTATTCTGGGAAATTTCGCTAATGGCTTCATAGCACCGCTGAACTTCATTGCCTGGGTCAGGAGACGAAAGATGTCCTCAGCTGATCAAATTCTCATGGCTCTGGCGGTCTCCAGAATTGGTTTGCACTGGGTAATATTAATAAATTGGTATACAACTGTGCTCCATCCAGTTTTATATAGTTTTGAAGTAAGAATTTTTGTTCATATTATTTCTGGTAACACTATTAGGGACTTTGGTCTTTTTGGTTTTTTTATTTTGCAGTGATATGCATAGATGAGAAAACACAGACTAATGTGTATGAAGGAAACATCACTGGGAAGACCAAACTGAGGGATATTTTACGTGTTTCAAATATGACTGTATTCGTGCTAGTACGCTTATTACCATTTTCTACGTCCCTGACATGTTTTCTGCTGTTAATCATTTCTCTATGGAAACATCTCAAGAAGATGCAGCTTAATGGCAAAGGATACCAAGATCCTAGCACCAAGGTCCACATAAGAGCCATGCAAACTGTGTTCTCCTTTCTCTTGCTATATGCCAGTTACTTACTGGCTCTAGTTATCTCAGTTTGGAGTTCTAATAGGCTGCAGAATGAACTGCTTCTCATGCTTTGTGAGGTTCTTGCAGTACTGCATCCATTAAGCCACTTGTTTATCCTGATTTGGGGAAACAAGAAGCTAAGACAGGCCTTGAAAA

>Horse_T2R47--Pseudo

ATGCCAAATACAATGGAGACAATATACATGATCCTGATTGCTAGCGAATTGAGTATAGGAATTTGGGGAAATGGATTTATTGTACTGGTTAACTGCACTGGCTGTTTCAAAAGGAGCATTATCTCCTTGATTGACATCATCCTGGTCCCCTTAGCCATCTCCAGAATCTGTTTGTTGTGTGTGATATCTTTAGATGGCTATGTTATGCTGCTCTCTCCAGATACATATGACCATGGGGAGCTAATGAACATTTTGGATGTTTTGTGGACATTTAGCAATCATTCAAGTGTCTGGTTTACTTCTTGCCTCAGCATCTTCTACTTACTCAAGATAGCCAATATAGCCCACCCATTTTTCCTCTGGCTGAAGCTAAAGATTAACAGGGTCATCCTTGGGATTCTTCTGGCATCCTTTCTCCTCTCCTTAATTATTAGTGTTGCACTGAATGAAGATTCCTGGTATAACTTCAAGGTCAATCATGAAGGAAATATAACTTTGAAATTCAAAGTGAGTAAAGTCTCAAATGCTTTCAAACAGATTATCCTGAACCTGGGGGCGATTGTTCCCTTTATACTCTGCCTGATATCATTTCTGTCGCTGCTTTTCTCCCTATTTAGACACACCAAGCAGATGAAATTTCATGTCACAGGGTCCAGAGACACCAGCACAGAGGCCCACATGAGGGCCATAAAGACAGTGCTGATCTTTCTGCTCTCTTCATTGTGTACTATGCCGTCTTTCTTGTAATGACCTCTAGCTATCTGATTCCTCAGAGAAAATTACGGATAATGTTTGGTGGCATAATAGCTGTCATTTTCCCATCTAGCCATTCTTTTATCCTGATAATGGGGAACAGCAAGCTGAGGGAGGCTTTTCTGAAGGTGTTAAGGACTGTAAAGGGTTTCCACCAAAAAAGGAAATCTTTTG

>Horse_T2R48--Pseudo

ATGGTAGCTTTACTACCAAACATTTTTTCTGTTCTAATAATGACAGAATTTATTCTGGGAAATTTTGCCAATGGCTTCATAGCACTGGTAAACTGCATTGACTGGGTCAAGAGACAAAGGATGTCCTCAGCTGATCAAATTCTCACAGCTCTGGCGATCTCCAGAATTGGTTTGCTCTGGGTAATATTAATAAATTGGTATACAGCTGTGCTCCGTACAGGTTTATATAGTTTAGAAGTAAGAACTGTTGTTCATGTTACTTCTCGTAATAATCTTGGGGACTCCAGTCTTTTTGGTTCCTCATATTACAGTGCTATGCTTAGATGAGAATATGCGGACTAATGAATATGAAGGAAACATCACTCAGAAGACCAAATTGAAGGGCGTTTTTCACCTTTCATTTATGACTCTATTCACACTAGTAAACTTCATACCATTTTCTATTTCCCTGACATCTTGTCTGCTGTTAATCATTTCCCTGTGGAAACATCTCAAGAAAATTCAGCTGAATCTAAAAGGATCCCAAGATCTCAGCACCAGGGTCCACATAAGAGCCATGCAAACTGTGGTTTCCTTTCTCCTGCTACATGTCAGTTACTTCCTGGCTCTAATTATCTCAGTTTGGAATTCTGAAAGGCTGAAGAATCAACTATTTGTCATGCTTTGTGAGGTTCTTGCAATGTTGTATCCTCTAAGCCACTCATTTATCCTGATTTGGAAAAACAAGAAGCTAAG

>Horse_T2R49--Pseudo

AGAGCACAGGACATCTTATTGGTTGTCTTAACCATGGGATTCATAATAGGTATTTGGGGAAATGGATTGTTTGGAATAATCAACTTCACCAACTGGGTAAGACACAGGGCAATCATCATCATTAACCTCATCCTCAACAGCTTATCCACAAGTAGAATATGTTTCTTGATCCTGATGCTGATTGAATCATTTCTACTGGTGCTGTCTGTACATCCACATAGCATTGGTCCATTTTGAAAAACTAATAATTTGTTTCACACACTGATAAAGCACTTAAGTGTCTGATGACGTCTGCCTCAGAATCTTGTTTCCTGAAGATAGCTACTTTTTTTCATCTGCTTTTTCTTTGATTAAAGTGGAGAATCAACAGGGTGATTATTACTATTTTTCTAGGGTCTTTGTTTTCTGTTGTTTTTAGAATCCTGGTTATAGGCCATATAATTCCTGATATTTTAATTCAAGAAACAAACAATACTTGGAAGATTAATAGGTATGAAACACAAACCATTATTTTCTTAGTGCACATCAGTGTTGAGATCATCACGCCCATAGCTATTTCCATAGCATCAGTTCTTCTGTTACTTCCTTCTCTTTAGAAGCATACCTAGAGGATGAAGTTCAGTATCATAGGGTCCCAAGGTCCCAGCAGTGAAGCTCATGGAAAAGCCATGAGAATGGTGACTTCCTTCTTATTTCTCTTTTTGATTAATATTGTGTTGATTCTTATAACAGTTTATAGGCACTTATTGTCCCCTAACAAGCTGATATTGACATTTGGTAAATTAACAGCATTTACCCATCCTTTAGGCCACTTTTTTTATCCTAATTTTGAGAAAGAGTAAGCTCAAGCAAGCTGCTCTTAGAATTTTGGGGCAGCTGAAGTGTTTTCTG

>Horse_T2R50--Pseudo

TCATCCATGTTGATCTTCATGGTCATCTTTTTCATGGAGACCTTGGCTACAATGTTGCAGAATGGCTTCATTGTTGCTGTACTGGGCAGGGAGTGGGTGAGATGCTGCACACTGCCTTCAGGTGACATGATGGTATCCTGCCTGGCTGCCTCCTTATTCTGCCTGCATGGGATGGCCCTCCTAAACAATCTCATTGACTCCTTTAACTTTTGTTCCAAAGTTTACTATTTCAACACTTCCTGGGGCTTTATCAACGCTCTTACTTTCTGGCTTACTGCCTAGCTTGCTGCCTTCTACTGTGTGAAGATCTCATCCTTCTTTCATCCTGTCTTCCTCTGGCTAAAGTGGAGGATTTCTCGGTCAGTGCCCAGGCTGCTGCTGGGCTCCATGATTATATCTATCGTGCCAGTCATTCCATTAGCCACTTGGAATAGCATTCTTGTGCAGCTGAGTGCCTCCCAGAGTTCCCATGGAAACGGCACCCTATGTGAAAGAATACAGACCATCTCTCTCTACTCTTTTTTGCCTACAGTGCTTATGTTGTTGATTCCCTTTCTCCTGTTCCTTGTGTCCACCCTCTTGCTCATATTCTCACTGTACAGGCACTTGGGGAAAATGAGGGACCACAGATCCGGCCCATGTGATCCCAGCACCCAGTCTCACACCATGGCCCCGAAGTCATTTACTGTCTTCCTTGTCTTCTACACATCATATTTCCTCTCCCTGATTATTGCTTTTATGAAAATCACAACACTGAAGAATCAGAGCCATGGGGCCTGGGAAGTGGTGACCTATGCAGGCATCTGTCTGCATTCTAGCATCCTGGTGCTAAGCAGCCCAAAGCTGAGAAAGGCCCTGAAG

>Horse_T2R51--Pseudo

ATGAAGCCAGGGCTCACAGCCTTCTTTATACTGCTTTTTGCCCTCCTCTGTGTCCTGGGAATCCTGGCCAATGGCTTCATTGTGCTGGTGCTGAGCAGAGAATGGATGCGGAGTGGGAGGCTGCTTCCCTCTGACATGATCCTTATTAGCTTGGGTGCCTCCCGCTTCTGCCTGCAGTGGGTTGGAATGGTGAGAAACTTCTACTTCTTCCTCCATCTGGTCGAGTACTGCAGGGGTACCGCATGGCAGTTCTTTGGTCTACACTGGGACTTCCTGAACTCAGCCACCTTCTGGTTCGGCACCTGGCTCAGTGTCCTCTTCTGCGTGAAGATTGCTACCTTCACCCACCCCACCTTCCTCTGGCTGAAGTGGAGGTTCCCAGGGTCAGTGCCCTGGCTCCTCCTAGGCTCTCTCCTGGTCGCTTTCATTGTCACCCTGCTCTTCTTTTGGGGGAACTACAGTATGTATCAAAGGATTCTTCATTAGAAAATTTTCTGGAAACATGACCTACGAGCAATGGAGCAGGAGGCTGGAAATTTACTATTTCCTACCCTTGAAATTTATCACGTTGTCAATTCCTTGCTCTATTTTCCTGATCTCAATTGCACTGTTGATTACTTTTCTGAGGAGACACACACGGAGAATGTGGCATAATGCCCACAGCCTGCAGGACTCTAGCACCCAGGCTCTCACCAGCGCTCTGAGGTCAATCATCTCCTTCCTTGTTCTTTATGTGATGTCCTTTGTGTCACTGGTCATCGATGCTGCACTGTTTTTGTCCTCAGACAGTGACTGGTACTGGCCATGGCAAATTTTAACTTACCTGGGCACATCTGTCCATTCCTTTATTCTCATCCTCAGCAACCTCAGGTTTCGAAGGGTGTTCAGGCAGTTACTTCTGTTGGCCAGGGGCTTCTGGGT

>Horse_T2R52--Pseudo

ATGTCCTCCTCACCCATGTTGATCTTCATGGTCATCTTTTTCTTGGAGTCCTTGGCTGCAAATTTGCAGAATGGCTTCATGGTTGCTGTACTGGGCAGGGATTGGATGCAGTGCCACACATTGCTCACAGATGACATGACTGTGGCCTGCTTAGCTGCCTCCAGGTTCTTCCTGCATTGGATGATTCCCCTGAATAAACTCCTAGCCTCCTTTGATTTTTGCTCCAAAATTCACTATTTCAACATCCCTTGGAGCTTCATCAACACACTTTCTGCCTCCCTGCCTGGCGTGCTGTCTTCTACTGTGTGAAGATCTCGTCCTTCTCTCATCCAGTCTTCTTCTTGCTGAAGTGGAGGATTTCTCAGTCACTGCCAAGGCTGCTGCTGGGTTCCCTGATCATATCTGGTGTGACAGTCATCTCATTAGCCACTGGGCATAGCATTCTTGTGTAGATGGTTGCCTGCCATATTTCCTATGGAAACAGCACTGTGGCTGATATAACAGAGCACATCTCTTTGTACATTTTTCTGGCTAAGATACTGCTTGTGTTATTGATTTCCTTCCTCCTGTTCCTGGTGTTCACTCTCTTGCTCATGTTCTCACTTCACCGGTCCTTGAGGCAGATGAGGGATCATAGACCTGGCCCACACGATCCCAGCACCCAGGCTCACACTATGGCCCCGAAGTCACTTGCTGTCTCTCTCGTCTTCTACACATAATATTTCTTGTCCCTCATTATTGTTTCTATGCATATCACAACACTTCATAATCACTGGAACTGGGTCTAGGATGTGGTGATCTATGCAGGCATCTGTCTATGCTCCAGCATCCTGGTACAAAGCAGCCTCAAGCTGAGAAAGGCCCTGAAGA

>Horse_T2R53--Pseudo

ATGCTGATTGCTGCCCTAAGACTGCTGATGCTGGTGGCAGTGGCTGAATTTCTCATTGGCCTGGTTGGAAATGGAATTCTTGTGGTATGGAGTTTTGGAGAATGGGTCAGAAAATCCAAGGGGTCCTCATACAACCTCATTGTGCTGGGCCTGGCTGTTTGCCGATTTCTCCTGCAGTGGTTGATTATGATGGACTTAATCCTGTTTCCGCTTTTCCAGAGCAGCTGTTGGCATCGCTATCTCAGTGTCTTCTGGGTTCTGGTAAGCCAGGACAGCCTGTGGTTTGCCACTTTCCTCAGATTCTTCTACTGCAGGAAGATCACGACCTTTGAACACCCCATTTACTTGTGGCTGAAGCAGAGGGCCTATTGCCTGAGTCTCTGGTCTGGGGTACCTCATGATCAGTTTGTGACTTGTGGTCCACATTGGCTTAAAGCCTTGCAATCCTTCCCATGGAAACAGCAGCATTCTATACCCCTTTTCAAACTGGCACTATCTGTGTATTTTACATCTCAGTGCAGGAAGTGTGGTGCCTTTCATGGTGTTTCTGGTTTCTTCTGGGATGCTGATCGTCTCTTTGTATAGACACCACAGGAAGATGAAGGCCCATACAGCTGGTAGGAGGGATGCTCGGGCTCAGGCTCACATCACTGTCCTGAAGTCCTTGGGTTGCTTCCTTGTACTTTACGTGGTTTATGTTCTGGCCAGCCCCTTCTCCATCACCTCCAAGTATTCTCCTGCTAATCTCACTACTGTCTTCATCTCTGAGACACTCATGGCTGCCTATCCTTCTCTTCATTCTGTCATATTGATCATGGAGAATCCCAGGGTGAAGCAGATTTGTCAGAGAATTTTGTGGAAGATAATGTGTGCTTGGAGATC

>Horse_T2R54--Pseudo

CAACTCACTGTCTTCTTCATGGTCATCTATGTGGTCGAGTCCTTGACAATAATTATGCAGAGTGGCTTAATTGTTGCAGTGCTGGGCAGAGAGTGGGTGCAGGTAAAGAGGCTGTCACCTGTGGACGTGATTCTCACCAGCCTGGGCATCTGCCGCTTCTGTCTCAGTGGGCATCGATGCTGTATAATTTTTGCTCCTATTTCAACCCTAACTATGTATTTTGGTACTACTCGATCACCTGGGACTTTTTAAATACTCTTACATTCTGGTTAACCAGCTTACTTGCTGTCATCTACTGCGTCAAAGTCTCTTCCTTCACCCACCCCGCCTTCCTCTGGTTGAGGTGGAGAATTTTGAGGTTGGTTCCCTGGCTGTTACTGGCTTCTCTGCTGATTTCTTGTGTGACTCTTATCTCTTCAGCTATTAGGAATCACATGAAGATACAGATAATCTCCATGGAGCATTTCCCTAGAAACAACACTATGGTTGAAAGACTTAAGATGCTTCTGAAGAAATTTACCTTATCTCAGCCAGTGGTTGTGTTGATTATTCCTTTCCTCCTGTTCCTGGCCTCCACCATCTTGCTCATGACCTCATTGTCCCAACACTTGGAGCAGATGCAACATCACAACACTGACCACAGCAACTCCAGCGTGAACGCTCACTCCACTGCCCTGAGGTCTCTTGCTATCTTCCTTCTCTTCTTCACCTCTTATTTTCTGACCATACTCATCTCCATTCTGAGCATCCTATATGATAAGAGATCCTGGTTCTGGGTCTGGGAAGCTGTTATCTATGCTATAGTCTCTGTTCAATCCACTTCACTAATGCTGAGCAGCCCTACATTGAAAAAGGTTTTAAAGGTAAGGTGCTGGGGCCTGGAGGCTG

>Horse_T2R55--Pseudo

TTGGAGAAAGTTTTCATGATTGTAGCAGGTGGGGAATTTATAGCAGGAATTTTAGGGAATGGATTTATTGGACTCACAAATTGCATTGCCTGGATTAGAAATCGGAAGTTATGTTTGGTTGGCTTCATTCTTACCAGTTCGGCCTTCACCAGAATCAGTCAATTACGGCTAACAATTGTCAATTTGTTTTCAGTGGTGGTCTATCAGGAAATCCCTGATACTAAGAAAAGAAATCATATACATACTGGTATCTGGATACTGGCCAACCACTTGAGCACTTGGTTTGCTACTTGTCTCACTGTCTTTAATTTTCTGAAGATCAACAATTTCTCCTATCCCCTTTTCCTTTGGCTGAAATGGAGAATTAATCAGGTAGTTTTCATGCTTCTGCTGTTACCTGTGCCCTTCCTGTTCATCAACTTTCCTTTCCCATACAGTTTTGATGTTTTCTGGTGTCATGTCCAAAAAAATATAAAAGAGATATGACTGGGTTATTCAATGTGAGTAAAAATAAACATGTAAGTGCCATGACAGTCTTCATTACTGGGTCCCTCCCTCCTTTCTCTCTTTCCTTGATTTCCTTTCTCCTGTTGCTCCTTTTCTTTGTGGAGACACATGAAACACAATTTGCTCAAAGTCAGGGTTTCCAAGGACCCCAGTATGGAGGCCCATGTCAGAGCCATGTTTTCTTTCTTGAGCTCTTTTTTCTGTACCAGTTTGCCTTTTCCTGATATTTTTGGGTTATTTTTCACTACAGAACAAGCTGGTTGTGATGTTTGGTTACATGTTAAGAATTCTATATCTTTGGGGTCACTCAAATGTCATGATTTTCCTCAGCCAAGTGAGGAAAGCCTTCTTGGGGATTCTCTGGCACCTCAAGTGTAGCCTCAA

>Human_T2R25--Pseudo

TTGATCTTCATAGCCATCTTTTGCCTGGAGTCATTGGCTGCAATGCTGCAGAATGGATTCTTGGTCACAATGCTGGGCAGGGAGTGGGTAAGGTGCCGGATGCTGTCCACAAGTGACATGATTGTGGCCTGTCTCGCTGCCTCCCGTTTCTGCCTGCATGGGGTAGCCATGGTGAACAACCTCCTGGCCTCCTTAGATTTTTCGCGTGCAGTTCCCTATATGAACATCTTCTGGGACCTTTTCAATGCCCTCACTTTGTGGTTTACTGCCTTGCTTGCTGCTTTCTACTGTGTGAAGATCTCATCTTTCTCCCACCCCACCTTCGCCTGGCTGAAGTGGAGGAACTCTCGGTTAGTGCCCAAGCTGATCAAGGGCTCCCTGATCATCTGTGGCCTGGAAGTCATCTCATCAGCCACTGGGAACATCCTGTTTGGTCAGAGGAAGGTCTCCCTGAGTTCCTACAGAAACGAAACTCTAGTTTATAGAGTGCAGGCTTCATTTCAGCTCTACTTTTTCCTTTATGAAGGGTTTGTGTGGTCGATTCTGTTCCTCCTGTTCCTAGTGTCCACTGTCTTGCTCATAGTCTCACTGTGCTGGCAGTTGGGGTAGATGAGGGACCTCAGGCCCGGCCCCTGTGATCCCAGCACCCAGGCTTACACTATGGCTTTAAAGTCACTCACCTTTTCCCTCATCTTCTGTACATTGTACTTCCTGTCCTTGTTTGCTTCTGCTTTGAAAATCATAAACTTTCAGAATCACTGGCACTGGGCCTGGTAAGTGCTAATCTATGCCAACATCTGTCTGCACTCTACCGTCCTGGTGCTGAGGAGCCCCAAACTGAAAAAGGGCCTGAAG

>Human_T2R26--Pseudo

ATGCTCCCCTTGTGAATCTATGGAGTTGAGGGTTTCTGTTATTTCACCCAGCACGTCACCTGACACAAAACTGAAAGAGAGGTCTGTTTTAGCTTCCTACTTCTCATAATCAGGATGAATGAGTGGAATGAAGGATACATGATTGCAAGAGTTTGGTAAAGCAGGAATACAAGATTGCTCTGCTGTGTCCTAGGATTCCAAGTTGATGTGATTATACACAGAAAGTAAATGGCAAATAACATAMGGAAGGAGATAACAGTTTGCAAAGGTTTTATATGGACCTTGGTGCTGAGATCTTGAGATCCTTTGCCATGGAGCTGCATCATCTTGAGATGTTTACACGGAGAACAGACTAACAGCAGAAAAGATATTAGAGTCAGAATGAACAGTGTGAAGTTTGCTAGCATGGTTGGTTATAGTCATGTTTGAAAGGTGTATTGCATTCCTCAATTCGATCTTCCAAGTCACATTTCCTTCAAATTCTTTTGTCCACACACTGTCATCCATGGTTACCACAGCAAGATTACAAATCAAAAATACCAAGGACCCCAACAGTATCACAAGAAGAACACTCTTAATTCTCTTCTTTAGGTGGAGAAAAATAAAATTGGAGAAATTGGCAATCTTGAACAAATAAAATATGCTGAGGCTCGTAGCAAGCCAAATGCTGAAATGATTGATTATTGCCGAGACATTAGAAGGAACAATTCTTACTTCTAAACTATATAAAGCTGAATTAAACACAGTTGCATACCAATGTAATAATATGATCAAAGTAAACCAATTCTGGAGAATGCCAGAGCAGTGACAATTTGGTCAGTTGAGGAGATCTTTTGTGTCTTAACCCAGTCATTGACATTAAYTAGAGCTATGAAGCCATTGGCAACATTTCCAAGAACAAATGCAAACACTACCAGAATTGATAAAATGATGAGCAGAACACAT

>Human_T2R27--Pseudo

TCAGCTATTCTTCATATTATCATGATGTCAGCAGAATTCTTCACAGGGATCACAGTAAATGGATTTCTTATCATTGTTAACTGTAATGAATTGATCAAACATAGAAAGCTAATGCCAATTCAAATCCTCTTAATGTGCATAGGGATGTCTAGATTTGGTCTGCAGATGGTGTTAATGGTACAAAGTTTTTTCTCTGTGTTCTTTCCACTCCTTTACGTCAAAATAATTTATGGTGCAGCAATGATGTTCCTTTGGATGTTTTTTAGCTCTATCAGCCTATGGTTTGCCACTTGCCTTTCTGTATTTTACTGCCTCAAGATTTCAGGCTTCACTCAGTCCTGTTTTCTTTGGTTGAAATTCAGGATCCCAAAGTTAATACCTTGGCTGCTTCTGGGAAGCGTTCTGGCCTCTGTGAGCATTGCATCTGTGTGTCGAGGTAGATTACGCTAAAAATGTGGAAGAGGATGCCCTCAGAAACACCACACTAAAAAAGAGTAAAACAAAGATAAAGAAAATTAGTGAAGTGCTTCTTGTCAACTTGGCATTAATATTTCCTCTAGCCATATTTGTGATGTGCACTTCTATGTTACTCATCTCTCTTTACAAGCACACTCATCGGATGCAACATGGATCTCATGGCTTTAGAAATGCCAACACAGAAGCCCATATAAATGCATTAAAAACAGTGATAACATTCTTTTGCTTCTTTATTTCTTATTTTGCTGCCTTCATGACAAATATGACATTTAGTTTACCTTACAGAAGTCACCAGTTCTTTATGCTGAAGGACATAATGGCAGCATATCCCTCTGGCCACTCGGTTATAATAATCTTGAGTAATTCTAAGTTCCAACAATCATTTAGAAGAATTCTCTGCCTCAAAAA

>Human_T2R28--Pseudo

ATGATATGTTTTCTGCTCATCATTTTATCAATTCTGGTAGTGTTTGCATTTGTTCTTGGAAATGTTGCCAATGGCTTCATAGCTCTAGTAGGTGTCCTTGAGTGGGTTAAGACACAAAAGATCTCATCAGCTGACCAAATTTCTCACTGCTCTGGTGGTGTCCAGAGTTGGTTTACTCTGGGTCATATTATTACATTGGTATGCAACTGTGTTTAATTTGGCTTCACATAGATTAGAAGTAAGAATTTTTGGTTCTAATGTCTCAGCAATAACCAAGCATTTCAGCATCTGGGTGTTACTAGCCTCAGCATATTTCATTTGCTCAAGACTGCCAATTTCTCCAACCTTATTTTTCTCCACCTAAAGAAAAGGATTAAGAATGTTGGTTTGGTGATGCTGTTGGGGCCCTTGGTATTTTTCATTTGTAATCTTGCTCTGATAACCACGGGTGAGAGTGTGTGGACAAAAGAATATGAAGGAAATTTGTCTTGGATGATCAAATTGAGGAATGCAATACAGCTTTCAAACTTGACTGTAACCATGCCAGCAAACGTCACACCCTGCACTCTGACACTAATATCTTTTCTGCTGTTAATCTATTCTCCATGTAAACATGTCAAGAAGATGCAGCTCCATGGCAAAGGATCTCAACATCTCAGCACCAAGGTGCACATAAAAGCTTTGCAAACTGTGATCTCCTTCCTTATGTTATTTGCCATTTACTTTCTGTGTCTAATCACATCAACTTGGAATCCTAGGACTCAGCAGAGCAAACTTGTATTCCTGCTTTACCAAACTCTTGGATTCATGTATCTTTTGTTCCACTCATTCATCCTGACTATGGGAAGTAGGAAGCCAAAACAGACCTTTCTTTCA

>Human_T2R29--Pseudo

ATGATAACTTTTCTACCCATCATTTTTTCCATTCTAGTAGTGGTTACATTTGTTCTTGGGAATTTTGCTAATGGCTTCATAGTGTTGGTAAATTCCATTGAGTGGGTCAAGAGACAAAAGATCTCCTTTGCTGACCAAATTCTCACTGCTCTGGCAGTCTCCAGAGTTGGTTTGCTCTGGGTAATATTATTACATTGGTATGCAACTGTTTTGAATCCAGGTTCATATAGTTTAGGAGTAAGAATTACTACTATTAATGCCTGGGCTGTAACCAACCATTTCAGCATCTGGGTTGCTACTAGCCTCAGCATATTTTATTTCCTCAAGATTGCCAATTTCTCCAACTTTATTTTTCTTCACTTAAAAAGGAGAATTAAGAGTGTCATTCCAGTGATACTATTGGGGTCTTTGTTATTTTTGGTTTGTCATCTTGTTGTGGTAAACATGGATGAGAGTATGTGGACAAAAGAATATGAAGGAAACGTGAGTTGGGAGATCAAATTGAGTGATCCGACGCACCTTTCAGATATGACTGTAACCACGCTTGCAAACTTAATACCCTTTACTCTGTCCCTGTTATCTTTTCTGCTCTTAATCTGTTCTTTGTGTAAACATCTCAAGAAGATGCAGTTCCATGGCAAAGGATCTCCAGATTCCAACACCAAGGTCCACATAAAAGCTTTGCAAACGGTGACCTCCTTCCTCTTGTTATTTGCTGTTTACTTTCTGTCCCTAATCACATCGATTTGGAATTTTAGGAGGAGGCTGTAGAACGAACCTGTCCTCATGCTCAGCCAAACTACTGCAATTATATACCCTTCATTTCATTCATTCATCCTAATTTGGGGAAGCAAGAAGCTGAAACAGACCTTTCTTTTGATTTTGTGTCAGATTAAG

>Human_T2R30--Pseudo

AAGGTCTCCCTGATTGATTTTATTCTCAACTGCTTGGCCATCTCCAGGATATGTTTCCTGTAGATAACAATTTTAGCTACCTCTTTCAATATAGGCTATGAGAAAATGCCTGATTCTAAGAATCTTGCAGTAAGTTTTGACATTCTCTGGACAGGATCCAGCTATTTCTGCCTGTCCTGTACCACTTGCCTCAGTGTCTTCTATTTCCTCAAGGTAGCCAACTTCTCCAATCCCATTTTCCTCTGGATGAAATGGAAAATTCACAAGGTGCTTCTCTTTATTGTACTAGAGGCAACGATCTCTTTCTGCACAACTTCCATTCTGAAGGAAATAATAATTAATAGTTTAATCTAAGAACGGGTAACAATAAAAGGCAACTTGACATTTAATTATATGGATACCATGCATGATTTCACTTCTCTGTTTCTCCTTCAGATGATGTTCATCCTTCCTTTTGTGGAAACACTGGCTTCCATTCTTCTCTTAATCCTCTCCTTATGGAGCCACACCAGGCAGATGAAGCTACATGGTATTTATTCCAGGGATCCCAGCACAGAAGCCCATGTAAAACCTATAAAAGCTATAATTTCATTTCTACTCCTCTTTATTGTGCATTATTTCATCAGTATCATACTAACATTGGCCTGTCCTCTTCTAGACTTCGTTGCGGCAAGGACTTTTAGTAGTGTGCTGGTATTTTTCCATCCATCTGGCCATTCATTTCTTCTAATTTTACGGGACAGCAAACTGAAGCAAGCTTCTCTCTGTGTCCTGAAGAAGATG

>Human_T2R31--Pseudo

ATGTTCGTTGGAATTAATATTTTCTTTCTGGTGGTGGCAACAAGAGGACTTGTCTTAGGAATGCTGGGAAACGGGCTCATTGGACTGGTAAACTGCATTGAGTGGGCCAAGAGTTGGAAGGTCTCATCAGCTGATTTCATCCTCACCAGCTTGGCTATAGTCAGAATCATTCGACTGTATTTAATACTATTTGATTCATTTATAATGGTATTGTCCCCTCATCTATATACCATCCGTAAACTAGTAAAACTGTTTACTATTCTTTGGGCATTAATTAATCAGTTAAGTATCTAGTTTGCCACCTGCCTAAGCATTTTCTACTTGCTTAAGATAGCCAATTTCTCCCGCTTATTTTTTGCCTGGCTGAAGTGGAGAATGAACAGAGTGGTTCTTGTGCTTTTCCTGCGGTCTTTGTTCTTATCGTTTGTTTACCTTTTTATGTCCAATGCCATTAGTGAGTTGTGAAAAAAACATGACTTTGCACTCAGATACAAGTAAAATAGTCTGTCTTCAAGGCCTTAGGCTTCTCAGCTTGACATACGTTATTCCCTTTCTTCTGACTCTGACCTCTTTGCTCCTTTTATTTATATCCTTAGTGAGACACACCAAGAATTTGCAGCTCAACTCTCTGGGCTCAAGGGACTCCAGCACAGAGGCCCATAAAAGGGCCATGAAAATGGTGATAGCCTTCCTCCTCCTTTTTTTTATTAACTTTATTTCCACTTTAATAGGAGATTGGATCTTCCTTGAGGTAGAGAATTATCAGGTCATGACGTTTATTATGATGATTTTACTTGCCTTTCCCTCAGGCCACTCATTTATTATAATTTTGGGAAACAACAAGCTAAGACAGAGCTCCTTGAGACTACTGTGGCATCTTAAATTCTCTCTGAAAAAAGCAAAACCTTTAAC

>Human_T2R32--Pseudo

ATGGTATATTTTCTGCTCATCATTTTATCAATTCTGGTAGTGTTTGCATTTGTTCTTGGAAATTTTTCCAATGGCTTCATAGCTCTAGTAAATGTCATTGACTGGGTTAAGACACGAAAGATCTCCTCAGCTGACCAAATCCTCACTGCTCTGGTGGTCTCCAGAATTGGTTTACTCTGGGTCATATTATTACATTGGTATGCAAATGTGTTTAATTCAGCTTTATATAGTTCAGAAGTAGGAGCTGTTGCTTCTAATATCTCAGCAATAATCAACCATTTCAGCATCTGGCTTGCTGCTAGCCTCAGCATATTTTATTTGCTCAAGATTGCCAATTTCTCCAACCTTATTTTTCTCCACCTAAAGAAGAGAATTAGGAGTGTTGTTCTGGTGATACTGTTGGGTCCCTTGGTATTTTTGATTTGTAATCTTGCTGTGATAACCATGGATGACAGTGTGTGGACAAAAGAATATGAAGGAAATGTGACTTGGAAGATCAAATTGAGGAATGCAATACACCTTTCAAACTTGACTGTAAGCACACTAGCAAACCTCATACCCTTCATTCTGACCCTAATATGTTTTCTGCTGTTAATCTGTTCTCTGCATAAACATCTCAAGAAGATGCAGCTCCATGGCAAAGGATCTCAAGATCTCAGCACCAAGGTCCACATAAAAGCTTTGCAAACTGTGATCTCCTTCCTCATGTTATATGCCATTTACTTTCTGTATCTAATCACATTAACCTGGAATCTTTGAACACAGCAGAACAAACTTGTATTCCTGCTTTGCCAAACTCTTGGAATCATGTATCCTTCATTCCACTCATTCTTCCTGATTATGGGAAGCAGGAAACTAAAACAGACGTTTCTTTCAGTTTTATGTCAGGTCACA

>Human_T2R33--Pseudo

ATGCCACCTGGAATTGGAAATACCTTTCTGATAGTAATGATGGGAGAATTCATAATCTGAATGTTAGGGAATGGTTCATTGTACTAGTTAACTGCATTGACTGGTGAGGAGTCAAATGATCTTATTAGACAACTGCATCCTCACCAGCCTGGCTATCTCCACAATCAGTCAACTTTGGATAATACTACTTGATTCATTTGTAACAGCATTATGGCCACATCTATATGCCTTCAATAAACTAATGAAATTTATTCATATTTTTTGGGCACTGACCAATCACTTAGTTACCTGGCTTGCTGCCTTAGTGTTTTCTACTTCTTTAAAATACCTATTTTTCCCACCCATGCTTCATCTGGCTGAGATGGAGAATTAGCAGAACGCTACTTGAACTCCCACTGGGGTCCTTGCTCTTACTGTTTTTCAACCTTGCATTAACAGGTGGACTTAGTGACTTGTGGATTAACATCTACACAATTTATGAAAGAAACTCAACTTGGTCTTTAGATGTAAGTAAAATTCTATATTGTAGCCTCTGGATTCTTGTCAGTTTGATCTACTTAATTTCCTTTCTTCTGTCCCTGATCTCACTGCTCCTTTTAATTCTGTCCTTGATGAGACATATCAGGAATTTGCAGCTCAACACCATGGGCCCGAGGGACCTCAGAATGAAGGCCCATAAGAGGGCCATGAAAATGAAAATGAAAATGATGGTGTCTTTTCTCCTCTTCTTTTTGGTTCACTTTTCTTCTCTCCTACCAACAGGTTGGATTTTCCTTATACAGCAGAAATAGCAGGCCAATTTTTTTGTCTTGTTAACGTCAATTATTTTTCCTTCAAGCCACTCATTTGTCCTAATTTTGGAAAACTGCAAGCTGAGACAGACTGCTGTGGGACCACTGTGGCATCTTAAGTGCCACCTAA

>Human_T2R34--Pseudo

ATGTTGGCGGCTGCCCTAGGATTGCTGATGCCCATTGCAGGGGCTGAATTTCTCATTGGCCTGGTTGGAAATGGAGTCCCTGTGGTCTGCAGTTTTAGAGGATGGGTCAAAAAAATGTAAGGAGTCCCTATAAATTCTCATGATTCTGGTAAGTAGCCACTTTCTCCTACTCAGGCCGATCATGTTGGACATAAGTCTGTTTCCACTTTCCCAGAGCAGTGGTTGGCTTTACTATCTTAATGTCTTCGAGTCCTGGTAAGCCAGGCCAACATGTRGTTTGCCACTTTCTTCAGTGGCTTCTGCTGCATGGAGATCATGACCTTTGTCCCGCTGACTTCTTGTAGCTGAAAAGACTGGGTTTTTGTTTTTTGCTAGTGTCTTTCAAGATCACTTTTTATTTCTCAGCTCTTGTTGGCTGGACCCTTTAAAAACCCTTAACAGGAAACAGCAACATCCTGCATCCCATTTTAAATCTGTTATTTTTATAGATTGCTGTCCAGTGAAGGAGACTGATTGCTATTTGTGATGTTTCTGTTCCACTTGTCTTTTTGTAAAGACATCACAGGAAGATGGAGGACCACACAGCTGTCAGGAGGAGGCTCAAACCAAGGTGCTCATCGCTCTGAACTTCCCCCTTTACATGGTTTCTGCCTTGGCCAGMCACTTTTCCATGACCTTCTAATCTCCCTCTGATCTCACCATTCTTGCCATCTCTGCAACACTCATGGCTGTTTATACTTCATTTCCGTCTATTGTAATGGTTATGAGGAATCAGACTTGTCAGAGAATTCTGTAGGAGATGATATGTACATGGAAATCCTAG

>Lizard_T2R38--Pseudo

TGGGTCTTTCTCAATATGGCCAGTCTTTGGTATGTCTCCTGGCTCAGCATTTTCTACTGTGTGAAGGTCATCAACTTTTCCAACTCTTTACTCCTTTGGCTCAAGTTAAGGATCAATCTGCTTCTACCCAAACTACTTGGAATATCAGTGGTCATTTTCATGGTCTCTTCTCTTCCTTCCATCTTCACATTTCATAAATGCAATGAACCATGTAATCAGACTGTAACACCCCTAATTAATGAGGAAGCTGACAACATGTGGATTAGTTATTTCCCAGTGGAGATAACATTTACTTGCATAAATTACAGCGTGAACATAGCAGCAACCCTTCTTTTGCTCATCTCCTTGTGGAGGCATGTGAGAAACCTCAGAAAGAATGGTACTAGTGTTCAGGACCTCAACACTCAGGTCCACCTCAAAGTCATAAGGCTTTGTTGATCACTCTCTTACTCTACCTTTTATATATTGCTAGTTTGATATTAATGGATACTGGCTTTTTTTATTTTCAAGCAAACCTATCACTGATTGGAGAGATAATGGTTACCATATTTCCTTCAGTGCATGCCATAATATTAATATGGACCAATCCAAAACTCAGAGAAGTGGCTGCTCACATGC

>Lizard_T2R39--Pseudo

ATTATAGAAGGTATATCCATGGTTGCCATTTTAGGAAATGGATTTATCATAGTTGTGAGTGGGAACCGATGGCTCCAAGCCAGGAAGATGGTCCCTTCTGATTTTCTCTTGACTAGTTTGAGTATTTCCAGAGTGTTTTTGCATGTAACCTTTGGACTTATCTATGTTTTGGAAGTCAGCATTGGTGAAACCTTTATGTATACTTTTGCATGGGAAACTATAAGCTTTGTCTGGGTATTTTCTAACATGGCCAGCTTCTGGTGTGCTTCATGGCTTAGTGTTTTCTACTGTGTGAAGGTCACCAACTTTGCCAACCGCTTTTTACTCTGGTTCAAGCCAAGGATCAATGTGCTCTCAGTTAGACTACTTGGAATGTCAATAAGTAGTCTTGTGTTCATGTCCATTCCCTTCTTCCAGAGTTACGCTGAAGGCAGTGCAATCTGACAAAGAATCTGCAAGTGAATGTCAGCAAAATAGAGGTTTGCAGACGTTTTCAGTTAATTGTTGTTTTGATGAATTTCATCATCAGCATGATTGCAACCATTCTTTTGCTCACCTCTTTGTGGAAACACATAAGGAATCTGAAAAAGAGTGAAATTGGTGCAAAAGACCTAAGTGCTCAGGTCCATATTAATGTCATGAAGCCTTTGGTGTTTTATATTTTCCTCTACCTTTCATATTTTGCCGGTGTGCTAAATTTTGCAAGTCATTCTGTGCACAATGTTGATGCTATGGAGCGTTTGTCTGACATCCTTCTTACCATATTTCCTGCAACACACACCATAATATTACTTTTGAGCAATCCAAAACTGAAAGCATTGTTAGTTCGCACTCTAAATACAAGAC

>Lizard_T2R40--Pseudo

CAAATGTCTCCATTTGCTATCTTCTCCTGTTGCATTATAGGCATTTTGTGCATTATTTCCCTTTCTGGGAATGGATTTATCTTCATTGTGACTGTGCTGCAATGGCTCCAGAAGAGGAAGATGCCACCTTGTGACTTCCTCCTGACTTGCTTGAGTGCCTCCAGATTGCTAACACAGTTTAATTATATGGCCAGCTATTTTTTGCCTTTCTTTTATTCGCCAAGTATAAGAAAAATGTTTTTTTTCTCCAGGGTCTTTCTTCATATGGCCAGTCTTTGGTGTGTCTCCTGGCTCAGCATTTTCTACTGTGTGAAAGTCATCAACTTTTCCAACTCTTTACTCCTTTGGCTAAAGTTAAGGATCAATCTGCTTGTACCCAAACTACTTGGAATATCAATGGTCATTTTCATGGTCTTTTCTCTTCCTTTCCATCTTCACATTTCATAAATTCAATAAATCATGTAATCAGACGATAACACCACCAACCCAGCCATGAACCTGAAGATAGCATGTGGATTCGTTTTTTTCCAGTGCAGATAACTTTCACTTGCATAAATTTCAGCATGAACATAGCAGCAACCCTTCTTTTGCTCATCTCTTTGTGGAGACATGTGAGAAACCTCAGAAAGAGTGGAACTAGTGTTCAGGACCTCAACACTCAGGTCCACCTCAAAGTCATGAGGCCTTTGTTCATCACTCTCTTACTCTACCTTTTATTTATTGCTAGTTCGATAACAATGACAACTGCCTTTTTTCATGTTCAAGAAAACCAAGCACTGATTTCAGAGATAATGATTTCCATATTTCCTTCAGTGCATCCCATAATATTAATATGGACCAATCCAAAACTCAAAGATGTGGCTGCTCACATGT

>Lizard_T2R41--Pseudo

TCAGGGAATGGATTTATCTTCATGGTGACTGTGCTGCAATGGCTTCAGAAGAGGAAGATGCCACCTTGTGACTTCCTCCTGACCTGTTTGAGCACCTCCAGATTGCTAACACAGTTGAATTCTATGGCCAGCTATTTTATGCATCTCTTTTATTTGTCAGGTATAAGAAAAATGGTTTTTTTCTCTGGGGTCTTTCTCAATACGGCCAGTCTCTGATGTGTCTCCTGGCTCAGCATTTTCTACTGTGTGAAGGTCATCAACTTTTCCAACTCTTTACTCCTTTGGCTAAAGATGAGGATCAATCTGCTTCTACCCAGACTACTCGGAATATCAATGGTCATTTCCATGGTCTTTTATCTTCCTTCCATCTTCACATTTCATGAATGCAACAAATCATGTAATCAGACGATAACACCACCAACCAACCATGATGCTGAAGATAGCATGTGGATTCGTTTTTTTCCAGTGCAGATAACTTTTACTTGCATAAATTTCAGCATGAACATAGCAGCAACCCTTCTTTTGCTCATCTCCTTGTGGAGGCATGTGAGAAATCTCAGAAAGAACGGTACTAGTGTTCGGGACCTCAACACTCAGGTCCACCTCAAAGTCATGAGGCCTTTGTTGATCACTCTCTTACTCTATCTTTTATTTATTGCTAGTTTGATAACTATGGCAACTGGCTTTTTTCAGTTTCAAACACATCAATCACTGATGGCAGAGATAATGATTTCCATATTTCCTTCAGTGCATCCCATAATATTAATATGGACCAATCCTAAACTCAAAGACGCGGCTGCTCACATATTA

>Lizard_T2R42--Pseudo

ATTGGTATTTTGGAAAATGGATTCATTATAGTTCTGAATGGACACCAGCGATTCCAAAACAGGAAGATGATTCTTTGTGATTTCCTTCTGACCAGCCTGAGTACTTTTAGGTTTATATGCAGTTGGGTTTTCTGATAAACTACATTTTGTACTTCACTCTAAAGATTGATTTATATCTTTTCTTAAAGGATGTTATGTTCTTTTCCTGGATGTTTTCCAACTCAATCAGCCACTGGTGTGCCACATGGCTCAGTATTTTCTATTGTGTAAAGGTCGCCAACTTTGCCAACACCCTCTTCCTCTGGCTGAAAGTAAAGATCAATATGCTTGCACCCATACTGCTTGGACTATCCATAGCCGCTTTCACAGTCTCTTTAATTTCTTTGGTTGTTGATTATTTTGGACAGACAAAGAGGTGCAATCTGACAGAGATTCTGCCAGAGAATGCCAGTCATTTAGATGTTTATGGCATTGCTCCCATTATTTTTCTTCCTATGCAATTTTATTTTTATGTCATAAATTTGTGCTTAAGCGCAATCACATCTATTTTTTGCTCGTCTTTCTGTGGAGACACACAAGGTGTTAAGGACCTCAGCACTCAGGTCCACATTAAAGTCATTGCATTTTTGTTGTTCTGGATCTTCTACTTTGTAGATTTCATTGCTCTGTTTATTTATTCTAACCTCATTAATAATATCGGGAGAGTTCAAGGGCTGCTTTTTGGTATCTGGATGTCTGCGTTTCCTTCTGCACACTCCATTATATTAATAATAACCAATTCTAAACGGAAAGAAATGTATATTTACATCATAA

>Lizard_T2R43--Pseudo

AGTATATCCATGGTTTCCATTTTAGGAAATGGATTTATCATAGTTGTGAGTGGGAACCGATGGCTCCAAAACAGGAAGATGGCCGCTTCTGATTTACTCTTGACTAGTTTGAGTATCTCCAGAGTTTGTTTGCATGTAACCTTTGGACTTTTCTATGTTTTAAAAGTCAGCATTGGTGATGCCTATATGGGTACTTCTGCCTATGATGCTATCATCTTTGCCTGTATGTTTTCTACCTTGGCCAGCCTCTGGTGTGCTTCATGGCTTAGTGTTTTCTATTGTGTGAAGGTCACCAACTTTGCCAACCGCTTTTTACTCTGGTTGAAGCCAAGGATCAATGTGCTCTCAATTAGACTGCTTGGAATGTCAGTGATTAGTCTTGTGGTCATCTCTGTTCCCTTCTTCTGGAGTTACGCTGAAGAAAAAAAGCGGTGCAATCTGACAGGGAGCCTGCCAGTGAACATCAGCAAAAGATGCCAAGCCTCACTTTTTATTTTTCATCCTCTTCATTTAAGTGTTGCTTCCATGAATTTCATCATCACCATAACTGCAAATGTTCTTTTGATCATCTCTTTGTGGAAACACACGCAGAATCTGAAAAAGAGTGGTATTCTTGCAAAAGACCTAAGTACTCAAATCCATATTACTATCATGAAGCCTTTGGTGTGTTATATTTTGCTCTGCTTTTATTTTTCACAGGTATGCTGTTTTTATCAGGTAGTTTTGTGTACACTTTTGATGCTAAGAATTTTTTATCTGACATCATTTTTACCACATTTCCTTCAGCACACACCATAATATTAATCTTGACTAATCCAAAACTGAAAGCACTGTTAATTCGTACTT

>Lizard_T2R44--Pseudo

ACTAGCGACAATATAGTTAAAGTTGATATCCTTGTTTGGGTTATTTTTGGAACTGTGTCTCTTATTGGTATTTTGGGAAATGGATTCATTATGGTGGTGAACGGACTCCAATGGCTTCAAAACAGAAAGATAATCCTTTGTGATTTTCTCCTGACCAGCGCAAGTACTTTCAGATTTATCATGCAGTTGGTTCTTCTGCTATACAACATCCTGTACTACTTCCCAGAGAATATTCCCTGTATTTACAGAATAAATCTTATGTTCTTTTGCTGGATTTTTTTCCAACACGATCAGCCACTGGTGTGCAACATGGTTCAATGTTTTCTACTGTGTGAAAGTCACCAACTTTGCCAACACCCTCTTCCTCTGGCTGAAAACAAGGATCAATATGCTTGTACCCAGGCTGCTTGGATTGTCCATAGCAGTTTTCATAGTCTCTTGTCTTCCTTCAATTGCTGATTATTCTGGACAAACAAAGTGATGCAATCTGACAGAAATTTTGCCGGAGAATAACAGCCAAAGAGAGATTTGTAGCACTCCTGGCATGACTTTTCTTCCCATTCAGTTCTCTGCTTATGTCATAAATTTGTGTTTAAGCACAATTACATCCATTCTTTTGCTTGCCTCTCTGTGGAAACACACAAGAAATCTCAAGAAAAGTGGTGTTGGTGTTAAGGACCTCAGCACTCAGGTCCACATTAAAGTCATGACATTTTTGTTGCTCTGGCTCTTCTTCTACCTTTTAGATTTCATTGGTCTGATAGTTTATACTAACATCGTTCTTAATACTTTAAAATTGAATGGAATAGCTTGTTGACATCTTAATGTCTGCATTTTCTTCCGCTCACCCCATTATATTAATATTAACCAATCCTAAAC

>Lizard_T2R45--Pseudo

ATGTCTATGGCTGAAATCATTTTTTTGATCATTTTTGAAATTGTGTCTTTTATTGGTATTTTGGAAAATGGATTCATTATAGTTGTGAATGGACACCAGCGGTTCCAAAACAGGAAGATGATTCCTTGTGATTTCCTCCTGACCAGCCTGAGTACATCTAGATTTATTATGCAGTTGGGTTTTCTGATATTCCACATTTTGTACTTCAGTCTAAAGATTAATTTACAGCTTTTTTAGGAGATGTCATTTTCTTTTGGTGGATGTTTTTCAACACGATCAGCAACTGGTGTGCCACATGGCTCAGTGTTTTCTATTGTGTAAAGGTCGCCAACTTTGCCAACCCCCTCTTCCTCTGGCTGAAAGCAAGGATCAATATGCTTGCACCCATACTGCTTGAACTGTCCATAGCCGTTTTCATGGTTTCTTTTCTTCTTTCACTTGTTGATTATTTTGTTCACACAAAGTGGTGCAACGTGACAGAAACTCTGCCGGAGAACGCCAGCCAAATAGGGGTTTGTTTCATGGCTCCCATTATTTTTCTTCCCATGCAATTTTCTTTTTATGTCATAACTTTGTGCCTAAGCACAATTGCAACCATTCTTTTGCTCATCTCTCTGTGGAGACACACAAAAAATCTCAAGAAAAGTGGTGTTGATATTAAGGACCTCAGCACTCAGGTCCACATTAAAGTCATGGCGTTTTTGTTGTTCTGGTTCTTCTTCTACTTCATAGATTTCATTGCTCTGATACTTTATGCTGACCTCATTAATAATATGAATAATGCCGGGAAAGTTCAAGGGCTACTTTTTGGTATCTGGATGTCTGCAGTTCCTTCTGCACACTCTATTATATTAATAATGACCAATCCTAAAC

>Lizard_T2R46--Pseudo

ATCTTTGTCTTTTTGCTAGCTGTAGCTGACTTGGGTTTTGGTGGACTCATCTCCAATGGCTTTATAGCTACAGTGACTATCAGGGAATGGACCAAACACAGAAGACTTGCTTCCAGTGAACAGCTCTTTCTGAGTCTGGCTCTGTCCAATATTTGCAGCACCATTTTAACAATTCTATATTTCTGGGGTACGTATGTCACCAATAACTTACTATTATTCAAAATATTTCCTGCTACATTTTTTGCCGCTGTGACCAGATTCTGGTTCACTGCCTGGCTCTGTGTCTTCTATTGCATCAAGATAGTGAATAGCACACATGCCCTCTTCCTTTGGTGCAAGATGAGGATATCATGGCTAATACCCCGGCTTCTTATAGGATCTGTGGCCCTCTCCTTCTTTGTTTCCTGCATTTCAGTAAAGATATTTCCTACGGAATTCCAAAGCAACACAACAGGAAATGTTACAAGCTACACTCAAGTAAAGTTTAAAGACTATCTGGGCTTTTTTCGTCTTTTATTTTTAATTATTGGTTCCAGCAGTCCCCTTATTGTGGTTTTATTTTGCTCCATCATGGTTATTGCTTCACTCTCTAGGCATGTCTGTCGGGATGGCAAGTACCACGTCCAGTTTTAAGGATCTCCAAACAGAAGCTCATATTAAAGCAGTTTGGATAGTGCTCTTCCTTTTATTCCTTTATGTTTCCTATTTTATGGCACATACCTGGTCTATAGTTGTAGAAATGGCGACTATGGAAATGTCTTTTGCTTCATTTGTGCTGATAACGTATTCCCCTGCTCAAGCTGCTGTACTGATTTTCACTAACCCAAAACTGAGGGAGGCATTTAATCA

>Lizard_T2R47--Pseudo

GGGAATGGATTTTTCTTCATGGTGACTGTGCTGCAATGGCTCCAGAAGAGGAAGATGCCACCTTGTGAATTCCTCCTGACCTGTTTGAGTGCCTCCAGATTACTAACAGAGTTGGATTGCATGGCCATCTATTTTATGGAAGAACAATACTCTATTTCTCCTGGATCTTTTTCACTATGGCCAGTCTCTGGTGTACCTCCTGGCTCAGCATTTTCTACTGTGTGAAGGTCATCAACTTTTCCAACTCTTTACTCCTTTGGCTAAAGTTAAGGCTCAATCTGCTTCTACCCAGACTACTTGGAATATCAATGACCATTTTCATGGTGTCTT

>Macaka_T2R27--Pseudo

GTGAGTGTATGTGTGTCATTTGTCCTTGGGAATGTAGCCAACAGCATCACAGTTCTAGTAAATGTCACTGAGTGGGTTAAGACAAAAAAAGATCTCCTTAGCTGACCAAATTCTCACTGCTCTGGAGGTCTCCAGAATTAGTTTATTCTGGGACATATTAGTATATCAGTATGCAACTGTGTTTAATTTGGCATTATTTAGTTTAGAAGTAGGAATTGTTGCTTCTAATGTCTGGACAATAAGCAATCATTTCAGCCTCTGGTTGCTACTAGCCTCAGCATATTTTATTTTTCAAGATTGACAATTTCTCTTCCTAAAGAAGAGGATTAAGAATGTTGGTTTGAATGTTGGTTTGGTGATACTTTTGTGGCCCTTGGTATTTTTGATTTGTAATCTTGCTCTGGTTACCATGGATGAGAGTGTGTGGACAAAAGAATAAGAAGGGTATTTGTCTTGGATGATCGAATTGAGGAATGCAATACACCTTTCAATCATGGCTGTACCCACGCTAGCCAACTTCACACTCTTCATTCTGACGCTAATATCTTTTCTGCTGTTAATCTGTTCTCTGTGTAAACATCTCAAAAAGATGTAGCTCCATGACAAAGGATCTCAAGATCTCAGCACCAGGGTCCACATAAAAGCTTTGTAAACTGTGATCTCCTTCCTCATGTTATTCGACATTTAATTTCTGTATCTAATCACATCAACTTGGAATCCTAGGACACAGCAGAGCAAACTTGTATTACTGCTTTGCCAAACTCTTGCAATCATGTATCCTTCATTAGTCCTGATTCTGGGTAGTAGGAATCCAAAACAGACCTTTCTTTTCATTTTGTG

>Macaka_T2R28--Pseudo

ATGATAACTTTTCTGCCCATCACTTTTTCCATTCTAATAGTGGTTATATTTGTTATTGGAAATTTTGCTAATGGCTTCATAGCATTGATAAATTCCACTGAGTGGGTCAAGAGACAAAAGATCTCCTTTGCTGGCCAAATTCTCACTGCTCTGGCGGTCTCCAGAGTTGGTTTGCTCTGGGTATTATCACTACATTGGTATGCAACTGAGTTTAATCTAGCTTTTCATAGTGTAGAAGTAAGAAGTACTGCTTATAATGTCTGGGTAGTGACCAACCATTTCAGCAACTGGCTTGCTACTAGCCTCAGCATGTTTTATTTGCTCAGAATTGCCACTTTCTCCAACCTTATTTTCTTCACTTAAATAGGAGAGTAAAGAGTGTCATTCTGGTGACACTGTTAGGGCCTTTGCTGTTTTTGGTTTGTCATCTTTTTGTGATGAACATGAATCAGATTGTACGGACAAAAGAATATGAAGGAAACATGACTTGGAAGATCAAATTGAAGAGTGCAATGTACCTTTCAAATACAACTGTAACCATGCTAGCAAACTTTGTACCCCTCACTCTGACCCTGATATCTTTTCTGCTGTTAATCTGTTCTCTGTGTAAACATCTCAAGAAGATGTGGGTCCATGGCAAAGGATCTCAAGATCCCAGCACCAAGGTCCACACAAAAGCTTTGCAAATTGTGACCTGTTTTCTCCTGGTATGTGCCATTTACTTTCTGTCCATGATCCTATCAGTTTGGAATTCTGGGAGGCTGGAAAAGAAACCTTTCTTCATGTTCTGCCAAGCTATTGTATTCAGCTATCCTTCAACCCACCTATTCATCCTGATTTGGGGAAACAAGACACTAAAGCAGACTTTTCTTTCAGTTTTAAGGAATGTAAAGTACTGGGTGAAAGGACAGAAGCCTTCATCTC

>Macaka_T2R29--Pseudo

TTCTCGAAACTGATTATATTTACTGTCGGATCCTTTGTACCCTTTCCTCTATCCTTACATGTTTCTCTTGTTAATCTCCCTGTGGAAATATCTCAGACATATGAAGCTCAATGCAATGGAATTCAGAGATCCTAGAGTCAAGGCCAATAAAAGAGCCATGATATCTGTGATGCTTTTCTTCTTATTATTTGCTATTTACTTTCTGTCTCTTCTTACAGTGATTTTTCATTCTAAGGTGATACAGATCACATTGGTCCTTATGCTTACCCAGGCTATTGCAAGTGTTTACCCTTCAGGCCACTCATTTATCCTATT

>Macaka_T2R30--Pseudo

CTACCCATATTTTCCATTCTAGTAGTGGTTACGTTTGTTCTTGGAAATTTTGCTAATGACTTCATAGCATTGGTAAATTCCATTGAATGGGTCAACAGACAAAAGATCTCCTCTGCTGACCAAATTCTCACTGCTCTGGCAGTCTCCAGAGTTGGTTTGCTCTGGGTAATATTACTACTTTGGTATGCAACTGTTCTGAATCCAGATTCATACAGTTTAGCAGTAAGAATTACTACTACTAATGCCTGGGCAGTAACCAACCATTTCAGCATCTGGGTTGCTACTAGCCTCGGCATATTTTATTTGCTCAAGATTGCCAATTTCTCCAACTTTATTTTTCTTCACCTAAAAAGGAGGATTAAGAGTATCATTCCAGTGATACTACTCGGGTCTTTGTTATTTTTGGTTTGTCATCTTGTTGTGGTAAACATGGATGGGAGTATGTGGACAAAAGAACATGATGGCAATATTGAGTTGGGAGATCAAATTGAGTGATCCAATGCACCTTTCAGATATGACTGTAACCACGCTTGCAAACTTAATAACCTTTATTCTTTCCCTGATATCTTTTCTGTTGTTAATTTGTTCTTCGAGTAAACATCTCAAGAAGATGCAGCTCCATGGCAGAGGATCTCCAGATCCCAACACCAAGGTCCACATAAAAGCTTTGCAAACTGTGATCTCCTTCCTCTTGTTACTTGCTATTTACATCAATTTGGAATCTTAGGAGGAGGCTGCAGAAAGAACCTGTCCTCCTGCTCTGCCAAACTACTGCAATTATATATCCTTCGTTTCATTCATCCTAATTCGGGGAAGCAAGAAGCTAAAACAGACCTTTCTTTTGATTTT

>Macaka_T2R31--Pseudo

TTTTTCCATTCTAAGAGTGGTTATATATTTTTTATTGGAAATTTTGCTAATGGCTTCATAGCATTGGTAAATTCCATTGAGTGGGTCAAGAGACAAAAGATCTCATTTGCTGACCAAATTCTCGCTGCTCTGGCTGTCTTCAGAGTTGGTTTGCTCTGGGTATTATTACTACATTGGTGTGCAACTGAGTTTAATCTAGCTTTTTATAGTGTAGAAGTAAGAAGTACTGCTTATAACGTCTGGGTAGTGACCAACCATTTCAGCAACTGGCTTGGTACTAGCCTCAGCATGTTTTATTTGCTCAAGATTGCCACTTTCTCCAACCTGATTTTTCTTCACTTAAAGAGGAGAGTTATGAATGTCATTCTGGTGATGCTGTTGGGGTCTTTGCTATTTTTGGCTTGTCATCTTTTTGTCATAAACATGAATCAGATTGTACAGACAAAAGAATATGAAGGAAACATGACATGGAAGGTCAAATTGAGGAGTGCAATATACTTTTCAAATATGACTGTAACCATGCTAGCAAACTTTGTACCCCTCACTCTGACCCTGATATCTTTTCTGCTGTTAATCTGTTCTCTGTGTAAACATCTGAAGAAGATGCAGGTCCATGGCAAAGGATCTCAAGATCCCAGCACCAAGGTCCACATAAAAGCTTTGCAAACTGTGACCTCCCCCACCCATTCATCCTGATTTGGGGAAACAAGAAACTAAAGCAGATTTTTCTTTCAACTTTGTGGAATGTGAGGTACTGGGTGAAAGGACAGAAGCCTTCAT

>Macaka_T2R32--Pseudo

AATTTTGCTAATGGCTTTATAGCATTGGTAAATTCCACTGAGTGGGTCAAGAGACAAAAGATCTCCTTTGCTGACCAAATTCTCACTGCTCTGGCAGTCTCCAGAATTGGTTTGCTCTGGGTGTTATTATTACATTGGTATTCAATTGTGTTGAATCCAGCTTTTTATCGTGTAGAGGTAAGAATTACCACTTAAAATGTCTGGGCAGTAACCAGCCATTTGAGCAACTGGCTTGCTACTAGCCTCGGCATATTTTATTTGCTTAAGATTGCCAATTTCTCCAGCCTTATATTTCTTCACTTAAAGAGGAGAGTTAAGAGTGTCATTCTGGTGATGCTGTTGGGGCCTTGGTATTTTTGCCTTGTCATCTTTTTGTGATAAATATGAATGAGATTGTACAGACAAAAGAATATGAAGGAAACATGACTTGGAAGATCAAATTGAGGAGGACAATGTTCCTTTCAGATACGGCTATAACCATGGTAGCAAACTTAGTACCCTTTACTCTGACTCTGATATCTTTTCTGCTGTTAATCTGTTCTCTGTGTAAACATCTCAAGAAGATGCAGCTTCATGGCAGAGGATCTCAAGATCCCAATGCCAAGGTCCACATAAAAGCTTTGCAAACTGTGATTTCCTTCCTCTTGTTATGTGCCATTTACTTTGTGTTCGTAACCATATCAGTTTGGAGTTTTCAGACTCTGGATAATAACCCTGTCTTCATGTTCTGCCAAGCTATTACATTCAGCTATCCTTCAGCCCACCCATTCATCCTGATTTGGGGAAACAAGAAGCTAAAGCAGACTTTTCTTTCAGTTTTGTGGAATGTGAGGT

>Macaka_T2R33--Pseudo

TTGTCTGTCTTTTATTTTCTAAAGATCATTAATTTCTCCTACATCTTTTACCTTTGGCTAAAATGGAGAATTAACAAGGTAGCTTTCACACTTCCACTGGCGTCTGCCTTTTCTGTTTATGAACTTTCCTTTGCCGTACAGTTTTGATGTCTTCTGGTATCATGTCCAAAAAAATATGAAAGACATATGACTGGATTACTTGATGTAAGTAATAATAAAACTGTTAATGACATAATCATCTTCATTACTGGATCTCTCTCTTCTTTCTCTATTTCCTTGATTTTCTTTTTCTTGTTACTCCTTTCTTCGTGAAGACACATGAAACACATTAGGTTCAATGTCAGGGCTTCCAGAGACCCCAGTATACAGGCCCATTTCAGAGCCAAGAAAACTGTTTTCCTTTTGTGTCCTGTTGCTCTACAAAAATTTGCCCTTTTCTTGACATGTACAGGGGAATTTTTTGCTACAGAACAAACTGGTTGTGATATCTGGTTTATGATAGGAAATCAGTATCCTTTGGGTCACTCATATGTTGTGATTTTT

>Macaka_T2R34--Pseudo

ATGTTAGGGAATGGGTTCATTGTACTAGTTAACTGCATTAACTGGTGAGGAGTCAAATGATCTTATCAGCCGACTGCATCCTCACCAGCCTTGCTATCTCCAGAATCAGTCAACTTTGGATAATACTACTTGATTCATTTTTAATAGCATTATGGCCACATCTATATGCCTTCAACAAACTAGTAAAATTTATTGGTATTTTGGGGGCACTGACCAATCACTTAGTTACCTGGCTTGCTGCCTGTCTTAGTGTTTTCTACTTCTTTAAAATAGTCAATTTTTGCCACCCCTGCTTCATCTGGCTGAGATGGAGAATTAGCAGAGTGCTACTTGTACTCCCACTGGGGTCTTTGTTCCTACTGGTTTTCAACCTTGCATTAACAGGTGGACTTAGTGACTTGTGGATTAACAGCTACACAACTTATGAAAGAAACTCAAGTTGGTCTTTAGATGTAAGGAAAATTCTATATTGTAATGTCTGGATTCTTGCCAGTTTGATCTACTTAATTTCCTTTCTTCTGTCCCTGATCTCACCGCTCCTTTTAATTCTGTCCTTGATGAAACGTATCAGGAGTTTGCAGCTCAACACCATGGGCCCAAGGAATCTCAGAATGAAGGCCCATAAGAGGGCCGTGATGGTGTCTTTCCTCCTCTTCTTTTTGGTTCACTTTGCTTCTCTCCTACTAACAGGTTGGATTTTCCTTGTACAGCAGAAATAGCAGGTCAATTTCTTTGTCTTGTTGACATCAATTATTTTTCCTTCAAATCACTCATTTGTCCTAATTTTGGAAAACCACAAGCTGAGACAGACTGCTGTGGAACTACTGTGGCATCTTAAGTGCTACCTAAAACGAGTGAAAGCTTTAGTTT

>Macaka_T2R35--Pseudo

CTTAGTGTCTTCCATTTCTTCAAGGTAGCCAACTTCTCCAAACCCATTTTCCTCTGGATGAAATAGAGAATTCACAAGGTGCTTCTTTTTGTTGTACTAGAGGCAATGATCTCTTTCTGCACAACTTTCATTTTGAAAGAAATAATAAATAGTTTAATCCAAGAATGGGTAATAATAAAAGGCAACTTGACATTTAACTATATGGAGACCATGCATGATTTCACTTCTCTGTTTCTCCTTCAGAGGATGTTCATCCTTCCTTTTGTGGAAACACTGGCCTCCATTTTTCTCTTACTCCTGTCCTTATGGAGCCACACCAGACAGATGAAGCTACACGGTGTTTATTCCAGGGATCCCAGCAGAGAAGCCCATGTAAGACCTATAAAAGCTATAATTTCATTTCTACTCCTCTGCTGTGCATCAGTTCATCAGTATCATACTAACATTGTCTTATCCTATTCTAGACAGCGTTGTGGCAAGGACTTTTAGTAGTGTGCTGCTATTTTTCCATCCATCTGGCCATTCATGTCTTCTAATTTTACGTGACAGCAAACTGAAGCAAGCTTC

>Macaka_T2R36--Pseudo

GTTATTTCAATTACTGTAGTGGTCCTATTTGTTATTGGAAATTTTGCCAATGGGTTCATAGTATTGATACATTTTATTGGCTGGGCCAAGAGACAAAAGATCCCCTCAGCTGATCAAATTCTCACTGTTCTGTCAGTCTCCAGAGTTGGCTTCCTCTGGACAGTATTGTTACATTGGTATTCAACGGTGTTTAATCCAGCTTCATAGAGTTTAGAATTAAGAATTACTGTTTATAATGTCTGGACAGTAATCAACCATTTCAGCATCTGGCTTGCTAGTAGCCTCAGCATTTTTTATTTGCTCAAGATTGCCAATTTCTCCAACTTTGTTTTTCTTCACCTAAAAAGAGACTTAAGAGTGTCATTCCAGTGATAATGCTGGGGCCTTTGCTATTGTTGGTTTGTCATCTTGTGTTGGTAAACATGTATATGAATGTGTGGATAAAAGAATATGAAGGAAAAGTGACTTGGAAGATCAAATCGAGGGACACGATACATCTTTCAAACTTGACTGTATCCACGCTTGCAAACTTAATACCTTTTACCATGTCCCTGACATATTTTCTGCTGTTAATGTCTTCTCTGAGTAAACATCTCAAGAAGATGCAGCTTCATGGCAAAGGAGCTCAAGATCCCAGCACCAAGGTCCACATAAAAGCTTTGCAAACCG

>Macaka_T2R37--Pseudo

ATGTCAAGTGCACAGGACAACATCGTTTTGCTTATCTTAGCTGCGGAACTCATAATAGGTATTTGGGTAAATGGATTGATTGGAATAGTGAACTGCATTAACTGGGTAAGACACAGGAAAATCAGTGTCATTTACCTCATCCTCATCAGCTTATCTGTATCCAGAATATGTTTTTTGATTATGTTGCTAATTGATTCACTTTTACAAGTGCTCTCTTCCAATCGATATAGCACTGGTAAAATTAGAAAATTAATATTGTTTTGCATAGTAGTCAACCACTTAGGTGTCTGGCTTGACACCTGCCTCAGCATCTTCTGTTTCCTGAATATAGCTACATTTTTTTCATCCTCTTTTTCTTTGGTTGAAGTGCAGAATTAACAGGGTGGTTATTATTTTTTTTTCTGGGATCTTTGTTATCTTTCCTTATTAGTATCCCAGTGATAGACCACATAATTTCTGCTGTTTTAAACAGAGAAACAAACAAGACTTGAAATTTCAAAGTGTATGAAGAGCAGACCATTTACTTAGTGCTCATGAACACTGAGATCATAATTCCCATAACTATTTTGATAGCATCATTTATTTAGTTACTTATTTCTCTTTGGAGACATAGCAAGAGGATGAGGTTCTATATCACAGTGTCCCAAGATCCCAGCACTGAAGCTCATGTAAAAGCCATGAGAATGGTGACCTCTTTCTTGTTTCTCTTTTTGATTTATATCATCTCAATTCATATAGTAGTTTATGGTCACATATTGTCTGCTAGCCAGCTGATATTGAGGTTTAATAAGTTAATAGTATCTTTCTATGTTTTAGGCCATTCATTTGTTACAATTTCAGGAAACAGCAAGATAAAGCAAGCTTCTTTTAGGGTTTTGGGGC

>Mouse_T2R34--Pseudo

CAGGTTGTGGCTGCTATCATACGGATCATATTTACAACCATTTTCATTTTGGAATTTCTCTTTGGGAACCTTTTAAATGGATTCATAGCACTGGTGAATTTTGTTAACTGGGTTCAGAGAAGAATAATTTCTCCAGTGGGTCAAATAATCACTGCCCTTACAATTTCCCGACTTGTTCAGCTCTGGCTTGTACAAATAAATATATTGCTTTTCCTTATTTACCAAGTAAAAATGACAGAAATAGTTGTAAGAGCGATCAATATTGCCTGGGTTGTTACCAATCACTTTAATCTTTGGATATCTACCAAACTCAGTATGTTTTACCTTCTGAAGATAGTCAATTTTTCGAGTTCAATTTTTCTTTACTTAAAATAGAGAGCTAGGCATGTGGTTTCAGTAACCCTGATGCTGTCCCTTGCCTTCTTGGTTTTGAACATCATAGTCATCAATACCCATATTGATGTCTGGATTGAACTGAATGTAAGAATCATGCCTTATAATTTTAGTTCAAGGAACTCTACTTTATTTCTCAAACATCTTTTATTCACCAACTCTATATTTGCAAGCATCCCCTTTGCTGTATCACTGGTAGCTTTTTTCCTGTTAATTCTCTCTTTGTGGAAACATGAAAAGAAGATGCGTAACAATGTTGGGGCTTCCAGAGACTCCAACACCCAGGCCCACATTAAAGCCTTGAAGACTAGCCTTGCTTTCCTCTCTCTTTATGCCATATTTTTACCTTCTCTTGTTATAAATGTTTGCAGCATCGAATTACAGGAGAATTATATGATTTTTTTATTTAGCCTATTTACTGGAATAGCTTTTTCTTCATTCCACTCCTATATCCTGATTCTGGGAAACAATAAATTGAAACAGGCTTCTCTTTCTGTGCTGTGGTGGATGAGGTGCAGGCCCAAAGATGTA

>Mouse_T2R35--Pseudo

ATGCAGCATCTTTTAAAGATAATATTTGCTATCTCCCACAGCACTCTTGCAATGATTTTCATCATGGAATTAGTAACTGAAATTATAGGAAATGGTTTGATGGCCCTGGTGCACTATATGGACTGGGTAAGGAAAAAGAAAATGCCATTAGTTAATCAAATCTTCATCACTTTTTCAGTCTCCAGAATTTTTCATCTCAGTTTATTGTTTATAAGTTTAGTCATCTTCTTTTCATATCCAGATGCAATTATAACTTCCAGGATGATACATGTTATTAATAATGTTTGGATTATAGTAAACAATTTCTCCATCTGGCTTGCTACCTGCCTCAAAGTCCTTTATTTTCTCAAGATATACGATTTTTCTAACTCTCTTTTTCTTTGTCTAAAGTGGAGAGTTGAAGAAGTAGTTTCAGCAACACTGCTGGTGTCACTGGTCCTCCAGATTTTAAATATTTTACTATGTAACTTGGAAATTAACATATGCATTAATGAATATAAAAGAAACATATCTTGCAACTTCAGTCCTCATTACCAAGCAAATTGTCAAAGTAACGTGTTAAGCTTTCACATTATTTTCCTGTCTGTCCCCTTTGTTTTATCCTTGTCGATGTTTTTTTCTGCTCATTTTCTCCCTGTGGACACTTCACAAGAGGATGCAGCAGCATGTTCAGGGAGGCAAAGATGCCAGAACCATGGCCCACTTCAAAGCCTTGAAAACCATGATTGCCTTTCTCCTACTATACTCTATTTTTATTCTGTCTGTCTTAGTACTTACTAATTTGGAAAAATGAAATCTTGAAGAAAAATCTTTTCATTGTATTTTGTCAGGTTGTATATATAGTTTTTCCTTCATTCCATTCATATGTCTTGATTTGGGAAGACAGGAAGCTGAGACAGGCCTGTCCCTCTGTATTGTGGCATCTGAAATGTAGATTAAATTATCTAGAAACTT

>Mouse_T2R36--Pseudo

ATGGAGCATCCTTTGAGGAGAACATTTGATTTCTCCCAGAGCATACTTCTAACCATTTTATTCATTGAATTAATAATTGGACTTATAAGAAATGGATTAATGGTATTGGTGCACTGCATAGATTGGGTTAAGAGAAAAAAATTTCATTTGTTAATCAAATCCTCACCACTTTGGCAAACTTCCAGAATTTGTCTGCTCTGGTTCATGCTAATACATCTCCTGATTACTTTATTGTATGCAGATTTAGCTAGTACTAGAACGATGATGCAATTCGCTAGCAATCCATGGACTATATCTAACCATATCAGCATCTGGCTTGCTACATGCCTTGGTGTCTTTTATTTTCTCAAGATAGCCAATTTTTCTAACTCTACTTTTCTCTATCTAAAATGGCGAGTTCAGTTCCTCTTGTTAAATATTTTACTGGTTAAATTTGAGATTAACATGTGGATAAATGAATATCATCAAATAAACATACCATACAGCTTCATTTCTTATTACCAAATTGTCAAATACAGGTGTTAAGTCTTCACATTATTTTCCTGTCTGTCCCCTTTATTTTGTCCCTGTCAACTTTTCTCCTGCTCATCTTCTCCCTGTGGACACATCACAAGAGGATGCAGCAGCATGTTCAAGGATACAGAGATGCCAGCACAATGGCCCACTTCAAAGCCTTGCAAGCAGTGATTGCCTTTCTCTTAATACACTCCATTTTTATCCTGTCACTGTTACTACAACTTTGGAAACATGAATTAAGGAAGAAACCTCCTTTTGTTGTATTTTGTCAGGTTGCATATATAGCTTTTCCTTCATCCCATTCATATGTCTTCATTCTGGGAGACAGAAAGCTGAGACAGGCTTGTCTCTCTGTGTTGTGGAGGCTGAAATGCAGGCCAAATTATG

>Mouse_T2R37--Pseudo

ATAGTTAATGTGGATTTCCTAATTGGAAATGTTGGGAATGGATTCATTGTTGTGGCAAACATAATGGACTTGGTCAAGAGAAGAAAGCTTTCTTCAGTGGATCAGCTGCTCACTGCACTGGCCGTCTCCAGAATCACTTTGCTGTGGTACCTGTACATAATGAAACGAACATTTTTAGTGGATCCAAACATTGGTGCAATTATGCAATCAACAAGACTGACTAATGTTATCTGGATAATTTCTAACCATTTTAGTATATGGCTGGCCACCACCCTCAGCATCTTTTATTTTCTCAAGATAGCAAATTTTTCTAACTCTATTTTCTGTTACCTGAGGTGGAGATTTGAAAAGGTGATTTTGATGGCATTGCTGGTGTCCCTGGTCCTCTTGTTTATAGATATTTTAGTAACAAACATGTACATTAATATTTGGACTGATGAATTCAAAGGAAATGTATCTGACAGTTACAAATTAAAGATTTTTTTTATAGGTTTCCAGACTTCTTGTGTTAACAAATACTATGTTCACATTTGTACCCTTCACTGTATCTATAATAATATTTTTTCTGCTTATCTTCTCCCTGTGGAAACATCTGAGGATGGTGAAGCACATTGCCCAAAGCTCCCAAAATGCCAGCACCACAGCCCACATCAATGCTTTGAGAACTGTAATTGTCTTCCTCCTCTTGTATGTCATTTTTATTTTATCCCTCTTTGCACATCTTTGGAGCTTTGAGTTTGGAGAAAAGACATATTTTATTTTCTTTTGCCTTGTTGGTATTTTTGCATTACCATCACTCCATTCATGCATCTTGATTCTGGGAAACAGTAAATTGAGGGAGATCTCTCTTTTGGTACTGTCACTGTTAAAGTGCAATATACAAGGATGTGAATCCCTGGGTCCCTGGCACACTAGGGAGGATAC

>Mouse_T2R38--Pseudo

ATGGTCATTTTCATTGCAGAATCCACATCTGGATGCTTAGGAAATGGATTCATAACAGTGTTGGAAATCGTGAACTGGATAAAAAAGAAAGATATCTTCAGTGGACTAGATCCTCATTGCTCTGGCCCTCTGGAGATTCATTGTATTCTGGCTTGCATTCAGATTTGTTGATTAGTGAGCCTATGGTCAAGATAACTACTGTTTTATGGGCAGTGACCAATCATTTCAACATCTGGCTTCCTACTTGCCTCAGCATCTTTTATTTTCCCCAATTGACAATTTTTTCCAACTCTTTCTTTCTTCACCTAAAGTGGAGAGTTAAAAAAGTGG

>Mouse_T2R39--Pseudo

ATGCAGCATCTTTTAAAGACAATATTTGTTATCTGCCATAGCACACTTGCAATCATTTTAATCTTTGAATTAATAATTGGAATTTTAGGAAATGGGTTCATGGCCCTGGTGCACTGTATGGACTGGGTTAAGAGAAAGAAAATGTCCTTAGTTAATAAAATCCTCACTGCTTTGGCAATCTCCAGAATTTTTCATCTCAGTTTATTGCTTATAAGTTTAGTCATATTCTTTTCATATTCTGATATTCCTATGACTTCAAGGATGACACAAGTCAGTAATAATGTTTGGATTATAGTCAATCATTTCAGTATCTGGCTTTCTACATGCCTCAGTGTCCTTTATTTTCTCAAGATATCCAATTTTTCTAACTCTTTTTTTCTTTATCTAAAGTGGAGAGTTGAAAAAGTAGTTTCAGTTACACTGTTGGTGTCATTGCTCCTCCTGCTTGGAAATTAGCATATGCATAAAGGAATGTCAAAGAAACATATCATGCAGCTTCAGTTCTCATTACTATGCAAAGTGTCACAGGCAGGTGTTAAGGCTTCACATTATTTTCCTGTCTGTCCCCGTTGTTTTGTCCCTGTCAACTTTTCTCCTGCTCATCTTCTCCCTGTGGACACTTCACCAGAGGATGCAGCAGCATGTTCAGGGAGGCAGAGATGCCAGAACCACGGCCCACTTCAAAGCCCTACAAACTGTGATTGCATTTTTCCTACTATATTCCATTTTTATTCTGTCTGTCTTAATACAAATATGAATTACTGAAGAAAAATCTTTTCGTTGTATTTTGTGAGGTTGTATATATAGCTTTTCCGACATTCCATTCATATATTCTGATTGTAGGAGACATGAAGCTGAGACAGGCCTGCCTGCCTCTCTGTA

>Mouse_T2R40--Pseudo

TTGACATTGCTGCCATATTTGCTCATCATTTTCTTAAACATGTTAGTCATAAGCAGATATATTGATGTCTGGATTGATGGATATGGGCAAATATGTCCTATAGTGCTATATCAAGAAACTCTACCCAATTTTCTAGGCTGGTTTGACACATCAACACTATGTTCATAACCACACCATTCACTGTGTTTCTGATAATGTTTACTTTGCTCATCTTTTCCTTTAGGAAACATCTGAAGAACACCCAGCACAATGTCAAAGGCTCCAGAGAAGTCAAAACTGCAGCCCACATAAAGACTCTGCAAATGGCGGTCACCTCATGTTACTACATATGGCTTTTGTCAC

>Mouse_T2R41--Pseudo

ATGGAGCATCCTTTGAGGAGAACATTTGATTTCTCCCAGAGCATACTTCTAACCATTTTATTCATTGAATTAATAATTGGACTTATAAGAAATGGATTAATGGTATTGGTGCACTGCATAGATTGGGTTAAGAGAAAAAAATTTCATTTGTTAATCAAATCCTCACCACTTTGGCAAACTTCCAGAATTTGTCTGCTCTGGTTCATGCTAATACATCTCCTGATTACTTTATTGTATGCAGATTTAGCTAGTACTAGAACGATGATGCAATTCGCTAGCAATCCATGGACTATATCTAACCATATCAGCATCTGGCTTGCTACATGCCTTGGTGTCTTTTATTTTCTCAAGATAGCCAATTTTTCTAACTCTACTTTTCTCTATCTAAAATGGCGAGTTCAGTTCCTCTTGTTAAATATTTTACTGGTTAAATTTGAGATTAACATGTGGATAAATGAATATCATCAAATAAACATACCATACAGCTTCATTTCTTATTACCAAATTGTCAAATACAGGTGTTAAGTCTTCACATTATTTTCCTGTCTGTCCCCTTTATTTTGTCCCTGTCAACTTTTCTCCTGCTCATCTTCTCCCTGTGGACACTTCACCAGAGGATGCAGCAGCATGTTCAAGGATACAGAGATGCCAGCACAATGGCCCACTTCAAAGCCTTGCAAGCAGTGATTGCCTTTCTCTTAATACACTCCATTTTTATCCTGTCACTGTTACTACAACTTTGGAAACATGAATTAAGGAAGAAACCTCCTTTTGTTGTATTTTGTCAGGTTGCATATATAGCTTTTCCTTCATCCCATTCATATGTCTTCATTCTGGGAGACAGAAAGCTGAGACAGGCTTGTCTCTCTGTGTTGTGGAGGCTGAAATGCAGGCCAAATTATG

>Mouse_T2R42--Pseudo

ATAGTGGGAAGTTTAGGCAATGCATTCATAGTTCTGGTGAACATCATGGACTAGAAGCTATCTTTTATAGATCAGATCTTCACTGCTCTGGCAATTTCCAGAATTGGTTTCCTCTGCTCAATAATTACAAGTGTATTGGTATATGAACTGTATCCAGCTTTAATAATGACTAAAAGACTGGTGAGCAAAATTAATACATCTTGGACAGTTACCAATCATTTCAGCATCTGGCTTGCTACAAGTCTCAGCGTCTTTTATTTTCTCAAAATAGGCAATTATTTCAAACGTGATGTTTCCCTTAAATTGAGAGTAAAACAAGTGGTATCTGTGACATTGCTGGCATCTTTGCTTATCTTCTTTTCAAATATTTTAATCGTAAACAGATGTATTGATGTTTGAATTGATGGACATGAAGCTAATATATCCTACAATGCTATCTTAAATACTGCTACTCATATTTCTAGGCTTATTATACTCATCAACACTATGTTCACACTTATACCATTCACTGTATCTCTGACAATGTTTCTCCTTCTCATTTTCTTGCTATGGAGACTTCTGAAGAACATGCAGGGCAATGCCACAGGCTTCAGAGACATCAGCACTGCAGCCCACATAAAGGCCCTGAAAATGGTGGTCACCTTTCTGTCAATGTATACCTTTTTTTTTTCCTGTCGATCCTTTTGCAGTTTTGTAACATTAAATTTCAGAAGAAAAATTCAGTTACTCTATTTTCCTTCGCTACCAGACTTGCCTTTCCTTCAGGCCACTCTTATATCCTGATTCTGGGAAACACTAAGCTTAGGCAGGCTGTTCTTTCCATGGCATGGTGGCTGAGGTGCAGGCTCAGTGATGCAG

>Opossum_T2R27--Pseudo

ATGCTAGATGTACCAGAGAATATATGTCTGATCGTGCTGGCTGGGGAAACTATAATGGGGACTTTGATCAATGTGTTCATTGGCCTGGTGAACTGCATAGACTGGGTGAAAAGTAGGACGATATCTTTGGTGGATTTCATCTTCTTGAGTCTGGCCTTCTCTCGAATCTGTCTGCTGTTGATTATGCTAGCCAGTGGGATGCTGGGGCTACTTTACAGGGACATATATGAAACCAGGGAAACCTTGCTAATCCTCAGTCATCTGTGGATCATAGCCAGCTACTCCAATATCTCCTTTGTCACCTGCCTCAGCGTTTTCTACTTCCTGAAGTATTGCCCAACTTCTTCCCATCCCCTCTTCTTCTGGCTCCAAGTAGAAGATGAACAGAAGTGGTTTCTCATGATCTTGCTGGAGTCTTTGACTAAATGTTCTTGTTTAATGAAGTCTTCTCCCGAGAAAGGAGTTCAACAGATAAGTTTCATTAAGAGCACAGAAAGAAAAGAGGAGAAAAACCCACACTGAGTTTTTCCAGATGAGGAAGACCCACTTCAAGGTTATGCATAATCTATTCTATCTCGGGCCTCTCATCCTCTTGATTATTTCTCTGCTATCCTGTGTGCTGCTGGTCTTGTCCCTGTGGAGACATACCTGGCAGATGCATCTCAATGCCACTGGCTCCAGGGATCCCAGCACAGAGGCACATAGAAGAGTCATTATATGGATGCTCTCCTTCCTCTTTCTCCTCATTCTCTACTACGTGGGTGTTTTCACAAGTATCTCAGCCTCTTCTATGCTGGAGAAAAGGATGGTAGAAGTGCTGGGAGTTGTCATAGCAGGTGTCTATCCCTCAGGCCATTCTTTCATCCTGATTCTGGGAAACAGCAAGCTAAGGCAGTCATTCCTATGGGTGTGGCGGCAAGCCCTGCTCCGCCTCAGAGGAGG

>Opossum_T2R28--Pseudo

AAAATCTTATTGGTTCTGGTAGCTGGTGAGTTCCTAATGGGGCTTCTTGGGAATGGCTTCACAGCAGTAGTGAACTGTATTAACTGGATCAAAGATAAGAAATTATCAATGGTGGACATCATCCTCATCAGCTTGGCTATCTCCAGAATCTTTCTACTCTGCTTAACATCAATGATCATCTTTGACTATTTCTCATTTTCACATGGCTTAGTGATAAGTGAGATGACCCATATTCGTATCTTGTGGATATTGAGCCACTTCTCAAGTGTTTGGTTTGCTTTTTGCCTCAGCATCTTCTACTGCCTTAAGATTGCTAACTTCTCCCACTCCATCTTCCTCTGGCTAAAGTGGAGAGTCAGTAGGGTGATCTTCATATTGCATGGGGAACATTTTCTCATTGCCTTGCCTACTTTATCTGTATTCTTGTGGAAATTTTATGCTAACTATAGGAATCCGTTTTTTCTTGGAAGCAAGAGAAATTTATCCCAAGAAGACTCAAGGAGTGAAAATCAATCACTCATTTTCCAAATTTCCTTTAACCTAATATCTCTTCTCCCCTTTGCTCTGTCTTTGATATCCTGTTTCCTGCTAGTACTGTCACTATGGAGGCACAATCAGCAGATGCAACGCAATCCACGGGATCCAGAGCAGCATGGAGGTCCATATTCAAGCCATGAAAATCATATTTTCTTTCCTCATCCTTTTTTTGCTGTACTATCTAGGCATCTTCATTGGTCAAGCAAGTCTCAGGATGTCTGAGAAAAATGTGGCTTCTCTGTTTAGTTTAGCTATTATGGATCTGTATCCTTCAGGCCACTCATTGATCCTGATTATGGGGAACAACAACCTGAGAAAAACCGTCCTGAGGATGTTCAAACAGATGAAGGGCTACTTGAAAGG

>Opossum_T2R29--Pseudo

GAAATTTTGCTGGTCTTGGTAGTCGGAGAGTTTCTAATGGGGATTCTCATGTATCCTTTTTGGCATTGGTGAGCCGAATTGACTGGGCCAAGAACAAAAATATCTCAGCAGTTGATCTTCTTCTCCTGGTCAGCTTTGGCATGTCTTGTATCATTCTGTTATGCACAATGAAATAAGATAGATTTCAAATCATGCTCTGGACACTAAAGATGGTTGATCTCTTTTGGATAATGGCCCCACTATTCAAGTGTTTGGTTCACTACCTGTCTTAGCATCTTCTACTTATCAATGATCACCAACTTCTCCCATTCCTTGTTCCTCTGGTAAAATTGGAGAATCAACAAAGTAGTCTTCATAATCTTAGTTGGACCTTTGGTCATTTCCTGATCCACTAGTTTTCTATTATAGCTGAGAGCTTATTATTATTATTATAGAATCTTTATTAAGTGAGAGAAATGCTTACCAAGAGATTGAAAGGAGTACAAGTAAACACATTATTGTCCAGATTTTCCTTGGTTTGCTGAGTCGGATCCCTTTCATTTGGTCCATATTCTCTTGTTTTCTGATAGTCCTGCCCCTGTGGAGACTCACTTGGAAAACGAAGTTGAATGTCACAGGCACCAGAGACCCAAACACAGAGGCCCATGTGAGAAT

>Opossum_T2R30--Pseudo

ATGTACAATGCAATGAAGACCTTCTTCACAATCACAATAATTGGAGAGTTCATAATGGGAATTTTGGGCAATGGATTCATTGGACTGGTGAATTTCATTGACTGGATCAAGAAAAGAAAAATCTACTTAGTTGATTTAATTCTCACCAGCTTAGCCATTTCCAGAATTAGCCTGTTATTTATAATAATGCTAGATGGCCTTATATTGGTGTTTTTTCCAGATGAATATGCCAAGAGTACGTTCACGAAAATTATAGATGGCTTGTGGACAATTACCAATCATTTAAGTGTCTGGTTTGCTACCTGCCTTAGCATGTTTTACTGCTTGAAGATAGCCAATTTTTCCCACCCTCTTTTCCTTTGGCTGAAGTGGAGAATTAACAGATTGATTCTTATAATTCTACTGGCATCCATATTCATGTCTGTGTTAATTGTCCTTCCAATAACAGAGAAACTTAATGAAGATTTCAGGGGCCAGATAAACATAAAGAACAAGAGAAATAATACTTTGAAGTTAAAAGTGAGTAAATCTGAATACTTTGGGATCCATATTTTTCTCAATCTGACAACTCTTGTTCCCTTTACTGTATCCCTGATCTCATTTTTCCTGTTAATTCTTTCCATGTGGAAACATACCAGGAAGATAAAACTCAGTGCTATGGATGCTAGAGATCCCAGCACAGAGATCCATTTGAGAGCCATGAAAGCTGTAATCTCTTTCCTCATCCTCTTTGTTATTTATTGTTTGGCCTTTCTCATAGCCACATCCAGCTACTTTTTCCCAGAGAGTGAACTAGCAGTGATATTTGGTGAGATAATTTCAGCCATCTACCCCTCAGGACACTCATTTATCCTGATTTTGGGGAATATCAAACTGAGGCGGACATCCCTGAATGCACTACAGCAAATGAAGCATTGTCTCAAAGGAGAGAAATCCCTTGTGCCCTAGACCAACCTTTGCAGA

>Opossum_T2R31--Pseudo

GAGTTATTATATGTAATTGTGTTAAGTATTGAGTTTATTCTGGGGCTTTTGGTGAATAGTTCCTTTGACCCAATGAATTGTATCAAATGAGTCAAGAACAAGCAAATTTCTTTGTCTGATTTCATCATTACTAACCTGGATTTCTCCAGAATCTGCATACAATGGATTATCAAAATTAATAGTATCTTACTGCTATTGTATCCCTATTTACATTATTAATTTTAAAATACAAGAAATGTTTTCTACTCTTTGTGCAGTTTTTGGGCACTTGCTTTGTTGCCTGCTTCAGTGTCCTCTGCTGCCTTTTCCCCAGTCAATCTTCCTCTGGCTAAAGTAGGTTTCTGCATGCTGTTGGGTTCCATAGTCTACTCCTTTAGTGCAATATCACTGATCTATAAGTTTAATATTTATTCTGATCTAGGACAAATGAGACACAAAGTGATTTATATCAAATATGTGAGGAGAAAGGAAAAGAAATATCATGTCCCCCACTTCCTTGGTCTTCTTTAGACACTGCTCCCTTTTGCTGGGTTCCTGATCTCTTCTCTCTTGCTCATTTTCTCCCTAAGGAGACCCAATGAGGAGATGCAGCATCATGTAACTGGCTCTAGACACTCAAACCCGAGGTTCATGTGAAAACTATCAAGGACATCCTCTCTTTCTTCTTTCTCTCTCACTTTAAAAATATTTTGTTAC

>Opossum_T2R32--Pseudo

CTTGGCCAAGAGGTCTTGGGAATTTTACCTAAATTTAATCTTAACAAGAGCAAGAAAATGTCTTTGTCTGAATTTATCATTACCAGCCTGGCTATTTCTAGGATTTGTTTGCTCTGGGTATATAACTGATATTTTAATGTTGCTCTATCCCAGTTTTCATGAAATAAGCATAATAATGCAAATATTTTCTACATTATGGATTTTTGCTAACTTCCTAAGCATCTGGCTGGTCACCTGCTTGAGTGGACTCTACTGCCTGAAGATTGCCAGTTTCTCCCACCATGCCTTCCTCTGGCTAAAGTGGGAGGCTTCCCAGGTAGTTAAATGGATTCTGCTTGTATTTGTGCTCTTTTCTTTCTTTAGTACATTGTCAGTGATCAAAGAATTTGGGGCTACTTTTACTTAAAGACAAATAATACCCATAGCAAACTGCATGGAAGACATTAGGAAAAAGAAAAGTGCTTTTTTTTTTTTAAGTATGCCTTTATTTCCCTATGGATACCCTATCCTTGATTCCCTATATTCTGATCATACTATTCCTGGGAAGACACACCCAACAGATGTAGGACAATGACACTTGCTACGGTGACTCAAGCACCAAGGTTCACGTGAGAGTGACCAAAATCATCTTCTCCTTCCTTTTCCTTTTCATTGTATTATTTTGCTGCCTTTTTTTTTATCATTATATTGAACTTTTTACTGCCAGACACCAATTTGGCATAGATGACTGGGGAGACAATTGCAACTGTCTACCCTTCTATACACTCAATCATCCTCATTTTTGGCAACAACAAGCTGAAATGGGCATTGCTGGGGATGTTTCAGATA

>Opossum_T2R33--Pseudo

TTGTTTATAACCTTCATCGAGTTCTTTCTGGGAACTTGGGTAAATAGCTTCATTGTGTTGCTGATCTGTATTATTTGGGTCAAGACCAGGAAAATTTCTTTGTCTGACTTCATCCTCCTGAATCTGACTCTCCAGAATCATCCTGCAGGGGTTACTATTGTTAGATTGTGTTTTATCCATGTTCTATCCTCATTTATATGACTTAGGTATATTGCAGCAAATTGGTGGCATTTTCTGGATGTTTATCAATAATTTAAACATGTGTCTGACCACCTGCCTCAGTGTCCTCTACTGCCTGAAGATTGCCAATTTCTCCCACCAGGCCTTCCTCTGGTTTAAGTGGAGTGTTTCCTATGTGGTTGTCTAGATTCTTCTGGGCTCTGCTTTACTCTTTCTGAAACTCAATGTTTATTCTGGCTTCAGTCAAATGAAACTCTCAGGAAACTGCACTGAAGTCATCAAAAGAAAGAGAATTGAGTGTATTATGTCATGTATTTTCTTAGTGCTCTCTGTTCAGTCTTTCCCTTCTCTTTATCGCTGCTCTCCACTGTCCTGCTCATCCTCTCCCTGGTGATACATACCAAGCAGATGCAGCATCATGCCACTGGCCCCAGGGATCTAAGCACCATGGCCCATGTGAGAGCGACCAAAGTCATCCTCTCATCTCTCTTCCTCTTAATTGGATACTTTCTTGTCTTCTTTCTTGCTATGTCTAGTCATTTCTTGTCAGACCCCAAAGTGGCAGGAAGAACTAGGACCATAATTTTGTCTGCTTATCCCTCTATTCAAGCCTTTATTGTAATCCTGGAGAACCAGAAGCTAAAGCAGGTATTTCCTAGGATGTTCCAGGTC

>Platypus_T2R5--Pseudo

AACCCCATCTCCTTCTGGCAGGCCGCGGGGCTCGGCGTCTTCCGCTGCGTGAAGATCGCCACCTTCACCCACCCCCCCCCTTCCTCTGGCTGAAGCGGAGGATCTCGGAGCTGGTGCCCCGGTTCCTCGTCGGTTCCCCGCCGATCTCCTTCGCGTCCTCCCTCCCCTTCGTCGTCGGCTACTTCGCCGTCTCCTCCCGGAGGAACGTCTCGGGGAACGGCACCGAGGCGGAGGGGAACCTCTTCCGGCGGCACCATCTCCCGTGCTCCGCGGTCATCGCGCCGTGCGTCCCGTTCCTCCTGCTCCCGGTCTCCTCCGTCCCGCCGATCGTCTCCCTCCGGAGACACCCGGGGGCCCTGCGGGACCGCGGCACCGAGGCTCCCGCCCGGGCGCTCGCCTCGCCGACCCCCTTCCTCTCCTTCTACATCCCGTATTTCGCCGCCCTGAGCTTCCCGGGGCTCCTCATCTTTCAGTTTGACGGTGTCGGGATGTGGCCGGTCCAGCTGGTGGCTT

>Rat_T2R37--Pseudo

ATGGTATCTGTCCTGCACAGCATCTCCACCATTATAATAATCACAGAGTTCATTTGGGGAAATTTAAGCAATGGTTTGATAGTGCTGAAGAACTGTCTTGATTGGATCAATATAAAAGAGCTTTCCACACTTGATCAGATACTCATTCTCTTGGCAATTTCCAGAATTAGTCTCATCTGGGAGACATTACTCATGTGGGTTAAAGATAAATTAGTTTCATCTATTACCATCGAAGAATTAAAAATGATTATGTTCAGTTTTATGCTATCTAGCCACTTCAGTCTCTGGCTTGCTACAGCTCTAAGCACCTTCTATTTATTCAGAATAGCTAACCACTCCTGGCAGATCTTTCCCTACTTAAAATGGAGACTAAAACAGCTGATTGTGCAGATGCTACTGGGAAGCGTGGTGTTCTTGATTGCAAATATAATACAAATAACCATCACTCTTGAAAAGAGGTTCTATCAATATAAAGGAAACACAAGTGTGAATTCCATAGAGAATGAGTTTGCACTTTTGATATAGATGATGTTCTTTAACATGACTATGTTCTCTGTGATACCACTTTTATTGGCCCTGATTTCTTTTTTTCTGCTAATCTTCTCTTTGTGGTAACATCTCCAGAGGATGCAGCTCAATTCCAGAGAAGATAGAGACCCTAGTACCAAGGCTCACAGGAATGCCCTAGGAATTATGGTCTCTTTCCTCTTGCTCTATACTATGTATGTCCTCTCTCTTCTTATATCATGGATTGCTCAGAAGAATCAAAGTGAACTGGTTCACATTATTTGTATGATAACTTCATTCTTGAATCCTTCAGTCCACTCATCTATCCTGATTCTGGGTAACTGTAAATTAAAGCAGAGTACTCTTTGTATACTGAGGCAACTGGGATGTAGGCTGAAATCACAGAATACAGCAACTACA

>Rat_T2R38--Pseudo

ATGTCCAGCCTACAGGAGATTTTGGTTGTGATCTTTTCTGTTATAGAATTCATAATGGGAACTTTGGGAAATGGATTTATTGTACTGATAAACAGTACTTCCTGGTTCAAGAGTCGGAGAATCTCTGTAATTGATTTTATTCTCACTTGCTCGGCCATCTCGAGAATGTGTGTTTTGTGGACAACAGTTGCAGGTGTCTCTTTCTACAAGGCATTATTTTACTTTAAAAGTTTGCAAGTATTTTTTGACATTATCTGGACAGGATCCAACTATTTATGTACAGCCTGTACAACCTGCATCAGTGTCTTCTACTTGTTCAAGATAGCCAACTTTTCTAATCCAATTTTCATCTGGGTTAAACAGAGAATTCATAAGGTGCTTCTGTCTATTGTTCTAGGAACAATCATCTATTTCTTCTTATTTCTCCGACTGGACAAAATTGGAACAAAACAAAACATTCACTTTTTTAGATACTCTAACTGGTTTCTTAGTCTACAATGTGATTCTCATATTTTTTTTTTGCAGTGTCTCTGACATCGTTTCTTCTTTTAATCTTCTCTTTACGGAGCCACCTCAGGAGGATGAAACTACAGGGCATACATACCAAGGACACAAGCACAGAAGCACACATAAGAGCTATGAAAATTATAATTTCATTCCTCTTGTTCTTCATCATATATTATATCAGCAACACTATGCTTACTTTTTCACATTCCATTCTTGACAATGTGGTTCCAAAAACTTTCTCTTATATCCTAACATTTATGTATTTATCTATTCATCCCTTTCTCCTGGTTTTATGGAACAGCAAATTGAAATGGGCATTCCAGTGTGTATTGAGAAAGCTAGTGT

>Rat_T2R39--Pseudo

TTTTTCTTGATCATTTTCTCTCTGTGGAAATATCTGAGGAAGATGCAACACAGAATGTAATAATGCAGAAACCCTATCACCCCTACCTACATCAGAGCCTTACACACAACAGTCACTTTCCTCATTGTGTATGTCATTTTCTTTCTTCCTCTTGTTTAAGTTTGGGATTCTCTGTTTCTGGAGAGAAAGCTGTTGCTTTTGTTCACAAAGGATGCA

>Rat_T2R40--Pseudo

AACTCTATATTTGTGAGCATCCCCTTTGCTGTGTCACTGGTGGCTTTTTTCCTGTTAGTTTTCTCCTTGTAAAAACATAAGAAAAAGATGCACCACAATACCAGGGGTTCCAGAGACTCCAACACCAAGGCCCACATTAAAGCCTTGAAAACTGACTTGCTCTCCTTTCTCTTTTTGCCATTTTTTTTTACTTTCTCTTGTTATAAATGTCTGCAGCATTAATTACAGGAGAATTACATAATATTTTTTTTGTTTAGGTTATTTACTGGAATAGCTTTTGCTTCACGCCACTCCTATGTCCTGATTCAGGGAAACACCAAGCTGAGGCAGGCTTCTCTTTCTGTACCCTGGTGGATGAGGTGCAGGCCCAAAGATGTGAGAACC

>Rat_T2R41--Pseudo

GTAATTGGCAACTTTGGCAATGGATTCATAGCAATGGTGAACATCACAGACTTAGTCAAGAGAAGAAAGATCTCTTCAGTGCTCATTGGATCAGACCATCACCGTTCTGGCCATCTCCAGAATTGTCCTGCTGTGGTCAGTATTAGTGAGCTGTTGGCTATATATGTTCTACCCAGGAAAATGGATGACAGAGAGAATTGTTAGGAAAATACTTAGTGTAGGGACAACATTCAACCAGATTTGCCTCTGGTTTGCTGTAAGTCTCAGCATCAAGATAGCACTATTTTTCCAACACTATTTTCCTTTATTTGAAGTTCAGTTAGAAAAGTAATGACAGGGACATTGATAATGTCTCTGCTTCTCTTGGTCTTAACTAGTACAGTTACTAATGTACTCGAGAACATTTAAATCCCTGAATATGTCTAACAGCTTGATTTTAAATAGCACACAGATTTCTATGCTGTTTTCATTGGCAAACACCTTATTTGTGCCCATACCACTTATTGTGTCACTGGTCACGTTTCTCCTGCTATTCTTTTCCCTGTGGAAACATCAGAGGAAGATGCACCACGGTGCCCAAGGATGCAGAGTTGCCAGCACCAAGGCCCACATCAGCGCCTCTGAGACAATGATTGCCTCCATCCTCCTGTATTCCATTTTCTTTCTGTCTCTTCGTGTCGAGGTTTGGAGTTCTCTGCTTCTGGAGAGAACGCTTTCACTTTTGATCACACAGGCTGCAAGTATTGCTTTTCCCTCACTGCACTCCGAGGTCCTGATTCTGGGCAATGCTAAACTGAGAAAGACTCCTTTTTCTTTCCTCCTGTGGCTGAGGTGTTGGCACAATGATGGAAACCGTAGAGTGCACAGACCTGCAGTTCATTTATGTGGATCATC

>Frog_T2R64--Partial

ATGATGTCCGTGGTCATGACGGTGATACTGATTGTAACATGGCCATGTGGGACTATCCTGAACTCATCCATTATAGCTGTCTATCTCAGCGACTGGAAGAAGGGAGTGAAACTTGGGGAGTGCGATCAGATCAGTCTCAGCTTGGGGTGCACCAACATTCTCTTGCAGTGCTTCACAACATTTGGAGTGGCATCCATATCATATGGACTATGCTTCCGCTAT

>Frog_T2R65--Partial

TCCCTGGCTTTCTCCAACATGGTCTTTTCTGTCACAGATGGAGTCTATACTATATGCATCCTTGTTATCCCATTTGGTGACGTTGAAGATCAATTGTATCTGCTATATATTGTCATGGTGTATGTGCTCTTCTGCAATTCCTGGCTCAGCGCCTGCCTGTGTTTCTACTACTTTGTGAAAATCAGTAACGTTAAGCCTGGTTACCTGGCACGACTCAAATCAAAGATCAACACCCTGGTACCAAGGCTAATACTGGTTGCTCAGGGGTTCTCCATCTTGAACTCACTTTTTTACATGCCGGTGTTTTTTAAAGAGAACGTTGAAAACCCAACCAACCAGACACAAAAAATTACTACTTACAGAACAGATGATTTTTATAATGTATTTTTCTTGCTGATCAATTGTTATATTCCTTTCCTGGTTATAGTGGTCACCACCAGCCTTATCATTGCCTCGCTCTACAAGCACACTCGTCATATGCAACAGAACATGGGAGAGTTTGGTGGCCCCAGCCTGAAGATTCATCAACGAGCAGCACGTACAATGACGTCTCTTCTTATTCTCTATCTTCTGTTTTACGTTGTAGTACTTGGGTCTAGCTTACTTATAAAAAATGAGTTGTTGAGTTGGGTTTACTACTTGGTGGGTTGTACCTTCTCCCCCATTCAGTCCATCATTCTAATTATGGGGAACACCAGACTGAAGAAGACCTTTGTGAACATGCTAAACAGCTGTAGGAAAACATTTGGAGAAGAAGACAATGTAACCACTTAA

>Frog_T2R66--Partial

ATGCTGTCTGCCATTCAGATAATAAAAACAATTATTCTGATCATAACCGGATCATGTGGGCTCATCCTAAACTCATGGATTGTAGCTGTGCATCTCAGCCATTGGAAGAAGGGAGTGAGCCTTGGGGACTGTGATCAAATCATTCTCATCAAAGGGGTCACCAACGTTCTCCTCCAGTGCTTAGTAACTTTCAATGGGATACTTATAAACTTTCAGCTGAATGACTATTTTGACAAGGAATTCCTTTATGTGACTAACATTGTCTTCTTCTTCCTGACTTCCCTCTGGAACTGGCTCACTGCCTGGCTCGCTATCTGCTACTGCTTCAGACTCGGCAACATTTCACATCGGGTCTTTATTGGC

>Lizard_T2R48--Partial

CAGTTGAATTCTATGGCCTGCTACTTTTTGCCTCTCTTTAATTCTTCAAGTATAAGAAAAATGTTTTTTTTCTCCAGGGTCTTTCTTAATATGGCCAACCTCTGGTGTGTCTCCTGGCTCAGCATTTTCTACTGTGTGAAGGTCATCAACTTTTCCAACTCTTTACTCCTTTGGCTAAAGTTAAGGATCAATCTGCTTCTACCCAGAATACTCGGAATATCAATGGTCATTTTTATGGTCATTTCTCTTCCTTCCATCTTCACATTTCATGAATGCAACAAATCATGTTTTTTTTCACTTTTTTTATTCCTTCCTCCTCTATATATTTTTTTTCCAATTTTTTTTATTACATTCCAACAATTAACAAAAATTTTGCGATGCATGTCTTATTTCAATATACATAATACCAAAAGTAAGTAA

>Lizard_T2R49--Partial

ATGGCCAGTCTCTGGTGTACCTCCTGGCTCAGCATTTTCTACTGTGTGAAGGTCATCAACTTTTCCAACTCTTTACTCCTTTGGCTAAAGTTAAGGCTCAATCTGCTTCTACCCAGACTACTTGGAATATCAATGACCATTTTCATGGTGTCTTCAGTTCATTCCATCTTCAGGTTTTTCAAATACAAAGAACCATGTAATCAGACTGTAACACCACTAACTAATGAGGATACTGACATCAGCATGTGGATTAGTTTTATTCCAGTGCAGATAACTTTCACTTGCATAAATTTCAGCGTGAACATAGCGGCAACCCTTCTTTTGCTCATCTCCTTGTGGAGGCATGTGAGAAACTTCAGAGAGAATGATACTAGTGTCCAGGACCTCAACACTCAAGTCCATCTCAAAGTCATAAGGCCTTTGTTGATCACGCTCTTACTCTACATTTTATTTATTGTTGGTTTGATAACAATTATGAGTGGCTTTTTTGATTTGCAAACAAACCAAGCACTGATTGACGATATAATGATTACCCTATTTGCTTCAGTGCATCCCATAATATTAATATGGACCAATCCAAAACTCAAAAATGTGGCTGCTCACATGTTAAACATCAGGCAAAGACCTTAA

>Macaka_T2R38--Partial

TTGGCTTGTCATCTTTTTGTCATAAACATGAATCAGATTGTACAGACAAAAGAATATGAAGAAAACATGACTTGGAAGATCATATTGAGGAATGCGATTTATCATCCAGATATGACTGTAACCACGCTACAGAACTTAGTACCTTTCACTCTGACGCTGATATGTTTTCTGCTGTTAATCTGTTCTCTGTGTAAACATCTCAAGAAGATGCAGCTCCATGGCAAAGGATCTCAAGATCCCAGCACCAAAGTCCACATAAAAGCTTTGCAAATTGTGATCTCCTTCCTCTTGTTATGTGTCGTTTACTTTGTGTCTGTAATTATATCAATTTGGAGTTTTGAGAGTCTGGGAAACAAACCTGTCTTCATGTTCTGCCAAGCTATTAGATTCAGCTATTCTTCAGCCCACCCATTCATCGTGATTTGGGGAAACAAGAAGCTAAAGCAGATTTTTCTTTCAGTTTTGTGGAACGTGAAGTACTGTGTGAAAGGACAGAAGCTTTCATCTCCATAA

>Opossum_T2R34--Partial

TCTTTTTTTTGTATTCTAGATTCTATCAAAATATGTGCTTATGACACAGCAGGGTACCTAGAACCCACCTGGACTATGATCAATCTTTCAAAAACCTTGTTTGCTGCTTGCCTCAGACTGTTCTCCTTTCTGAAGATTGCCAGCTTCTCCAACCCCATGGTTCTCTGGGTGAAGTGGAGAATTAACAGAGGTATCTTCAAGATGCTAACGGGCTGTTTACTCATCAACTGCTTTGTTTGCCTGCCAATGGCAAAGCAAATGATCAATGAATACAAATGTCATTTAGCTCAAGGAAACCAAAACATATAA

>Opossum_T2R35--Partial

TTATGATATATTTCCTCATTCTCTATGTTCCTTACTCCATTGCTCAACTGGTTATCTTTCTCCCTTCCTTTGGGATTAGAAATAATTGGATCAGAAGCATATTGATAATAGTCAATTGTACTTACTCTCCAGGACATACTATTTTCATTATCCTATTGCATCCTAAACTCAAAGCAAGAATAAAGAGCATTCTATGGTGCAATTGA

>Opossum_T2R36--Partial

ATGACAAATTTTATATTAATAGTAGTTTTGTTTGTGACCTCCATTATTTTCTTTCTGGATGCTTGGATAAATGGCTCCATTGTGTTGCTGCGCTGTATTACTTGGATCAGGGACAGGAAAATTTCTTTGTCTGACTTTATCATCCTGAATCTGGCTCTCTTCAGGGCCATCCTGCATGGGATACCATTGTTGGTTTCAGCTGTGACCATATTTTATCCCCAATTTCGTCACTTGGATATATTTTTGCATATTGTTGATATTTTCTGGATGTTTACCAATAACGTAAACATCTGTCTGACCAGCTGCCTCAGTGTCCTCTACTGCCTGAAGATTGCCAATTTCTCCAACCAGGCCTTCCTCTGGCTCAAGAGGAGAATATCTTGTGTGGTTGTCTGGATTCTTGTGGGATCTCTGCTCTATTCCTGCTTCAGCACACTGTCAGGGGTCTTGAAATTCCATTTTTATTCTGACAAGAGTCAAATGATATTCTCAAGAAACCACACTGAAGAAATCAAAAGAATAAAAATGCACTATTTTCTCTTGCATTTTCTTGGTACTCTCTGGTCACTCATTCCACTCTCCTTGTCCCTGGTCTCCTCTGTCCTGCTCATTCTCTCCCTGGTGAGACATACCAGGCAGATGAAGCATCATTCCACTGGCACCAGGGACCTCAGCACCATGGCCCAT

>Platypus_T2R6--Partial

ATGCCCTCTGTTGTGGCGAAGAGACTGGTCATAGCTGTGGCCGTGGTCGAGCTGGTGTCCTCCCTGGGGATAAATGGCTTCATACTGGCCGTGAATGTCCTGGACTGGGTGAGGGGAAGCCAGCTGACCTCCTGCGACCTGCTGCTGAGTGGCCTTAGCGGCTCCCAGCTCTGCCGGCAGTGGTTCCTGATGCTGGACTGCTTCACTGTCTGCGGGTACCTGACCATGTCCCCGCTGGAGGATTGGGCCACCACTGTACTCTGGCAGCTCACGAGCCAGGCCAGCCTCTGGCTCGCCGCCTGCCTCAGCCTCCTCTATTTCCTGAAGATTGGCACCTTCACCCACCCTGGGTTCATGTGGCTGAAATGCAGGGTGCCCAGGCTGGGGCTGCAGCTGCTTCTGGGCTCCTTGCTGGTTTCTGCCGTCTCCACCCTCACCCTGCTGCTGGTGGCCTCCAAGGCAGCACCCTGGGGAGCCTTCCAGGGAGAGTCCAGGAACTTCTCATACCTGCTAGACTGGAGAAGGAAGTCAAACATCTACCTCCCTCTGCTCCTGAGCCTGCAGTTCCTTCTGCCGCTGTCCATCTTTCTGGTCTCCATTTCCCTGCTGGTC

>Rat_T2R42--Partial
[truncated: 721 more chars]
