# Supplementary figures and images for: Dynamic evolution of bitter taste receptor genes in vertebrates
Source: BMC Evol Biol. 2009 Jan 15;9:12. doi: 10.1186/1471-2148-9-12 (PMC2646699; doi:10.1186/1471-2148-9-12)

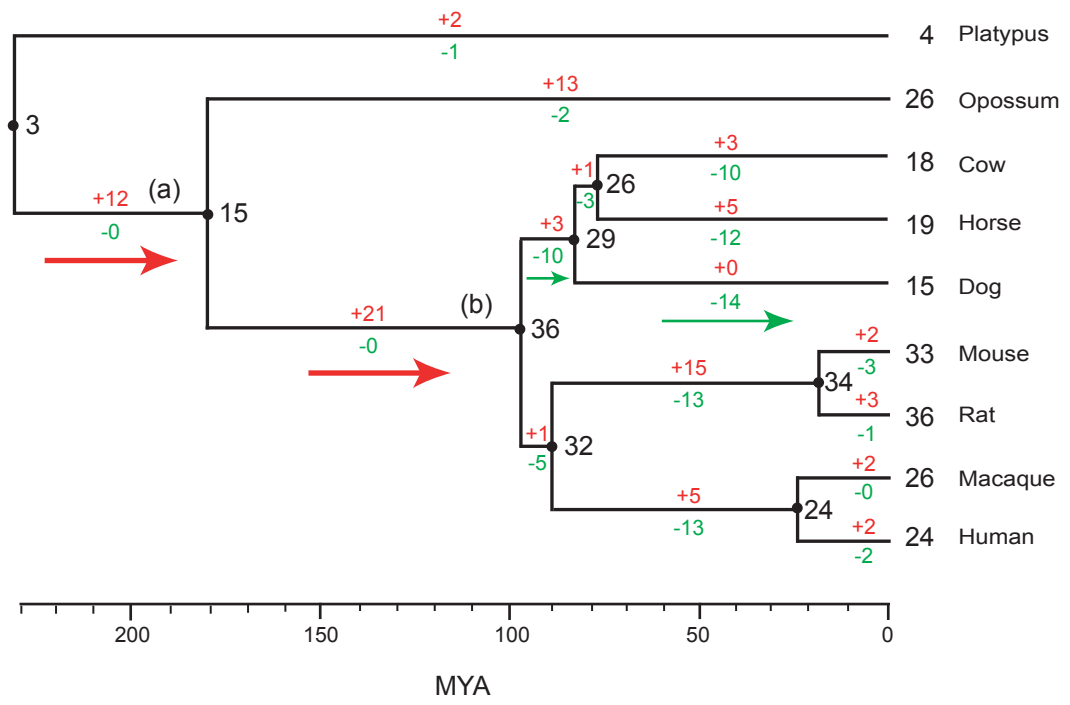

Supplement: Additional file 2 — Supplementary Figure 1. Evolutionary changes in the number of T2R genes in mammals. The phylogenetic tree contains the Euungulata (Perissodactyla + Cetartiodactyla) clade based on the report by Waddell et al The divergence times were based on those reported by Waddell et al. and Murphy et al. [file 1471-2148-9-12-S2.pdf]

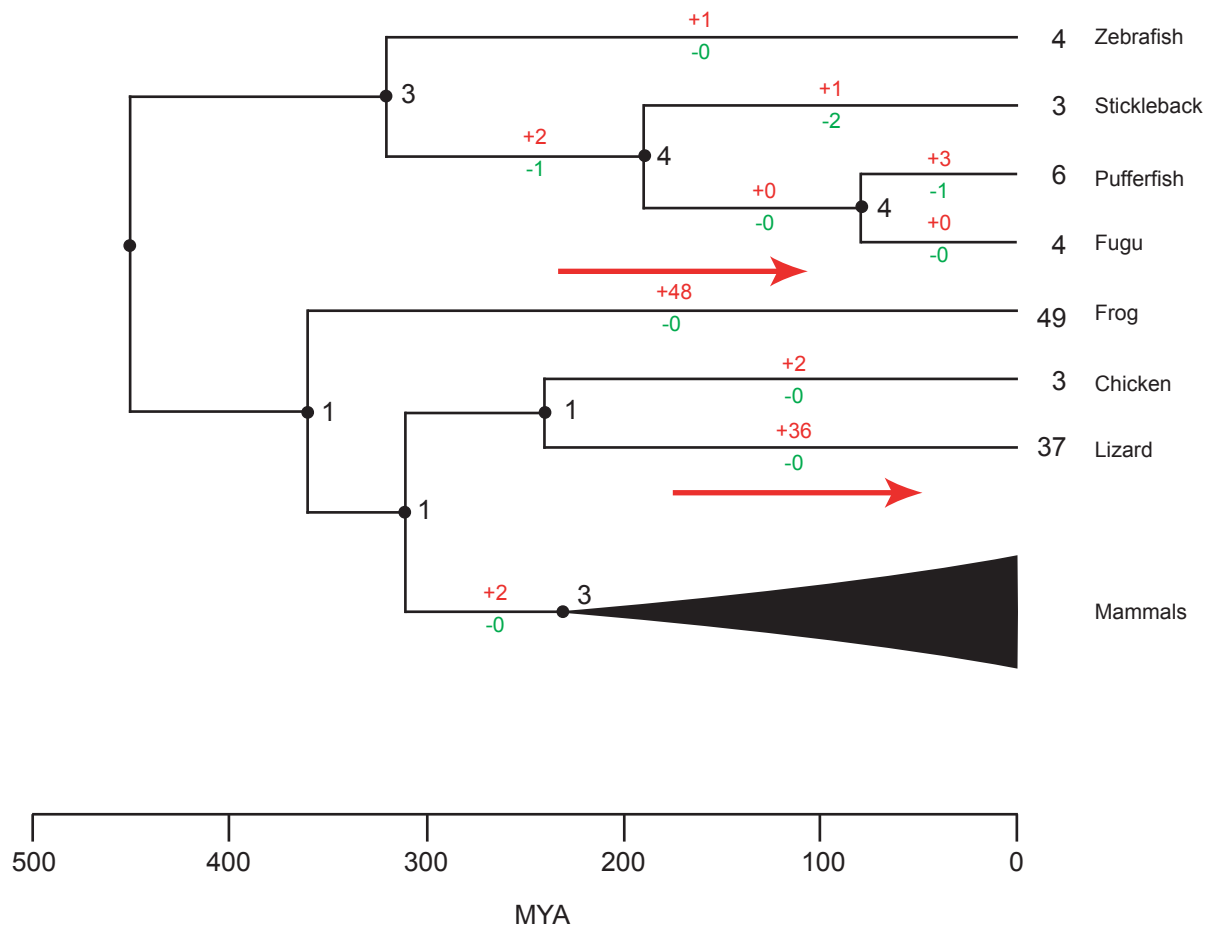

Supplement: Additional file 3 — Supplementary Figure 2. Evolutionary changes in the number of T2R genes in teleost fishes, the frog, lizard and chicken. The phylogenetic tree was obtained from Benton, and the divergence times were based on those reported by Hedges et al., Janke et al. and Aeschlimann et al Separate analyses were performed for teleost fishes and tetrapods. [file 1471-2148-9-12-S3.pdf]
